# Electrocatalytic Polarity Inversion of Azlactones: Access to α‐Quaternary Amino Acids with Electron‐Rich SOMOphiles

**DOI:** 10.1002/anie.8095368

**Published:** 2026-08-26

**Authors:** Paula Rodríguez, David Buedo, Alberto Vega‐Peñaloza, Kian Gosling, Stuart C. D. Kennington, Bart Limburg, Xavier Companyó

**Affiliations:** ^1^ Section of Organic Chemistry Department of Inorganic and Organic Chemistry University of Barcelona Barcelona Spain; ^2^ Institut de Química Teòrica i Computacional (IQTC) Barcelona Spain

**Keywords:** azlactones, electrocatalysis, quaternary amino acids, SOMOphiles, umpolung reactivity

## Abstract

The use of azlactones as nucleophiles is a well‐established strategy to access α‐quaternary amino acids. Herein, we present an electrocatalytic approach that reverses the natural polarity of azlactones. Indirect oxidation of the azlactone enolate, using triarylamine electrocatalysts, generates an electrophilic radical intermediate that couples with a variety of electron‐rich SOMOphiles. This strategy enables Csp^3^─Csp^2^ and Csp^3^─Csp^3^ bond formation at the α‐position of the azlactone, providing access to arylated, β‐oxoalkylated, alkenylated, and allylated quaternary amino acid derivatives.

## Graphical abstract

Electrocatalytic polarity inversion of azlactones unlocks their coupling with electron‐rich reaction partners. Triarylamine‐mediated oxidation of the azlactone enolate generates an electrophilic radical intermediate that undergoes selective C4 functionalization with diverse SOMOphiles. This electrocatalytic platform enables access to arylated, β‐oxoalkylated, alkenylated, and allylated α‐quaternary amino acid derivatives.

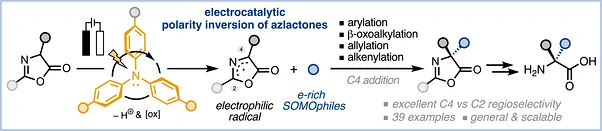

## 1 Introduction

Unnatural amino acids are fundamental building blocks in modern organic chemistry, with widespread application in drug discovery, protein engineering, and material science [1, 2, 3, 4]. Amongst these, α‐quaternary amino acids constitute a particularly valuable motif, as they enhance chemical stability and enforce well‐defined peptide conformation [5, 6, 7, 8]. Despite significant advances in the synthesis of unnatural amino acids [9, 10, 11], access to their quaternary analogues remains challenging due to the steric congestion around the α‐carbon [12].

Azlactones are an important class of amino acid surrogates. Beyond their growing interest in medicinal and materials chemistry [13, 14, 15], the presence of multiple reactive sites and broad reactivity profile make them versatile platforms in synthetic chemistry [16, 17, 18]. In this context, the use of azlactones (**A**) as nucleophiles is a well‐established strategy to access α‐quaternary amino acids. Upon deprotonation, the enolate **B** features two distinct nucleophilic sites at C2 [19, 20, 21, 22, 23, 24, 25, 26] and C4 positions [27, 28, 29, 30, 31, 32, 33, 34, 35, 36, 37]. Regioselective C4 addition of **B** to electrophiles affords **C**, key precursor to α‐quaternary amino acids (Figure 1A).

**FIGURE 1:**
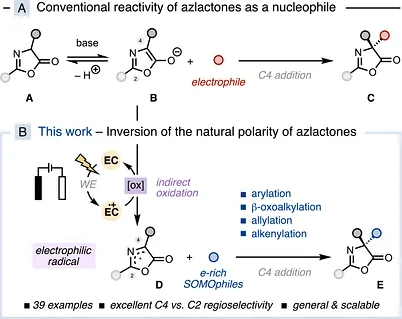
(A) Conventional reactivity of azlactones with electrophiles. (B) Electrocatalytic polarity inversion of azlactones allows its functionalization with electron‐rich coupling partners.

Despite significant progress in azlactone functionalization, most methods remain limited to polar reactions with electrophiles [16, 17, 18, 19, 20, 21, 22, 23, 24, 25, 26, 27, 28, 29, 30, 31, 32, 33, 34, 35, 36, 37], prompting recent efforts to expand their reactivity profile. In this context, Ohshima and coworkers reported an iron‐catalyzed cross‐coupling of azlactones with indoles via the transient formation of an azlactone homocoupling dimer [38, 39]. More recently, the groups of Zheng [40], Lu [41], and Zhan [42] independently developed photochemical alkylations of azlactones with redox active esters proceeding via radical‐radical coupling. Overall, strategies beyond nucleophilic additions and radical‐radical couplings remain unexplored to date.

Herein, we report an electrochemical [43, 44, 45, 46] strategy that enables the inversion of the natural polarity of azlactones, thereby unlocking their C4 functionalization with a broad range of electron‐rich coupling partners (Figure 1B). This umpolung reactivity is achieved through the electrochemical indirect oxidation [47, 48] of the azlactone enolate **B**, mediated by triarylamine‐based electrocatalysts, to generate the electrophilic radical intermediate **D**, that is subsequently intercepted by diverse SOMOphiles. Modulation of the residue at the C2 position of the azlactone proved crucial for governing the regiochemical outcome of the C–C bond formation. The protocol enables the construction of Csp^3^─Csp^2^ and Csp^3^─Csp^3^ bonds at the quaternary center, providing access to arylated, β‐oxoalkylated, allylated, and alkenylated α‐quaternary amino acid derivatives **E** in good yields and with excellent regiocontrol. The electrochemical platform allows the functionalization of a range of proteogenic and non‐proteogenic amino acid, along with the incorporation of biologically relevant molecules into the amino acid core. In addition, the method operates across diverse electrochemical setups and is readily scalable to 1 mmol, underscoring its practicality and robustness. This work establishes electrocatalytic umpolung of azlactones as a novel, sustainable, and practical platform for the construction of α‐quaternary amino acids.

## 2 Results and Discussion

### 2.1 Reaction Design and Optimization

We selected the valine‐derived azlactone **1a** and 1,3‐dimethoxybenzene (**2a**) [49, 50, 51] as model substrates to develop the cross‐dehydrogenative [52] azlactone arylation via electrochemical umpolung (Figure 2). Cyclic voltammetry (CV) analysis of **1a** in MeCN showed no oxidation within the solvent redox window (red traces, Figure 2Aii). Commonly used strong bases, such as alkoxides, amidines, and guanidines, were unsuitable to promote enolization of **1a** owing to their low oxidation potentials (*E*_ox_ < +1.00 V vs. SCE). Given that azlactones exhibit an aqueous p*K*_a_ ∼ 9 [53, 54], we hypothesized that collidine (p*K*_a_ of collidinium in water = 7.5) [55] would be capable of promoting enolization of **1a**, while remaining non‐oxidizable at the working potentials (grey trace, Figure 2Aii). CV of a mixture of **1a** with collidine showed an oxidation wave with *E*_p_ = +1.30 V vs SCE that we ascribed to its enolate **6a** (green trace, Figure 2Aii). We screened different types of electrocatalysts, identifying triarylamines [56] as the best suited for the indirect oxidation of **6a**. CVs of a solution of **1a** and 5 mol% of tris(4‐chlorophenyl)amine **3a**, upon addition of increasing amounts of collidine, showed a catalytic current consistent with homogeneous single‐electron oxidation of **6a** to **7a** by the oxidized electrocatalyst **3a^·+^** (purple traces, Figure 2Aii). Importantly, no indirect oxidation of the radical trap **2a** (*E*_p_ = +1.67 V vs. SCE, blue trace, Figure 2Aii) was observed [57]. Having established the feasibility of the electrocatalytic system, we optimized the cross‐dehydrogenative umpolung arylation of azlactones (Figure 2Ai). Electrolysis of a mixture of **1a** and **2a** with 5 mol% of **3a**, 50 mol% of collidine, and 4 equiv. of HFIP as proton source, was conducted at 5 mA using an RVC anode, a platinum cathode, and TBAPF_6_ as supporting electrolyte. This furnished the C4 arylated product **4a** in 25% yield, C2 arylated product **5a** in 8% yield, along with side‐product **8** –arising from azlactone ring opening by hexafluoroisopropoxide– in 46% yield (Figure 2Ai, entry 1 & box 1). We hypothesized that collidinium·BF_4_ [58], having a non‐nucleophilic conjugated base, could serve as a proton source suitable to suppress the undesired side reaction, and simultaneously function as supporting electrolyte. The reaction with 2 equiv. of coll·HBF_4_ yielded product **4a** in 45% yield without evidence of azlactone opening, albeit still with a moderate 3:1 C4/C2 ratio (Figure 2Ai, entry 2). This regiochemical outcome is consistent with the delocalized spin density of radical **7** across C2 and C4 positions [40, 41]. We reasoned that increasing steric hindrance at C2 could steer the reactivity toward C4. Whereas the use of azlactone **1b**, featuring a *tert*‐butyl group at C2, completely precluded reactivity (Figure 2Ai, entry 3), bulky aromatic substituents at C2 greatly improved regioselectivity at expenses of reactivity. For instance, 2‐mesityl azlactone **1c** afforded **4c** in 24% of yield with complete regiocontrol (>20:1 r.r., Figure 2Ai, entry 4), while the *o*‐tolyl azlactone **1d** yielded product **4d** in an improved 41% yield and 16:1 r.r. (Figure 2Ai, entry 5). Considering that the valine‐derived azlactones **1a**‐**1d**, featuring an isopropyl group at C4, are amongst the most sterically hindered azlactones that can be accessed from proteogenic amino acids [59], we next examined azlactones derived from alanine (**1e**) and leucine (**1f**). For these less sterically congested amino acid surrogates, the *o*‐tolyl moiety at C2 imparted excellent regiodiscrimination, affording products **4e** and **4f** in 68% and 59% yields, respectively, as a single C4‐arylated regioisomer (Figure 2Ai, entry 6–7).

**FIGURE 2:**
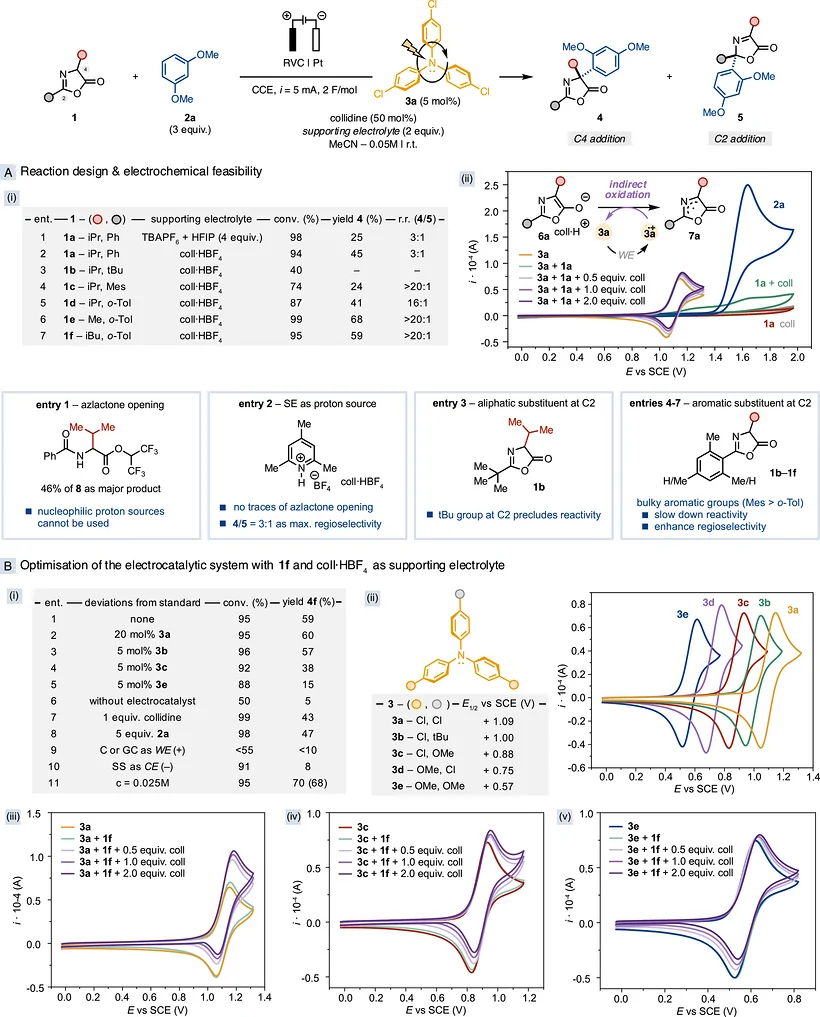
(A) (i) Reaction design. Yield and r.r. determined by ^1^H‐NMR using 1,3,5‐trimethoxybenzene as internal standard. (ii) Electrochemical feasibility. **3a** (5 mM), **1a** (100 mM), collidine (50 mM aliquot addition). (B) (i) Reaction optimization (ii) Library of electrocatalyst **3a**–**3e** used (5 mM), (iii–v) Catalytic current experiments: **3** (5 mM), **1f** (100 mM), collidine (50 mM aliquot addition). All CVs performed in MeCN with TBAPF_6_ (100 mM) at scan rate of 100 mV/s.

Having identified a C2 substituent capable of ensuring high levels of regioselectivity, we optimized the remaining parameters of the electrocatalytic system with azlactone **1f** (Figure 2B). Increasing the loading of **3a** to 20 mol% did not improve the reaction yield (Figure 2Bi, entry 2), suggesting that the electrocatalytic performance is governed by the available concentration of **6**. Next, we synthesized a series of *p*‐substituted triarylamines **3** encompassing a wide range of oxidation potentials (Figure 2Bii). Sequential replacement of chlorine atoms in **3a** with electron‐donating substituents shifts the oxidation of the corresponding electrocatalysts **3b**–**3e** to lower potentials, from +1.09 V for **3a** to +0.57 V for **3e**, while maintaining reversibility. Indirect oxidation of the enolate **6f** was observed for the entire series of electrocatalysts **3a**–**3e**, yet the efficiency of the homogeneous electron transfer depends on the potential difference between enolate **6f** (*E*_p_ = +1.30 V) and the electrocatalyst **3** [47, 48]. While **3a** exhibited the highest catalytic current (Figure 2Biii), other catalysts —such as **3c** (Figure 2Biv) and **3e** (Figure 2Bv)— were also capable of oxidizing **6f** albeit to a lesser extent. These observations correlate with the outcomes of the electrochemical arylation of **1f** using these catalysts (Figure 2Bi, entries 3–5). As the indirect oxidation of the azlactone enolate becomes less favorable, turnover of the electrocatalyst becomes less efficient, leading to accumulation of the oxidized catalyst (**3^·+^**) and depletion of neutral **3**, which subsequently decomposes during the reaction. To maintain the constant current, the cell potential increases, promoting direct anodic oxidation of the azlactone. This results in high substrate conversion but low product formation. Indeed, in the absence of electrocatalyst **3**, product **4f** was formed in trace amounts, highlighting its essential role for productive reactivity via indirect oxidation of **6** (Figure 2Bi, entry 6). Increasing the concentration of collidine or the equivalents of **2a** did not lead to any significant improvement (Figure 2Bi, entries 7–8). Alternative carbon‐based anodes and stainless‐steel cathode yielded only trace amounts of **4f** (Figure 2Bi, entry 9–10). Finally, by diluting the reaction to 0.025 M, the arylated azlactone **4f** was isolated in 68% yield (Figure 2Bi, entry 11).

### 2.2 Generality of the Electrocatalytic Methodology

Next, we assessed the generality of the electrocatalytic umpolung arylation of azlactones **1** with **2a** (Figure 3). The sterically demanding valine‐derived azlactone produced the α‐arylated azlactone **4d** in 58% yield with high regioselectivity (16:1, C4/C2). Substrates derived from alanine and leucine yielded products **4e** and **4f** in 79% and 68% yield, respectively, and complete C4 regiocontrol. The structure of product **4e** was unambiguously confirmed by X‐ray diffraction analysis [60]. The hindered azlactone derived from isoleucine afforded product **4g** in 62% yield, 10:1 r.r., and 2:1 d.r., whereas product **4h**, arising from the non‐proteogenic amino acid norleucine, was isolated in 77% yield with exclusive C4 regioselectivity. The electrochemical protocol also tolerated aromatic substituents at C4. Azlactones derived from phenylalanine, tyrosine, and tryptophan produced products **4i**–**4k** with yields of 62%, 48%, and 62%, respectively. Despite the presence of potentially oxidizable aromatic side chains, the electrocatalytic platform selectively produced the arylated azlactones with no competitive oxidation. Additionally, phenylglycine underwent efficient arylation to produce the α,α‐diarylated azlactone **4n** with >20:1 r.r. and 79% yield. Sulphur‐containing amino acid derivatives, including methionine‐ and cysteine‐derived azlactones, were also compatible with the reaction conditions, yielding products **4l** and **4m** in yields of 62% and 77%, respectively. Finally, arylated azlactone **4o** bearing a cyclopropyl substituent at C4 was obtained in 54% yield with no evidence of cyclopropane ring opening. This observation is consistent with the delocalized spin density of **7** [40, 41]. Next, we investigated the influence of the aromatic substituent at C2 using alanine‐derived azlactones under optimized conditions (Figure 3). Phenyl‐, mesityl‐, *p*‐tolyl‐, and *m*‐tolyl‐substituted azlactones participated efficiently in the electrocatalytic arylation catalyzed by **3a**, affording products **4p**–**4s** in 55%–70% yield with complete regiocontrol. However, substantial electronic variation at the C2 aryl group required an adjustment of the electrocatalyst to better align with the oxidation potential of the corresponding enolate intermediate. For instance, since the electron‐rich *p*‐methoxy‐substituted azlactone oxidizes at a lower potential, the *bis*(4‐methoxyphenyl)(4‐chlorophenyl)amine **3d** proved to be the most suitable electrocatalyst, affording product **4t** in 33% yield. Conversely, the electron‐deficient *p*‐CF_3_‐substituted azlactone required the more oxidizing electrocatalyst tris(4‐bromophenyl)amine **3f** [57] to form product **4u** in 28% yield. These results demonstrate the adaptability of the electrocatalytic platform to a broad range of amino acid‐derived azlactones bearing different C2 aromatic substituents, and highlight the importance of matching the redox properties of the catalyst to the electronics of the substrate.

**FIGURE 3:**
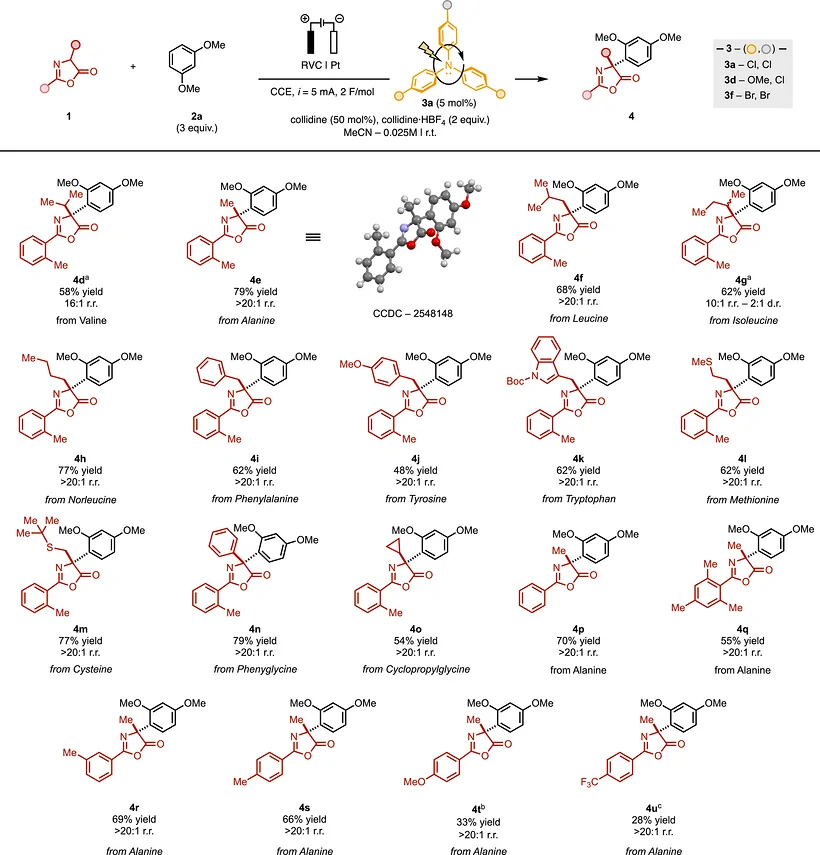
Electrocatalytic umpolung arylation of azlactones. ^a^1.0 equiv. of collidine in 4.0 mL of MeCN ^b^ **3d** used as electrocatalyst. ^c^ **3f** used as electrocatalyst.

Subsequently, we investigated the scope of the reaction using a variety of electron‐rich aromatic compounds (Figure 4). Efficient electrocatalysis requires the selective indirect oxidation of azlactone enolate **6** to radical **7**, while preventing electron transfer with radical trap **2** (see Figure 2Aii). To satisfy this key design principle, a redox potential difference (Δ*E*) of at least 500 mV between the electrocatalyst **3** and **2** was found to be required [57]. By leveraging the library of triarylamine electrocatalysts shown in Figure 2Bii, the system can be precisely tuned to optimize catalytic performance to specific substrates, thereby ensuring broad reactivity across structurally diverse electron‐rich SOMOphiles. For example, with the more electron‐rich 1,3,5‐trimethoxybenzene **2b**, whose oxidation takes place at *E*_p_ = +1.50 V [57] (compared to *E*_p_ = +1.67 V for **2a**), the best results were obtained with catalyst **3c** (*E*_1/2_ = +0.88 V, compared to *E*_1/2_ = +1.09 V for **3a**), yielding **4v** in 61% yield. Fused aromatics also participated in the electrocatalytic functionalization of azlactones. While 2‐methoxynaphthalene produced **4w** in 20% yield, the more activated 2,7‐dimethoxynaphthalene afforded **4x** in 67% yield, consistent with the greater electron density of the latter facilitating radical addition.

**FIGURE 4:**
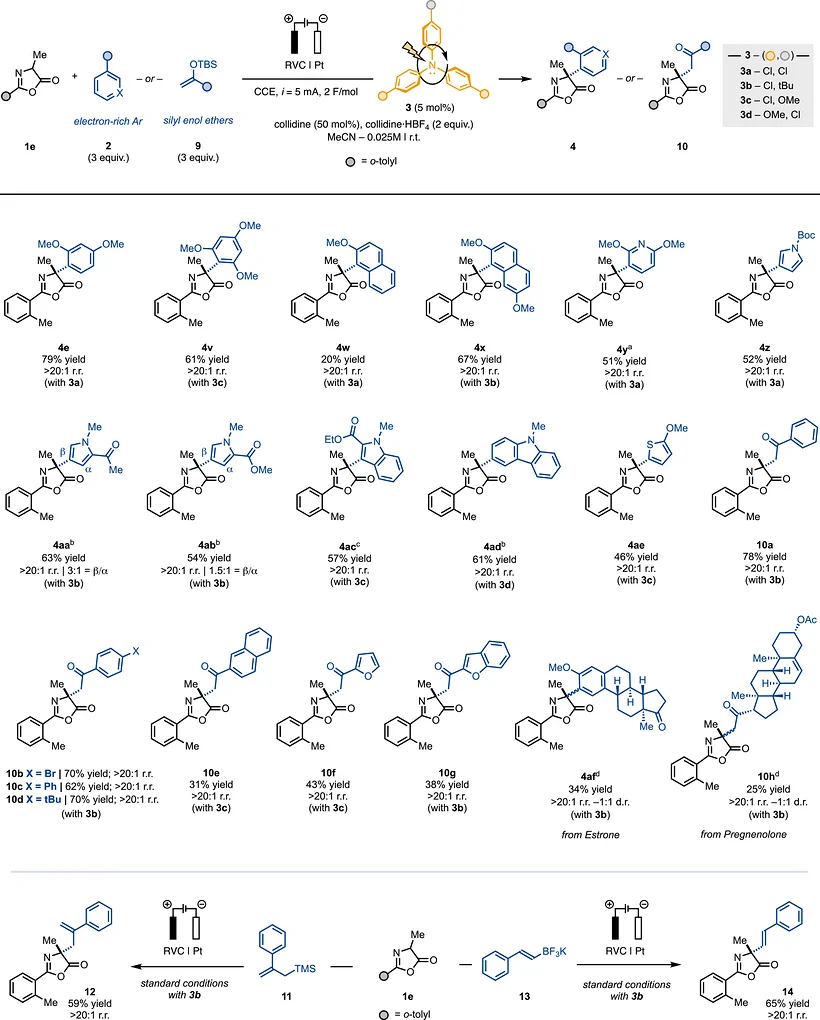
Electrocatalytic umpolung functionalization of azlactones with electron‐rich aromatics **2**, silyl enol ethers (SEEs) **9**, the allyl silane **11**, and the styryl trifluoroborate **13**. ^a^1.0 equiv. of collidine in 4.0 mL of MeCN ^b^10 equiv. of the electron‐rich SOMOphile. ^c^5 equiv. of the electron‐rich SOMOphile. ^d^MeCN/CH_2_Cl_2_ = 1:1 as solvent.

Nitrogenated aromatic heterocycles can also be incorporated into the amino acid surrogate. For example, 2,6‐dimethoxypyridine produced product **4y** with excellent regioselectivity and 51% yield. In the case of pyrroles, electron‐withdrawing substituents were instrumental in raising the oxidation potential sufficiently above that of the azlactone enolate, thereby enabling selective electrocatalytic activation of **1**. Consequently, *N*‐Boc pyrrole produced product **4z** in a 52% yield, whereas *N*‐methylpyrroles with carbonyl substituents at the 2‐position yielded products **4aa** and **4ab** in 63% and 54% yield, respectively. The methodology is also amenable to other classes of electron‐rich heteroaromatic SOMOphiles, providing access to the indole‐substituted azlactone **4ac** in 57% yield, the carbazole‐substituted azlactone **4ad** in 61% yield, and the thiophene‐substituted azlactone **4ae** in 46% yield (Figure 4).

Attempts to use more electron‐rich SOMOphiles exhibiting lower oxidation potentials (Δ*E* < 0.50 V) were unsuccessful. In these cases, oxidation of the SOMOphile becomes competitive with the productive indirect oxidation of the azlactone enolate **6**, resulting in SOMOphile decomposition. Conversely, less activated substrates, exhibiting higher oxidation potentials (*E*p > 1.90 V), also failed to furnish the desired products, most likely because they are insufficiently electron‐rich to achieve a favorable polarity match with the electrophilic azlactone radical **7**, leading to slow C─C bond formation [57].

Next, we explored silyl enol ethers (SEEs) **9** as SOMOphiles [61, 62, 63, 64] for Csp^3^─Csp^3^ bond formation at the α‐position of the amino acid surrogate (Figure 4). Under the optimized electrochemical conditions, and selecting the most suitable electrocatalyst, a range of methylbenzoyl motifs were successfully installed at the C4 of azlactone **1e**. The SEE from acetophenone, using catalyst **3b**, afforded the β‐oxoalkylated azlactone **10a** in 78% yield. A variety of aromatic substituents were well tolerated, delivering the corresponding quaternary products **10b**‐**10d** in 62%–70% yield with excellent regiocontrol, while SEEs bearing fused or heteroaromatic systems were also compatible, affording the naphthyl (**10e**), furyl (**10f**), and benzofuryl (**10g**) derivatives in 31%–43% yield. No products arising from the addition of the azlactone radical intermediate to the heteroaromatic moieties of the SEEs were detected.

We then explored whether the electrocatalytic arylation and β‐oxoalkylation protocols could be harnessed to embed complex, biologically relevant natural products into the amino acid core. We identified estrone, a human endogenous steroid bearing a phenolic motif, as a suitable candidate for cross‐dehydrogenative arylation. Following O‐methylation, the derivative was subjected to optimized conditions with electrocatalyst **3b**, affording the conjugated amino acid derivative **4af** in 34% yield. Likewise, Pregnenolone, bearing a terminal acetyl group, was selected as substrate for β‐oxoalkylation within a complex molecular setting. Accordingly, its SEE was exposed to standard electrochemical conditions with 5 mol% of **3b**, providing the coupled azlactone **10h** in 25% yield.

Finally, we evaluated structurally diverse electron‐rich SOMOphiles to further expand the repertoire of functionalities that can be installed at the α‐position of the amino acid scaffold (Figure 4, bottom) [65, 66, 67, 68]. On the one hand, exposure of **1e** to allyl silane **11** with catalyst **3b** afforded the α‐allylated quaternary azlactone **12** in 59% yield as a single regioisomer. On the other hand, the styryl trifluoroborate salt **13** also proved to be a competent coupling partner, delivering the α‐alkenylated quaternary amino acid surrogate **14** in 65% yield.

### 2.3 Protocol Transferability, Scalability, and Product Derivatization

To investigate the transferability of the protocol, we examined the electrochemical umpolung functionalization of **1e** with **2a** using alternative electrochemical setups to the IKA ElectraSyn 2.0 platform [69]. Using the Open‐ESyn platform developed by Lennox [70, 71], a lid compatible with the standard ElectraSyn electrodes that could be operated with a conventional galvanostat was 3D‐printed (Figure 5Aii, left). This set up afforded **4e** in 76% yield. Likewise, product **4e** was isolated in 73% yield using the electrochemical reactor developed by Lin (Figure 5Aii, right) [72]. Next, we evaluated the scalability of the electrochemical protocol. We designed and 3D‐printed a reactor lid [70, 71] which accommodates four standard ElectraSyn electrodes —two serving as anodes and two as cathodes—and fits with a conventional 29/32‐neck round‐bottom flask (see Figure 5Ai) [57]. As the effective electrode surface area is doubled, the electrolysis was conducted at 10 mA to maintain comparable current density to that employed under optimized conditions. Under these conditions and using 10 mol% of electrocatalyst **3a**, the arylation of **1e** could be successfully scaled up to 1 mmol, providing azlactone **4e** in 72% yield. We also examined the scalability of the electrochemical β‐oxoalkylation protocol using brominated SEE **9b**. Using the same double‐electrode setup (see figure 5Ai) and keeping the catalyst loading of **3b** at 5 mol%, product **10b** was isolated in a 70% yield at a 1 mmol scale, matching the efficiency observed at 0.2 mmol (see Figure 4).

**FIGURE 5:**
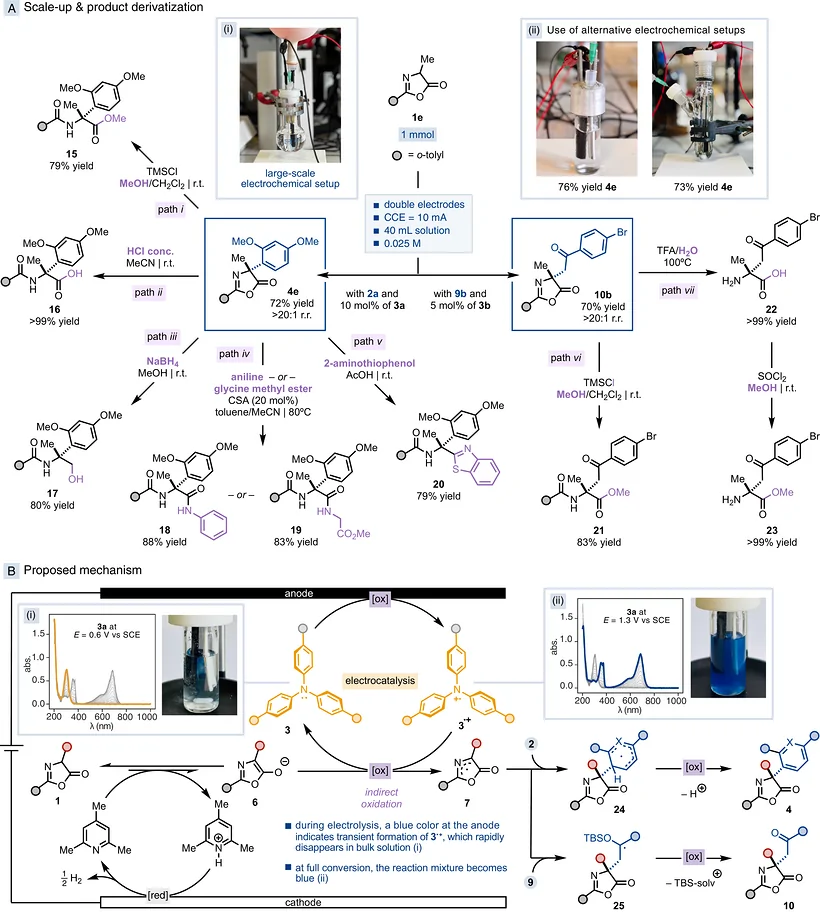
(A) Electrocatalytic formation of **4e** and **10b** at 1 mmol scale and product derivatizations. (i) Large‐scale electrochemical reactor. (ii) Alternative electrochemical setups. (B) Proposed mechanism. Spectroelectrochemical analyses of **3a** at (i) 0.60 V versus SCE, and (ii) 1.30 V versus SCE.

To further demonstrate the synthetic utility of the method, we investigated a series of derivatizations of the quaternary azlactones **4e** and **10b** (Figure 5A). Treating the functionalized azlactones with TMSCl in MeOH/CH_2_Cl_2_ enables smooth ring opening, providing the quaternary *N*‐benzoylated amino esters **15** and **21** in 79% and 83% yield, respectively (paths i and vi, Figure 5A). Additionally, the *N*‐benzoylated amino acid **16** could be obtained in quantitative yield by treating **4e** with HCl conc. in MeCN (path ii, Figure 5A). Exposure of **10b** to TFA in water at 100°C provided the quaternary amino acid **22**, which was subsequently esterified to the amino ester **23** (path vii, Figure 5A). Further diversification of **4e** was achieved through reduction to provide the *N*‐protected amino alcohol **17** in 80% yield (path iii, Figure 5A). The arylated azlactone **4e** proved to be a versatile coupling partner, undergoing direct amidation with aniline to afford **18** in 88% yield, and coupling with glycine methyl ester to form dipeptide **19** in an 83% yield (path iv, Figure 5A). Finally, treatment of **4e** with 2‐aminothiophenol under acidic conditions enabled azlactone‐ring opening and subsequent cyclization to generate the benzothiazole‐derived product **20** in 79% yield (path v, Figure 5A) [73, 74].

### 2.4 Mechanistic Studies

Based on the experimental results, together with the relevant precedents [40, 41, 42, 50, 51, 63, 64, 65, 66, 67, 68], the reaction mechanism shown in Figure 5B is proposed for the electrocatalytic umpolung functionalization of azlactones with electron‐rich SOMOphiles. First, collidine deprotonates azlactone **1** to form enolate **6**, while anodic oxidation of the triarylamine catalyst **3** generates its radical cation **3^·+^**. Comparison of the UV–vis spectra of **3a** at *E* = +0.60 V (Figure 5Bi) and *E* = +1.30 V (Figure 5Bi) reveals the emergence of a broad absorption band in the low‐energy visible region at the higher potential. Whereas no absorption bands are observed at λ > 380 nm at *E* = +0.60 V, in agreement with the fact that **3a** is a white solid, the new band centered at λ = 700 nm at *E* = +1.30 V is consistent with the blue color of the oxidized species **3^·+^**. Indeed, during electrolysis, the transient appearance of this blue color at the anode indicates catalyst activation (see picture in Figure 5Bi), which quickly disappears in the bulk solution, supporting a well‐matched electron‐transfer kinetics between **3^·+^** and **6** to generate the radical species **7**. Subsequently, this electrophilic open‐shell intermediate undergoes addition to electron‐rich SOMOPhiles such as **2** or **9**, forging the new C─C bond and generating either cyclohexadienyl radical **24** or the α‐alkoxy radical **25**. Further anodic oxidation of **24** or **25**, followed by deprotonation or solvent‐assisted desilylation, affords the functionalized quaternary azlactones **4** and **10**. Simultaneously, collidinium ion is reduced at the cathode to preserve electroneutrality within the electrochemical cell, regenerating collidine and producing hydrogen, as evidenced by the gas evolution at the cathode. Upon complete consumption of azlactone **1**, the reaction mixture becomes homogeneous and persistently blue (see picture in Figure 5Bii), characteristic of the oxidized electrocatalyst **3^·+^**. This observation is consistent with the inability of the electrocatalytic cycle to turnover in the absence of oxidizable species, while provides a convenient visual indicator of reaction completion.

## 3 Conclusion

In conclusion, we have developed an electrocatalytic strategy that enables the C4 functionalization of azlactones with electron‐rich SOMOphiles through polarity inversion, providing access to structurally diverse α‐quaternary amino acid derivatives. The successful reactivity and selectivity of the transformation are underpinned by several strategic features: (i) the identification of the collidine/collidinium·BF_4_ system as an effective non‐nucleophilic, non‐oxidizable base that also serves as a donor for cathodic proton reduction and supporting electrolyte, thereby preventing undesired nucleophilic azlactone ring‐opening; (ii) the modulation of the substituent at the C2 position of the azlactone, with the *o*‐tolyl group emerging as optimal, for achieving an effective balance between reactivity and regioselectivity across a broad substrate scope; and (iii) the use of a tailored library of *p*‐substituted triarylamines as electrocatalysts that enables precise tuning of the electrochemical system to match the electronic properties of different substrates, thereby ensuring efficient indirect oxidation of the azlactone enolates while suppressing detrimental electron‐transfer with the electron‐rich SOMOphiles. The methodology exhibits broad compatibility with both proteogenic and non‐proteogenic amino acid‐derived azlactones, as well as structurally diverse electron‐rich coupling partners, enabling the synthesis of arylated, β‐oxoalkylated, alkenylated, and allylated α‐quaternary amino acid derivatives. Moreover, the electrochemical conjugation of steroid‐based motifs into the amino acid framework highlights the late‐stage functionalization potential of the platform. The operational simplicity, scalability, and compatibility with diverse electrochemical setups further underscore the practicality and robustness of the methodology. Overall, this work establishes the electrocatalytic umpolung activation of azlactones as a sustainable and versatile strategy for the synthesis of α‐quaternary amino acids with electron‐rich coupling partners, while expanding the toolbox of synthetic electrochemical methods available for the construction of valuable organic scaffolds [75].

## Author Contributions

P.R. optimized the electrochemical methodology. P.R., with support from D.B., A.V.P., K.G., and S.C.D.K., investigated the generality of the method. D.B. and P.R. studied the alternative electrochemical setups and the scale‐up of the protocol. D.B. and A.V.P. performed product derivatization. B.L. conducted the spectroelectrochemical analyses. X.C. conceived and directed the project, acquired funding, and wrote the manuscript with input from P.R., D.B., A.V.P., and B.L. All authors contributed to and approved the final version of the manuscript.

## Conflicts of Interest

The authors declare no conflicts of interest.

## Supporting information




**Supporting File**: anie74374‐sup‐0001‐SuppMat.pdf.

## Data Availability

The data that supports the findings of this study are available in the Supporting Information of this article.

## References

[1] L. Wang and P. G. Schultz , “Expanding the Genetic Code,” Angewandte Chemie International Edition 44 (2005): 34–66, 10.1002/anie.200460627. 15599909

[2] M. A. T. Blaskovich , “Unusual Amino Acids in Medicinal Chemistry,” Journal of Medicinal Chemistry 59 (2016): 10807–10836, 10.1021/acs.jmedchem.6b00319. 27589349

[3] Y. Yu , C. Hu , L. Xia , and J. Wang , “Artificial Metalloenzyme Design with Unnatural Amino Acids and Non‐Native Cofactors,” ACS Catal 8 (2018): 1851–1863, 10.1021/acscatal.7b03754.

[4] K. K. Sharma , K. Sharma , K. Rao , et al., “Unnatural Amino Acids: Strategies, Designs, and Applications in Medicinal Chemistry and Drug Discovery,” Journal of Medicinal Chemistry 67 (2024): 19932–19965, 10.1021/acs.jmedchem.4c00110. PMC11901032 39527066

[5] J. Venkatraman , S. C. Shankaramma , and P. Balaram , “Design of Folded Peptides,” Chemical Reviews 101 (2001): 3131–3152, 10.1021/cr000053z. 11710065

[6] C. Toniolo , M. Crisma , F. Formaggio , and C. Peggion , “Control of Peptide Conformation by the Thorpe‐Ingold Effect (Cα‐tetrasubstitution),” Biopolymers 60 (2001): 396–419, 10.1002/1097-0282(2001)60:6<396::AID-BIP10184>3.0.CO;2-7. 12209474

[7] M. Crisma , A. Moretto , M. De Zotti , et al., “Turn Stabilization in Short Peptides by C α ‐Methylated α‐Amino Acids,” Biopolymers 80 (2005): 279–293, 10.1002/bip.20181. 15612047

[8] M. Tanaka , “Design and Synthesis of Chiral α,α‐Disubstituted Amino Acids and Conformational Study of Their Oligopeptides,” Chemical & Pharmaceutical Bulletin 55 (2007): 349–358, 10.1248/cpb.55.349. 17329870

[9] C. Nájera and J. M. Sansano , “Catalytic Asymmetric Synthesis of α‐Amino Acids,” Chemical Reviews 107 (2007): 4584–4671, 10.1021/cr050580o. 17915933

[10] B. Eftekhari‐Sis and M. Zirak , “α‐Imino Esters in Organic Synthesis: Recent Advances,” Chemical Reviews 117 (2017): 8326–8419, 10.1021/acs.chemrev.7b00064. 28603976

[11] P. Pecchini , M. Fochi , F. Bartoccini , G. Piersanti , and L. Bernardi , “Enantioselective Organocatalytic Strategies to Access Noncanonical α‐amino Acids,” Chemical Science 15 (2024): 5832–5868, 10.1039/D4SC01081G. PMC11041364 38665517

[12] Y. Zhang , J. Vanderghinste , J. Wang , and S. Das , “Challenges and Recent Advancements in the Synthesis of α,α‐disubstituted α‐Amino Acids,” Nature Communications 15 (2024): 1474. 10.1038/s41467-024-45790-2 PMC10874380 38368416

[13] A. Scala , A. Piperno , N. Micale , F. Christ , and Z. Debyser , “Synthesis and Anti‐HIV Profile of a Novel Tetrahydroindazolylbenzamide Derivative Obtained by Oxazolone Chemistry,” Acs Medicinal Chemistry Letters 10 (2019): 398–401, 10.1021/acsmedchemlett.8b00511. PMC6466547 30996769

[14] L. Jia , S. M. Kilbey , and X. Wang , “Tailoring Azlactone‐Based Block Copolymers for Stimuli‐Responsive Disassembly of Nanocarriers,” Langmuir 36 (2020): 10200–10209, 10.1021/acs.langmuir.0c01681. 32787052

[15] F. François , G. Gody , J. Wilson , L. Fontaine , and S. Pascual , “Azlactone‐Based Copolymers by Redox‐initiated MADIX Polymerization at Room Temperature in Ethanol,” Polymer Chemistry 15 (2024): 1453–1459, 10.1039/d4py00070f.

[16] A. R. Alba and R. Rios , “Oxazolones in Organocatalysis, New Tricks for an Old Reagent,” Chemistry—An Asian Journal 6 (2011): 720–734, 10.1002/asia.201000636. 21344651

[17] P. P. de Castro , A. G. Carpanez , and G. W. Amarante , “Azlactone Reaction Developments,” Chemistry—A European Journal 22 (2016): 10294–10318, 10.1002/chem.201600071. 27245128

[18] M. H. R. Carvalho , G. F. Vargas , J. P. R. S. Ribeiro , P. P. de Castro , and G. W. Amarante , “Azlactone Rings: Uniting Tradition and Innovation in Synthesis,” Org Chem Front 12 (2025): 4352–4398, 10.1039/D5QO00657K.

[19] D. Uraguchi , Y. Ueki , and T. Ooi , “Chiral Organic Ion Pair Catalysts Assembled Through a Hydrogen‐Bonding Network,” Science 326 (2009): 120–123, 10.1126/science.1176758. 19713492

[20] A. Balaguer , X. Companyó , T. Calvet , M. Font‐Bardía , A. Moyano , and R. Rios , “Highly Regio‐ and Diastereoselective Oxazol‐5‐One Addition to Nitrostyrenes,” European Journal of Organic Chemistry 2009 (2009): 199–203, 10.1002/ejoc.200801005.

[21] A. R. Alba , G. Valero , T. Calbet , M. Font‐Bardía , A. Moyano , and R. Rios , “Enantioselective Organocatalytic Addition of Azlactones to Maleimides: A Highly Stereocontrolled Entry to 2,2‐Disubstituted‐2 *H* ‐oxazol‐5‐ones,” Chemistry—A European Journal 16 (2010): 9884–9889, 10.1002/chem.201000239. 20486106

[22] H. Jiang , M. W. Paixão , D. Monge , and K. A. Jørgensen , “Acyl Phosphonates: Good Hydrogen Bond Acceptors and Ester/Amide Equivalents in Asymmetric Organocatalysis,” Journal of the American Chemical Society 132 (2010): 2775–2783, 10.1021/ja9097803. 20131827

[23] X. Han , F. Chen , C. Ye , and Y. Wang , “Enantioselective Conjugate Addition of Azlactones to Nitroolefins with a Thiourea‐Based Bifunctional Organocatalyst,” Synlett 28 (2017): 989–993, 10.1055/s-0036-1588942.

[24] X.‐D. Bai , Q.‐F. Zhang , and Y. He , “Enantioselective Iridium Catalyzed α‐alkylation of Azlactones by a Tandem Asymmetric Allylic Alkylation/Aza‐Cope Rearrangement,” Chemical Communications 55 (2019): 5547–5550, 10.1039/C9CC01450K. 30993284

[25] K. Fujita , M. Miura , Y. Funahashi , T. Hatanaka , and S. Nakamura , “Enantioselective Reaction of 2 *H* ‐Azirines with Oxazol‐5‐(4 *H*)‐ones Catalyzed by Cinchona Alkaloid Sulfonamide Catalysts,” Organic Letters 23 (2021): 2104–2108, 10.1021/acs.orglett.1c00259. 33650878

[26] L. Lin , M. Wang , J. Zhou , F. Li , and H. Liu , “Highly Diastereo‐ and Enantioselective C2 Addition of 5 *H* ‐oxazol‐4‐ones to γ‐keto‐α,β‐unsaturated Esters,” Chemical Communications 59 (2023): 3606–3609, 10.1039/d3cc00554b. 36891781

[27] S. Cabrera , E. Reyes , J. Alemán , A. Milelli , S. Kobbelgaard , and K. A. Jørgensen , “Organocatalytic Asymmetric Synthesis of α,α‐Disubstituted α‐Amino Acids and Derivatives,” Journal of the American Chemical Society 130 (2008): 12031–12037, 10.1021/ja804567h. 18698775

[28] Y. Hayashi , K. Obi , Y. Ohta , D. Okamura , and H. Ishikawa , “Diphenylprolinol Silyl Ether as a Catalyst in an Enantioselective, Catalytic Michael Reaction for the Formation of α,α‐Disubstituted α‐Amino Acid Derivatives,” Chemistry—An Asian Journal 4 (2009): 246–249, 10.1002/asia.200800394. 19065594

[29] A. R. Alba , X. Companyó , G. Valero , A. Moyano , and R. Rios , “Enantioselective Organocatalytic Addition of Oxazolones to 1,1‐Bis(phenylsulfonyl)Ethylene: A Convenient Asymmetric Synthesis of Quaternary α‐Amino Acids,” Chemistry—A European Journal 16 (2010): 5354–5361, 10.1002/chem.200903025. 20209524

[30] N. Bravo , A.‐N. R. Alba , G. Valero , X. Companyó , A. Moyano , and R. Rios , “Asymmetric Organocatalytic Michael Addition of Azlactones to Cis‐1,2‐bis(phenylsulfonyl)Ethene. A Simple Entry to Quaternary α‐Amino Acids,” New Journal of Chemistry 34 (2010): 1816–1820, 10.1039/C0NJ00321B.

[31] G. Li , W. Sun , J. Li , F. Jia , L. Hong , and R. Wang , “Organocatalytic Enantioselective Formal Arylation of Azlactones Using Quinones as the Aromatic Partner,” Chemical Communications 51 (2015): 11280–11282, 10.1039/C5CC03677A. 26083993

[32] X. Wei , D. Liu , Q. An , and W. Zhang , “Hydrogen‐Bond Directed Regioselective Pd‐Catalyzed Asymmetric Allylic Alkylation: The Construction of Chiral α‐Amino Acids with Vicinal Tertiary and Quaternary Stereocenters,” Organic Letters 17 (2015): 5768–5771, 10.1021/acs.orglett.5b02868. 26575242

[33] T. Wang , Z. Yu , D. L. Hoon , C. Y. Phee , Y. Lan , and Y. Lu , “Regiodivergent Enantioselective γ‐Additions of Oxazolones to 2,3‐Butadienoates Catalyzed by Phosphines: Synthesis of α,α‐Disubstituted α‐Amino Acids and *N*,*O* ‐Acetal Derivatives,” Journal of the American Chemical Society 138 (2016): 265–271, 10.1021/jacs.5b10524. 26629975

[34] W. Li , X. Xu , Y. Liu , H. Gao , Y. Cheng , and P. Li , “Enantioselective Organocatalytic 1,6‐Addition of Azlactones to *Para* ‐Quinone Methides: An Access to α,α‐Disubstituted and β,β‐Diaryl‐α‐Amino Acid Esters,” Organic Letters 20 (2018): 1142–1145, 10.1021/acs.orglett.8b00072. 29363977

[35] H.‐C. Lin , P.‐P. Xie , Z.‐Y. Dai , et al., “Nucleophile‐Dependent *Z* /*E*‐And Regioselectivity in the Palladium‐Catalyzed Asymmetric Allylic C–H Alkylation of 1,4‐Dienes,” Journal of the American Chemical Society 141 (2019): 5824–5834, 10.1021/jacs.8b13582. 30862155

[36] V. Haider , P. Zebrowski , J. Michalke , U. Monkowius , and M. Waser , “Enantioselective Organocatalytic Syntheses of α‐selenated α‐ and β‐amino Acid Derivatives,” Organic & Biomolecular Chemistry 20 (2022): 824–830, 10.1039/D1OB02235K. PMC8790592 35015015

[37] C. Gui , Y. Peng , Y. Zhou , et al., “Cu‐Catalyzed Intermolecular Asymmetric Propargylic Substitution of *N* ‐Hydroxyphthalimide Esters with Propargyl Carbonates,” ACS Catalysis 13 (2023): 13735–13742, 10.1021/acscatal.3c03814.

[38] T. Tsuji , T. Tanaka , T. Tanaka , R. Yazaki , and T. Ohshima , “Catalytic Aerobic Cross‐Dehydrogenative Coupling of Azlactones en Route to α,α‐Disubstituted α‐Amino Acids,” Organic Letters 22 (2020): 4164–4170, 10.1021/acs.orglett.0c01248. 32396012

[39] T. Tanaka , T. Tanaka , T. Tsuji , R. Yazaki , and T. Ohshima , “Strategy for Catalytic Chemoselective Cross‐Enolate Coupling Reaction via a Transient Homocoupling Dimer,” Organic Letters 20 (2018): 3541–3544, 10.1021/acs.orglett.8b01313. 29851494

[40] Y. Li , J. Yang , X. Geng , et al., “Modular Construction of Unnatural α‐Tertiary Amino Acid Derivatives by Multicomponent Radical Cross‐Couplings,” Angewandte Chemie International Edition 61 (2022): e202210755, 10.1002/anie.202210755. 36205517

[41] K. Zhu , Y. Ma , Z. Wu , J. Wu , and Y. Lu , “Energy‐Transfer‐Enabled Regioconvergent Alkylation of Azlactones via Photocatalytic Radical–Radical Coupling,” ACS Catalysis 13 (2023): 4894–4902, 10.1021/acscatal.2c05698.

[42] C. Gao , Y.‐Z. Liu , Y.‐N. Wang , Z.‐Q. Zhang , and Z.‐P. Zhan , “Regioselective Synthesis of α‐Quaternary Amino Acid Derivatives Enabled by Photoinduced Energy Transfer,” Journal of Organic Chemistry 89 (2024): 10393–10402, 10.1021/acs.joc.4c01153. 38953569

[43] M. Yan , Y. Kawamata , and P. S. Baran , “Synthetic Organic Electrochemical Methods Since 2000: On the Verge of a Renaissance,” Chemical Reviews 117 (2017): 13230–13319, 10.1021/acs.chemrev.7b00397. PMC5786875 28991454

[44] T. H. Meyer , I. Choi , C. Tian , and L. Ackermann , “Powering the Future: How Can Electrochemistry Make a Difference in Organic Synthesis?,” Chem 6 (2020): 2484–2496, 10.1016/j.chempr.2020.08.025.

[45] D. Pollok and S. R. Waldvogel , “Electro‐organic Synthesis‐A 21st Century Technique,” Chemical Science 11 (2020): 12386–12400, 10.1039/d0sc01848a. PMC8162804 34123227

[46] N. E. S. Tay , D. Lehnherr , and T. Rovis , “Photons or Electrons? A Critical Comparison of Electrochemistry and Photoredox Catalysis for Organic Synthesis,” Chemical Reviews 122 (2022): 2487–2649, 10.1021/acs.chemrev.1c00384. PMC10021920 34751568

[47] R. Francke and R. D. Little , “Redox Catalysis in Organic Electrosynthesis: Basic Principles and Recent Developments,” Chemical Society Reviews 43 (2014): 2492–2521, 10.1039/c3cs60464k. 24500279

[48] R. Francke , A. Prudlik , and D. R , Little in Electrochemistry in Organic Synthesis (Georg Thieme Verlag KG, 2022), 293–324.

[49] W. R. Bowman and J. M. D. Storey , “Synthesis Using Aromatic Homolytic Substitution—Recent Advances,” Chemical Society Reviews 36 (2007): 1803–1822, 10.1039/b605183a. 18213987

[50] J. C. Conrad , J. Kong , B. N. Laforteza , and D. W. C. MacMillan , “Enantioselective α‐Arylation of Aldehydes via Organo‐SOMO Catalysis. An Ortho‐Selective Arylation Reaction Based on an Open‐Shell Pathway,” Journal of the American Chemical Society 131 (2009): 11640–11641, 10.1021/ja9026902. PMC2758563 19639997

[51] K. C. Nicolaou , R. Reingruber , D. Sarlah , and S. Bräse , “Enantioselective Intramolecular Friedel–Crafts‐Type α‐Arylation of Aldehydes,” Journal of the American Chemical Society 131 (2009): 2086–2087, 10.1021/ja809405c. 19173649

[52] Y. Yuan , J. Yang , and A. Lei , “Recent Advances in Electrochemical Oxidative Cross‐Coupling with Hydrogen Evolution Involving Radicals,” Chemical Society Reviews 50 (2021): 10058–10086, 10.1039/D1CS00150G. 34369504

[53] M. Goodman and L. Levine , “Peptide Synthesis via Active Esters. IV. Racemization and Ring‐Opening Reactions of Opitcally Active Oxazolones,” Journal of the American Chemical Society 86 (1964): 2918–2922, 10.1021/ja01068a030.

[54] J. de Jersey and B. Zerner , “On the Spontaneous and Enzyme‐Catalyzed Hydrolysis of Saturated Oxazolinones,” Biochemistry 8 (1969): 1967–1974, 10.1021/bi00833a029. 5814935

[55] D. D. Perrin , Dissociation Constants of Organic Bases in Aqueous Solution (Butterworths, London, 1965).

[56] S. Liu , L. Ma , F. Hao , et al., “Molecular Electro(Photo)Catalysis: Triarylamine and Triarylimidazole Derivatives Mediated Oxidation Systems in Organic Electrosynthesis,” Advanced Synthesis & Catalysis 366 (2024): 3943–3962, 10.1002/adsc.202400435.

[57] See Supporting Information for further details.

[58] C. Kiaku , D. Martinage , Y. Sicim , et al., “eFluorination of Activated Alcohols Using Collidinium Tetrafluoroborate,” Organic Letters 26 (2024): 2697–2701, 10.1021/acs.orglett.3c00976. 37204455

[59] J. L. Fauchère , M. Charton , L. B. Kier , A. Verloop , and V. Pliska , “Amino Acid Side Chain Parameters for Correlation Studies in Biology and Pharmacology,” International Journal of Peptide and Protein Research 32 (1988): 269–278, 10.1111/j.1399-3011.1988.tb01261.x. 3209351

[60] Deposition Number 2548148 (for 4e) Contains the Supplementary Crystallographic Data for this Paper. These data are provided free of charge by the joint Cambridge Crystallographic Data Centre and Fachinformationszentrum Karlsruhe Access Structures service.

[61] P. V. Pham , D. A. Nagib , and D. W. C. MacMillan , “Photoredox Catalysis: A Mild, Operationally Simple Approach to the Synthesis of α‐Trifluoromethyl Carbonyl Compounds,” Angewandte Chemie International Edition 50 (2011): 6119–6122, 10.1002/anie.201101861. PMC3310164 21604347

[62] D. Spinnato , B. Schweitzer‐Chaput , G. Goti , M. Ošeka , and P. Melchiorre , “A Photochemical Organocatalytic Strategy for the α‐Alkylation of Ketones by Using Radicals,” Angewandte Chemie International Edition 59 (2020): 9485–9490, 10.1002/anie.201915814. PMC7317845 32053279

[63] D. Mazzarella , C. Qi , M. Vanzella , A. Sartorel , G. Pelosi , and L. Dell'Amico , “Electrochemical Asymmetric Radical Functionalization of Aldehydes Enabled by a Redox Shuttle,” Angewandte Chemie International Edition 63 (2024): e202401361, 10.1002/anie.202401361. 38623693

[64] N. Li , X. Ye , Y. Liu , and J. Song , “Enantioselective Radical α‐enolation of Esters via Electrochemical Chiral Isothiourea Catalysis,” Nat Catal 8 (2025): 957–967, 10.1038/s41929-025-01408-4.

[65] Q. Zhang , K. Liang , and C. Guo , “Enantioselective Nickel‐Catalyzed Electrochemical Radical Allylation,” Angewandte Chemie International Edition 61 (2022): e202210632, 10.1002/anie.202210632. 35912822

[66] Q. Zhang , J. Zhang , W. Zhu , R. Lu , and C. Guo , “Enantioselective Nickel‐Catalyzed Anodic Oxidative Dienylation and Allylation Reactions,” Nature Communications 15 (2024): 4477, 10.1038/s41467-024-48936-4. PMC11127924 38796470

[67] Y. Duan , Y. Zhang , J. Chen , F. Zeng , L. Cheng , and S. Luo , “Asymmetric Electrochemical Alkenylation by Synergistic Chiral Primary Amine and Naphthalene Catalysis,” Angewandte Chemie International Edition 64 (2025): e202505826, 10.1002/anie.202505826. 40243283

[68] X. Ye , B. Wei , Y. Li , et al., “Lewis Base‐Electrocatalyzed Enantioselective Radical Cross‐Coupling of Esters,” Journal of the American Chemical Society 148 (2026): 12264–12275, 10.1021/jacs.6c00782. 41805355

[69] M. Yan , Y. Kawamata , and P. S. Baran , “Synthetic Organic Electrochemistry: Calling all Engineers,” Angewandte Chemie International Edition 57 (2018): 4149–4155, 10.1002/anie.201707584. PMC5823775 28834012

[70] A. Choi , D. M. Heard , C. S. Haydon , and A. J. J. Lennox , “Open‐ESyn: A 3D‐Printed Toolkit for Expanded ElectraSyn Functionality and Reproducibility for Electrosynthesis,” ChemElectroChem 11 (2024): e202400454, 10.1002/celc.202400454.

[71] D. M. Heard , S. W. Deeks , and A. J. J. Lennox , “ERCAD: A Parametric Reactor Design Tool That Enables Rapid Prototyping and Optimization of Electrochemical Reactors through 3D Printing,” ACS Cent Sci 10 (2024): 2028–2035, 10.1021/acscentsci.4c00988. PMC11613433 39634220

[72] N. Fu , G. S. Sauer , and S. Lin , “A General, Electrocatalytic Approach to the Synthesis of Vicinal Diamines,” Nature Protocols 13 (2018): 1725–1743, 10.1038/s41596-018-0010-0. 30072721

[73] A. Rouf and C. Tanyeli , “Bioactive Thiazole and Benzothiazole Derivatives,” European Journal of Medicinal Chemistry 97 (2015): 911–927, 10.1016/j.ejmech.2014.10.058. 25455640

[74] Aayishamma 1 , G. S. Purawarga Matada , R. Pal , et al., “Benzothiazole a Privileged Scaffold for Cutting‐Edges Anticancer Agents: Exploring Drug Design, Structure‐activity Relationship, and Docking Studies,” European Journal of Medicinal Chemistry 279 (2024): 116831, 10.1016/j.ejmech.2024.116831. 39255643

[75] A preliminary version of this manuscript has been deposited in ChemRxiv repository: P. Rodríguez , D. Buedo , A. Vega‐Peñaloza , et al., “Electrocatalytic Polarity Inversion of Azlactones: Access to α‐Quaternary Amino Acids With Electron‐Rich Coupling Partners,”, ChemRxiv June 2, 2026, 10.26434/chemrxiv.15004182/v1.

