# Supplementary material for: Electrocatalytic Polarity Inversion of Azlactones: Access to α‐Quaternary Amino Acids with Electron‐Rich SOMOphiles
Source: Angew Chem Int Ed Engl. 2026 Aug 26;65(42):e8095368. doi: 10.1002/anie.8095368 (PMC13653626; doi:10.1002/anie.8095368)
Supplement: Supplementary file 1 [file ANIE-65-e8095368-s001.pdf]

Supporting Information

Electrocatalytic Polarity Inversion of Azlactones: Access to α-Quaternary Amino Acids with Electron-Rich SOMOphiles

Paula Rodríguez, David Buedo, Alberto Vega-Peñaloza, Kian Gosling, Stuart C. D. Kennington, Bart Limburg and Xavier Companyó*

Section of Organic Chemistry – Department of Inorganic and Organic Chemistry
University of Barcelona
carrer Martí i Franquès 1, 08028 Barcelona, Spain

E-mail: x.companyo@ub.edu

Table of Contents

A. General Information **4**

B. Synthesis of Starting Materials **5**

B.1. General Procedure A – *Synthesis of azlactones* *5*

B.2. Synthesis of tyrosine derived azlactone 1j *10*

B.3. Synthesis of tryptophan derived azlactone 1k *12*

B.4. Synthesis of methylated estrone 2i *14*

B.5. General Procedure B – *Synthesis of TBS-protected silyl enol ethers* ***9*** *15*

B.6. Synthesis of the TBS-protected silyl enol ether from acetylated pregnenolone 9h *17*

B.7. Synthesis of trimethyl(2-phenylallyl)silane (11) *18*

B.8. Synthesis of Potassium trans-Styryl Trifluoroborate (13) *19*

B.9. General Procedure C – *Synthesis of triarylamine electrocatalysts* ***3a-3e*** *20*

B.10. Synthesis of tris(4-bromophenyl)amine 3f *22*

B.11. Synthesis of 4-bromo-N-(4-bromophenyl)-N-(4-(tert-butyl)phenyl)aniline 3g *23*

B.12. General Procedure D – *Synthesis of pyridine derived supporting electrolytes* *24*

C. Optimisation of the electrocatalytic reaction **26**

C.1. Optimisation – *Reaction design* *26*

C.2. Optimisation – *Solvent screening* *27*

C.3. Optimisation – *Electrocatalyst screening* *28*

C.4. Optimisation – *Electrocatalyst loading* *30*

C.5. Optimisation – *Base and supporting electrolyte* *31*

C.6. Optimisation – *Electrodes, current intensity, SOMOphile equivalents, concentration* *32*

D. Electrocatalytic Functionalisation of Azlactones – General Procedure E **33**

E. Electrochemical feasibility studies **49**

E.1. Cyclic voltammetry *– Assessment of the selective indirect oxidation of the enolate* *49*

E.2. Cyclic voltammetry – *Comparison of the oxidation potential of the enolate depending on the aromatic ring of the azlactone* *51*

E.3. Electron-rich SOMOphile generality – *Electrocatalyst tuning* *52*

E.4. Fate of the excess of SOMOphiles *55*

F. Unsuccessful substrates **57**

G. Large-scale electrochemical setup **59**

H. Alternative electrochemical setups **61**

H.1. Galvanostat reaction with Electrasyn vial and electrodes *61*

H.2. Galvanostat reaction with non-commercial cell and electrodes *62*

I. Derivatisations **63**

I.1. N-benzoylated amino ester formation *63*

I.2. N-benzoylated amino acid formation *64*
I.3. N-benzoylated amino alcohol formation *65*
I.5. Amide formation with aniline *66*
I.4. Dipeptide formation with glycine *67*
I.6. Benzothiazole formation with o-aminothiophenol *68*
I.7. Amino acid formation *69*
I.8. Amino ester formation *70*
J. Mechanistic considerations........71
J.1. Spectroelectrochemistry *71*
J.2. Radical trapping experiment *72*
K. References 73
L. Determination of the absolute configuration. X-Ray Crystallographic Data of compound 4e.76
M. NMR Spectra 86
M.1. NMR spectra of azlactones 1 .... *86*
M.2. NMR spectra of triarylamine electrocatalysts 3 .... *96*
M.3. NMR spectra of products 4 .... *100*
M.4. NMR spectra of products 10 .... *132*
M.5. NMR spectra of products 12 and 14 .... *141*
M.6. NMR spectra of derivatisation products *143*

A. General Information

NMR experiments: NMR spectra (^1H, ^{13}C and ^{19}F) were recorded on a Bruker Avance III HD 400 (400 MHz) spectrometer equipped with a CryoProbe™ Prodigy. The chemical shifts (δ) for ^1H and ^{13}C are given in ppm relative to residual signals of the solvents ($CHCl_3$, 7.26 ppm, ^1H NMR, 77.16 ppm ^{13}C NMR). Coupling constants are given in Hz. The following abbreviations are used to indicate the multiplicity: s, singlet; d, doublet; t, triplet; q, quartet; m, multiplet; bs, broad signal. NMR yields were calculated by using 1,3,5-trimethoxybenzene as internal standard.

High-Resolution Mass Spectra (HRMS) were obtained with a LTQ Orbitrap Velos (Thermo Fisher Scientific) SN 03135B instrument.

Chromatographic purification of products was accomplished by flash chromatography in Thermo Scientific Chemicals silica gel, 40-60 μ, 60 Å. Thin-layer chromatography (TLC) was carried out with Merck TLC plates with silica gel 60 F_{254}. Visualisation of the developed chromatography was performed by checking UV absorbance (254 nm) as well as with aqueous potassium permanganate and *p*-anisaldehyde solutions. Organic solutions were concentrated under reduced pressure on IKA rotary evaporators.

Cyclic voltammetry (CV) measurements were performed using a computer-controlled BASi Epsilon EClipse™ potentiostat (Metrohm, The Netherlands) with a C-3 voltammetry cell stand in an undivided cell fitted with a glassy carbon working electrode (3 mm diameter), a platinum counter electrode (1.6 mm) and a Ag/AgCl reference electrode. CVs were recorded at 100 mV/s scan rate in a 0.1 M solution of $TBAPF_6$, which was previously recrystallised. Following the suggestions by Nicewicz,[1] we converted all measured values from Ag/AgCl into SCE by subtracting 0.03 V.

Materials: Commercial grade reagents and solvents were purchased at the highest commercial quality from Sigma Aldrich, Apollo Scientific, TCI and Fluorochem and used as received, unless otherwise stated.

B. Synthesis of Starting Materials

B.1. General Procedure A – Synthesis of azlactones

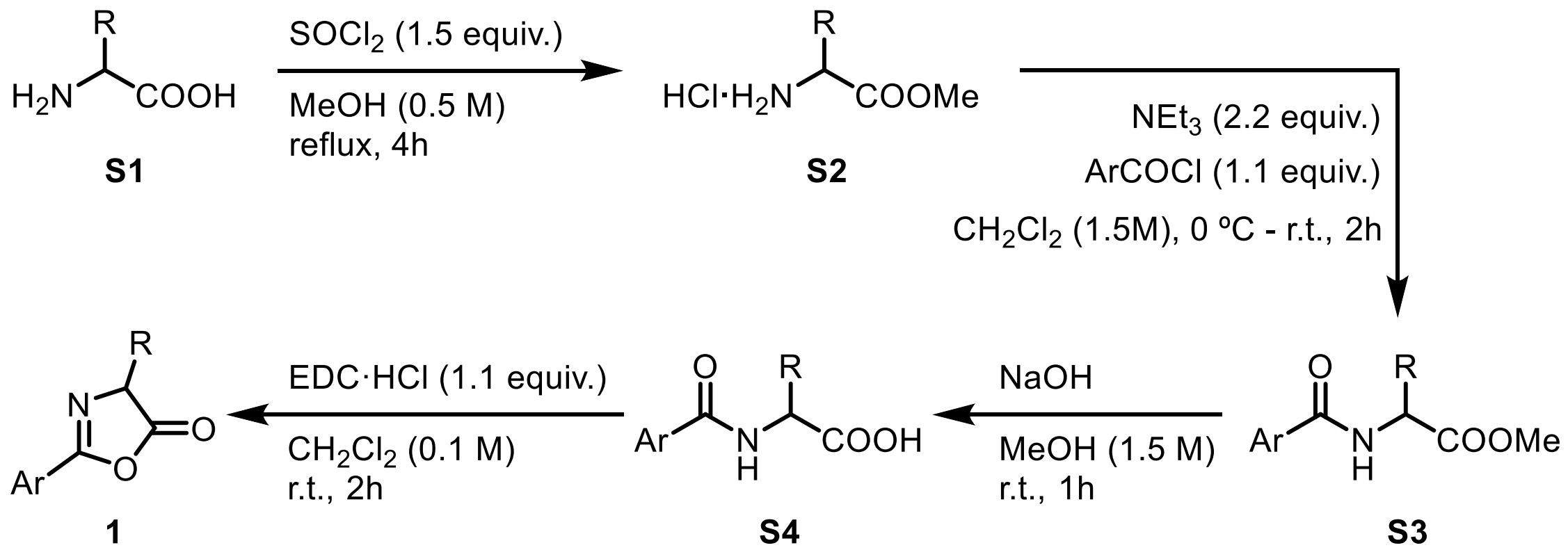

The reaction was carried out following the previously described procedures.[2–4]

The corresponding amino acid (**S1**) was suspended in MeOH (0.5 M) and cooled down to 0 ºC. Then, $SOCl_2$ (1.5 equiv.) was carefully added dropwise. The reaction mixture was heated at 65 ºC under reflux for4 h. The solvent was removed under reduced pressure to afford a white solid, which was subsequently washed with Et_2O. The corresponding ester hydrochlorides (**S2**) were directly used in the next step.

A mixture of the corresponding methyl ester hydrochloride (**S2**) and triethylamine (2.2 equiv.) in CH_2Cl_2 (1.5 M) was cooled down to 0 ºC. Under vigorous stirring, the corresponding acyl chloride was added dropwise. The mixture was subsequently stirred for a further 30 min at 0 °C and was then allowed to slowly warm to room temperature. After 2 h, the mixture was washed twice with HCl (1 M, aq.), twice with $NaHCO_3$ (sat., aq.) and twice with brine. The organic phase was dried over Na_2SO_4, filtered and concentrated under vacuum. The corresponding N-acyl methyl esters (**S3**) were directly used without further purification.

S3 were then dissolved in MeOH (1.5 M) and 2 M aqueous solution of NaOH (5 mL per 10 mmol) was added at room temperature. The resulting mixture was stirred for 1 h before evaporating MeOH under reduced pressure. The resulting aqueous mixture was washed twice with CH_2Cl_2 after ensuring a basic pH. Then, it was cooled to 0 °C before conc. aq. HCl was added to acidify the solution (until pH = 0-1). The resulting acidic aqueous phase was extracted three times with CH_2Cl_2. The organic phase was dried over Na_2SO_4, filtered and concentrated under vacuum. The corresponding products (**S4**) were dried under high vacuum and directly used in the next step.

The N-acyl derivatives (**S4**) were subsequently suspended in CH_2Cl_2 (0.1 M) and cooled to 0 °C. EDC·HCl (1.1 equiv.) was added portionwise over a 15 min period. After 30 min at 0 °C the cooling bath was removed, and the mixture was stirred for 2 h at room temperature. After this time the mixture was further diluted with CH_2Cl_2 (0.05 M) and washed with H_2O, twice with $NaHCO_3$ (sat., aq.) and twice with brine. Then, the organic phase was dried over Na_2SO_4, filtered and concentrated under vacuum. Pure azlactones (**1**) were obtained by silica gel chromatography.

4-isopropyl-2-phenyloxazol-5(4*H*)-one (1a)

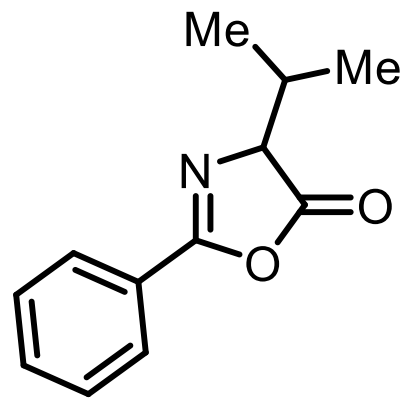

88% yield, white solid. Spectroscopic data match with those previously reported.[5]

^{1}H NMR (400 MHz, $CDCl_3$) δ 8.06 – 7.98 (m, 2H), 7.61 – 7.54 (m, 1H), 7.52 – 7.45 (m, 2H), 4.29 (d, *J* = 4.6 Hz, 1H), 2.38 (hd, *J* = 6.9, 4.6 Hz, 1H), 1.15 (d, *J* = 6.9 Hz, 3H), 1.02 (d, *J* = 6.9 Hz, 3H) ppm.

2-(tert-butyl)-4-isopropyloxazol-5(4*H*)-one (1b)

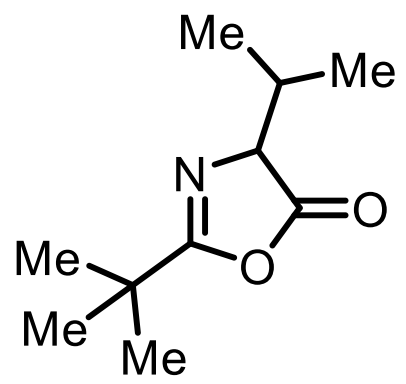

88% yield, colourless oil. Spectroscopic data match with those previously reported.[6]

^{1}H NMR (400 MHz, $CDCl_3$) δ 4.05 (d, *J* = 4.3 Hz, 1H), 2.28 (pd, *J* = 6.9, 4.3 Hz, 1H), 1.29 (s, 9H), 1.05 (d, *J* = 6.9 Hz, 3H), 0.95 (d, *J* = 6.9 Hz, 3H) ppm.

4-isopropyl-2-mesityloxazol-5(4*H*)-one (1c)

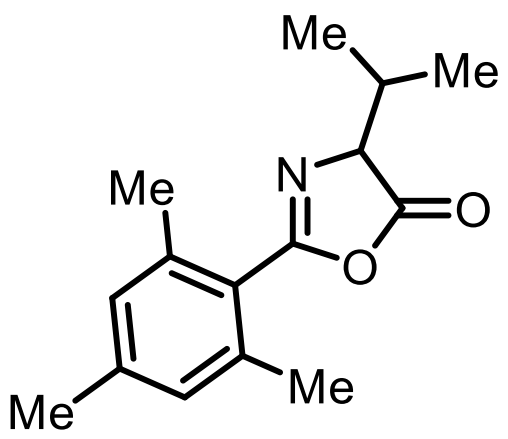

67% yield, colourless oil. Spectroscopic data match with those previously reported.[7]

^{1}H NMR (400 MHz, $CDCl_3$) δ 6.91 (h, *J* = 0.7 Hz, 2H), 4.31 (d, *J* = 4.5 Hz, 1H), 2.43 (pd, *J* = 6.9, 4.5 Hz, 1H), 2.34 (d, *J* = 0.7 Hz, 6H), 2.31 (d, *J* = 0.7 Hz, 3H), 1.20 (d, *J* = 6.9 Hz, 3H), 1.08 (d, *J* = 6.9 Hz, 3H) ppm.

4-isopropyl-2-(*o*-tolyl)oxazol-5(4*H*)-one (1d)

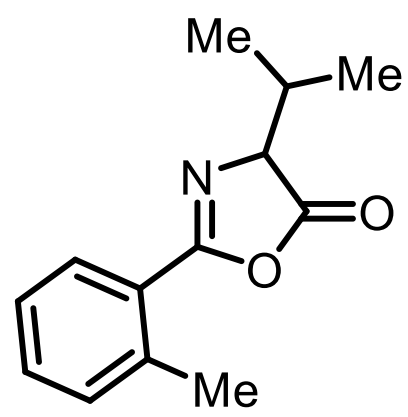

70% yield, colourless oil.

^{1}H NMR (400 MHz, $CDCl_3$) δ 7.89 – 7.82 (m, 1H), 7.46 – 7.38 (m, 1H), 7.34 – 7.27 (m, 2H), 4.31 (d, *J* = 4.5 Hz, 1H), 2.66 (s, 3H), 2.39 (pd, *J* = 6.9, 4.5 Hz, 1H), 1.17 (d, *J* = 6.9 Hz, 3H), 1.02 (d, *J* = 6.9 Hz, 3H) ppm. **^{13}C NMR** (101 MHz, $CDCl_3$) δ 178.1, 162.0, 139.8, 131.9, 130.0, 126.1, 125.0, 71.1, 31.3, 22.2, 19.1, 17.7 ppm.

4-methyl-2-(*o*-tolyl)oxazol-5(4*H*)-one (1e)

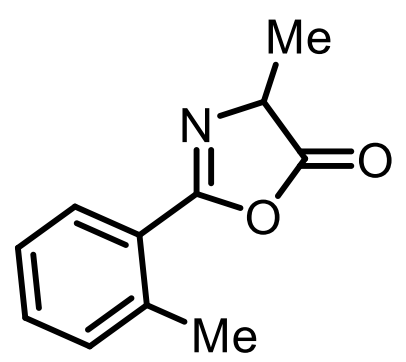

60% yield, colourless oil. Spectroscopic data match with those previously reported.[8]

^{1}H NMR (400 MHz, $CDCl_3$) δ 7.86 (dd, *J* = 8.3, 1.5 Hz, 1H), 7.42 (td, *J* = 7.7, 1.5 Hz, 1H), 7.34 – 7.27 (m, 2H), 4.47 (q, *J* = 7.6 Hz, 1H), 2.65 (s, 3H), 1.59 (d, *J* = 7.6 Hz, 3H) ppm.

4-isobutyl-2-(*o*-tolyl)oxazol-5(4*H*)-one (1f)

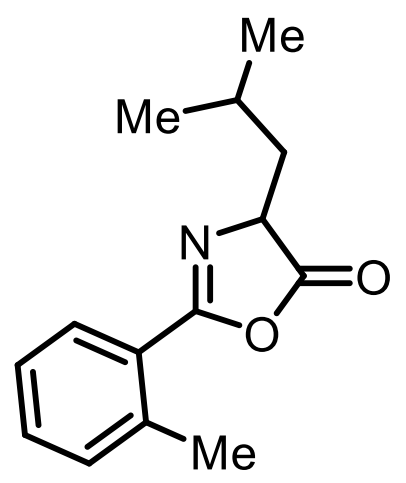

45% yield, colourless oil.

^{1}H NMR (400 MHz, $CDCl_3$) δ 7.89 – 7.82 (m, 1H), 7.44 – 7.37 (m, 1H), 7.32 – 7.26 (m, 2H), 4.43 (dd, *J* = 9.1, 5.6 Hz, 1H), 2.65 (s, 3H), 2.06 (dh, *J* = 7.5, 6.6 Hz, 1H), 1.87 (ddd, *J* = 13.3, 7.5, 5.6 Hz, 1H), 1.67 (ddd, *J* = 13.7, 9.1, 6.4 Hz, 1H), 1.06 – 1.00 (m, 6H) ppm. **^{13}C NMR** (101 MHz, $CDCl_3$) δ 179.2, 161.6, 139.8, 131.9, 131.8, 130.0, 126.1, 125.0, 64.3, 41.0, 25.5, 22.8, 22.3, 22.2 ppm.

4-(sec-butyl)-2-(*o*-tolyl)oxazol-5(4*H*)-one (1g)

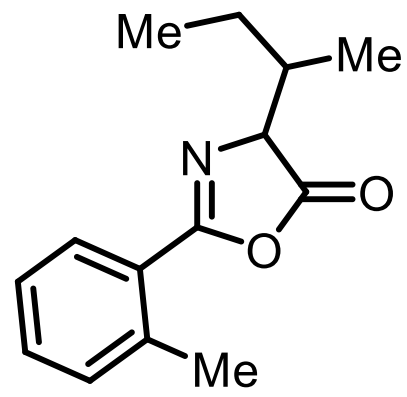

47% yield, yellowish oil.

^{1}H NMR (400 MHz, $CDCl_3$) δ (for mixture of diastereomers) 7.87 – 7.81 (m, 1H), 7.43 – 7.37 (m, 1H), 7.35 – 7.24 (m, 2H), 4.45 – 4.34 (m, 1H), 2.65 (s, 3H), 2.20 – 2.08 (m, 1H), 1.76 – 1.32 (m, 2H), 1.12 – 0.88 (m, 6H) ppm. **^{13}C NMR** (101 MHz, $CDCl_3$) δ (for mixture of diastereomers) 178.6, 177.8, 161.8, 161.8, 139.7, 139.7, 131.8, 131.7, 129.9, 126.0, 125.0, 70.0, 69.4, 37.7, 37.6, 26.5, 25.1, 22.1, 15.6, 14.5, 11.8, 11.7 ppm.

4-butyl-2-(*o*-tolyl)oxazol-5(4*H*)-one (1h)

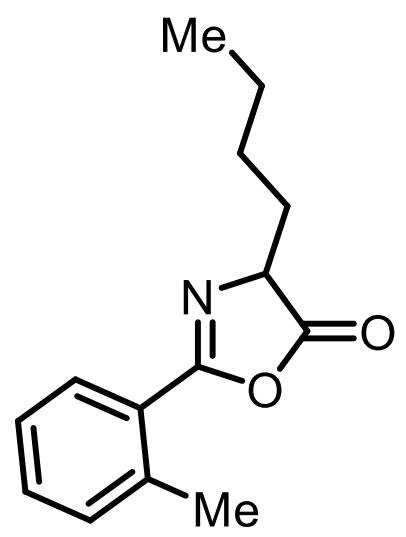

47% yield, white solid.

^{1}H NMR (400 MHz, $CDCl_3$) δ 7.88 – 7.83 (m, 1H), 7.45 – 7.39 (m, 1H), 7.33 – 7.26 (m, 2H), 4.43 (dd, *J* = 7.2, 5.5 Hz, 1H), 2.65 (s, 3H), 2.10 – 1.99 (m, 1H), 1.93 – 1.80 (m, 1H), 1.57 – 1.33 (m, 4H), 0.93 (t, *J* = 7.2 Hz, 3H) ppm. **^{13}C NMR** (101 MHz, $CDCl_3$) δ 178.9, 161.9, 139.8, 131.9, 130.1, 126.1, 125.0, 65.8, 31.5, 27.5, 22.4, 22.2, 13.9 ppm.

4-benzyl-2-(*o*-tolyl)oxazol-5(4*H*)-one (1i)

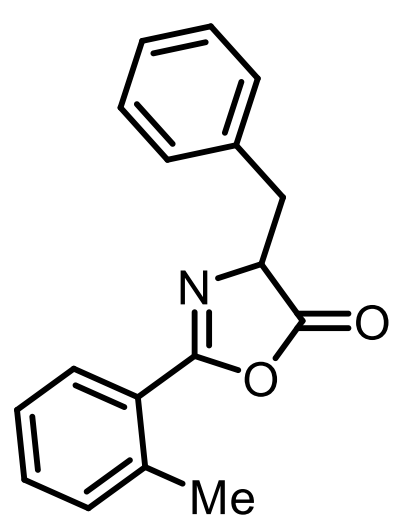

34% yield, white solid. Spectroscopic data match with those previously reported.[9]

^{1}H NMR (400 MHz, $CDCl_3$) δ 7.73 – 7.66 (m, 1H), 7.42 – 7.34 (m, 1H), 7.32 – 7.14 (m, 7H), 4.71 (dd, *J* = 6.2, 5.0 Hz, 1H), 3.38 (dd, *J* = 13.9, 4.9 Hz, 1H), 3.23 (dd, *J* = 13.9, 6.2 Hz, 1H), 2.50 (d, *J* = 2.1 Hz, 3H) ppm.

4-(2-(methylthio)ethyl)-2-(*o*-tolyl)oxazol-5(4*H*)-one (1l)

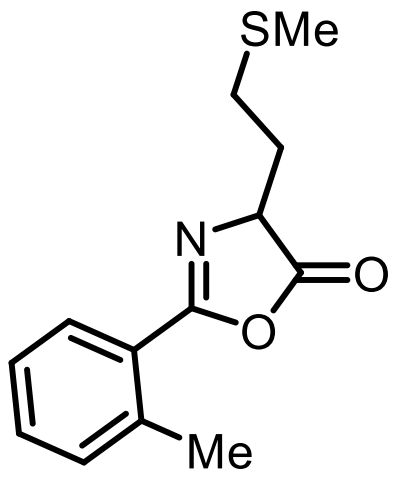

40% yield, colourless oil.

^{1}H NMR (400 MHz, $CDCl_3$) δ 7.90 – 7.83 (m, 1H), 7.45 – 7.39 (m, 1H), 7.33 – 7.26 (m, 2H), 4.61 (dd, *J* = 7.2, 5.8 Hz, 1H), 2.73 (t, *J* = 7.1 Hz, 2H), 2.64 (s, 3H), 2.33 (dtd, *J* = 14.0, 7.0, 5.8 Hz, 1H), 2.21 – 2.08 (m, 4H) ppm. **^{13}C NMR** (101 MHz, $CDCl_3$) δ 178.7, 162.4, 139.8, 132.0, 131.9, 130.1, 126.1, 124.9, 64.0, 30.6, 30.3, 22.3, 15.3 ppm.

4-((tert-butylthio)methyl)-2-(*o*-tolyl)oxazol-5(4*H*)-one (1m)

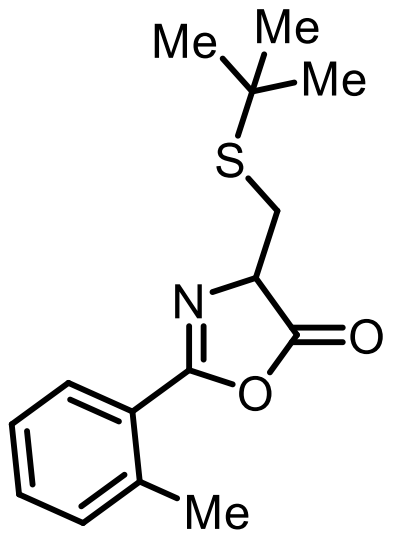

29% yield, white solid.

^{1}H NMR (400 MHz, $CDCl_3$) δ 7.89 – 7.85 (m, 1H), 7.45 – 7.39 (m, 1H), 7.31 – 7.26 (m, 2H), 4.71 (t, *J* = 5.0 Hz, 1H), 3.30 (dd, *J* = 12.8, 4.7 Hz, 1H), 3.02 (dd, *J* = 12.8, 5.2 Hz, 1H), 2.66 (s, 3H), 1.32 (s, 9H) ppm. **^{13}C NMR** (101 MHz, $CDCl_3$) δ 177.3, 162.9, 140.1, 132.1, 131.9, 130.2, 126.0, 124.8, 66.1, 42.8, 30.9, 29.2, 22.3 ppm.

4-methyl-2-(4-(trifluoromethyl)phenyl)oxazol-5(4*H*)-one (1n)

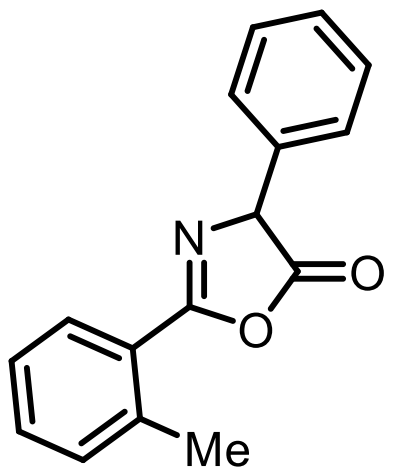

18% yield, yellow solid. Spectroscopic data match with those previously reported.[10]

^{1}H NMR (400 MHz, CD_3CN) δ 7.91 (dd, *J* = 7.9, 1.5 Hz, 1H), 7.52 (td, *J* = 7.5, 1.5 Hz, 1H), 7.49 – 7.38 (m, 7H), 5.64 (s, 1H), 2.69 (s, 3H) ppm.

4-cyclopropyl-2-(*o*-tolyl)oxazol-5(4*H*)-one (1o)

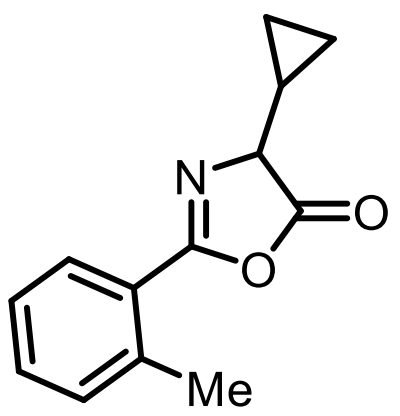

26% yield, colourless oil.

^{1}H NMR (400 MHz, $CDCl_3$) δ 7.89 – 7.82 (m, 1H), 7.45 – 7.39 (m, 1H), 7.33 – 7.26 (m, 2H), 4.32 (d, *J* = 5.8 Hz, 1H), 2.63 (s, 3H), 1.40 – 1.29 (m, 1H), 0.74 – 0.63 (m, 2H), 0.63 – 0.52 (m, 1H), 0.50 – 0.39 (m, 1H) ppm. **^{13}C NMR** (101 MHz, $CDCl_3$) δ 178.1, 162.3, 139.9, 132.0, 131.9, 130.1, 126.1, 124.9, 66.7, 22.3, 12.3, 2.0, 1.4 ppm.

4-methyl-2-phenyloxazol-5(4*H*)-one (1p)

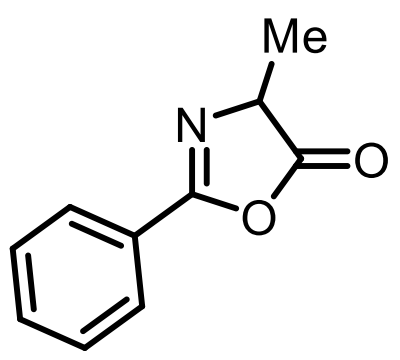

60% yield, white solid. Spectroscopic data match with those previously reported.[8]

^{1}H NMR (400 MHz, DMSO) δ 7.97 – 7.90 (m, 2H), 7.69 – 7.63 (m, 1H), 7.60 – 7.54 (m, 2H), 4.69 (q, *J* = 7.6 Hz, 1H), 1.44 (d, *J* = 7.6 Hz, 3H) ppm.

2-mesityl-4-methyloxazol-5(4*H*)-one (1q)

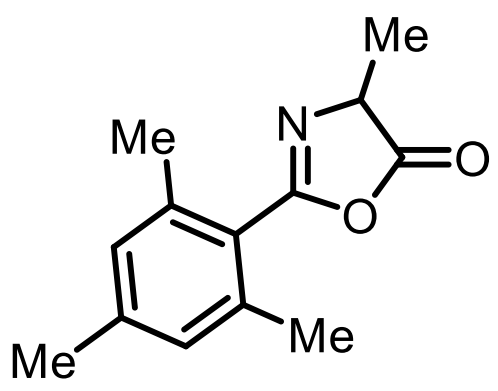

73% yield, white solid. Spectroscopic data match with those previously reported.[3]

^{1}H NMR (400 MHz, $CDCl_3$) δ 6.92 – 6.91 (m, 2H), 4.46 (q, *J* = 7.6 Hz, 1H), 2.33 (s, 6H), 2.31 (s, 3H), 1.62 (d, *J* = 7.6 Hz, 3H) ppm.

4-methyl-2-(*m*-tolyl)oxazol-5(4*H*)-one (1r)

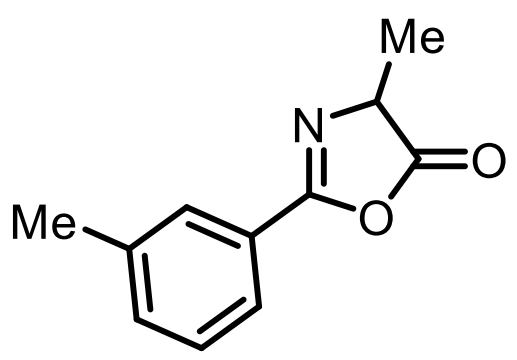

45% yield, white solid.

^{1}H NMR (400 MHz, $CDCl_3$) δ 7.84 – 7.81 (m, 1H), 7.80 – 7.74 (m, 1H), 7.42 – 7.32 (m, 2H), 4.44 (q, *J* = 7.6 Hz, 1H), 2.41 (s, 3H), 1.58 (d, *J* = 7.6 Hz, 3H) ppm. **^{13}C NMR** (101 MHz, $CDCl_3$) δ 179.2, 161.9, 138.8, 133.7, 128.8, 128.4, 125.9, 125.2, 61.1, 21.4, 17.1 ppm.

4-methyl-2-(*p*-tolyl)oxazol-5(4*H*)-one (1s)

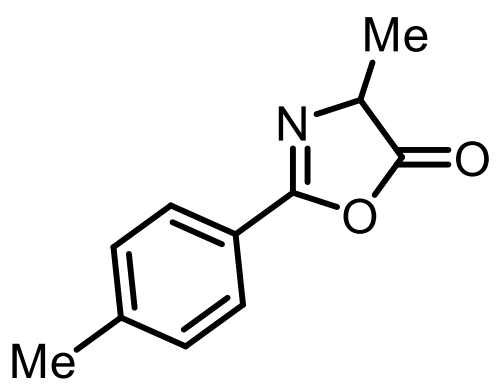

63% yield, white solid. Spectroscopic data match with those previously reported.[3]

^{1}H NMR (400 MHz, $CDCl_3$) δ 7.92 – 7.83 (m, 2H), 7.33 – 7.24 (m, 2H), 4.42 (q, *J* = 7.6 Hz, 1H), 2.42 (s, 3H), 1.57 (d, *J* = 7.6 Hz, 3H) ppm.

2-(4-methoxyphenyl)-4-methyloxazol-5(4*H*)-one (1t)

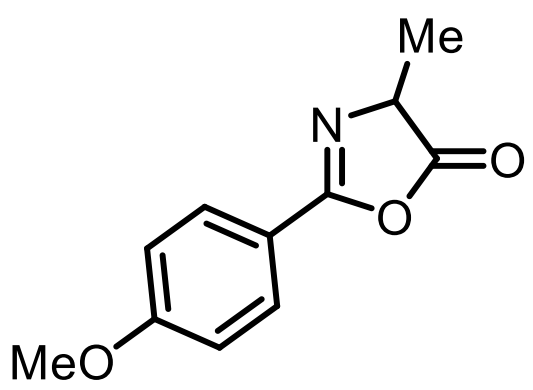

69% yield, white solid. Spectroscopic data match with those previously reported.[3]

^{1}H NMR (400 MHz, $CDCl_3$) δ 7.97 – 7.89 (m, 2H), 7.02 – 6.93 (m, 2H), 4.42 (q, *J* = 7.6 Hz, 1H), 3.88 (s, 3H), 1.57 (d, *J* = 7.6 Hz, 3H) ppm.

4-methyl-2-(4-(trifluoromethyl)phenyl)oxazol-5(4*H*)-one (1u)

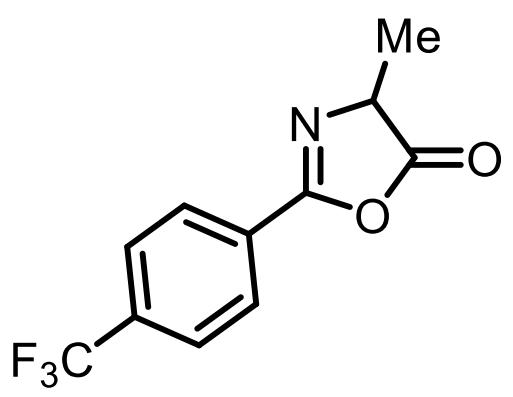

53% yield, white solid. Spectroscopic data match with those previously reported.[3]

^{1}H NMR (400 MHz, $CDCl_3$) δ 8.15 – 8.10 (m, 2H), 7.80 – 7.72 (m, 2H), 4.49 (q, *J* = 7.6 Hz, 1H), 1.61 (d, *J* = 7.6 Hz, 3H) ppm.

B.2. Synthesis of tyrosine derived azlactone 1j

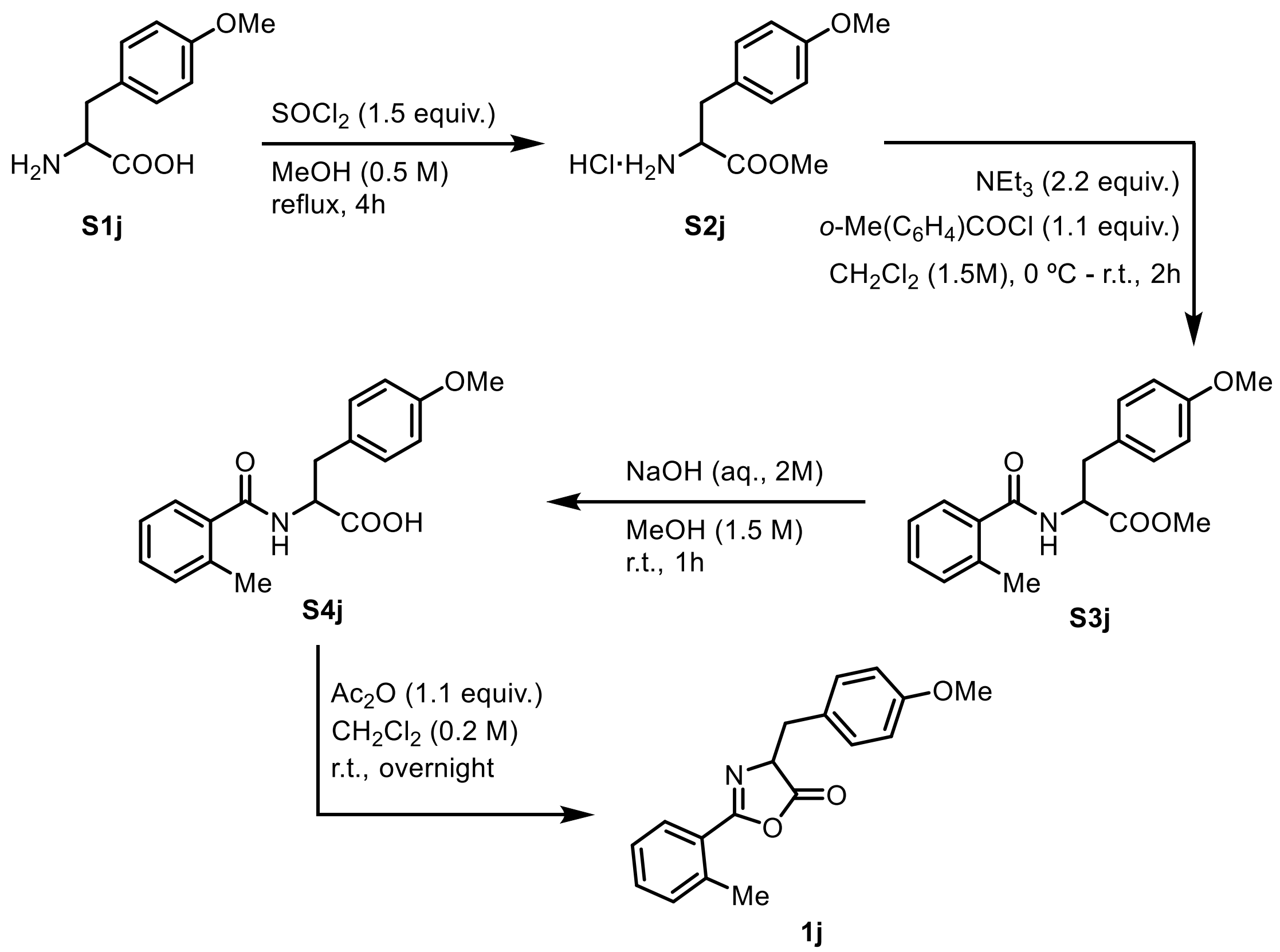

The reaction was carried out following the previously described procedures.[2,3,11]

Methyl protected *L*-tyrosine (**S1j**) was suspended in MeOH (0.5 M) and cooled down to 0 ºC. Then, $SOCl_2$ (1.5 equiv.) was carefully added dropwise. The reaction mixture was heated at 65 ºC under reflux during 4h. The solvent was removed under reduced pressure to afford a white solid, which was subsequently washed with Et_2O. The corresponding ester hydrochloride (**S2j**) was directly used in the next step.

A mixture of the corresponding methyl ester hydrochloride (**S2j**) and triethylamine (2.2 equiv.) in CH_2Cl_2 (1.5 M) was cooled down to 0 ºC. Under vigorous stirring, *o*-toluyl chloride was added dropwise. The mixture was subsequently stirred for a further 30 min at 0 °C and was then allowed to slowly warm to room temperature. After 2 h, the mixture was washed twice with HCl (1 M, aq.), twice with $NaHCO_3$ (sat., aq.) and twice with brine. The organic phase was dried over Na_2SO_4, filtered and concentrated under vacuum. The corresponding N-acyl methyl ester (**S3j**) was directly used without further purification.

S3j was then dissolved in MeOH (1.5 M) and 2 M aqueous solution of NaOH (5 mL per 10 mmol) was added at room temperature. The resulting mixture was stirred for 1 h before evaporating MeOH under reduced pressure. The resulting aqueous mixture was washed twice with CH_2Cl_2 after ensuring a basic pH. Then, it was cooled to 0 °C before conc. aq. HCl was added to acidify the solution (until pH = 0-1). The resulting acidic aqueous phase was extracted three times with CH_2Cl_2. The organic phase was dried over Na_2SO_4, filtered and concentrated under vacuum. The corresponding product (**S4j**) was dried under high vacuum and directly used in the next step.

The N-acyl derivative (**S4j**) was subsequently suspended in CH_2Cl_2 (0.1 M) and cooled to 0 °C. Ac_2O (1.1 equiv.) was slowly added into the reaction mixture. After removal of the cooling bath, the mixture

was stirred overnight at room temperature. After this time, the crude was concentrated under vacuum and pure azlactone **1j** was obtained by silica gel chromatography.

4-(4-methoxybenzyl)-2-(*o*-tolyl)oxazol-5(4*H*)-one (1j)

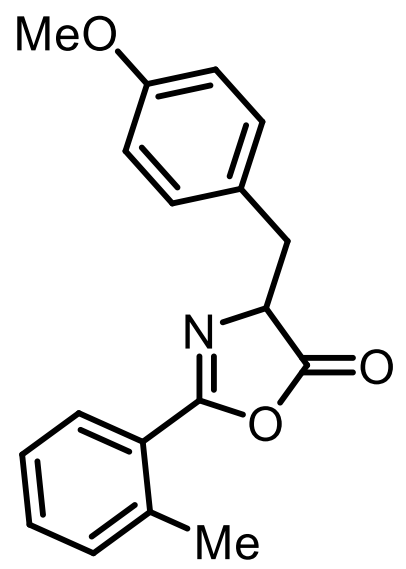

50% yield, colourless oil.

^{1}H NMR (400 MHz, $CDCl_3$) δ 7.71 (dd, *J* = 7.8, 1.5 Hz, 1H), 7.39 (td, *J* = 7.6, 1.5 Hz, 1H), 7.29 – 7.21 (m, 2H), 7.21 – 7.15 (m, 2H), 6.85 – 6.76 (m, 2H), 4.68 (dd, *J* = 6.0, 4.9 Hz, 1H), 3.75 (s, 3H), 3.34 (dd, *J* = 14.0, 4.9 Hz, 1H), 3.19 (dd, *J* = 14.0, 6.0 Hz, 1H), 2.53 (s, 3H) ppm. **^{13}C NMR** (101 MHz, $CDCl_3$) δ 177.9, 162.0, 158.9, 139.6, 131.9, 131.7, 130.9, 130.0, 127.3, 126.0, 124.9, 113.9, 67.1, 55.3, 36.5, 22.0 ppm.

B.3. Synthesis of tryptophan derived azlactone 1k

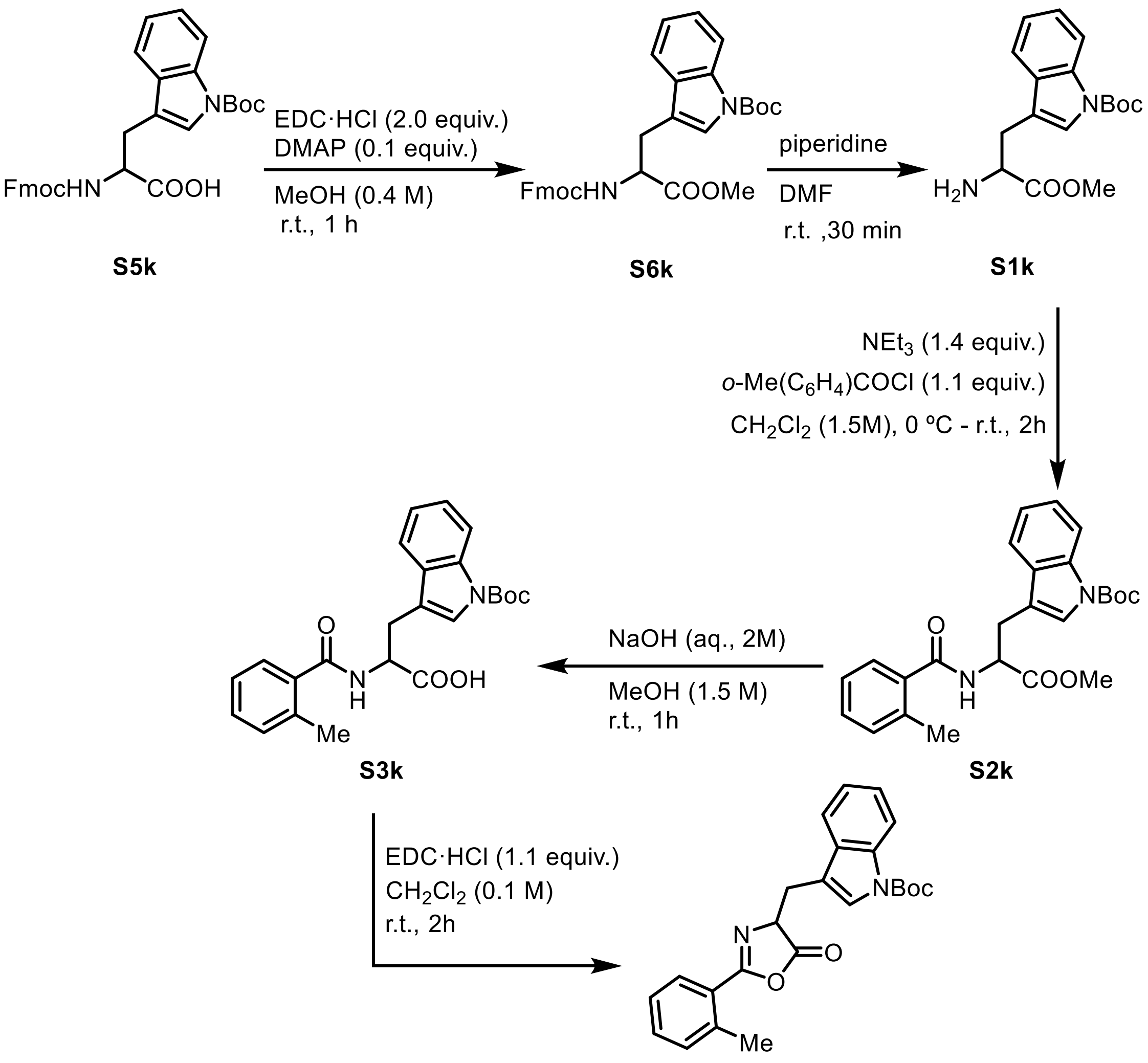

The reaction was carried out following the previously described procedures.[3,4,12]

Commercially available Fmoc-Trp(Boc)-OH (**S5k**) was dissolved in MeOH (0.4 M) and DMAP (0.1 equiv.) and EDC (2.0 equiv.) were added. The reaction was allowed to stir at room temperature for 1 h. The reaction was diluted with EtOAc and washed with HCl (1 M, aq.), Na_2CO_3 (sat., aq.), and brine. The organic layer was dried over $MgSO_4$, and the solvent was removed by rotary evaporation. The resulting compound Fmoc-Trp(Boc)-OMe (**S6k**) was used without further purification.

S6k was dissolved in a 1:1 solution of piperidine:DMF (0.25 M). The reaction was stirred at room temperature for 30 min, after which time it was diluted with EtOAc. The reaction was washed twice with Na_2CO_3 (sat., aq.), and once with brine. The organic layer was dried over $MgSO_4$, and the solvent was removed by rotary evaporation. The crude material was purified by flash chromatography (1:1 EtOAc/hexanes) to yield H_2N-Trp(Boc)-OMe (**S1k**) (1.8 g, 5.7 mmol, 67% yield).

A mixture of the methyl ester (**S1k**) and triethylamine (1.4 equiv.) in CH_2Cl_2 (1.5 M) was cooled down to 0 ºC. Under vigorous stirring, *o*-toluyl chloride was added dropwise. The mixture was subsequently stirred for further 30 min at 0 °C and was then allowed to slowly warm to room temperature. After 2 h, the mixture was washed twice with HCl (1 M, aq.), twice with $NaHCO_3$ (sat., aq.) and twice with brine. The organic phase was dried over Na_2SO_4, filtered and concentrated under vacuum. The corresponding N-acyl methyl ester (**S2k**) was directly used without further purification.

S2k was then dissolved in MeOH (1.5 M) and 2 M aqueous solution of NaOH (5 mL per 10 mmol) was added at room temperature. The resulting mixture was stirred for 1 h before evaporating MeOH under reduced pressure. The resulting aqueous mixture was washed twice with CH_2Cl_2 after ensuring a basic pH. Then it was cooled to 0 °C before conc. aq. HCl was added to acidify the solution (until pH = 3-4). The resulting acidic aqueous phase was extracted three times with CH_2Cl_2. The organic phase was dried over Na_2SO_4, filtered and concentrated under vacuum. The corresponding product (**S3k**) was dried under high vacuum and directly used in the next step.

The N-acyl derivative (**S3k**) was subsequently suspended in CH_2Cl_2 (0.1 M) and cooled to 0 °C. EDC·HCl (1.1 equiv.) was added portionwise over a 15 min period. After 30 min at 0 °C the cooling bath was removed, and the mixture was stirred for 2 h at room temperature. After this time the mixture was further diluted with CH_2Cl_2 (0.05 M) and washed with H_2O, twice with $NaHCO_3$ (sat., aq.) and twice with brine. Then, the organic phase was dried over Na_2SO_4, filtered and concentrated under vacuum. Pure azlactone **1k** was obtained by silica gel chromatography.

***tert*-butyl 3-((5-oxo-2-(*o*-tolyl)-4,5-dihydrooxazol-4-yl)methyl)-1*H*-indole-1-carboxylate (1k)**

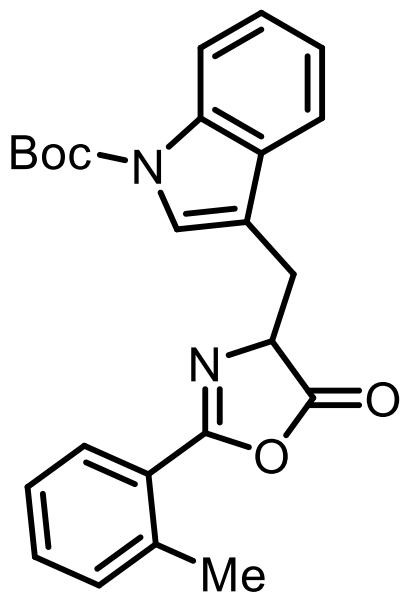

44% yield, yellow solid.

^{1}H NMR (400 MHz, $CDCl_3$) δ 8.11 (d, *J* = 8.2 Hz, 1H), 7.76 – 7.69 (m, 1H), 7.66 – 6.61 (m, 1H), 7.56 (s, 1H), 7.42 – 7.33 (m, 1H), 7.32 – 7.26 (m, 1H), 7.26 – 7.19 (m, 3H), 4.76 (dd, *J* = 6.7, 4.9 Hz, 1H), 3.49 (ddd, *J* = 14.9, 4.9, 1.0 Hz, 1H), 3.28 (ddd, *J* = 14.9, 6.8, 1.0 Hz, 1H), 2.53 (s, 3H), 1.62 (s, 9H) ppm. **^{13}C NMR** (101 MHz, $CDCl_3$) δ 177.9, 162.4, 149.6, 139.8, 135.4, 132.0, 131.8, 130.4, 130.1, 126.0, 124.8, 124.8, 124.6, 122.6, 119.4, 115.3, 114.8, 83.7, 66.1, 28.3, 26.9, 22.1 ppm.

B.4. Synthesis of methylated estrone 2i

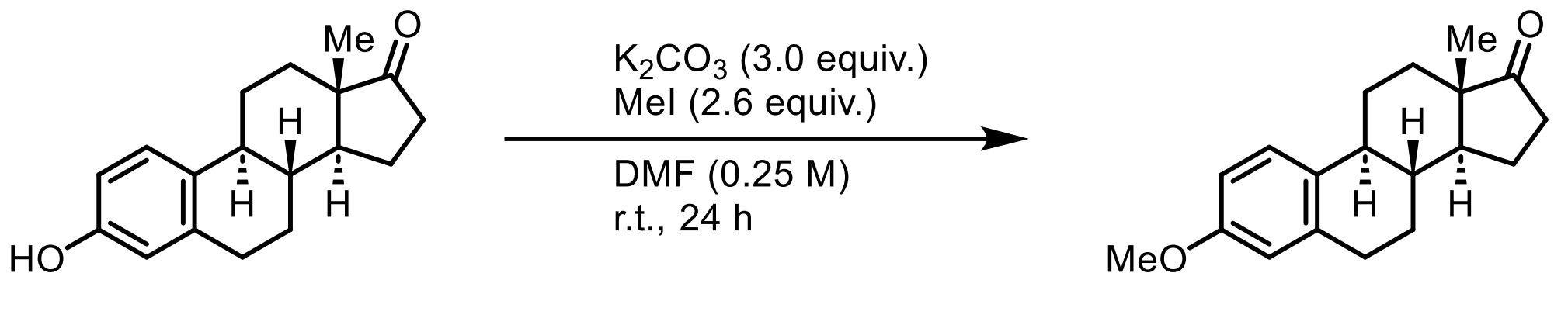

The reaction was carried out following the previously described procedure.[13]

To an oven-dried round bottom flask was added estrone (**S8**) and K_2CO_3 (3.0 equiv.). The system was purged with N_2 before DMF and MeI (2.6 equiv.) were added. The reaction mixture was stirred at room temperature for 24 h. After this time, the reaction mixture was diluted with EtOAc and H_2O. Layers were separated, and the organic layer was washed with brine three times, dried over $MgSO_4$, and filtered. The solvent was removed under reduced pressure to give **2i** (87% yield) as a white solid which was used without further purification.

(8*R*,9*S*,13*S*,14*S*)-3-Methoxy-13-methyl-6,7,8,9,11,12,13,14,15,16-decahydro-17H-cyclopenta[a]phenanthren-17-one (2i)

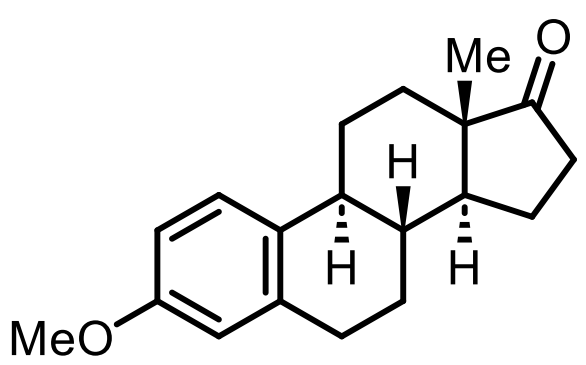

87% yield, white solid. Spectroscopic data match with those previously reported.[13]

^{1}H NMR (400 MHz, $CDCl_3$) δ 7.24 – 7.18 (m, 1H), 6.72 (dd, *J* = 8.6, 2.8 Hz, 1H), 6.65 (t, *J* = 1.9 Hz, 1H), 2.98 – 2.82 (m, 2H), 2.56 – 2.45 (m, 1H), 2.40 (dq, *J* = 9.0, 3.5 Hz, 1H), 2.31 – 2.21 (m, 1H), 2.20 – 1.93 (m, 4H), 1.69 – 1.37 (m, 6H), 0.91 (s, 3H) ppm.

B.5. General Procedure B** – Synthesis of TBS-protected silyl enol ethers* ***9

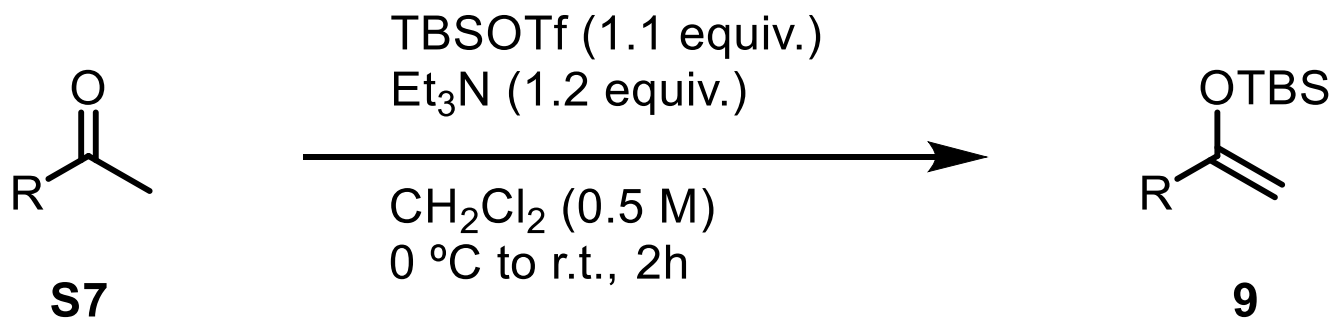

The reaction was carried out following the previously described procedure.[14]

To a flame-dried two necked round bottom flask ketone **S7** was added under nitrogen atmosphere and dissolved with freshly distilled CH_2Cl_2 (0.5 M). Subsequently, triethylamine (1.2 equiv.) was added at room temperature. The mixture was stirred for 5 minutes at room temperature. Then, the reaction mixture was cooled down to 0 °C and TBSOTf (1.1 equiv.) was added dropwise. The reaction was stirred for 2 h at room temperature and then quenched with a saturated NH_4Cl aqueous solution. The aqueous layer was extracted with hexane (3x25 mL). The combined organic layers were dried over $MgSO_4$ and concentrated under reduced pressure. The crude product **9** was purified by column chromatography on silica gel using hexane as eluent.

***tert*-butyldimethyl((1-phenylvinyl)oxy)silane (9a)**

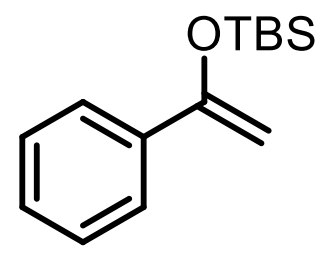

79% yield, colourless oil. Spectroscopic data match with those previously reported.[14]

^{1}H NMR (400 MHz, $CDCl_3$) δ 7.65 – 7.55 (m, 2H), 7.36 – 7.26 (m, 3H), 4.89 (d, *J* = 1.7 Hz, 1H), 4.42 (d, *J* = 1.7 Hz, 1H), 1.01 (s, 9H), 0.21 (s, 6H) ppm.

((1-(4-bromophenyl)vinyl)oxy)(*tert*-butyl)dimethylsilane (9b)

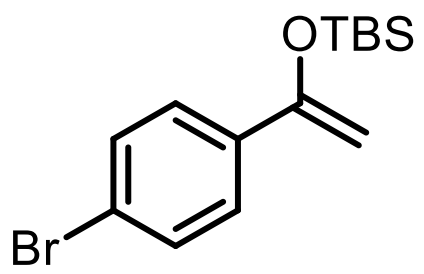

91% yield, colourless oil. Spectroscopic data match with those previously reported.[15]

^{1}H NMR (400 MHz, $CDCl_3$) δ 7.51 – 7.40 (m, 4H), 4.87 (d, *J* = 1.9 Hz, 1H), 4.43 (d, *J* = 1.9 Hz, 1H), 0.99 (s, 9H), 0.21 (s, 6H) ppm.

((1-([1,1'-biphenyl]-4-yl)vinyl)oxy)(*tert*-butyl)dimethylsilane (9c)

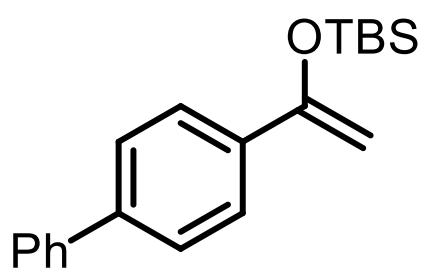

94% yield, white solid. Spectroscopic data match with those previously reported.[14]

^{1}H NMR (400 MHz, Acetone) δ 7.79 – 7.72 (m, 2H), 7.72 – 7.63 (m, 4H), 7.52 – 7.42 (m, 2H), 7.41 – 7.31 (m, 1H), 5.05 (d, *J* = 1.9, 1H), 4.50 (d, *J* = 1.9 Hz, 1H), 1.04 (s, 9H), 0.27 (s, 6H).

***tert*-butyl((1-(4-(*tert*-butyl)phenyl)vinyl)oxy)dimethylsilane (9d)**

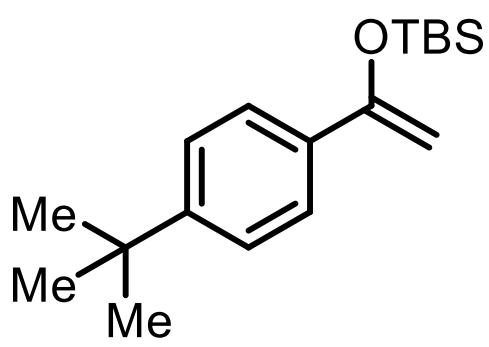

81% yield, colourless oil. Spectroscopic data match with those previously reported.[16]

^{1}H NMR (400 MHz, $CDCl_3$) δ 7.58 – 7.51 (m, 2H), 7.39 – 7.31 (m, 2H), 4.85 (t, *J* = 1.5 Hz, 1H), 4.36 (t, *J* = 1.5 Hz, 1H), 1.32 (s, 9H), 1.01 (s, 9H), 0.21 (s, 6H) ppm.

***tert*-butyldimethyl((1-(naphthalen-2-yl)vinyl)oxy)silane (9e)**

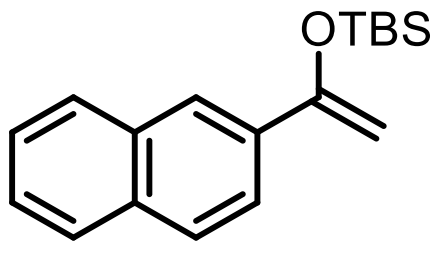

90% yield, white solid. Spectroscopic data match with those previously reported.[16]

^{1}H NMR (400 MHz, $CDCl_3$) δ 8.11 – 8.06 (m, 1H), 7.89 – 7.74 (m, 3H), 7.71 (dd, *J* = 8.6, 1.8 Hz, 1H), 7.52 – 7.41 (m, 2H), 5.04 (d, *J* = 1.9 Hz, 1H), 4.54 (d, *J* = 1.7 Hz, 1H), 1.10 – 1.00 (m, 10H), 0.24 (t, *J* = 0.5 Hz, 6H) ppm.

***tert*-butyl((1-(furan-2-yl)vinyl)oxy)dimethylsilane (9f)**

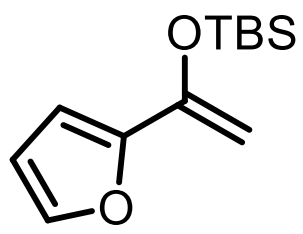

90% yield, colourless oil. Spectroscopic data match with those previously reported.[17]

^{1}H NMR (400 MHz, $CDCl_3$) δ 7.34 (dd, *J* = 1.8, 0.9 Hz, 1H), 6.41 (dd, *J* = 3.3, 0.9 Hz, 1H), 6.37 (dd, *J* = 3.3, 1.8 Hz, 1H), 4.87 (d, *J* = 1.6 Hz, 1H), 4.35 (d, *J* = 1.6 Hz, 1H), 0.99 (s, 9H), 0.21 (s, 6H) ppm.

((1-(benzofuran-2-yl)vinyl)oxy)(*tert*-butyl)dimethylsilane (9g)

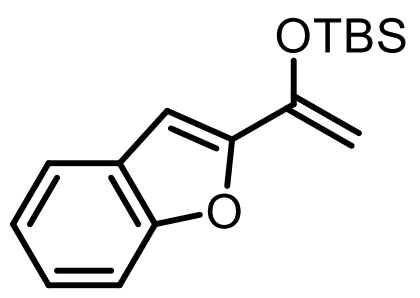

87% yield, white solid. Spectroscopic data match with those previously reported.[15]

^{1}H NMR (400 MHz, $CDCl_3$) δ 7.55 (ddd, *J* = 7.6, 1.4, 0.7 Hz, 1H), 7.44 (dq, *J* = 8.2, 1.0 Hz, 1H), 7.27 (ddd, *J* = 8.2, 7.2, 1.4 Hz, 1H), 7.20 (ddd, *J* = 7.7, 7.3, 1.1 Hz, 1H), 6.78 (d, *J* = 1.0 Hz, 1H), 5.18 (d, *J* = 1.7 Hz, 1H), 4.56 (d, *J* = 1.7 Hz, 1H), 1.03 (s, 9H), 0.25 (s, 6H) ppm.

B.6. Synthesis of the TBS-protected silyl enol ether from acetylated pregnenolone 9h

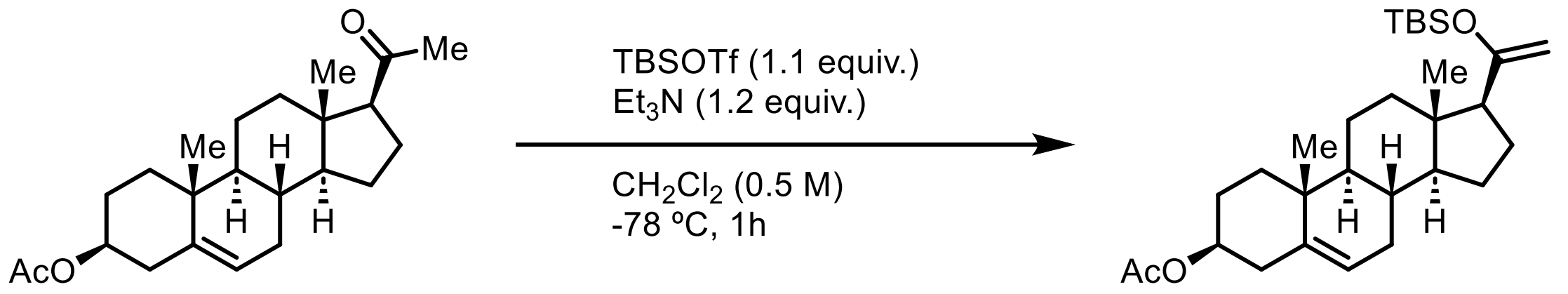

S9 **9h**

The reaction was carried out following the previously described procedure.[14]

To a flame-dried two necked round bottom flask ketone **S9** was added under nitrogen atmosphere and dissolved with freshly distilled CH_2Cl_2 (0.5 M). The reaction mixture was cooled to -78 °C. Then, triethylamine (1.2 equiv.) and TBSOTf (1.1 equiv.) were added dropwise. The reaction was stirred for 1 h and then quenched with a saturated NH_4Cl aqueous solution at room temperature. The aqueous layer was extracted with CH_2Cl_2. The combined organic layers were dried over $MgSO_4$ and concentrated under reduced pressure. The crude product was purified by column chromatography on silica gel using hexane as eluent to afford **9h** (68% yield) as a white solid.

Spectroscopic data match with those previously reported.[14]

B.7. Synthesis of trimethyl(2-phenylallyl)silane (11)

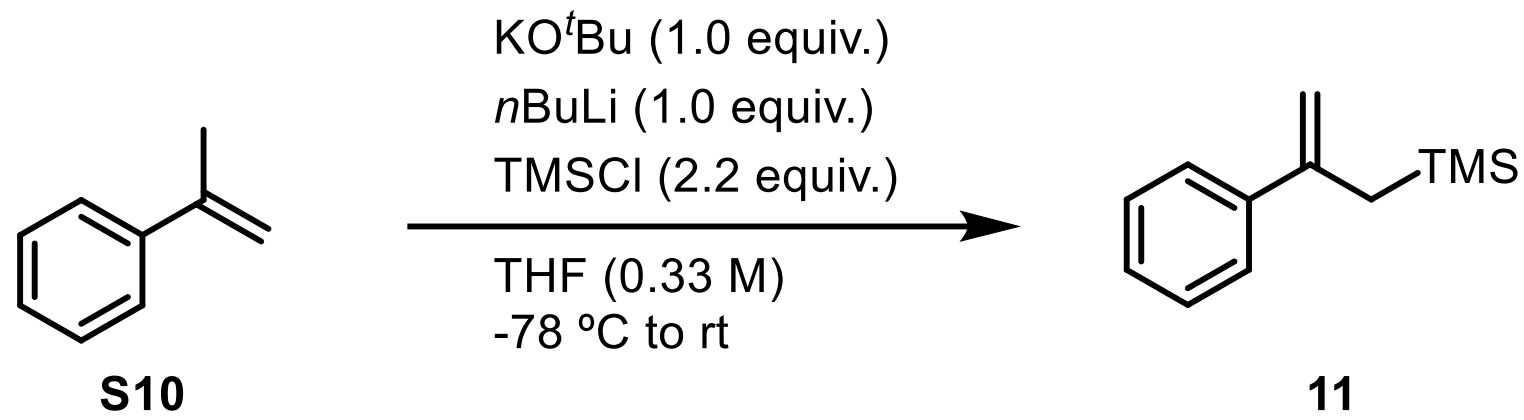

The reaction was carried out following the previously described procedure.[18]

KO^{t}Bu (1.0 equiv) and α-methylstyrene (**S10**, 1.0 equiv.) were dissolved in THF (0.33 M) and the resulting solution was cooled down to –78 °C. Subsequently, *n*BuLi (2.5M in hexane, 1.0 equiv) was added over a period of 5-10 minutes at this temperature. The solution was warmed to –50 °C and stirred at this temperature for 5 h. The resulting solution was then added to a solution of chlorotrimethylsilane (2.2 equiv) in THF (2.4 M) at –78 °C. The reaction mixture was allowed to warm to room temperature and stirred overnight. The solvent was removed under reduced pressure and the residue taken up in *n*-pentane. The resulting suspension was filtered through a pad of Celite® and concentrated under reduced pressure. The residue was purified by column chromatography on silica using hexane as eluent. The desired allylsilane was obtained as a colourless oil (22% yield).

Trimethyl(2-phenylallyl)silane (11)

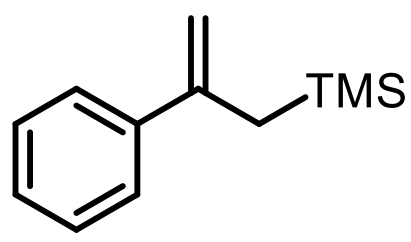

22% yield, colourless oil. Spectroscopic data match with those previously reported.[18]

^{1}H NMR (400 MHz, $CDCl_3$) δ 7.43 – 7.37 (m, 2H), 7.33 – 7.27 (m, 2H), 7.26 – 7.22 (m, 1H), 5.12 (d, *J* = 1.7 Hz, 1H), 4.86 (dt, *J* = 1.8, 1.1 Hz, 1H), 2.02 (d, *J* = 1.1 Hz, 2H), -0.10 (s, 9H) ppm.

B.8. Synthesis of Potassium* trans-*Styryl Trifluoroborate (13)

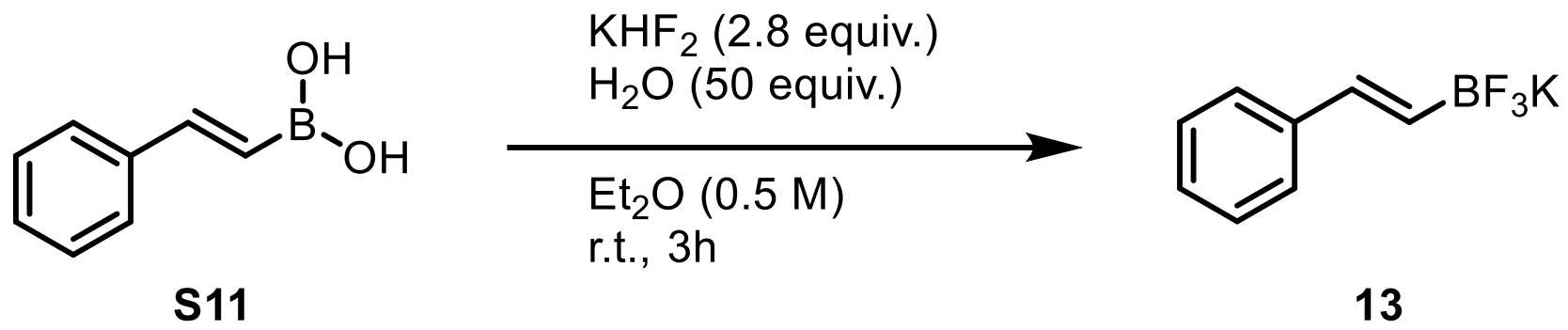

The reaction was carried out following the previously described procedure.[19]

To a solution of *trans*-2-phenylethenyl boronic acid **S11** in Et_2O (0.5 M) was added KHF_2 (2.8 equiv.), followed by H_2O (50 equiv.) over a period of 30 min. After stirring at rt for 3 h, the solution was concentrated and the crude material was dissolved in acetone, filtered, and concentrated. The resulting white solid was purified by dissolving it in hot acetone and precipitating with Et_2O, resulting in a white solid (78% yield).

Potassium *trans*-Styryl Trifluoroborate (13)

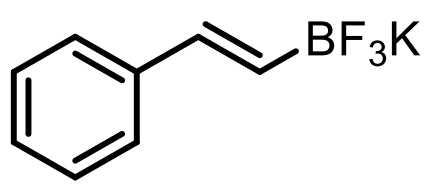

78% yield, white solid. Spectroscopic data match with those previously reported.[20]

^{1}H NMR (400 MHz, Acetone) δ 7.39 – 7.30 (m, 2H), 7.27 – 7.17 (m, 2H), 7.07 (ddt, J = 7.2, 5.5, 1.3 Hz, 1H), 6.70 – 6.56 (m, 1H), 6.41 – 6.24 (m, 1H) ppm.

B.9. General Procedure C** – Synthesis of triarylamine electrocatalysts **3a-3e

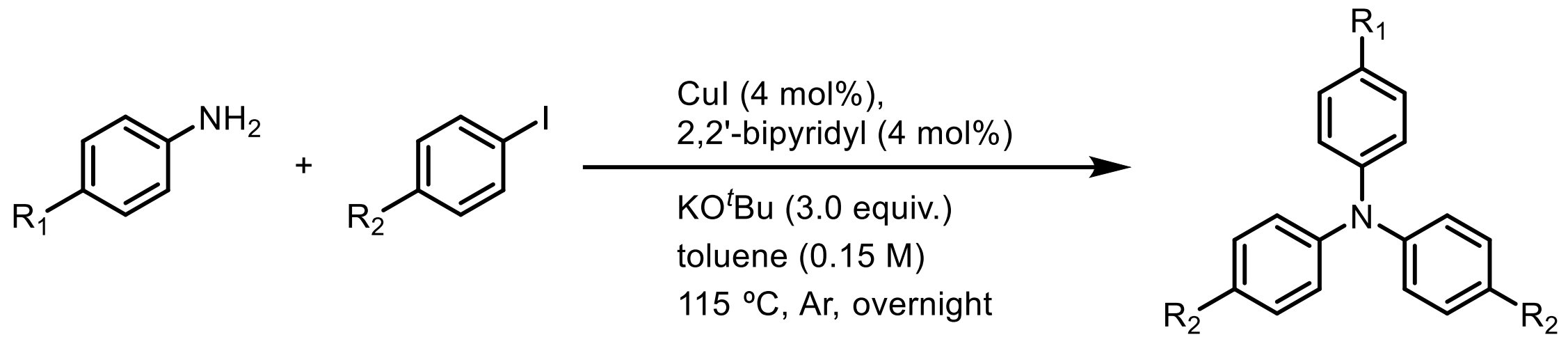

The reaction was carried out following the previously described procedure.[21]

The corresponding aniline (**S12**) and iodobenzene (**S13**, 2.1 equiv.) were added into a sealed tube, which was then purged with N_2. After, the mixture was dissolved with previously degassed toluene (0.15 M). Copper(I) iodide (4 mol %) and 2,2'-bipyridyl (4 mol %) were added to the solution, and the reaction vessel was placed in an oil bath and heated to 115 ºC. Once the right temperature was reached, potassium tert-butoxide (3.0 equiv.) was added and the reaction was left overnight at 115 ºC. Then, the reaction was allowed to cool down to room temperature. Solids were removed by vacuum filtration, and solvent was removed under reduced pressure. The crude was dissolved in CH_2Cl_2 and washed twice with $NaHSO_3$ (0.1 M, aq.), and once with H_2O. The crude products were purified by column chromatography on silica gel using hexane as eluent, followed by recrystallisation from MeOH.

tris(4-chlorophenyl)amine (3a)

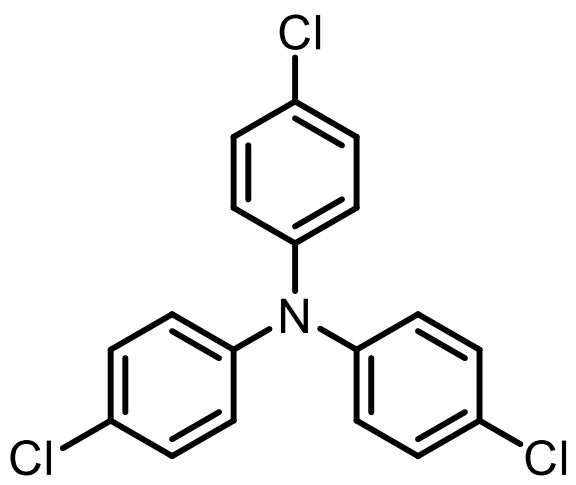

45% yield, white solid. Spectroscopic data match with those previously reported.[22]

^{1}H NMR (400 MHz, $CDCl_3$) δ 7.25 – 7.15 (m, 6H), 7.03 – 6.91 (m, 6H) ppm.

4-(*tert*-butyl)-*N*,*N*-bis(4-chlorophenyl)aniline (3b)

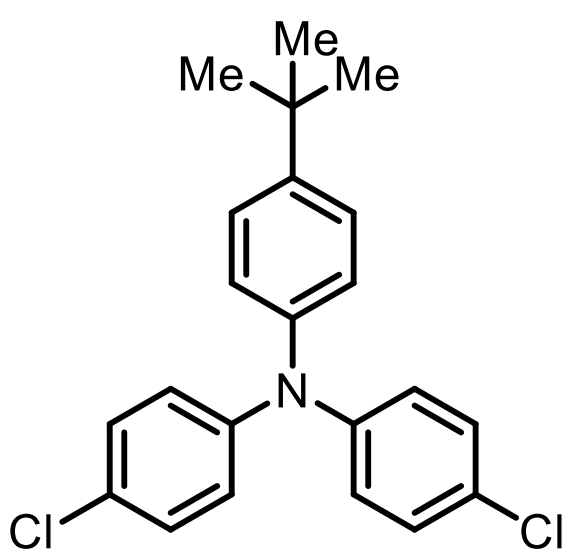

40% yield, white solid.

^{1}H NMR (400 MHz, $CDCl_3$) δ 7.28 – 7.24 (m, 2H), 7.21 – 7.15 (m, 4H), 7.00 – 6.95 (m, 6H), 1.31 (s, 9H) ppm. **^{13}C NMR** (101 MHz, $CDCl_3$) δ 146.9, 146.4, 144.4, 129.4, 127.6, 126.5, 124.9, 124.4, 34.5, 31.5 ppm. **HRMS (ESI)** Calculated for $C_{22}H_{21}Cl_2N^{\cdot+}$ ($[M]^{\cdot+}$) 369.1046. Found 369.1049.

4-chloro-*N*-(4-chlorophenyl)-*N*-(4-methoxyphenyl)aniline (3c)

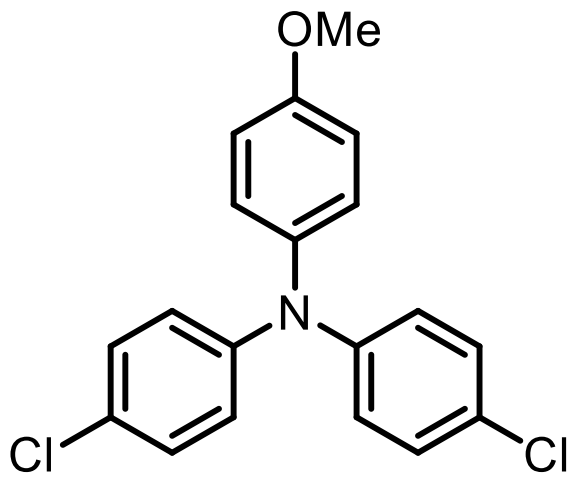

38% yield, white solid.

^{1}H NMR (400 MHz, Acetone) δ 7.32 – 7.21 (m, 4H), 7.12 – 7.05 (m, 2H), 7.03 – 6.91 (m, 6H), 3.81 (s, 3H) ppm. **^{13}C NMR** (101 MHz, Acetone) δ 158.1, 147.6, 140.5, 130.1, 128.6, 127.3, 124.7, 116.0, 55.8 ppm. **HRMS (ESI)** Calculated for $C_{19}H_{15}Cl_2NO^{\cdot+}$ ($[M]^{\cdot+}$) 343.0525. Found 343.0526.

4-chloro-*N*,*N*-bis(4-methoxyphenyl)aniline (3d)

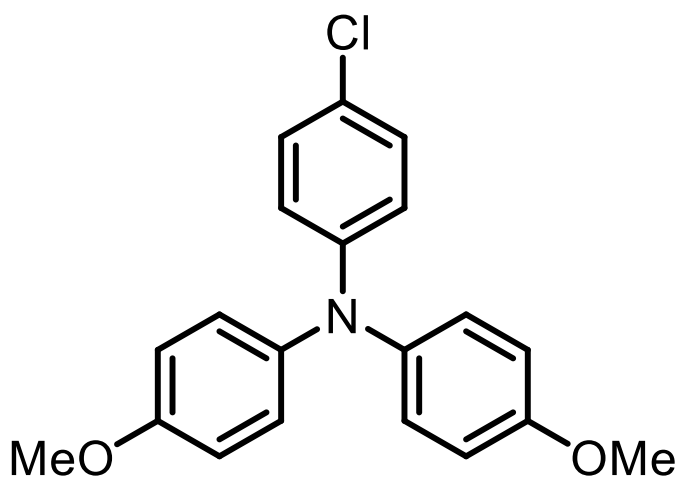

48% yield, white solid.

^{1}H NMR (400 MHz, $CDCl_3$) δ 7.14 – 7.07 (m, 2H), 7.05 – 7.00 (m, 4H), 6.87 – 6.80 (m, 6H), 3.79 (s, 6H) ppm. **^{13}C NMR** (101 MHz, $CDCl_3$) δ 156.1, 147.6, 140.8, 129.0, 126.6, 125.2, 121.9, 114.9, 55.6 ppm. **HRMS (ESI)** Calculated for $C_{20}H_{18}ClNO_2^{\cdot+}$ ($[M]^{\cdot+}$) 339.1021. Found 339.1022.

tris(4-methoxyphenyl)amine (3e)

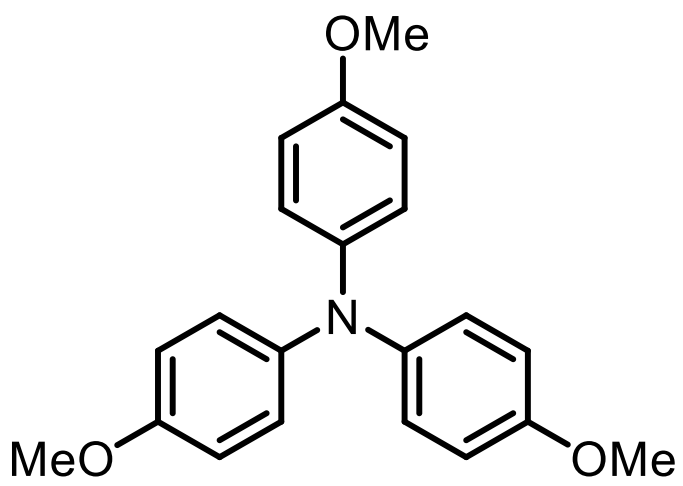

75% yield, white needles. Spectroscopic data match with those previously reported.[22]

^{1}H NMR ^{1}H NMR (400 MHz, Acetone) δ 6.97 – 6.87 (m, 6H), 6.87 – 6.75 (m, 6H), 3.74 (s, 9H) ppm.

B.10. Synthesis of tris(4-bromophenyl)amine 3f

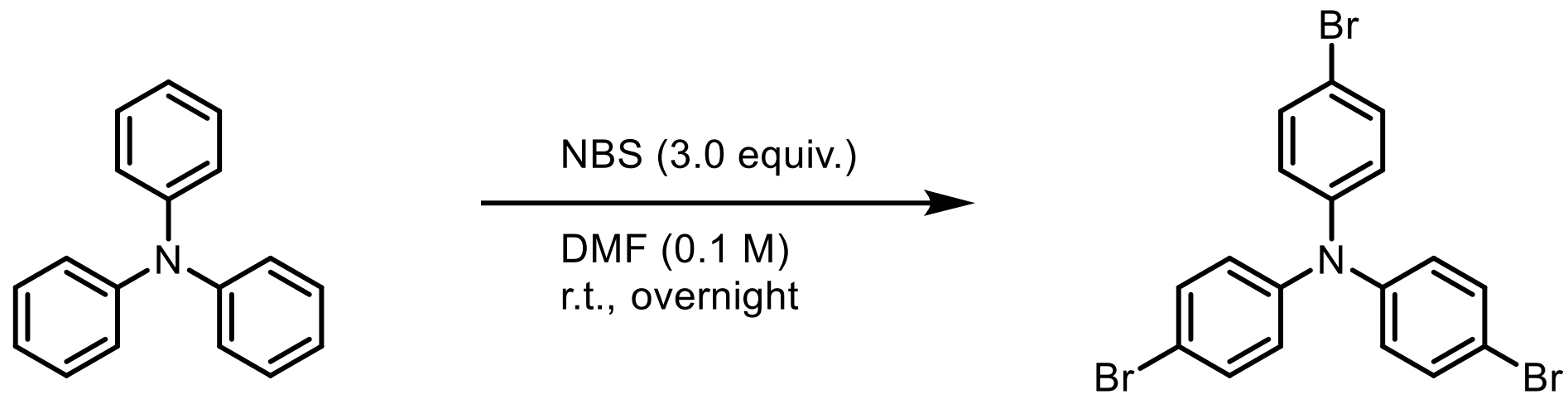

S14 **3f**

The reaction was carried out following the previously described procedure.[23]

To a solution of triphenylamine (**S14**) in DMF (0.2 M) was added a solution of *N*-bromosuccinimide (NBS; 3.0 equiv.) in DMF (0.8 M) at room temperature, and the resulting mixture was stirred overnight. The mixture was then poured into H_2O and the crude product was extracted with CH_2Cl_2. The combined organic extracts were dried over $MgSO_4$, filtered, and concentrated under vacuum. The crude product was washed with cold MeOH to afford the desired product as a white solid (74% yield).

tris(4-bromophenyl)amine (3f)

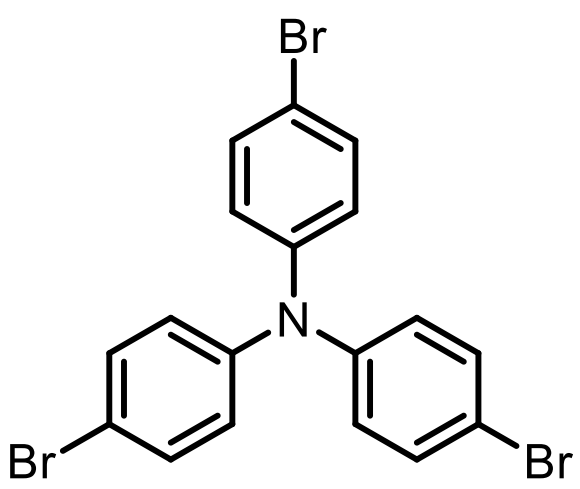

74% yield, white solid. Spectroscopic data match with those previously reported.[23]

^{1}H NMR (500 MHz, $CDCl_3$) δ 7.42 – 7.30 (m, 6H), 6.99 – 6.88 (m, 6H) ppm.

*B.11. Synthesis of 4-bromo-*N*-(4-bromophenyl)-*N*-(4-(*tert*-butyl)phenyl)aniline 3g*

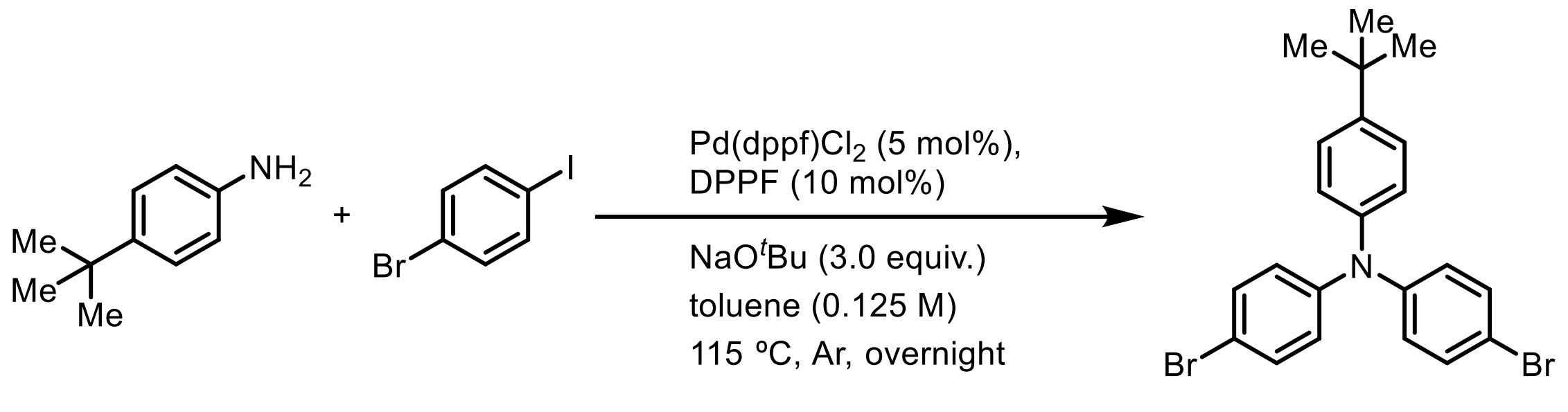

The reaction was carried out following the previously described procedure.[24]

Aniline **S12g**, 4-bromoiodobenzene (**S13g**, 2.1 equiv.), Pd(dppf)Cl_2 (5 mol%), 1,1′-Ferrocenediyl-bis(diphenylphosphine) (DPPF, 10 mol%) and NaO^{t}Bu (3.0 equiv.) were added into a sealed tube, which was subsequently purged with Ar. Then, previously degassed toluene was added and the tube was sealed, placed in an oil bath and heated to 115 ºC overnight. Then, the reaction was allowed to cool down to room temperature. Solids were removed by vacuum filtration, and solvent was removed under reduced pressure. The crude was dissolved in CH_2Cl_2 and washed twice with saturated NH_4Cl aqueous solution, dried over $MgSO_4$, filtered, and evaporated under reduced pressure. The crude products were purified by column chromatography on silica gel using hexane as eluent, followed by recrystallisation from MeOH.

tris(4-chlorophenyl)amine (3g)

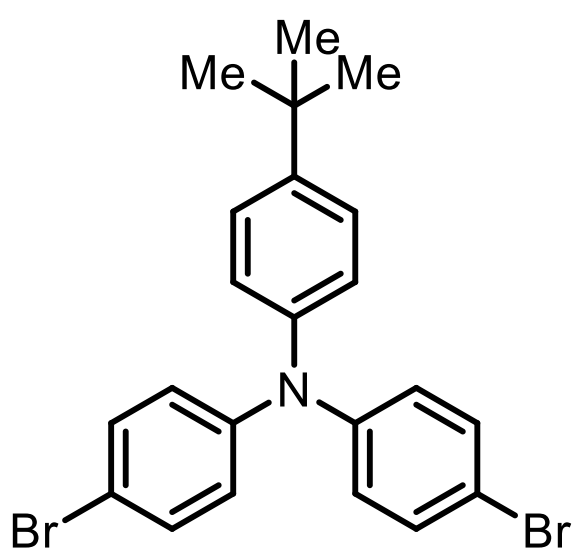

47% yield, white solid.

^{1}H NMR (400 MHz, Acetone) δ 7.48 – 7.35 (m, 6H), 7.08 – 6.99 (m, 2H), 6.99 – 6.91 (m, 4H), 1.31 (s, 9H) ppm. **^{13}C NMR** (101 MHz, Acetone) δ 148.0, 147.8, 145.0, 133.2, 127.5, 126.0, 125.7, 115.5, 34.9, 31.7. **HRMS (ESI)** Calculated for $C_{22}H_{21}Br_2N^{\cdot+}$ ($[M]^{\cdot+}$) 457.0035. Found 457.0040.

B.12. General Procedure D – *Synthesis of pyridine derived supporting electrolytes*

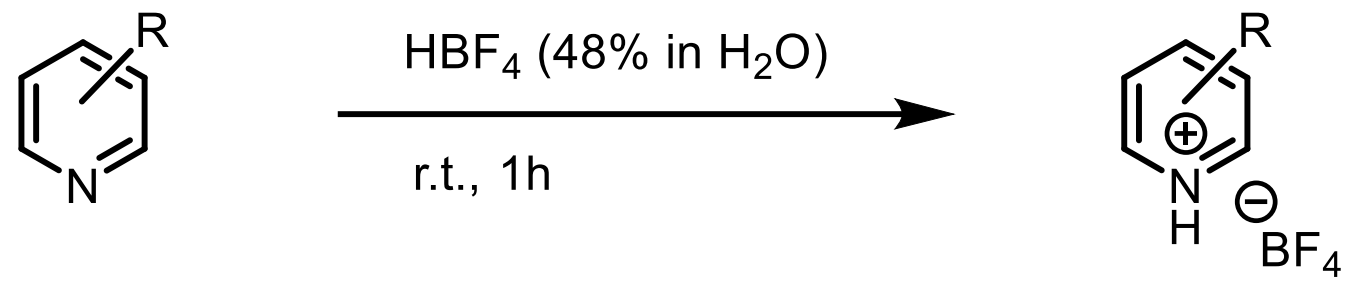

The reaction was carried out following the previously described procedure.[25]

To a round bottomed flask containing tetrafluoroboric acid solution (1.0 equiv.), the corresponding pyridine was slowly added (*note*: exothermic). After addition, the reaction mixture was left to stir for 1 hour at room temperature. Then, the solvent was removed under reduced pressure. The resulting solid was washed three times with Et_2O to afford a white crystalline solid.

2,4,6-collidine·HBF$_4$

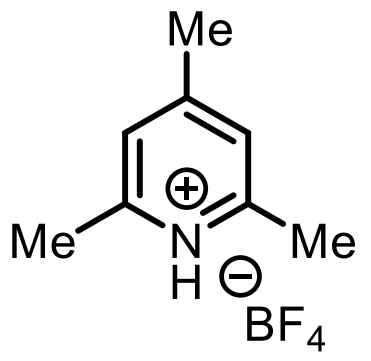

>99% yield, white solid. Spectroscopic data match with those previously reported.[26]

^{1}H NMR (400 MHz, $CDCl_3$) δ 7.31 (s, 2H), 2.80 (s, 6H), 2.58 (s, 3H) ppm.

pyridine·HBF$_4$

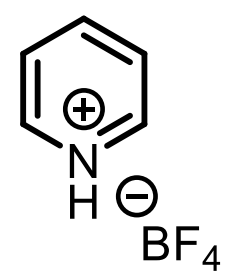

90% yield, white solid. Spectroscopic data match with those previously reported.[27]

^{1}H NMR (400 MHz, DMSO) δ 8.95 – 8.89 (m, 2H), 8.65 – 8.56 (m, 1H), 8.08 (t, J = 7.1 Hz, 2H) ppm.

2,6-lutidine·HBF$_4$

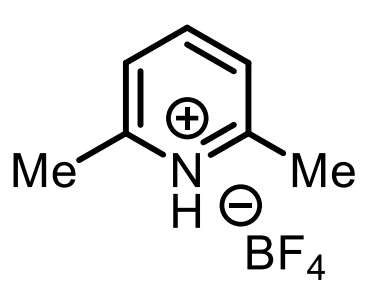

90% yield, white solid. Spectroscopic data match with those previously reported.[28]

^{1}H NMR (400 MHz, $CDCl_3$) δ 8.23 (t, J = 7.9 Hz, 1H), 7.56 (d, J = 7.9 Hz, 2H), 2.85 (s, 6H) ppm.

2,4-lutidine·HBF$_4$

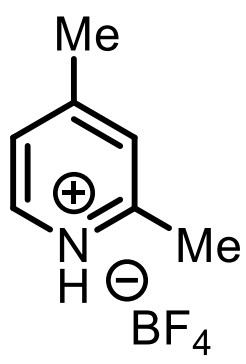

75% yield, white solid.

^{1}H NMR (400 MHz, $CDCl_3$) δ 13.36 (bs, 1Hz), 8.52 (t, J = 6.3 Hz, 1H), 7.61 (d, J = 6.3 Hz, 1H), 7.53 (s, 1H), 2.82 (s, 3H), 2.65 (s, 3H) ppm. **^{13}C NMR** (101 MHz, $CDCl_3$) δ 160.6, 153.1, 140.7, 128.3, 125.6, 22.5, 19.7 ppm. **^{19}F NMR** (400 MHz, $CDCl_3$) δ –150.4 (s), –150.5 (s) ppm.

4-picoline·HBF_4

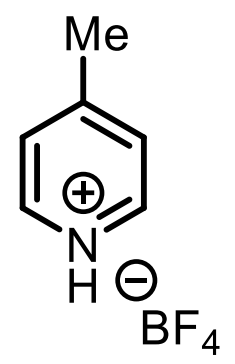

90% yield, white solid.

^{1}H NMR (400 MHz, $CDCl_3$) δ 8.78 – 8.72 (m, 2H), 7.83 – 7.78 (m, 2H), 2.71 (s, 3H) ppm. **^{13}C NMR** (101 MHz, $CDCl_3$) δ 161.2, 141.3, 140.7, 128.3, 128.1, 22.8 ppm. **^{19}F NMR** (376 MHz, $CDCl_3$) δ –150.1 (s), –151.0 ppm.

4-methoxypyridine·HBF_4

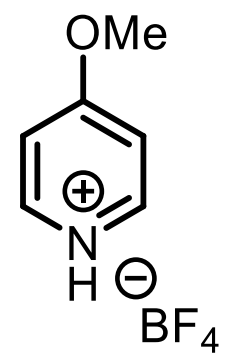

57% yield, white solid.

^{1}H NMR (400 MHz, DMSO) δ 8.79 – 8.73 (m, 2H), 7.61 – 7.53 (m, 2H), 4.09 (s, 3H) ppm. **^{13}C NMR** (101 MHz, DMSO) δ 171.6, 143.3, 112.8, 57.8 ppm. **^{19}F NMR** (376 MHz, DMSO) δ –148.2 (s), –148.2 (s) ppm.

C. Optimisation of the electrocatalytic reaction

C.1. Optimisation – Reaction design

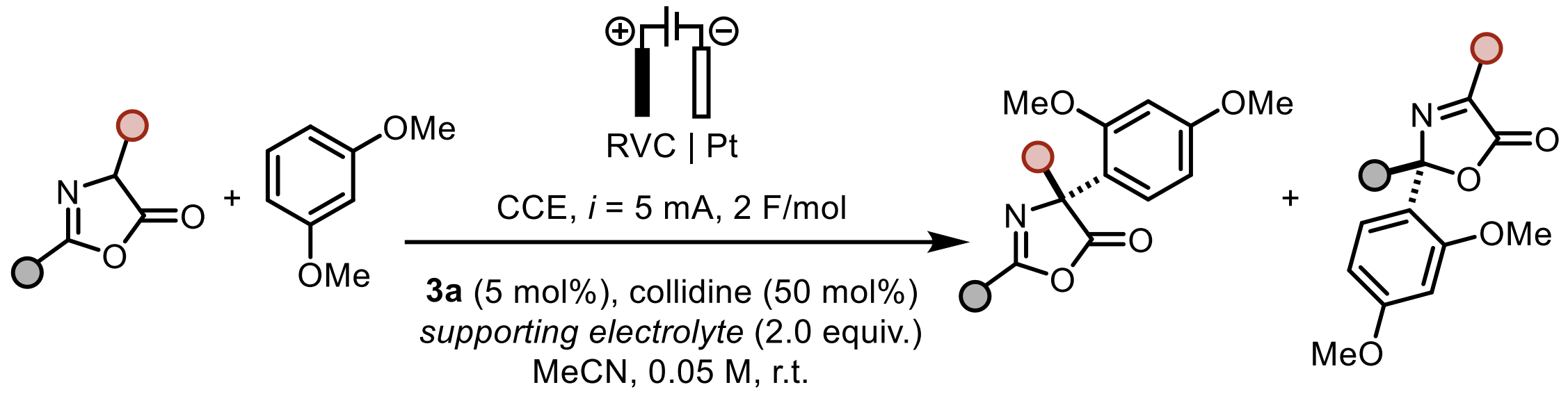

entry	1 – (○,○)	supporting electrolyte	conv. (%)[a]	yield **4** (%)[a]	r.r. (**4**/**5**)
1	**1a** – ^{i}Pr, Ph	$TBAPF_6$ + HFIP (4 equiv.)	98	25	3:1
2	**1a** – ^{i}Pr, Ph	col·HBF_4	94	45	3:1
3	**1b** – ^{i}Pr, ^{t}Bu	col·HBF_4	40	-	-
4	**1c** – ^{i}Pr, Mes	col·HBF_4	74	24	>20:1
5	**1d** – ^{i}Pr, *o*-Tol	col·HBF_4	87	41	16:1
6	**1e** – Me, *o*-Tol	col·HBF_4	99	68	>20:1
7	**1f** – ^{i}Bu, *o*-Tol	col·HBF_4	**95**	**59**	**>20:1**

Table S1. Reaction and substrate design. [a] Yield and conversion were determined by ^{1}H NMR spectroscopy using 1,3,5-trimethoxybenzene as the internal standard.

A maximum regioselectivity of 3:1 was achieved with a phenyl group at C2 (Table S1, entry 2), so azlactones **1b**, bearing a *tert*-butyl substituent at C2, was tested to assess if the increase of steric hindrance at C2 could improve the selectivity towards C4 (Table S1, entry 3). However, during electrolysis, the reaction mixture rapidly developed a deep blue colour, suggesting that the activated electrocatalyst **3a**$^{•+}$ is unable to efficiently oxidise the enolate of azlactone **1b**. Simultaneously, the cell potential increased to approximately 4 V at early stages, while in a successful reaction the cell potential remains constant at 2-2.3 V. The elevated cell potential increases the anodic oxidation power, leading to direct oxidation of both the azlactone **1b** and 1,3-dimethoxybenzene **2a**. Analysis of the crude reaction mixture by ^{1}H NMR revealed no traces of the corresponding product, complete consumption of the electrocatalyst, and showed that only 0.6 equivalents of 1,3-dimethoxybenzene remained from the initial 3 equivalents employed.

A bulky aromatic group such as mesityl greatly improved the regioselectivity but decreased the yield significantly (Table S1, entry 4). Finally, an *o*-tolyl group at C2 of the azlactone proved to be key to achieve perfect regioselectivity without significantly losing reactivity (Table S1, entry 5).

The Leucine-derived azlactone **1f**, bearing an ^{i}Bu group at the C4 position, showed a moderate steric hindrance effect, making it optimal to be the model substrate for optimisation (Table S1, entry 7).

C.2. Optimisation – Solvent screening

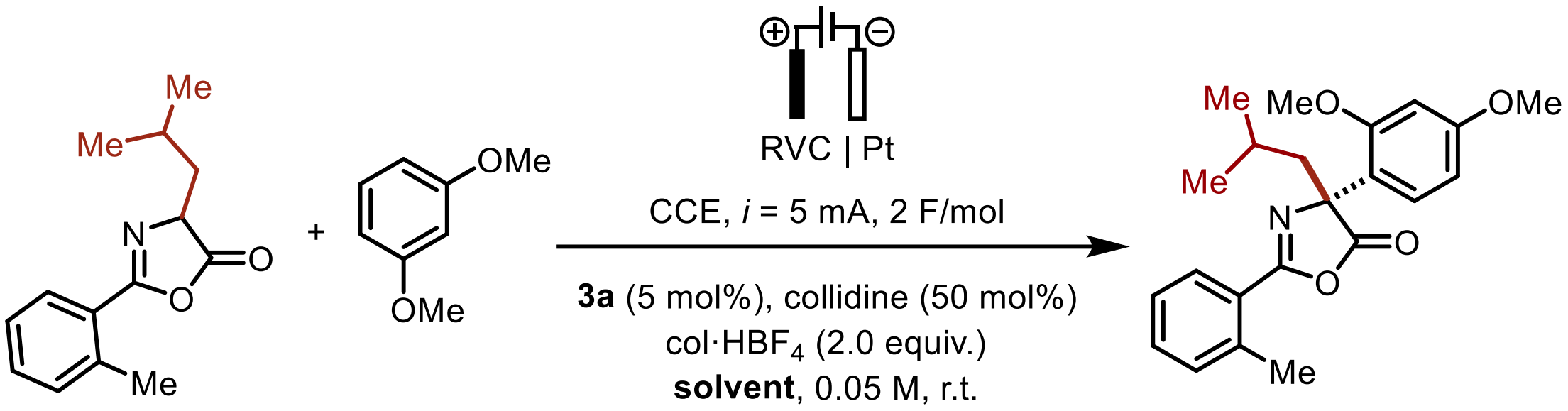

1f **2a** (3.0 equiv.) **4f**

entry	solvent	conv. (%)[a]	yield **4f** (%)[a]
**1**	**MeCN**	**95**	**59**
**2**	CH_2Cl_2	96	48
**3**	DCE	82	22
**4**	THF[b]	10	5
**5**	DME	17	n.d.
**6**	DMF	76	5
**7**	Acetone	71	5
**8**	MeCN/DMF (9:1)	73	13
**9**	MeCN/DMSO (9:1)	84	n.d.

Table S2. Optimisation of the solvent. [a] Yield and conversion were determined by ^{1}H NMR spectroscopy using 1,3,5-trimethoxybenzene as the internal standard. [b] col·HBF_4 showed a very low solubility in THF. DCE: dichloroethane, THF: tetrahydrofuran, DMF: dimethylformamide, DME: 1,2-dimethoxyethane.

*C.3. **Optimisation** – Electrocatalyst screening*

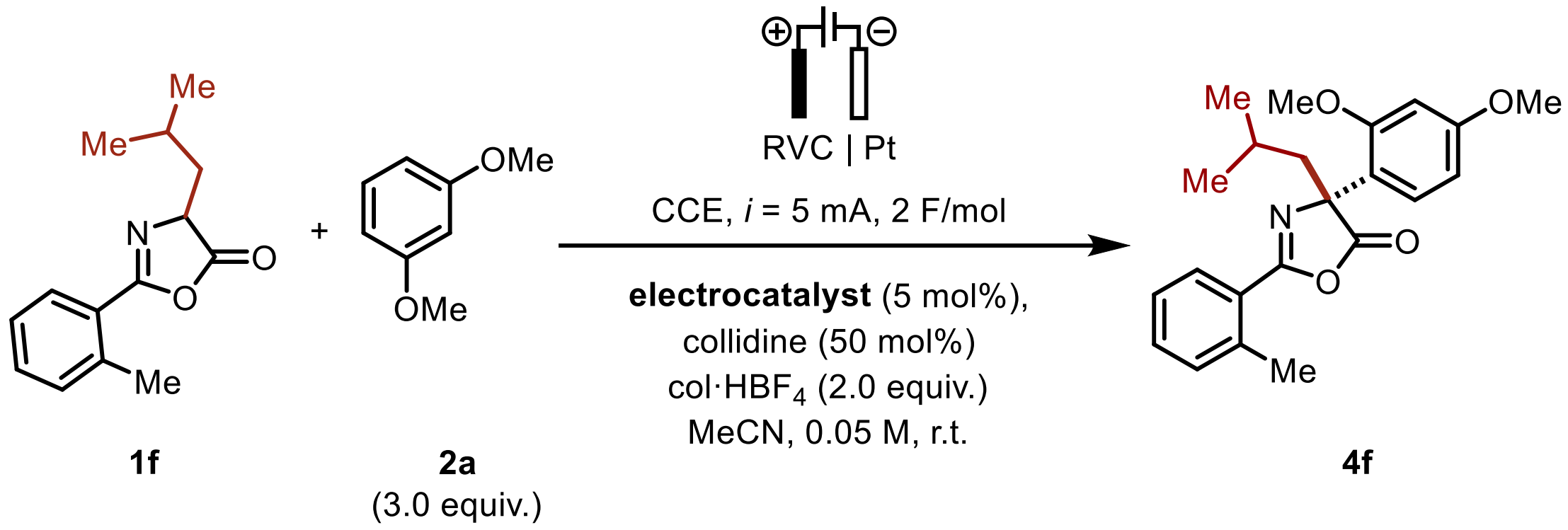

entry	electrocatalyst	conv. (%)[a]	yield **4f** (%)[a]
**1**	**3a**	**95**	**59**
**2**	**3b**	96	57
**3**	**3c**	92	38
**4**	**3d**	91	24
**5**	**3e**	88	15
**6**	**3f**	98	54
**7**	**3g**	97	49
**8**	**3h**	90	41
**9**	**3i**	21	5
**10**	**3j**	90	-

Table S3. Optimisation of the electrocatalyst used in this study. [a] Yield and conversion were determined by ^{1}H NMR spectroscopy using 1,3,5-trimethoxybenzene as the internal standard.

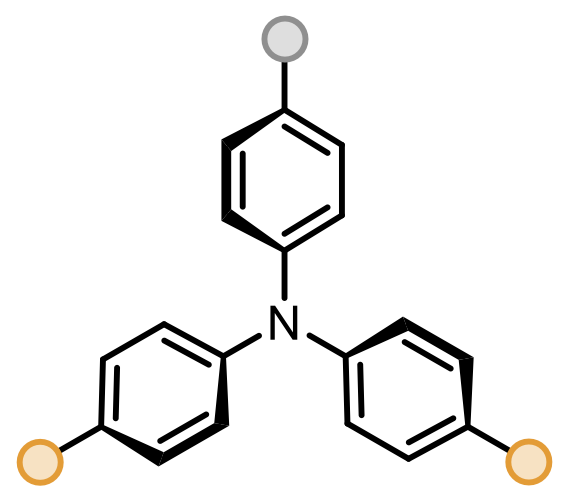

	,	E$_{1/2}$ *vs.* SCE (V)
**3a**	Cl, Cl	+ 1.09
**3b**	Cl, ^{t}Bu	+ 1.00
**3c**	Cl, OMe	+ 0.88
**3d**	OMe, Cl	+ 0.75
**3e**	OMe, OMe	+ 0.57
**3f**	Br, Br	+ 1.11
**3g**	Br, ^{t}Bu	+ 1.02
**3h**	Br, OMe	+ 0.90

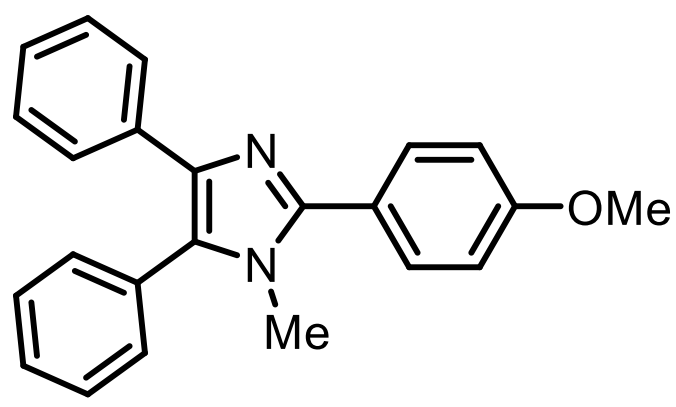

3i, E$_{1/2}$ = + 1.11 V *vs.* SCE

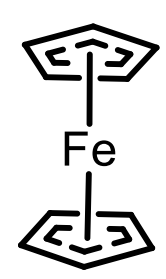

3j, E$_{1/2}$ = + 0.41 V *vs.* SCE

Figure S1. Electrocatalysts **3a-j** and their corresponding $E_{1/2}$ *vs.* SCE.

The use of less oxidizing electrocatalysts results in high azlactone conversion with a marked decrease in product yield (Table S3, entries 3–5).

As the difference between the oxidation potential of the azlactone enolate (*E*p) and the oxidation potential of the electrocatalyst ($E_{1/2}$) increases, indirect electron transfer from enolate **6** to the activated electrocatalyst $\mathbf{3}^{\cdot+}$ becomes progressively less thermodynamically favourable and therefore occurs to a lesser extent. Consequently, regeneration of the neutral electrocatalyst **3** becomes less efficient, leading to accumulation of the catalyst in its oxidised form ($\mathbf{3}^{\cdot+}$). Experimentally, this is evidenced by the homogeneous blue colour that develops during the early stages of electrolysis (Figure 5Bii).

Under these conditions, the concentration of the neutral electrocatalyst **3** decreases, reducing its oxidation at the anode. To maintain the constant current of 5 mA, the cell potential raises substantially, thereby increasing the oxidising power of the anode. As a result, direct oxidation of the azlactone at the electrode becomes increasingly competitive. Although this pathway still consumes the starting material, it does not promote the selective electrocatalytic activation required for productive C–C bond formation. This is consistent with the control experiment performed in the absence of electrocatalyst (Figure 2Bi, entry 6), in which 50% of azlactone **1f** was consumed, whereas only trace amounts of the desired product **4f** were detected.

At the same time, the oxidised electrocatalyst $\mathbf{3}^{\cdot+}$, which is unable to efficiently oxidise the azlactone enolate **6** under these conditions while also not oxidising the electron-rich coupling partner undergoes gradual decomposition. This is evidenced by the ^{1}H NMR analysis of the reaction crudes which confirm substantial decomposition of the electrocatalysts.

C.4. ***Optimisation*** *– Electrocatalyst loading*

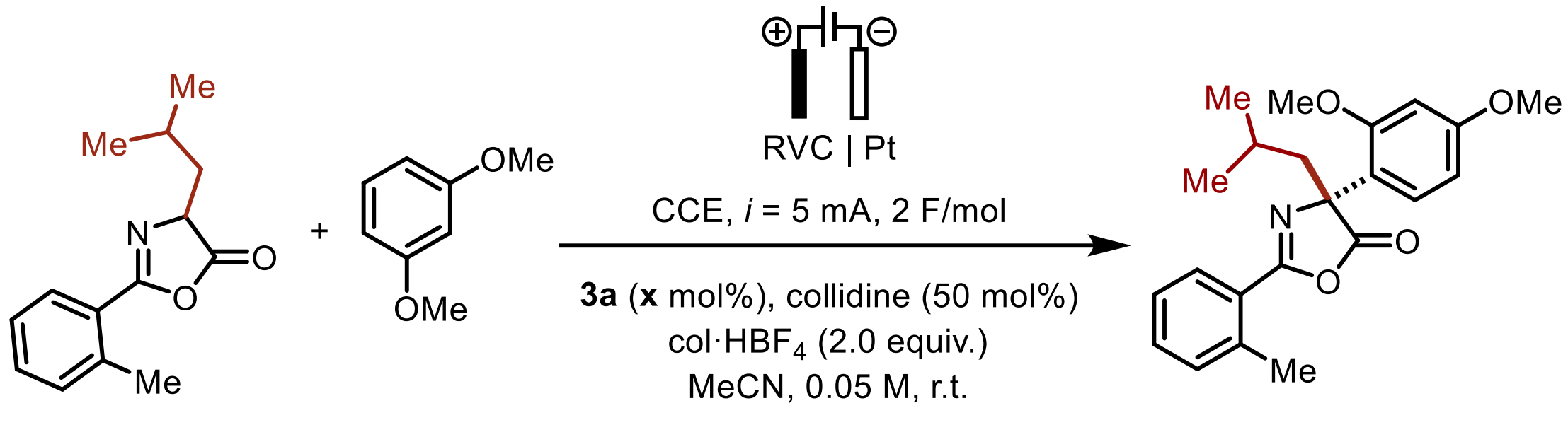

1f **2a** (3.0 equiv.) **4f**

entry	mol% electrocatalyst **3a**	conv. (%)[a]	yield **4f** (%)[a]
**1**	0	50	5
**2**	1	64	18
**3**	2	69	28
**4**	3	93	46
**5**	4	92	52
**6**	**5**	**95**	**59**
**7**	10	96	59
**8**	20	95	60

Table S4. Optimisation of the electrocatalyst loading. [a] Yield and conversion were determined by ^{1}H NMR spectroscopy using 1,3,5-trimethoxybenzene as the internal standard.

C.5. Optimisation – *Base and supporting electrolyte*

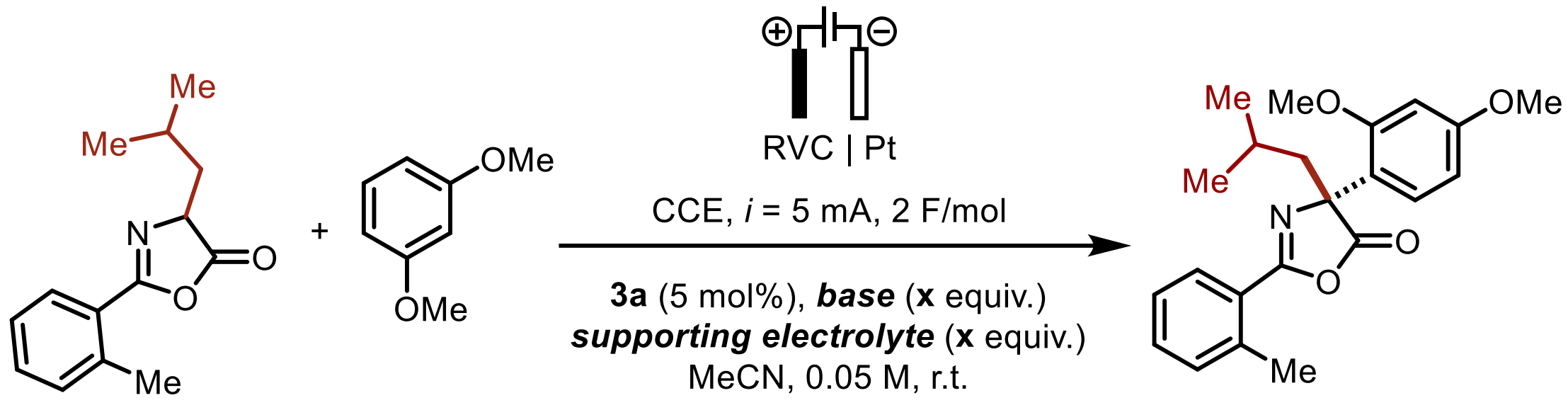

1f **2a** (3.0 equiv.) **4f**

**entry**	base	base equiv.	supporting electrolyte	supporting electrolyte equiv.	conv. (%)[a]	yield **4** (%)[a]
**1**	**collidine**	**0.5**	**col·HBF$_4$**	**2.0**	**95**	**59**
**2**	pyridine	0.5	pyr·HBF$_4$	2.0	30	8
**3**	2,6-lutidine	0.5	2,6-lut HBF$_4$	2.0	92	47
**4**	2,4-lutidine	0.5	2,4-lut HBF$_4$	2.0	80	29
**5**	4-picoline	0.5	4-pic HBF$_4$	2.0	75	18
**6**	4-OMepyridine	0.5	4-OMepyr·HBF$_4$	2.0	79	16
**7**	collidine	0.25	col·HBF$_4$	2.0	94	52
**8**	collidine	1.0	col·HBF$_4$	2.0	99	43
**9**	collidine	2.0	col·HBF$_4$	2.0	99	34
**11**	collidine	3.0	col·HBF$_4$	2.0	99	30
**12**	collidine	0.5	col·HBF$_4$	0.5	96	55
**13**	collidine	0.5	col·HBF$_4$	1.0	95	50
**14**	collidine	0.5	col·HBF$_4$	4.0	97	52
**15**	collidine	0.5	col·HBF$_4$	6.0	89	50
**16**	collidine	0.5	col·HBF$_4$ + *n*Bu$_4$NPF$_6$	2.0 + 1.0	94	51
**17**	collidine	0.5	col·HBF$_4$ + *n*Bu$_4$NBF$_4$	2.0 + 1.0	92	45
**18**	collidine	0.5	col·HBF$_4$ + *n*Bu$_4$NClO$_4$	2.0 + 1.0	97	50

Table S5. Optimisation of the base/supporting electrolyte system. [a] Yield and conversion were determined by ^{1}H NMR spectroscopy using 1,3,5-trimethoxybenzene as the internal standard.

C.6. ***Optimisation*** *– Electrodes, current intensity, SOMOphile equivalents, concentration*

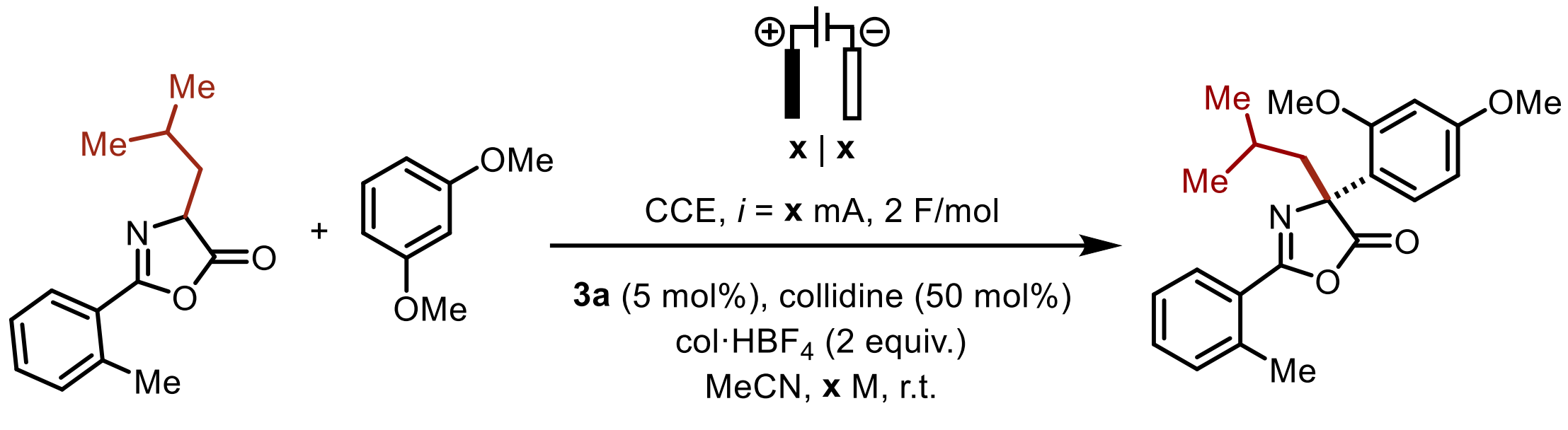

1f **2a** (**x** equiv.) **4f**

**entry**	electrodes	current intensity (mA)	SOMOphile equivalents	concentration (M)	conv. (%)[a]	yield **4** (%)[a]
**1**	RVC (+) | Pt (–)	5	3	0.05	95	59
**2**	C (+) | Pt (–)	5	3	0.05	54	7
**3**	GC (+) | Pt (–)	5	3	0.05	52	9
**4**	RVC (+) | SS (–)	5	3	0.05	91	8
**5**	RVC (+) | Pt (–)	3	3	0.05	99	56
**6**	RVC (+) | Pt (–)	5	1	0.05	95	40
**7**	RVC (+) | Pt (–)	5	2	0.05	96	50
**8**	RVC (+) | Pt (–)	5	5	0.05	98	47
**9**	RVC (+) | Pt (–)	5	10	0.05	97	43
**10**	**RVC (+) | Pt (–)**	**5**	**3**	**0.025**	**95**	**70 (68)**

Table S6. Final tuning of the reaction conditions. [a] Yield and conversion were determined by ^{1}H NMR spectroscopy using 1,3,5-trimethoxybenzene as the internal standard. RVC: reticulated vitreous carbon, GC: glassy carbon.

D. Electrocatalytic Functionalisation of Azlactones – General Procedure E

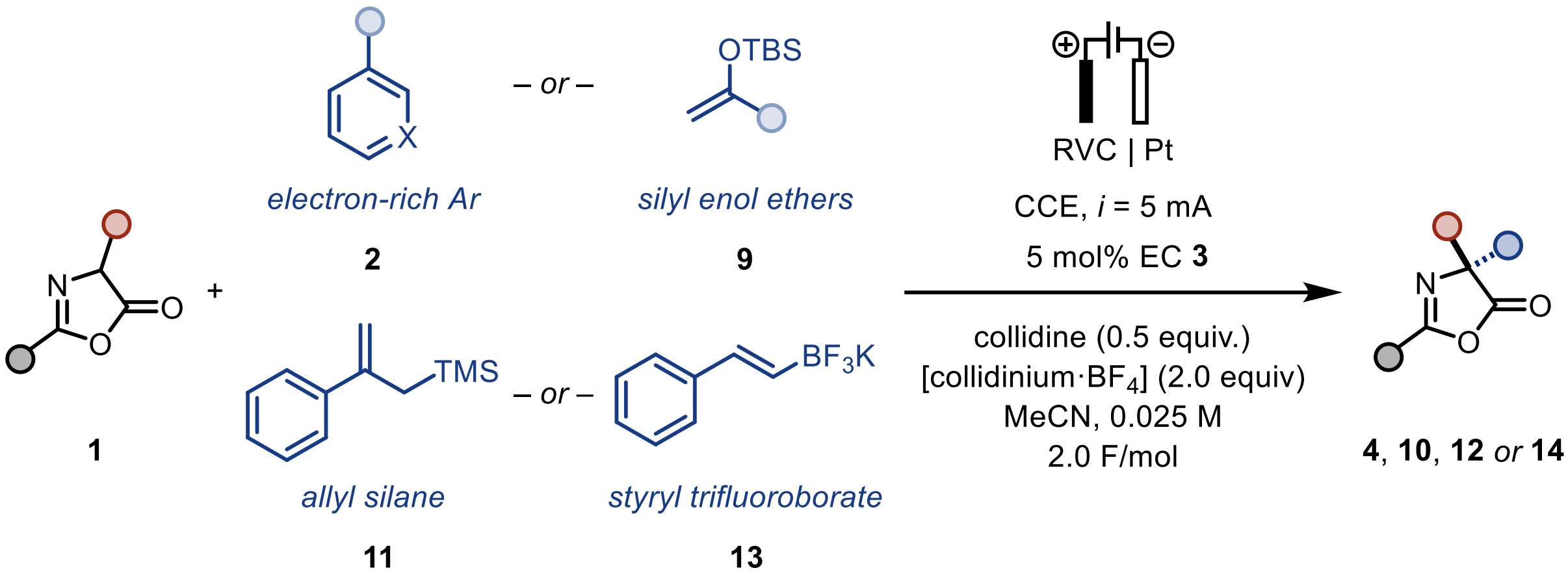

A 10 mL ElectraSyn vial with a magnetic stirring bar was charged with the corresponding azlactone (**1**, 0.2 mmol, 1.0 equiv.), the corresponding radical trap (0.6 mmol, 3.0 equiv.), the corresponding electrocatalyst (**3**, 0.01 mmol, 5 mol%), and [collidinium·BF_4] (0.4 mmol, 2.0 equiv.). The ElectraSyn vial cap equipped with anode (RVC) and cathode (platinum foil) was inserted and the system was purged with N_2. Subsequently, MeCN (8 mL, 0.025 M) and collidine (0.1 mmol, 0.5 equiv.) were added. The resulting mixture was stirred and electrolysed at constant current (5 mA) until the solution became completely deep blue, indicating full consumption of the starting material. Then the electrodes were rinsed with CH_2Cl_2, and the crude mixture was directly purified by flash column chromatography on silica gel.

4-(2,4-dimethoxyphenyl)-4-isopropyl-2-(*o*-tolyl)oxazol-5(4*H*)-one (4d)

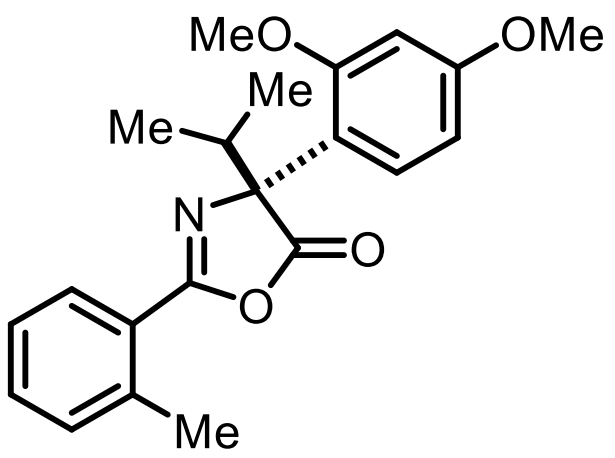

***4d** was prepared following a slightly modified General Procedure E from azlactone **1d** (43.4 mg, 0.2 mmol) and 1,3-dimethoxybenzene (**2a**, 79 µL, 0.6 mmol) with electrocatalyst **3a** (3.5 mg, 0.01 mmol), [collidinium·BF_4] (83.6 mg, 0.4 mmol) and collidine (26 µL,* 0.2 mmol*) in MeCN (*4 mL*). The product was isolated by chromatography on silica gel (hexanes/ethyl acetate = 8:1 to 6:1).*

58% yield (41 mg, 0.12 mmol), white solid. **^{1}H NMR** (400 MHz, $CDCl_3$) δ 7.90 – 7.85 (m, 1H), 7.47 – 7.37 (m, 2H), 7.32 – 7.26 (m, 2H), 6.55 (dd, *J* = 8.7, 2.5 Hz, 1H), 6.48 (d, *J* = 2.5 Hz, 1H), 3.80 (s, 3H), 3.75 (s, 3H), 3.04 (hept, *J* = 6.7 Hz, 1H), 2.65 (s, 3H), 1.14 (d, *J* = 6.8 Hz, 3H), 1.09 (d, *J* = 6.8 Hz, 3H) ppm; **^{13}C NMR** (101 MHz, $CDCl_3$) δ 179.1, 161.1, 161.0, 159.1, 139.3, 131.7, 131.5, 129.9, 129.3, 126.0, 125.8, 118.1, 105.1, 99.8, 75.6, 55.8, 55.5, 31.9, 21.9, 17.4, 16.7 ppm; **HRMS (ESI)** Calculated for $C_{21}H_{24}NO_4^+$ ($[M+H]^+$) 354.1700. Found 354.1698.

4-(2,4-dimethoxyphenyl)-4-methyl-2-(*o*-tolyl)oxazol-5(4*H*)-one (4e)

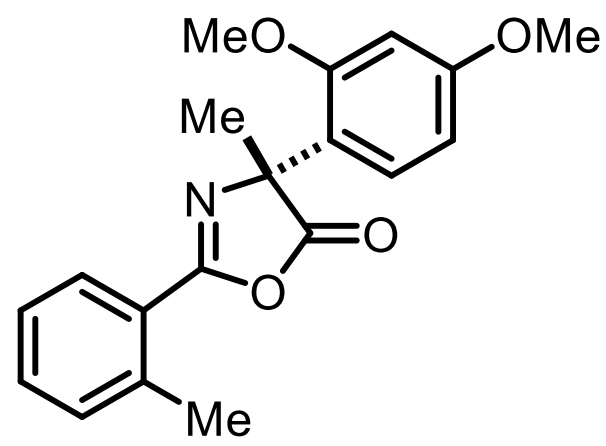

***4e** was prepared following General Procedure E from azlactone **1e** (37.8 mg, 0.2 mmol) and 1,3-dimethoxybenzene (**2a**, 79 μL, 0.6 mmol) with electrocatalyst **3a** (3.5 mg, 0.01 mmol), [collidinium·BF_4] (83.6 mg, 0.4 mmol) and collidine (13 μL, 0.1 mmol) in MeCN (8 mL). The product was isolated by chromatography on silica gel (hexanes/ethyl acetate = 5:1 to 4:1).*

79% yield (51 mg, 0.16 mmol), white solid. **^{1}H NMR** (400 MHz, $CDCl_3$) δ 7.90 – 7.85 (m, 1H), 7.48 – 7.37 (m, 2H), 7.32 – 7.27 (m, 2H), 6.56 (dd, *J* = 8.7, 2.5 Hz, 1H), 6.45 (d, *J* = 2.5 Hz, 1H), 3.81 (s, 3H), 3.71 (s, 3H), 2.61 (s, 3H), 1.87 (s, 3H) ppm; **^{13}C NMR** (101 MHz, $CDCl_3$) δ 181.3, 161.6, 161.3, 158.5, 139.3, 131.7, 131.6, 129.8, 128.2, 126.0, 125.9, 118.7, 105.0, 99.4, 68.2, 55.7, 55.5, 23.0, 21.8 ppm; **HRMS (ESI)** Calculated for $C_{19}H_{20}NO_4^+$ ($[M+H]^+$) 326.1398. Found 326.1385.

4-(2,4-dimethoxyphenyl)-4-isobutyl-2-(*o*-tolyl)oxazol-5(4*H*)-one (4f)

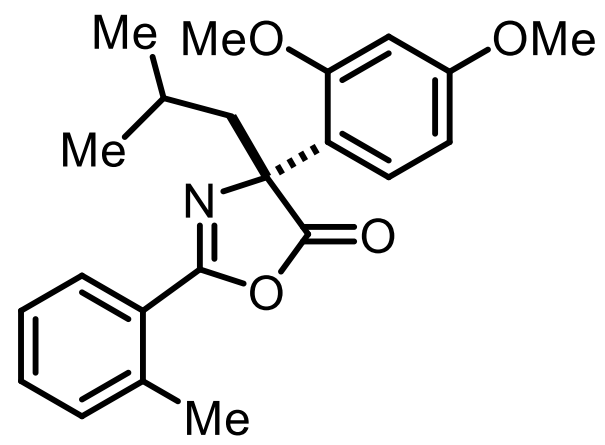

***4f** was prepared following General Procedure E from azlactone **1f** (46.2 mg, 0.2 mmol) and 1,3-dimethoxybenzene (**2a**, 79 μL, 0.6 mmol) with electrocatalyst **3a** (3.5 mg, 0.01 mmol), [collidinium·BF_4] (83.6 mg, 0.4 mmol) and collidine (13 μL, 0.1 mmol) in MeCN (8 mL). The product was isolated by chromatography on silica gel (hexanes/ethyl acetate = 6:1).*

68% yield (50 mg, 0.14 mmol), yellowish oil. **^{1}H NMR** (400 MHz, $CDCl_3$) δ 7.92 (dd, *J* = 8.2, 1.5 Hz, 1H), 7.47 (d, *J* = 8.8 Hz, 1H), 7.42 (td, *J* = 7.3, 1.5 Hz, 1H), 7.34 – 7.28 (m, 2H), 6.54 (dd, *J* = 8.8, 2.5 Hz, 1H), 6.45 (d, *J* = 2.5 Hz, 1H), 3.79 (s, 3H), 3.70 (s, 3H), 2.66 (s, 3H), 2.52 (dd, *J* = 13.5, 5.5 Hz, 1H), 2.15 (dd, *J* = 13.5, 5.5 Hz, 1H), 1.71 (hept, *J* = 6.7,z 1H), 1.03 (d, *J* = 6.7 Hz, 6H) ppm; **^{13}C NMR** (101 MHz, $CDCl_3$) δ 180.8, 161.3, 161.3, 158.6, 139.4, 131.7, 131.6, 130.0, 128.2, 126.0, 125.8, 119.4, 104.9, 99.5, 71.8, 55.7, 55.5, 44.0, 25.0, 24.3, 24.2, 21.9 ppm. **HRMS (ESI)** Calculated for $C_{22}H_{25}NO_4^+$ ($[M+H]^+$) 368.1856. Found 368.1854.

4-(*sec*-butyl)-4-(2,4-dimethoxyphenyl)-2-(*o*-tolyl)oxazol-5(4*H*)-one (4g)

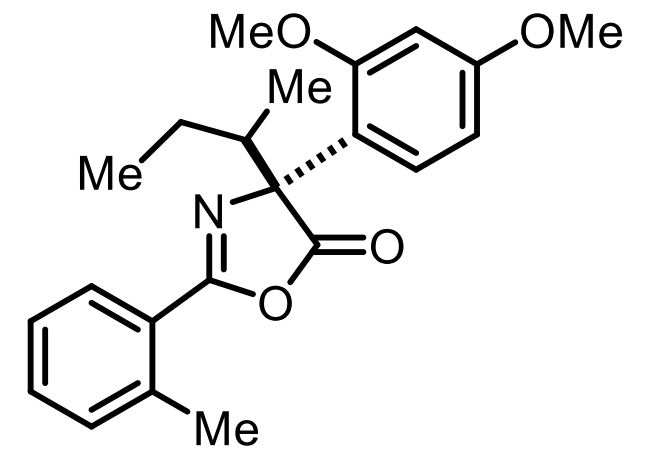

***4g** was prepared following a slightly modified General Procedure E from azlactone **1g** (46.2 mg, 0.2 mmol) and 1,3-dimethoxybenzene (**2a**, 79 μL, 0.6 mmol) with electrocatalyst **3a** (3.5 mg, 0.01 mmol), [collidinium·BF_4] (83.6 mg, 0.4 mmol) and collidine (26 μL,* 0.2 mmol*) in MeCN (*4 mL*). The product was isolated by chromatography on silica gel (hexanes/ethyl acetate = 9:1).*

62% yield (37 mg, 0.14 mmol), colourless oil. *This product has been characterized as a mixture of diastereoisomers* (dr = 2:1). **^{1}H NMR** (400 MHz, $CDCl_3$) *δ* (for major diastereomer) 7.90 – 7.83 (m, 1H), 7.43 – 7.37 (m, 1H), 7.35 (d, *J* = 9.0 Hz, 1H), 7.31 – 7.26 (m, 2H), 6.57 – 6.51 (m, 1H), 6.49 – 6.45 (m, 1H), 3.80 (s, 3H), 3.75 (s, 3H), 2.74 – 2.61 (m, 4H), 1.79 – 1.67 (m, 1H), 1.36 – 1.24 (m, 1H), 1.11 – 0.96 (m, 6H) ppm; *δ* (for minor diastereomer) 7.90 – 7.83 (m, 1H), 7.43 – 7.37 (m, 1H), 7.35 (d, *J* = 9.0 Hz, 1H), 7.31 – 7.26 (m, 2H), 6.57 – 6.51 (m, 1H), 6.49 – 6.45 (m, 1H), 3.80 (s, 3H), 3.74 (s, 3H), 2.74 – 2.61 (m, 4H), 1.79 – 1.67 (m, 1H), 1.36 – 1.24 (m, 1H), 1.11 – 0.96 (m, 6H) ppm; **^{13}C NMR** (101 MHz, $CDCl_3$) δ (for major diastereomer) 179.3, 161.0, 161.0, 159.1, 139.4, 131.7, 131.6, 130.0, 129.1, 126.0, 125.9, 118.3, 105.2, 99.9, 76.2, 55.9, 55.5, 38.9, 23.6, 21.9, 13.7, 12.6 ppm; δ (for minor diastereomer) 179.3, 161.0, 160.9, 159.2, 139,4, 131.7, 131.6, 130.0, 129.4, 126.0, 125.8, 118.2, 105.1, 99.9, 76.0, 55.9, 55.5, 38.6, 24.1, 22.0, 13.2, 12.3 ppm. **HRMS (ESI)** Calculated for $C_{22}H_{26}NO_4^+$ ($[M+H]^+$) 368.1856. Found 368.1849.

4-butyl-4-(2,4-dimethoxyphenyl)-2-(*o*-tolyl)oxazol-5(4*H*)-one (4h)

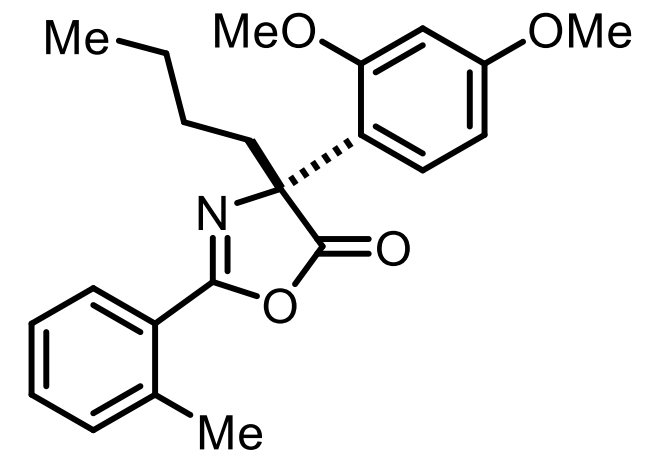

***4h** was prepared following General Procedure E from azlactone **1h** (46.2 mg, 0.2 mmol) and 1,3-dimethoxybenzene (**2a**, 79 μL, 0.6 mmol) with electrocatalyst **3a** (3.5 mg, 0.01 mmol), [collidinium·BF_4] (83.6 mg, 0.4 mmol) and collidine (13 μL, 0.1 mmol) in MeCN (8 mL). The product was isolated by chromatography on silica gel (hexanes/ethyl acetate = 9:1).*

77% yield (57 mg, 0.15 mmol), white solid. **^{1}H NMR** (400 MHz, $CDCl_3$) δ 7.91 – 7.85 (m, 1H), 7.50 – 7.37 (m, 2H), 7.34 – 7.27 (m, 2H), 6.55 (dd, *J* = 8.7, 2.5 Hz, 1H), 6.44 (d, *J* = 2.5 Hz, 1H), 3.80 (s, 3H), 3.71 (s, 3H), 2.62 (s, 3H), 2.42 (td, *J* = 12.5, 4.2 Hz, 1H), 2.24 (ddd, *J* = 12.8, 11.6, 4.6 Hz, 1H), 1.45 – 1.27 (m, 4H), 0.93 (t, *J* = 7.2 Hz, 3H) ppm; **^{13}C NMR** (101 MHz, $CDCl_3$) δ 180.7, 161.6, 161.4, 158.6, 139.3, 131.7, 131.6, 129.9, 128.3, 126.0, 126.0, 119.1, 105.0, 99.4, 71.8, 55.7, 55.5, 35.0, 25.2, 22.9, 21.8, 14.1 ppm. **HRMS (ESI)** Calculated for $C_{22}H_{26}NO_4^+$ ($[M+H]^+$) 368.1856. Found 368.1852.

4-benzyl-4-(2,4-dimethoxyphenyl)-2-(*o*-tolyl)oxazol-5(4*H*)-one (4i)

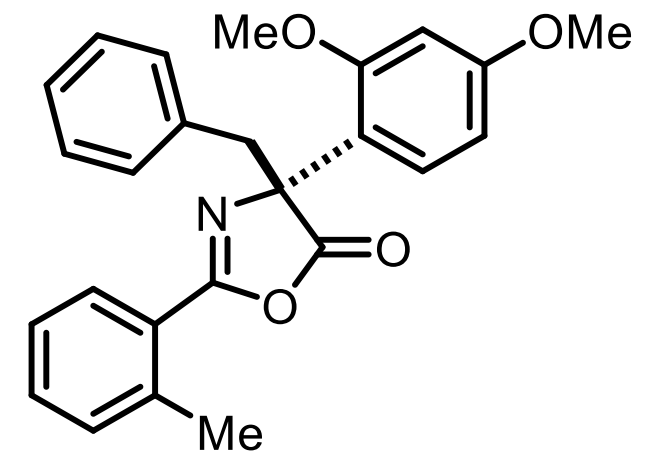

***4i** was prepared following General Procedure E from azlactone **1i** (53.0 mg, 0.2 mmol) and 1,3-dimethoxybenzene (**2a**, 79 µL, 0.6 mmol) with electrocatalyst **3a** (3.5 mg, 0.01 mmol), [collidinium·BF_4] (83.6 mg, 0.4 mmol) and collidine (13 µL, 0.1 mmol) in MeCN (8 mL). The product was isolated by chromatography on silica gel (hexanes/ethyl acetate = 5:1).*

62% yield (50 mg, 0.12 mmol), white solid. **^{1}H NMR** (400 MHz, $CDCl_3$) δ 7.58 (d, *J* = 8.7 Hz, 1H), 7.50 (dd, *J* = 8.1, 1.5 Hz, 1H), 7.32 (dd, *J* = 7.5, 1.5 Hz, 1H), 7.29 – 7.12 (m, 7H), 6.60 (dd, *J* = 8.7, 2.5 Hz, 1H), 6.47 (d, *J* = 2.5 Hz, 1H), 3.82 (m, 4H), 3.68 (s, 3H), 3.52 (d, *J* = 12.5 Hz, 1H), 2.28 (s, 3H) ppm; **^{13}C NMR** (101 MHz, $CDCl_3$) δ 179.9, 161.9, 161.6, 158.7, 138.9, 133.8, 131.4, 131.3, 131.2, 129.8, 128.2, 128.1, 127.5, 126.0, 125.8, 118.9, 105.1, 99.6, 73.0, 55.8, 55.6, 41.2, 21.0 ppm. **HRMS (ESI)** Calculated for $C_{25}H_{24}NO_4^+$ ($[M+H]^+$) 402.1700. Found 402.1693.

4-(2,4-dimethoxyphenyl)-4-(4-methoxybenzyl)-2-(*o*-tolyl)oxazol-5(4*H*)-one (4j)

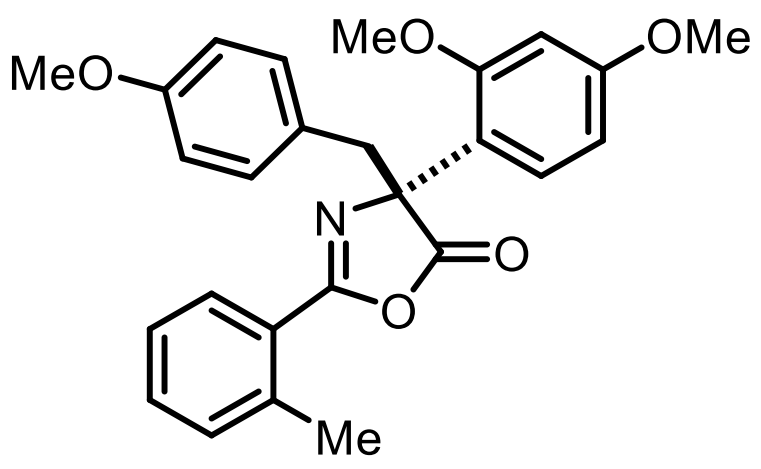

***4j** was prepared following General Procedure E from azlactone **1j** (59.0 mg, 0.2 mmol) and 1,3-dimethoxybenzene (**2a**, 79 µL, 0.6 mmol) with electrocatalyst **3a** (3.5 mg, 0.01 mmol), [collidinium·BF_4] (83.6 mg, 0.4 mmol) and collidine (13 µL, 0.1 mmol) in MeCN (8 mL). The product was isolated by chromatography on silica gel (hexanes/ethyl acetate = 4:1).*

48% yield (41 mg, 0.10 mmol), white solid. **^{1}H NMR** (400 MHz, $CDCl_3$) δ 7.57 (d, *J* = 8.7 Hz, 1H), 7.54 (dd, *J* = 8.0, 1.5 Hz, 1H), 7.34 (td, *J* = 7.6, 1.5 Hz, 1H), 7.22 – 7.13 (m, 4H), 6.78 – 6.73 (m, 2H), 6.60 (dd, *J* = 8.7, 2.5 Hz, 1H), 6.47 (d, *J* = 2.5 Hz, 1H), 3.83 (s, 3H), 3.74 (m, 4H), 3.69 (s, 3H), 3.47 (d, *J* = 12.7 Hz, 1H), 2.31 (s, 3H) ppm; **^{13}C NMR** (101 MHz, $CDCl_3$) δ 180.0, 161.8, 161.5, 159.0, 158.7, 138.9, 132.2, 131.4, 131.3, 129.8, 128.1, 126.1, 125.8, 125.7, 119.0, 113.6, 105.1, 99.6, 73.0, 55.7, 55.6, 55.3, 40.3, 21.1 ppm. **HRMS (ESI)** Calculated for $C_{26}H_{26}NO_5^+$ ($[M+H]^+$) 432.1805. Found 432.1800.

***tert*-butyl 3-((4-(2,4-dimethoxyphenyl)-5-oxo-2-(*o*-tolyl)-4,5-dihydrooxazol-4-yl)methyl)-1*H*-indole-1-carboxylate (4k)**

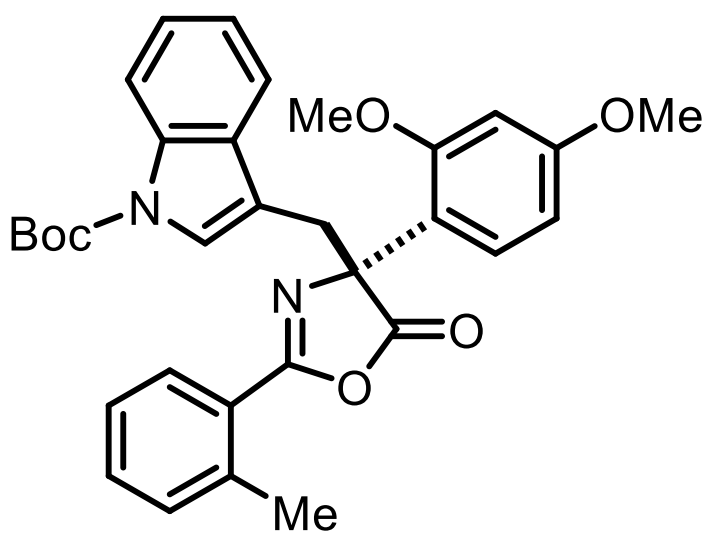

***4k** was prepared following General Procedure E from azlactone **1k** (80.9 mg, 0.2 mmol) and 1,3-dimethoxybenzene (**2a**, 79 µL, 0.6 mmol) with electrocatalyst **3a** (3.5 mg, 0.01 mmol), [collidinium·BF_4] (83.6 mg, 0.4 mmol) and collidine (13 µL, 0.1 mmol) in MeCN (8 mL). The product was isolated by chromatography on silica gel (hexanes/ethyl acetate = 9:1 to 4:1).*

62% yield (67 mg, 0.12 mmol), yellow solid. **^{1}H NMR** (400 MHz, $CDCl_3$) δ 8.09 (d, *J* = 8.3 Hz, 1H), 7.67 – 7.60 (m, 2H), 7.52 – 7.45 (m, 2H), 7.33 – 7.22 (m, 2H), 7.20 – 7.09 (m, 3H), 6.62 (dd, *J* = 8.7, 2.5 Hz, 1H), 6.50 (d, *J* = 2.5 Hz, 1H), 3.90 (d, *J* = 13.6 Hz, 1H), 3.84 (s, 3H), 3.71 (s, 3H), 3.66 (d, *J* = 13.6 Hz, 1H), 2.31 (s, 3H), 1.57 (s, 9H) ppm; **^{13}C NMR** (101 MHz, $CDCl_3$) δ 180.0, 162.3, 161.6, 158.7, 149.5, 138.9, 135.3, 131.4, 131.3, 130.7, 129.8, 128.2, 125.9, 125.9, 125.8, 124.5, 122.6, 120.0, 118.8, 115.0, 113.0, 105.1, 99.7, 83.6, 72.6, 55.8, 55.6, 30.8, 28.2, 21.2 ppm. **HRMS (ESI)** Calculated for $C_{32}H_{33}N_2O_6^+$ ($[M+H]^+$) 541.2333. Found 541.2324.

4-(2,4-dimethoxyphenyl)-4-(2-(methylthio)ethyl)-2-(*o*-tolyl)oxazol-5(4*H*)-one (4l)

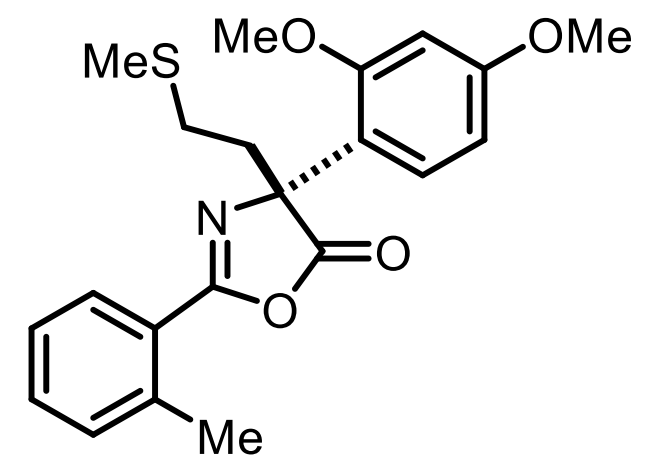

***4l** was prepared following General Procedure E from azlactone **1l** (49.8 mg, 0.2 mmol) and 1,3-dimethoxybenzene (**2a**, 79 μL, 0.6 mmol) with electrocatalyst **3a** (3.5 mg, 0.01 mmol), [collidinium·BF$_4$] (83.6 mg, 0.4 mmol) and collidine (13 μL, 0.1 mmol) in MeCN (8 mL). The product was isolated by chromatography on silica gel (hexanes/ethyl acetate = 5:1).*

62% yield (48 mg, 0.12 mmol), colourless oil. **^{1}H NMR** (400 MHz, CDCl$_3$) δ 7.91 – 7.85 (m, 1H), 7.47 – 7.39 (m, 2H), 7.34 – 7.27 (m, 2H), 6.56 (dd, *J* = 8.7, 2.5 Hz, 1H), 6.45 (d, *J* = 2.5 Hz, 1H), 3.80 (s, 3H), 3.71 (s, 3H), 2.74 – 2.44 (m, 7H), 2.14 (s, 3H) ppm; **^{13}C NMR** (101 MHz, CDCl$_3$) δ 179.9, 161.8, 161.5, 158.6, 139.5, 131.8, 131.8, 129.9, 128.2, 126.1, 125.5, 118.2, 105.1, 99.6, 71.6, 55.8, 55.6, 35.2, 28.0, 21.9, 15.7 ppm. **HRMS (ESI)** Calculated for $C_{21}H_{24}NO_4S^+$ ($[M+H]^+$) 386.1421. Found 386.1418.

4-((*tert*-butylthio)methyl)-4-(2,4-dimethoxyphenyl)-2-(*o*-tolyl)oxazol-5(4*H*)-one (4m)

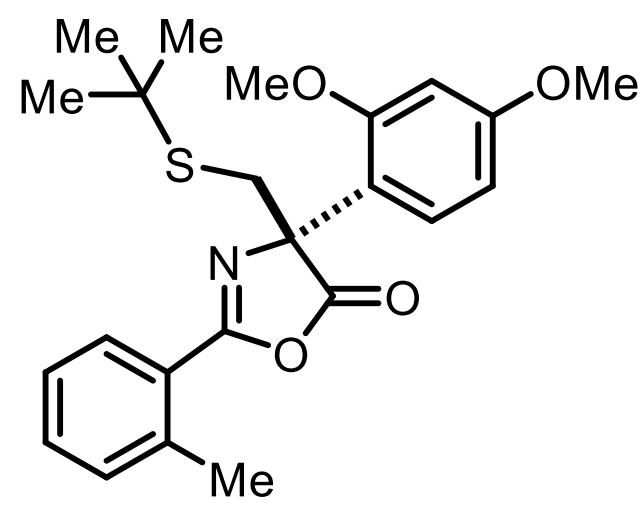

***4m** was prepared following General Procedure E from azlactone **1m** (55.5 mg, 0.2 mmol) and 1,3-dimethoxybenzene (**2a**, 79 μL, 0.6 mmol) with electrocatalyst **3a** (3.5 mg, 0.01 mmol), [collidinium·BF$_4$] (83.6 mg, 0.4 mmol) and collidine (13 μL, 0.1 mmol) in MeCN (8 mL). The product was isolated by chromatography on silica gel (hexanes/ethyl acetate = 9:1).*

77% yield (64 mg, 0.15 mmol), yellow oil. **^{1}H NMR** (400 MHz, CDCl$_3$) δ 7.91 – 7.86 (m, 1H), 7.46 (d, *J* = 8.7 Hz, 1H), 7.40 (td, *J* = 7.5, 1.5 Hz, 1H), 7.31 – 7.26 (m, 2H), 6.55 (dd, *J* = 8.7, 2.5 Hz, 1H), 6.45 (d, *J* = 2.5 Hz, 1H), 3.80 (s, 3H), 3.76 – 3.68 (m, 4H), 3.29 (d, *J* = 11.8 Hz, 1H), 2.64 (s, 3H), 1.33 (s, 9H) ppm; **^{13}C NMR** (101 MHz, CDCl$_3$) δ 179.2, 162.5, 161.6, 158.6, 139.6, 131.6, 131.6, 130.1, 128.1, 125.9, 118.5, 105.0, 99.7, 77.5, 77.2, 76.8, 72.4, 55.8, 55.6, 43.0, 33.3, 31.0, 21.9 ppm. **HRMS (ESI)** Calculated for $C_{23}H_{28}NO_4S^+$ ($[M+H]^+$) 414.1734. Found 414.1729.

4-(2,4-dimethoxyphenyl)-4-phenyl-2-(*o*-tolyl)oxazol-5(4*H*)-one (4n)

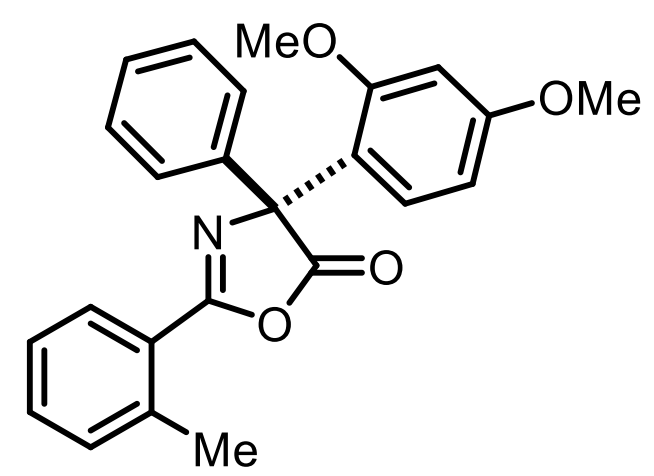

***4n** was prepared following General Procedure E from azlactone **1n** (50.2 mg, 0.2 mmol) and 1,3-dimethoxybenzene (**2a**, 79 μL, 0.6 mmol) with electrocatalyst **3a** (3.5 mg, 0.01 mmol), [collidinium·BF$_4$] (83.6 mg, 0.4 mmol) and collidine (13 μL, 0.1 mmol) in MeCN (8 mL). The product was isolated by chromatography on silica gel (hexanes/ethyl acetate = 9:1 to 4:1).*

79% yield (61 mg, 0.16 mmol), white solid. **^{1}H NMR** (400 MHz, CDCl$_3$) δ 7.98 – 7.92 (m, 1H), 7.85 – 7.80 (m, 2H), 7.48 – 7.38 (m, 4H), 7.34 – 7.29 (m, 2H), 6.61 (d, *J* = 8.7 Hz, 1H), 6.47 (d, *J* = 2.5 Hz, 1H), 6.37 (dd, *J* = 8.7, 2.5 Hz, 1H), 3.79 (s, 3H), 3.77 (s, 3H), 2.72 (s, 3H) ppm; **^{13}C NMR** (101 MHz, CDCl$_3$) δ 179.2, 161.5, 161.2, 158.5, 139.7, 137.7, 131.8, 131.7, 131.3, 130.0, 128.6, 128.6, 127.9, 126.0, 125.7, 121.6, 104.9, 99.3, 74.5, 55.9, 55.5, 22.1 ppm. **HRMS (ESI)** Calculated for $C_{24}H_{21}NNaO_4^+$ ($[M+Na]^+$) 410.1363. Found 410.1382.

4-cyclopropyl-4-(2,4-dimethoxyphenyl)-2-(*o*-tolyl)oxazol-5(4*H*)-one (4o)

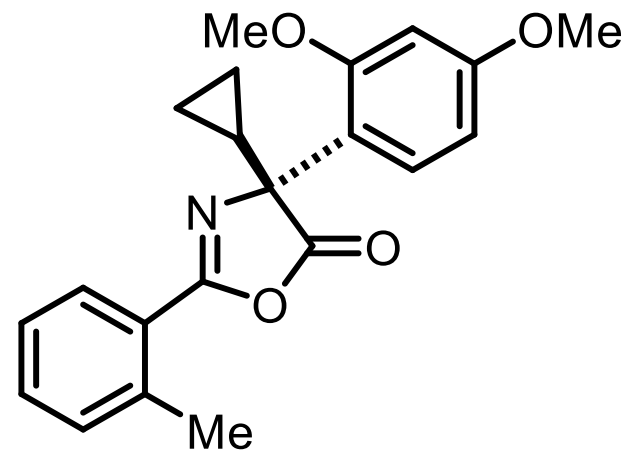

***4o** was prepared following General Procedure E from azlactone **1o** (43.1 mg, 0.2 mmol) and 1,3-dimethoxybenzene (**2a**, 79 μL, 0.6 mmol) with electrocatalyst **3a** (3.5 mg, 0.01 mmol), [collidinium·BF_4] (83.6 mg, 0.4 mmol) and collidine (13 μL, 0.1 mmol) in MeCN (8 mL). The product was isolated by chromatography on silica gel (hexanes/ethyl acetate = 9:1).*

54% yield (38 mg, 0.11 mmol), white foam. **^{1}H NMR** (400 MHz, $CDCl_3$) δ 7.90 (d, *J* = 8.6 Hz, 1H), 7.88 – 7.84 (m, 1H), 7.44 – 7.38 (m, 1H), 7.32 – 7.26 (m Hz, 2H), 6.60 (dd, *J* = 8.6, 2.5 Hz, 1H), 6.44 (d, *J* = 2.5 Hz, 1H), 3.81 (s, 3H), 3.70 (s, 3H), 2.57 (s, 3H), 1.74 – 1.63 (m, 1H), 0.94 – 0.83 (m, 2H), 0.60 – 0.51 (m, 1H), 0.37 – 0.28 (m, 1H) ppm; **^{13}C NMR** (101 MHz, $CDCl_3$) δ 180.5, 161.9, 161.5, 158.4, 139.5, 131.7, 131.7, 129.9, 129.6, 126.0, 125.7, 119.4, 105.1, 99.3, 70.8, 55.8, 55.6, 21.8, 16.2, 2.6, -0.5 ppm. **HRMS (ESI)** Calculated for $C_{21}H_{22}NO_4^+$ ($[M+H]^+$) 352.1543. Found 352.1544.

4-(2,4-dimethoxyphenyl)-4-methyl-2-phenyloxazol-5(4*H*)-one (4p)

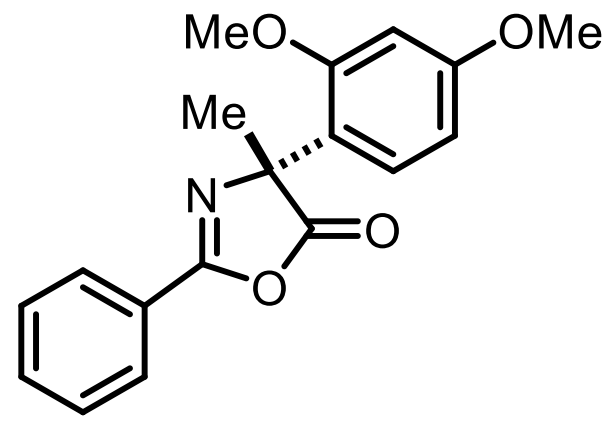

***4p** was prepared following General Procedure E from azlactone **1p** (35.0 mg, 0.2 mmol) and 1,3-dimethoxybenzene (**2a**, 79 μL, 0.6 mmol) with electrocatalyst **3a** (3.5 mg, 0.01 mmol), [collidinium·BF_4] (83.6 mg, 0.4 mmol) and collidine (13 μL, 0.1 mmol) in MeCN (8 mL). The product was isolated by chromatography on silica gel (hexanes/ethyl acetate = 4:1).*

70% yield (44 mg, 0.14 mmol), white solid. **^{1}H NMR** (400 MHz, $CDCl_3$) δ 8.10 – 7.98 (m, 2H), 7.60 – 7.53 (m, 1H), 7.51 – 7.45 (m, 2H), 7.44 (d, *J* = 8.6 Hz, 1H), 6.56 (dd, *J* = 8.6, 2.5 Hz, 1H), 6.42 (d, *J* = 2.5 Hz, 1H), 3.80 (s, 3H), 3.65 (s, 3H), 1.86 (s, 3H).; **^{13}C NMR** (101 MHz, $CDCl_3$) δ 181.1, 161.7, 160.9, 158.5, 132.5, 128.8, 128.1, 128.0, 126.6, 118.6, 105.1, 99.5, 68.1, 55.8, 55.6, 22.8 ppm. **HRMS (ESI)** Calculated for $C_{18}H_{18}NO_4^+$ ($[M+H]^+$) 312.1230. Found 312.1233.

4-(2,4-dimethoxyphenyl)-2-mesityl-4-methyloxazol-5(4*H*)-one (4q)

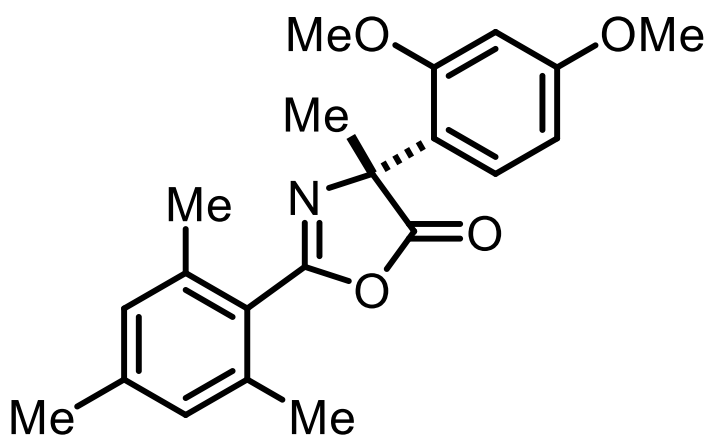

***4q** was prepared following General Procedure E from azlactone **1q** (43.4 mg, 0.2 mmol) and 1,3-dimethoxybenzene (**2a**, 79 μL, 0.6 mmol) with electrocatalyst **3a** (3.5 mg, 0.01 mmol), [collidinium·BF_4] (83.6 mg, 0.4 mmol) and collidine (13 μL, 0.1 mmol) in MeCN (8 mL). The product was isolated by chromatography on silica gel (hexanes/ethyl acetate = 5:1).*

55% yield (39 mg, 0.11 mmol), white solid. **^{1}H NMR** (400 MHz, $CDCl_3$) δ 7.44 (d, *J* = 8.6 Hz, 1H), 6.91 (s, 2H), 6.57 (dd, *J* = 8.6, 2.5 Hz, 1H), 6.49 (d, *J* = 2.5 Hz, 1H), 3.81 (s, 3H), 3.78 (s, 3H), 2.39 (s, 6H), 2.30 (s, 3H), 1.90 (s, 3H) ppm; **^{13}C NMR** (101 MHz, $CDCl_3$) δ 181.4, 161.6, 161.3, 158.2, 140.5, 137.9, 128.8, 128.4, 124.3, 118.2, 105.1, 99.3, 67.8, 55.5, 55.5, 23.5, 21.3, 20.0 ppm. **HRMS (ESI)** Calculated for $C_{21}H_{24}NO_4^+$ ($[M+H]^+$) 354.1700. Found 354.1706.

4-(2,4-dimethoxyphenyl)-4-methyl-2-(*m*-tolyl)oxazol-5(4*H*)-one (4r)

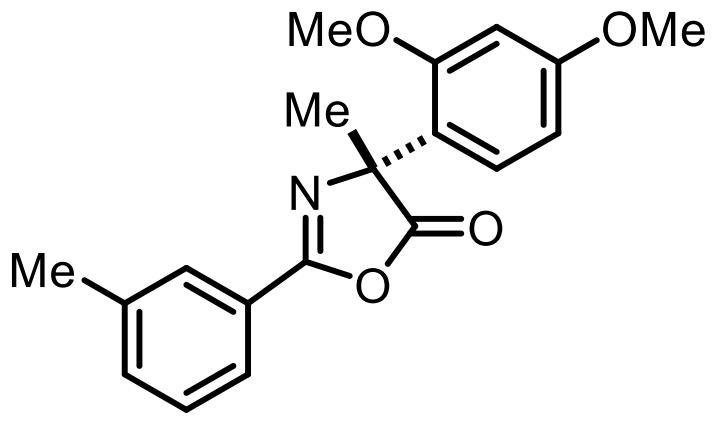

***4r** was prepared following General Procedure E from azlactone **1r** (37.8 mg, 0.2 mmol) and 1,3-dimethoxybenzene (**2a**, 79 µL, 0.6 mmol) with electrocatalyst **3a** (3.5 mg, 0.01 mmol), [collidinium·BF_4] (83.6 mg, 0.4 mmol) and collidine (13 µL, 0.1 mmol) in MeCN (8 mL). The product was isolated by chromatography on silica gel (hexanes/ethyl acetate = 4:1).*

69% yield (45 mg, 0.14 mmol), white solid. **^{1}H NMR** (400 MHz, $CDCl_3$) δ 7.89 – 7.86 (m, 1H), 7.85 – 7.79 (m, 1H), 7.43 (d, *J* = 8.7 Hz, 1H), 7.40 – 7.33 (m, 2H), 6.56 (dd, *J* = 8.6, 2.5 Hz, 1H), 6.42 (d, *J* = 2.5 Hz, 1H), 3.79 (s, 3H), 3.6 (s, 3H), 2.40 (s, 3H), 1.85 (s, 3H) ppm; **^{13}C NMR** (101 MHz, $CDCl_3$) δ 181.1, 161.7, 161.0, 158.5, 138.7, 133.4, 128.7, 128.4, 128.1, 126.4, 125.2, 118.6, 105.1, 99.4, 68.0, 55.8, 55.6, 22.8, 21.4 ppm. **HRMS (ESI)** Calculated for $C_{19}H_{20}NO_4^+$ ($[M+H]^+$) 326.1387. Found 326.1390.

4-(2,4-dimethoxyphenyl)-4-methyl-2-(*p*-tolyl)oxazol-5(4*H*)-one (4s)

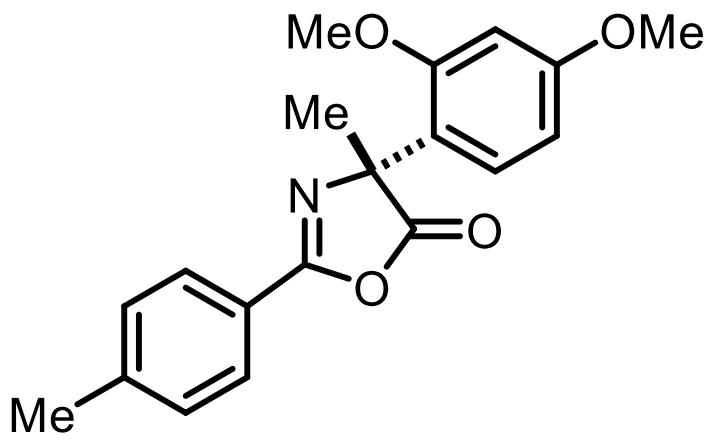

***4s** was prepared following General Procedure E from azlactone **1s** (37.8 mg, 0.2 mmol) and 1,3-dimethoxybenzene (**2a**, 79 µL, 0.6 mmol) with electrocatalyst **3a** (3.5 mg, 0.01 mmol), [collidinium·BF_4] (83.6 mg, 0.4 mmol) and collidine (13 µL, 0.1 mmol) in MeCN (8 mL). The product was isolated by chromatography on silica gel (hexanes/ethyl acetate = 5:1).*

66% yield (43 mg, 0.13 mmol), white solid. **^{1}H NMR** (400 MHz, $CDCl_3$) δ 7.95 – 7.89 (m, 2H), 7.43 (d, *J* = 8.7 Hz, 1H), 7.31 – 7.26 (m, 2H), 6.55 (dd, *J* = 8.7, 2.5 Hz, 1H), 6.42 (d, *J* = 2.5 Hz, 1H), 3.80 (s, 3H), 3.65 (s, 3H), 2.43 (s, 3H), 1.85 (s, 3H) ppm; **^{13}C NMR** 13C NMR (101 MHz, CDCl3) δ 181.3, 161.7, 160.9, 158.5, 143.2, 129.6, 128.1, 128.0, 123.8, 118.8, 105.1, 99.5, 68.1, 55.8, 55.6, 22.9, 21.8 ppm. **HRMS (ESI)** Calculated for $C_{19}H_{20}NO_4^+$ ($[M+H]^+$) 326.1387. Found 326.1392.

4-(2,4-dimethoxyphenyl)-2-(4-methoxyphenyl)-4-methyloxazol-5(4*H*)-one (4t)

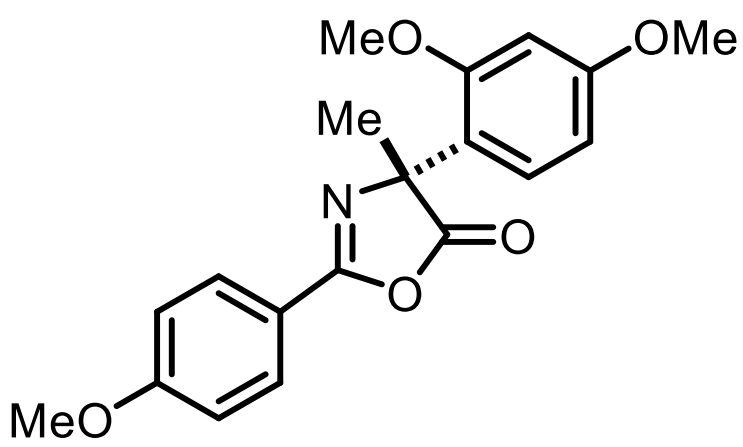

***4t** was prepared following General Procedure E from azlactone **1t** (41.0 mg, 0.2 mmol) and 1,3-dimethoxybenzene (**2a**, 79 µL, 0.6 mmol) with electrocatalyst **3d** (3.4 mg, 0.01 mmol), [collidinium·BF_4] (83.6 mg, 0.4 mmol) and collidine (13 µL, 0.1 mmol) in MeCN (8 mL). The product was isolated by chromatography on silica gel (hexanes/ethyl acetate = 4:1 to 2:1).*

33% yield (23 mg, 0.07 mmol), white solid. **^{1}H NMR** (400 MHz, CDCl3) δ 8.00 – 7.94 (m, 2H), 7.42 (d, J = 8.7 Hz, 1H), 6.99 – 6.95 (m, 2H), 6.55 (dd, J = 8.6, 2.5 Hz, 1H), 6.42 (d, J = 2.5 Hz, 1H), 3.87 (s, 3H), 3.80 (s, 3H), 3.65 (s, 3H), 1.84 (s, 3H) ppm; **^{13}C NMR** (100 MHz, $CDCl_3$) *δ* ^{13}C NMR (101 MHz, $CDCl_3$) δ 181.3, 163.1, 161.7, 160.6, 158.5, 129.8, 128.1, 118.9, 118.9, 114.2, 105.1, 99.5, 68.0, 55.8, 55.6, 55.6, 22.9 ppm. **HRMS (ESI)** Calculated for $C_{19}H_{20}NO_5^+$ ($[M+H]^+$) 342.2336. Found 342.1342.

4-(2,4-dimethoxyphenyl)-4-methyl-2-(4-(trifluoromethyl)phenyl)oxazol-5(4*H*)-one (4u)

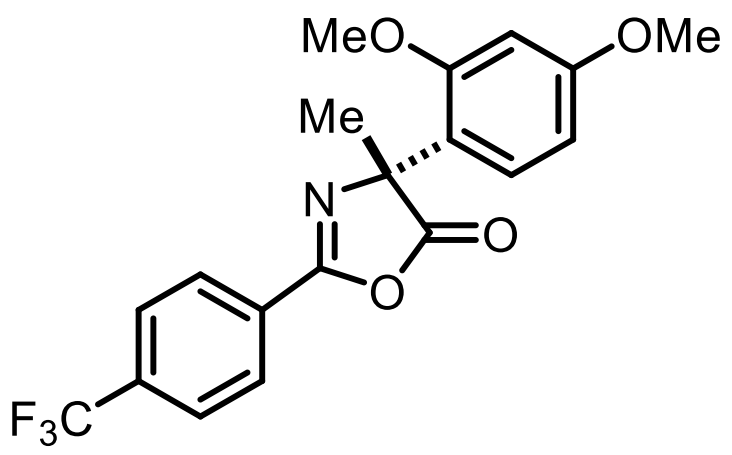

4u *was prepared following General Procedure E from azlactone* ***1u*** *(48.6 mg, 0.2 mmol) and 1,3-dimethoxybenzene (**2a**, 79 µL, 0.6 mmol) with electrocatalyst **3a** (3.5 mg, 0.01 mmol), [collidinium·BF_4] (83.6 mg, 0.4 mmol) and collidine (13 µL, 0.1 mmol) in MeCN (8 mL). The product was isolated by chromatography on silica gel (hexanes/ethyl acetate = 8:1).*

28% yield (21 mg, 0.06 mmol), white solid. **^{1}H NMR** (400 MHz, $CDCl_3$) δ 8.18 – 8.13 (m, 2H), 7.78 – 7.72 (m, 2H), 7.44 (d, *J* = 8.7 Hz, 1H), 6.58 (dd, *J* = 8.6, 2.5 Hz, 1H), 6.43 (d, *J* = 2.4 Hz, 1H), 3.81 (s, 3H), 3.65 (s, 3H), 1.87 (s, 3H) ppm; **^{13}C NMR** (101 MHz, $CDCl_3$) δ 180.5, 161.9, 159.8, 158.4, 134.1 (q, $^{2}J_{CF}$ = 32.7 Hz), 130.0, 128.4, 128.1, 125.9 (q, $^{3}J_{CF}$ = 3.8 Hz), 123.7 (q, $^{1}J_{CF}$ = 272.8 Hz), 118.1, 105.2, 99.4, 68.3, 55.8, 55.6, 22.7 ppm; **^{19}F NMR** (376 MHz, $CDCl_3$) δ –63.1 (s) ppm. **HRMS (ESI)** Calculated for $C_{19}H_{17}F_3NO_4^+$ ($[M+H]^+$) 380.1104. Found 380.1105.

4-methyl-2-(*o*-tolyl)-4-(2,4,6-trimethoxyphenyl)oxazol-5(4*H*)-one (4v)

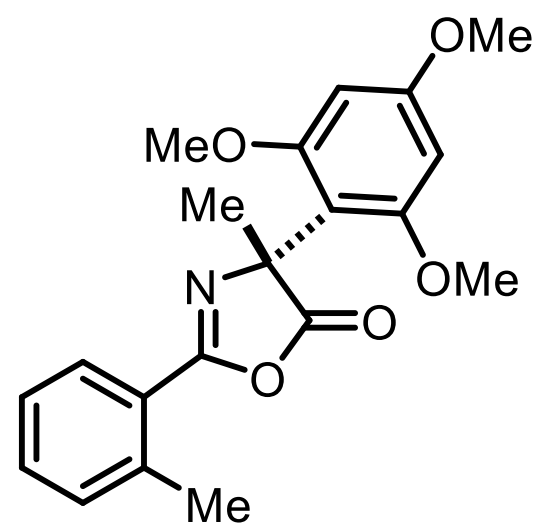

4v *was prepared following General Procedure E from azlactone **1e** (37.8 mg, 0.2 mmol) and 1,3,5-trimethoxybenzene (**2b**, 100.8 mg, 0.6 mmol) with electrocatalyst **3c** (3.4 mg, 0.01 mmol), [collidinium·BF_4] (83.6 mg, 0.4 mmol) and collidine (13 µL, 0.1 mmol) in MeCN (8 mL). The product was isolated by chromatography on silica gel (hexanes/ethyl acetate = 3:1).*

61% yield (43 mg, 0.12 mmol), white solid. **^{1}H NMR** (400 MHz, $CDCl_3$) δ 7.90 – 7.81 (m, 1H), 7.39 (td, *J* = 7.5, 1.5 Hz, 1H), 7.31 – 7.21 (m, 2H), 6.14 (s, 2H), 3.79 (s, 3H), 3.77 (s, 6H), 2.61 (s, 3H), 1.99 (s, 3H) ppm; **^{13}C NMR** (101 MHz, $CDCl_3$) δ 182.2, 161.5, 160.8, 160.5, 139.1, 131.5, 131.4, 129.9, 126.3, 125.9, 106.6, 92.1, 69.1, 55.8, 55.4, 25.3, 21.7 ppm. **HRMS (ESI)** Calculated for $C_{19}H_{22}NO_4^+$ ($[M+H]^+$) 328.1543. Found 328.1542.

4-(6-methoxynaphthalen-2-yl)-4-methyl-2-(*o*-tolyl)oxazol-5(4*H*)-one (4w)

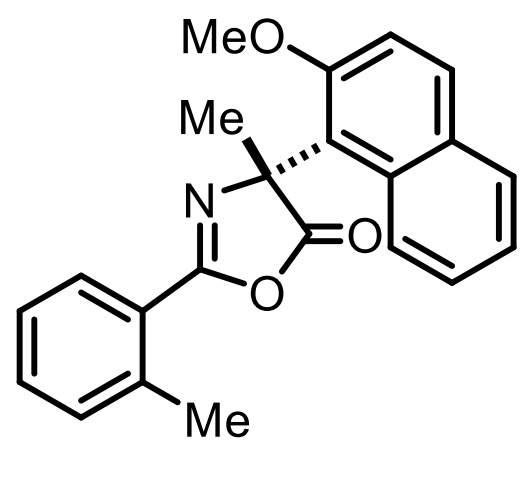

4w *was prepared following General Procedure E from azlactone **1e** (37.8 mg, 0.2 mmol) and 2-methoxybenzene (**2c**, 94.9 mg, 0.6 mmol) with electrocatalyst **3a** (3.5 mg, 0.01 mmol), [collidinium·BF_4] (83.6 mg, 0.4 mmol) and collidine (13 µL, 0.1 mmol) in MeCN (8 mL). The product was isolated by chromatography on silica gel (hexanes/ethyl acetate = 9:1).*

20% yield (14 mg, 0.04 mmol), colourless oil. **^{1}H NMR** (400 MHz, $CDCl_3$) δ 8.63 (dq, *J* = 8.8, 0.9 Hz, 1H), 7.94 (dd, *J* = 7.8, 1.5 Hz, 1H), 7.83 – 7.79 (m, 2H), 7.54 (ddd, *J* = 8.8, 6.7, 1.5 Hz, 1H), 7.43 – 7.36 (m, 2H), 7.32 – 7.25 (m, 2H), 7.23 (d, *J* = 9.0 Hz, 1H), 3.88 (s, 3H), 2.63 (s, 3H), 2.19 (s, 3H) ppm; **^{13}C NMR** (101 MHz, $CDCl_3$) δ 181.4, 161.0, 154.0, 139.8, 132.7, 131.8, 131.7, 131.2, 131.0, 130.1, 129.0, 126.3, 126.0, 125.8, 125.6, 120.8, 115.1, 69.6, 57.1, 26.8, 22.2 ppm. **HRMS (ESI)** Calculated for $C_{22}H_{20}NO_3^+$ ($[M+H]^+$) 346.1438. Found 346.1439.

4-(3,6-dimethoxynaphthalen-2-yl)-4-methyl-2-(*o*-tolyl)oxazol-5(4*H*)-one (4x)

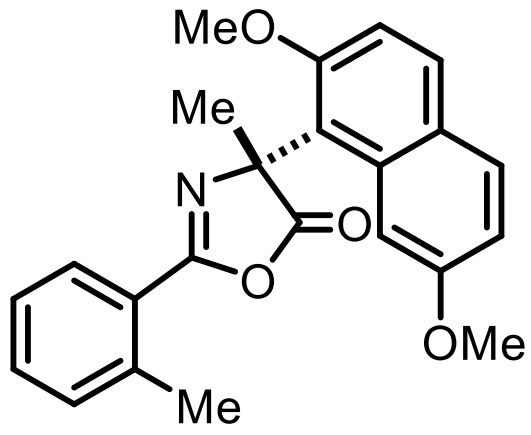

***4x** was prepared following General Procedure E from azlactone **1e** (37.8 mg, 0.2 mmol) and 2,7-dimethoxynaphtalene (**2d**, 112.8 mg, 0.6 mmol) with electrocatalyst **3b** (3.7 mg, 0.01 mmol), [collidinium·BF_4] (83.6 mg, 0.4 mmol) and collidine (13 µL, 0.1 mmol) in MeCN (8 mL). The product was isolated by chromatography on silica gel (hexanes/ethyl acetate = 9:1).*

67% yield (50 mg, 0.13 mmol), colourless oil. **^{1}H NMR** (400 MHz, $CDCl_3$) δ 8.04 – 7.92 (m, 2H), 7.74 – 7.67 (m, 2H), 7.40 (td, *J* = 7.5, 1.5 Hz, 1H), 7.33 – 7.25 (m, 2H), 7.11 – 7.03 (m, 2H), 3.97 (s, 3H), 3.86 (s, 3H), 2.65 (s, 3H), 2.20 (s, 3H) ppm; **^{13}C NMR** (101 MHz, $CDCl_3$) δ 181.2, 160.8, 157.8, 154.5, 139.8, 134.0, 131.8, 131.7, 130.7, 130.4, 130.1, 126.4, 126.0, 125.5, 119.8, 116.3, 112.5, 105.3, 69.6, 57.0, 55.4, 26.3, 22.3 ppm; **HRMS (ESI)** Calculated for $C_{23}H_{22}NO_4^+$ ($[M+H]^+$) 376.1543. Found 376.1550.

4-(2,6-dimethoxypyridin-3-yl)-4-methyl-2-(*o*-tolyl)oxazol-5(4*H*)-one (4y)

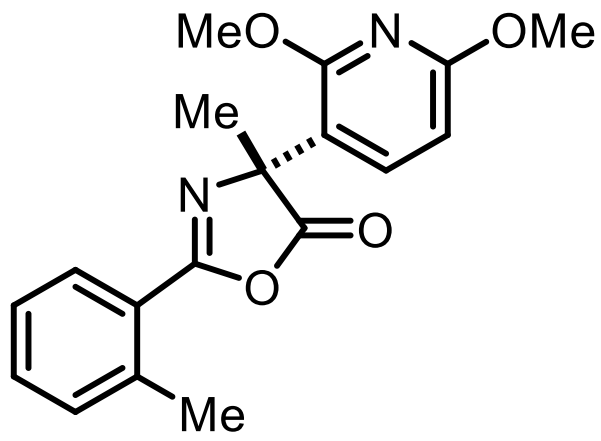

***4y** was prepared following a slightly modified General Procedure E from azlactone **1e** (37.8 mg, 0.2 mmol) and 2,6-dimethoxypyridine (**2e**, 79 µL, 0.6 mmol) with electrocatalyst **3a** (3.5 mg, 0.01 mmol), [collidinium·BF_4] (83.6 mg, 0.4 mmol) and collidine (*26 µL, 0.2 mmol*) in MeCN (*4 mL*). The product was isolated by chromatography on silica gel (hexanes/ethyl acetate = 5:1).*

51% yield (33 mg, 0.10 mmol), white solid. **^{1}H NMR** (400 MHz, $CDCl_3$) δ 7.90 – 7.85 (m, 1H), 7.71 (d, *J* = 8.3 Hz, 1H), 7.45 – 7.39 (m, 1H), 7.33 – 7.27 (m, 2H), 6.39 (d, *J* = 8.3 Hz, 1H), 3.90 (s, 3H), 3.86 (s, 3H), 2.61 (s, 3H), 1.85 (s, 3H) ppm; **^{13}C NMR** (101 MHz, $CDCl_3$) δ 180.6, 163.5, 161.6, 160.1, 139.3, 138.8, 131.7, 131.7, 129.9, 126.0, 125.7, 111.2, 101.4, 67.8, 53.8, 53.7, 22.6, 21.7 ppm. **HRMS (ESI)** Calculated for $C_{18}H_{19}N_2O_4^+$ ($[M+H]^+$) 327.1339. Found 327.1339.

***tert*-butyl 2-(4-methyl-5-oxo-2-(*o*-tolyl)-4,5-dihydrooxazol-4-yl)-1*H*-pyrrole-1-carboxylate (4z)**

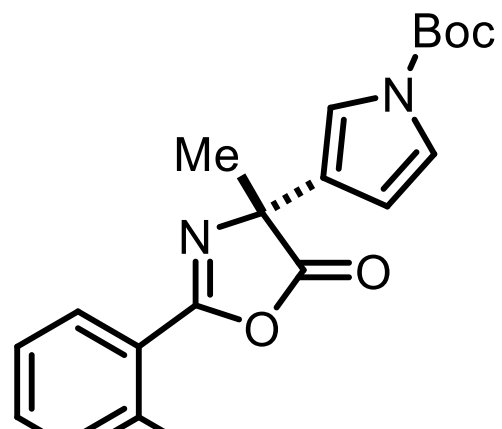

***4z** was prepared following General Procedure E from azlactone **1e** (37.8 mg, 0.2 mmol) and N-Bocpyrrole (**2f**, 100.3 mg, 0.6 mmol) with electrocatalyst **3a** (3.5 mg, 0.01 mmol), [collidinium·BF_4] (83.6 mg, 0.4 mmol) and collidine (13 µL, 0.1 mmol) in MeCN (8 mL). The product was isolated by chromatography on silica gel (hexanes/ethyl acetate = 9:1).*

52% yield (37 mg, 0.10 mmol), yellow oil. **^{1}H NMR** (400 MHz, $CDCl_3$) δ 7.89 – 7.83 (m, 1H), 7.40 (td, *J* = 7.5, 1.5 Hz, 1H), 7.31 – 7.24 (m, 2H), 7.23 (dd, *J* = 3.5, 1.8 Hz, 1H), 6.57 (dd, *J* = 3.5, 1.8 Hz, 1H), 6.19 (t, *J* = 3.5 Hz, 1H), 2.62 (s, 3H), 1.92 (s, 3H), 1.45 (s, 9H) ppm; **^{13}C NMR** (101 MHz, $CDCl_3$) δ 178.7, 163.0, 149.2, 139.2, 131.6, 131.5, 130.0, 129.9, 126.2, 125.9, 123.6, 115.9, 110.4, 84.5, 67.0, 27.9, 25.7, 21.5 ppm. **HRMS (ESI)** Calculated for $C_{20}H_{23}N_2O_4^+$ ($[M+H]^+$) 355.1652. Found 355.1652.

4-(2-acetyl-1-methyl-1*H*-pyrrol-3-yl)-4-methyl-2-(*o*-tolyl)oxazol-5(4*H*)-one (4aaα)

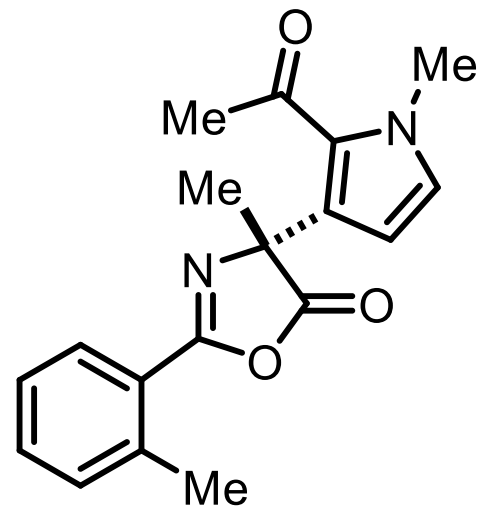

4aaα *was prepared following a slightly modified General Procedure E from azlactone* ***1e*** *(37.8 mg, 0.2 mmol) and 2-acetly-1-methylpyrrole (***2g**, 237 μL, 2.0 mmol*) with electrocatalyst* ***3b*** *(3.7 mg, 0.01 mmol), [collidinium·BF$_4$] (83.6 mg, 0.4 mmol) and collidine (13 μL, 0.1 mmol) in MeCN (8 mL). The product was isolated by chromatography on silica gel (hexanes/ethyl acetate = 9:1 to 4:1).*

63% global yield (39 mg, 0.13 mmol) of 3:1 regiomeric mixture. Pure samples of each regioisomer were obtained upon careful chromatographic purification.

minor regioisomer, yellow oil. **^{1}H NMR** (400 MHz, $CDCl_3$) δ 7.94 – 7.89 (m, 1H), 7.45 (td, *J* = 7.5, 1.4 Hz, 1H), 7.35 – 7.29 (m, 2H), 6.92 (d, *J* = 4.3 Hz, 1H), 6.33 (d, *J* = 4.3 Hz, 1H), 4.11 (s, 3H), 2.68 (s, 3H), 2.44 (s, 3H), 1.91 (s, 3H).; **^{13}C NMR** (101 MHz, CDCl3) δ 189.2, 177.6, 161.2, 140.3, 136.0, 133.2, 132.5, 132.2, 130.3, 126.3, 124.2, 118.9, 108.1, 68.4, 34.7, 27.8, 25.5, 22.6. **HRMS (ESI)** Calculated for $C_{18}H_{19}N_2O_3^+$ ([M+H]$^+$) 311.1390. Found 311.1391.

4-(5-acetyl-1-methyl-1*H*-pyrrol-3-yl)-4-methyl-2-(*o*-tolyl)oxazol-5(4*H*)-one (4aaβ)

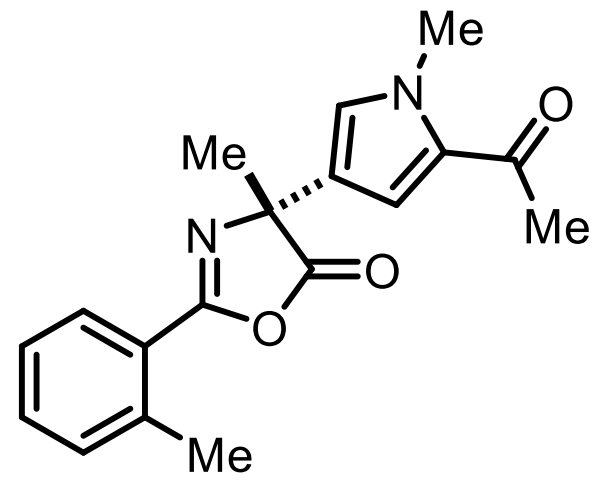

4aaβ *was prepared following a slightly modified General Procedure E from azlactone* ***1e*** *(37.8 mg, 0.2 mmol) and 2-acetly-1-methylpyrrole (***2g**, 237 μL, 2.0 mmol*) with electrocatalyst* ***3b*** *(3.7 mg, 0.01 mmol), [collidinium·BF$_4$] (83.6 mg, 0.4 mmol) and collidine (13 μL, 0.1 mmol) in MeCN (8 mL). The product was isolated by chromatography on silica gel (hexanes/ethyl acetate = 9:1 to 4:1).*

63% global yield (39 mg, 0.13 mmol) of 3:1 regiomeric mixture. Pure samples of each regioisomer were obtained upon careful chromatographic purification.

major regioisomer, yellow oil. **^{1}H NMR** (400 MHz, CDCl3) δ 7.97 – 7.86 (m, 1H), 7.49 – 7.40 (m, 1H), 7.35 – 7.29 (m, 2H), 7.00 (d, J = 2.0 Hz, 1H), 6.88 (d, J = 2.0 Hz, 1H), 3.90 (s, 3H), 2.71 (s, 3H), 2.42 (s, 3H), 1.82 (s, 3H) ppm; **^{13}C NMR** (101 MHz, $CDCl_3$) δ 188.9, 179.6, 160.8, 139.9, 132.1, 132.0, 131.3, 130.2, 127.8, 126.2, 124.8, 121.6, 116.5, 67.7, 37.9, 27.3, 26.6, 22.4 ppm. **HRMS (ESI)** Calculated for $C_{18}H_{19}N_2O_3^+$ ([M+H]$^+$) 311.1390. Found 311.1394.

methyl 1-methyl-3-(4-methyl-5-oxo-2-(*o*-tolyl)-4,5-dihydrooxazol-4-yl)-1*H*-pyrrole-2-carboxylate (4abα)

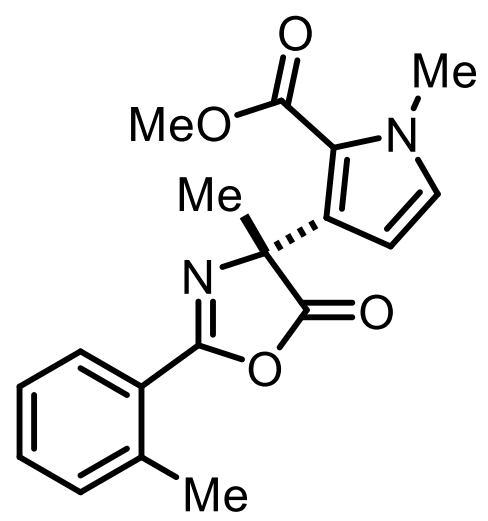

4abα *was prepared following a slightly modified General Procedure E from azlactone* ***1e*** *(37.8 mg, 0.2 mmol) and methyl 1-methylpyrrole-2-carboxylate (***2h**, 278 mg, 2.0 mmol*) with electrocatalyst* ***3b*** *(3.7 mg, 0.01 mmol), [collidinium·BF$_4$] (83.6 mg, 0.4 mmol) and collidine (13 μL, 0.1 mmol) in MeCN (8 mL). The product was isolated by chromatography on silica gel (hexanes/ethyl acetate = 9:1 to 4:1).*

54% global yield (35 mg, 0.11 mmol) of 1.5:1 regiomeric mixture. Pure samples of each regioisomer were obtained upon careful chromatographic purification.

minor regioisomer, yellowish oil. **^{1}H NMR** (400 MHz, $CDCl_3$) δ 7.94 – 7.89 (m, 1H), 7.45 (td, *J* = 7.5, 1.4 Hz, 1H), 7.35 – 7.29 (m, 2H), 6.91 (d, *J* = 4.2 Hz, 1H), 6.32 (d, *J* = 4.2 Hz, 1H), 4.10 (s, 3H), 3.80 (s, 3H), 2.68 (s, 3H), 1.91 (s, 3H) ppm; **^{13}C NMR** (101 MHz, $CDCl_3$) δ 177.6, 161.8, 161.1, 140.3, 134.7, 132.4, 132.2, 130.3, 126.3, 125.0, 124.2, 117.0, 108.1, 68.4, 51.3, 34.0, 25.6, 22.6 ppm. **HRMS (ESI)** Calculated for $C_{18}H_{19}N_2O_4^+$ ([M+H]$^+$) 327.1339. Found 327.1342.

methyl 1-methyl-4-(4-methyl-5-oxo-2-(*o*-tolyl)-4,5-dihydrooxazol-4-yl)-1*H*-pyrrole-2-carboxylate (4abβ)

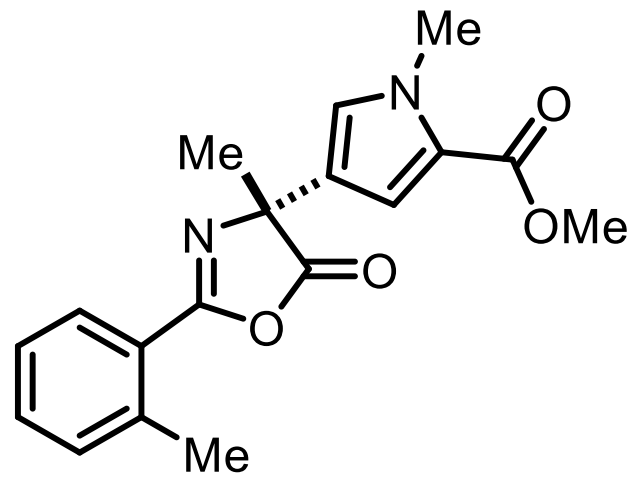

4abβ *was prepared following a slightly modified General Procedure E from azlactone* ***1e*** *(37.8 mg, 0.2 mmol) and methyl 1-methylpyrrole-2-carboxylate (***2h**, 278 mg, 2.0 mmol*) with electrocatalyst* ***3b*** *(3.7 mg, 0.01 mmol), [collidinium·BF*$_4$*] (83.6 mg, 0.4 mmol) and collidine (13 µL, 0.1 mmol) in MeCN (8 mL). The product was isolated by chromatography on silica gel (hexanes/ethyl acetate = 9:1 to 4:1).*

54% global yield (35 mg, 0.11 mmol) of 1.5:1 regiomeric mixture. Pure samples of each regioisomer were obtained upon careful chromatographic purification.

major regioisomer, yellowish oil. **^{1}H NMR** (400 MHz, $CDCl_3$) δ 7.95 – 7.85 (m, 1H), 7.49 – 7.39 (m, 1H), 7.35 – 7.28 (m, 2H), 7.02 (d, *J* = 2.1 Hz, 1H), 6.86 (d, *J* = 2.0 Hz, 1H), 3.90 (s, 3H), 3.80 (s, 3H), 2.71 (s, 3H), 1.81 (s, 3H) ppm; **^{13}C NMR** (101 MHz, $CDCl_3$) δ 179.6, 161.6, 160.7, 140.0, 132.1, 132.0, 130.2, 126.4, 126.1, 124.9, 123.0, 121.7, 115.0, 67.8, 51.3, 37.1, 26.3, 22.5 ppm. **HRMS (ESI)** Calculated for $C_{18}H_{19}N_2O_4^+$ ($[M+H]^+$) 327.1339. Found 327.1346.

ethyl 1-methyl-3-(4-methyl-5-oxo-2-(*o*-tolyl)-4,5-dihydrooxazol-4-yl)-1*H*-indole-2-carboxylate (4ac)

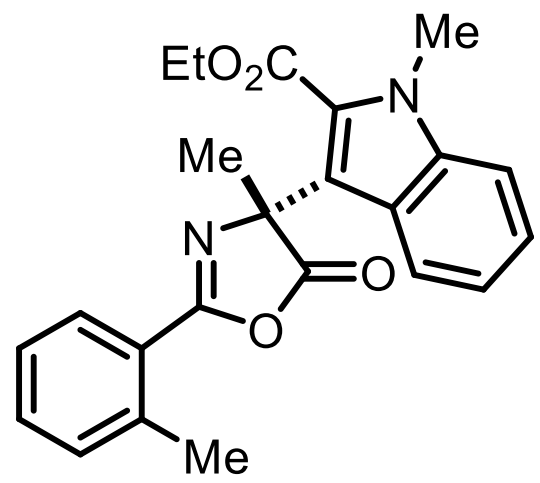

4ac *was prepared following a slightly modified General Procedure E from azlactone* ***1e*** *(37.8 mg, 0.2 mmol) and ethyl 1-methyl-1*H*-indole-2-carboxylate (***2i**, 204 mg, 1.0 mmol*) with electrocatalyst* ***3c*** *(3.4 mg, 0.01 mmol), [collidinium·BF*$_4$*] (83.6 mg, 0.4 mmol) and collidine (13 µL, 0.1 mmol) in MeCN (8 mL). The product was isolated by chromatography on silica gel (hexanes/ethyl acetate = 9:1 to 8:2).*

57% yield (44 mg, 0.11 mmol), colourless oil. **^{1}H NMR** (400 MHz, $CDCl_3$) δ 8.17 (dt, *J* = 8.3, 0.9 Hz, 1H), 7.94 (dd, *J* = 8.1, 1.5 Hz, 1H), 7.42 (td, *J* = 7.4, 1.5 Hz, 1H), 7.40 – 7.33 (m, 2H), 7.33 – 7.27 (m, 2H), 7.21 (ddd, *J* = 8.3, 6.3, 1.8 Hz, 1H), 4.36 – 4.14 (m, 2H), 3.83 (s, 3H), 2.67 (s, 3H), 2.18 (s, 3H), 1.25 (t, *J* = 7.1 Hz, 3H) ppm; **^{13}C NMR** (126 MHz, $CDCl_3$) δ 179.6, 163.1, 160.8, 139.9, 137.8, 131.9, 130.1, 128.5, 126.1, 125.3, 125.2, 124.3, 122.8, 120.9, 113.9, 110.5, 69.1, 61.8, 31.6, 27.1, 22.3, 14.0 ppm. **HRMS (ESI)** Calculated for $C_{23}H_{23}N_2O_4^+$ ($[M+H]^+$) 391.1652. Found 391.1651.

4-methyl-4-(9-methyl-9*H*-carbazol-3-yl)-2-(*o*-tolyl)oxazol-5(4*H*)-one (4ad)

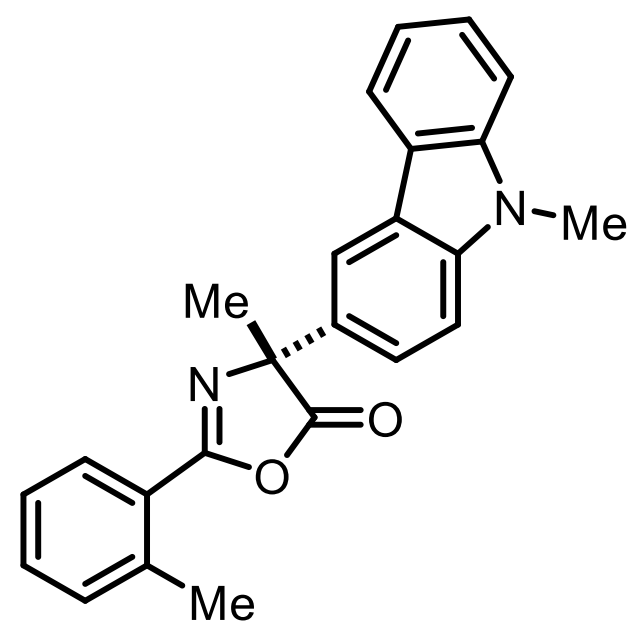

4ad** was prepared following a slightly modified General Procedure E from azlactone **1e** (37.8 mg, 0.2 mmol) and 9-methyl-9*H*-*carbazole (2j**, 362 mg, 2 mmol*) with electrocatalyst **3d** (3.4 mg, 0.01 mmol), [collidinium·BF_4] (83.6 mg, 0.4 mmol) and collidine (13 µL, 0.1 mmol) in MeCN (8 mL). The product was isolated by chromatography on silica gel (hexanes/CH_2Cl_2 = 1:1).*

61% yield (45 mg, 0.12 mmol), white solid. **^{1}H NMR** (400 MHz, $CDCl_3$) δ 8.38 (dd, *J* = 1.9, 0.6 Hz, 1H), 8.12 (dt, *J* = 7.8, 1.0 Hz, 1H), 7.98 (dd, *J* = 7.8, 1.4 Hz, 1H), 7.79 (dd, *J* = 8.6, 1.9 Hz, 1H), 7.52 – 7.32 (m, 6H), 7.25 (ddd, *J* = 8.0, 7.1, 1.0 Hz, 1H), 3.85 (s, 3H), 2.82 (s, 3H), 2.00 (s, 3H) ppm; **^{13}C NMR** (101 MHz, $CDCl_3$) δ 180.1, 160.3, 141.5, 140.8, 140.0, 132.0, 132.0, 130.2, 129.5, 126.2, 126.1, 125.1, 123.2, 123.0, 122.8, 120.6, 119.2, 117.5, 108.8, 108.7, 71.4, 29.3, 28.0, 22.5 ppm. **HRMS (ESI)** Calculated for $C_{24}H_{21}N_2O_2^+$ ($[M+H]^+$) 369.1598. Found 369.1591.

4-(5-methoxythiophen-2-yl)-4-methyl-2-(*o*-tolyl)oxazol-5(4*H*)-one (4ae)

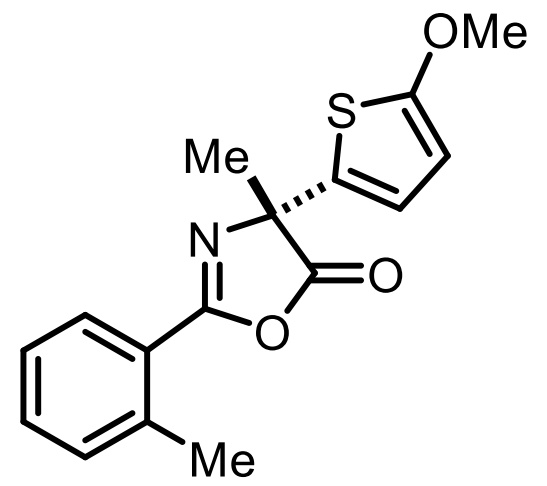

4ae** was prepared following General Procedure E from azlactone **1e** (37.8 mg, 0.2 mmol) and 2-methoxythiophene (2k**, 69 µL, 0.6 mmol*) with electrocatalyst **3c** (3.4 mg, 0.01 mmol), [collidinium·BF_4] (83.6 mg, 0.4 mmol) and collidine (13 µL, 0.1 mmol) in MeCN (8 mL). The product was isolated by chromatography on silica gel (hexanes/ethyl acetate = 9:1).*

46% yield (28 mg, 0.09 mmol), colourless oil. **^{1}H NMR** (500 MHz, $CDCl_3$) δ 7.90 (dd, *J* = 7.8, 1.4 Hz, 1H), 7.45 (td, *J* = 7.5, 1.5 Hz, 1H), 7.35 – 7.29 (m, 2H), 6.80 (d, *J* = 4.0 Hz, 1H), 6.10 (d, *J* = 4.0 Hz, 1H), 3.86 (s, 3H), 2.72 (s, 3H), 1.87 (s, 3H) ppm; **^{13}C NMR** (126 MHz, $CDCl_3$) δ 178.1, 166.6, 161.4, 140.3, 132.3, 132.0, 130.3, 127.2, 126.1, 124.5, 122.1, 103.9, 69.4, 60.4, 26.8, 22.5 ppm. **HRMS (ESI)** Calculated for $C_{16}H_{16}NO_3S^+$ ($[M+H]^+$) 302.0845. Found 302.0845.

4-((8*R*,9*S*,13*S*,14*S*)-3-methoxy-13-methyl-17-oxo-7,8,9,11,12,13,14,15,16,17-decahydro-6*H*-cyclopenta[*a*]phenanthren-2-yl)-4-methyl-2-(*o*-tolyl)oxazol-5(4*H*)-one (4af)

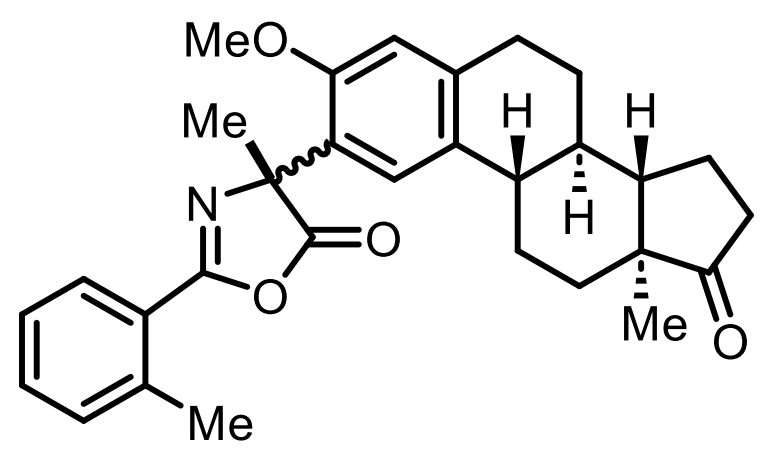

***4af** was prepared following a slightly modified General Procedure E from azlactone **1e** (37.8 mg, 0.2 mmol) and o-methylated estrone (**2l**, 170.4 mg, 0.6 mmol) with electrocatalyst **3b** (3.7 mg, 0.01 mmol), [collidinium·BF_4] (83.6 mg, 0.4 mmol) and collidine (13 µL, 0.1 mmol) in a* 1:1 mixture of $MeCN/CH_2Cl_2$ *(8 mL). The product was isolated by chromatography on silica gel (hexanes/ethyl acetate = 9:1).*

34% yield (32 mg, 0.07 mmol), yellow oil. *This product has been characterized as a mixture of diastereoisomers* (dr = 1:1). **^{1}H NMR** (400 MHz, $CDCl_3$) *δ* (for mixture of diastereomers) 7.91 – 7.84 (m, 1H), 7.47 – 7.38 (m, 2H), 7.33 – 7.26 (m, 2H), 6.60 (s, 1H), 3.69 (s, 3H), 2.94 – 2.85 (m, 2H), 2.61 (s, 1.5H), 2.60 (s, 1.5H), 2.56 – 2.42 (m, 2H), 2.35 – 2.25 (m, 1H), 2.21 – 1.96 (m, 4H), 1.88 (s, 1.5H), 1.87 (s, 1.5H), 1.72 – 1.37 (m, 7H), 1.37 – 1.20 (m, 1H), 0.93 (s, 1.5H), 0.92 (s, 1.5H) ppm; **^{13}C NMR** (101 MHz, $CDCl_3$) δ (for mixture of diastereomers) 181.4, 181.3, 161.4, 161.3, 155.4, 155.4, 139.4, 139.3, 138.9, 138.9, 132.4, 132.3, 131.7, 131.7, 131.6, 129.9, 126.0, 125.9, 125.8, 124.5, 124.4, 123.5, 112.1, 112.1, 68.6, 55.8, 55.8, 50.5, 50.5, 48.1, 44.2, 44.2, 38.4, 38.3, 36.0, 31.7, 29.7, 26.6, 26.5, 26.1,

26.0, 22.9, 22.9, 21.9, 21.8, 21.7, 14.0.ppm. **HRMS (ESI)** Calculated for $C_{30}H_{34}NO_4^+$ ($[M+H]^+$) 472.2482. Found 472.2482.

4-methyl-4-(2-oxo-2-phenylethyl)-2-(*o*-tolyl)oxazol-5(4*H*)-one (10a)

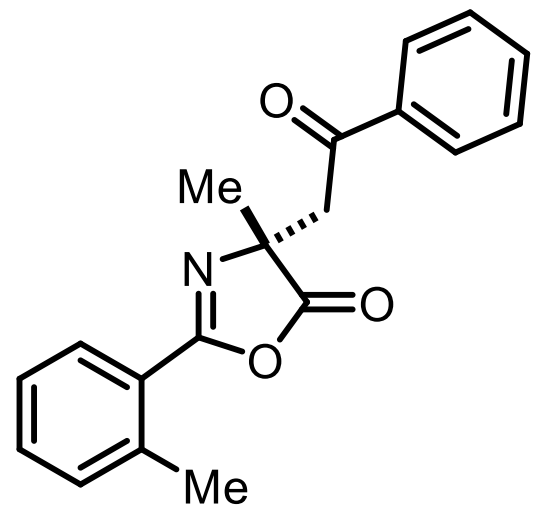

***10a** was prepared following General Procedure E from azlactone **1e** (37.8 mg, 0.2 mmol) and tert-butyldimethyl((1-phenylvinyl)oxy)silane (**9a**, 140.4 mg, 0.6 mmol) with electrocatalyst **3b** (3.7 mg, 0.01 mmol), [collidinium·BF$_4$] (83.6 mg, 0.4 mmol) and collidine (13 μL, 0.1 mmol) in MeCN (8 mL). The product was isolated by chromatography on silica gel (hexanes/ethyl acetate = 4:1).*

78% yield (48 mg, 0.16 mmol), white solid. **^{1}H NMR** (400 MHz, $CDCl_3$) δ 7.93 – 7.87 (m, 3H), 7.61 – 7.50 (m, 1H), 7.47 – 7.36 (m, 3H), 7.32 – 7.23 (m, 2H), 3.94 (d, *J* = 18.0 Hz, 1H), 3.54 (d, *J* = 18.0 Hz, 1H), 2.60 (s, 3H), 1.61 (s, 3H) ppm; **^{13}C NMR** (101 MHz, $CDCl_3$) δ 195.6, 181.0, 162.3, 139.5, 135.8, 133.8, 131.8, 131.6, 130.1, 128.8, 128.2, 126.0, 125.6, 66.2, 46.7, 25.0, 21.9 ppm. **HRMS (ESI)** Calculated for $C_{19}H_{18}NO_3^+$ ($[M+H]^+$) 308.1281. Found 308.1294.

4-(2-(4-bromophenyl)-2-oxoethyl)-4-methyl-2-(*o*-tolyl)oxazol-5(4*H*)-one (10b)

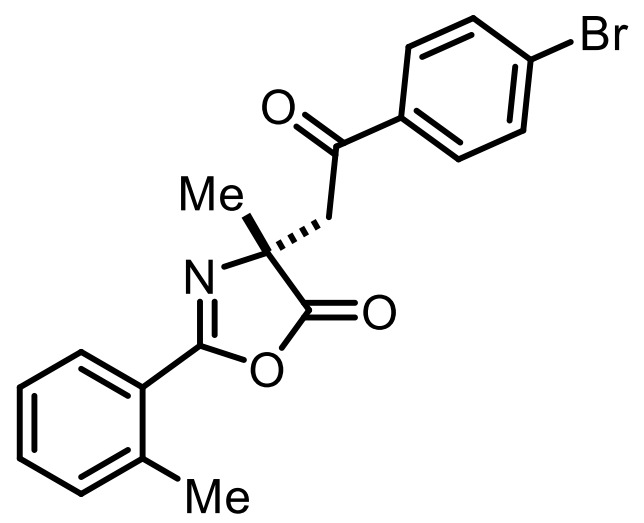

***10b** was prepared following General Procedure E from azlactone **1e** (37.8 mg, 0.2 mmol) and ((1-(4-bromophenyl)vinyl)oxy)(tert-butyl)dimethylsilane (**9b**, 187.8 mg, 0.6 mmol) with electrocatalyst **3b** (3.7 mg, 0.01 mmol), [collidinium·BF$_4$] (83.6 mg, 0.4 mmol) and collidine (13 μL, 0.1 mmol) in MeCN (8 mL). The product was isolated by chromatography on silica gel (hexanes/ethyl acetate = 4:1).*

70% yield (54 mg, 0.14 mmol), white solid. **^{1}H NMR** (400 MHz, $CDCl_3$) δ 7.89 (dd, *J* = 7.8, 1.5 Hz, 1H), 7.79 – 7.73 (m, 2H), 7.62 – 7.55 (m, 2H), 7.40 (td, *J* = 7.5, 1.5 Hz, 1H), 7.32 – 7.25 (m, 2H), 3.88 (d, *J* = 18.0 Hz, 1H), 3.50 (d, *J* = 18.0 Hz, 1H), 2.59 (s, 3H), 1.61 (s, 3H) ppm; **^{13}C NMR** (101 MHz, $CDCl_3$) δ 194.7, 180.8, 162.4, 139.5, 134.5, 132.2, 131.8, 131.7, 130.1, 129.7, 129.1, 126.0, 125.5, 66.1, 46.5, 25.0, 21.9 ppm. **HRMS (ESI)** Calculated for $C_{19}H_{17}BrNO_3^+$ ($[M+H]^+$) 386.0386. Found 386.0395.

4-(2-([1,1'-biphenyl]-4-yl)-2-oxoethyl)-4-methyl-2-(*o*-tolyl)oxazol-5(4*H*)-one (10c)

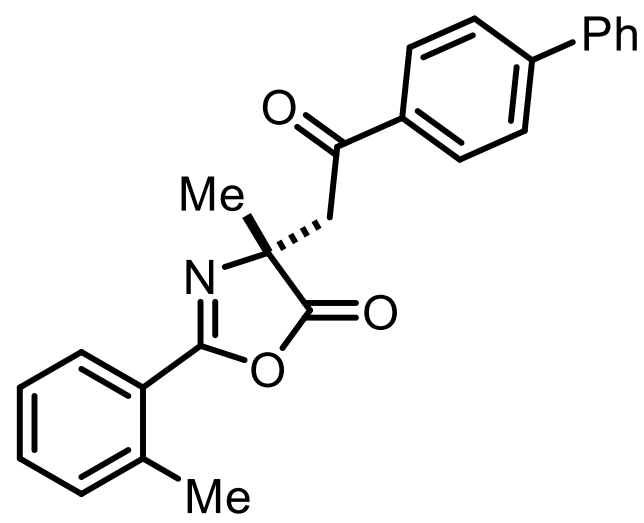

***10c** was prepared following General Procedure E from azlactone **1e** (37.8 mg, 0.2 mmol) and ((1-([1,1'-biphenyl]-4-yl)vinyl)oxy)(tert-butyl)dimethylsilane (**9c**, 186.0 mg, 0.6 mmol) with electrocatalyst **3b** (3.7 mg, 0.01 mmol), [collidinium·BF$_4$] (83.6 mg, 0.4 mmol) and collidine (13 μL, 0.1 mmol) in MeCN (8 mL). The product was isolated by chromatography on silica gel (hexanes/CH_2Cl_2 = 1:3).*

62% yield (47 mg, 0.12 mmol), white solid. **^{1}H NMR** (400 MHz, $CDCl_3$) δ 8.03 – 7.95 (m, 2H), 7.92 (dd, *J* = 7.9, 1.5 Hz, 1H), 7.69 – 7.65 (m, 2H), 7.64 – 7.59 (m, 2H), 7.50 – 7.44 (m, 2H), 7.44 – 7.38 (m, 2H), 7.33 – 7.26 (m, 2H), 3.98 (d, *J* = 17.9 Hz, 1H), 3.58 (d, *J* = 17.9 Hz, 1H), 2.63 (s, 3H), 1.64 (s, 3H) ppm; **^{13}C NMR** (101 MHz, $CDCl_3$) δ 195.2, 181.0, 162.3, 146.5, 139.7, 139.6, 134.5, 131.8, 131.7, 130.1, 129.1, 128.9, 128.5, 127.4, 127.4, 126.0, 125.6, 66.2, 46.7, 25.0, 21.9 ppm. **HRMS (ESI)** Calculated for $C_{25}H_{22}NO_3^+$ ($[M+H]^+$) 384.1594. Found 384.1598.

4-(2-(4-(*tert*-butyl)phenyl)-2-oxoethyl)-4-methyl-2-(*o*-tolyl)oxazol-5(4*H*)-one (10d)

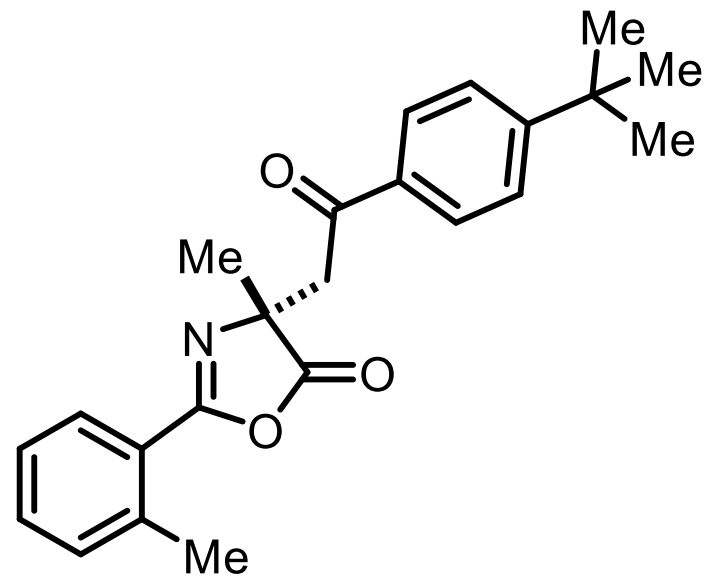

***10d** was prepared following General Procedure E from azlactone **1e** (37.8 mg, 0.2 mmol) and tert-butyl((1-(4-(tert-butyl)phenyl)vinyl)oxy)dimethylsilane (**9d**, 174.0 mg, 0.6 mmol) with electrocatalyst **3b** (3.7 mg, 0.01 mmol), [collidinium·BF_4] (83.6 mg, 0.4 mmol) and collidine (13 µL, 0.1 mmol) in MeCN (8 mL). The product was isolated by chromatography on silica gel (hexanes/ethyl acetate = 4:1).*

70% yield (51 mg, 0.14 mmol), white solid. **^{1}H NMR** (400 MHz, $CDCl_3$) δ 7.89 (dd, *J* = 7.7, 1.5 Hz, 1H), 7.87 – 7.82 (m, 2H), 7.48 – 7.43 (m, 2H), 7.40 (td, *J* = 7.5, 1.5 Hz, 1H), 7.31 – 7.24 (m, 2H), 3.92 (d, *J* = 17.9 Hz, 1H), 3.51 (d, *J* = 17.9 Hz, 1H), 2.59 (s, 3H), 1.61 (s, 3H), 1.33 (s, 9H) ppm; **^{13}C NMR** (101 MHz, $CDCl_3$) δ 195.2, 181.1, 162.2, 157.7, 139.6, 133.3, 131.7, 131.6, 130.1, 128.2, 126.0, 125.8, 125.6, 66.2, 46.7, 35.3, 31.2, 25.0, 21.9 ppm. **HRMS (ESI)** Calculated for $C_{23}H_{26}NO_3^+$ ($[M+H]^+$) 364.1907. Found 364.1916.

4-methyl-4-(2-(naphthalen-2-yl)-2-oxoethyl)-2-(*o*-tolyl)oxazol-5(4*H*)-one (10e)

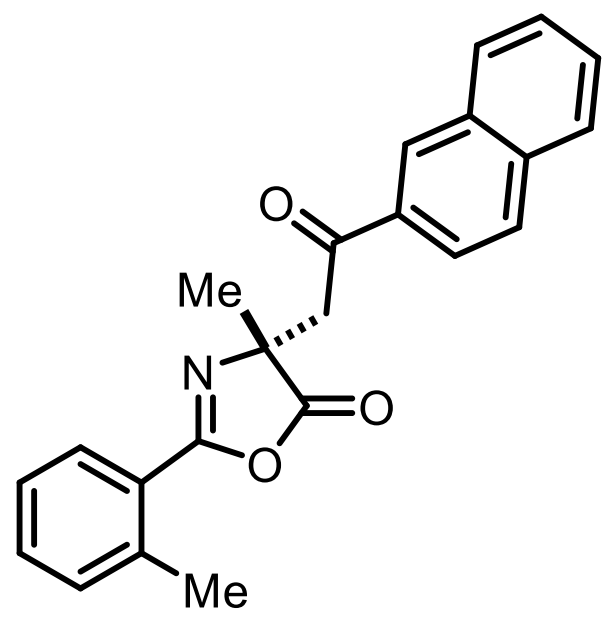

***10e** was prepared following General Procedure E from azlactone **1e** (37.8 mg, 0.2 mmol) and tert-butyldimethyl((1-(naphthalen-2-yl)vinyl)oxy)silane (**9e**, 174.0 mg, 0.6 mmol) with electrocatalyst **3c** (3.4 mg, 0.01 mmol), [collidinium·BF_4] (83.6 mg, 0.4 mmol) and collidine (13 µL, 0.1 mmol) in MeCN (8 mL). The product was isolated by chromatography on silica gel (hexanes/ethyl acetate = 4:1).*

31% yield (22 mg, 0.06 mmol), colourless oil. **^{1}H NMR** (400 MHz, $CDCl_3$) δ 8.45 (d, *J* = 1.7 Hz, 1H), 7.98 – 7.89 (m, 3H), 7.89 – 7.84 (m, 2H), 7.61 (ddd, *J* = 8.2, 6.9, 1.5 Hz, 1H), 7.56 (ddd, *J* = 8.2, 6.9, 1.5 Hz, 1H), 7.40 (td, *J* = 7.5, 1.5 Hz, 1H), 7.33 – 7.24 (m, 2H), 4.10 (d, *J* = 17.9 Hz, 1H), 3.67 (d, *J* = 17.9 Hz, 1H), 2.61 (s, 3H), 1.67 (s, 3H).; **^{13}C NMR** (101 MHz, $CDCl_3$) δ 195.6, 181.1, 162.4, 139.6, 136.0, 133.2, 132.5, 131.8, 131.7, 130.2, 130.2, 129.7, 128.9, 128.8, 128.0, 127.1, 126.1, 125.6, 123.7, 66.3, 46.8, 25.0, 21.9 ppm **HRMS (ESI)** Calculated for $C_{23}H_{20}NO_3^+$ ($[M+H]^+$) 358.1438. Found 358.1440.

4-(2-(furan-2-yl)-2-oxoethyl)-4-methyl-2-(*o*-tolyl)oxazol-5(4*H*)-one (10f)

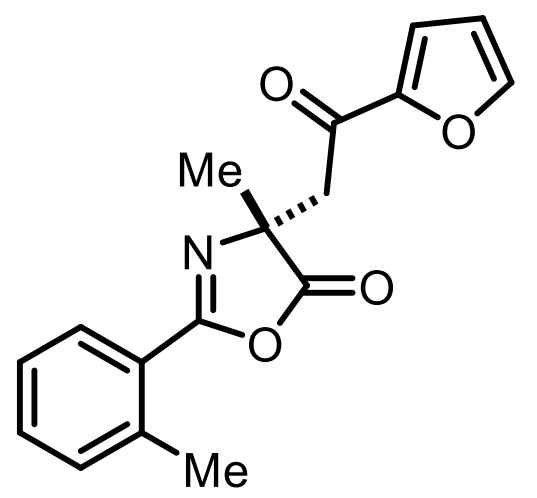

***10f** was prepared following General Procedure E from azlactone **1e** (37.8 mg, 0.2 mmol) and tert-butyl((1-(furan-2-yl)vinyl)oxy)dimethylsilane (**9f**, 134.4 mg, 0.6 mmol) with electrocatalyst **3c** (3.4 mg, 0.01 mmol), [collidinium·BF_4] (83.6 mg, 0.4 mmol) and collidine (13 µL, 0.1 mmol) in MeCN (8 mL). The product was isolated by chromatography on silica gel (hexanes/CH_2Cl_2 = 1:9).*

43% yield (26 mg, 0.09 mmol), white solid. **^{1}H NMR** (400 MHz, $CDCl_3$) δ 7.87 (dd, *J* = 7.9, 1.5 Hz, 1H), 7.57 (dd, *J* = 1.7, 0.8 Hz, 1H), 7.39 (td, *J* = 7.5, 1.5 Hz, 1H), 7.30 – 7.23 (m, 2H), 7.18 (dd, *J* = 3.6, 0.8 Hz, 1H), 6.53 (dd, *J* = 3.6, 1.7 Hz, 1H), 3.79 (d, *J* = 17.8 Hz, 1H), 3.40 (d, *J* = 17.9 Hz, 1H), 2.58 (s, 3H), 1.59 (s, 3H) ppm; **^{13}C NMR** (101 MHz, $CDCl_3$) δ 184.7, 180.7, 162.4, 151.9, 146.9, 139.6, 131.8, 131.7, 130.1, 126.0, 125.5, 117.9, 112.7, 66.0, 46.1, 24.9, 21.9 ppm. **HRMS (ESI)** Calculated for $C_{17}H_{16}NO_4^+$ ($[M+H]^+$) 298.1074. Found 298.1079.

4-(2-(benzofuran-2-yl)-2-oxoethyl)-4-methyl-2-(*o*-tolyl)oxazol-5(4*H*)-one (10g)

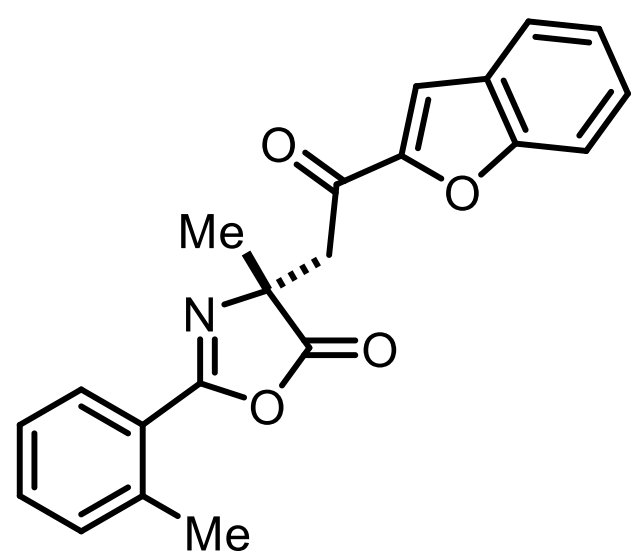

***10g** was prepared following General Procedure E from azlactone **1e** (37.8 mg, 0.2 mmol) and ((1-(benzofuran-2-yl)vinyl)oxy)(tert-butyl)dimethylsilane (**9g**, 164.4 mg, 0.6 mmol) with electrocatalyst **3b** (3.4 mg, 0.01 mmol), [collidinium·BF_4] (83.6 mg, 0.4 mmol) and collidine (13 µL, 0.1 mmol) in MeCN (8 mL). The product was isolated by chromatography on silica gel (hexanes/ethyl acetate = 4:1).*

38% yield (26 mg, 0.08 mmol), yellow oil. **^{1}H NMR** (400 MHz, $CDCl_3$) δ 7.89 (dd, *J* = 7.8, 1.5 Hz, 1H), 7.69 (dt, *J* = 7.8, 0.9 Hz, 1H), 7.55 (dq, *J* = 8.4, 1.0 Hz, 1H), 7.52 – 7.45 (m, 2H), 7.39 (td, *J* = 7.5, 1.5 Hz, 1H), 7.33 – 7.23 (m, 3H), 3.95 (d, *J* = 18.0 Hz, 1H), 3.54 (d, *J* = 18.0 Hz, 1H), 2.59 (s, 3H), 1.64 (s, 3H) ppm; **^{13}C NMR** (101 MHz, $CDCl_3$) δ 186.8, 180.7, 162.5, 155.8, 151.7, 139.6, 131.9, 131.7, 130.1, 128.8, 127.0, 126.0, 125.4, 124.2, 123.6, 113.5, 112.6, 66.0, 46.5, 24.9, 21.9 ppm. **HRMS (ESI)** Calculated for $C_{21}H_{18}NO_4^+$ ($[M+H]^+$) 348.1230. Found 348.1241.

(3*S*,8*S*,9*S*,10*R*,13*S*,14*S*,17*S*)-10,13-dimethyl-17-(2-(4-methyl-5-oxo-2-(*o*-tolyl)-4,5-dihydrooxazol-4-yl)acetyl)-2,3,4,7,8,9,10,11,12,13,14,15,16,17-tetradecahydro-1*H*-cyclopenta[*a*]phenanthren-3-yl acetate (10h)

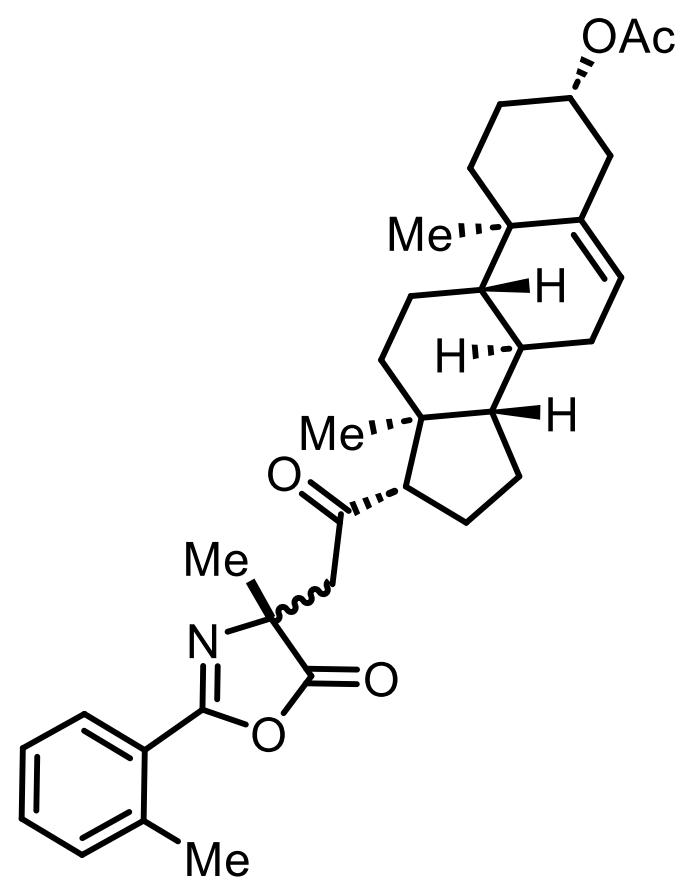

***10h** was prepared following a slightly modified General Procedure E from azlactone **1e** (37.8 mg, 0.2 mmol) and the SEE of acetylated pregnenolone (**9h**, 283 mg, 0.6 mmol) with electrocatalyst **3b** (3.7 mg, 0.01 mmol), [collidinium·BF_4] (83.6 mg, 0.4 mmol) and collidine (13 µL, 0.1 mmol) in a* 1:1 mixture of $MeCN/CH_2Cl_2$ *(8 mL). The product was isolated by chromatography on silica gel (hexanes/ethyl acetate = 9:1).*

25% global yield (27 mg, 0.05 mmol) of 1:1 diastereomeric mixture. Pure samples of each diastereoisomer were obtained upon careful chromatographic purification.

diastereomer *a*, yellow oil. **^{1}H NMR** (400 MHz, $CDCl_3$) δ 7.86 (dd, *J* = 8.1, 1.3 Hz, 1H), 7.39 (td, *J* = 7.5, 1.5 Hz, 1H), 7.30 – 7.24 (m, 2H), 5.36 (dt, *J* = 5.2, 1.8 Hz, 1H), 4.66 – 4.53 (m, 1H), 3.27 (d, *J* = 18.1 Hz, 1H), 3.01 (d, *J* = 18.1 Hz, 1H), 2.60 (s, 3H), 2.51 – 2.44 (m, 1H), 2.36 – 2.26 (m, 2H), 2.16 – 2.06 (m, 2H), 2.03 (s, 3H), 1.97 (dtd, *J* = 17.1, 5.1, 2.0 Hz, 1H), 1.91 – 1.83 (m, 2H), 1.67 – 1.50 (m, 6H), 1.48 (s, 3H), 1.46 – 1.41 (m, 2H), 1.23 – 1.07 (m, 3H), 1.04 – 0.95 (m, 4H), 0.53 (s, 3H) ppm; **^{13}C NMR** (101 MHz, $CDCl_3$) δ 207.3, 181.0, 170.7, 162.0, 139.8, 139.5, 131.7, 131.7, 130.2, 126.0, 125.4, 122.4, 73.9, 66.1, 62.0, 57.1, 52.1, 49.9, 44.8, 39.0, 38.2, 37.1, 36.7, 31.9, 31.9, 27.8, 24.7, 24.6, 23.0, 22.1, 21.6, 21.1, 19.4, 13.4 ppm. **HRMS (ESI)** Calculated for $C_{34}H_{44}NO_5^+$ ($[M+H]^+$) 546.3214. Found 546.3213.

diastereomer *b*, white solid. **^{1}H NMR** 400 MHz, $CDCl_3$) δ 7.84 (dd, *J* = 8.3, 1.5 Hz, 1H), 7.42 – 7.37 (m, 1H), 7.30 – 7.26 (m, 2H), 5.36 (m, 1H), 4.60 (m, 1H), 3.20 (d, *J* = 17.9 Hz, 1H), 3.02 (d, *J* = 18.0 Hz, 1H), 2.59 (s, 3H), 2.47 – 2.40 (m, 1H), 2.34 – 2.28 (m, 2H), 2.03 (s, 5H), 2.01 – 1.94 (m, 1H), 1.90 – 1.84 (m, 2H), 1.69 – 1.55 (m, 6H), 1.50 – 1.43 (m, 5H), 1.16 (m, 3H), 1.01 (m, 4H), 0.60 (s, 3H) ppm; **^{13}C NMR** (101 MHz, $CDCl_3$) δ 207.1, 181.0, 170.7, 162.2, 139.8, 139.4, 131.7, 131.6, 130.1, 126.0, 125.7, 122.4, 74.0, 66.2, 62.7, 57.0, 51.9, 50.0, 44.7, 38.9, 38.2, 37.1, 36.7, 31.9, 31.9, 27.8, 24.8, 24.6, 22.8, 21.8, 21.6, 21.1, 19.4, 13.3 ppm. **HRMS (ESI)** Calculated for $C_{34}H_{44}NO_5^+$ ($[M+H]^+$) 546.3214. Found 546.3213.

4-methyl-4-(2-phenylallyl)-2-(*o*-tolyl)oxazol-5(4*H*)-one (12)

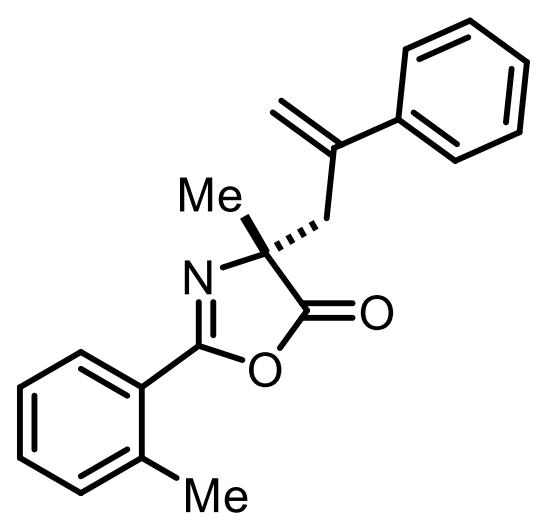

12 *was prepared following General Procedure E from azlactone* ***1e*** *(37.8 mg, 0.2 mmol) and trimethyl(2-phenylallyl)silane (****11****, 114.0 mg, 0.6 mmol) with electrocatalyst* ***3b*** *(3.7 mg, 0.01 mmol), [collidinium·BF_4] (83.6 mg, 0.4 mmol) and collidine (13 µL, 0.1 mmol) in MeCN (8 mL). The product was isolated by chromatography on silica gel (hexanes/ ethyl acetate = 8:1).*

59% yield (36 mg, 0.12 mmol), colourless oil. **^{1}H NMR** (400 MHz, $CDCl_3$) δ 7.55 (dd, *J* = 8.3, 1.5 Hz, 1H), 7.34 (td, *J* = 7.5, 1.5 Hz, 1H), 7.30 – 7.25 (m, 2H), 7.21 – 7.15 (m, 5H), 5.31 (s, 1H), 5.20 (s, 1H), 3.19 (d, *J* = 13.8 Hz, 1H), 3.10 (d, *J* = 13.8 Hz, 1H), 2.29 (s, 3H), 1.56 (s, 3H) ppm; **^{13}C NMR** (101 MHz, $CDCl_3$) δ 180.6, 159.8, 143.7, 141.2, 140.0, 131.7, 131.6, 130.1, 128.3, 127.6, 126.6, 125.8, 124.6, 118.1, 70.8, 43.7, 24.6, 22.0 ppm. **HRMS (ESI)** Calculated for $C_{20}H_{20}NO_2^+$ ($[M+H]^+$) 306.1489. Found 306.1482.

(*E*)-4-methyl-4-styryl-2-(*o*-tolyl)oxazol-5(4*H*)-one (14)

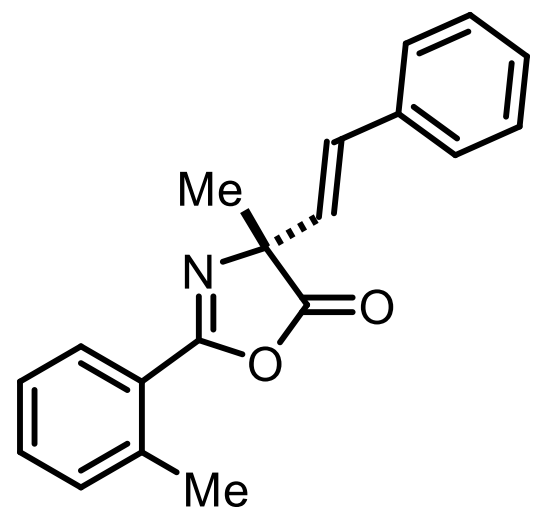

14 *was prepared following General Procedure E from azlactone* ***1e*** *(37.8 mg, 0.2 mmol) and potassium styryl trifluoroborate (****13****, 126.0 mg, 0.6 mmol) with electrocatalyst* ***3b*** *(3.7 mg, 0.01 mmol), [collidinium·BF_4] (83.6 mg, 0.4 mmol) and collidine (13 µL, 0.1 mmol) in MeCN (8 mL). The product was isolated by chromatography on silica gel (hexanes/ethyl acetate = 9:1).*

65% yield (38 mg, 0.13 mmol), colourless oil. **^{1}H NMR** (400 MHz, $CDCl_3$) δ 7.92 (dd, *J* = 7.9, 1.4 Hz, 1H), 7.49 – 7.43 (m, 1H), 7.42 – 7.38 (m, 2H), 7.36 – 7.30 (m, 4H), 7.29 – 7.24 (m, 1H), 6.78 (d, *J* = 16.0 Hz, 1H), 6.36 (d, *J* = 16.0 Hz, 1H), 2.74 (s, 3H), 1.73 (s, 3H) ppm; **^{13}C NMR** (101 MHz, $CDCl_3$) δ 179.0, 160.5, 140.0, 136.0, 132.1, 132.0, 131.3, 130.2, 128.8, 128.4, 127.0, 126.9, 126.2, 124.9, 70.4, 25.2, 22.4 ppm. **HRMS (ESI)** Calculated for $C_{19}H_{18}NO_2^+$ ($[M+H]^+$) 292.1332. Found 292.1327.

E. Electrochemical feasibility studies

E.1. ***Cyclic voltammetry*** *– Assessment of the selective indirect oxidation of the enolate*

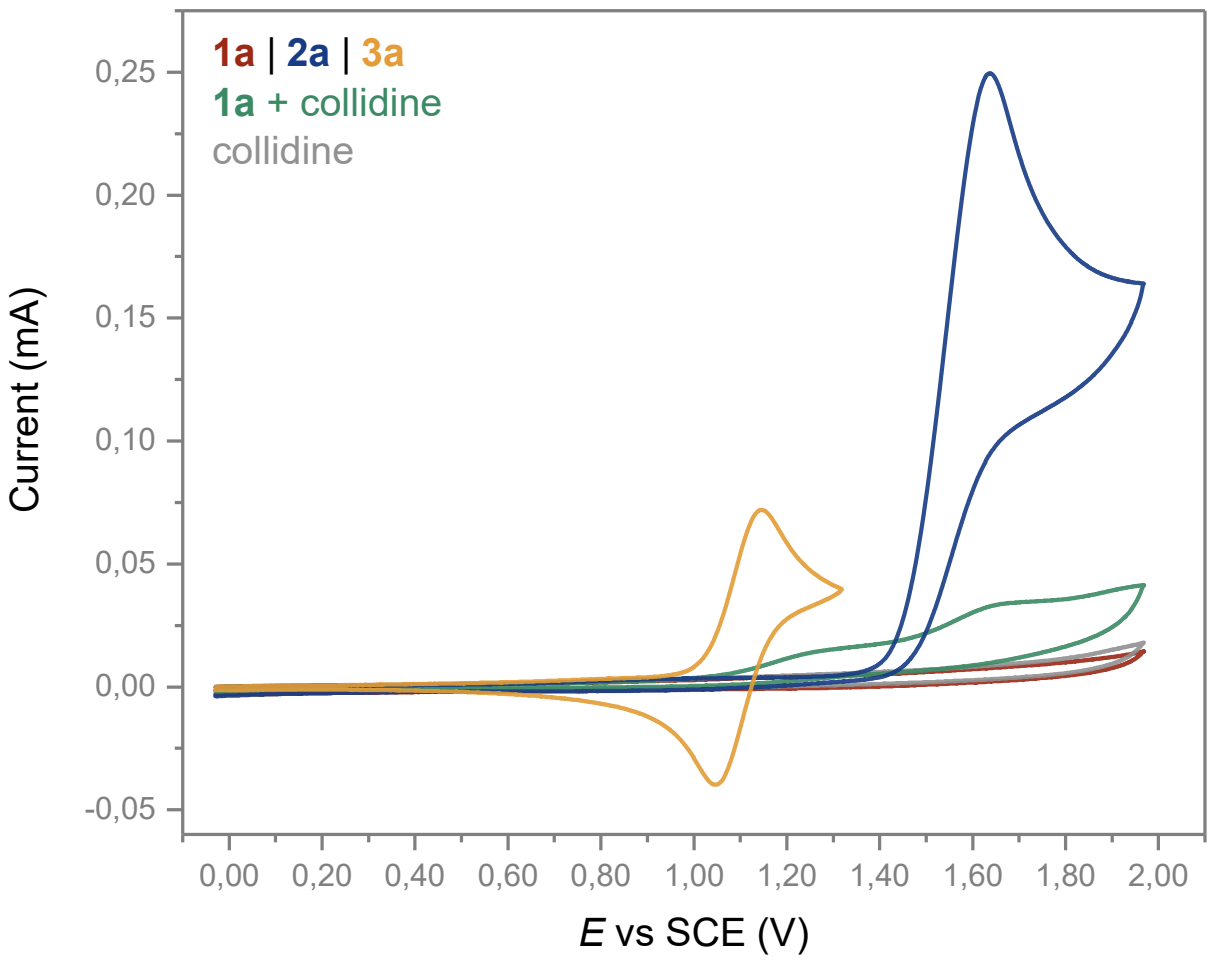

Figure S2. Cyclic voltammetry of the isolated reaction components: **1a** (100 mM); **2a** (10 mM); **3a** (5 mM); collidine (200 mM). CV recorded in MeCN with *n*Bu_4NPF_6 (0.1 M) as supporting electrolyte at 100 mV/s scan rate.

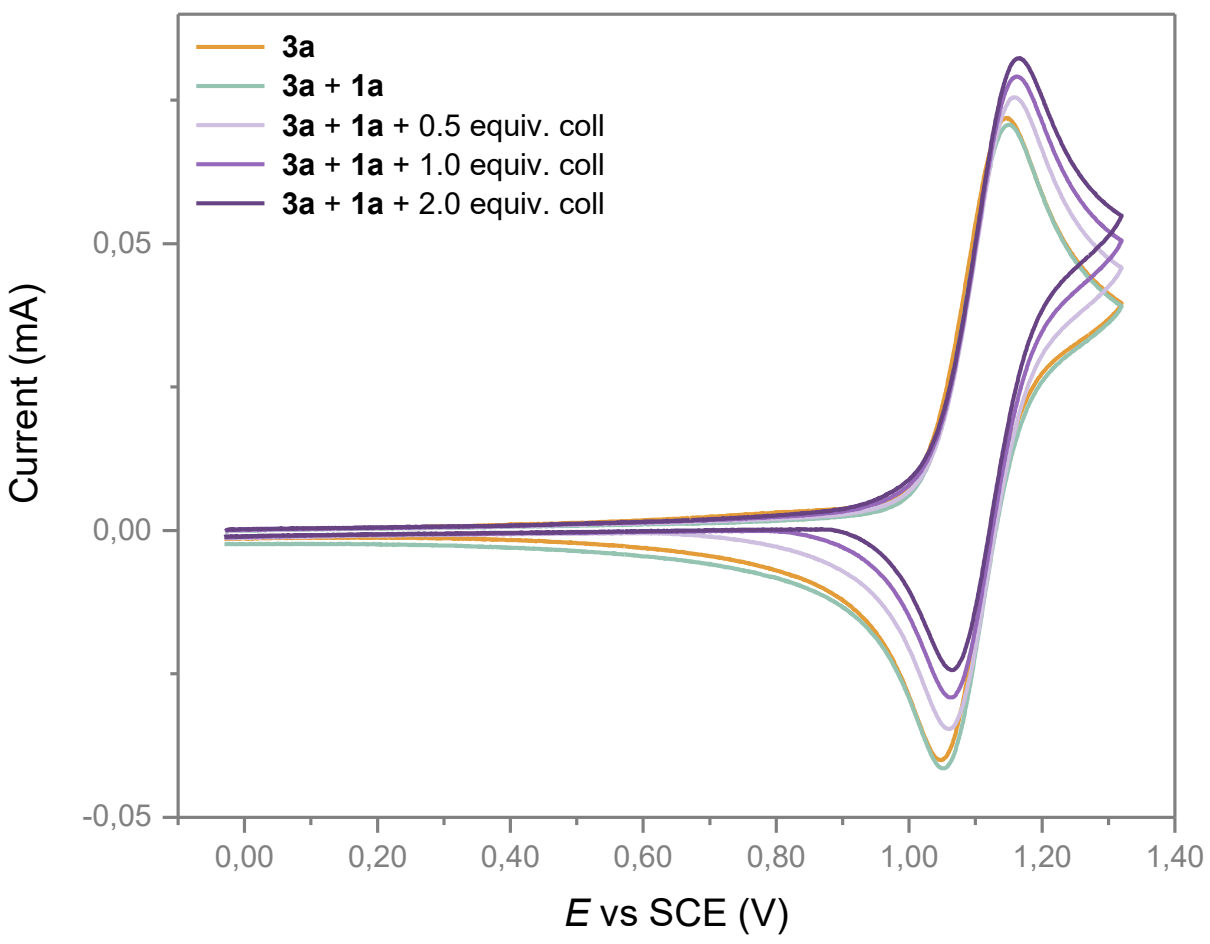

Figure S3. Catalytic current experiment between the electrocatalyst **3a** (5 mM) and the enolate **6a** (azlactone **1a** (100 mM) and 50 mM aliquot addition of collidine). Cyclic voltammetry recorded in MeCN with *n*Bu_4NPF_6 (0.1 M) as supporting electrolyte at 100 mV/s scan rate.

As shown in Figure S3, the electrocatalyst **3a** can indirectly oxidise the enolate of azlactone **1a**. At the same time, **3a** does not interact either with 1,3-dimethoxybenzene (**2a**) nor collidine (Figure S4).

Figure S5 shows the cyclic voltammetry for the oxidation of enolates of azlactones bearing a *o*-toluyl group at C2 derived from Valine (**6d**, blue trace), Alanine (**6e**, green trace), and Leucine (**6f**, red trace). All of them being very similar meaning that changes in the side chain of the amino acid do not significantly alter the oxidation potential of the enolate of the azlactone.

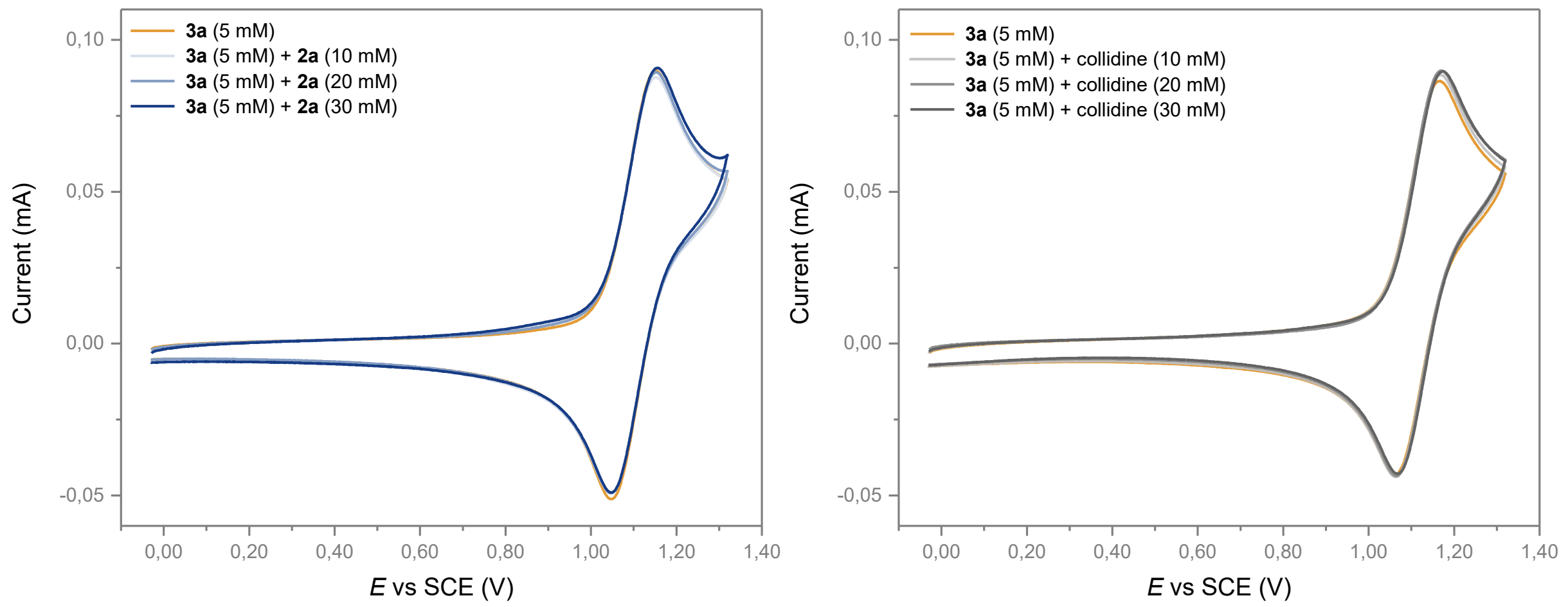

Figure S4. Catalytic current blank experiments between the electrocatalyst **3a** and the radical trap **2a** and collidine. Cyclic voltammetry recorded in MeCN with $n\text{Bu}_4\text{NPF}_6$ (0.1 M) as supporting electrolyte at 100 mV/s scan rate.

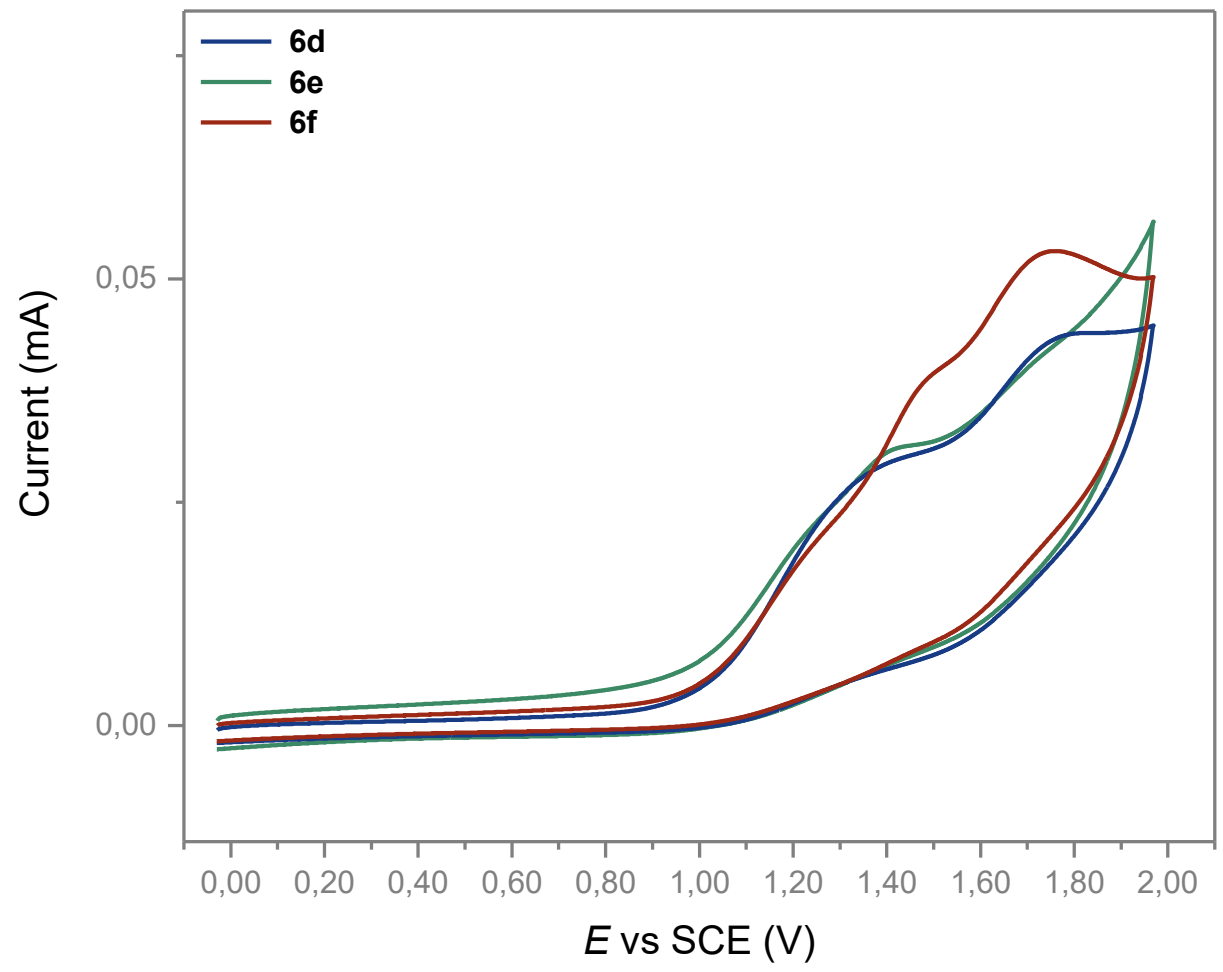

Figure S5. Cyclic voltammetry of the in situ generated enolates of azlactones **1d**, **1e**, and **1f** (100 mM) adding 2 equivalents of collidine in MeCN with $n\text{Bu}_4\text{NPF}_6$ (0.1 M) as supporting electrolyte at 100 mV/s scan rate.

E.2. ***Cyclic voltammetry*** *– Comparison of the oxidation potential of the enolate depending on the aromatic ring of the azlactone*

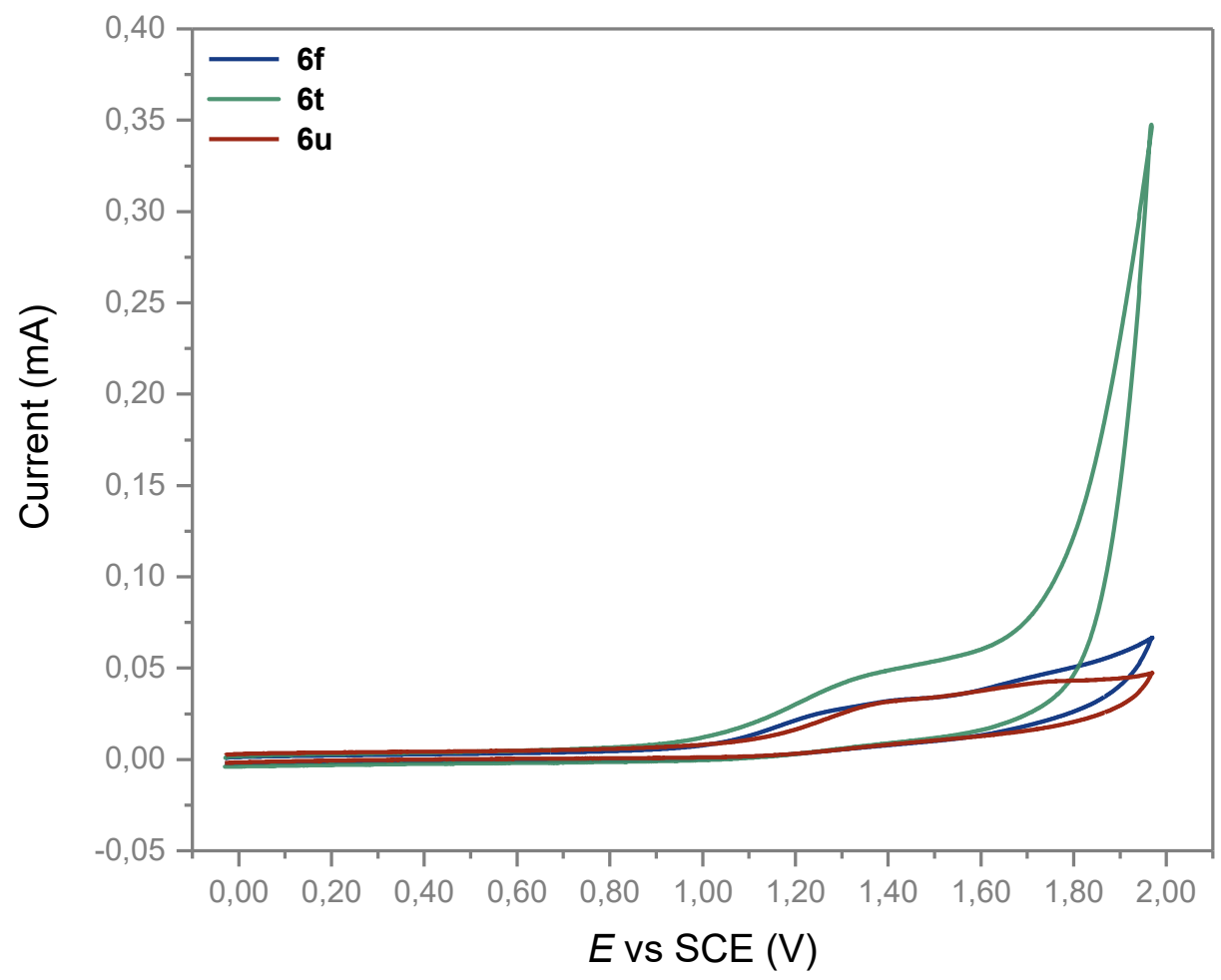

Figure S6. Cyclic voltammetry of the in situ generated enolates of azlactones **1f**, **1t**, and **1u** (100 mM) adding 2 equivalents of collidine in MeCN with $n\mathrm{Bu_4NPF_6}$ (0.1 M) as supporting electrolyte at 100 mV/s.

Substantial electronic variation at the C2 aryl group requires an adjustment of the electrocatalyst to ensure efficient indirect oxidation. In case of the electron-rich *p*-methoxy-substituted azlactone **1t** (Figure S6, green trace), which undergoes oxidation at a lower potential than the enolate of azlactone **1e** (Figure S6, blue trace), the most suitable catalyst was **3d** ($E_{1/2}$ = 0.75 V vs SCE). Conversely, the electron-deficient *p*-trifluoromethyl-substituted azlactone **1u** required the more oxidising electrocatalyst **3f** ($E_{1/2}$ = 1.11 V vs SCE), since the oxidation potential of its enolate was slightly higher (Figure S6, red trace).

E.3. Electron-rich SOMOphile generality – Electrocatalyst tuning

To ensure the general application of the methodology with a wide range of radical traps, different electrocatalysts were selected depending on the oxidation potential of the radical trap. Those electrocatalysts should be able to selectively oxidise the enolate of the azlactone in front of the oxidation of the electron rich SOMOphile. Figure S7 shows a series of triarylamine electrocatalysts with modified electronic properties and Figure S8 shows how despite lowering the oxidation potential of the electrocatalyst by 0.5 V (**3e**), there is still catalytic current with the enolate, meaning that the indirect oxidation is feasible. Table S7 shows the E_p values for all the radical traps used in this study, as well as the chosen electrocatalyst for each case in order to ensure a ΔE high enough to avoid the indirect oxidation of the radical trap.

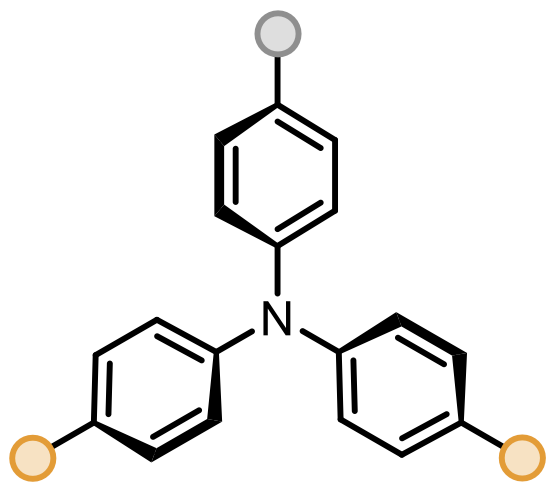

	(orange), (grey)	$E_{1/2}$ *vs.* SCE (V)
**3a**	Cl, Cl	+ 1.09
**3b**	Cl, ^{t}Bu	+ 1.00
**3c**	Cl, OMe	+ 0.88
**3d**	OMe, Cl	+ 0.75
**3e**	OMe, OMe	+ 0.57

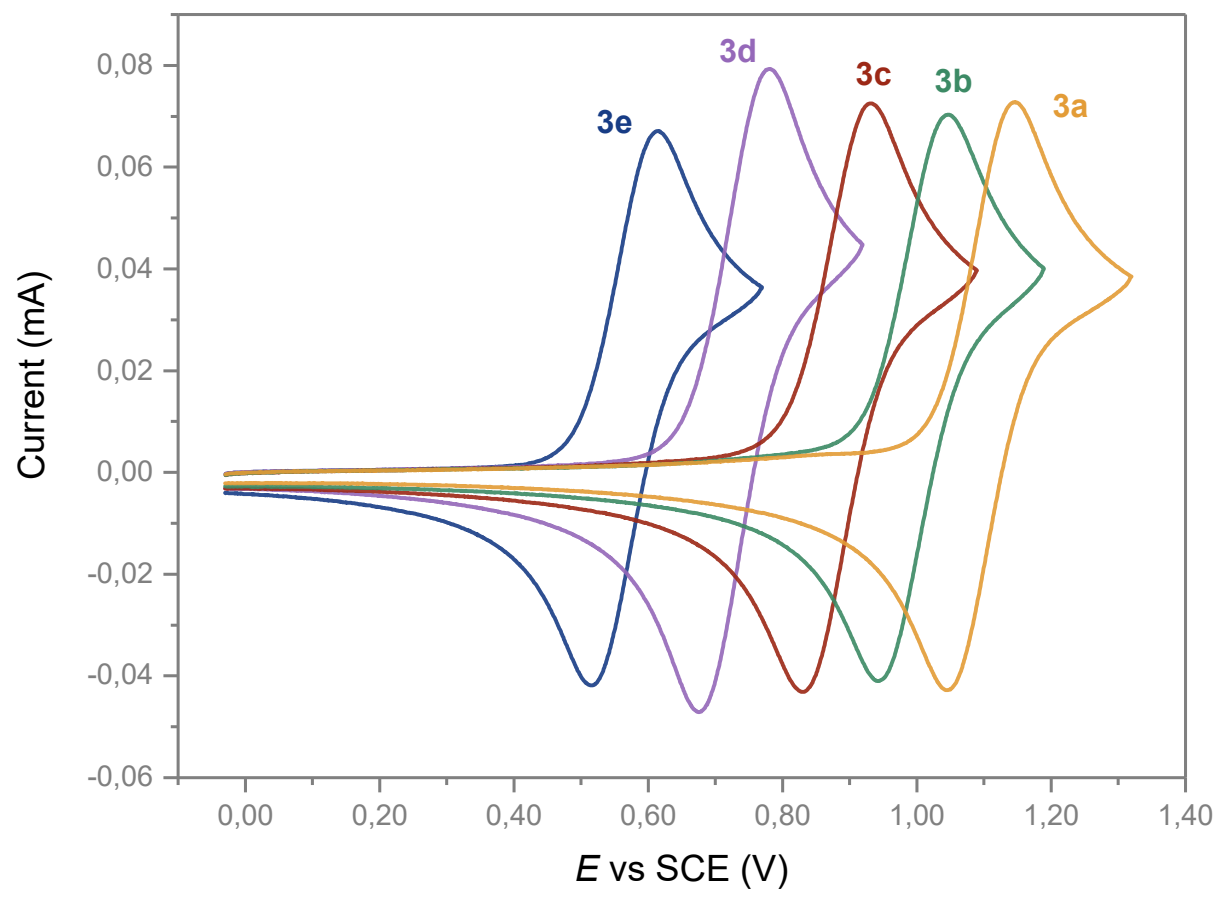

Figure S7. Cyclic voltammetry of the isolated electrocatalysts **3a-e** (5 mM) in MeCN with *n*Bu_4NPF_6 (0.1 M) as supporting electrolyte and 100 mV/s scan rate.

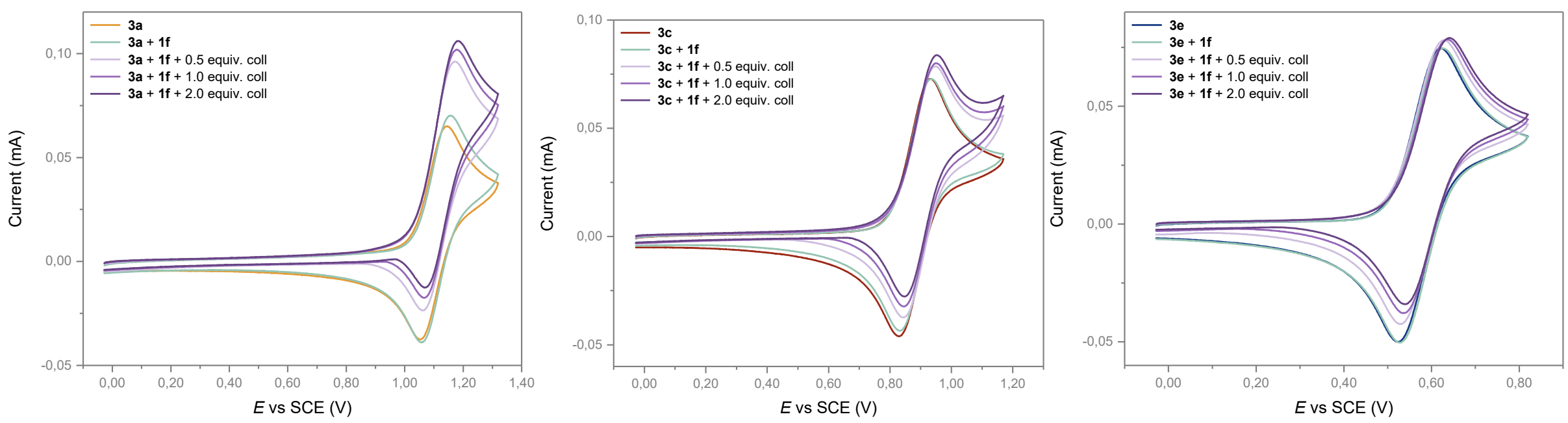

Figure S8. Catalytic current experiments between electrocatalysts **3a**, **3c**, and **3e** (5 mM), and the enolate **6f** (azlactone **1f** (100 mM) and 50 mM aliquot addition of collidine). Cyclic voltammetry recorded in MeCN with *n*Bu_4NPF_6 (0.1 M) as supporting electrolyte at 100 mV/s scan rate.

SOMOphile	E_p (V)	electrocatalyst	$E_{1/2}$ (V)	ΔE
1,3-dimethoxybenzene (**2a**)	1.67	**3a**	1.09	0.58
1,3,5-trimethoxybenzene (**2b**)	1.50	**3c**	0.88	0.62
2-methoxynaphtalene (**2c**)	1.61	**3a**	1.09	0.52
2,7-dimethoxynaphtalene (**2d**)	1.50	**3b**	1.00	0.50
2,6-dimethoxypyridine (**2e**)	1.67	**3a**	1.09	0.58
Boc-pyrrole (**2f**)	1.85	**3a**	1.09	0.76
2-acetyl-1-methylpyrrole (**2g**)	1.56	**3b**	1.00	0.56
methyl 1-methylpyrrole-2-carboxylate (**2h**)	1.66	**3b**	1.00	0.66
ethyl 1-methyl-1*H*-indole-2-carboxylate (**2i**)	1.49	**3c**	0.88	0.61
9-methyl-9*H*-carbazole (**2j**)	1.27	**3d**	0.75	0.52
2-methoxythiophene (**2k**)	1.50	**3c**	0.88	0.62
methylated estrone (**2l**)	1.65	**3b**	1.00	0.65
acetophenone SEE (**9a**)	1.63	**3b**	1.00	0.63
p-Br-acetophenone SEE (**9b**)	1.60	**3b**	1.00	0.60
p-Ph-acetophenone SEE (**9c**)	1.52	**3b**	1.00	0.52
p-^{t}Bu-acetophenone SEE (**9d**)	1.60	**3b**	1.00	0.60
naphthalene-acetophenone SEE (**9e**)	1.43	**3c**	0.88	0.55
furan-acetophenone SEE (**9f**)	1.43	**3c**	0.88	0.55
benzofuran-acetophenone SEE (**9g**)	1.54	**3b**	1.00	0.54
methylated estrone (**9h**)	1.65	**3b**	1.00	0.65
acetylated pregnenolone SEE (**9i**)	1.63	**3b**	1.00	0.63
trimethylsilyl(2-phenylallyl)silane (**11**)	1.55	**3b**	1.00	0.55
potassium *trans*-styryl trifluoroborate (**13**)	1.53	**3b**	1.00	0.53

Table S7. E_p values for the radical traps used in this study and comparison with the $E_{1/2}$ of the corresponding electrocatalyst. Values determined by cyclic voltammetry in a 10 mM solution of **2**, **9**, **11** or **13** and a 5 mM solution of **3** in MeCN with nBu$_4$NPF$_6$ (0.1 M) as supporting electrolyte at 100 mV/s scan rate.

Figure S9 shows how 1,3,5-trimethoxybenzene (**2b**) interacts with **3a**, while **3c** remains untouched when adding increasing amounts of **2b**. In a similar way, **3a** indirectly oxidises the silyl enol ether of 4-*tert*-butylacetophenone (**9d**), but **3b** cannot, ensuring selectivity towards the oxidation of the enolate (Figure S10). In Figure S11, two more examples are shown: **3a** cannot indirectly oxidise the 2,6-dimethoxypyridine (**2e**), as well as **3b** cannot indirectly oxidise **2h**.

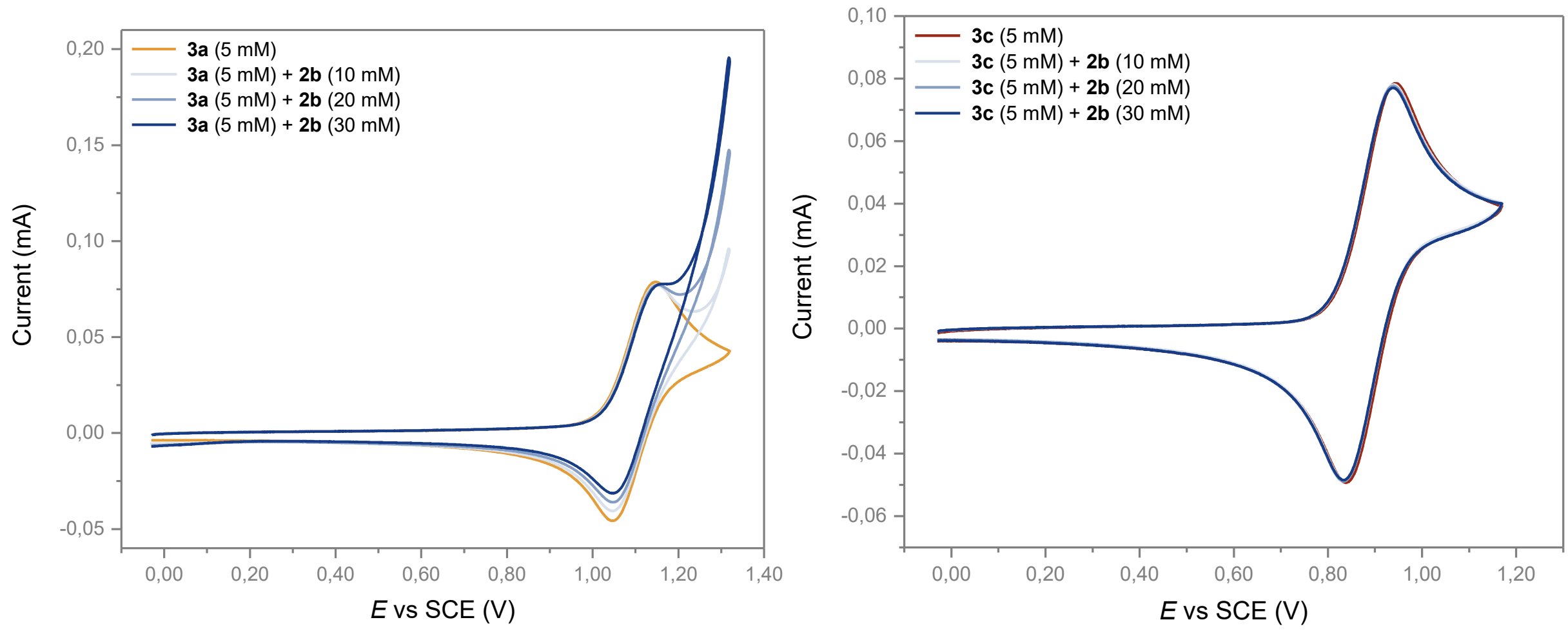

Figure S9. Catalytic current experiments between electrocatalysts **3a**, and **3c** (5 mM), and 1,3,5-trimethoxybenzene (**2b**). Cyclic voltammetry recorded in MeCN with $n\mathrm{Bu_4NPF_6}$ (0.1 M) as supporting electrolyte at 100 mV/s scan rate.

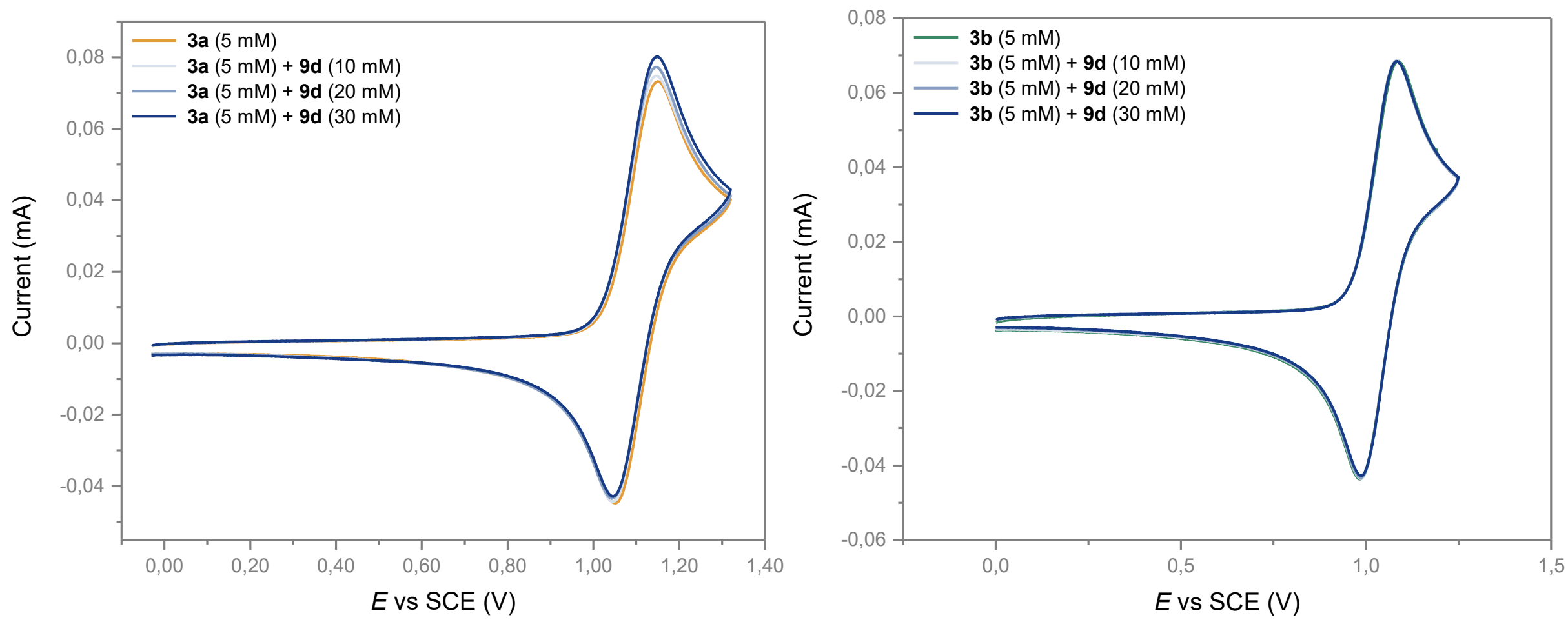

Figure S10. Catalytic current experiments between electrocatalysts **3a**, and **3b** (5 mM), and the silyl enol ether of 4-*tert*-butylacetophenone (**9d**). Cyclic voltammetry recorded in MeCN with $n\mathrm{Bu_4NPF_6}$ (0.1 M) as supporting electrolyte at 100 mV/s scan rate.

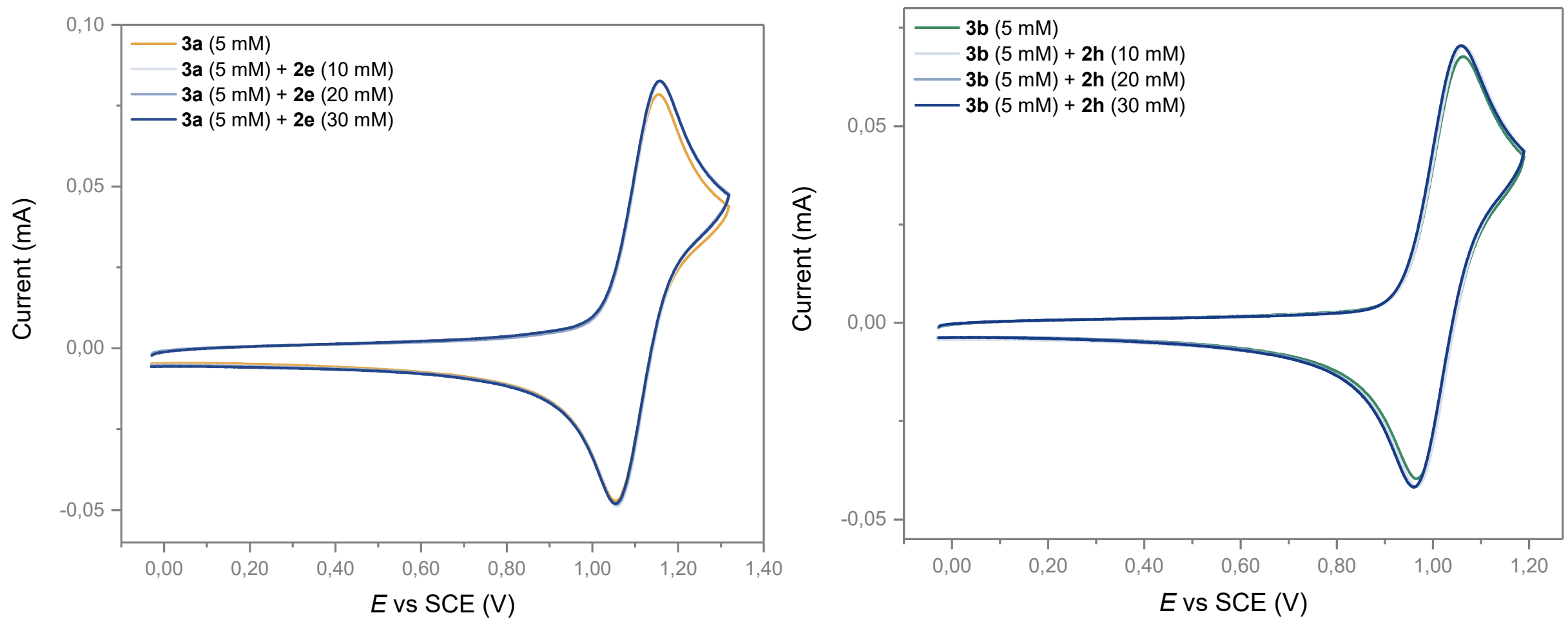

Figure S11. Catalytic current experiments between electrocatalyst **3a** (5 mM) and 2,6-dimethoxypyridine (**2e**); and between **3b** (5 mM), and methyl 1-methylpyrrole-2-carboxylate (**2h**). Cyclic voltammetry recorded in MeCN with $n\mathrm{Bu_4NPF_6}$ (0.1 M) as supporting electrolyte at 100 mV/s scan rate.

E.4. Fate of the excess of SOMOphiles

entry	yield (%)[a]	unreacted SOMOphile (equiv.) [a]	unreacted SOMOphile (%)[a]
**4d**	58	2	83
**4e**	79	1.9	86
**4f**	68	2.3	99
**4g**	62	2.2	92
**4h**	77	2.1	94
**4i**	62	2.0	84
**4j**	48	1.9	75
**4k**	62	1.7	71
**4l**	62	2.2	92
**4m**	77	1.7	76
**4n**	79	1.9	86
**4o**	54	1.5	61
**4p**	70	2.2	96
**4q**	55	1.9	78
**4r**	69	1.2	52
**4s**	66	1.9	81
**4t**	33	2.3	86
**4u**	28	1.7	63
**4v**	61	2.3	96
**4w**	20	2.6	93
**4x**	67	2.3	99
**4y**	51	2.1	84
**4z**	52	1.9	77
**4aa**[b]	63	8.3	89
**4ab**[b]	54	3.3	35
**4ac**[c]	57	3.7	89
**4ad**[b]	61	9.1	97
**4ae**	46	0.7	28
**4af**	34	2.5	94
**10a**	78	1.8	81
**10b**	70	2.0	87
**10c**	62	1.9	80
**10d**	70	1.3	57
**10e**	31	2.0	74
**10f**	43	0.8	31
**10g**	38	1.7	65
**10h**	25	1.6	58
**12**	59	1.9	79
**14**	65	1.8	77

Table S8. Analysis of the crude reaction mixtures to determine the fate of the excess of the electron-rich SOMOphiles. [a] Yield and remaining amount of electron-rich SOMOphile were determined by ^{1}H NMR spectroscopy using 1,3,5-trimethoxybenzene as the internal standard. [b] Reaction run with 5 equiv. of electron-rich SOMOphile. [c] Reaction run with 10 equiv. of electron-rich SOMOphile.

As summarised in Table S8, most entries in the substrate scope show that 60–99% of the excess of SOMOphile remains unreacted. The only notable exceptions are the formation of **4ab** from the *N*-methylpyrrole derivative **2h**, **4ae** from 2-methoxythiophene **2k**, and **10f** from the furyl-derived silyl enol

ether **9f**, for which less than 35% of the excess of SOMOphile is recovered. We attribute the increased consumption of these substrates to competing decomposition pathways, such as oligomerisation/polymerisation or oxidative ring-opening.

F. Unsuccessful substrates

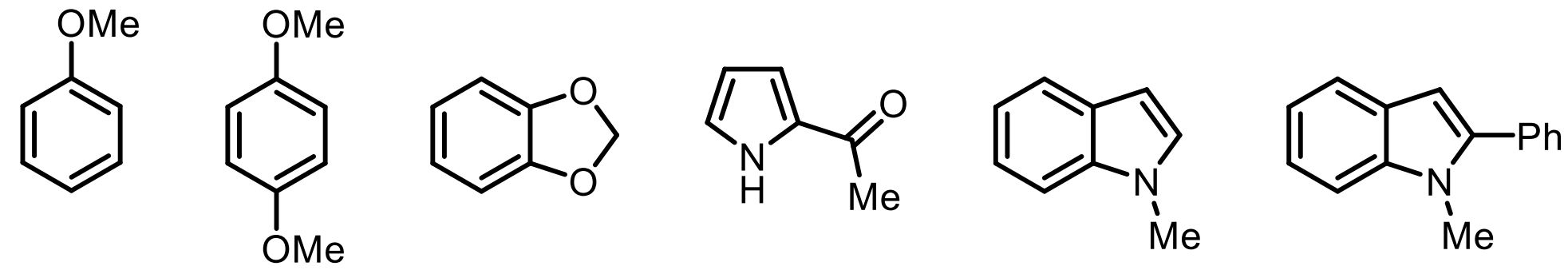

S16 **S17** **S18** **S19** **S20** **S21**

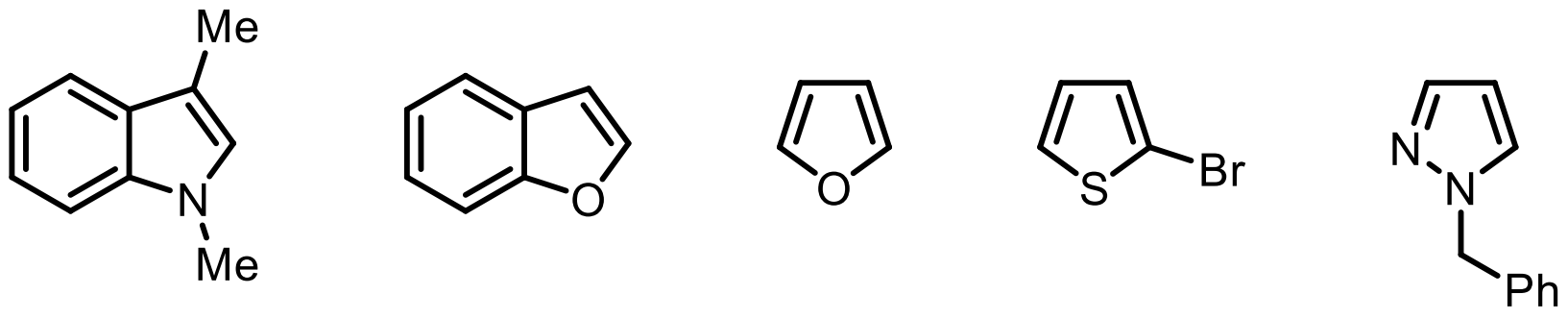

S22 **S23** **S24** **S25** **S26**

Figure S12. Electron-rich aromatic substrates unable to form the corresponding functionalised azlactone.

entry	SOMOphile	E_p (V)	electrocatalyst	$E_{1/2}$ (V)	ΔE	conv. (%)[a]	yield **4** (%)[a]
**1**	anisole (**S16**)	2.10	**3a**	1.09	1.01	89	-
**2**	1,4-dimethoxybenzene (**S17**)	1.53	**3a**	1.09	0.44	96	traces
**3**	1,4-dimethoxybenzene (**S17**)	1.53	**3b**	1.00	0.53	97	traces
**4**[b]	1,4-dimethoxybenzene (**S17**)	1.53	**3b**	1.00	0.53	>99	traces
**5**	1,3-benzodioxole (**S18**)	1.72	**3a**	1.09	0.63	72	-
**6**	1,3-benzodioxole (**S18**)	1.72	**3b**	1.00	0.72	69	-
**7**[b]	1,3-benzodioxole (**S18**)	1.72	**3a**	1.09	0.63	70	-
**8**	2-acetylpyrrole (**S19**)	1.89	**3a**	1.09	0.80	89	-
**9**	*N*-methylindole (**S20**)	0.97	**3e**	0.57	0.40	>99	-
**10**	*N*-methyl-2-phenylindole (**S21**)	0.93	**3e**	0.57	0.36	>99	-
**11**	*N*-methyl-3-methylindole (**S22**)	0.87	**3e**	0.57	0.30	>99	-
**12**	benzofuran (**S23**)	1.90	**3a**	1.09	0.81	98	-
**13**	furan (**S24**)	2.12	**3a**	1.09	1.03	99	-
**14**	2-bromothiophene (**S25**)	2.20	**3a**	1.09	1.11	>99	-
**15**	*N*-benzylpyrazole (**S26**)	2.18	**3a**	1.09	1.09	>99	-

Table S9. Evaluation of other electron-rich aromatic SOMOphiles under optimised standard conditions. [a] Yield and conversion were determined by ^{1}H NMR spectroscopy using 1,3,5-trimethoxybenzene as the internal standard. [b] Reaction run at 50 ºC.

The oxidation potential of anisole (**S16**), benzofuran (**S23**), furan (**S24**), 2-bromothiophene (**S25**), and *N*-benzylpyrazole (**S26**) is high enough to ensure selectivity towards the oxidation of the enolate by the corresponding electrocatalyst. However, its comparatively less electron-richness results in a suboptimal radical match with the electrophilic radical coming from the oxidation of the enolate, as well as a lower stability of the subsequent cyclohexadienyl radical intermediate.

In a similar manner 1,4-dimethoxybenzene (**S17**) and 1,3-benzodioxole (**S18**) reactive sites are comparatively less activated. Even if the electron-richness (Ep > 1.90 V) of the aromatic ring is similar to 1,3-dimethoxybenzene (**2a**), in the case of **S17** and **S18** only one methoxy group will activate each of the *ortho* positions of the substrate and stabilise the subsequent cyclohexadienyl radical, while 2 methoxy groups activate each reactive site in **2a** and stabilise the corresponding radical intermediate. Product formation is not observed even when heating up to 50 ºC.

In the case of 2-acetylpyrrole (**S19**), no product formation was observed even if its oxidation potential should also not interfere with the oxidation of the enolate. However, signs of pyrrole polymerisation were observed in the reaction crude, showing the necessity for protection of the nitrogen atom in pyrrole.

Regarding indoles **S20**, **S21**, and **S22**, the desired products were not formed because their oxidation potential difference with electrocatalyst was too low, leading to competitive oxidation of both the electron-rich SOMOphile and the azlactone enolate. All reactions were carried out using electrocatalyst **3e**, as it exhibits the lowest oxidation potential in the catalyst series and therefore affords the largest possible ΔE for each substrate, maximising the likelihood of satisfying the ΔE criterion for efficient electrocatalysis.

To further investigate the scope of the methodology, and in response to a reviewer's suggestion, electron-rich aliphatic olefins were tested as electron-rich coupling partners under the optimised electrocatalytic conditions (Figure S13).

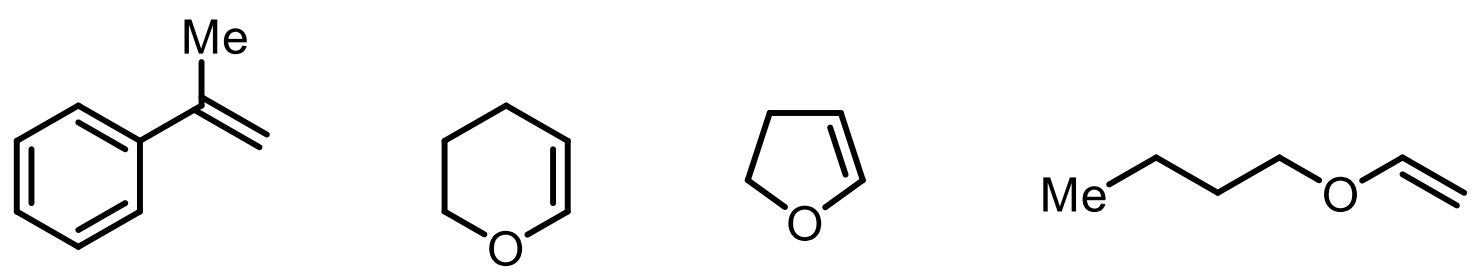

S27 **S28** **S29** **S30**

Figure S13. Electron-rich aliphatic olefins unable to form the corresponding functionalised azlactone.

entry	SOMOphile	E_p (V)	electrocatalyst	$E_{1/2}$ (V)	ΔE	conv. (%)[a]	yield (%)[a]
**1**	α-methylstyrene (**S27**)	1.95	**3a**	1.09	0.86	98	-
**2**	3,4-dihydro-2*H*-pyran (**S28**)	1.72	**3a**	1.09	0.63	66	-
**3**	2,3-dihydrofuran (**S29**)	1.52	**3b**	1.00	0.52	33	-
**4**	butyl vinyl ether (**S30**)	1.91	**3a**	1.09	0.82	95	-

Table S10. Evaluation of electron-rich aliphatic olefins under optimised standard conditions. [a] Yield and conversion were determined by ^{1}H NMR spectroscopy using 1,3,5-trimethoxybenzene as the internal standard.

Despite all selected olefins satisfied the electrochemical criterion of ΔE > 0.5 to ensure selectivity towards the oxidation of the enolate, none of them afforded the desired coupling product. In all cases, partial or complete consumption of azlactone **1f** was observed together with complete degradation of the three equivalents of the electron-rich alkene.

G. Large-scale electrochemical setup

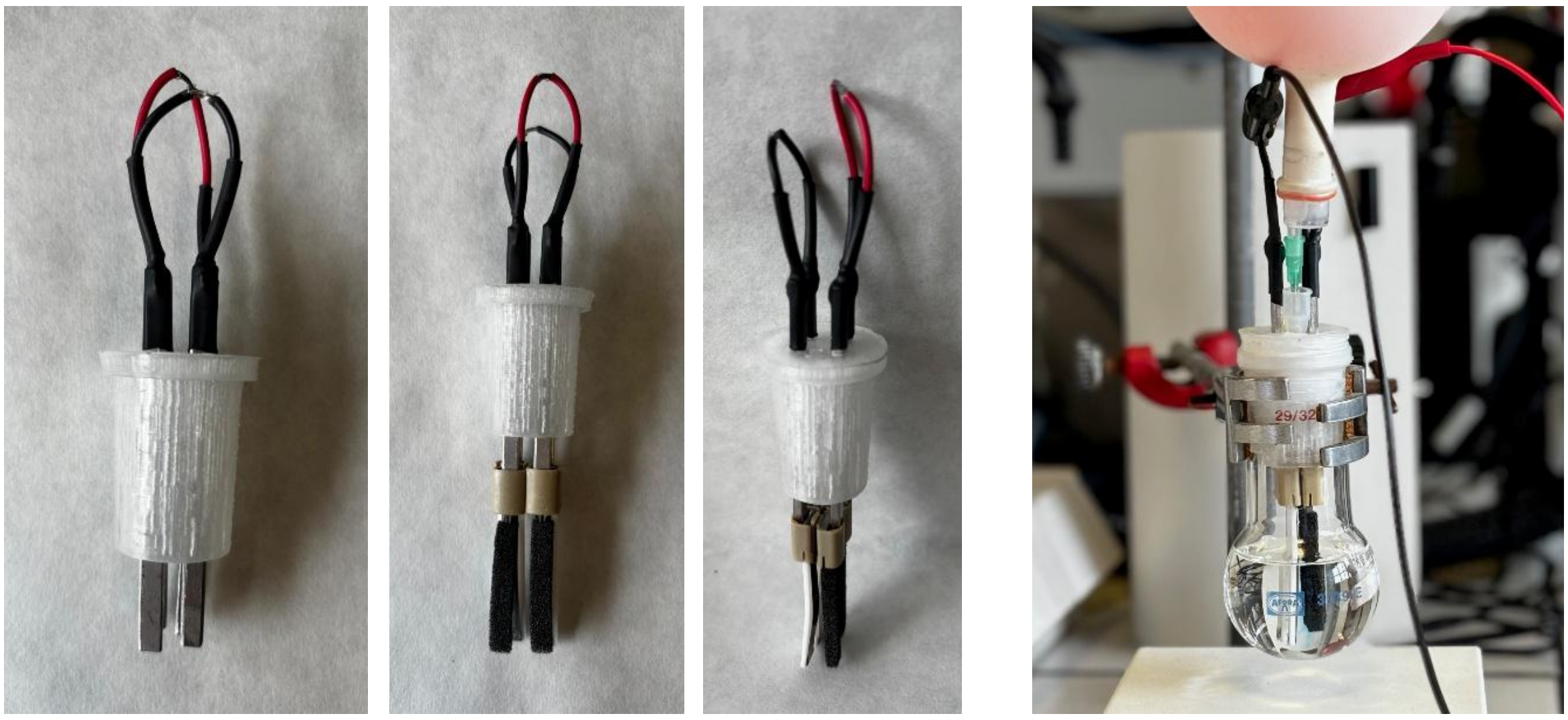

Figure S14. Experimental setup for the large-scale electrochemical reaction. In a 3D-printed cap (polypropylene), 4 stainless steel bars (80 mm x 5 mm x 3 mm) were inserted and connected two by two by a copper wire. Electrasyn electrodes (2 x RVC + 2 x Pt) were inserted into the underside of the lid.

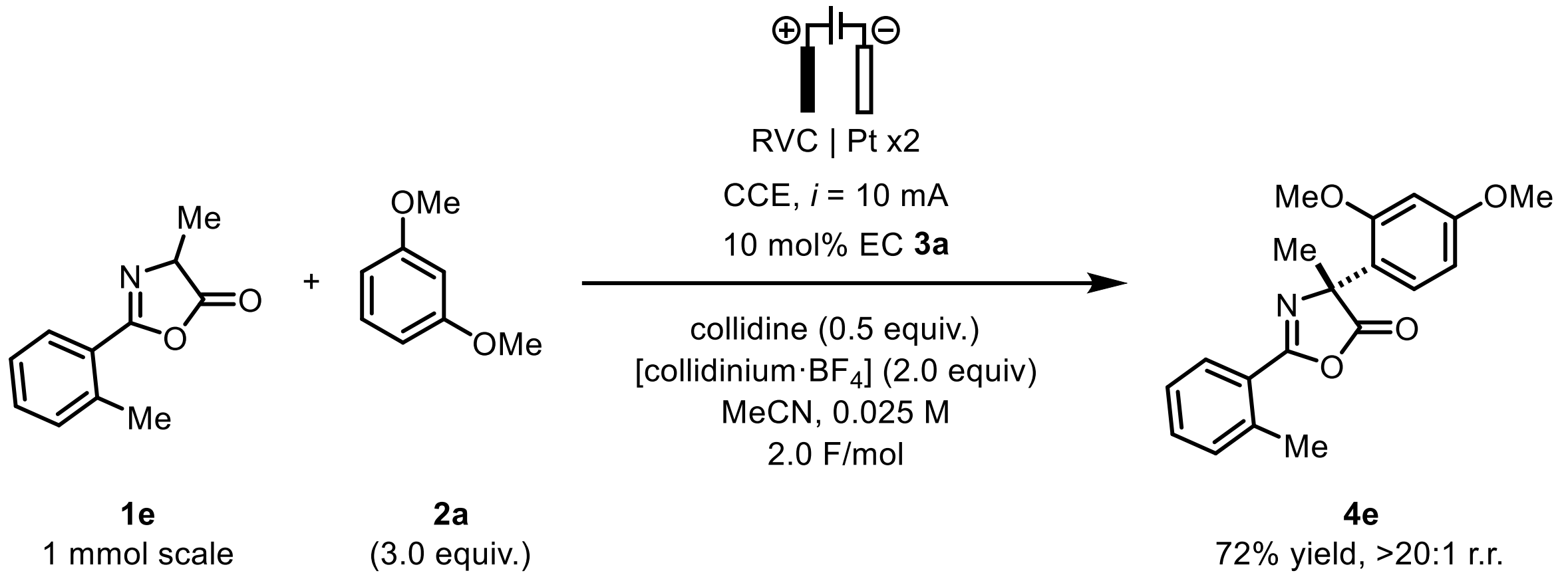

A 50 mL round bottom flask with a magnetic stirring bar was charged with azlactone **1e** (189 mg, 1 mmol, 1.0 equiv.), electrocatalyst **3a** (34.5 mg, 0.1 mmol, 10 mol%), and [collidinium·BF_4] (418 mg, 2 mmol, 2.0 equiv.). The 3D printed cap equipped with two anodes (RVC) and two cathodes (platinum foil) was inserted into the round bottom flask and the system was purged with N_2. Subsequently, MeCN (80 mL, 0.025 M), 1,3-dimethoxybenzene (**2a**, 392 µL, 3 mmol, 3.0 equiv.), and collidine (66 µL, 0.5 mmol, 0.5 equiv.) were added. The resulting mixture was stirred and electrolysed at constant current (10 mA) for 6 h. Then the electrodes were rinsed with CH_2Cl_2, and the crude mixture was directly purified by flash column chromatography on silica gel (hexanes/ethyl acetate = 5:1 to 4:1) to yield the desired product **4e** (234 mg, 72% yield).

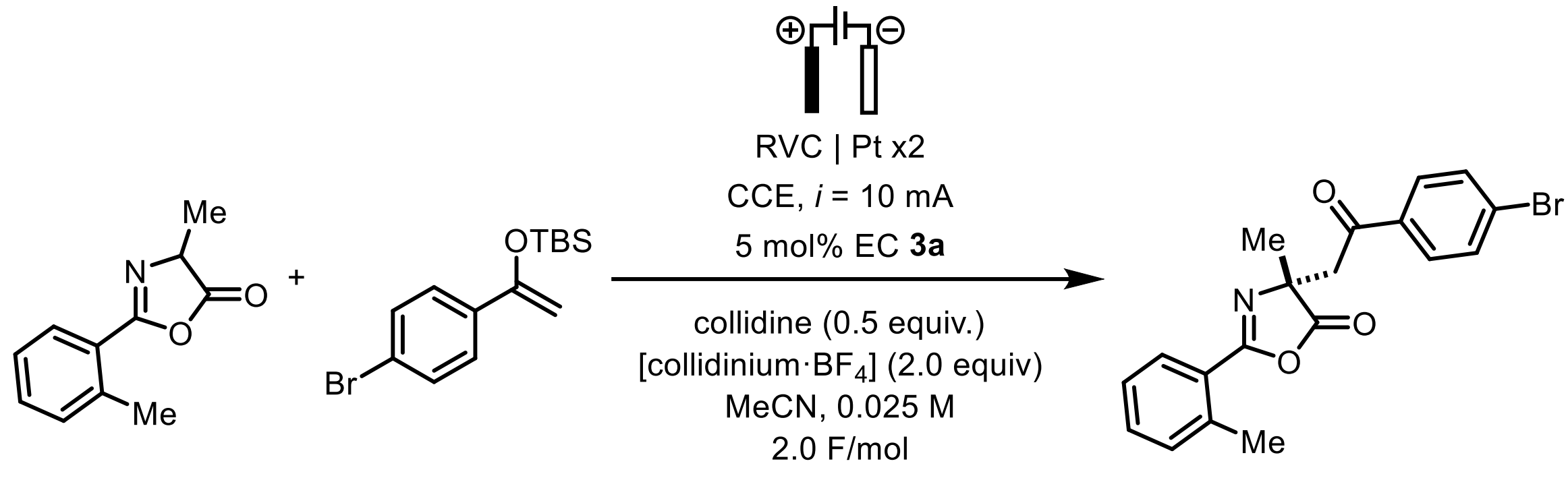

A 50 mL round bottom flask with a magnetic stirring bar was charged with azlactone **1e** (189 mg, 1 mmol, 1.0 equiv.), ((1-(4-bromophenyl)vinyl)oxy)(tert-butyl)dimethylsilane (**9b**, 939 mg, 3.0 mmol, 3.0 equiv.), electrocatalyst **3b** (18.5 mg, 0.05 mmol, 5 mol%), and [collidinium·BF_4] (418 mg, 2 mmol, 2.0 equiv.). The 3D printed cap equipped with two anodes (RVC) and two cathodes (platinum foil) was inserted into the round bottom flask and the system was purged with N_2. Subsequently, MeCN (80 mL, 0.025 M), and collidine (66 µL, 0.5 mmol, 0.5 equiv.) were added. The resulting mixture was stirred and electrolysed at constant current (10 mA) for 7 h. Then the electrodes were rinsed with CH_2Cl_2, and the crude mixture was directly purified by flash column chromatography on silica gel (hexanes/ethyl acetate = 90:10) to yield the desired product **10b** (267 mg, 70% yield).

H. Alternative electrochemical setups

H.1. Galvanostat reaction with Electrasyn vial and electrodes

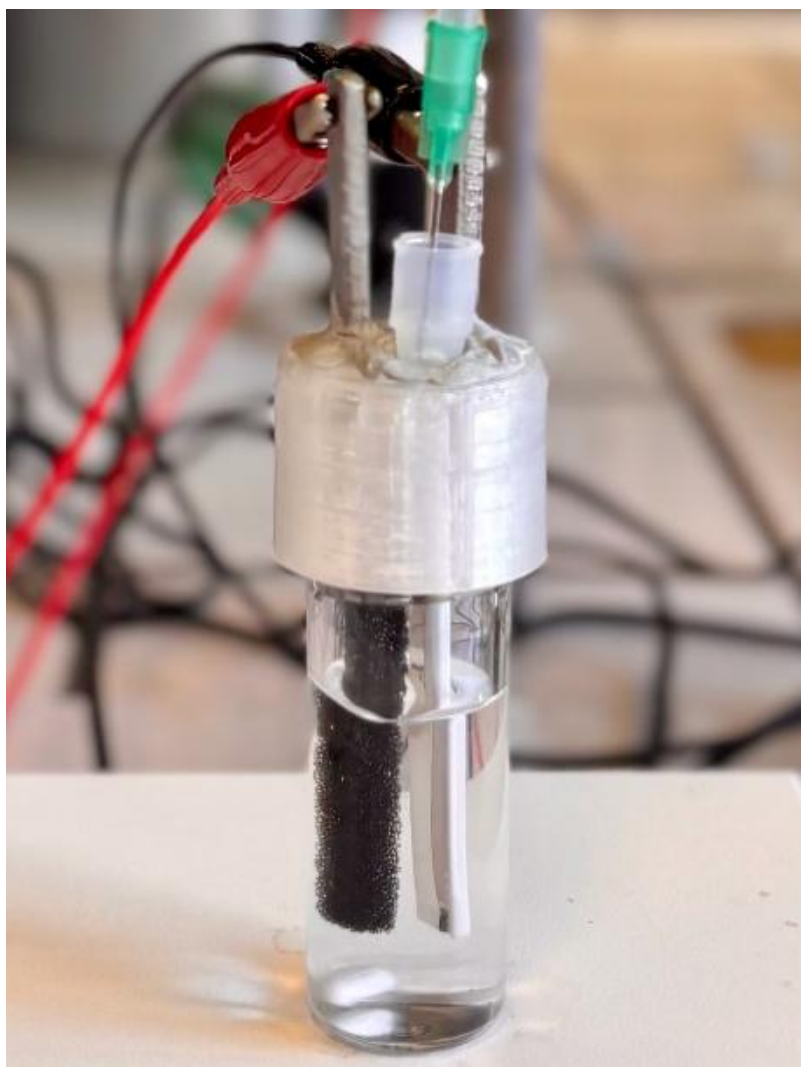

Figure S15. Experimental setup for the electrochemical galvanostat reaction with an Electrasyn vial and electrodes. Details for the 3D-print design of the vial cap have been previously reported.[29]

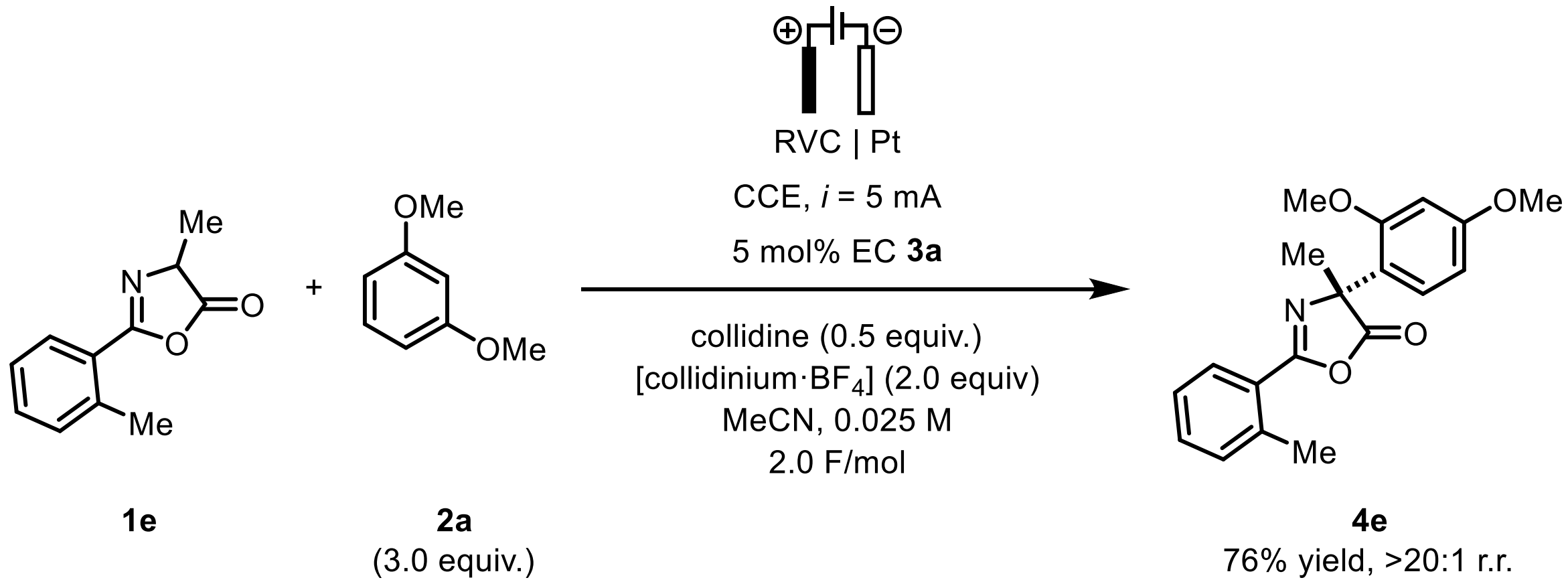

A 10 mL ElectraSyn vial with a magnetic stirring bar was charged with azlactone **1e** (37.8 mg, 0.2 mmol, 1.0 equiv.), electrocatalyst **3a** (3.5 mg, 0.01 mmol, 5 mol%), and [collidinium·BF_4] (83.6 mg, 0.4 mmol, 2.0 equiv.). The 3D-printed vial cap equipped with anode (RVC) and cathode (platinum foil) was inserted and the system was purged with N_2. Subsequently, MeCN (8 mL, 0.025 M), 1,3-dimethoxybenzene (**2a**, 79 µL, 0.6 mmol, 3.0 equiv.), and collidine (13 µL, 0.1 mmol, 0.5 equiv.) were added. The resulting mixture was stirred and electrolysed at constant current (5 mA) for 2.5 h. Then the electrodes were rinsed with CH_2Cl_2, and the crude mixture was directly purified by flash column chromatography on silica gel (hexanes/ethyl acetate = 5:1 to 4:1) to yield the desired product **4e** (49 mg, 76% yield).

H.2. Galvanostat reaction with non-commercial cell and electrodes

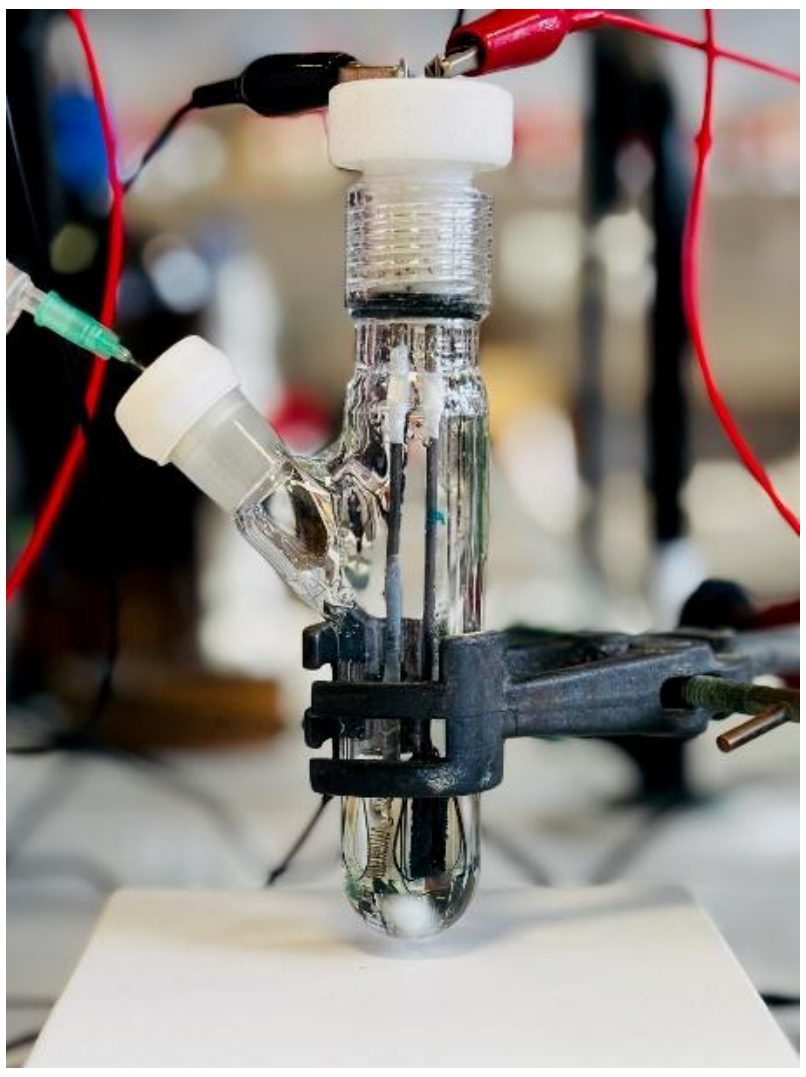

Figure S16. Experimental setup for the electrochemical galvanostat reaction with handmade cell and electrodes. Details for the preparation of the cell, the cap, and the electrodes have been previously reported.[30]

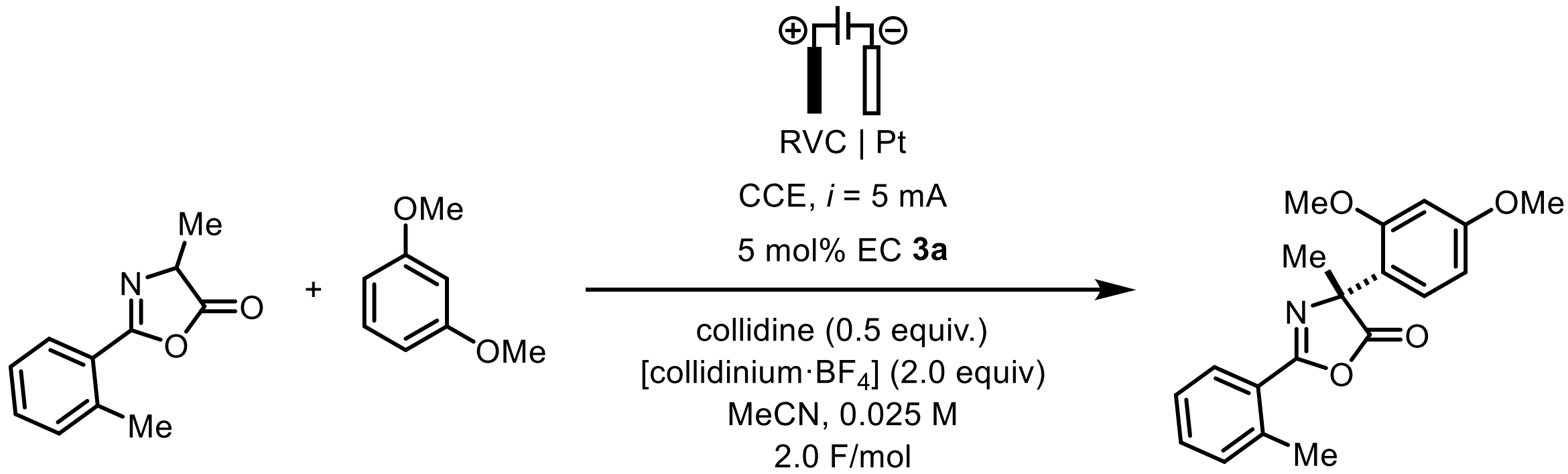

The electrochemical cell with a magnetic stirring bar was charged with azlactone **1e** (37.8 mg, 0.2 mmol, 1.0 equiv.), electrocatalyst **3a** (3.5 mg, 0.01 mmol, 5 mol%), and [collidinium·BF_4] (83.6 mg, 0.4 mmol, 2.0 equiv.). The cell was closed with the corresponding cap with the anode (RVC) and cathode (platinum wire) and the system was purged with N_2. Subsequently, MeCN (8 mL, 0.025 M), 1,3-dimethoxybenzene (**2a**, 79 µL, 0.6 mmol, 3.0 equiv.), and collidine (13 µL, 0.1 mmol, 0.5 equiv.) were added. The resulting mixture was stirred and electrolysed at constant current (5 mA) for 2.5 h. Then the electrodes were rinsed with CH_2Cl_2, and the crude mixture was directly purified by flash column chromatography on silica gel (hexanes/ethyl acetate = 5:1 to 4:1) to yield the desired product **4e** (47 mg, 73% yield).

I. Derivatisations

I.1. N-benzoylated amino ester formation

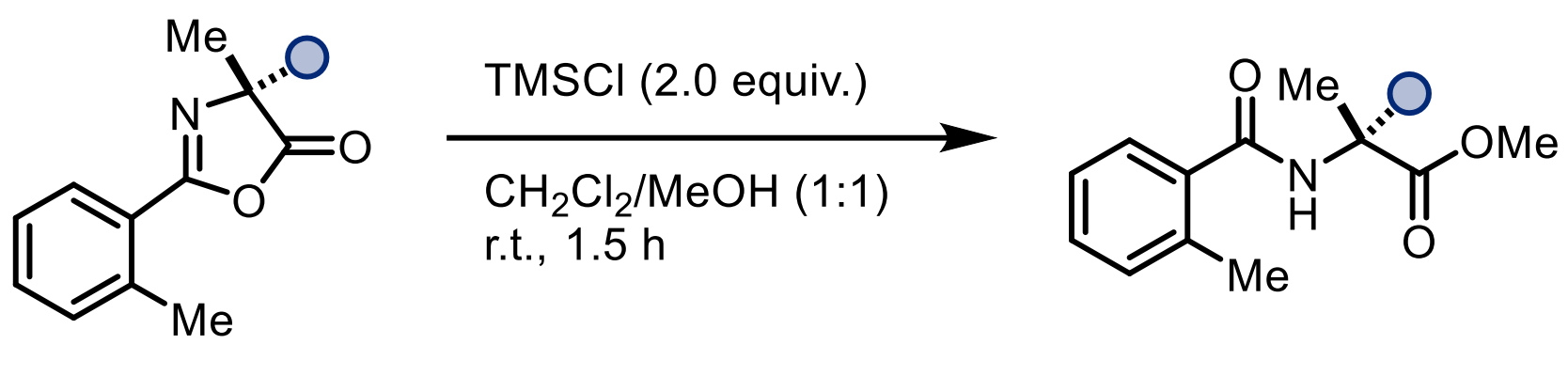

4e *or* **10b** **15** *or* **21**

The reaction was carried out following the previously described procedure[31]

The corresponding azlactone (0.2 mmol) was dissolved in a mixture of CH_2Cl_2/MeOH (1:1) (0.25 M), then chlorotrimethylsilane (2.0 equiv.) was added and left reacting for 1.5 h. The crude product was purified by silica gel chromatography (hexanes/EtOAc, 3:7) to afford the corresponding desired product.

methyl 2-(2,4-dimethoxyphenyl)-2-(2-methylbenzamido)propanoate (15)

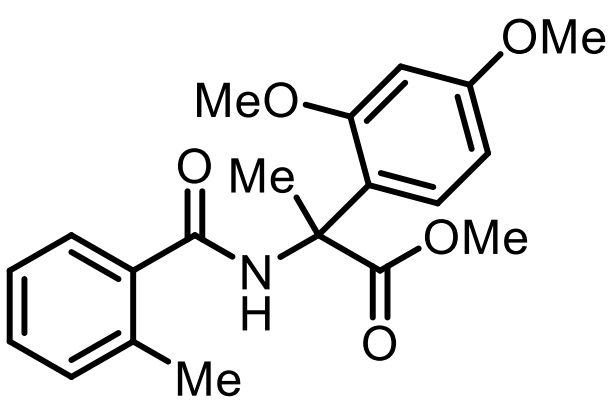

56 mg (0.16 mmol, 79 % yield), white solid. **^{1}H NMR** (400 MHz, $CDCl_3$) δ 7.58 (d, *J* = 8.6 Hz, 1H), 7.42 (s, 1H), 7.33 (dd, *J* = 7.8, 1.5 Hz, 1H), 7.29 – 7.23 (m, 1H), 7.20 – 7.12 (m, 2H), 6.55 (dd, *J* = 8.6, 2.5 Hz, 1H), 6.44 (d, *J* = 2.5 Hz, 1H), 3.81 (s, 3H), 3.76 (s, 3H), 3.72 (s, 3H), 2.34 (s, 3H), 2.14 (s, 3H) ppm; **^{13}C NMR** (101 MHz, $CDCl_3$) δ 175.8, 168.2, 160.7, 157.3, 137.1, 136.1, 131.0, 129.7, 129.5, 127.2, 125.7, 121.1, 103.7, 99.1, 55.4, 55.4, 53.1, 22.2, 19.8 ppm; **HRMS (ESI)** Calculated for $C_{20}H_{23}NO_5^+$ ($[M+H]^+$) 358.1649. Found 358.1660.

methyl 4-(4-bromophenyl)-2-methyl-2-(2-methylbenzamido)-4-oxobutanoate (21)

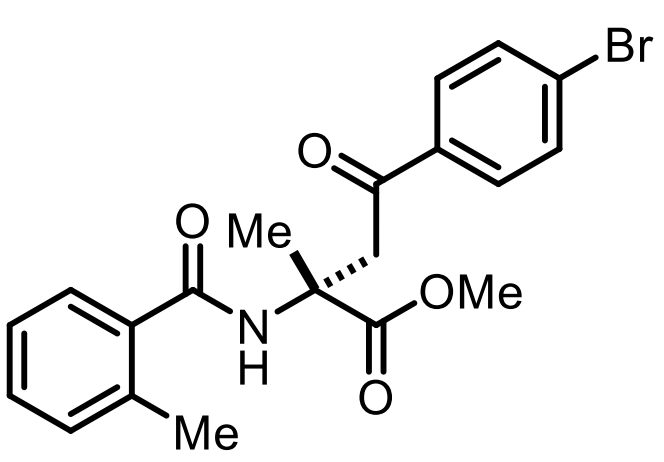

69 mg (0.17 mmol, 83 % yield), white solid, mixture of rotamers (2.8:1). **^{1}H NMR** (400 MHz, $CDCl_3$) δ (for major rotamer) 7.59 – 7.53 (m, 2H), 7.51 – 7.46 (m, 2H), 7.34 – 7.16 (m, 2H), 7.22 – 7.13 (m, 2H), 6.00 (s, 1H), 3.25 (s, 3H), 2.95 (d, *J* = 13.2 Hz, 1H), 2.66 (d, *J* = 13.2 Hz, 1H), 2.41 (s, 3H), 1.77 (s, 3H) ppm; δ (for minor rotamer) 7.87 – 7.80 (m, 2H), 7.62 – 7.58 (m, 2H), 7.34 – 7.16 (m, 2H), 7.22 – 7.13 (m, 2H), 7.10 (s, 1H), 4.72 (d, *J* = 17.9 Hz, 1H), 3.81 (s, 3H), 3.52 (d, *J* = 17.9 Hz, 1H), 2.30 (s, 3H), 1.85 (s, 3H) ppm; **^{13}C NMR (101 MHz, $CDCl_3$)** δ (for major rotamer) 196.9, 176.5, 169.6, 137.7, 136.6, 134.8, 132.1, 131.1, 130.4, 129.8, 127.7, 126.8, 125.7, 57.8, 51.8, 48.2, 24.4, 19.7 ppm; δ (for minor rotamer) 196.9, 174.7, 169.5, 136.4, 135.9, 135.2, 132.1, 131.0, 130.1, 127.0, 125.9, 123.2, 107.1, 58.2, 53.2, 43.9, 23.9, 19.7 ppm. **HRMS (ESI)** Calculated for $[C_{20}H_{21}NO_4Br]^+$ ($[M+H]^+$) 418.0648. Found 418.0666.

I.2. N-benzoylated amino acid formation

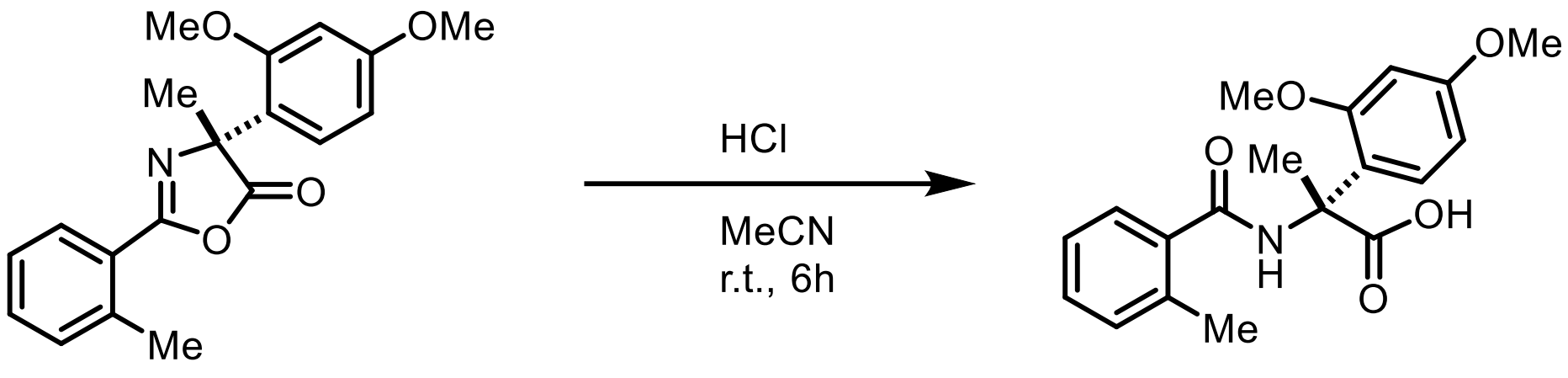

4e **16**

The reaction was carried out following the previously described procedure[32]

A vial containing a magnetic stir bar was charged with azlactone **4e** (0.2 mmol) and acetonitrile (0.4 M). Concentrated HCl (50 µL) was added, and the mixture was stirred at room temperature for 6 h. The solvent was removed under reduced pressure, and the crude residue was dissolved in 1 mL of an aqueous solution of 1M NaOH (pH = 12) and washed twice with CH_2Cl_2. Then, 1M HCl was added until pH = 3. The resulting acidic aqueous phase was extracted three times with CH_2Cl_2. The organic phase was dried over $MgSO_4$, filtered and concentrated under vacuum to afford **16**.

2-(2,4-dimethoxyphenyl)-2-(2-methylbenzamido)propanoic acid (16)

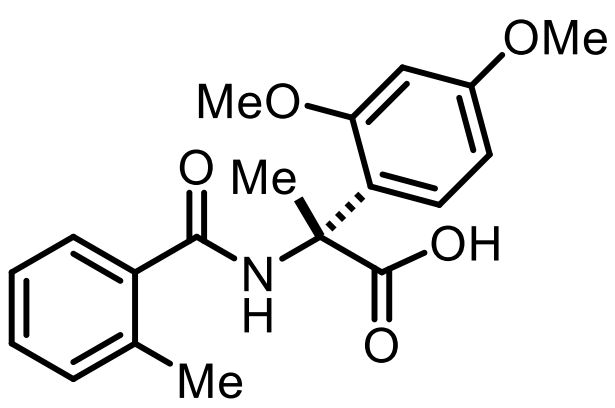

69 mg (0.2 mmol, >99 % yield), white solid. **^{1}H NMR** (400 MHz, DMSO) δ 12.95 (bs, 1H), 7.62 (s, 1H), 7.36 (d, J = 8.5 Hz, 1H), 7.34 – 7.28 (m, 1H), 7.27 – 7.19 (m, 3H), 6.56 (d, J = 2.5 Hz, 1H), 6.52 (dd, J = 8.5, 2.5 Hz, 1H), 3.76 (s, 3H), 3.74 (s, 3H), 2.24 (s, 3H), 1.97 (s, 3H) ppm; **^{13}C NMR** (101 MHz, DMSO) δ 175.3, 167.1, 159.9, 157.6, 137.3, 135.0, 130.6, 129.4, 128.7, 126.5, 125.7, 121.4, 103.9, 98.8, 59.4, 55.5, 55.2, 22.1, 19.1 ppm; **HRMS (ESI)** Calculated for $C_{19}H_{21}NO_5^+$ ([M+H]$^+$) 344.1492. Found 344.1504.

I.3. N-benzoylated amino alcohol formation

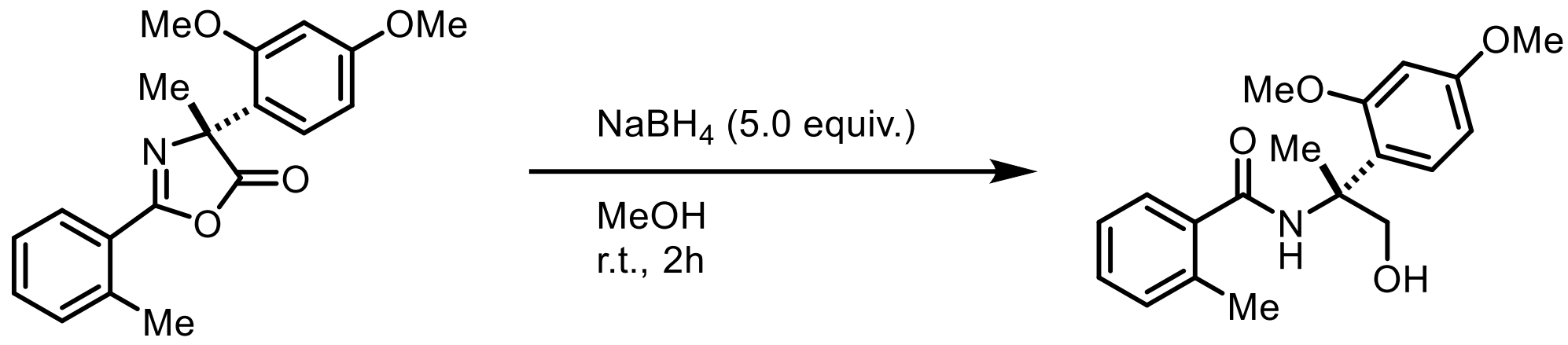

4e **17**

The reaction was carried out following the previously described procedure[32]

A vial equipped with a magnetic stir bar was charged with azlactone **4e** (0.2 mmol) and MeOH (0.5 M). Sodium borohydride (5.0 equiv.) was added in one portion, and the mixture was stirred at room temperature for 2 h. The reaction was then diluted with CH_2Cl_2 (5 mL) and washed with saturated aqueous $NaHCO_3$ solution (2 x 5 mL). The organic layer was dried over anhydrous $MgSO_4$, filtered, and concentrated under reduced pressure. The crude residue was purified by silica gel chromatography (hexanes/EtOAc, 50:50) to afford **17** as white solid.

***N*-(2-(2,4-dimethoxyphenyl)-1-hydroxypropan-2-yl)-2-methylbenzamide (17)**

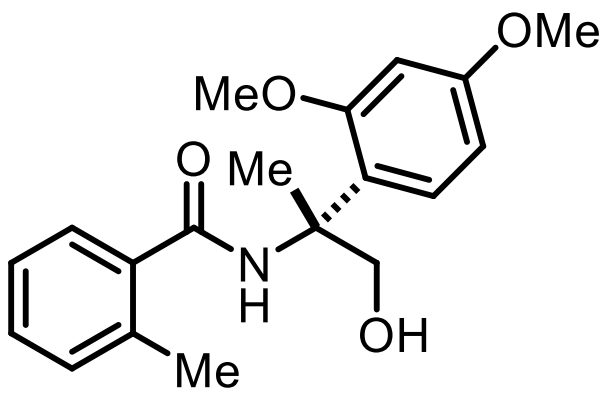

52 mg (0.16 mmol, 79 % yield), white solid. **^{1}H NMR** (400 MHz, DMSO) δ 7.82 (s, 1H), 7.40 – 7.27 (m, 2H), 7.27 – 7.17 (m, 3H), 6.55 (d, *J* = 2.5 Hz, 1H), 6.49 (dd, *J* = 8.6, 2.5 Hz, 1H), 4.92 (t, *J* = 6.3 Hz, 1H), 3.94 (dd, *J* = 11.1, 6.6 Hz, 1H), 3.78 (s, 3H), 3.75 (d, *J* = 1.2 Hz, 3H), 3.62 (dd, *J* = 11.0, 6.2 Hz, 1H), 2.33 (s, 3H), 1.71 (s, 3H).ppm; **^{13}C NMR** (101 MHz, DMSO) δ 168.5, 159.3, 157.9, 138.2, 134.9, 130.3, 128.9, 128.7, 126.9, 125.4, 123.0, 104.1, 99.2, 66.7, 60.0, 55.3, 55.1, 22.2, 19.2 ppm; **HRMS (ESI)** Calculated for $C_{19}H_{23}NO_4^+$ ($[M+H]^+$) 330.1700. Found 330.1711.

I.5. Amide formation with aniline

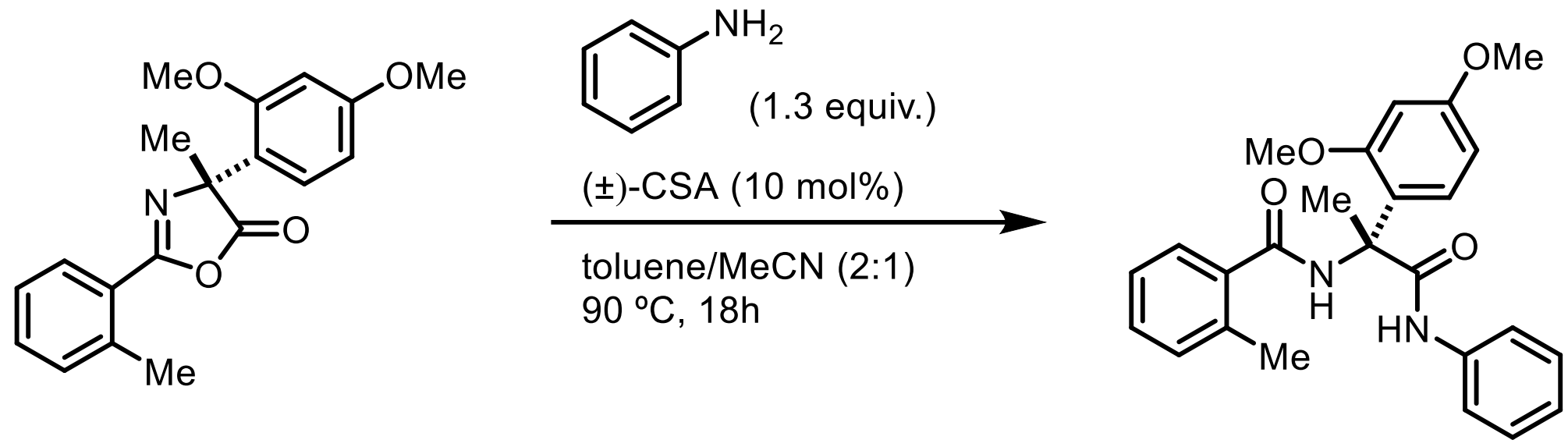

4e **18**

The reaction was carried out following the modified previously described procedure[33]

A vial was charged with aniline (1.3 equiv.) and a 2:1 mixture of toluene/acetonitrile (0.17 M). To this solution were added the azlactone **4e** (0.2 mmol) and (±)-camphorsulfonic acid (10 mol%). The reaction mixture was heated at 90 °C and stirred during 18 h. After cooling down to room temperature, the mixture was diluted with CH_2Cl_2 (10 mL) and washed with water (3 x 5 mL). The organic layer was dried over anhydrous Na_2SO_4, filtered, and concentrated under reduced pressure to obtain pure product **18** without further purification.

_N_-(2-(2,4-dimethoxyphenyl)-1-oxo-1-(phenylamino)propan-2-yl)-2-methylbenzamide (18)

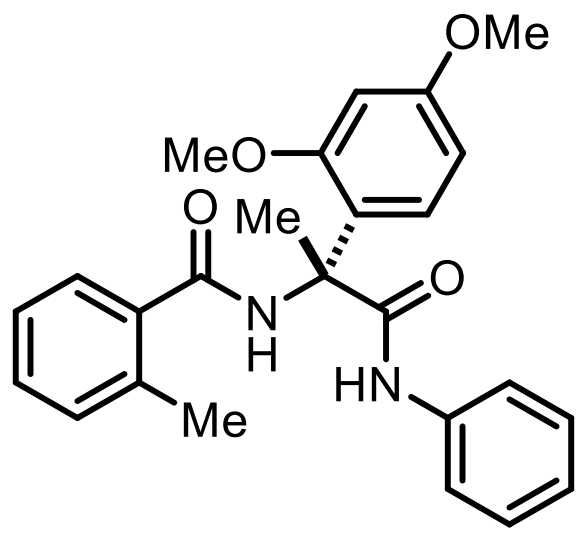

74 mg (0.18 mmol, 88 % yield), white solid. **^{1}H NMR** (400 MHz, $CDCl_3$) δ 7.66 (d, *J* = 8.6 Hz, 1H), 7.58 (s, 1H), 7.42 – 7.34 (m, 4H), 7.33 – 7.25 (m, 3H), 7.20 – 7.14 (m, 2H), 7.13 – 7.06 (m, 1H), 6.61 (dd, *J* = 8.6, 2.5 Hz, 1H), 6.48 (d, *J* = 2.4 Hz, 1H), 3.84 (s, 3H), 3.74 (s, 3H), 2.39 (s, 3H), 2.18 (s, 3H) ppm; **^{13}C NMR** (101 MHz, $CDCl_3$) δ 173.2, 168.4, 161.4, 157.9, 137.7, 136.9, 136.3, 131.1, 130.1, 129.8, 129.1, 127.3, 125.8, 124.7, 120.3, 104.3, 99.7, 61.1, 55.7, 55.5, 29.9, 23.6, 20.0 ppm; **HRMS (ESI)** Calculated for $C_{25}H_{27}N_2O_4^+$ ($[M+H]^+$) 419.1965. Found 419.1980.

I.4. Dipeptide formation with glycine

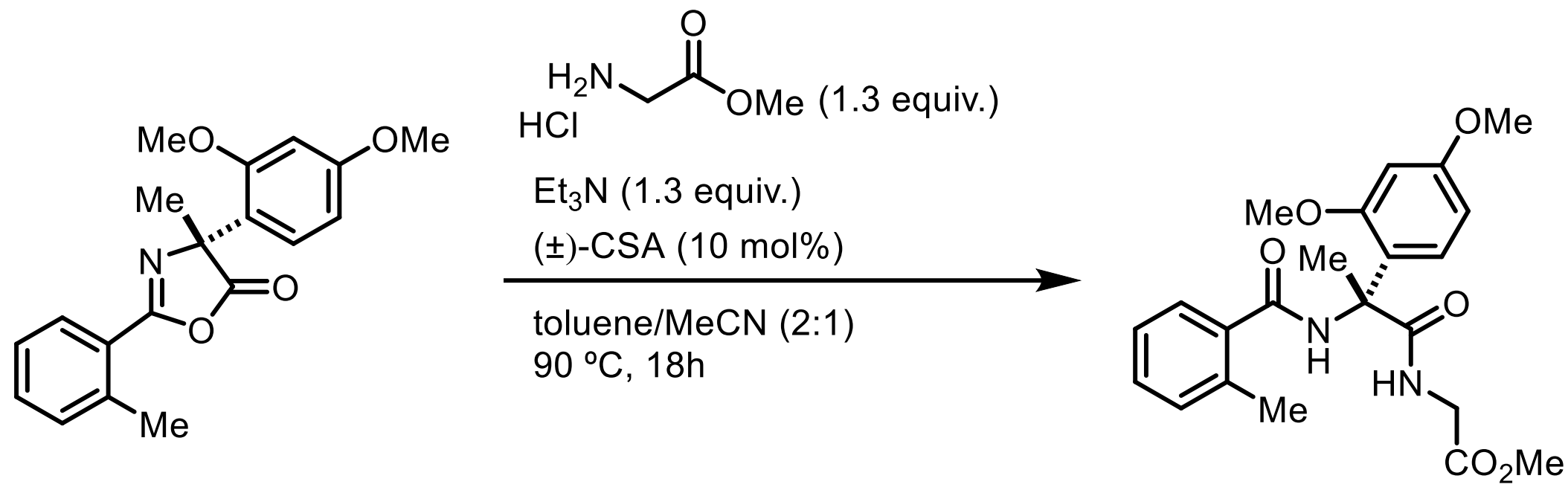

The reaction was carried out following the modified previously described procedure[33]

A vial was charged with glycine methyl ester hydrochloride (1.3 equiv.) and a 2:1 mixture of toluene/acetonitrile (0.17 M). Then, triethylamine (1.3 equiv.) was added, and the mixture was stirred at room temperature for 10 min. To this solution were added the azlactone **4e** (0.2 mmol) and (±)-camphorsulfonic acid (10 mol%). The reaction mixture was heated at 90 °C and stirred during 18 h. After cooling down to room temperature, the mixture was diluted with CH_2Cl_2 (10 mL) and washed with water (3 x 5 mL). The organic layer was dried over anhydrous Na_2SO_4, filtered, and concentrated under reduced pressure. The crude product was purified by silica gel chromatography (hexanes/EtOAc, 60:40 to 40:60) to afford **19** as a white solid.

methyl (2-(2,4-dimethoxyphenyl)-2-(2-methylbenzamido)propanoyl)glycinate (19)

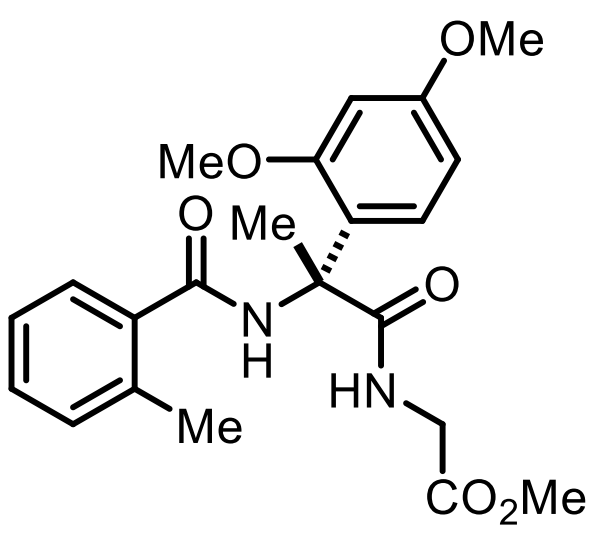

69 mg (0.17 mmol, 83 % yield), white solid. **^{1}H NMR** (400 MHz, $CDCl_3$) δ 7.64 – 7.56 (m, 2H), 7.35 (dd, *J* = 7.8, 1.5 Hz, 1H), 7.25 (td, *J* = 7.5, 1.5 Hz, 1H), 7.18 – 7.12 (m, 2H), 6.56 (dd, *J* = 8.6, 2.5 Hz, 1H), 6.46 (d, *J* = 2.5 Hz, 1H), 5.93 (t, *J* = 5.4 Hz, 1H), 3.99 (dd, *J* = 5.5, 4.1 Hz, 2H), 3.82 (s, 3H), 3.76 (s, 3H), 3.71 (s, 3H), 2.36 (s, 3H), 2.12 (s, 3H) ppm; **^{13}C NMR** (101 MHz, $CDCl_3$) δ 175.6, 170.0, 168.1, 161.3, 157.9, 137.1, 136.2, 130.9, 130.0, 129.7, 127.3, 125.7, 120.3, 104.0, 99.5, 60.3, 55.5, 55.4, 52.4, 41.5, 23.7, 19.9 ppm; **HRMS (ESI)** Calculated for $C_{22}H_{26}N_2O_6^+$ ($[M+H]^+$) 415.1864. Found 415.1881.

I.6. Benzothiazole formation with o*-aminothiophenol*

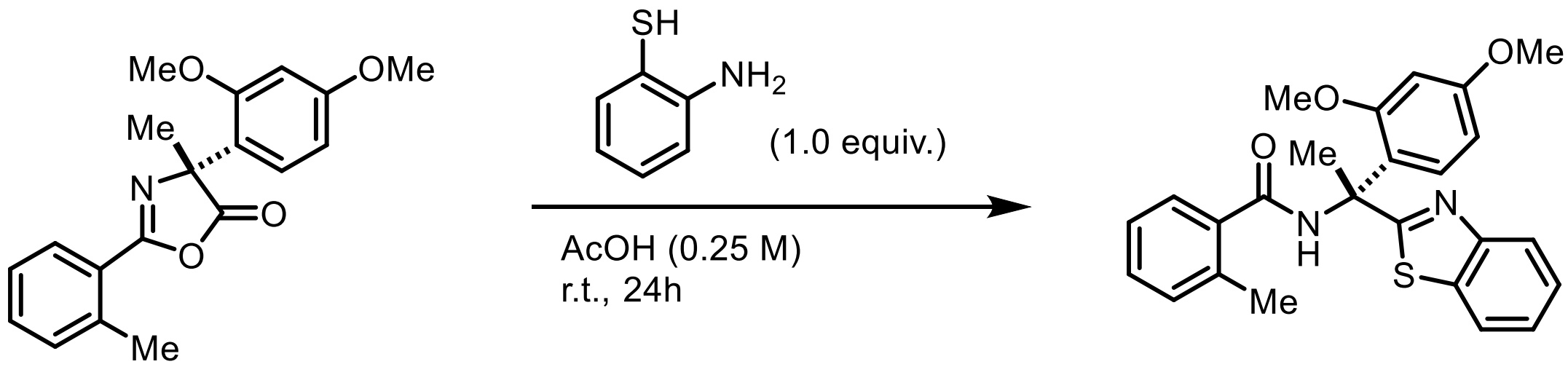

The reaction was carried out following the modified previously described procedure[34]

Azlactone **4e** (0.2 mmol) was dissolved in acetic acid (0.25 M) and 2-aminophenol (1.0 equiv.) was added. The reaction mixture was left stirring during 24h at room temperature. Then, the crude mixture was partitioned in CH_2Cl_2/H_2O and the organic phase was separated, dried over $MgSO_4$, filtered, and concentrated under vacuum. Pure product **20** was obtained upon silica gel chromatography (hexanes/EtOAc, 2:1 to 2:3).

_N_-(1-(benzo[_d_]thiazol-2-yl)-1-(2,4-dimethoxyphenyl)ethyl)-2-methylbenzamide (20)

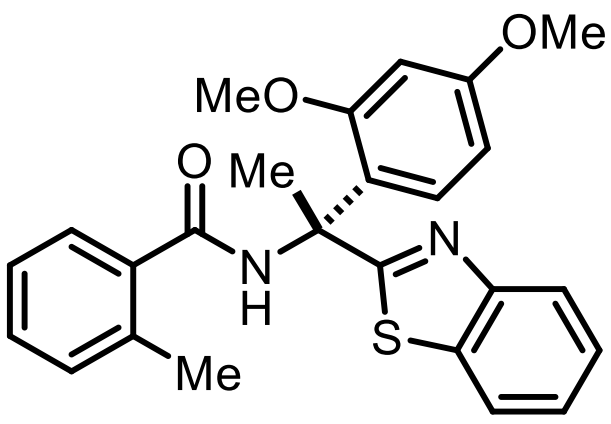

68 mg (0.16 mmol, 79 % yield), white solid. **^{1}H NMR** (400 MHz, $CDCl_3$) δ 8.10 (s, 1H), 7.97 (ddd, *J* = 8.2, 1.2, 0.6 Hz, 1H), 7.76 (ddd, *J* = 8.0, 1.3, 0.6 Hz, 1H), 7.57 (d, *J* = 8.6 Hz, 1H), 7.50 – 7.43 (m, 2H), 7.37 – 7.28 (m, 2H), 7.25 – 7.19 (m, 2H), 6.57 (dd, *J* = 8.7, 2.5 Hz, 1H), 6.43 (d, *J* = 2.5 Hz, 1H), 3.83 (s, 3H), 3.53 (s, 3H), 2.43 (s, 3H), 2.37 (s, 3H) ppm; **^{13}C NMR** (101 MHz, $CDCl_3$) δ 179.4, 168.4, 161.2, 158.4, 152.2, 137.4, 136.6, 135.6, 131.0, 129.7, 129.4, 127.2, 126.0, 125.7, 125.1, 123.6, 123.0, 121.8, 103.9, 99.8, 60.7, 55.4, 31.1, 27.7, 19.9 ppm; **HRMS (ESI)** Calculated for $C_{25}H_{24}N_2O_3S^+$ ($[M+H]^+$) 433.1580. Found 433.1599.

I.7. Amino acid formation

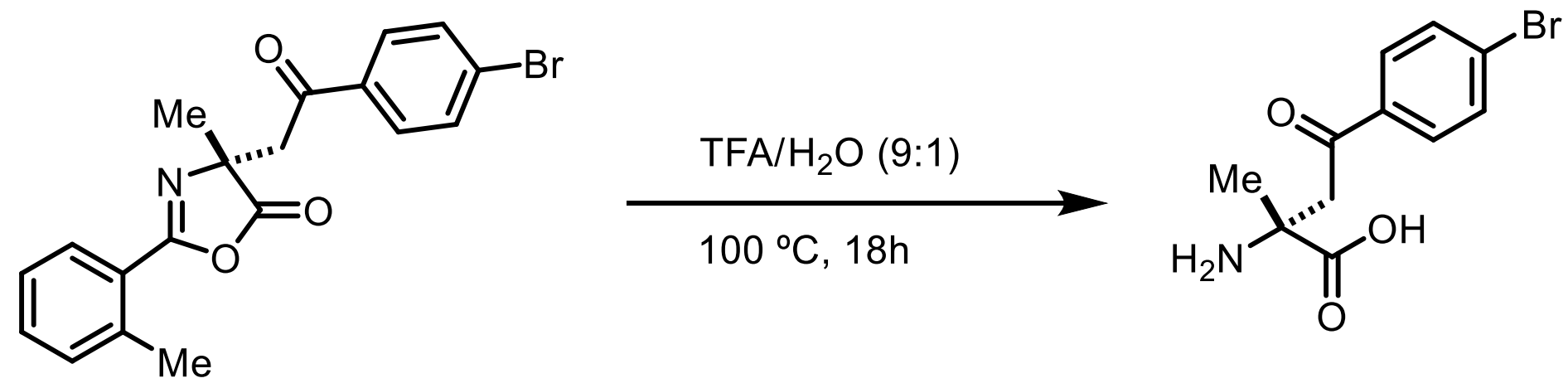

10b **22**

The reaction was carried out following the previously described procedure[35]

Azlactone **10b** (0.6 mmol) was dissolved in TFA/water (90:10; 0.05 M) in a sealed tube, and the solution was heated at 100 °C for 18 h. When the reaction was complete, the solvent was evaporated in vacuo. Water was added to the residue, and the mixture was washed three times with CH_2Cl_2. The aqueous phase was evaporated in vacuo to afford **22** as a white solid.

2-amino-4-(4-bromophenyl)-2-methyl-4-oxobutanoic acid (22)

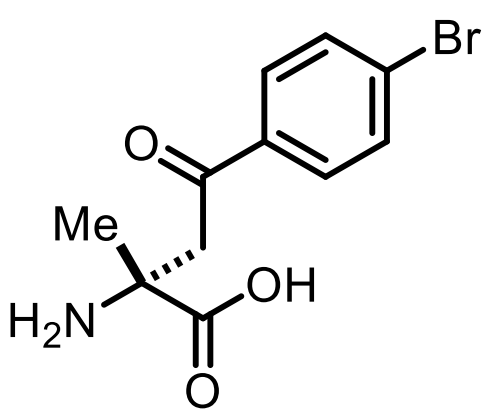

171.60 mg (0.6 mmol, >99 % yield), white solid. **^{1}H NMR** (400 MHz, DMSO) δ 7.89 (d, *J* = 8.4 Hz, 2H), 7.75 (d, *J* = 8.4 Hz, 2H), 3.66 (d, *J* = 18.0 Hz, 1H), 3.27 (d, *J* = 18.0 Hz, 1H), 1.38 (s, 3H) ppm; **^{13}C NMR** (101 MHz, DMSO) δ 197.4, 171.8, 135.2, 131.9, 130.0, 127.8, 57.2, 44.4, 23.7 ppm; **HRMS (ESI)** Calculated for $C_{11}H_{13}NO_4Br^+$ ($[M+H]^+$) 286.0073. Found 286.0065.

I.8. Amino ester formation

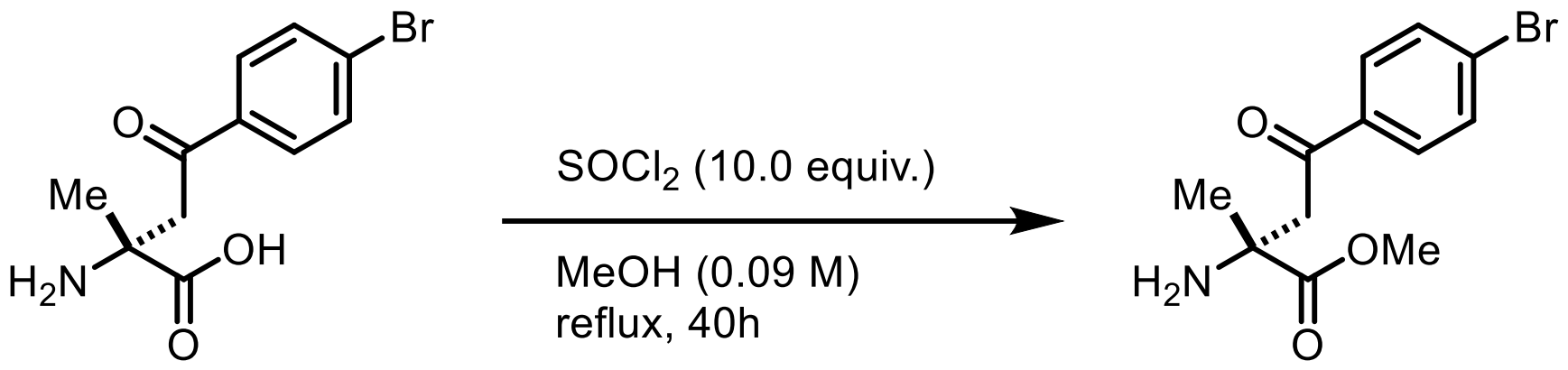

22 **23**

The reaction was carried out following the previously described procedure.[36]

To a solution of amino acid **22** (0.5 mmol) in MeOH (0.09 M), $SOCl_2$ (10.0 equiv.) was added dropwise at rt. Then the reaction mixture heated under reflux for 40 h. After cooling down to room temperature, the solvent was removed in vacuo to afford **23** as a white solid without further purification.

2-amino-4-(4-bromophenyl)-2-methyl-4-oxobutanoic acid (23)

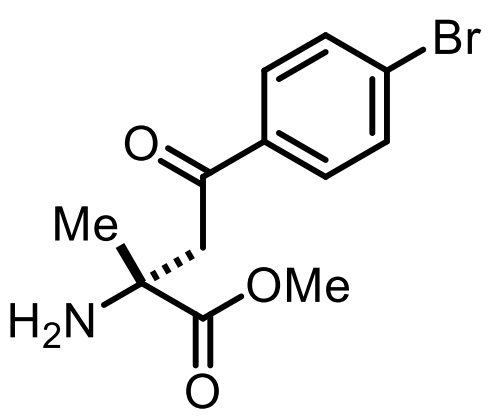

150 mg (0.5 mmol, >99 % yield), white solid. **^{1}H NMR** (400 MHz, MeOD) δ 7.96 (d, J = 7.7 Hz, 2H), 7.73 (d, J = 7.1 Hz, 2H), 4.03 (d, J = 18.5 Hz, 1H), 3.81 (s, 3H), 3.71 (d, J = 18.5 Hz, 1H), 1.69 (s, 3H) ppm; **^{13}C NMR** (101 MHz, MeOD) δ 197.5, 172.5, 135.4, 133.4, 131.2, 130.5, 58.1, 54.1, 46.1, 23.7 ppm; **HRMS (ESI)** Calculated for $[C_{12}H_{15}NO_3Br]^+$ ($[M+H]^+$) 300.0230. Found 300.0225.

J. Mechanistic considerations

J.1. Spectroelectrochemistry

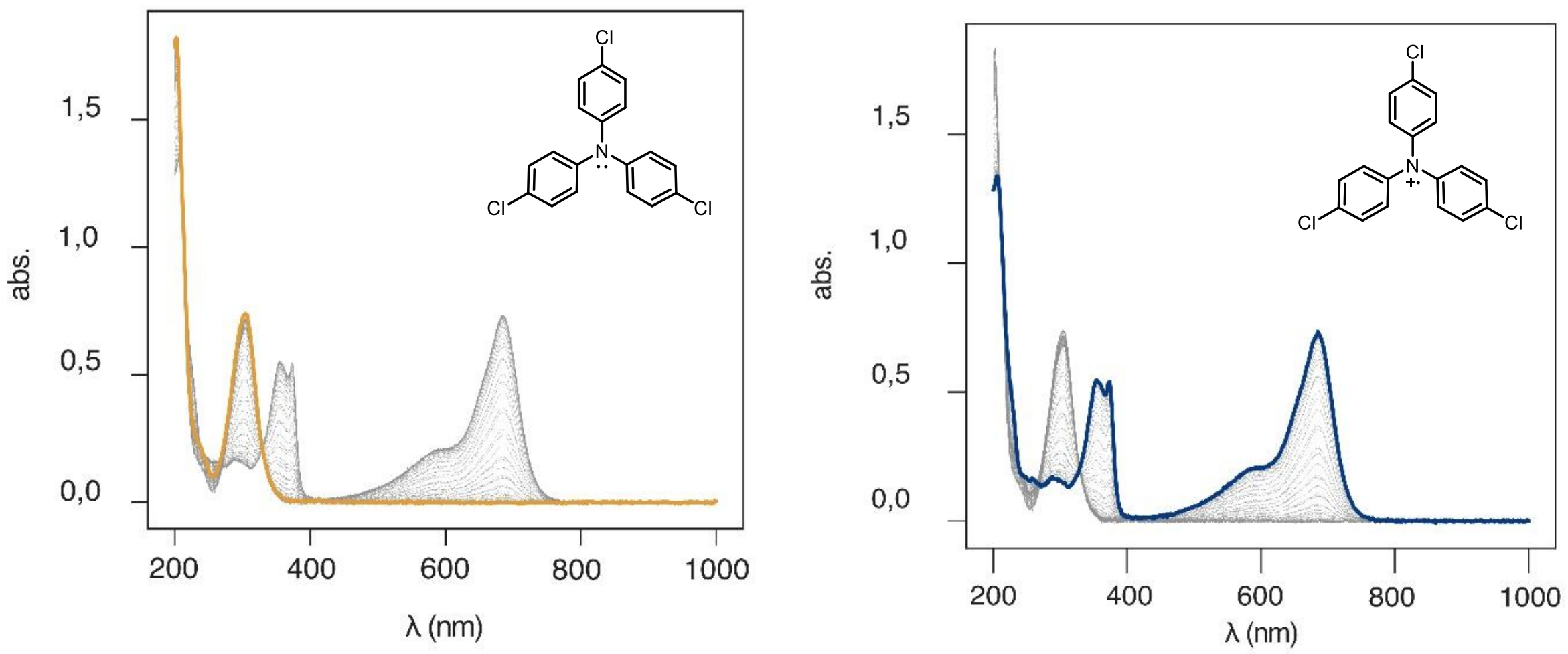

Figure S17. UV-vis spectra of **3a** at E = 0.6 V vs SCE (orange trace, left) and at E = 1.3 V vs SCE (blue trace, right).

Spectroelectrochemistry experiments were performed using an Agilent Cary 60 spectrometer and an PalmSens EmStat4S HR potentiostat. An optically transparent thin-layer electrode (OTTLE) cell was employed as reported previously.[37] The cell consists of a Specac Omni Cell body with CaF_2 windows and a thin spacer (approximately 0.2 mm) in between which electrodes (working / auxiliary: Pt-mesh, psuedoreference Ag-wire) are mounted. Parts of the cell which are not covered by the electrodes are masked by black tape. Cyclic voltammetry was then run of a 1 mM solution of **3a** containing 0.1 M of $n$$Bu_4NPF_6$ as supporting electrolyte at a scan-rate of 1 mV s–1 and UV-vis spectra were collected every 15 s.

J.2. Radical trapping experiment

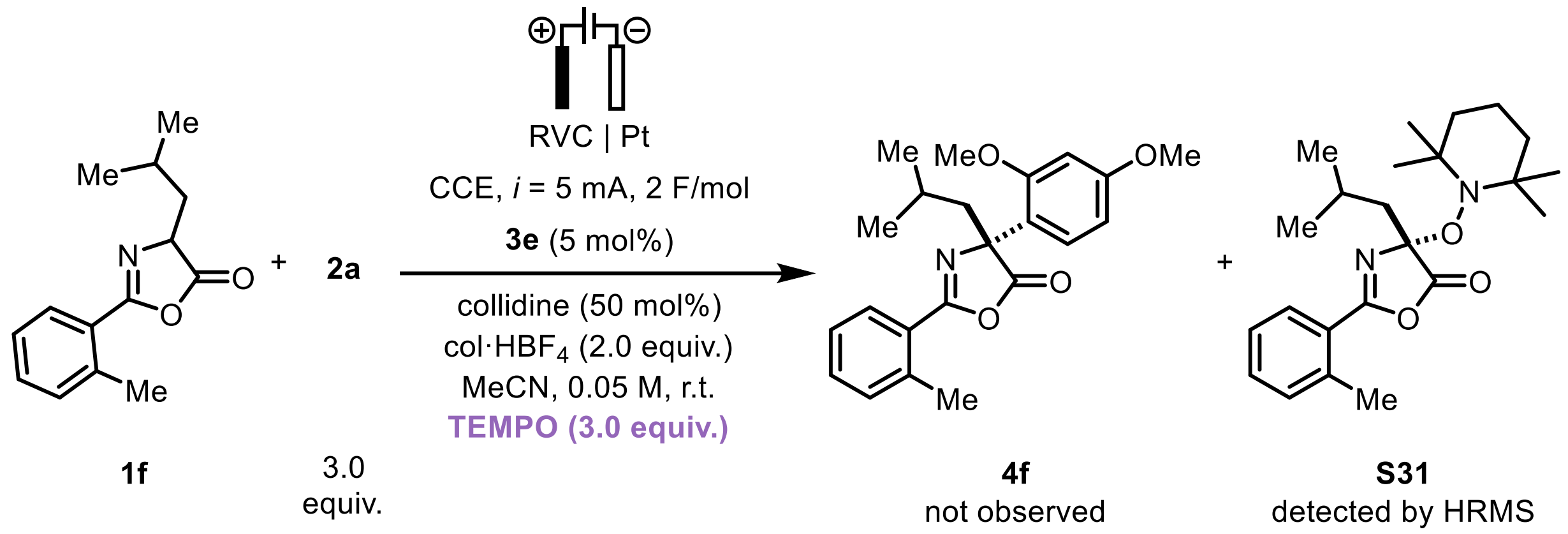

A 10 mL ElectraSyn vial with a magnetic stirring bar was charged with azlactone **1f** (37.8 mg, 0.2 mmol, 1.0 equiv.), electrocatalyst **3e** (3.4 mg, 0.01 mmol, 5 mol%), TEMPO (93.6 mg, 0.6 mmol, 3.0 equiv.), and [collidinium·BF_4] (83.6 mg, 0.4 mmol, 2.0 equiv.). The ElectraSyn vial cap equipped with anode (RVC) and cathode (platinum foil) was inserted and the system was purged with N_2. Subsequently, MeCN (8 mL, 0.025 M), 1,3-dimethoxybenzene (**2a**, 79 μL, 0.6 mmol, 3.0 equiv.), and collidine (13 μL, 0.1 mmol, 0.5 equiv.) were added. The resulting mixture was stirred and electrolysed at constant current (5 mA) for 2 hours. Then the electrodes were rinsed with CH_2Cl_2. Product **4f** was not observed and product **S31** was detected by HRMS. **HRMS (ESI)** Calculated for $C_{23}H_{35}N_2O_3^+$ ([M+H]$^+$) 387.2642. Found 387.2640.

K. References

[1] H. Roth, N. Romero, D. Nicewicz, "Experimental and Calculated Electrochemical Potentials of Common Organic Molecules for Applications to Single-Electron Redox Chemistry" *Synlett* **2015**, *27*, 714–723.

[2] R. Rengasamy, M. J. Curtis-Long, W. D. Seo, S. H. Jeong, I.-Y. Jeong, K. H. Park, "New Building Block for Polyhydroxylated Piperidine: Total Synthesis of 1,6-Dideoxynojirimycin" *J. Org. Chem.* **2008**, *73*, 2898–2901.

[3] M. Weber, S. Jautze, W. Frey, R. Peters, "Bispalladacycle-Catalyzed Michael Addition of In Situ Formed Azlactones to Enones" *Chemistry – A European Journal* **2012**, *18*, 14792–14804.

[4] G. Danger, A. Michaut, M. Bucchi, L. Boiteau, J. Canal, R. Plasson, R. Pascal, "5(4*H*)-Oxazolones as Intermediates in the Carbodiimide- and Cyanamide-Promoted Peptide Activations in Aqueous Solution" *Angew. Chem. Int. Ed.* **2013**, *52*, 611–614.

[5] E. Badiola, B. Fiser, E. Gómez-Bengoa, A. Mielgo, I. Olaizola, I. Urruzuno, J. M. García, J. M. Odriozola, J. Razkin, M. Oiarbide, C. Palomo, "Enantioselective Construction of Tetrasubstituted Stereogenic Carbons through Brønsted Base Catalyzed Michael Reactions: α′-Hydroxy Enones as Key Enoate Equivalent" *J. Am. Chem. Soc.* **2014**, *136*, 17869–17881.

[6] D. Uraguchi, K. Koshimoto, S. Miyake, T. Ooi, "Chiral Ammonium Betaines as Ionic Nucleophilic Catalysts" *Angew. Chem. Int. Ed.* **2010**, *49*, 5567–5569.

[7] H. Wegmann, W. Steglich, "α-Aminosaeuren als nucleophile Acylaequivalente, V. Sterisch gelenkte Michael-Addition von Oxazolin-5-on-Anionen an aktivierte Doppelbindungen; Synthese von 1,4-Dicarbonylverbindungen und γ-Oxonitrilen" *Chem. Ber.* **1981**, *114*, 2580–2594.

[8] A. D. Melhado, M. Luparia, F. D. Toste, "Au(I)-Catalyzed Enantioselective 1,3-Dipolar Cycloadditions of Münchnones with Electron-Deficient Alkenes" *J. Am. Chem. Soc.* **2007**, *129*, 12638–12639.

[9] D. Zhu, X. Zhang, M. Yan, "Enantioselective Addition of Azlactones to Ethylene Sulfonyl Fluoride via Dual Catalysis" *Org. Lett.* **2021**, *23*, 4228–4232.

[10] G. Yin, X. Wang, Y. Wang, T. Shi, Y. Zeng, Y. Wang, X. Peng, Z. Wang, "Lawesson's reagent promoted deoxygenation of azlactones for the syntheses of 2,4-disubstituted thiazoles" *Org. Biomol. Chem.* **2022**, *20*, 9589–9592.

[11] K. Gottwald, D. Seebach, "Ring opening with kinetic resolution of azlactones by Ti-TADDOLates" *Tetrahedron* **1999**, *55*, 723–738.

[12] T. C. Sanders, C. T. Seto, "4-Heterocyclohexanone-Based Inhibitors of the Serine Protease Plasmin" *J. Med. Chem.* **1999**, *42*, 2969–2976.

[13] C. Stein, J. L. Tyler, J. Wiener, F. Boser, C. G. Daniliuc, F. Glorius, "Anomeric Amide-Enabled Alkene-Arene and Alkene-Alkene Aminative Coupling" *Angew. Chem. Int. Ed.* **2025**, *64*, e202418141.

[14] J. Duran, J. Mateos, A. Moyano, X. Companyó, "Catalytic asymmetric defluorinative allylation of silyl enol ethers" *Chem. Sci.* **2023**, *14*, 7147–7153.

[15] I. Khan, B. G. Reed-Berendt, R. L. Melen, L. C. Morrill, "FLP-Catalyzed Transfer Hydrogenation of Silyl Enol Ethers" *Angew. Chem. Int. Ed.* **2018**, *57*, 12356–12359.

[16] W. Ma, A. Schmidt, C. Strohmann, C. C. J. Loh, "Stereoselective Entry into α,α'- *C* -Oxepane Scaffolds through a Chalcogen Bonding Catalyzed Strain-Release *C* -Septanosylation Strategy" *Angew. Chem. Int. Ed.* **2024**, *63*, e202405706.

[17] S. D. Schnell, J. A. González, J. Sklyaruk, A. Linden, K. Gademann, "Boron Trifluoride-Mediated Cycloaddition of 3-Bromotetrazine and Silyl Enol Ethers: Synthesis of 3-Bromo-pyridazines" *J. Org. Chem.* **2021**, *86*, 12008–12023.

[18] M. Mahlau, P. García-García, B. List, "Asymmetric Counteranion-Directed Catalytic Hosomi–Sakurai Reaction" *Chemistry – A European Journal* **2012**, *18*, 16283–16287.

[19] G. A. Molander, L. A. Felix, "Stereoselective Suzuki−Miyaura Cross-Coupling Reactions of Potassium Alkenyltrifluoroborates with Alkenyl Bromides" *J. Org. Chem.* **2005**, *70*, 3950–3956.

[20] K. J. Gonzalez, A. W. Rand, B. M. Stoltz, "Development of a Non-Directed Petasis-Type Reaction by an Aromaticity-Disrupting Strategy" *Angew. Chem. Int. Ed.* **2023**, *62*, e202218921.

[21] X. Wu, A. P. Davis, P. C. Lambert, L. Kraig Steffen, O. Toy, A. J. Fry, "Substituent effects on the redox properties and structure of substituted triphenylamines. An experimental and computational study" *Tetrahedron* **2009**, *65*, 2408–2414.

[22] C. Sivarajan, S. Saha, S. Mulla, R. Mitra, "$NaNH_2$ as a Nitrogen Source and Base to Synthesize Triarylamines from Aryl Halides Using Pd-Catalyzed C–N Coupling" *J. Org. Chem.* **2024**, *89*, 17021–17030.

[23] Z. Li, H. Ishizuka, Y. Sei, M. Akita, M. Yoshizawa, "Extended Fluorochromism of Anthracene Trimers with a *meta*-Substituted Triphenylamine or Triphenylphosphine Core" *Chem. Asian J.* **2012**, *7*, 1789–1794.

[24] P. Li, D. Shimoyama, N. Zhang, Y. Jia, G. Hu, C. Li, X. Yin, N. Wang, F. Jäkle, P. Chen, "A New Platform of B/N-Doped Cyclophanes: Access to a π-Conjugated Block-Type B_3N_3 Macrocycle with Strong Dipole Moment and Unique Optoelectronic Properties" *Angew. Chem. Int. Ed.* **2022**, *61*, e202200612.

[25] M. C. Leech, D. Nagornîi, J. M. Walsh, C. Kiaku, D. L. Poole, J. Mason, I. C. A. Goodall, P. Devo, K. Lam, "eFluorination Using Cheap and Readily Available Tetrafluoroborate Salts" *Org. Lett.* **2023**, *25*, 1353–1358.

[26] P.-T. Li, Q. Mou, W. Yu, "Regioselective 1,4-Hydroamination of 1,3-Dienes by Photoredox/Cobalt Dual Catalysis" *Org. Lett.* **2025**, *27*, 1973–1978.

[27] R. Guo, X. Qi, H. Xiang, P. Geaneotes, R. Wang, P. Liu, Y. Wang, "Stereodivergent Alkyne Hydrofluorination Using Protic Tetrafluoroborates as Tunable Reagents" *Angew. Chem. Int. Ed.* **2020**, *59*, 16651–16660.

[28] A. P. Atkins, A. C. Rowett, D. M. Heard, J. A. Tate, A. J. J. Lennox, "Electrochemical Benzylic $C(sp^3)$–H Acyloxylation" *Org. Lett.* **2022**, *24*, 5105–5108.

[29] A. Choi, D. M. Heard, C. S. Haydon, A. J. J. Lennox, "Open-ESyn: A 3D-Printed Toolkit for Expanded ElectraSyn Functionality and Reproducibility for Electrosynthesis" *ChemElectroChem* **2024**, *11*, e202400454.

[30] N. Fu, G. S. Sauer, S. Lin, "A general, electrocatalytic approach to the synthesis of vicinal diamines" *Nat. Protoc.* **2018**, *13*, 1725–1743.

[31] M. Weber, W. Frey, R. Peters, "Catalytic Asymmetric Synthesis of Spirocyclic Azlactones by a Double Michael-Addition Approach" *Chem. Eur. J.* **2013**, *19*, 8342–8351.

[32] S. Cabrera, E. Reyes, J. Alemán, A. Milelli, S. Kobbelgaard, K. A. Jørgensen, "Organocatalytic Asymmetric Synthesis of α,α-Disubstituted α-Amino Acids and Derivatives" *J. Am. Chem. Soc.* **2008**, *130*, 12031–12037.

[33] A. A. Pereira, P. P. de Castro, A. C. de Mello, B. R. V. Ferreira, M. N. Eberlin, G. W. Amarante, "Brønsted acid catalyzed azlactone ring opening by nucleophiles" *Tetrahedron* **2014**, *70*, 3271–3275.

[34] A. R. Katritzky, K. Sakizadeh, J. Swinson, S. M. Helmann, L. R. Krepski, S. V. Pathre, J. K. Rasmussen, "A Novel Synthesis of Benzothiazoles" *Synth. Commun.* **1988**, *18*, 651–658.

[35] M. Serra, E. Bernardi, G. Marrubini, L. Colombo, "One-Pot Vinylation of Azlactones: Fast Access to Enantioenriched α-Vinyl Quaternary Amino Acids" *European J. Org. Chem.* **2017**, *2017*, 2964–2970.

[36] M. G. Núñez, A. J. M. Farley, D. J. Dixon, "Bifunctional Iminophosphorane Organocatalysts for Enantioselective Synthesis: Application to the Ketimine Nitro-Mannich Reaction" *J. Am. Chem. Soc.* **2013**, *135*, 16348–16351.
[37] M. Krejčik, M. Daněk, F. Hartl, "Simple construction of an infrared optically transparent thin-layer electrochemical cell" *J. Electroanal. Chem. Interfacial Electrochem.* **1991**, *317*, 179–187.

L. Determination of the absolute configuration. X-Ray Crystallographic Data of compound 4e

A colourless prism-like specimen with approximate dimensions 0.202 mm x 0.089 mm x 0.049 mm, was used for the X-ray crystallographic analysis.

The X-ray intensity data were measured at 100K on a D8 Venture system equipped with a multilayer monochromator and a Mo microfocus (λ = 0.71073 Å). The frames were integrated with the Bruker SAINT software package using a narrow-frame algorithm. The integration of the data using an orthorhombic unit cell yielded a total of 182377 reflections to a maximum θ angle of 30.55 (0.70 Å resolution), of which 10099 were independent (average redundancy 18.059, completeness = 99.9%, R_{int} = 12.93%, R_{sig} = 4.80%) and 9139 (90.49%) were greater than $2\sigma(F^2)$. The final cell constants of a = 20.7545(7) Å, b = 6.5905(2) Å, c = 24.0886(9) Å, volume = 3294.90(19) $Å^3$, are based upon the refinement of the XYZ-centroids of reflections above 20 σ(I). Data were corrected for absorption effects using the multi-scan method (SADABS). The calculated minimum and maximum transmission coefficients (based on crystal size) are 0.5475 and 0.7433.

The structure was solved and refined using the Bruker SHELXTL Software Package, using the space group *Pca*2_1, with Z = 8 for the formula unit, $C_{19}H_{19}NO_4$. The final anisotropic full-matrix least-squares refinement on F^2 with 441 variables converged at R1 = 4.20%, for the observed data and wR2 = 10.22% for all data. The goodness-of-fit was 1.019. The largest peak in the final difference electron density synthesis was 0.362 $e^-/Å^3$ and the largest hole was -0.266 $e^-/Å^3$ with an RMS deviation of 0.048 $e^-/Å^3$. On the basis of the final model, the calculated density was 1.312 g/cm^3 and F(000), 1376 e^-.

Table S11. Sample and crystal data for **4e**

Identification code	**4e**	
Chemical formula	$C_{19}H_{19}NO_4$	
Formula weight	325.35 g/mol	
Temperature	100(2) K	
Wavelength	0.71073 Å	
Crystal system	orthorhombic	
Space group	P c a 21	
Unit cell dimensions	a = 20.7545(7) Å	α = 90°
	b = 6.5905(2) Å	β = 90°
	c = 24.0886(9) Å	γ = 90°
Volume	3294.90(19) $Å^3$	
Z	8	
Density (calculated)	1.312 g/cm^3	
Absorption coefficient	0.092 mm^{-1}	
F(000)	1376	

Table S12. Data collection and structure refinement for **4e**.

Theta range for data collection	1.96 to 30.55°	
Index ranges	-29<=h<=29, -9<=k<=9, -34<=l<=34	
Reflections collected	182377	
Independent reflections	10099 [R(int) = 0.1293]	
Refinement method	Full-matrix least-squares on F^2	
Refinement program	SHELXL-2019/1 (Sheldrick, 2019)	
Function minimised	Σ w(F_o^2 - F_c^2)^2	
Data / restraints / parameters	10099 / 1 / 441	
Goodness-of-fit on F^2	1.019	
Final R indices	9139 data; I>2σ(I)	R1 = 0.0420, wR2 = 0.0978
	all data	R1 = 0.0493, wR2 = 0.1022
Weighting scheme	$w=1/[\sigma^2(F_o^2)+(0.0430P)^2+1.1331P]$ where $P=(F_o^2+2F_c^2)/3$	
Absolute structure parameter	0.5(3)	
Largest diff. peak and hole	0.362 and -0.266 eÅ^{-3}	
R.M.S. deviation from mean	0.048 eÅ^{-3}	

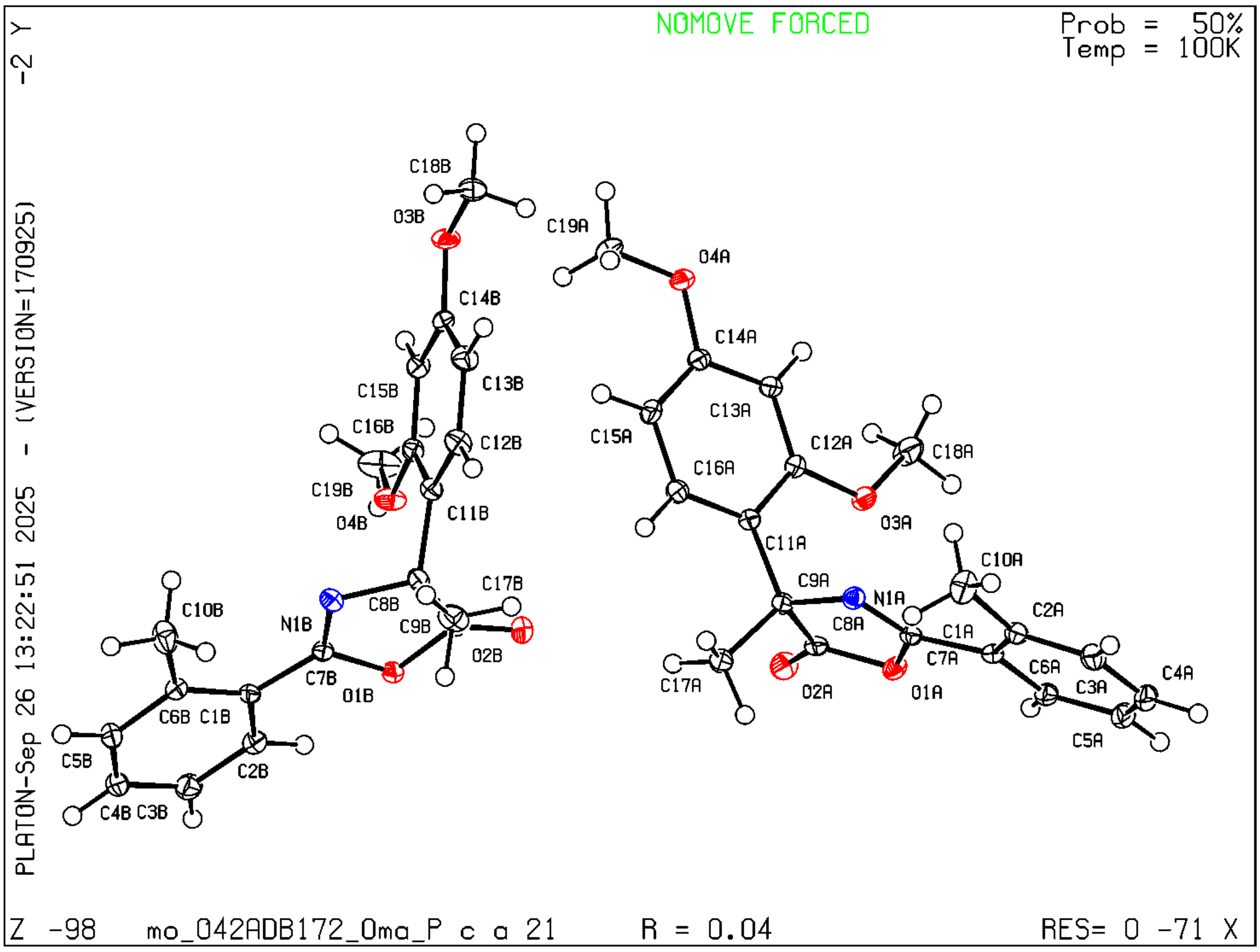

Figure S18. ORTEP diagram and atomic numbering of **4e**.

Table S13. Atomic coordinates and equivalent isotropic atomic displacement parameters (Å^2) for **4e**. U(eq) is defined as one third of the trace of the orthogonalised U_{ij} tensor.

	**x/a**	**y/b**	**z/c**	**U(eq)**
C1A	0.31326(9)	0.4792(3)	0.74443(8)	0.0135(4)
C2A	0.32454(10)	0.6473(3)	0.77944(9)	0.0157(4)
C3A	0.29639(11)	0.6449(4)	0.83232(9)	0.0192(4)
C4A	0.26003(11)	0.4817(4)	0.85083(9)	0.0217(4)
C5A	0.25083(12)	0.3151(4)	0.81670(10)	0.0205(4)
C6A	0.27666(10)	0.3152(3)	0.76355(9)	0.0163(4)
C7A	0.33725(9)	0.4739(3)	0.68694(8)	0.0127(4)
C8A	0.34223(10)	0.3291(3)	0.60385(9)	0.0146(4)
C9A	0.37729(10)	0.5331(3)	0.60324(8)	0.0131(4)
C10A	0.36488(13)	0.8273(4)	0.76322(10)	0.0237(5)
C11A	0.44749(10)	0.5163(3)	0.58613(8)	0.0130(4)
C12A	0.48593(10)	0.3633(3)	0.60991(8)	0.0139(4)
C13A	0.55158(10)	0.3520(3)	0.59896(9)	0.0145(4)
C14A	0.57979(10)	0.4949(3)	0.56382(8)	0.0139(4)
C15A	0.54272(10)	0.6433(3)	0.53799(9)	0.0160(4)
C16A	0.47669(10)	0.6513(3)	0.54980(9)	0.0155(4)
C17A	0.33770(10)	0.6766(3)	0.56682(9)	0.0184(4)
C18A	0.48790(12)	0.0594(4)	0.66390(13)	0.0307(6)
C19A	0.67585(11)	0.6178(4)	0.52176(10)	0.0201(4)
N1A	0.37217(8)	0.6022(3)	0.66159(7)	0.0139(3)
O1A	0.31564(7)	0.3066(2)	0.65606(6)	0.0151(3)
O2A	0.33183(8)	0.2100(2)	0.56727(7)	0.0198(3)
O3A	0.45396(7)	0.2327(2)	0.64375(7)	0.0195(3)
O4A	0.64522(7)	0.4745(2)	0.55752(7)	0.0166(3)
C1B	0.36421(9)	0.9989(3)	0.25449(8)	0.0136(4)
C2B	0.32742(10)	0.8325(3)	0.23745(9)	0.0177(4)
C3B	0.30036(11)	0.8256(4)	0.18478(10)	0.0219(5)
C4B	0.30893(11)	0.9881(4)	0.14909(9)	0.0211(4)
C5B	0.34582(11)	0.1531(4)	0.16529(9)	0.0183(4)
C6B	0.37477(10)	0.1626(3)	0.21778(9)	0.0150(4)
C7B	0.38913(9)	0.0015(3)	0.31181(8)	0.0134(4)
C8B	0.43274(10)	0.0661(3)	0.39397(8)	0.0147(4)
C9B	0.39467(10)	0.8668(3)	0.39627(9)	0.0151(4)
C10B	0.41459(13)	0.3453(4)	0.23214(10)	0.0237(5)
C11B	0.50318(10)	0.0387(3)	0.40976(8)	0.0141(4)
C12B	0.53524(11)	0.1666(3)	0.44582(9)	0.0177(4)
C13B	0.60103(11)	0.1449(3)	0.45689(10)	0.0189(4)
C14B	0.63496(10)	0.9920(3)	0.43082(9)	0.0152(4)
C15B	0.60381(10)	0.8560(3)	0.39514(9)	0.0147(4)
C16B	0.53826(10)	0.8794(3)	0.38517(8)	0.0138(4)
C17B	0.39687(11)	0.2213(4)	0.43000(10)	0.0221(5)
C18B	0.73382(11)	0.0914(4)	0.47416(10)	0.0218(5)

	x/a	y/b	z/c	U(eq)
C19B	0.53471(13)	0.5755(4)	0.33088(13)	0.0304(6)
N1B	0.42672(9)	0.1279(3)	0.33517(7)	0.0149(3)
O1B	0.36604(7)	0.8427(2)	0.34483(6)	0.0151(3)
O2B	0.38480(8)	0.7534(3)	0.43361(7)	0.0205(3)
O3B	0.69977(7)	0.9575(3)	0.43749(7)	0.0187(3)
O4B	0.50361(7)	0.7535(3)	0.35181(7)	0.0190(3)

Table S14. Bond lengths (Å) for **4e**.

C1A-C6A	1.399(3)	C1A-C2A	1.412(3)
C1A-C7A	1.472(3)	C2A-C3A	1.402(3)
C2A-C10A	1.504(3)	C3A-C4A	1.388(3)
C3A-H3A	0.950000	C4A-C5A	1.385(3)
C4A-H4A	0.950000	C5A-C6A	1.388(3)
C5A-H5A	0.950000	C6A-H6A	0.950000
C7A-N1A	1.271(3)	C7A-O1A	1.403(2)
C8A-O2A	1.199(3)	C8A-O1A	1.382(2)
C8A-C9A	1.529(3)	C9A-N1A	1.482(3)
C9A-C11A	1.518(3)	C9A-C17A	1.529(3)
C10A-H10A	0.980000	C10A-H10B	0.980000
C10A-H10C	0.980000	C11A-C16A	1.387(3)
C11A-C12A	1.408(3)	C12A-O3A	1.359(2)
C12A-C13A	1.390(3)	C13A-C14A	1.395(3)
C13A-H13A	0.950000	C14A-O4A	1.373(2)
C14A-C15A	1.391(3)	C15A-C16A	1.401(3)
C15A-H15A	0.950000	C16A-H16A	0.950000
C17A-H17A	0.980000	C17A-H17B	0.980000
C17A-H17C	0.980000	C18A-O3A	1.427(3)
C18A-H18A	0.980000	C18A-H18B	0.980000
C18A-H18C	0.980000	C19A-O4A	1.428(3)
C19A-H19A	0.980000	C19A-H19B	0.980000
C19A-H19C	0.980000	C1B-C2B	1.398(3)
C1B-C6B	1.412(3)	C1B-C7B	1.474(3)
C2B-C3B	1.388(3)	C2B-H2B	0.950000
C3B-C4B	1.385(3)	C3B-H3B	0.950000
C4B-C5B	1.386(3)	C4B-H4B	0.950000
C5B-C6B	1.401(3)	C5B-H5B	0.950000
C6B-C10B	1.501(3)	C7B-N1B	1.272(3)
C7B-O1B	1.399(2)	C8B-N1B	1.479(3)
C8B-C11B	1.521(3)	C8B-C9B	1.534(3)
C8B-C17B	1.534(3)	C9B-O2B	1.187(3)
C9B-O1B	1.384(2)	C10B-H10D	0.980000
C10B-H10E	0.980000	C10B-H10F	0.980000
C11B-C12B	1.381(3)	C11B-C16B	1.408(3)

C12B-C13B	1.399(3)	C12B-H12B	0.950000
C13B-C14B	1.380(3)	C13B-H13B	0.950000
C14B-O3B	1.374(2)	C14B-C15B	1.400(3)
C15B-C16B	1.390(3)	C15B-H15B	0.950000
C16B-O4B	1.361(2)	C17B-H17D	0.980000
C17B-H17E	0.980000	C17B-H17F	0.980000
C18B-O3B	1.435(3)	C18B-H18D	0.980000
C18B-H18E	0.980000	C18B-H18F	0.980000
C19B-O4B	1.431(3)	C19B-H19D	0.980000
C19B-H19E	0.980000	C19B-H19F	0.980000

Table S15. Bond angles (º) for **4e**.

C6A-C1A-C2A	119.97(19)	C6A-C1A-C7A	118.35(19)
C2A-C1A-C7A	121.64(19)	C3A-C2A-C1A	117.7(2)
C3A-C2A-C10A	118.5(2)	C1A-C2A-C10A	123.79(19)
C4A-C3A-C2A	121.8(2)	C4A-C3A-H3A	119.100000
C2A-C3A-H3A	119.100000	C5A-C4A-C3A	119.9(2)
C5A-C4A-H4A	120.000000	C3A-C4A-H4A	120.000000
C4A-C5A-C6A	119.6(2)	C4A-C5A-H5A	120.200000
C6A-C5A-H5A	120.200000	C5A-C6A-C1A	120.9(2)
C5A-C6A-H6A	119.600000	C1A-C6A-H6A	119.600000
N1A-C7A-O1A	116.78(18)	N1A-C7A-C1A	128.99(19)
O1A-C7A-C1A	114.17(17)	O2A-C8A-O1A	121.80(19)
O2A-C8A-C9A	130.8(2)	O1A-C8A-C9A	107.05(17)
N1A-C9A-C11A	110.41(16)	N1A-C9A-C8A	103.14(16)
C11A-C9A-C8A	113.29(17)	N1A-C9A-C17A	108.39(16)
C11A-C9A-C17A	113.89(17)	C8A-C9A-C17A	107.07(17)
C2A-C10A-H10A	109.500000	C2A-C10A-H10B	109.500000
H10A-C10A-H10B	109.500000	C2A-C10A-H10C	109.500000
H10A-C10A-H10C	109.500000	H10B-C10A-H10C	109.500000
C16A-C11A-C12A	117.94(19)	C16A-C11A-C9A	122.94(18)
C12A-C11A-C9A	119.05(18)	O3A-C12A-C13A	123.96(18)
O3A-C12A-C11A	114.92(18)	C13A-C12A-C11A	121.12(19)
C12A-C13A-C14A	119.36(19)	C12A-C13A-H13A	120.300000
C14A-C13A-H13A	120.300000	O4A-C14A-C15A	124.50(19)
O4A-C14A-C13A	114.58(18)	C15A-C14A-C13A	120.91(19)
C14A-C15A-C16A	118.48(19)	C14A-C15A-H15A	120.800000
C16A-C15A-H15A	120.800000	C11A-C16A-C15A	122.09(19)
C11A-C16A-H16A	119.000000	C15A-C16A-H16A	119.000000
C9A-C17A-H17A	109.500000	C9A-C17A-H17B	109.500000
H17A-C17A-H17B	109.500000	C9A-C17A-H17C	109.500000
H17A-C17A-H17C	109.500000	H17B-C17A-H17C	109.500000
O3A-C18A-H18A	109.500000	O3A-C18A-H18B	109.500000
H18A-C18A-H18B	109.500000	O3A-C18A-H18C	109.500000

H18A-C18A-H18C	109.500000	H18B-C18A-H18C	109.500000
O4A-C19A-H19A	109.500000	O4A-C19A-H19B	109.500000
H19A-C19A-H19B	109.500000	O4A-C19A-H19C	109.500000
H19A-C19A-H19C	109.500000	H19B-C19A-H19C	109.500000
C7A-N1A-C9A	106.98(17)	C8A-O1A-C7A	105.71(15)
C12A-O3A-C18A	118.05(17)	C14A-O4A-C19A	116.25(17)
C2B-C1B-C6B	120.01(19)	C2B-C1B-C7B	118.41(19)
C6B-C1B-C7B	121.53(19)	C3B-C2B-C1B	121.0(2)
C3B-C2B-H2B	119.500000	C1B-C2B-H2B	119.500000
C4B-C3B-C2B	119.4(2)	C4B-C3B-H3B	120.300000
C2B-C3B-H3B	120.300000	C3B-C4B-C5B	120.2(2)
C3B-C4B-H4B	119.900000	C5B-C4B-H4B	119.900000
C4B-C5B-C6B	121.7(2)	C4B-C5B-H5B	119.100000
C6B-C5B-H5B	119.100000	C5B-C6B-C1B	117.7(2)
C5B-C6B-C10B	118.7(2)	C1B-C6B-C10B	123.6(2)
N1B-C7B-O1B	116.62(18)	N1B-C7B-C1B	129.58(19)
O1B-C7B-C1B	113.78(17)	N1B-C8B-C11B	110.68(17)
N1B-C8B-C9B	103.10(17)	C11B-C8B-C9B	112.60(17)
N1B-C8B-C17B	108.50(17)	C11B-C8B-C17B	113.84(18)
C9B-C8B-C17B	107.48(18)	O2B-C9B-O1B	122.1(2)
O2B-C9B-C8B	130.9(2)	O1B-C9B-C8B	106.70(17)
C6B-C10B-H10D	109.500000	C6B-C10B-H10E	109.500000
H10D-C10B-H10E	109.500000	C6B-C10B-H10F	109.500000
H10D-C10B-H10F	109.500000	H10E-C10B-H10F	109.500000
C12B-C11B-C16B	118.06(19)	C12B-C11B-C8B	123.21(19)
C16B-C11B-C8B	118.70(18)	C11B-C12B-C13B	121.9(2)
C11B-C12B-H12B	119.100000	C13B-C12B-H12B	119.100000
C14B-C13B-C12B	119.1(2)	C14B-C13B-H13B	120.500000
C12B-C13B-H13B	120.500000	O3B-C14B-C13B	124.55(19)
O3B-C14B-C15B	114.71(19)	C13B-C14B-C15B	120.7(2)
C16B-C15B-C14B	119.15(19)	C16B-C15B-H15B	120.400000
C14B-C15B-H15B	120.400000	O4B-C16B-C15B	123.48(19)
O4B-C16B-C11B	115.46(18)	C15B-C16B-C11B	121.06(19)
C8B-C17B-H17D	109.500000	C8B-C17B-H17E	109.500000
H17D-C17B-H17E	109.500000	C8B-C17B-H17F	109.500000
H17D-C17B-H17F	109.500000	H17E-C17B-H17F	109.500000
O3B-C18B-H18D	109.500000	O3B-C18B-H18E	109.500000
H18D-C18B-H18E	109.500000	O3B-C18B-H18F	109.500000
H18D-C18B-H18F	109.500000	H18E-C18B-H18F	109.500000
O4B-C19B-H19D	109.500000	O4B-C19B-H19E	109.500000
H19D-C19B-H19E	109.500000	O4B-C19B-H19F	109.500000
H19D-C19B-H19F	109.500000	H19E-C19B-H19F	109.500000
C7B-N1B-C8B	107.18(18)	C9B-O1B-C7B	106.02(16)
C14B-O3B-C18B	116.91(18)	C16B-O4B-C19B	118.01(17)

Table S16. Torsion angles (°) for **4e**.

C6A-C1A-C2A-C3A	-1.9(3)	C7A-C1A-C2A-C3A	175.99(19)
C6A-C1A-C2A-C10A	178.1(2)	C7A-C1A-C2A-C10A	-4.1(3)
C1A-C2A-C3A-C4A	1.7(3)	C10A-C2A-C3A-C4A	-178.2(2)
C2A-C3A-C4A-C5A	0.1(4)	C3A-C4A-C5A-C6A	-1.8(4)
C4A-C5A-C6A-C1A	1.6(4)	C2A-C1A-C6A-C5A	0.2(3)
C7A-C1A-C6A-C5A	-177.7(2)	C6A-C1A-C7A-N1A	-178.2(2)
C2A-C1A-C7A-N1A	3.9(3)	C6A-C1A-C7A-O1A	4.8(3)
C2A-C1A-C7A-O1A	-173.13(18)	O2A-C8A-C9A-N1A	179.2(2)
O1A-C8A-C9A-N1A	5.9(2)	O2A-C8A-C9A-C11A	-61.4(3)
O1A-C8A-C9A-C11A	125.25(18)	O2A-C8A-C9A-C17A	65.0(3)
O1A-C8A-C9A-C17A	-108.35(18)	N1A-C9A-C11A-C16A	-108.0(2)
C8A-C9A-C11A-C16A	136.9(2)	C17A-C9A-C11A-C16A	14.2(3)
N1A-C9A-C11A-C12A	68.9(2)	C8A-C9A-C11A-C12A	-46.2(3)
C17A-C9A-C11A-C12A	-168.85(19)	C16A-C11A-C12A-O3A	-178.10(19)
C9A-C11A-C12A-O3A	4.8(3)	C16A-C11A-C12A-C13A	2.3(3)
C9A-C11A-C12A-C13A	-174.80(19)	O3A-C12A-C13A-C14A	-179.5(2)
C11A-C12A-C13A-C14A	0.0(3)	C12A-C13A-C14A-O4A	177.64(19)
C12A-C13A-C14A-C15A	-2.5(3)	O4A-C14A-C15A-C16A	-177.61(19)
C13A-C14A-C15A-C16A	2.6(3)	C12A-C11A-C16A-C15A	-2.2(3)
C9A-C11A-C16A-C15A	174.7(2)	C14A-C15A-C16A-C11A	-0.1(3)
O1A-C7A-N1A-C9A	2.0(2)	C1A-C7A-N1A-C9A	-174.92(19)
C11A-C9A-N1A-C7A	-126.06(18)	C8A-C9A-N1A-C7A	-4.7(2)
C17A-C9A-N1A-C7A	108.56(19)	O2A-C8A-O1A-C7A	-178.92(19)
C9A-C8A-O1A-C7A	-4.8(2)	N1A-C7A-O1A-C8A	1.9(2)
C1A-C7A-O1A-C8A	179.34(16)	C13A-C12A-O3A-C18A	-7.9(3)
C11A-C12A-O3A-C18A	172.5(2)	C15A-C14A-O4A-C19A	0.1(3)
C13A-C14A-O4A-C19A	179.89(19)	C6B-C1B-C2B-C3B	-0.5(3)
C7B-C1B-C2B-C3B	177.2(2)	C1B-C2B-C3B-C4B	-1.3(3)
C2B-C3B-C4B-C5B	1.9(4)	C3B-C4B-C5B-C6B	-0.7(3)
C4B-C5B-C6B-C1B	-1.1(3)	C4B-C5B-C6B-C10B	179.4(2)
C2B-C1B-C6B-C5B	1.7(3)	C7B-C1B-C6B-C5B	-175.93(18)
C2B-C1B-C6B-C10B	-178.8(2)	C7B-C1B-C6B-C10B	3.5(3)
C2B-C1B-C7B-N1B	174.7(2)	C6B-C1B-C7B-N1B	-7.6(3)
C2B-C1B-C7B-O1B	-7.0(3)	C6B-C1B-C7B-O1B	170.72(18)
N1B-C8B-C9B-O2B	179.2(2)	C11B-C8B-C9B-O2B	59.8(3)
C17B-C8B-C9B-O2B	-66.3(3)	N1B-C8B-C9B-O1B	-6.1(2)
C11B-C8B-C9B-O1B	-125.42(18)	C17B-C8B-C9B-O1B	108.42(19)
N1B-C8B-C11B-C12B	111.1(2)	C9B-C8B-C11B-C12B	-134.1(2)
C17B-C8B-C11B-C12B	-11.4(3)	N1B-C8B-C11B-C16B	-66.8(2)
C9B-C8B-C11B-C16B	48.0(3)	C17B-C8B-C11B-C16B	170.7(2)
C16B-C11B-C12B-C13B	1.9(3)	C8B-C11B-C12B-C13B	-176.0(2)
C11B-C12B-C13B-C14B	0.3(3)	C12B-C13B-C14B-O3B	178.8(2)
C12B-C13B-C14B-C15B	-2.0(3)	O3B-C14B-C15B-C16B	-179.32(19)
C13B-C14B-C15B-C16B	1.4(3)	C14B-C15B-C16B-O4B	-179.1(2)

C14B-C15B-C16B-C11B	0.9(3)	C12B-C11B-C16B-O4B	177.44(19)
C8B-C11B-C16B-O4B	-4.6(3)	C12B-C11B-C16B-C15B	-2.5(3)
C8B-C11B-C16B-C15B	175.49(19)	O1B-C7B-N1B-C8B	-1.9(2)
C1B-C7B-N1B-C8B	176.4(2)	C11B-C8B-N1B-C7B	125.40(19)
C9B-C8B-N1B-C7B	4.8(2)	C17B-C8B-N1B-C7B	-109.0(2)
O2B-C9B-O1B-C7B	-179.5(2)	C8B-C9B-O1B-C7B	5.1(2)
N1B-C7B-O1B-C9B	-2.3(2)	C1B-C7B-O1B-C9B	179.19(16)
C13B-C14B-O3B-C18B	-0.1(3)	C15B-C14B-O3B-C18B	-179.32(19)
C15B-C16B-O4B-C19B	7.1(3)	C11B-C16B-O4B-C19B	-172.8(2)

Table S17. Anisotropic atomic displacement parameters (Å^2) for **4e**. The anisotropic atomic displacement factor exponent takes the form: $-2\pi^2[h^2 a^{*2} U_{11} + ... + 2 h k a^* b^* U_{12}]$

	U_{11}	U_{22}	U_{33}	U_{23}	U_{13}	U_{12}
C1A	0.0100(8)	0.0164(9)	0.0140(8)	0.0011(7)	-0.0003(7)	0.0002(7)
C2A	0.0134(9)	0.0190(10)	0.0147(9)	-0.0003(7)	-0.0011(7)	0.0015(8)
C3A	0.0197(10)	0.0237(10)	0.0142(9)	-0.0040(8)	-0.0011(8)	0.0001(9)
C4A	0.0188(10)	0.0332(12)	0.0130(9)	-0.0010(9)	0.0015(8)	-0.0031(9)
C5A	0.0173(10)	0.0275(11)	0.0168(10)	0.0015(8)	0.0001(8)	-0.0067(9)
C6A	0.0138(9)	0.0206(10)	0.0146(9)	-0.0004(7)	-0.0006(7)	-0.0037(8)
C7A	0.0101(8)	0.0144(9)	0.0135(9)	0.0001(7)	-0.0008(7)	0.0014(7)
C8A	0.0101(8)	0.0181(9)	0.0155(9)	0.0001(7)	0.0001(7)	0.0021(7)
C9A	0.0127(8)	0.0132(9)	0.0133(8)	0.0009(7)	0.0003(7)	0.0018(7)
C10A	0.0313(12)	0.0198(11)	0.0199(10)	-0.0037(8)	0.0033(9)	-0.0063(10)
C11A	0.0119(8)	0.0137(9)	0.0133(8)	0.0000(7)	0.0003(7)	0.0002(7)
C12A	0.0140(9)	0.0141(9)	0.0135(9)	0.0015(7)	0.0009(7)	-0.0009(7)
C13A	0.0120(9)	0.0157(9)	0.0157(9)	0.0011(7)	-0.0010(7)	0.0005(7)
C14A	0.0126(8)	0.0160(9)	0.0130(9)	-0.0025(7)	0.0003(7)	-0.0012(7)
C15A	0.0157(9)	0.0164(10)	0.0161(9)	0.0034(7)	0.0021(7)	-0.0016(8)
C16A	0.0149(9)	0.0150(9)	0.0166(9)	0.0032(7)	0.0001(7)	0.0017(7)
C17A	0.0153(9)	0.0218(10)	0.0182(10)	0.0051(8)	0.0018(8)	0.0052(8)
C18A	0.0206(11)	0.0281(13)	0.0434(15)	0.0226(11)	0.0075(10)	0.0056(10)
C19A	0.0141(9)	0.0229(11)	0.0232(11)	0.0019(9)	0.0028(8)	-0.0050(8)
N1A	0.0134(8)	0.0139(8)	0.0143(8)	0.0002(6)	0.0014(6)	0.0016(6)
O1A	0.0144(7)	0.0159(7)	0.0151(7)	-0.0023(5)	0.0021(5)	-0.0034(5)
O2A	0.0212(7)	0.0206(7)	0.0177(7)	-0.0054(6)	0.0007(6)	-0.0016(6)
O3A	0.0143(7)	0.0190(7)	0.0253(8)	0.0125(6)	0.0032(6)	0.0032(6)
O4A	0.0117(7)	0.0189(7)	0.0192(7)	0.0021(6)	0.0004(6)	-0.0019(6)
C1B	0.0101(8)	0.0179(9)	0.0126(8)	0.0009(7)	-0.0003(7)	0.0028(7)
C2B	0.0151(9)	0.0213(10)	0.0168(10)	0.0008(8)	0.0007(7)	-0.0019(8)
C3B	0.0186(10)	0.0288(12)	0.0182(10)	-0.0023(9)	-0.0006(8)	-0.0068(9)
C4B	0.0163(9)	0.0349(12)	0.0121(9)	-0.0004(9)	-0.0010(8)	-0.0014(9)
C5B	0.0181(10)	0.0225(11)	0.0143(9)	0.0042(8)	0.0001(8)	0.0024(8)
C6B	0.0128(8)	0.0176(9)	0.0146(9)	0.0019(7)	0.0011(7)	0.0005(7)
C7B	0.0111(9)	0.0151(9)	0.0139(9)	0.0020(7)	0.0007(7)	0.0024(7)

	U_{11}	U_{22}	U_{33}	U_{23}	U_{13}	U_{12}
C8B	0.0157(9)	0.0162(9)	0.0122(9)	0.0001(7)	-0.0018(7)	0.0031(7)
C9B	0.0112(8)	0.0208(10)	0.0134(9)	0.0004(7)	-0.0002(7)	0.0047(8)
C10B	0.0326(12)	0.0186(11)	0.0200(11)	0.0034(8)	-0.0036(9)	-0.0074(9)
C11B	0.0139(9)	0.0149(9)	0.0135(9)	-0.0001(7)	-0.0014(7)	-0.0001(7)
C12B	0.0193(10)	0.0157(9)	0.0181(10)	-0.0028(8)	-0.0030(8)	0.0011(8)
C13B	0.0193(10)	0.0184(10)	0.0189(10)	-0.0022(8)	-0.0043(8)	-0.0051(8)
C14B	0.0118(9)	0.0194(10)	0.0144(9)	0.0028(8)	-0.0004(7)	-0.0044(7)
C15B	0.0148(9)	0.0149(9)	0.0145(9)	-0.0013(7)	0.0008(7)	-0.0011(7)
C16B	0.0149(9)	0.0147(9)	0.0119(8)	-0.0013(7)	-0.0011(7)	-0.0024(7)
C17B	0.0207(10)	0.0237(11)	0.0218(10)	-0.0055(9)	-0.0019(9)	0.0099(9)
C18B	0.0154(10)	0.0279(11)	0.0221(10)	0.0004(9)	-0.0055(8)	-0.0097(9)
C19B	0.0219(11)	0.0259(12)	0.0433(15)	-0.0187(11)	-0.0053(11)	0.0045(10)
N1B	0.0150(8)	0.0149(8)	0.0148(8)	0.0005(6)	-0.0020(6)	0.0015(7)
O1B	0.0127(7)	0.0181(7)	0.0146(7)	0.0030(5)	-0.0016(5)	-0.0026(6)
O2B	0.0196(7)	0.0251(8)	0.0169(7)	0.0063(6)	0.0007(6)	0.0008(6)
O3B	0.0114(7)	0.0236(8)	0.0211(7)	-0.0022(6)	-0.0030(6)	-0.0051(6)
O4B	0.0142(7)	0.0207(8)	0.0221(8)	-0.0104(6)	-0.0031(6)	0.0014(6)

Table S18. Hydrogen atomic coordinates and isotropic atomic displacement parameters (Å^2) for **4e**.

	x/a	y/b	z/c	U(eq)
H3A	0.3024	0.7581	0.8562	0.023000
H4A	0.2415	0.4842	0.8869	0.026000
H5A	0.2270	0.2015	0.8296	0.025000
H6A	0.2694	0.2023	0.7398	0.020000
H10A	0.3637	0.9286	0.7930	0.035000
H10B	0.3478	0.8870	0.7290	0.035000
H10C	0.4095	0.7837	0.7571	0.035000
H13A	0.5770	0.2479	0.6152	0.017000
H15A	0.5617	0.7370	0.5129	0.019000
H16A	0.4511	0.7524	0.5324	0.019000
H17A	0.3418	0.6357	0.5279	0.028000
H17B	0.3537	0.8156	0.5713	0.028000
H17C	0.2923	0.6704	0.5780	0.028000
H18A	0.4578	-0.0310	0.6832	0.046000
H18B	0.5216	0.1031	0.6897	0.046000
H18C	0.5076	-0.0129	0.6326	0.046000
H19A	0.7226	0.5972	0.5228	0.030000
H19B	0.6657	0.7557	0.5342	0.030000
H19C	0.6603	0.5989	0.4837	0.030000
H2B	0.3208	0.7225	0.2623	0.021000
H3B	0.2762	0.7106	0.1733	0.026000
H4B	0.2895	0.9864	0.1134	0.025000
H5B	0.3516	1.2627	0.1402	0.022000

	x/a	y/b	z/c	U(eq)
H10D	0.4129	1.4429	0.2015	0.036000
H10E	0.4593	1.3038	0.2384	0.036000
H10F	0.3975	1.4085	0.2659	0.036000
H12B	0.5119	1.2723	0.4636	0.021000
H13B	0.6221	1.2341	0.4820	0.023000
H15B	0.6272	0.7491	0.3780	0.018000
H17D	0.3995	1.1806	0.4691	0.033000
H17E	0.3516	1.2271	0.4186	0.033000
H17F	0.4166	1.3553	0.4253	0.033000
H18D	0.7785	1.0450	0.4781	0.033000
H18E	0.7129	1.0913	0.5106	0.033000
H18F	0.7334	1.2291	0.4588	0.033000
H19D	0.5022	0.4813	0.3165	0.046000
H19E	0.5589	0.5098	0.3609	0.046000
H19F	0.5644	0.6134	0.3010	0.046000

Table S19. Hydrogen Bonds (Angstrom, Deg) for **4e**.

Donor – H…Acceptor [ARU]	D – H	H…A	D…A	D-H…A
C2B – H2B…O1B	0.9500	2.3400	2.709(3)	103.00
C6A – H6A…O1A	0.9500	2.3400	2.713(3)	103.00
C10A – H10B…N1A	0.9800	2.5300	2.867(3)	100.00
C13B – H13B…O4A [x,1+y,z]	0.9500	2.4600	3.382(3)	164.00
C17A – H17A…O2B	0.9800	2.5600	3.392(3)	143.00

M. NMR Spectra

M.1. NMR spectra of azlactones 1

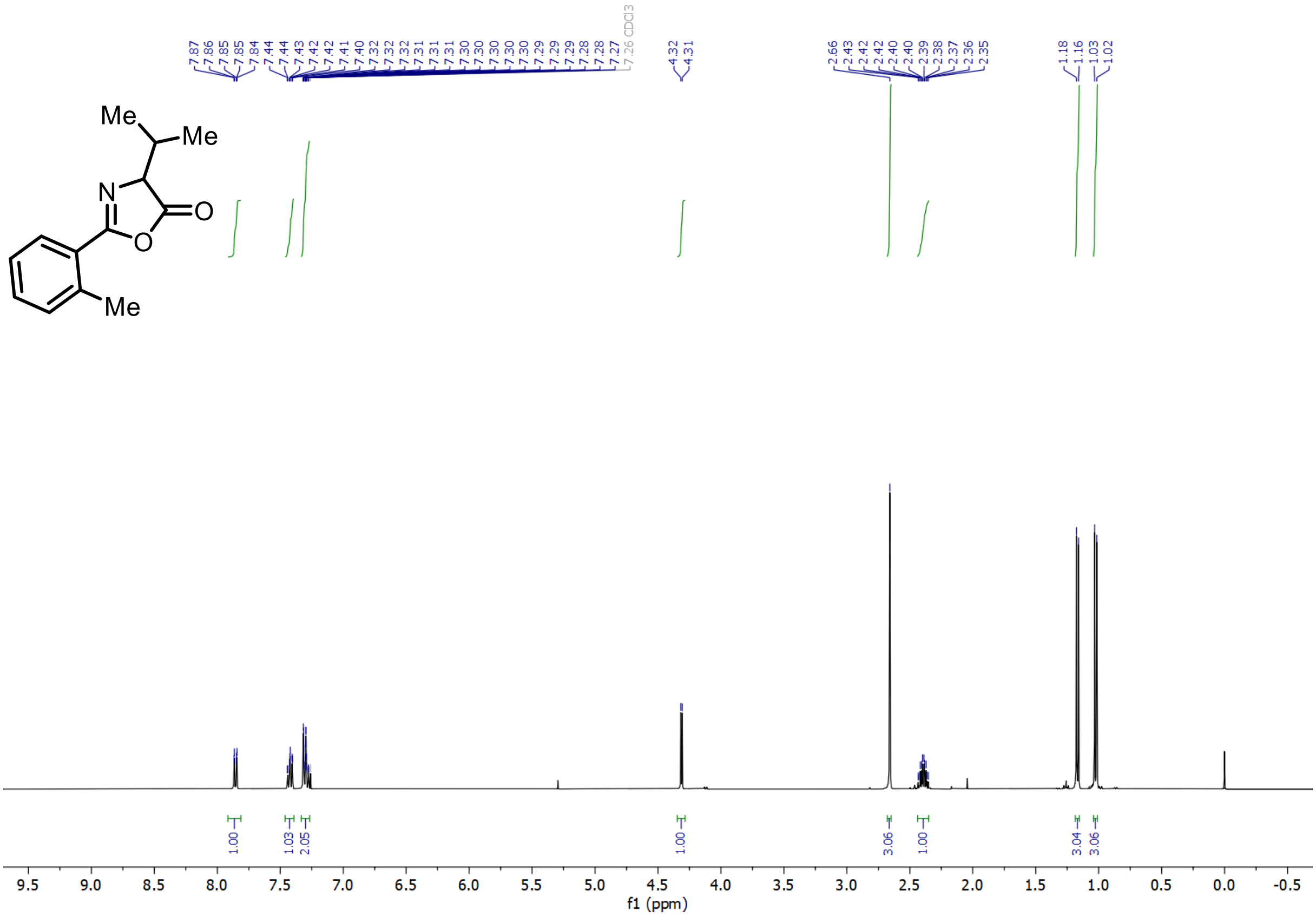

^{1}H NMR spectra of **1d**.

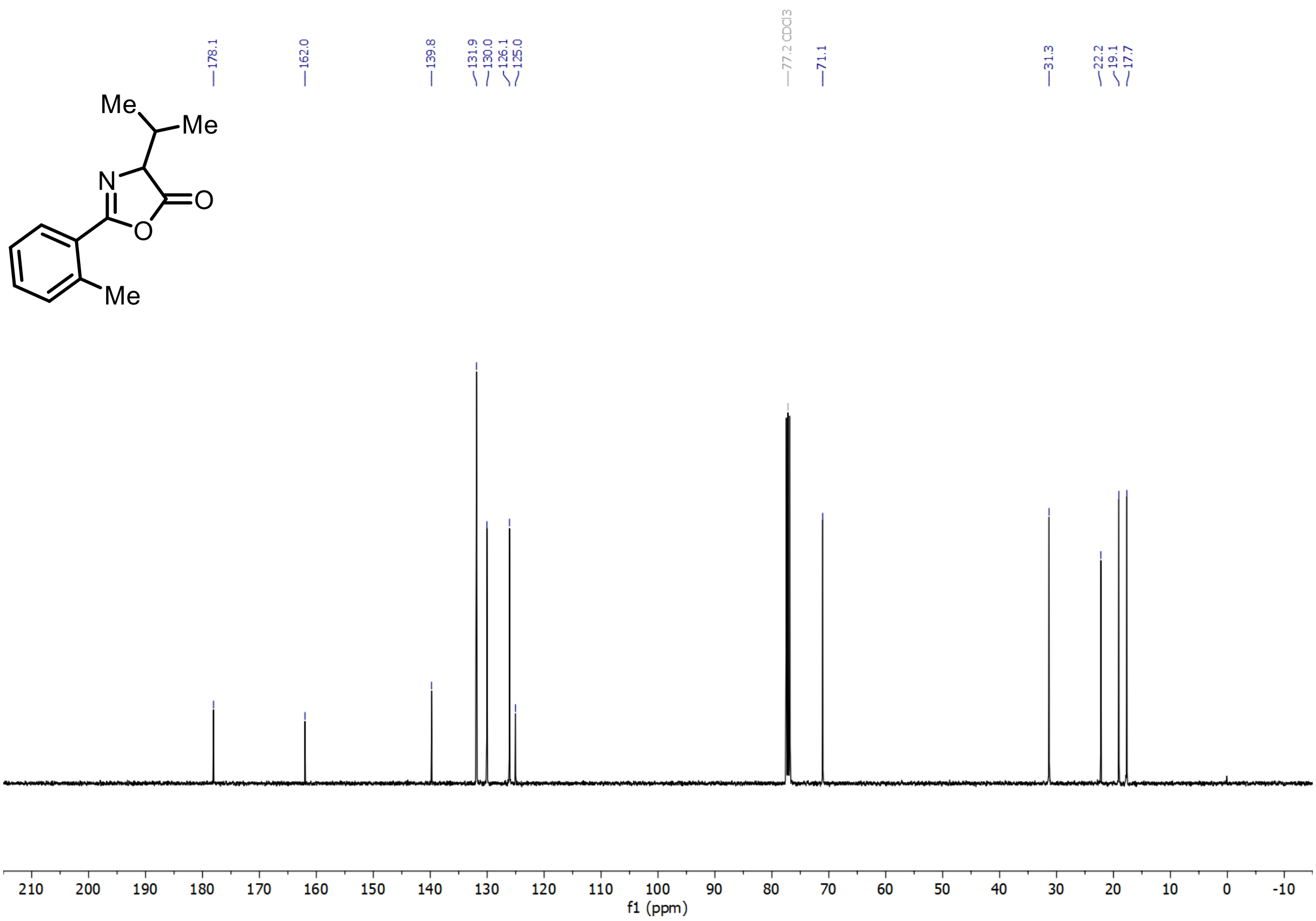

^{13}C NMR spectra of **1d**.

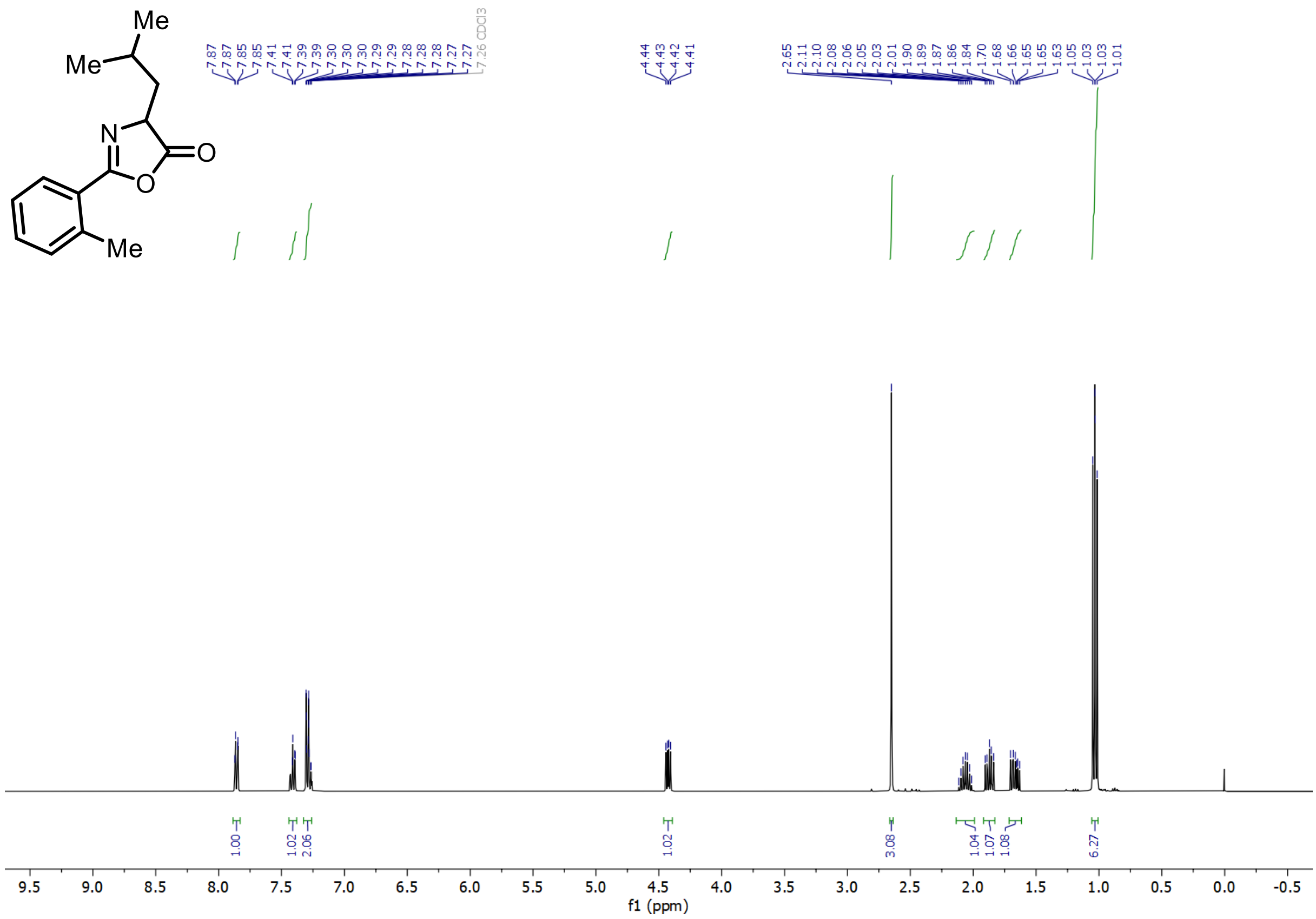

^{1}H NMR spectra of **1f**.

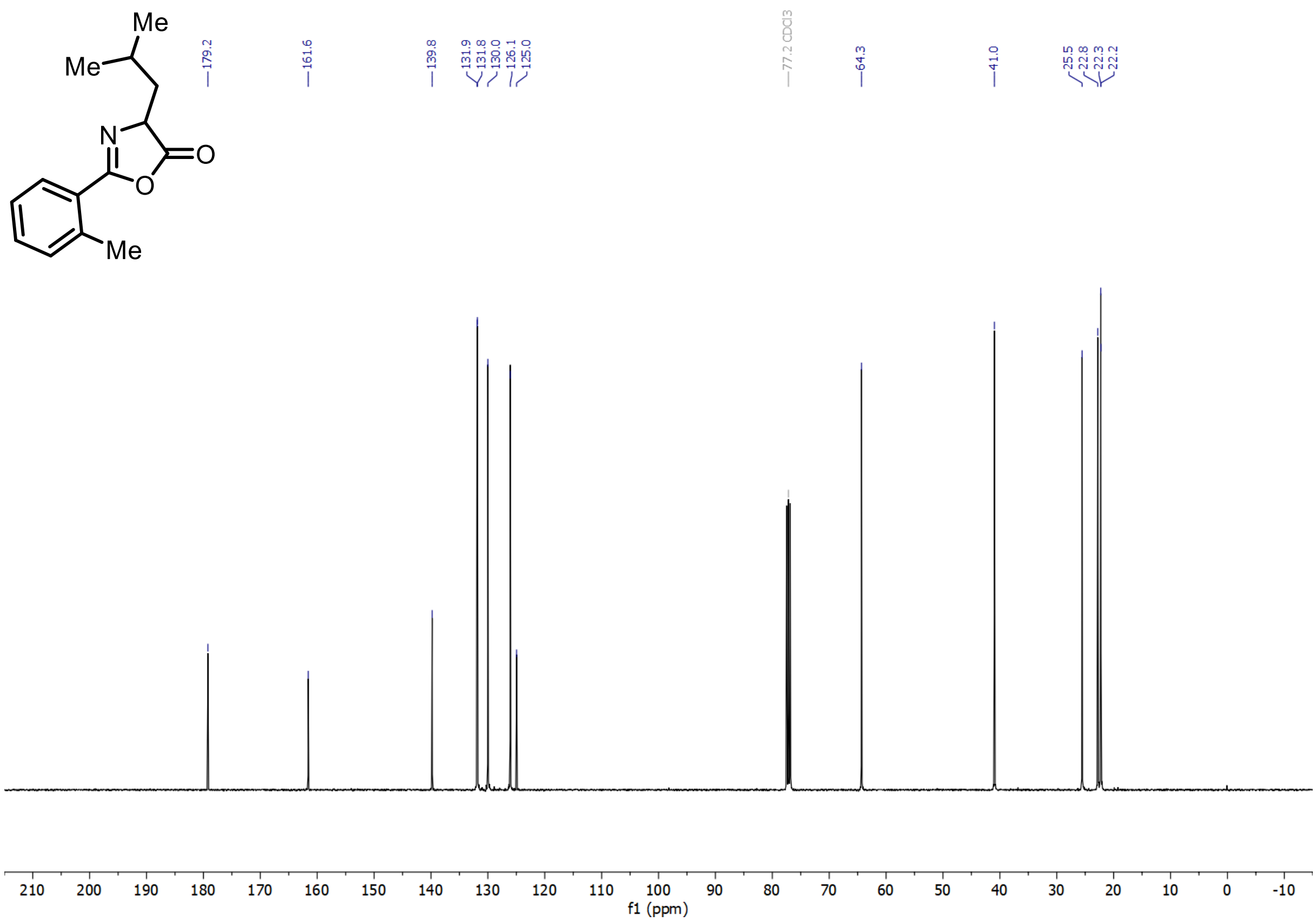

^{13}C NMR spectra of **1f**.

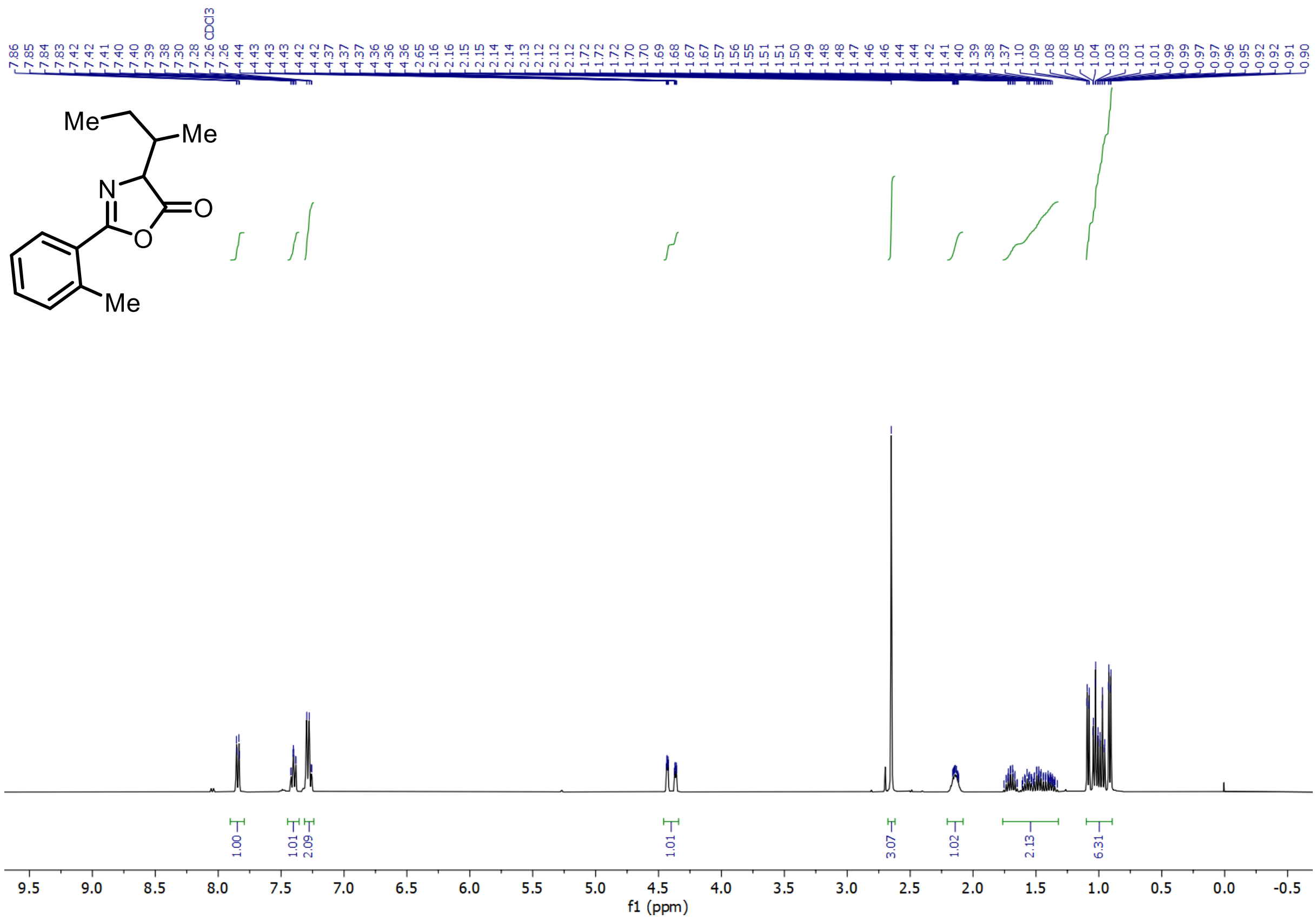

^{1}H NMR spectra of **1g**.

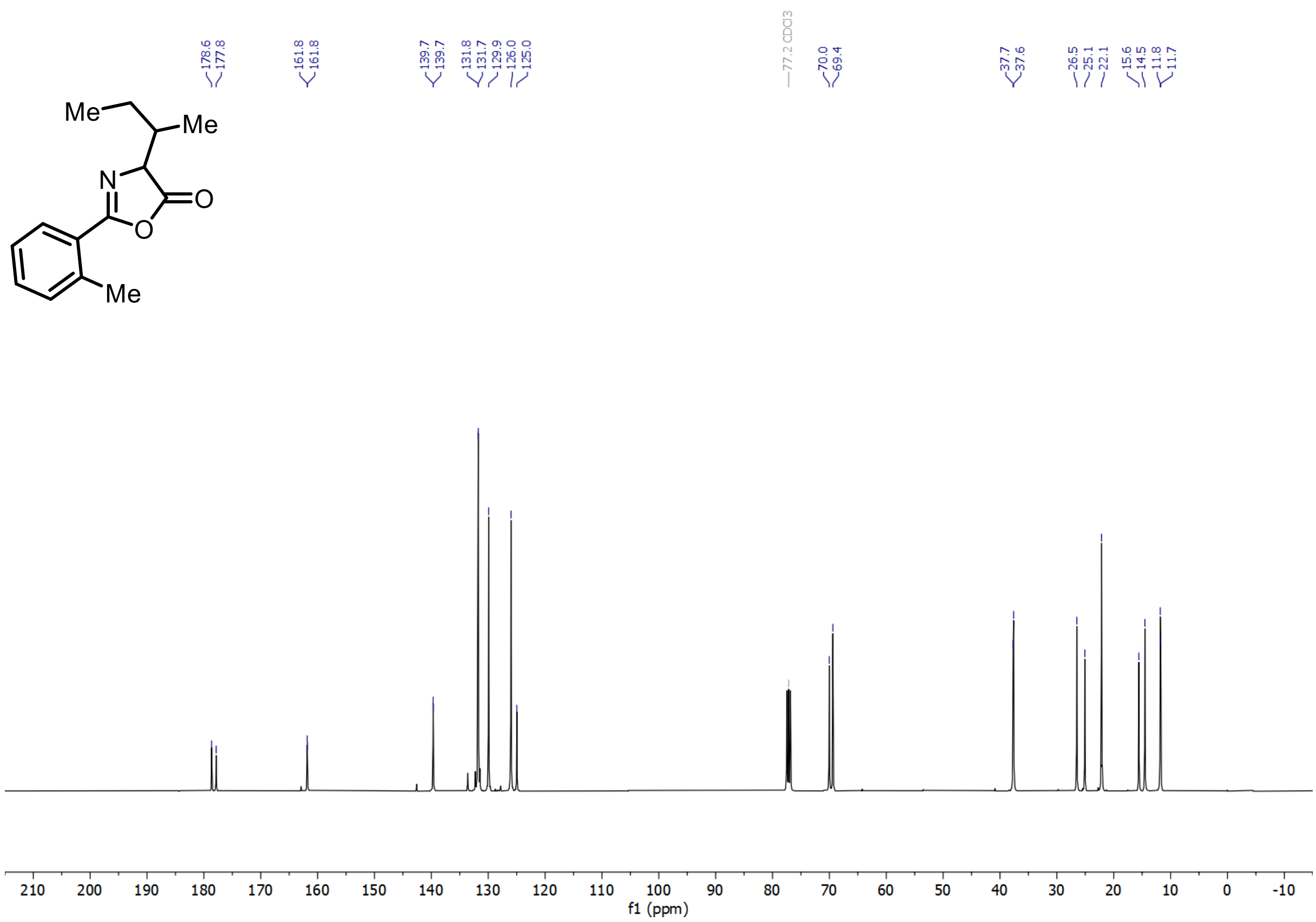

^{13}C NMR spectra of **1g**.

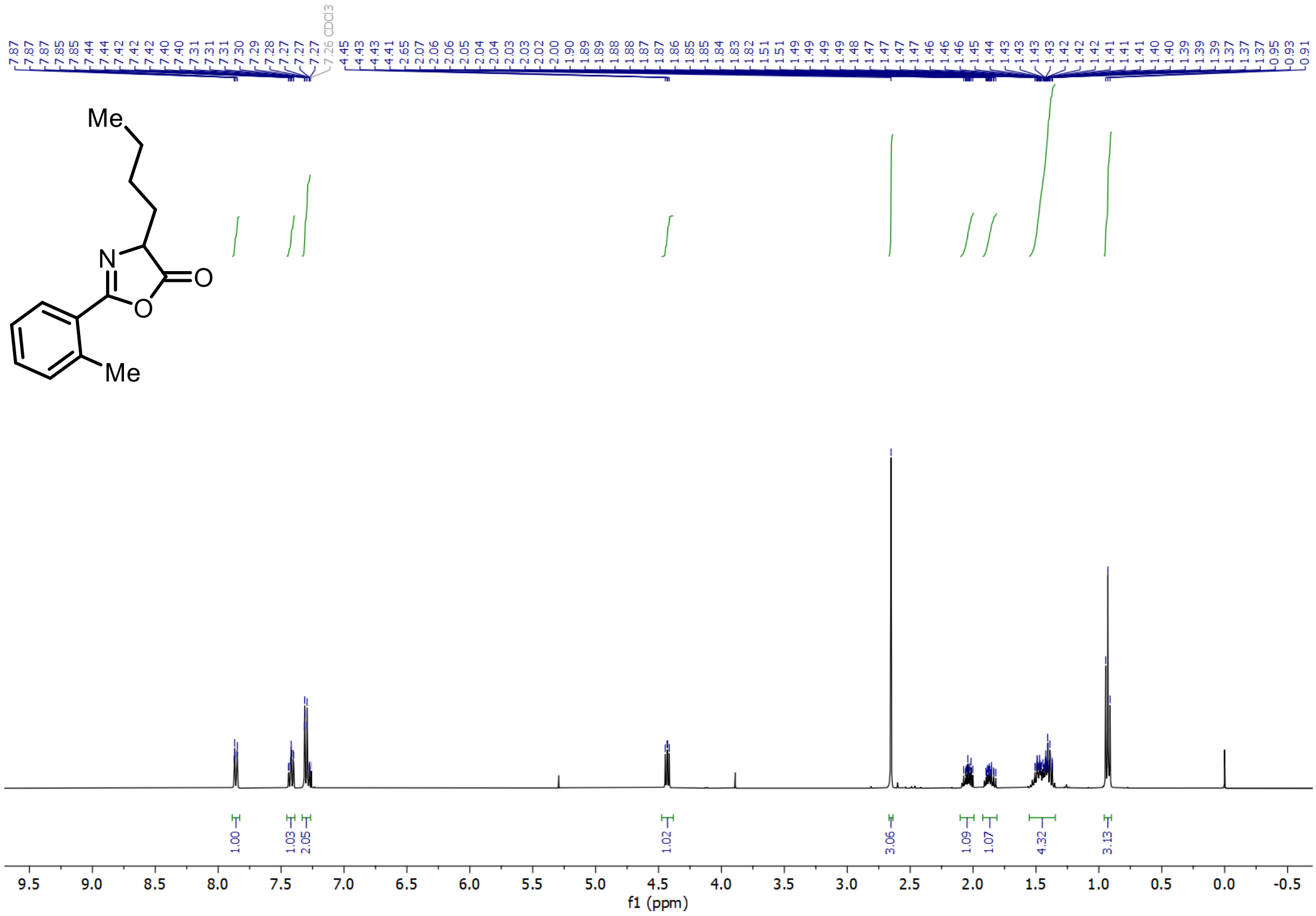

^{1}H NMR spectra of **1h**.

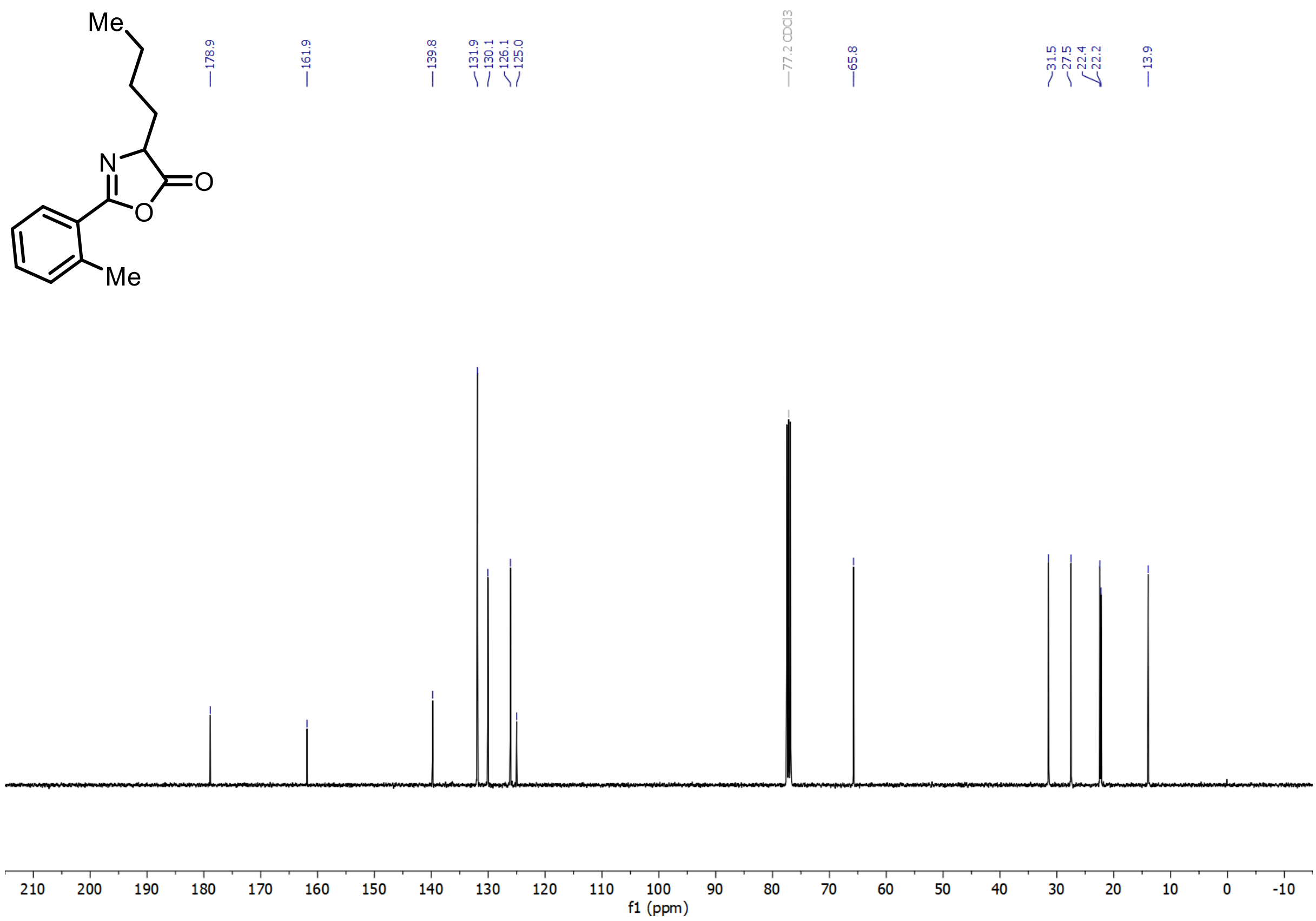

^{13}C NMR spectra of **1h**.

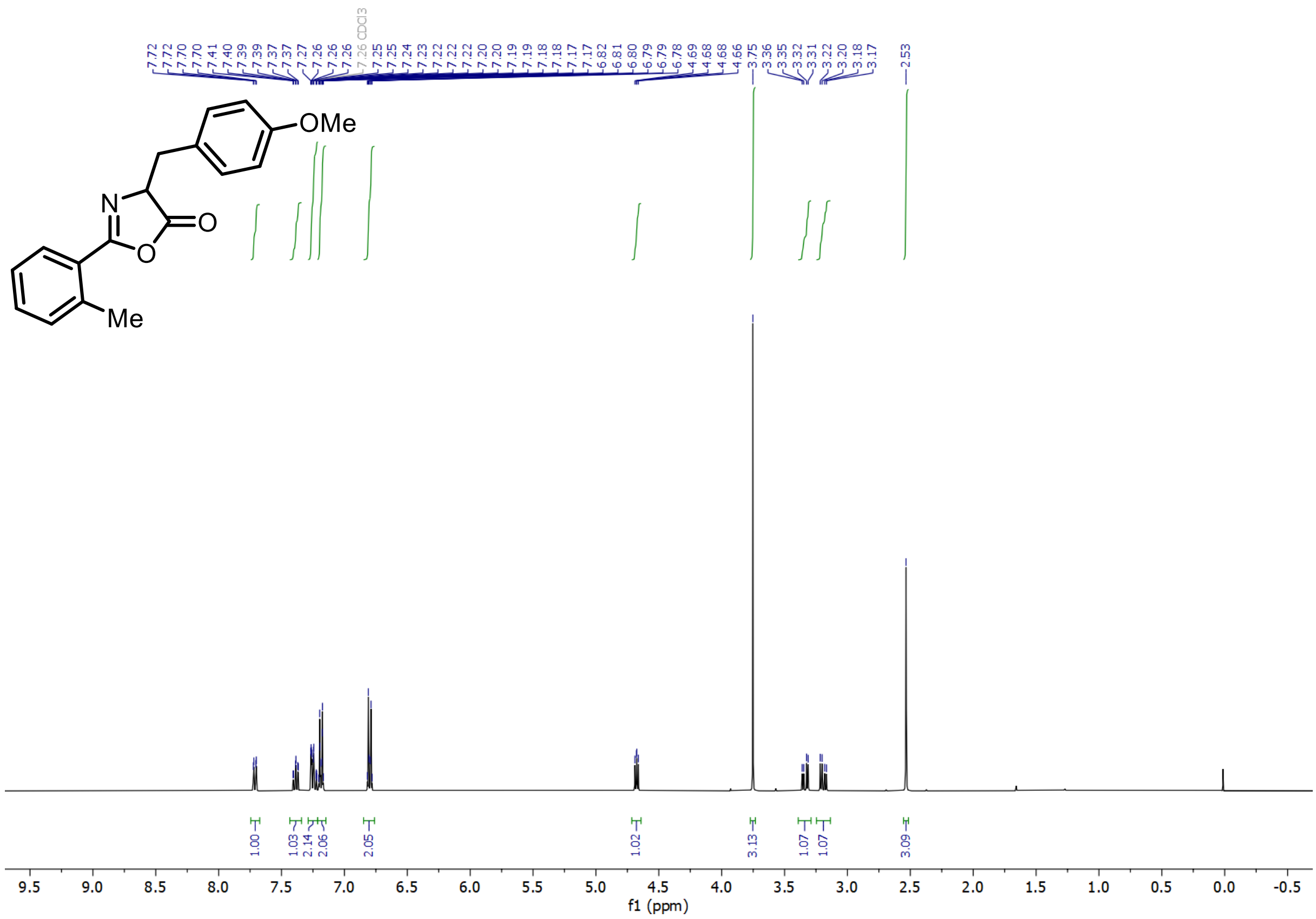

^{1}H NMR spectra of **1j**.

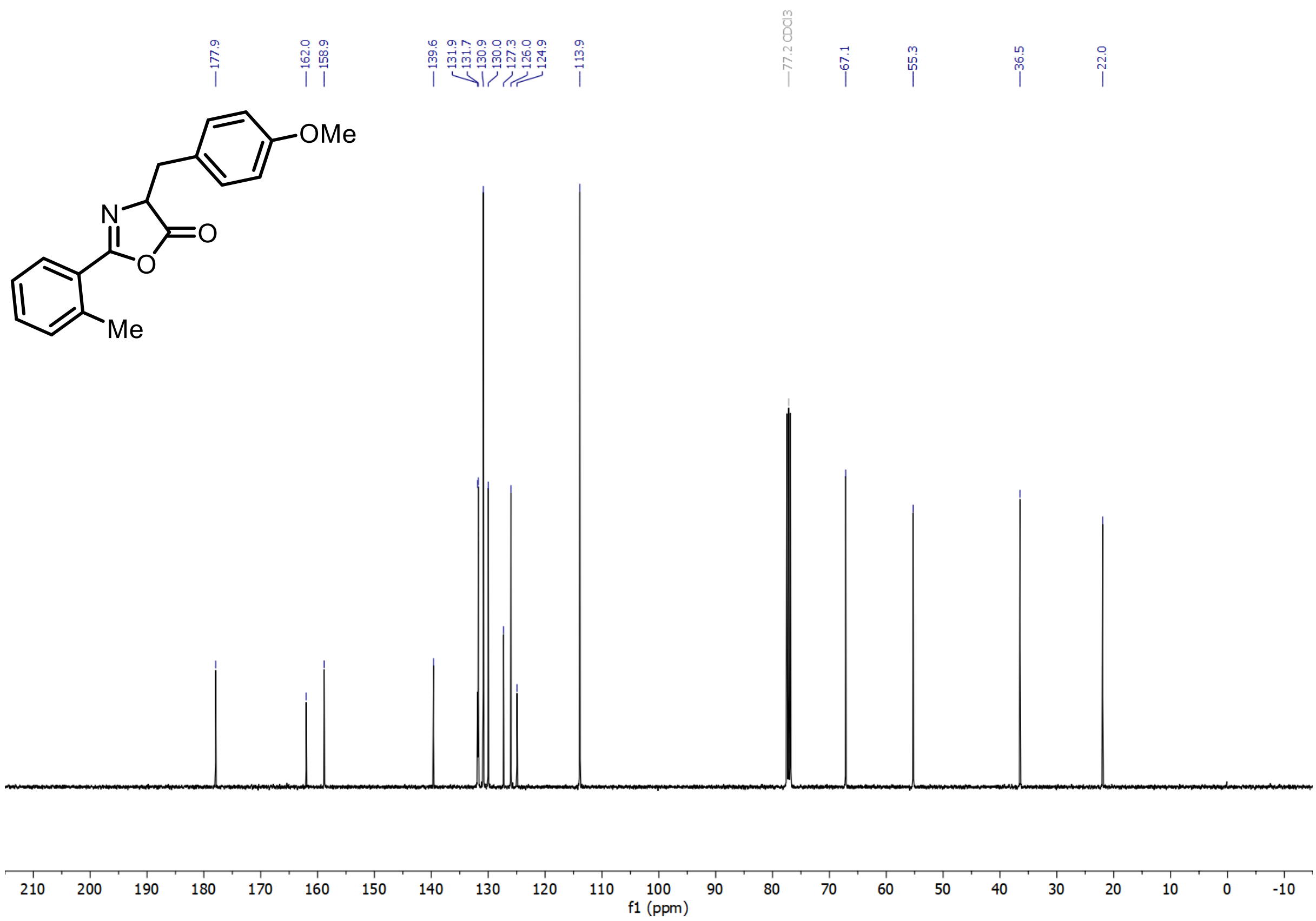

^{13}C NMR spectra of **1j**.

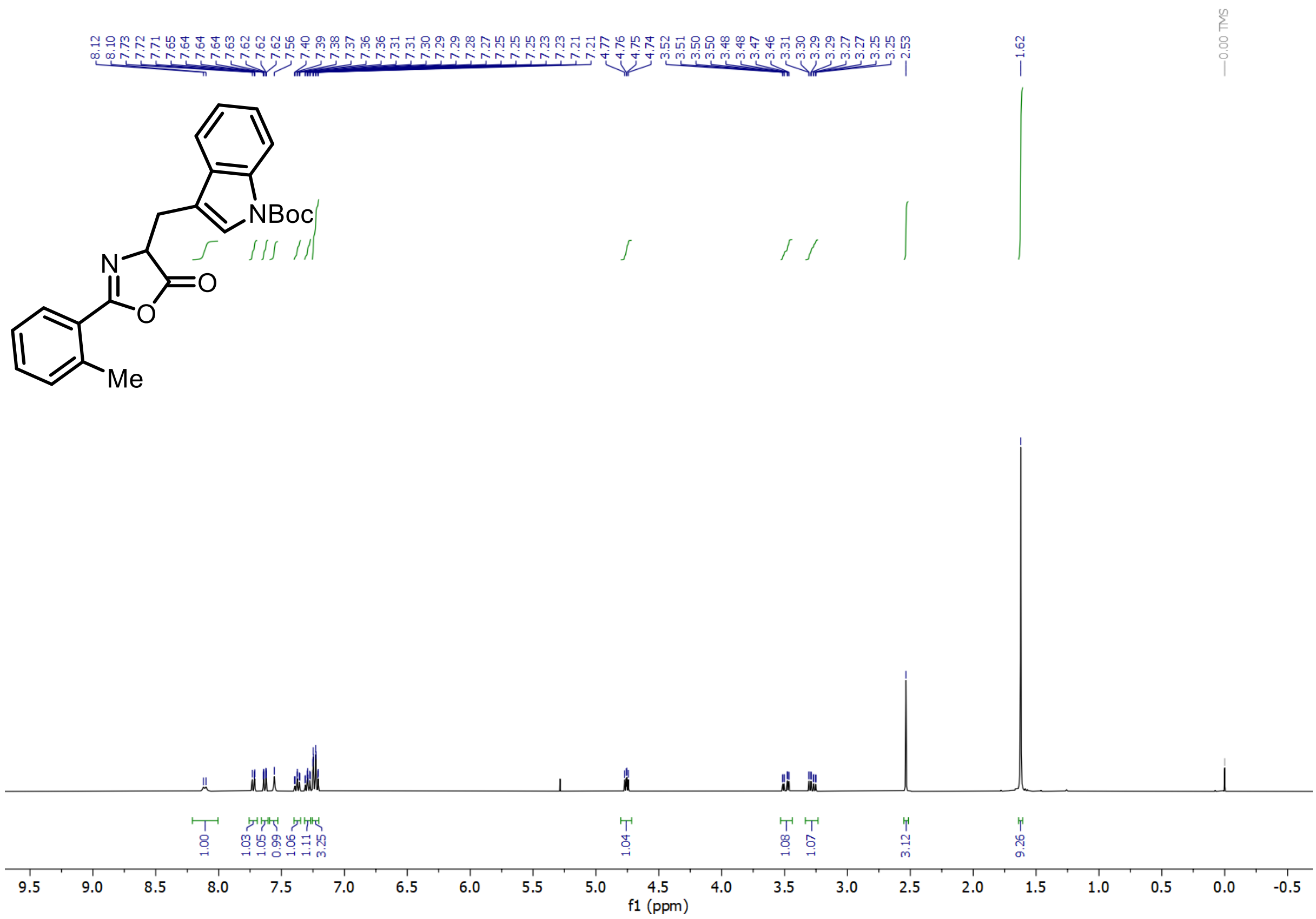

^{1}H NMR spectra of **1k**.

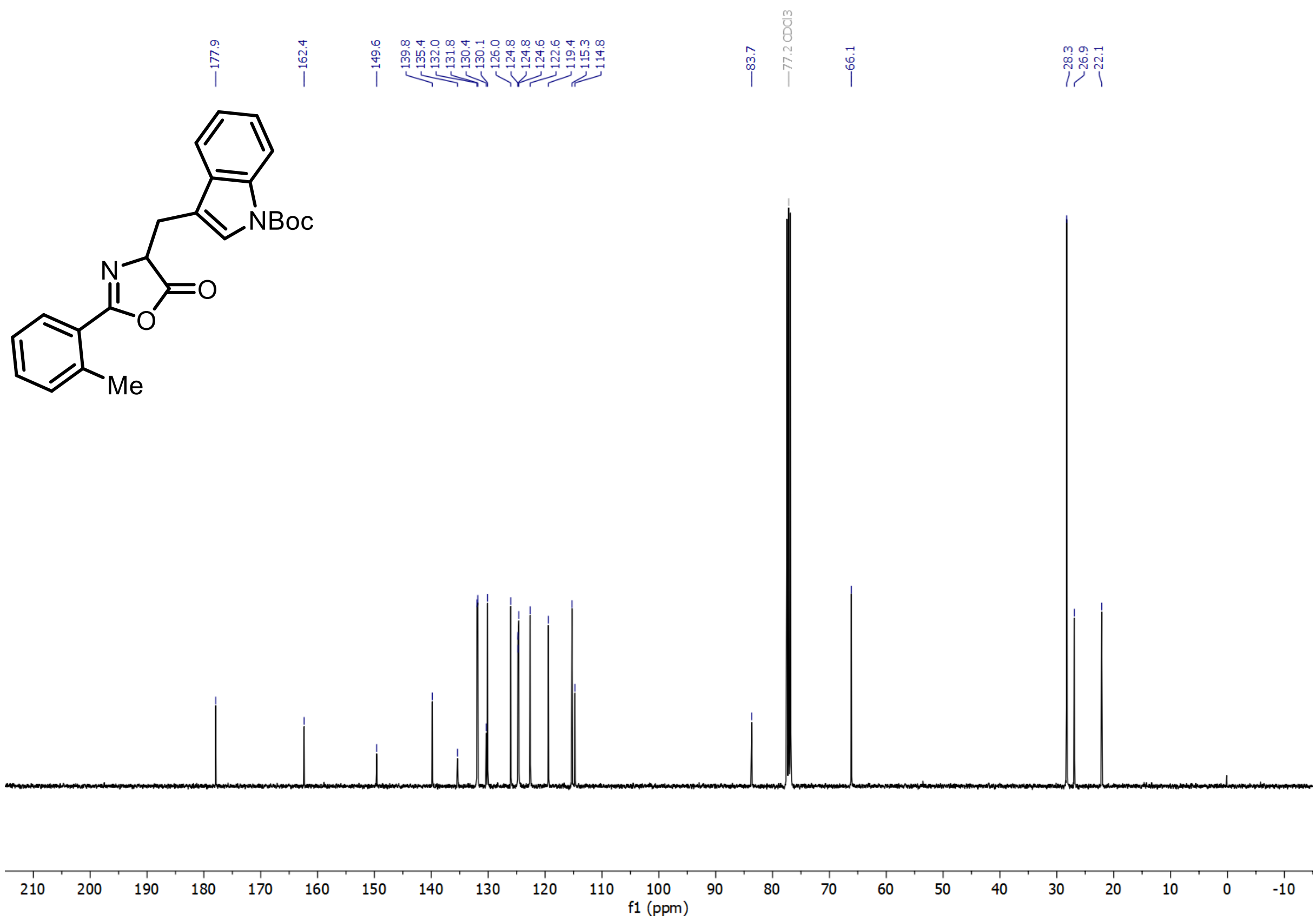

^{13}C NMR spectra of **1k**.

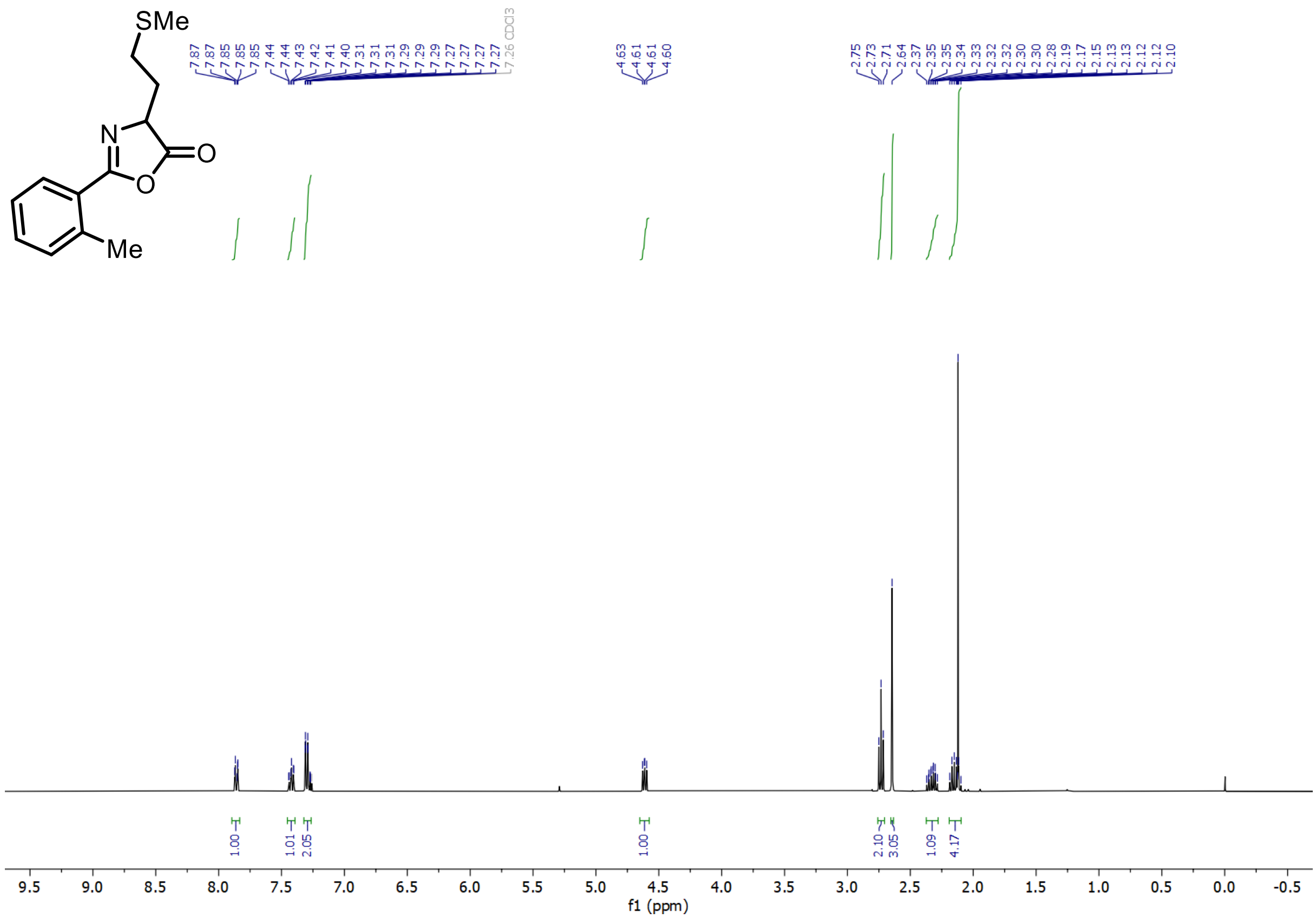

^{1}H NMR spectra of **1l**.

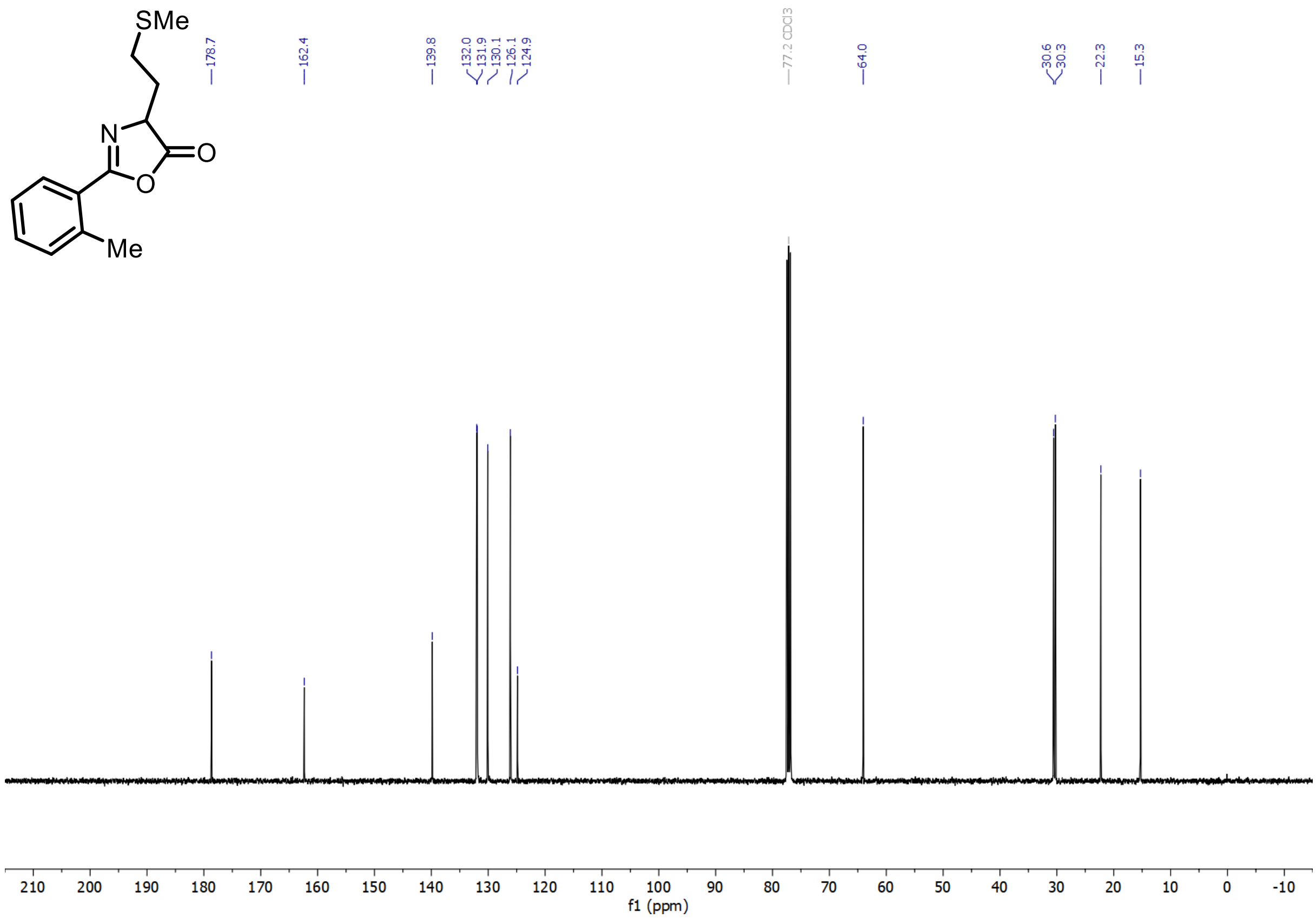

^{13}C NMR spectra of **1l**.

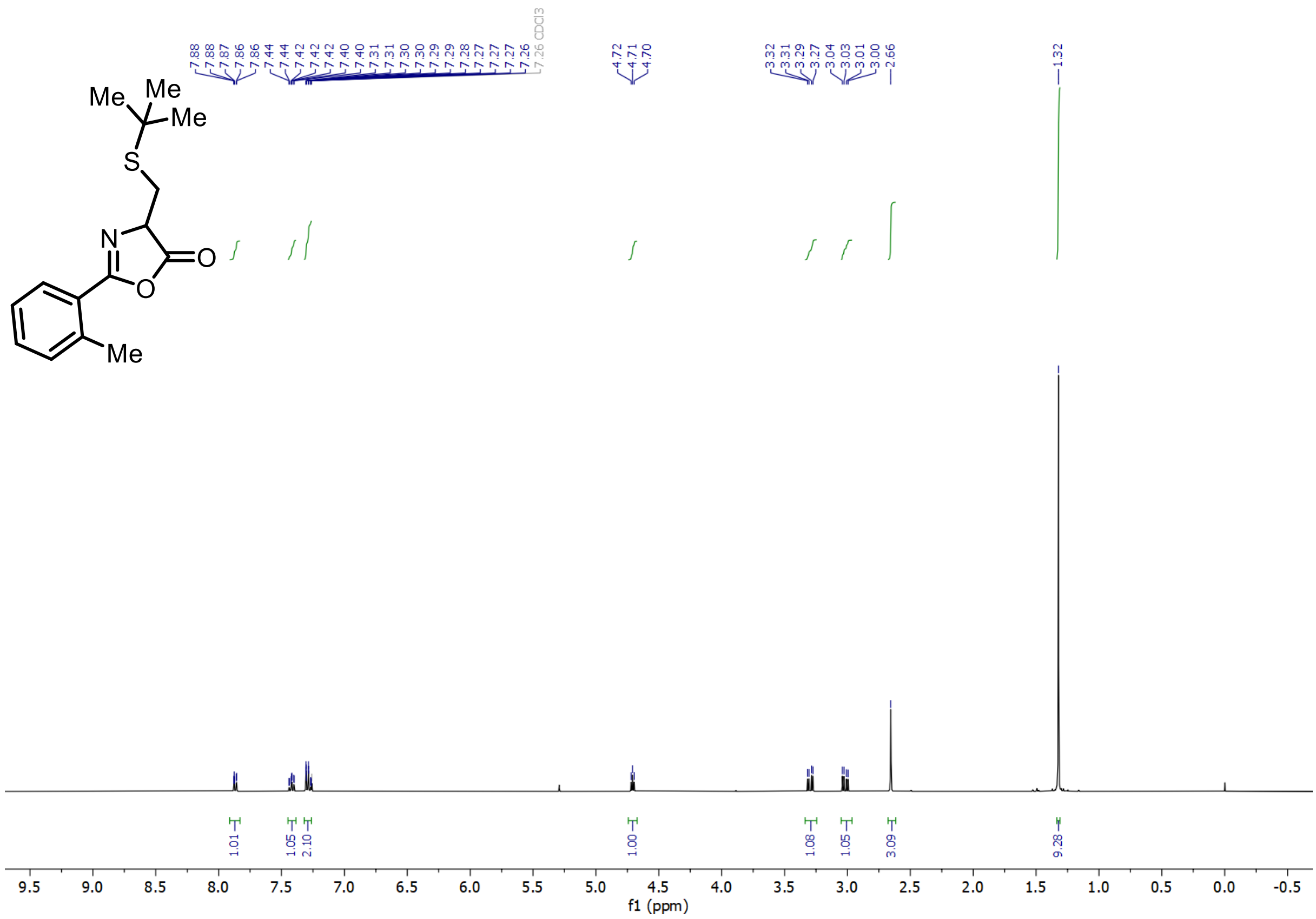

^{1}H NMR spectra of **1m**.

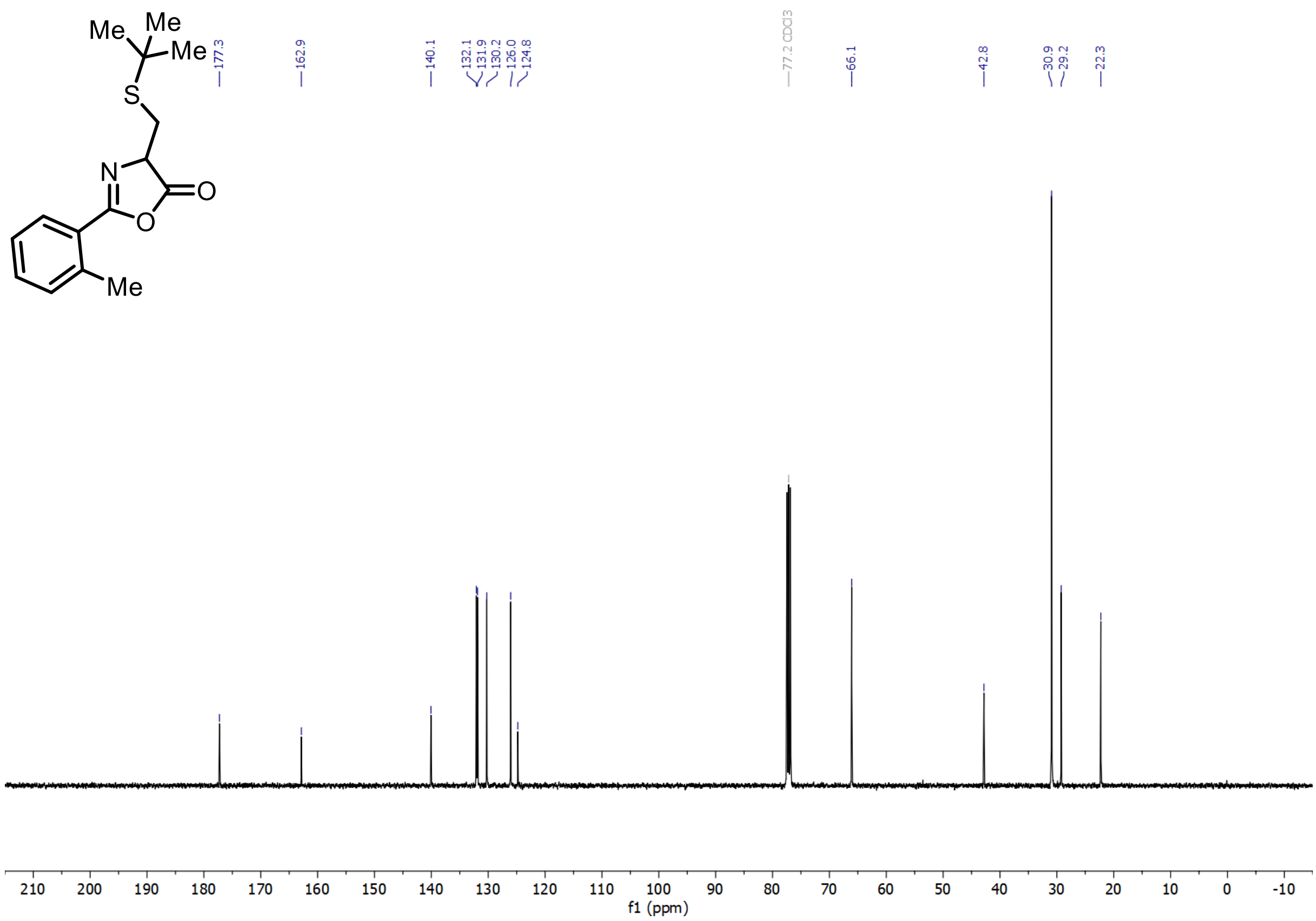

^{13}C NMR spectra of **1m**.

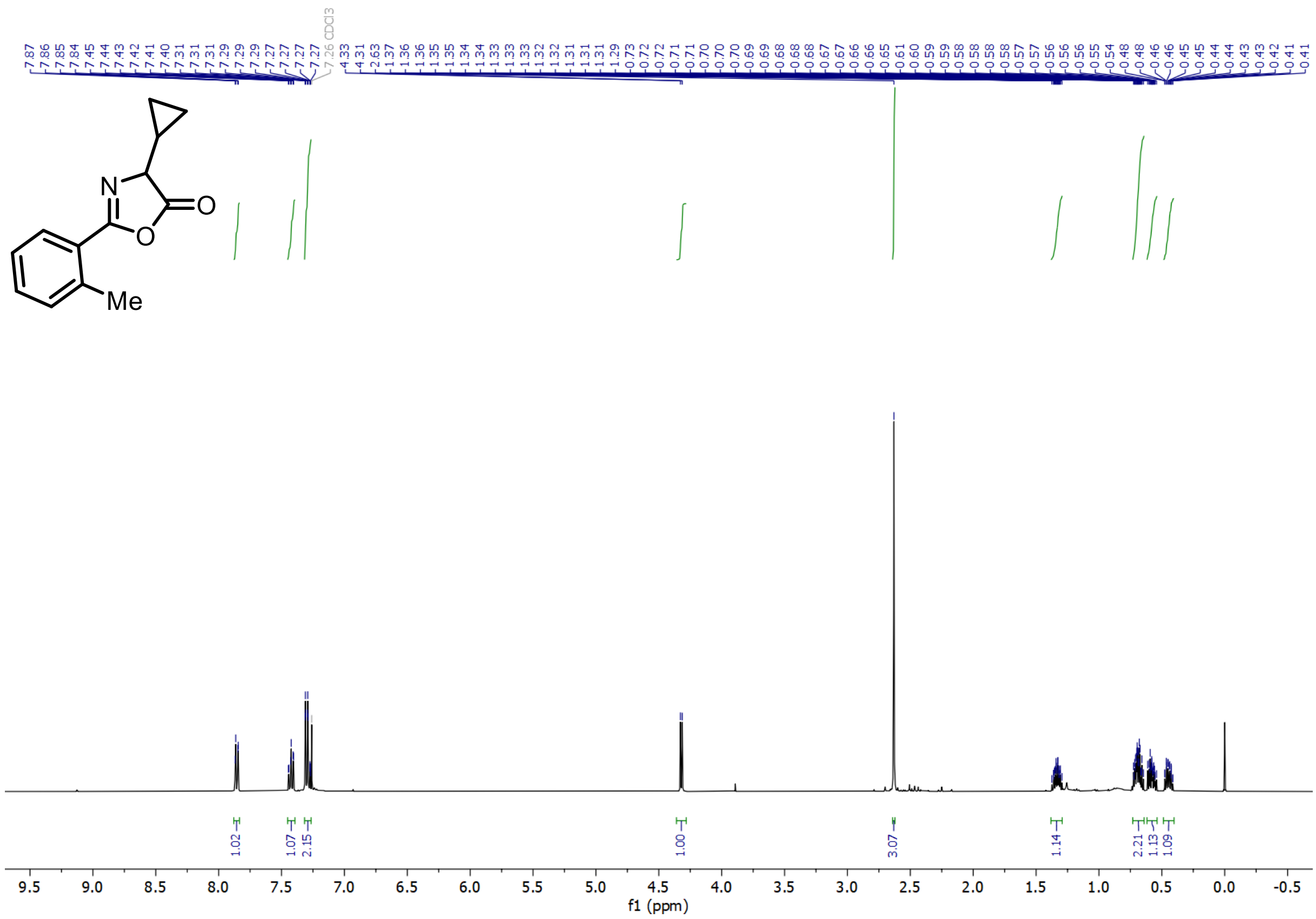

^{1}H NMR spectra of **1o**.

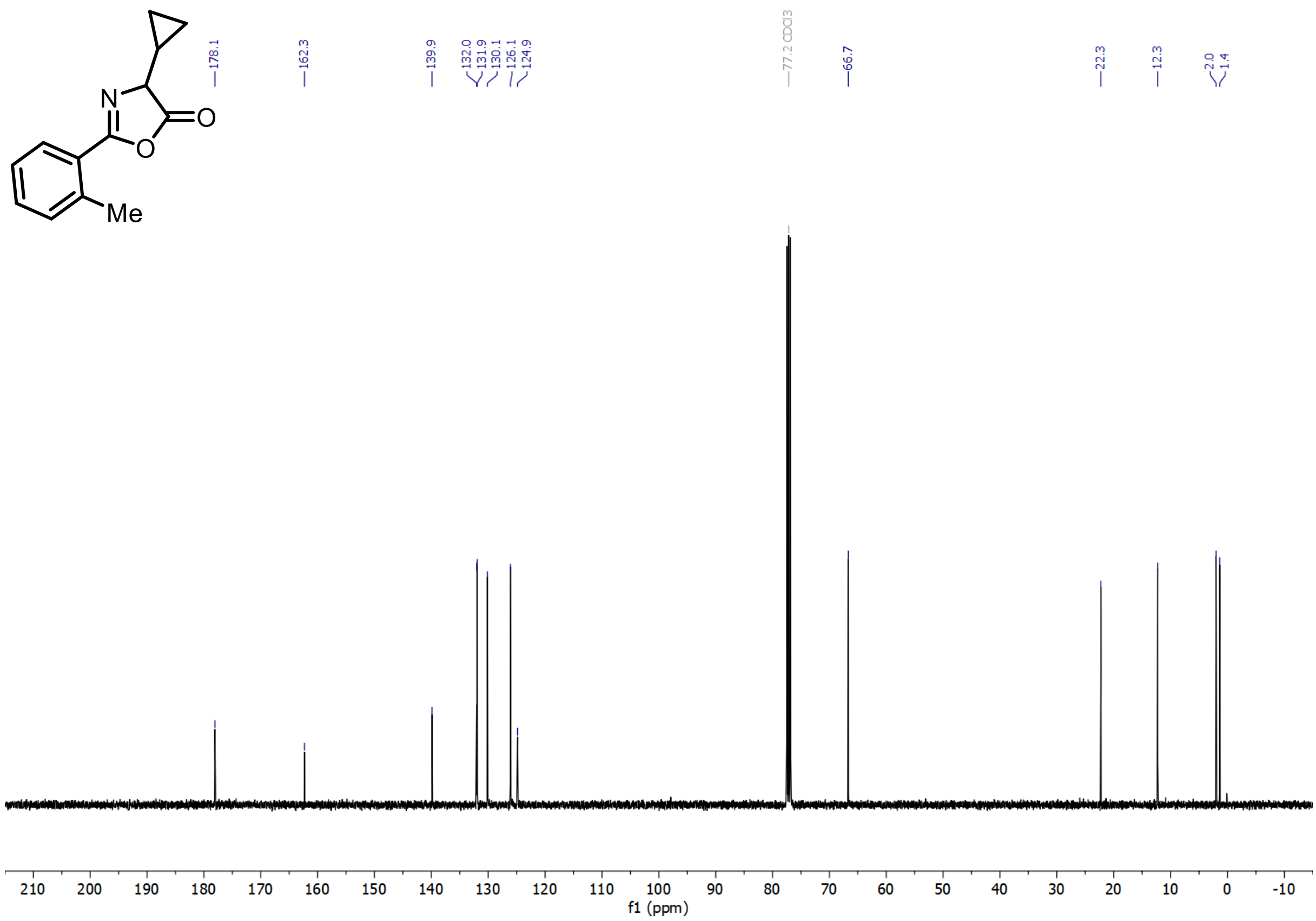

^{13}C NMR spectra of **1o**.

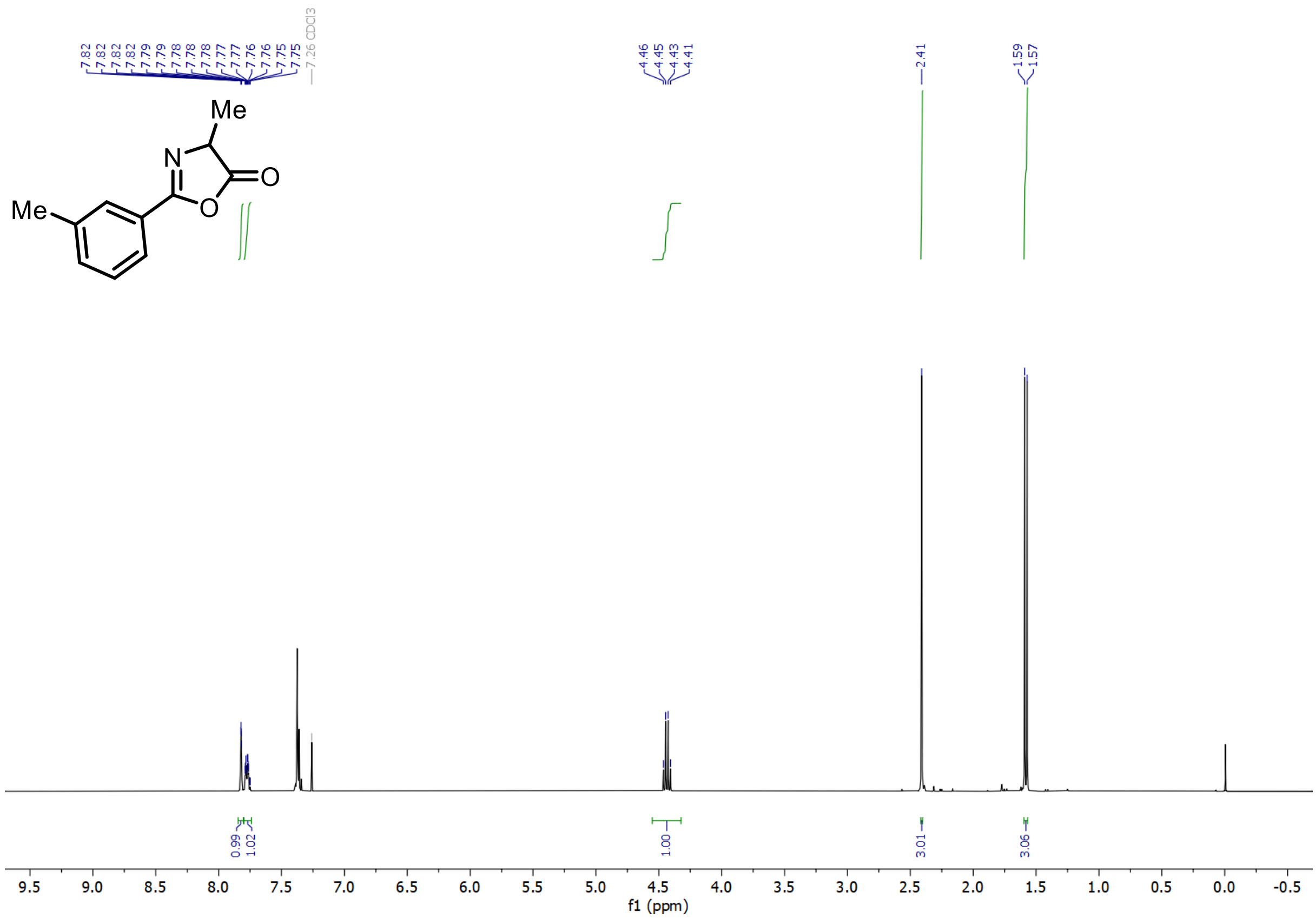

^{1}H NMR spectra of **1r**.

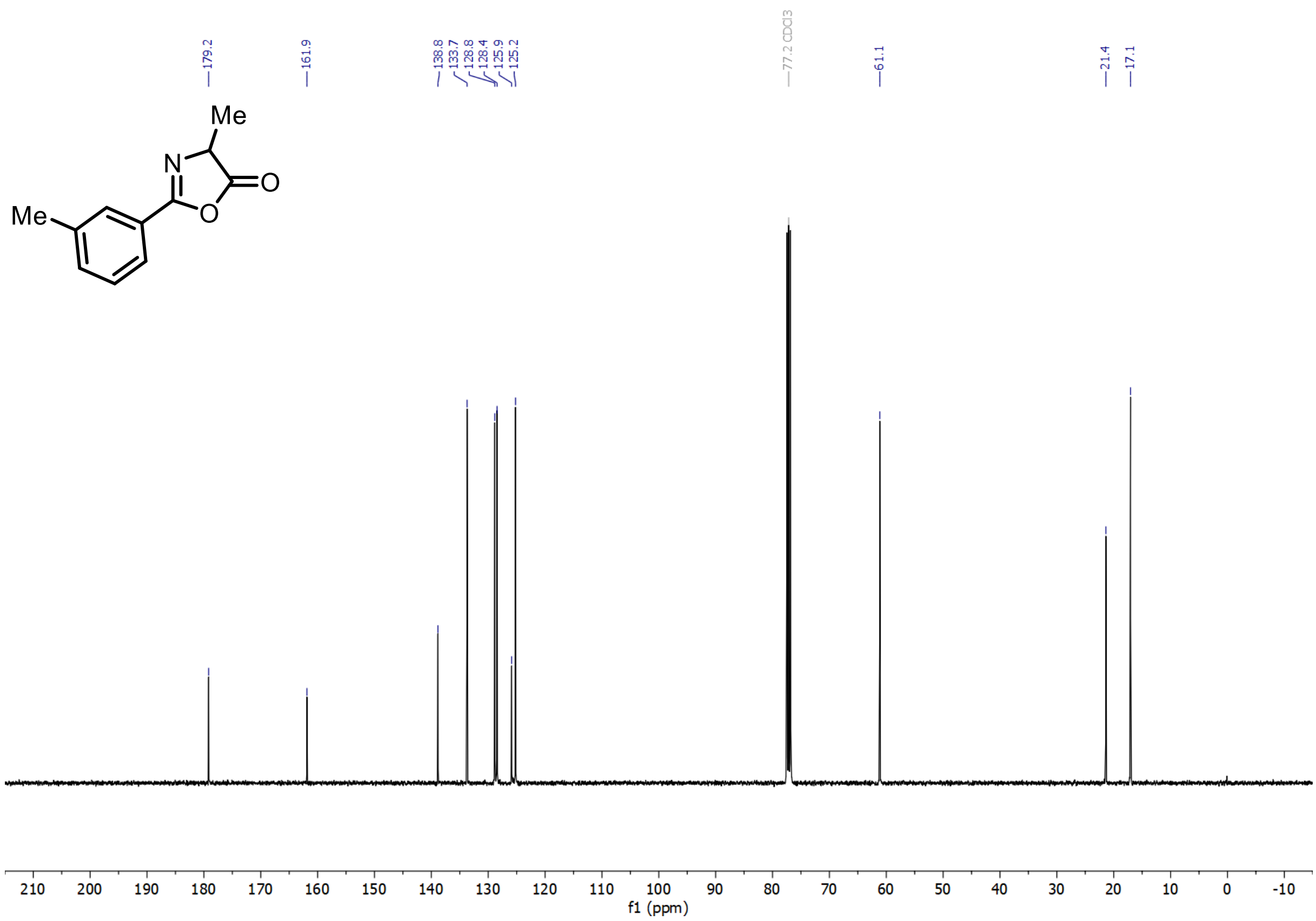

^{13}C NMR spectra of **1r**.

M.2. NMR spectra of triarylamine electrocatalysts 3

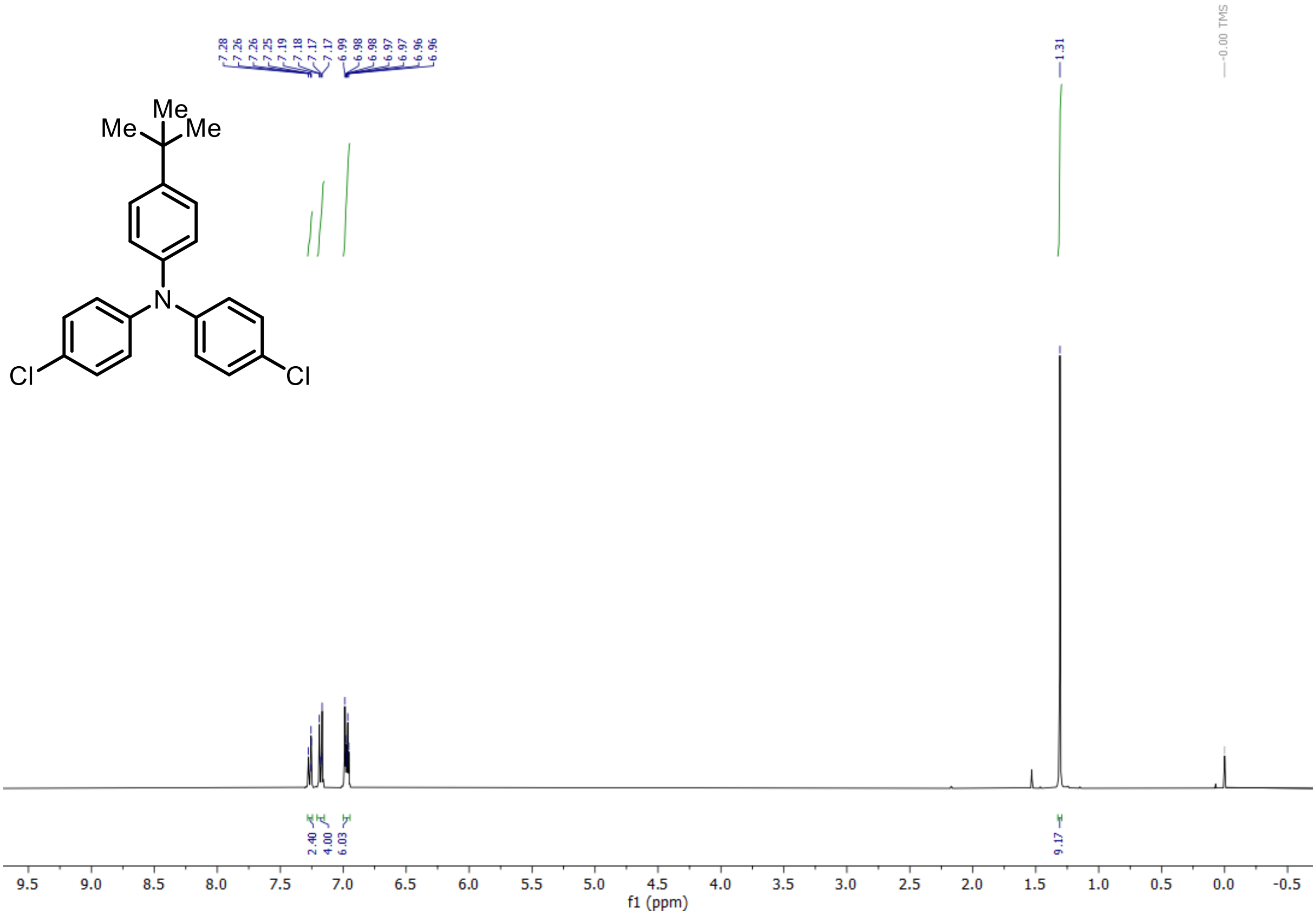

^{1}H NMR spectra of **3b**.

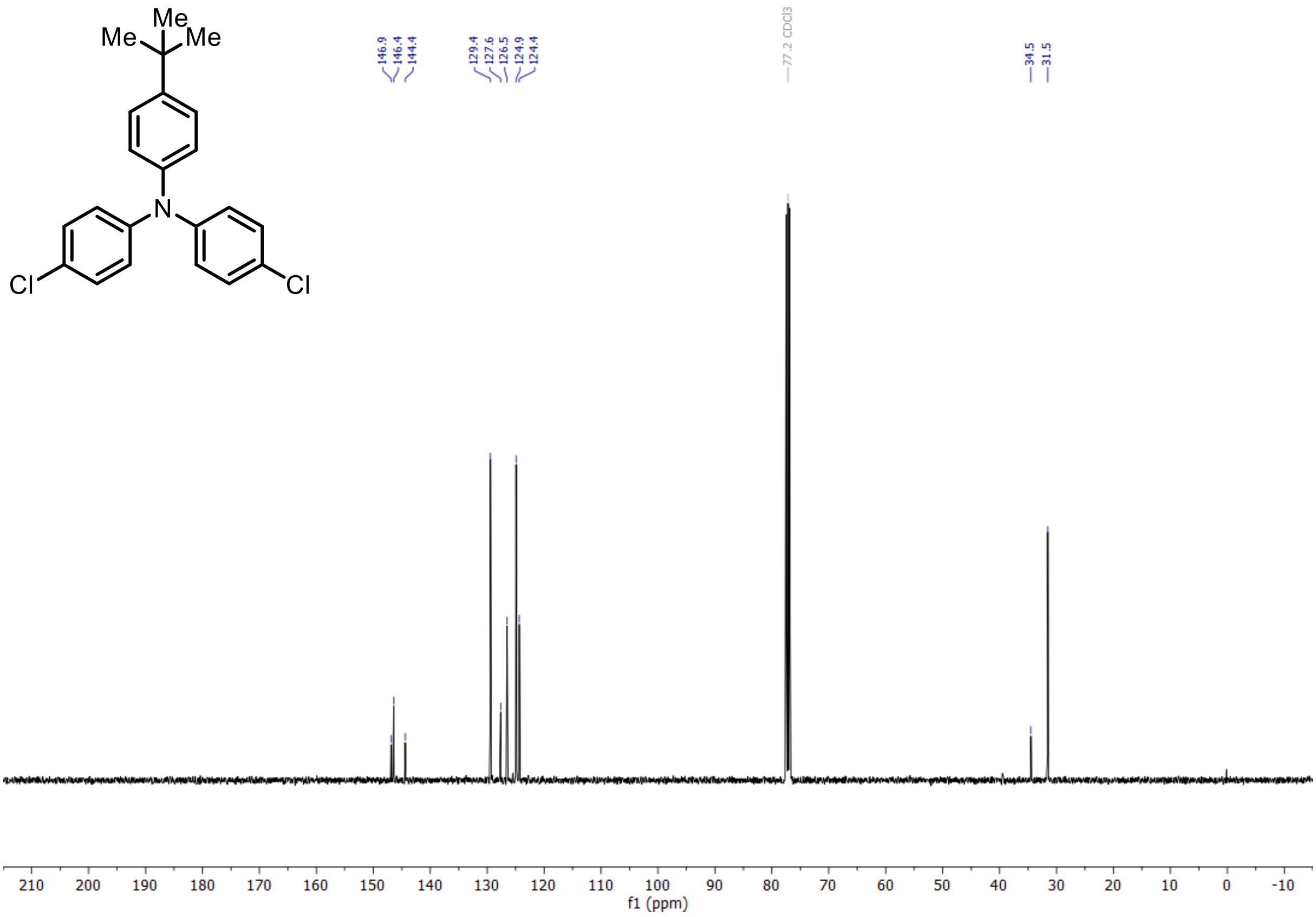

^{13}C NMR spectra of **3b**.

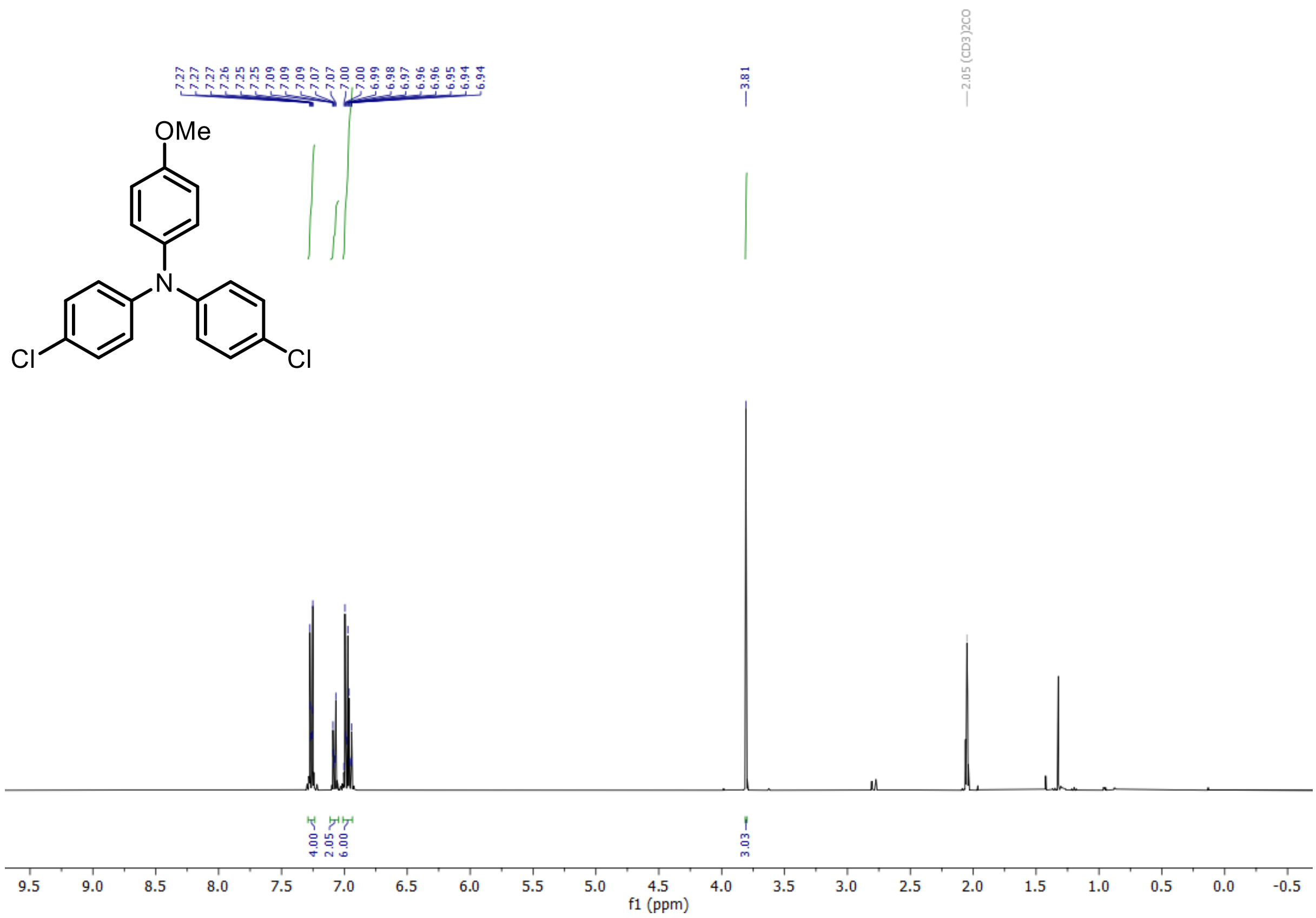

^{1}H NMR spectra of **3c**.

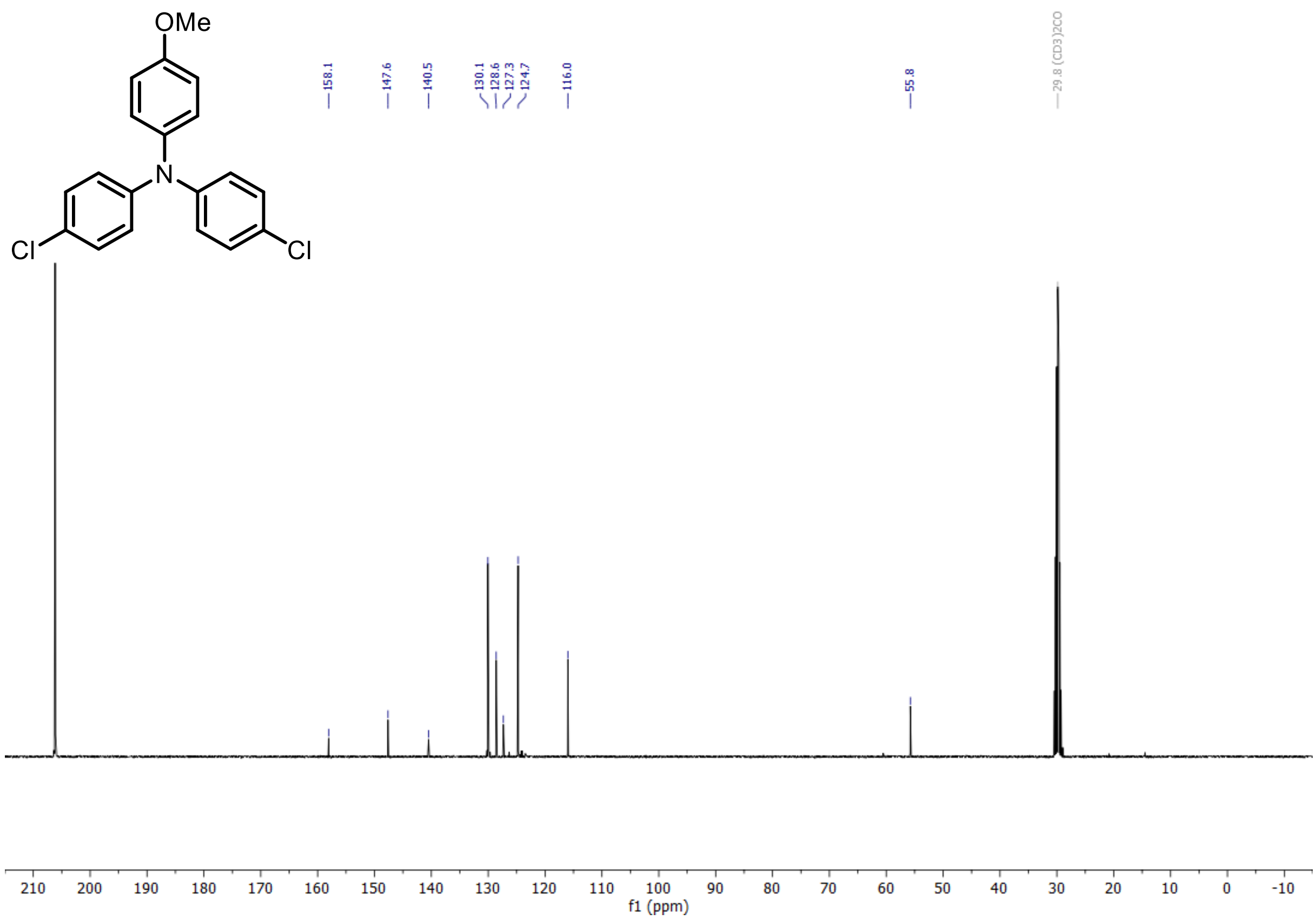

^{13}C NMR spectra of **3c**.

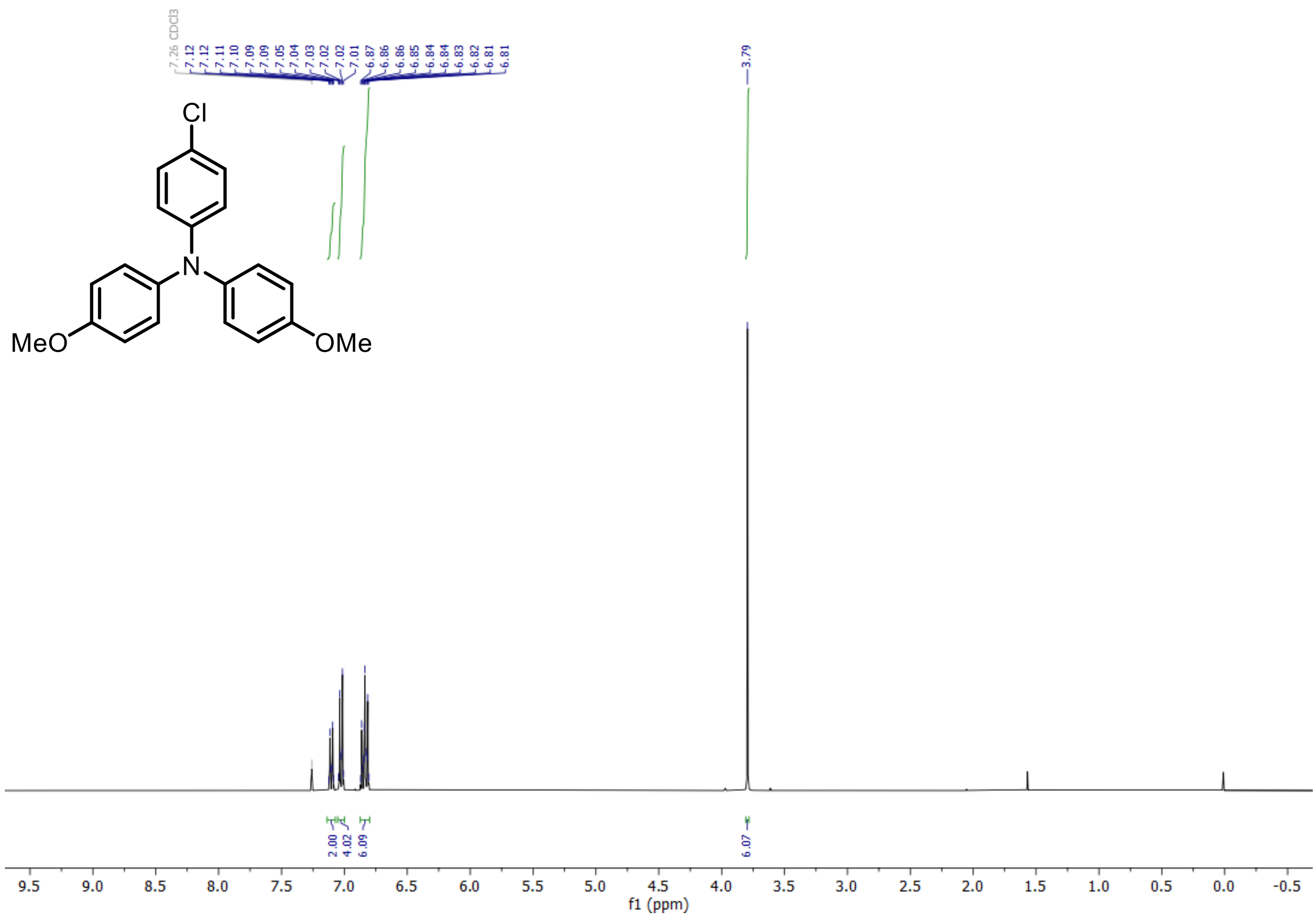

^{1}H NMR spectra of **3d**.

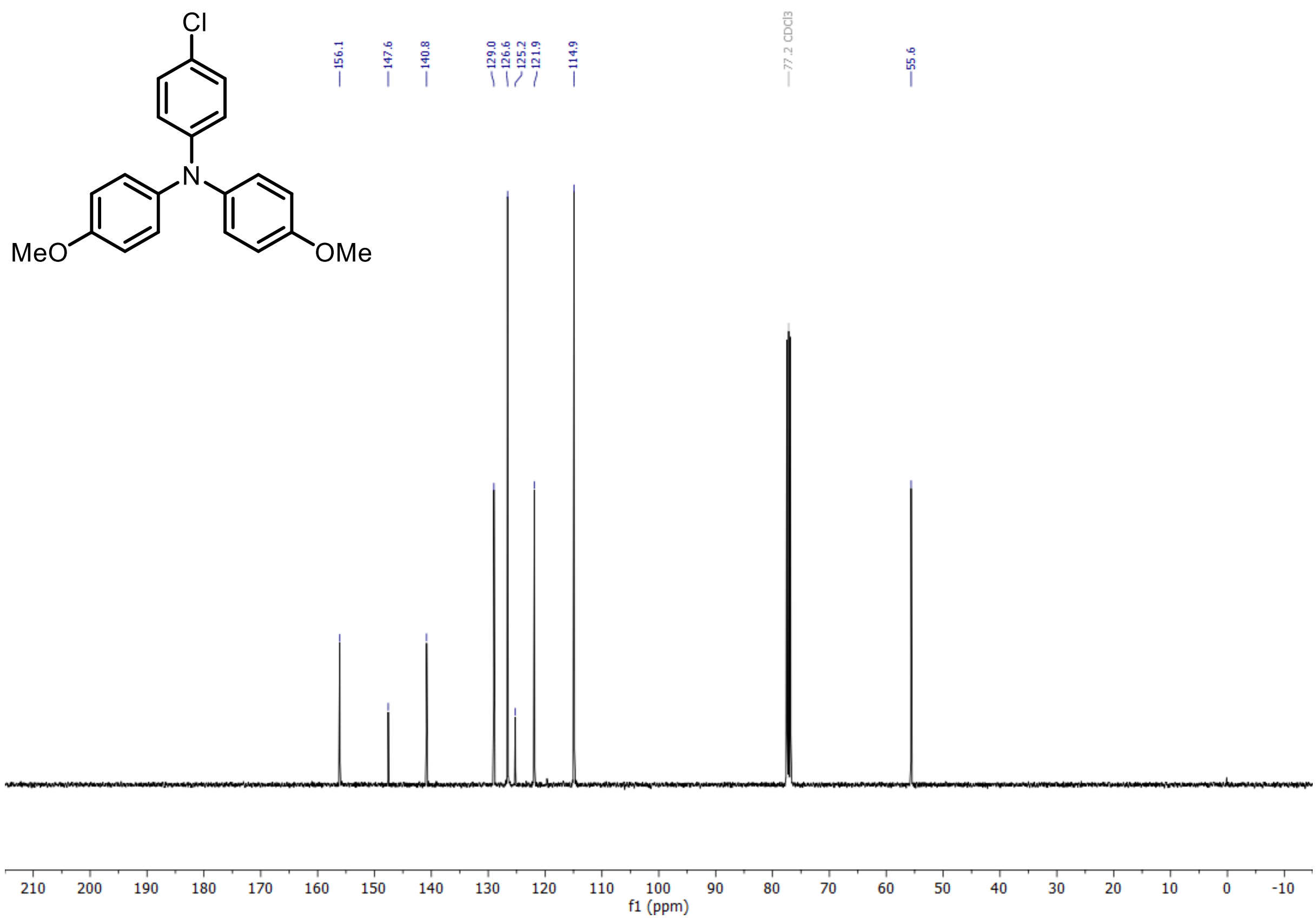

^{13}C NMR spectra of **3d**.

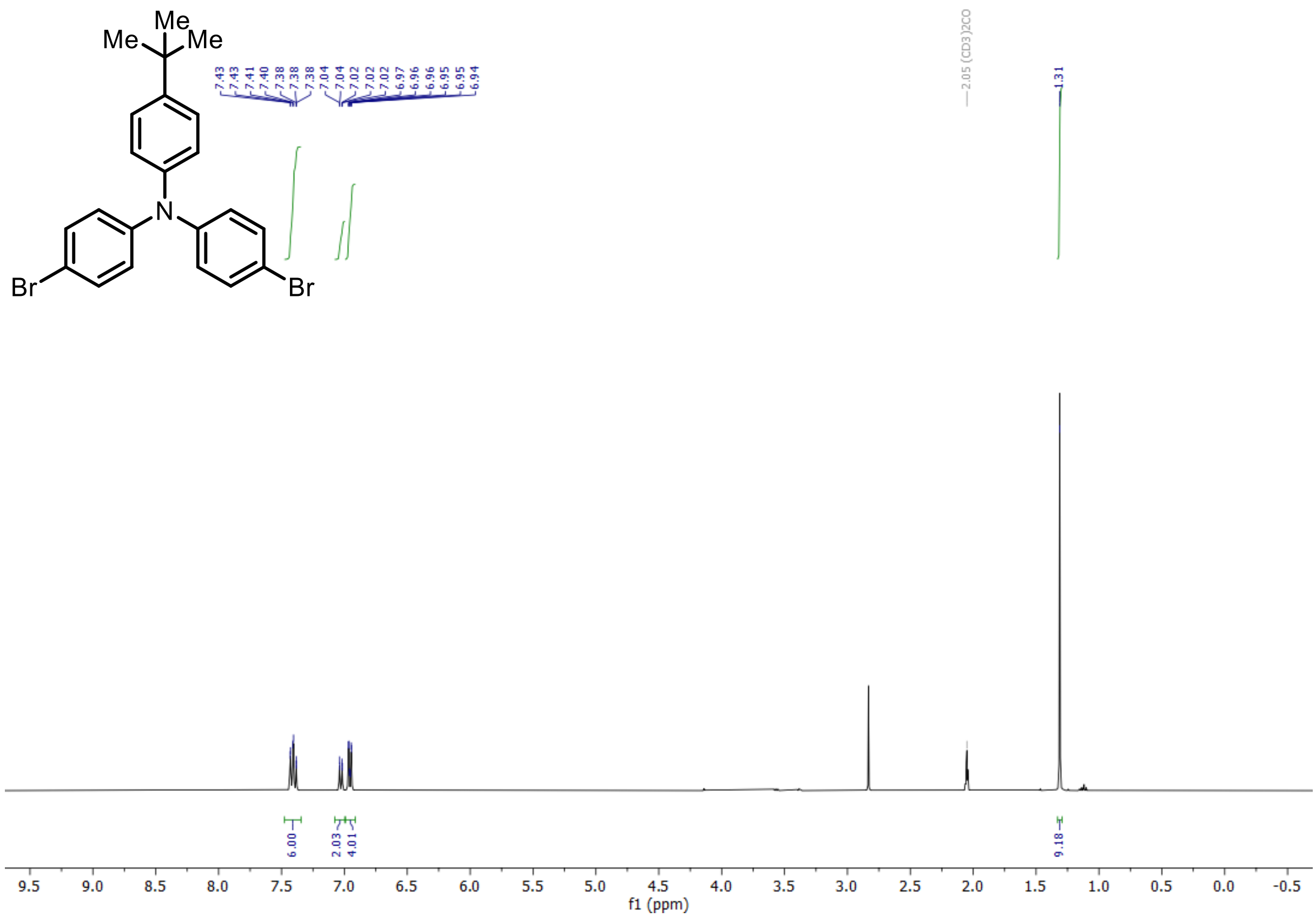

^{1}H NMR spectra of **3g**.

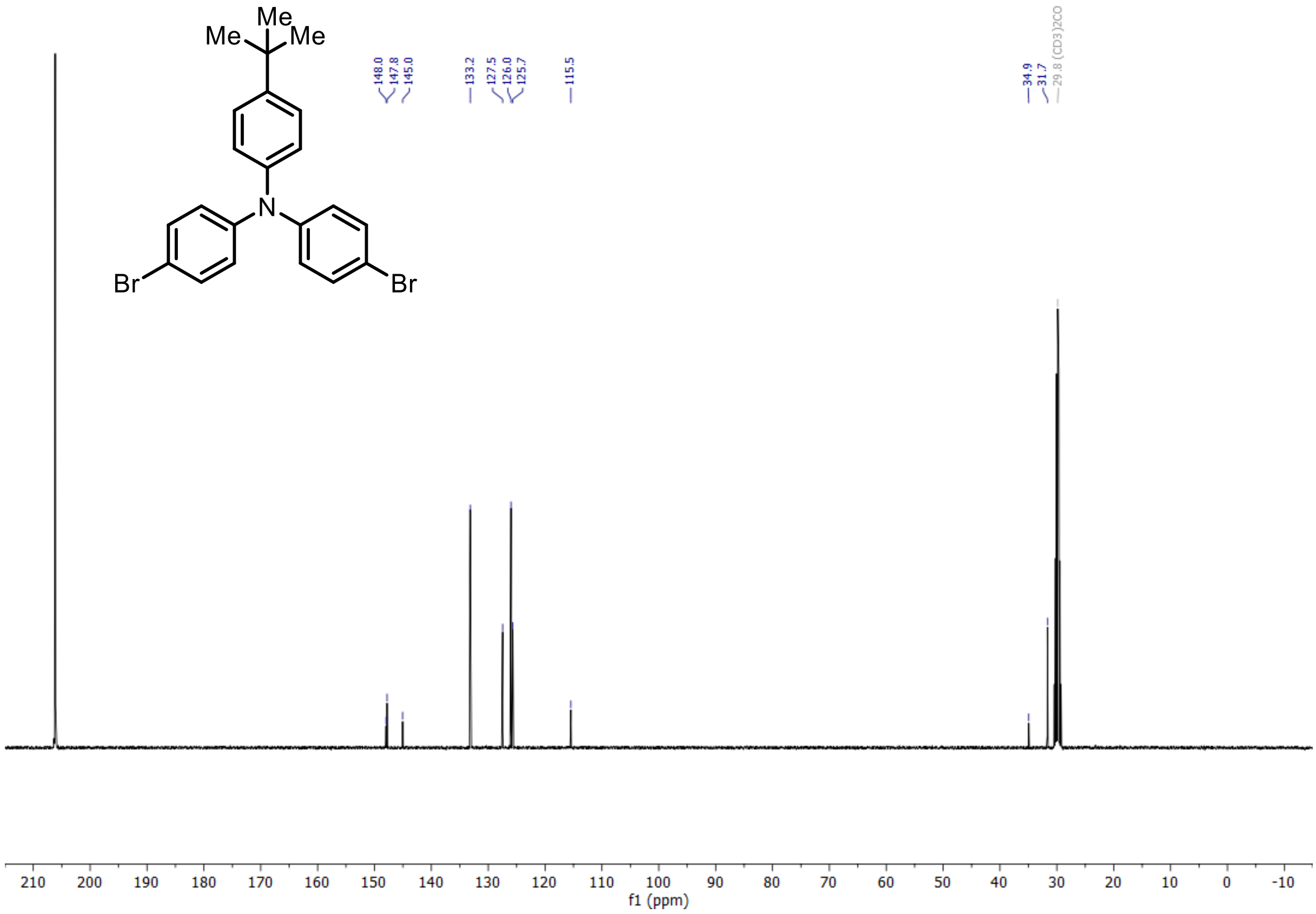

^{13}C NMR spectra of **3g**.

M.3. NMR spectra of products 4

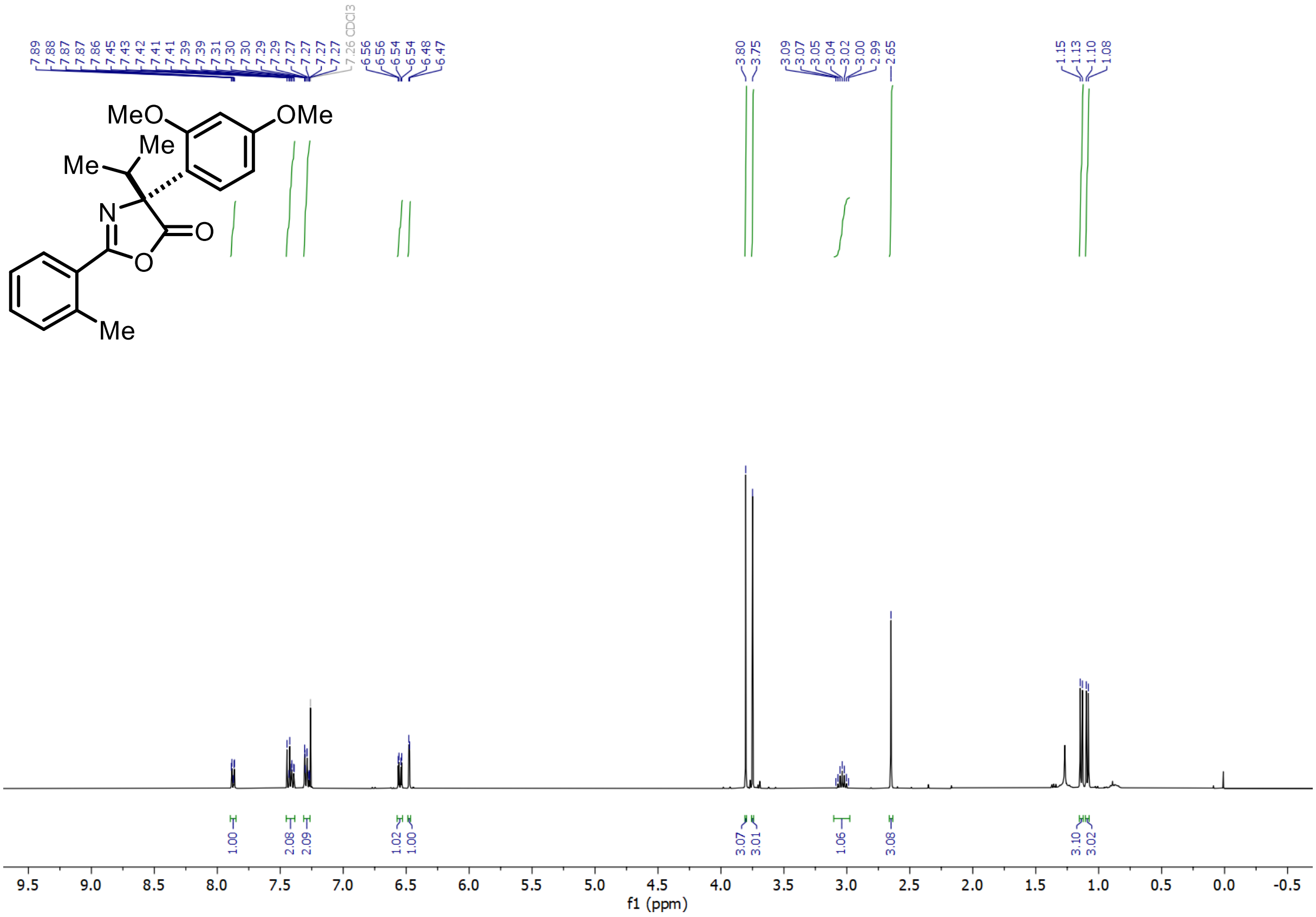

^{1}H NMR spectra of **4d**.

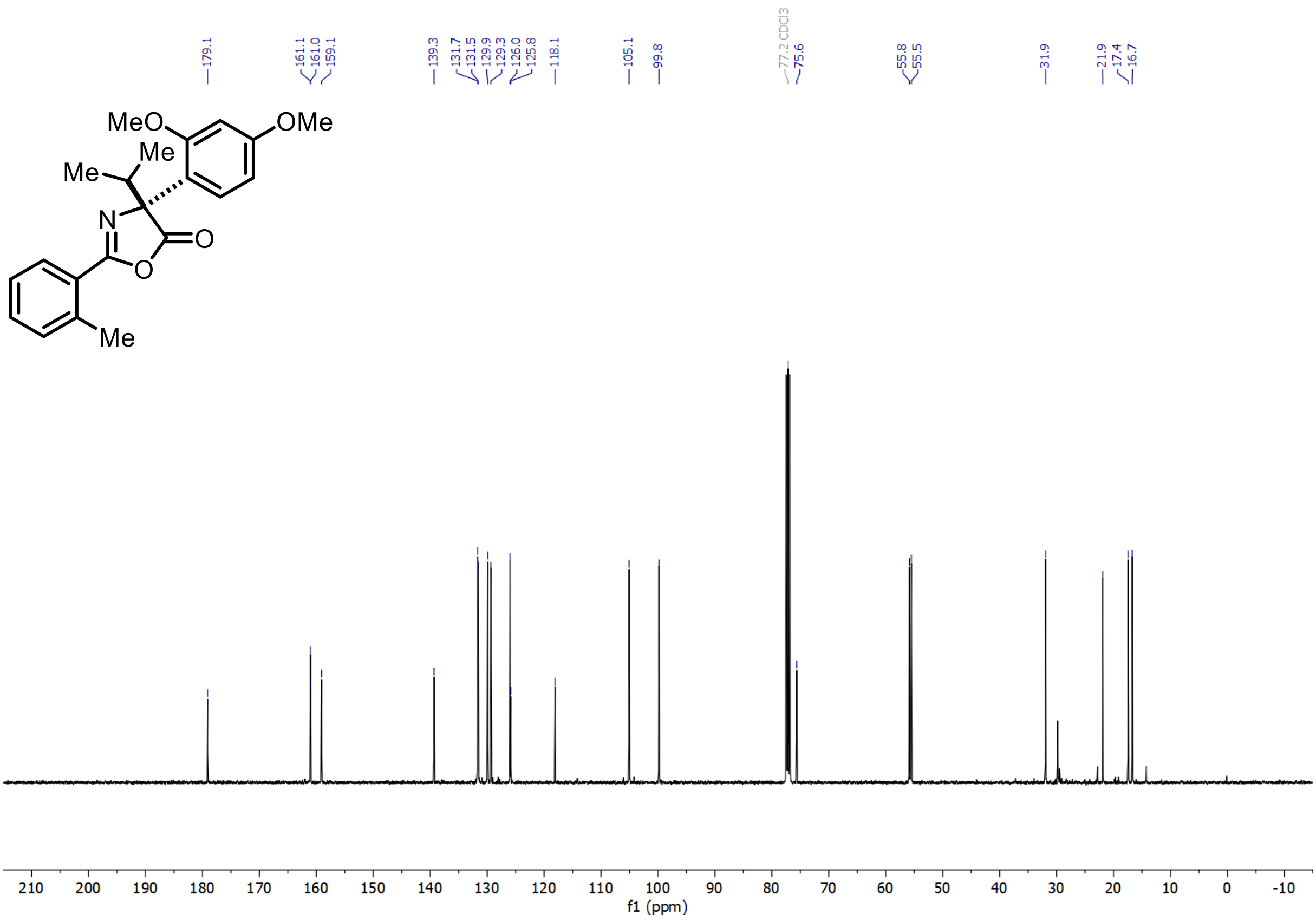

^{13}C NMR spectra of **4d**.

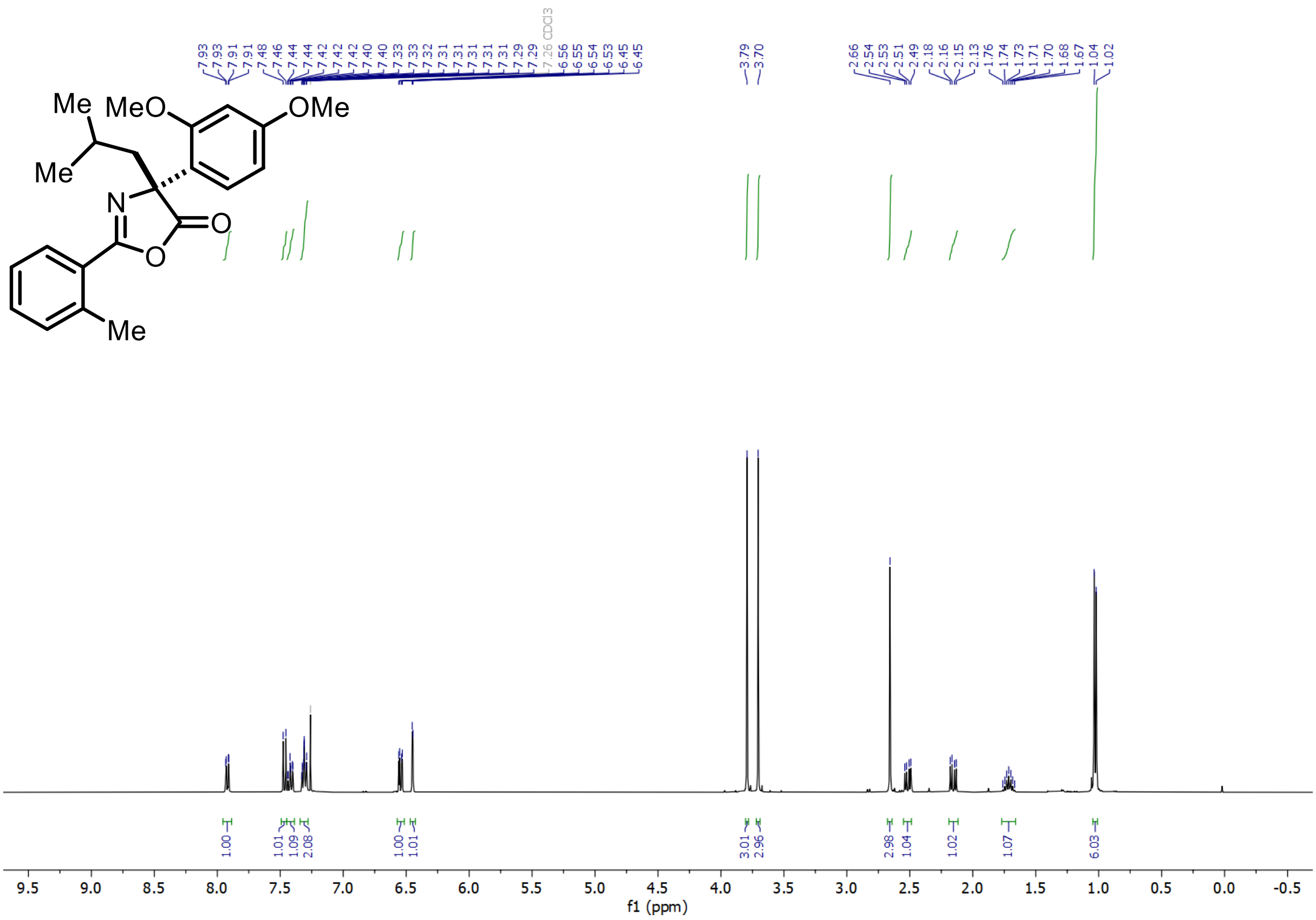

^{1}H NMR spectra of **4f**.

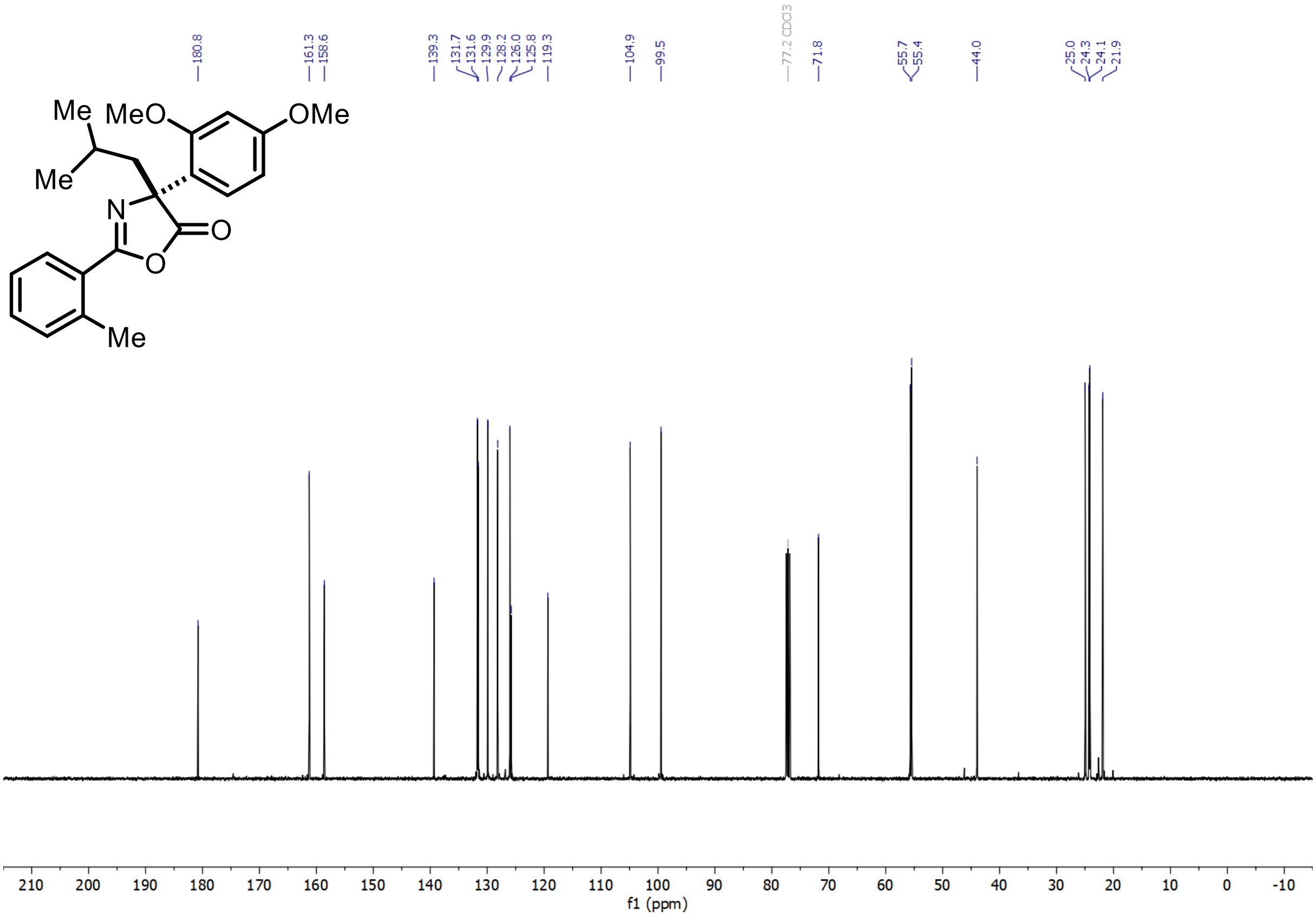

^{13}C NMR spectra of **4f**.

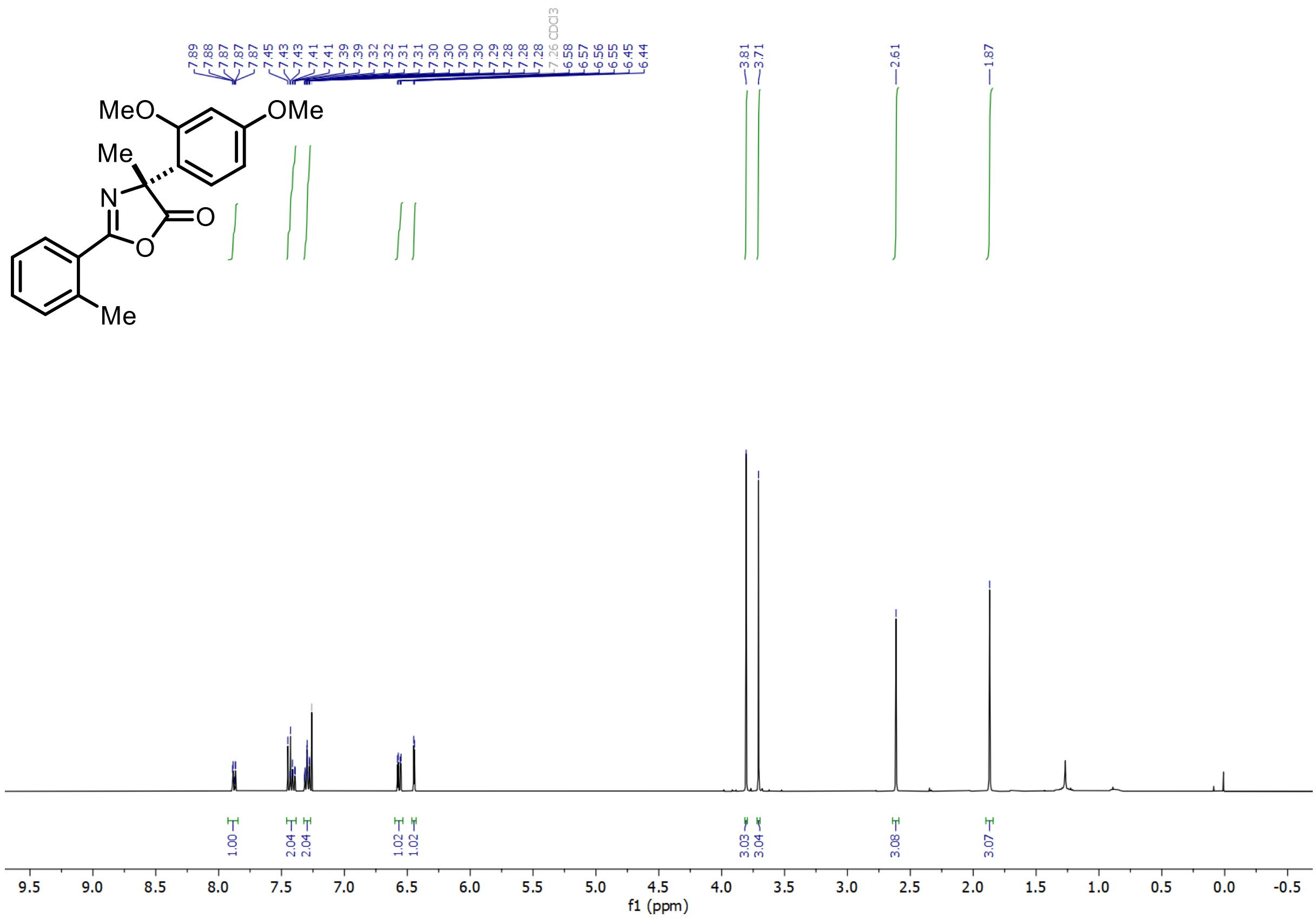

^{1}H NMR spectra of **4e**.

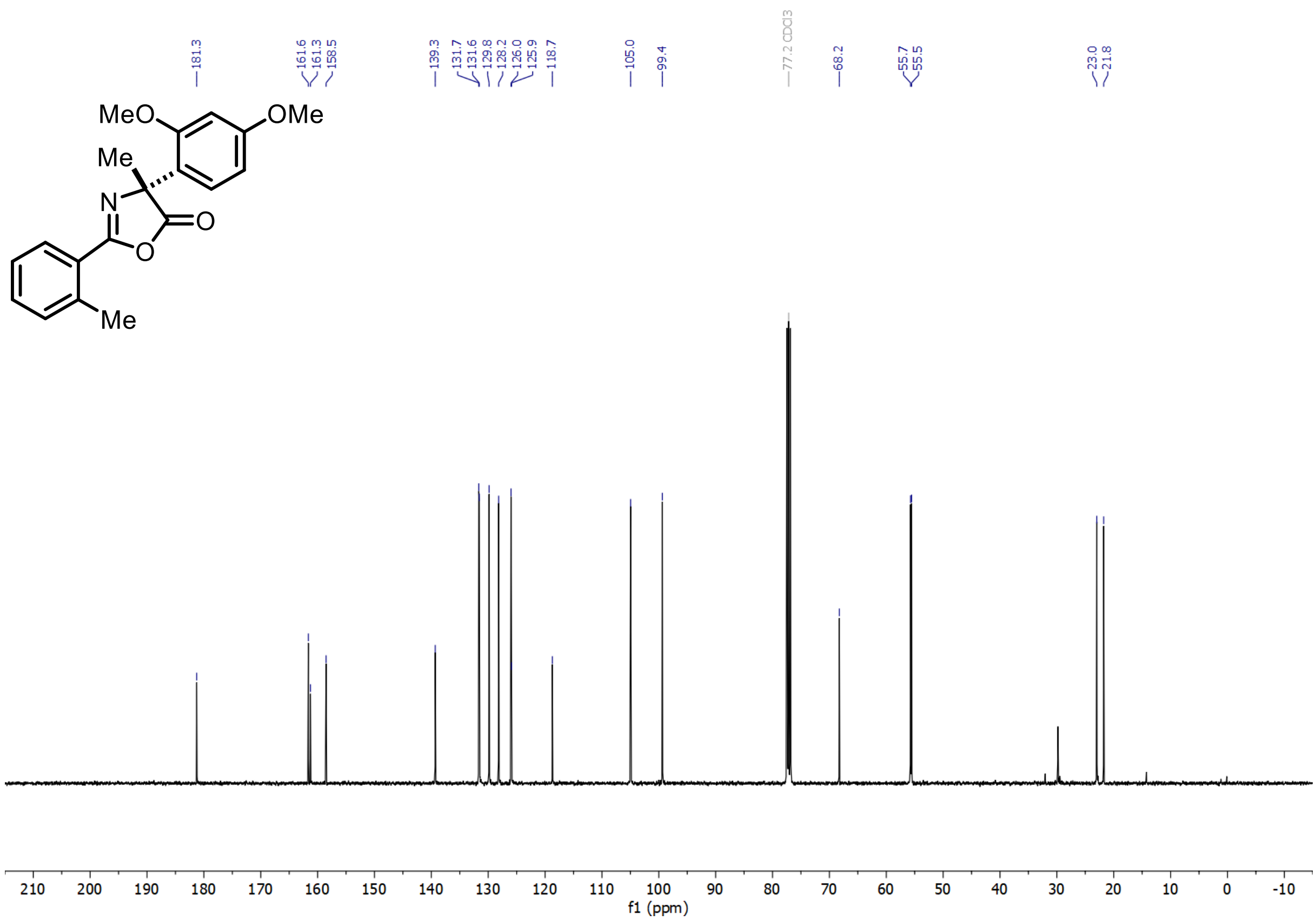

^{13}C NMR spectra of **4e**.

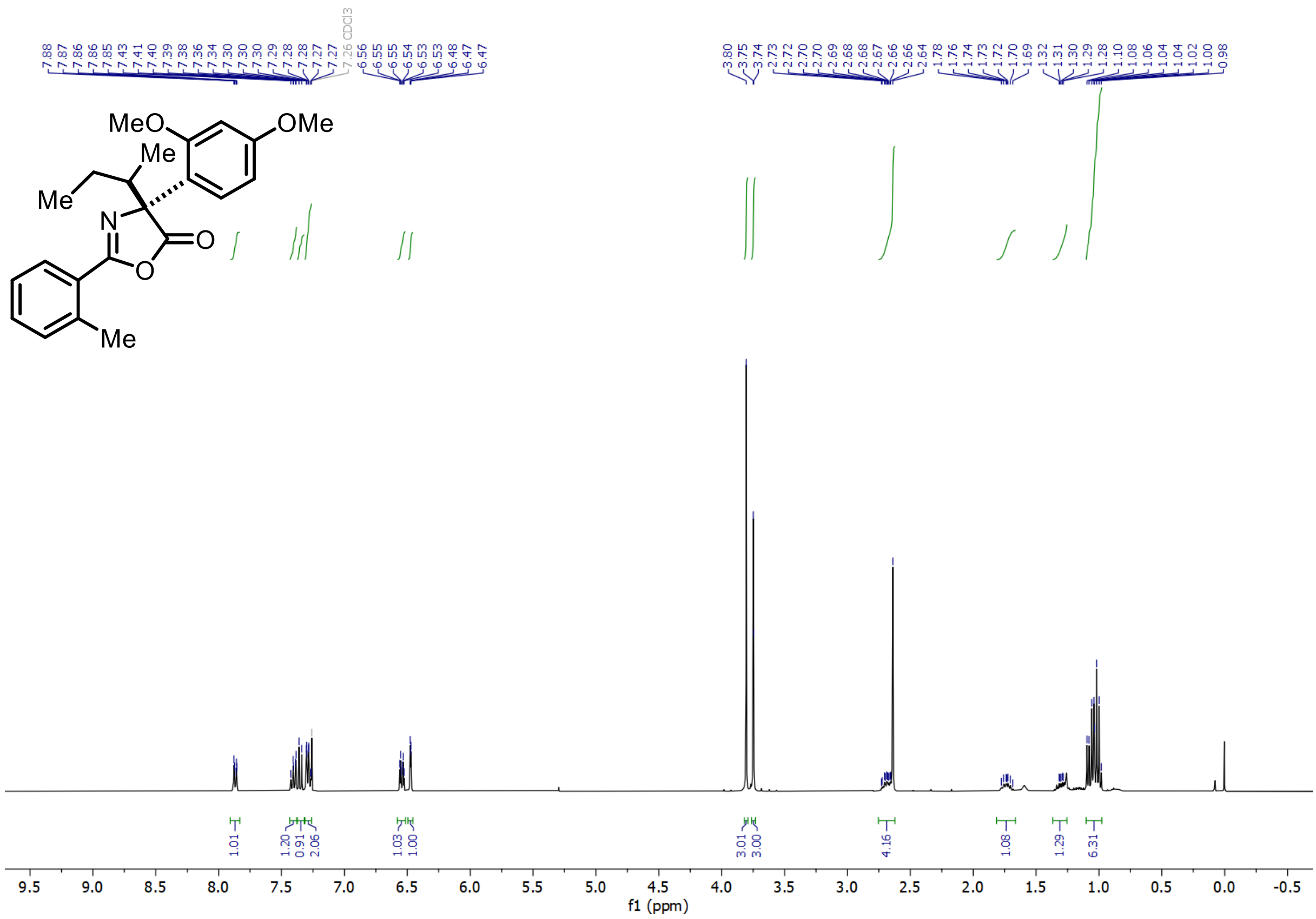

^{1}H NMR spectra of **4g**.

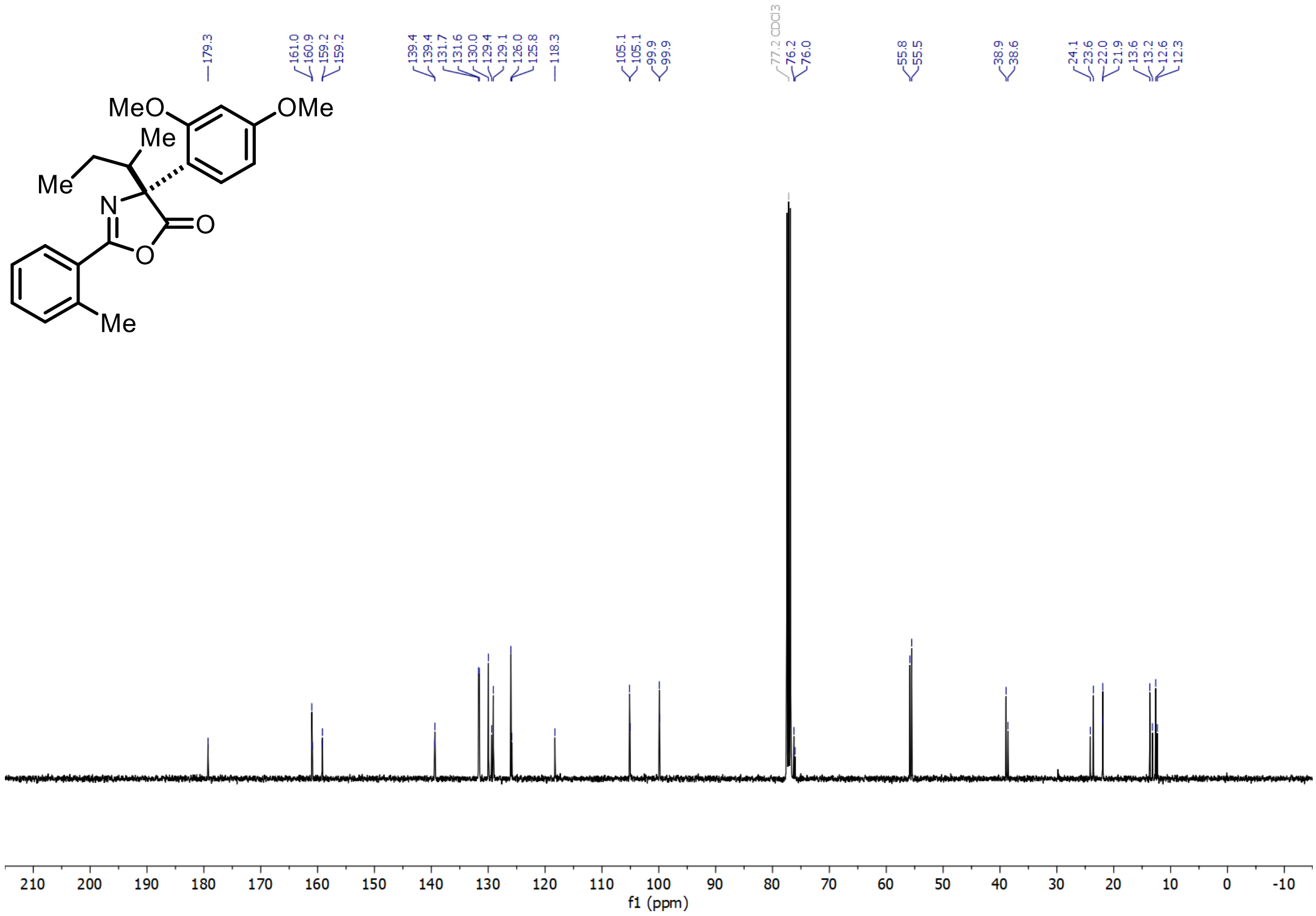

^{13}C NMR spectra of **4g**.

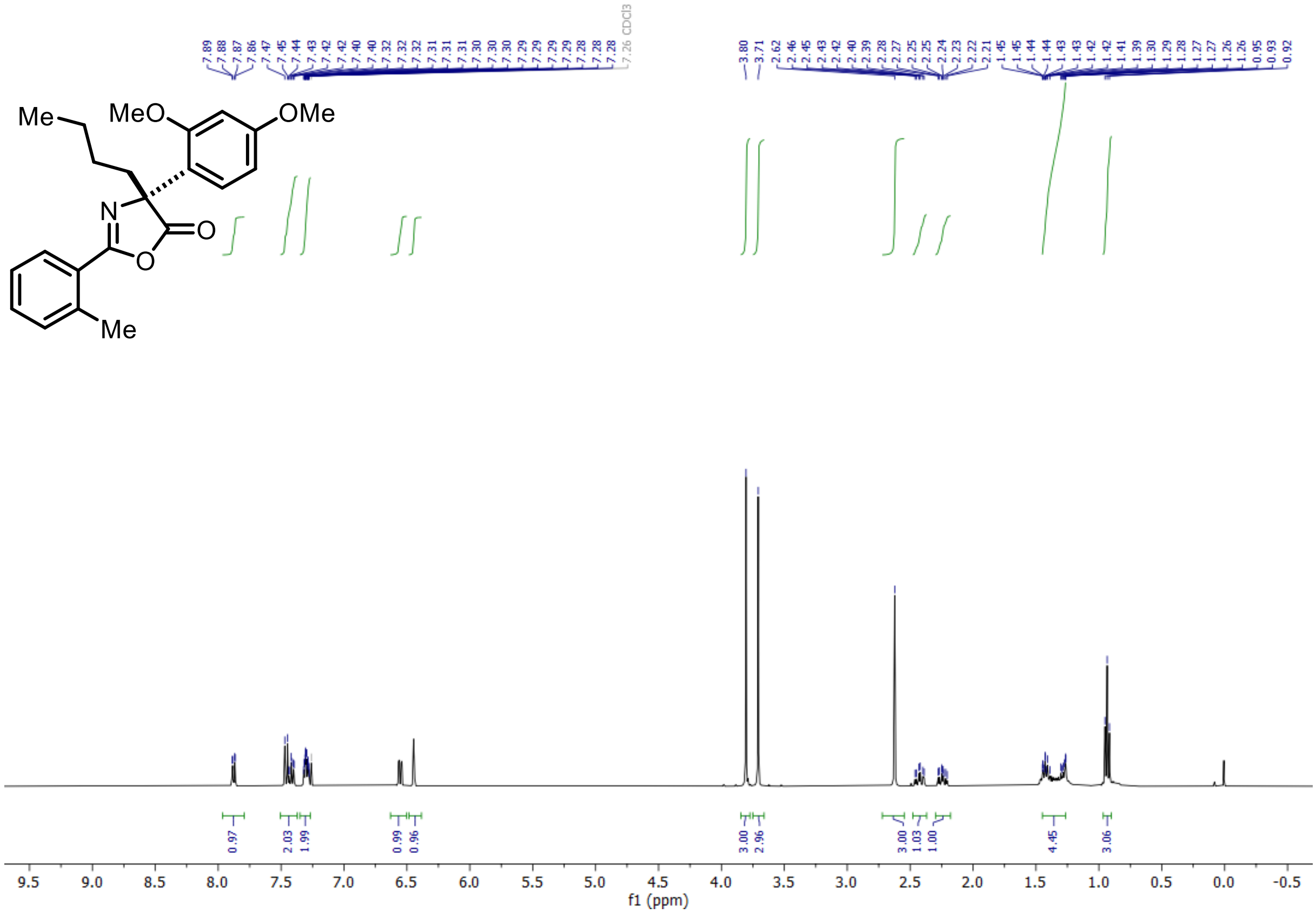

^{1}H NMR spectra of **4h**.

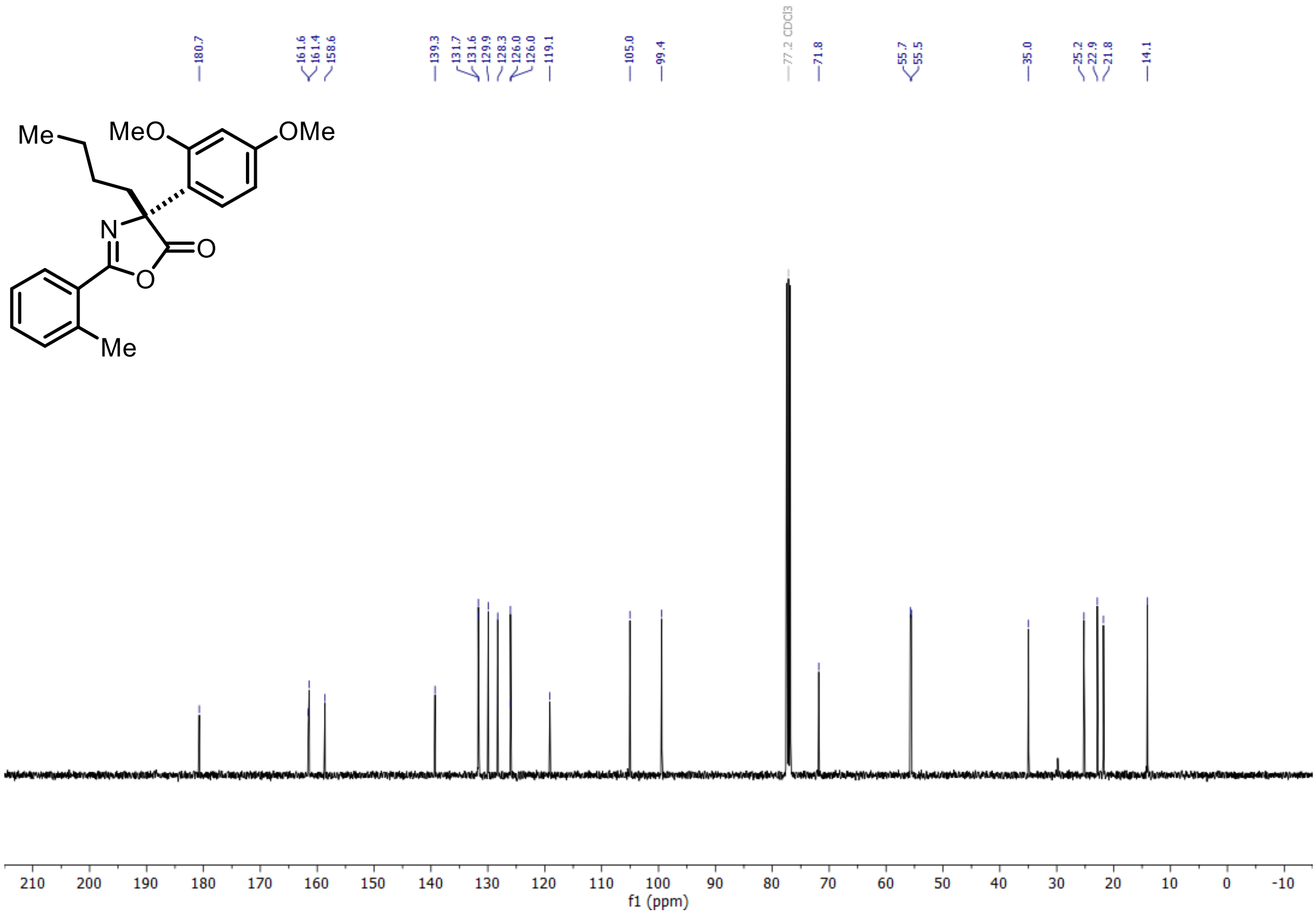

^{13}C NMR spectra of **4h**.

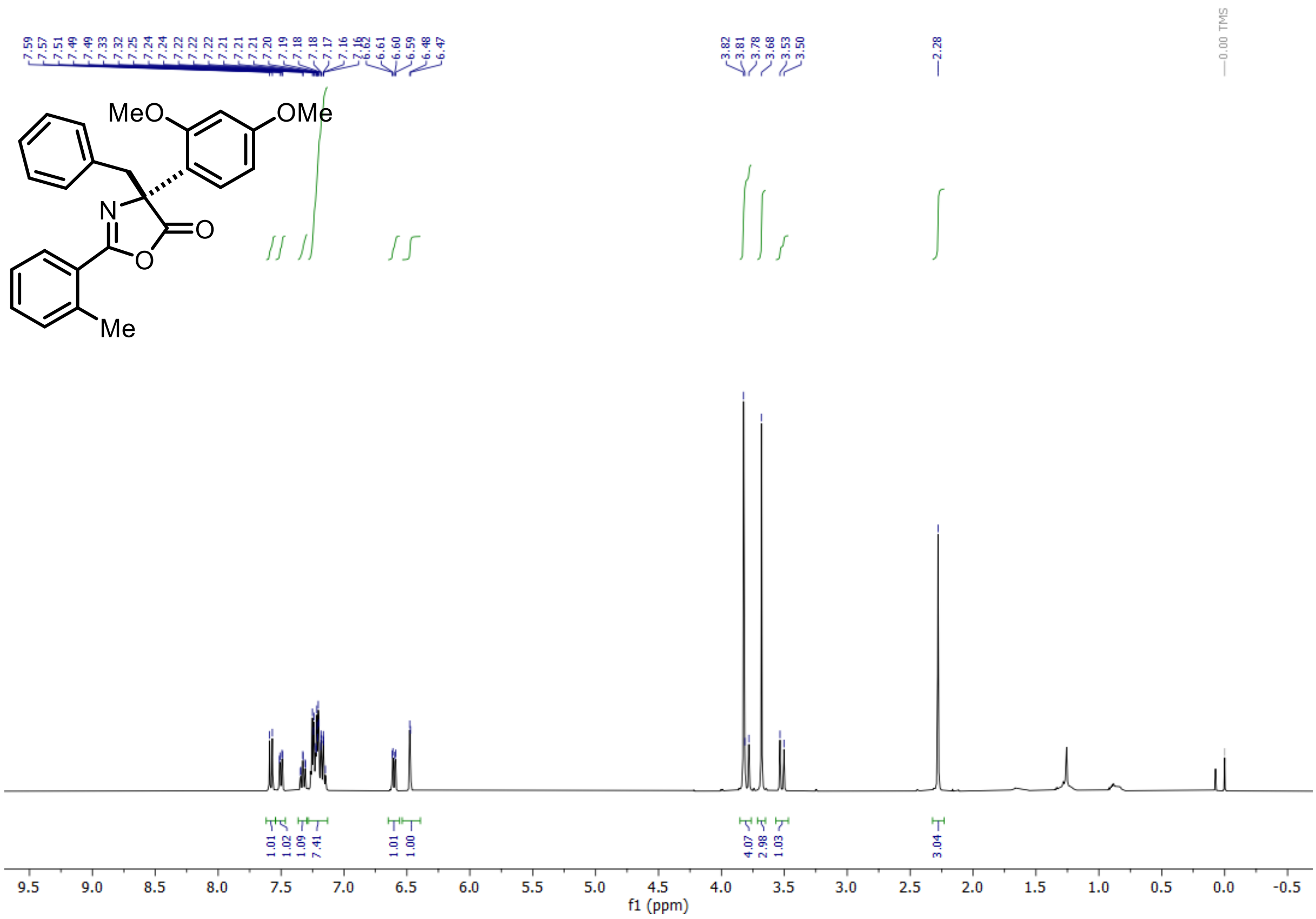

^{1}H NMR spectra of **4i**.

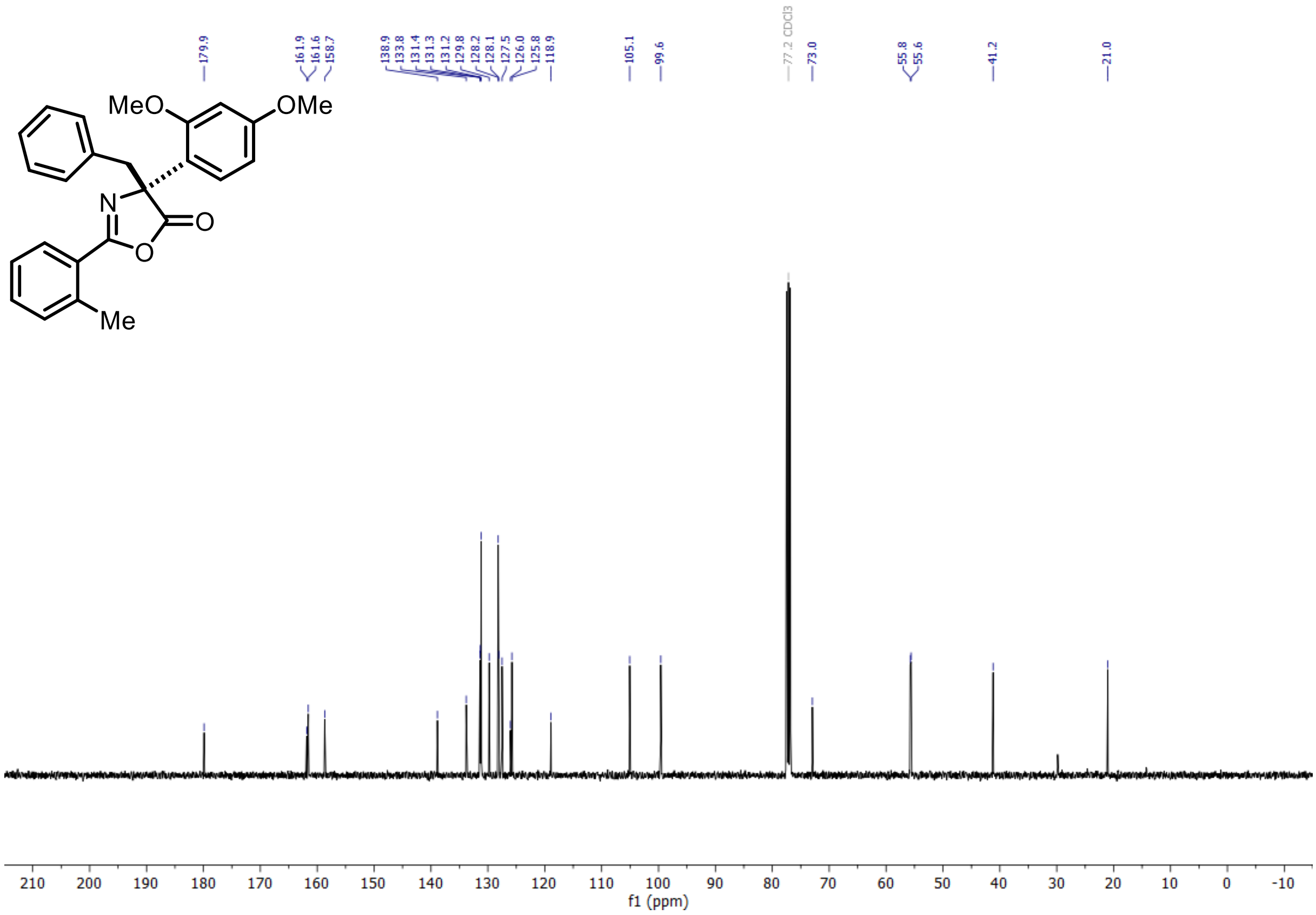

^{13}C NMR spectra of **4i**.

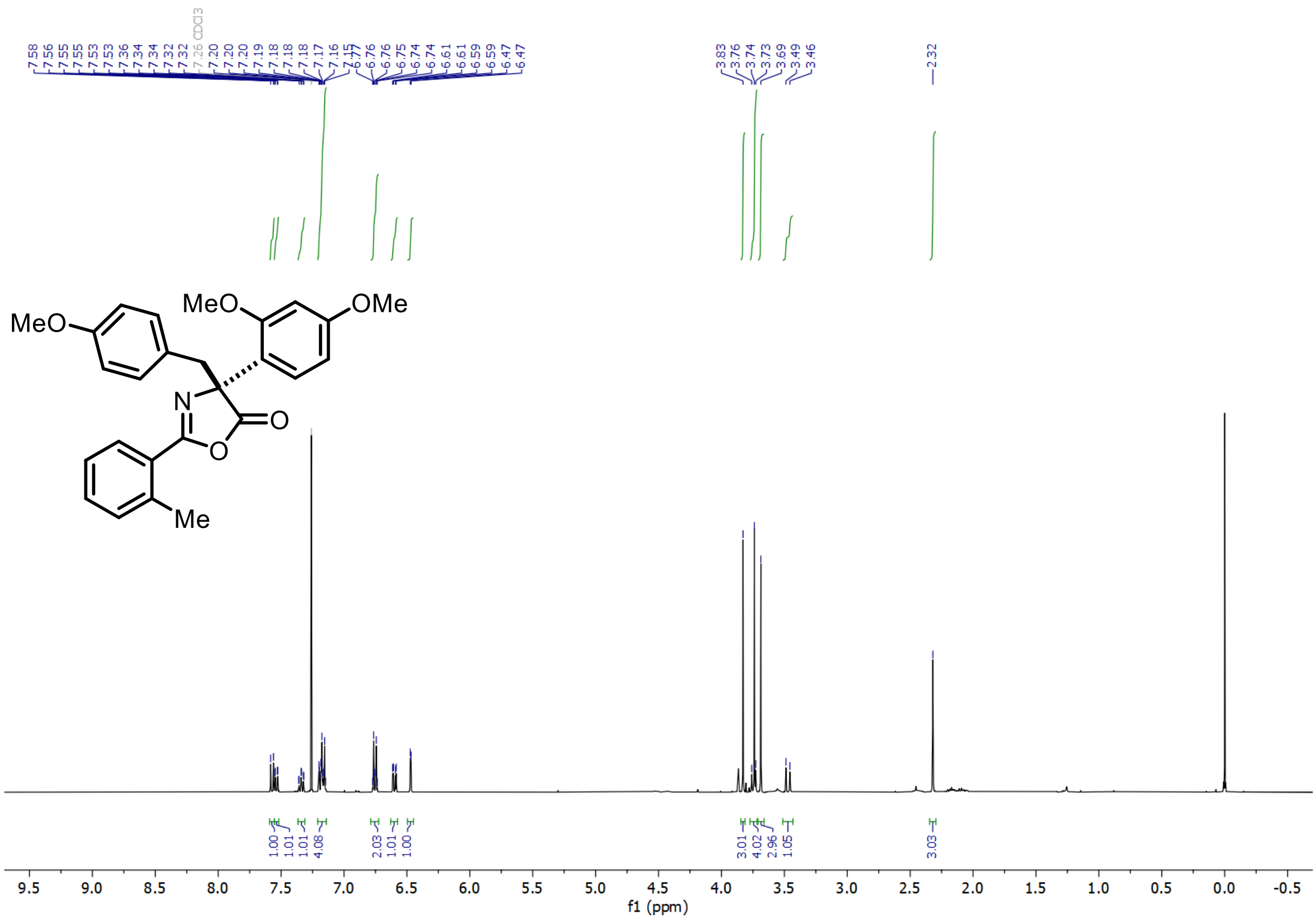

^{1}H NMR spectra of **4j**.

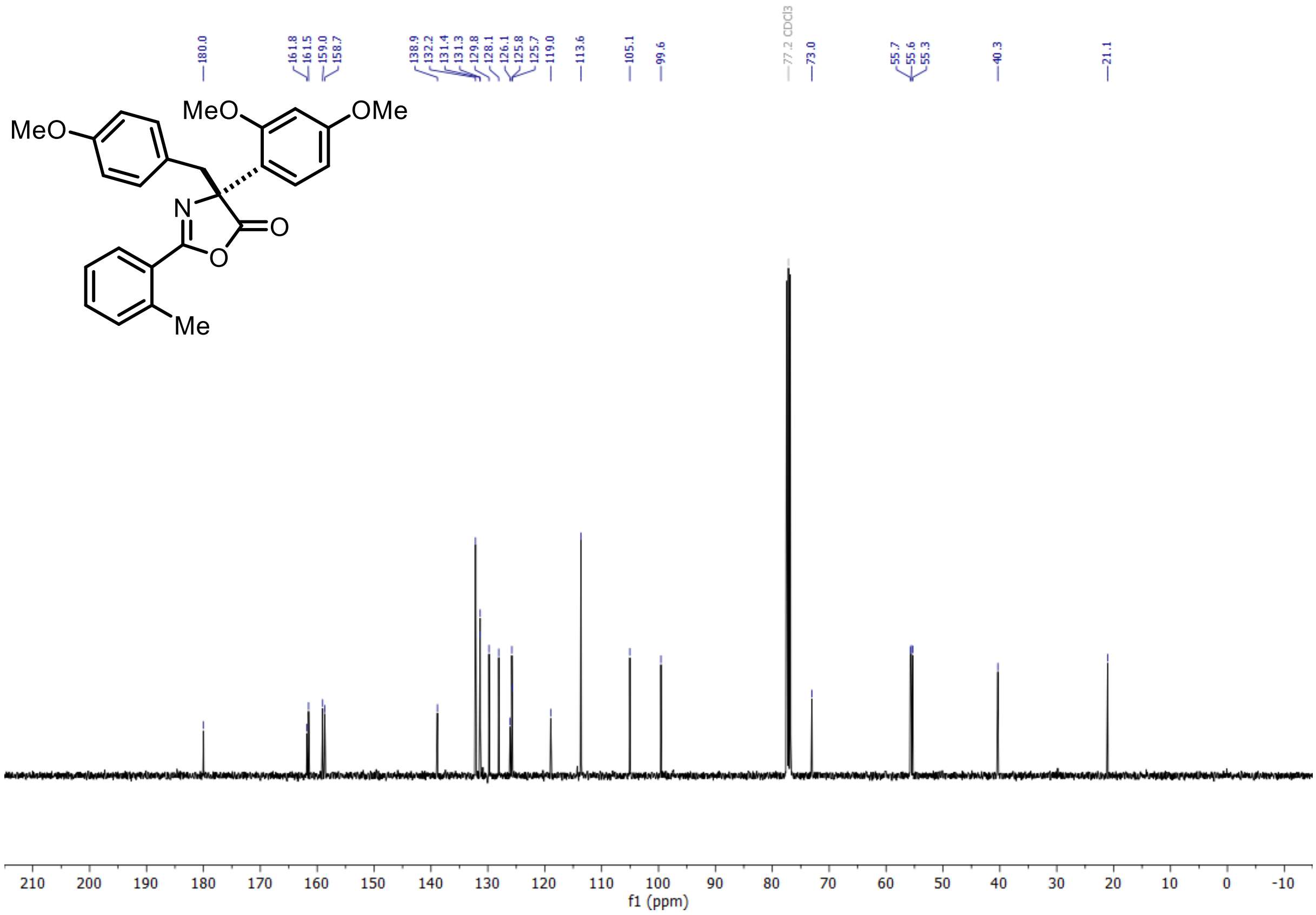

^{13}C NMR spectra of **4j**.

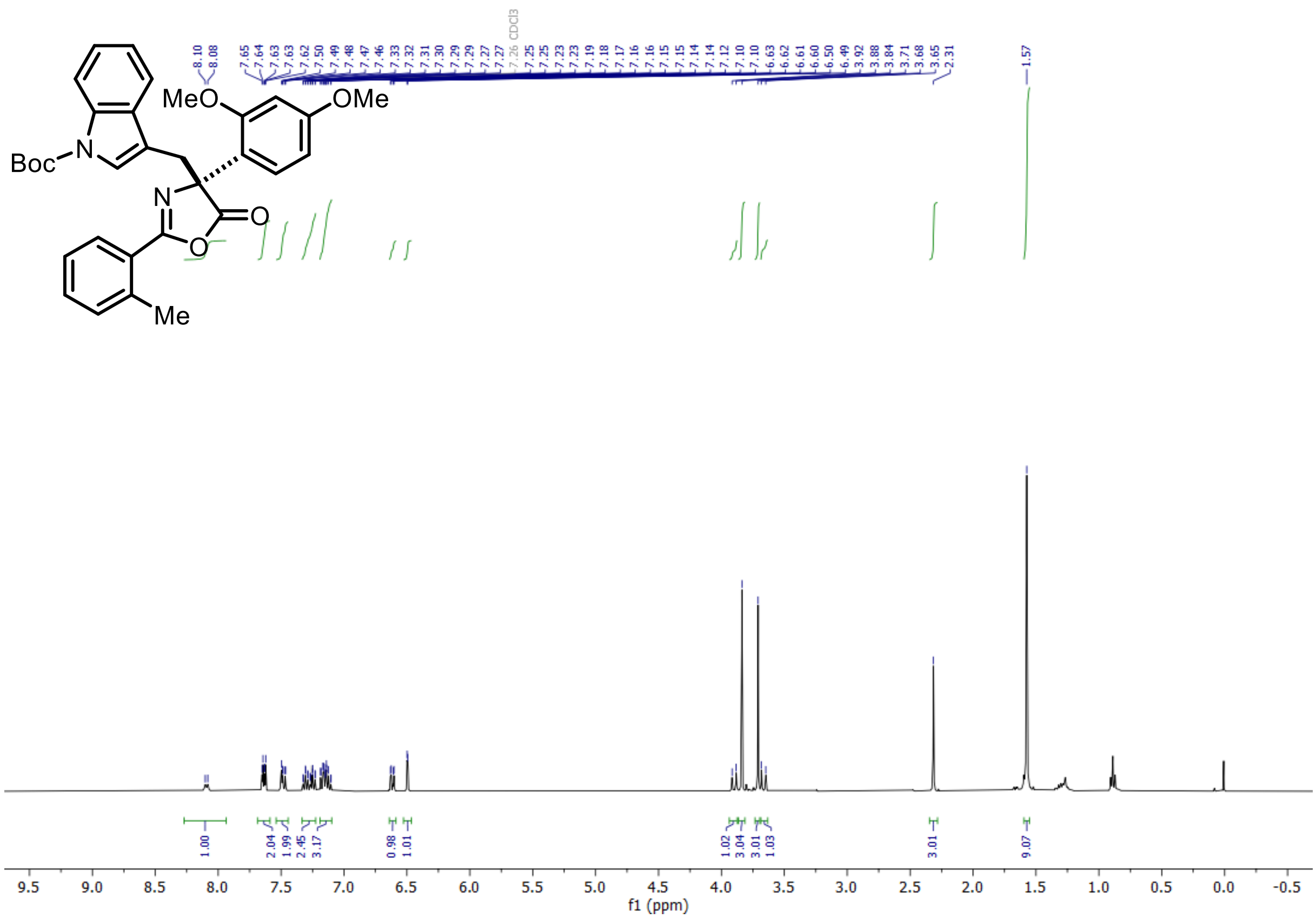

^{1}H NMR spectra of **4k**.

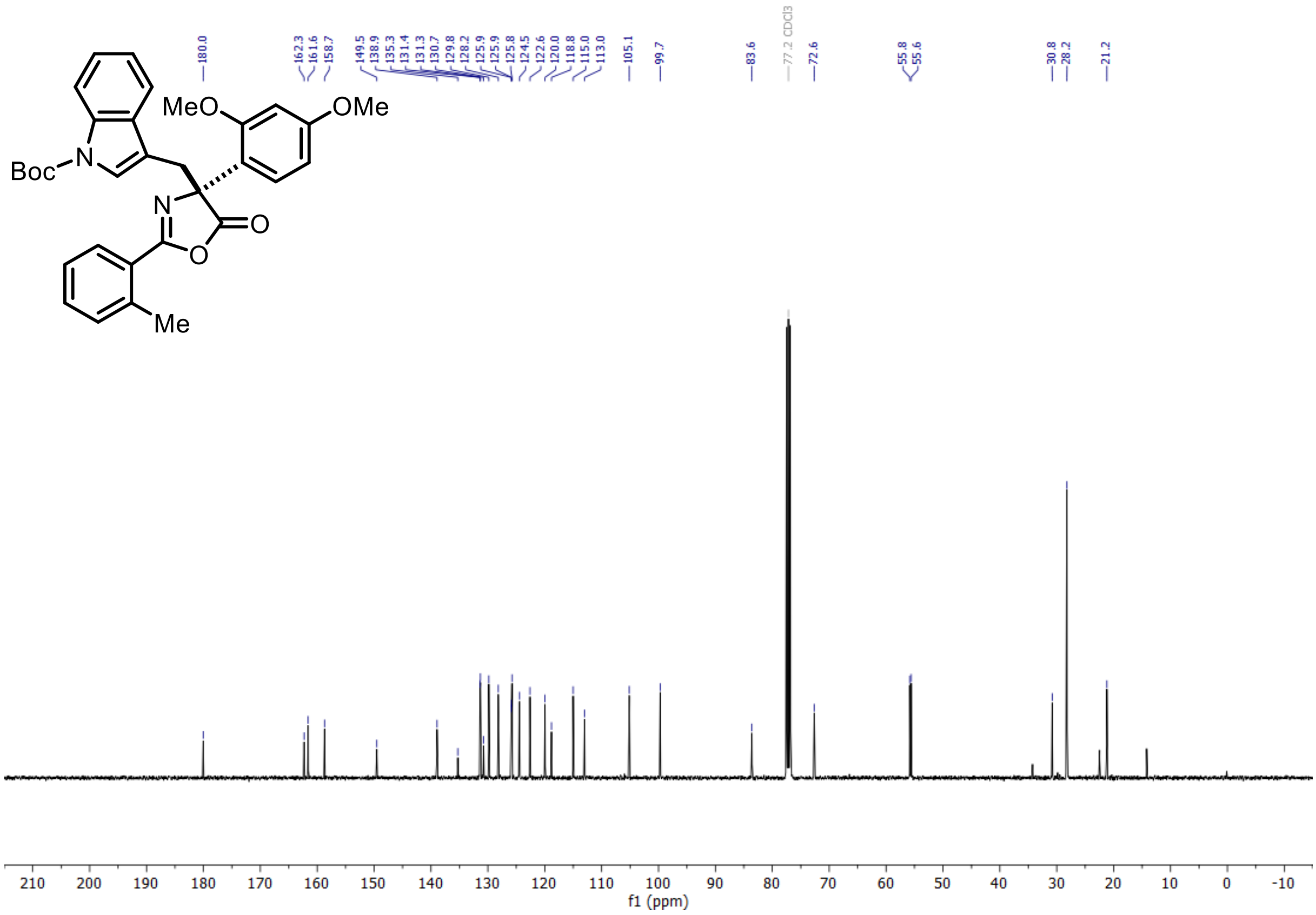

^{13}C NMR spectra of **4k**.

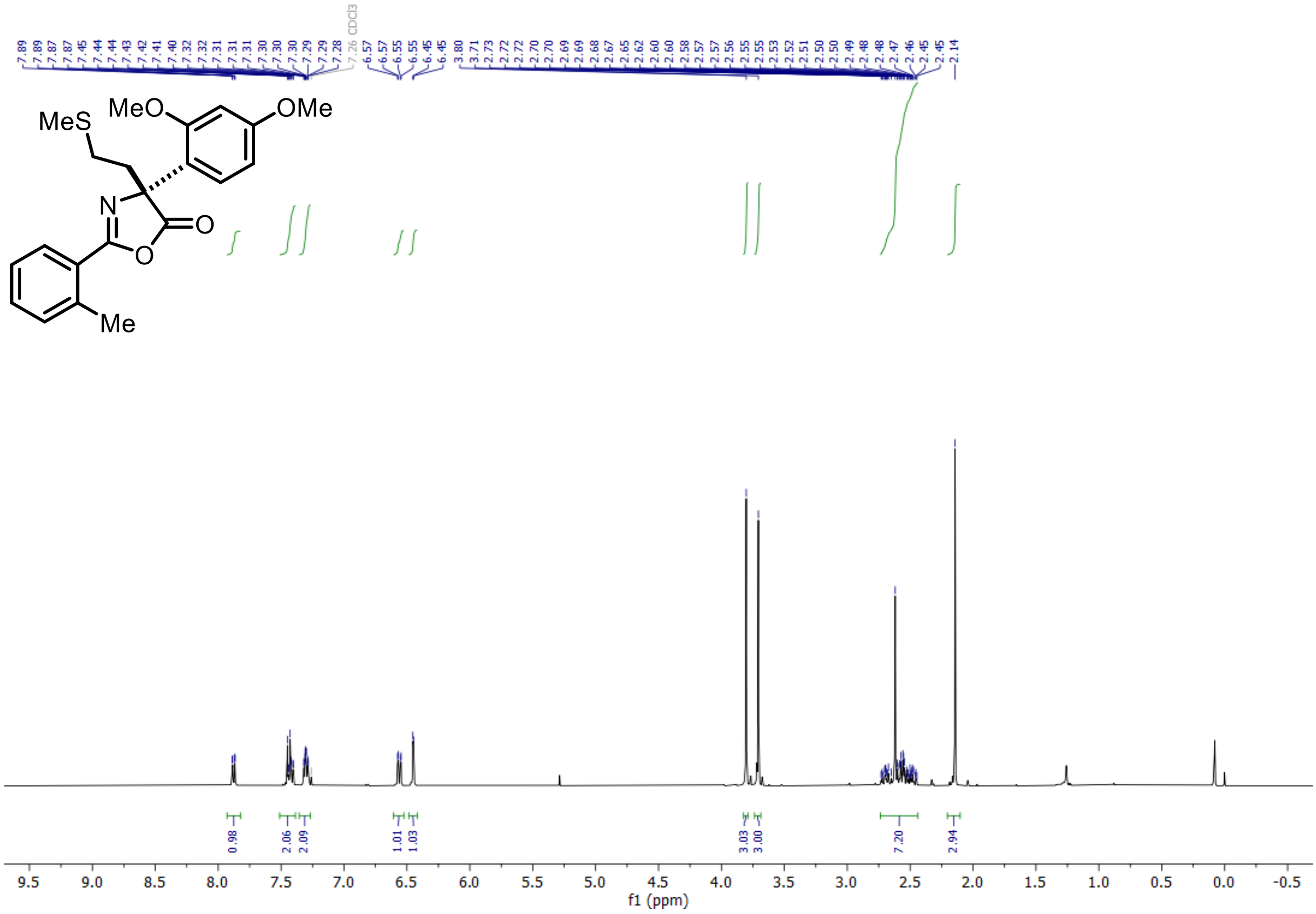

^{1}H NMR spectra of **4l**.

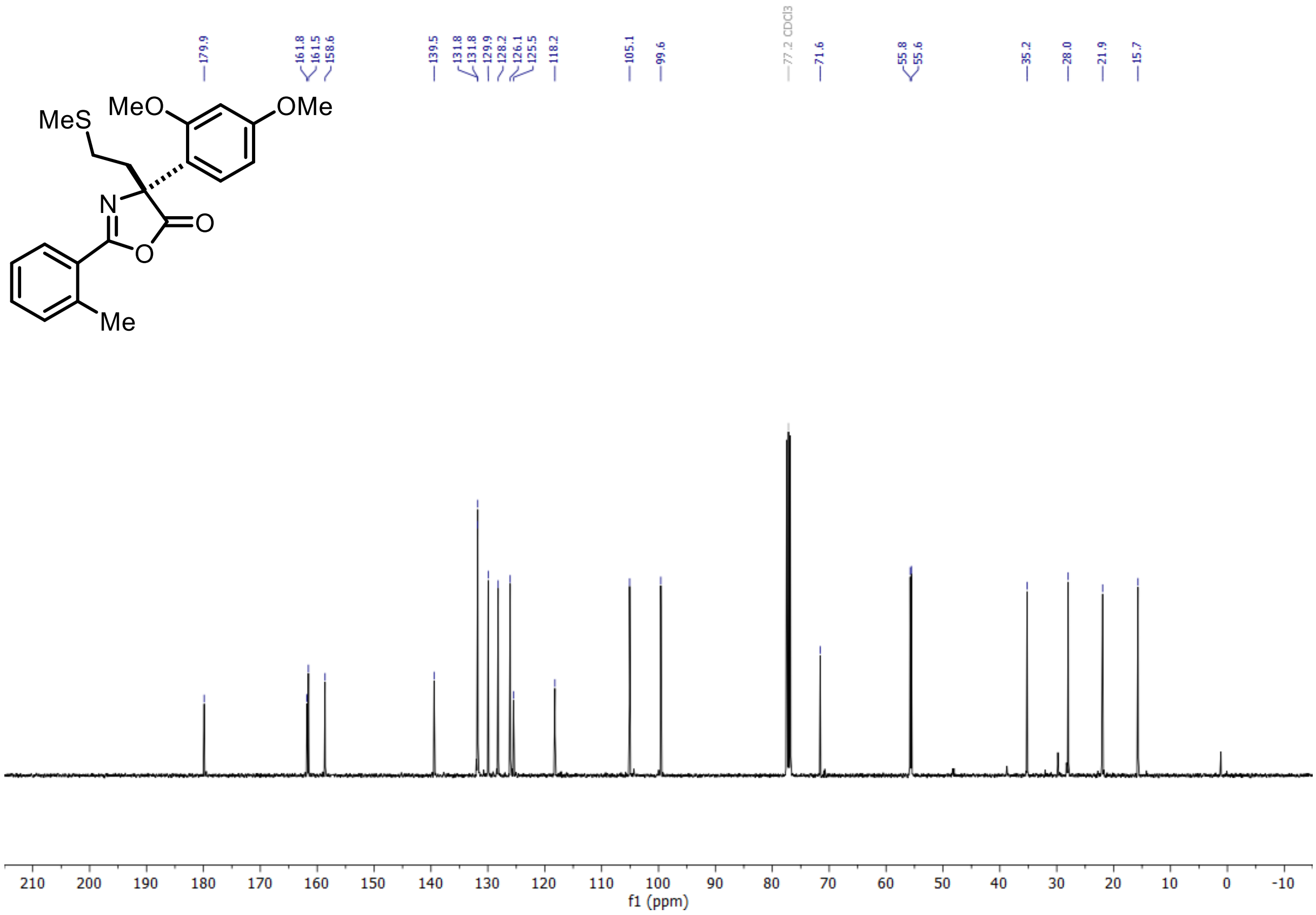

^{13}C NMR spectra of **4l**.

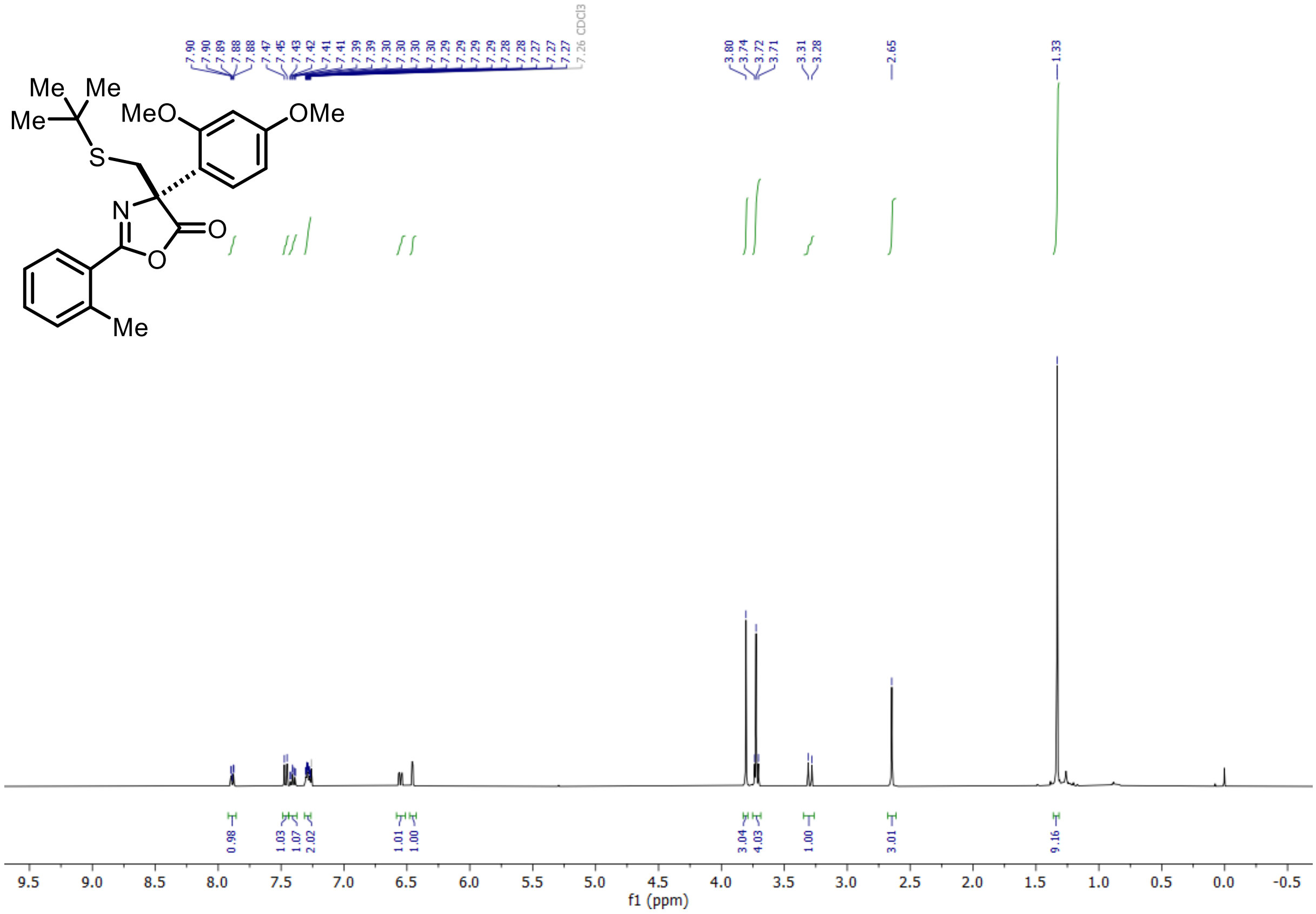

^{1}H NMR spectra of **4m**.

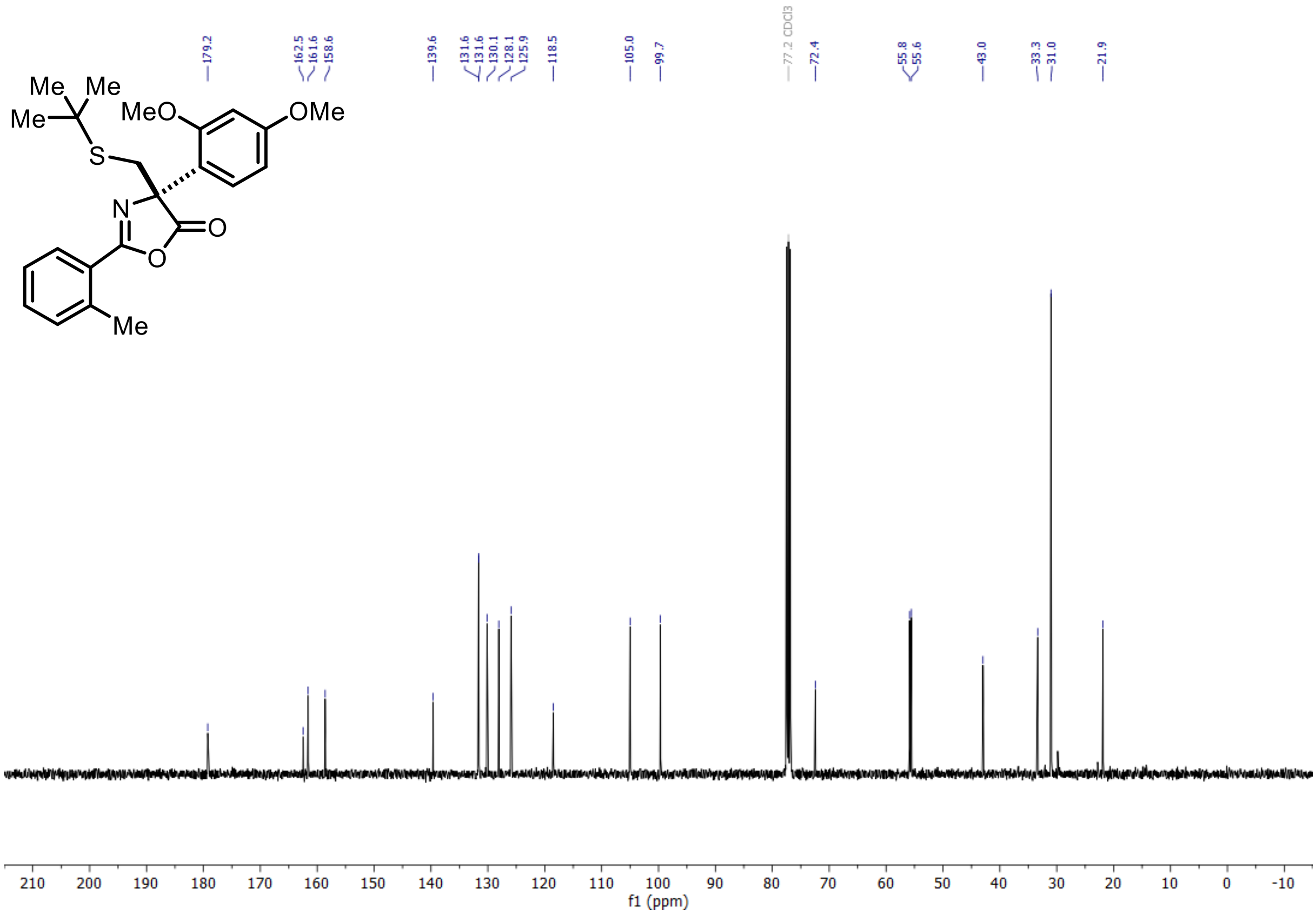

^{13}C NMR spectra of **4m**.

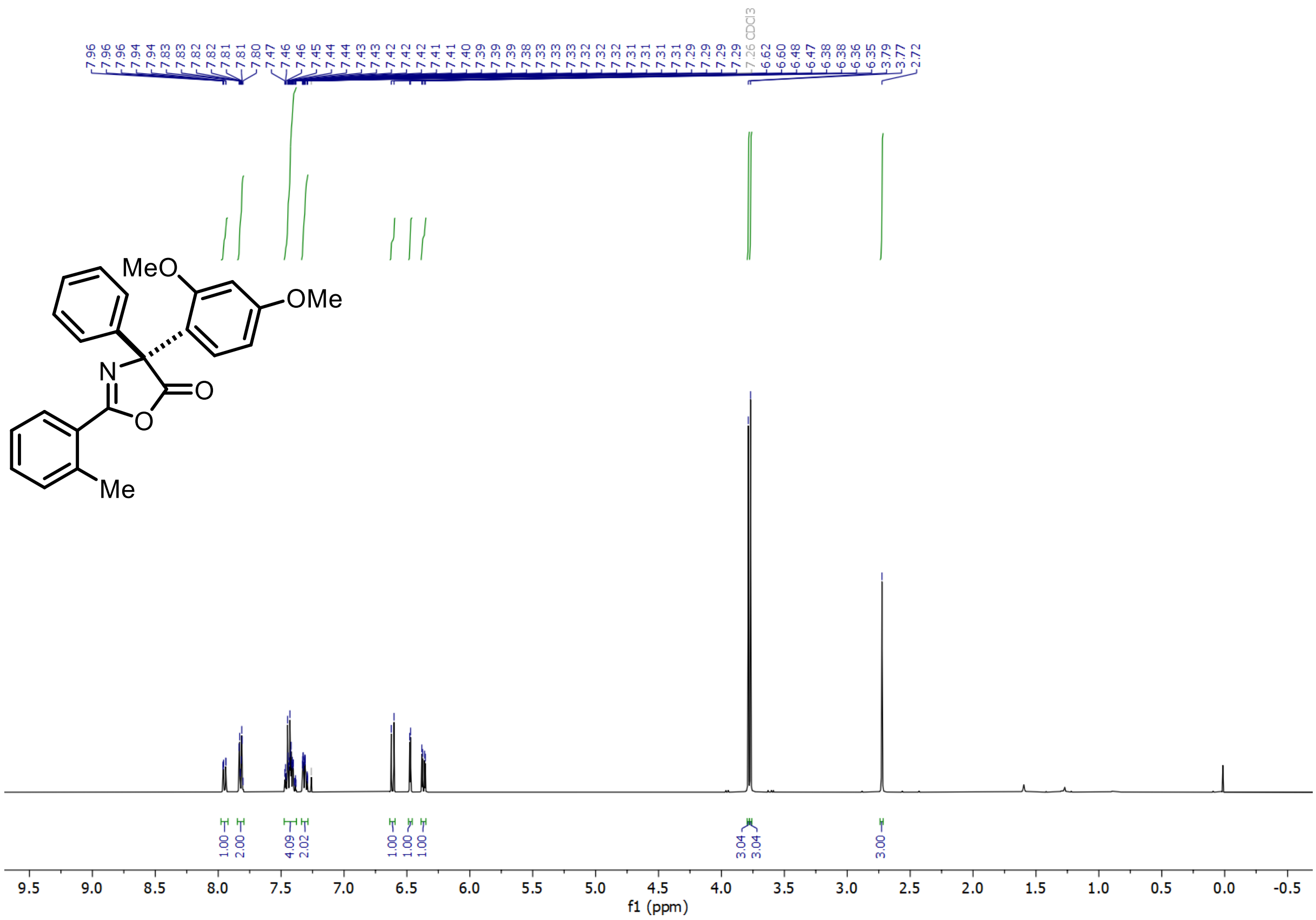

^{1}H NMR spectra of **4n**.

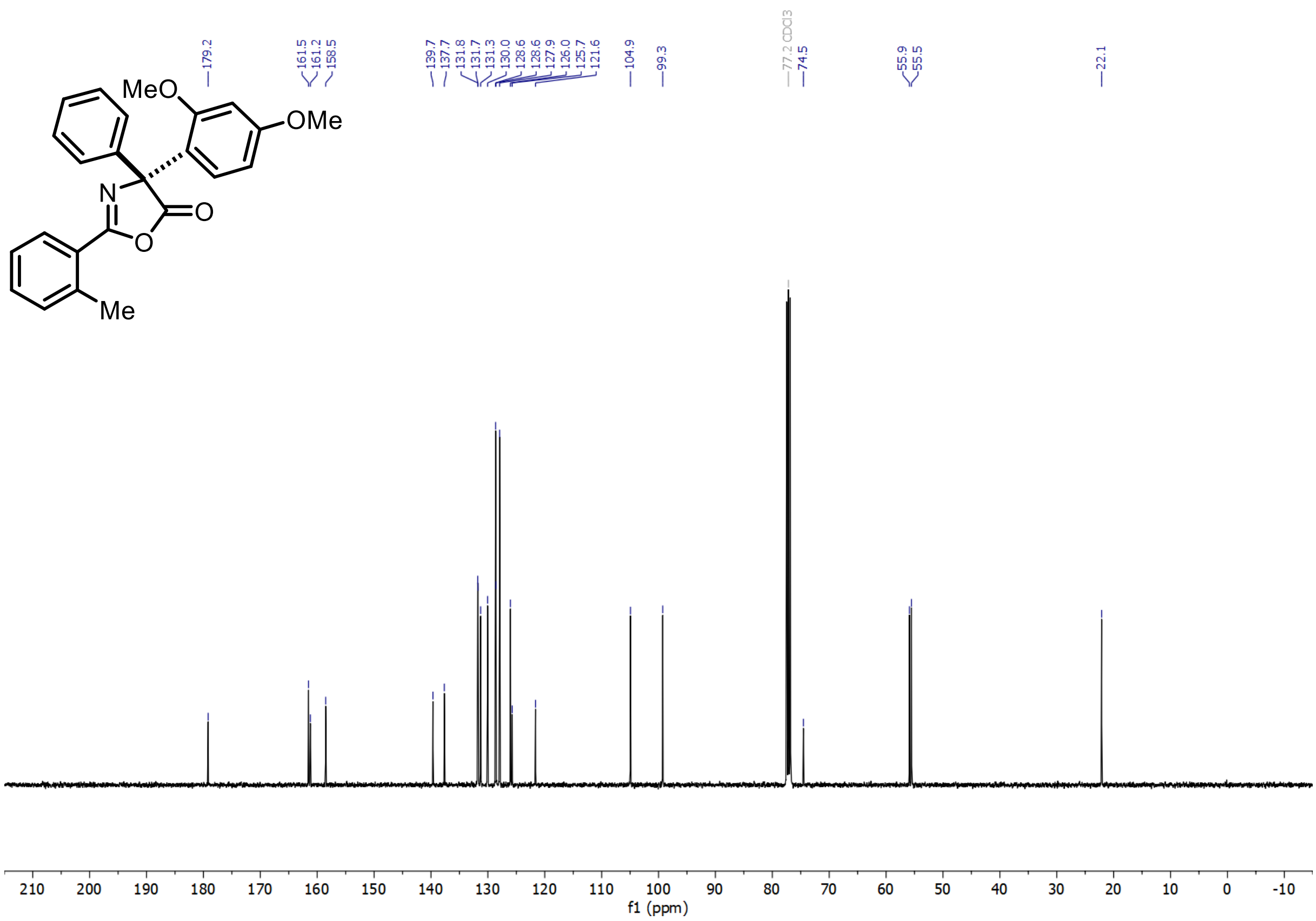

^{13}C NMR spectra of **4n**.

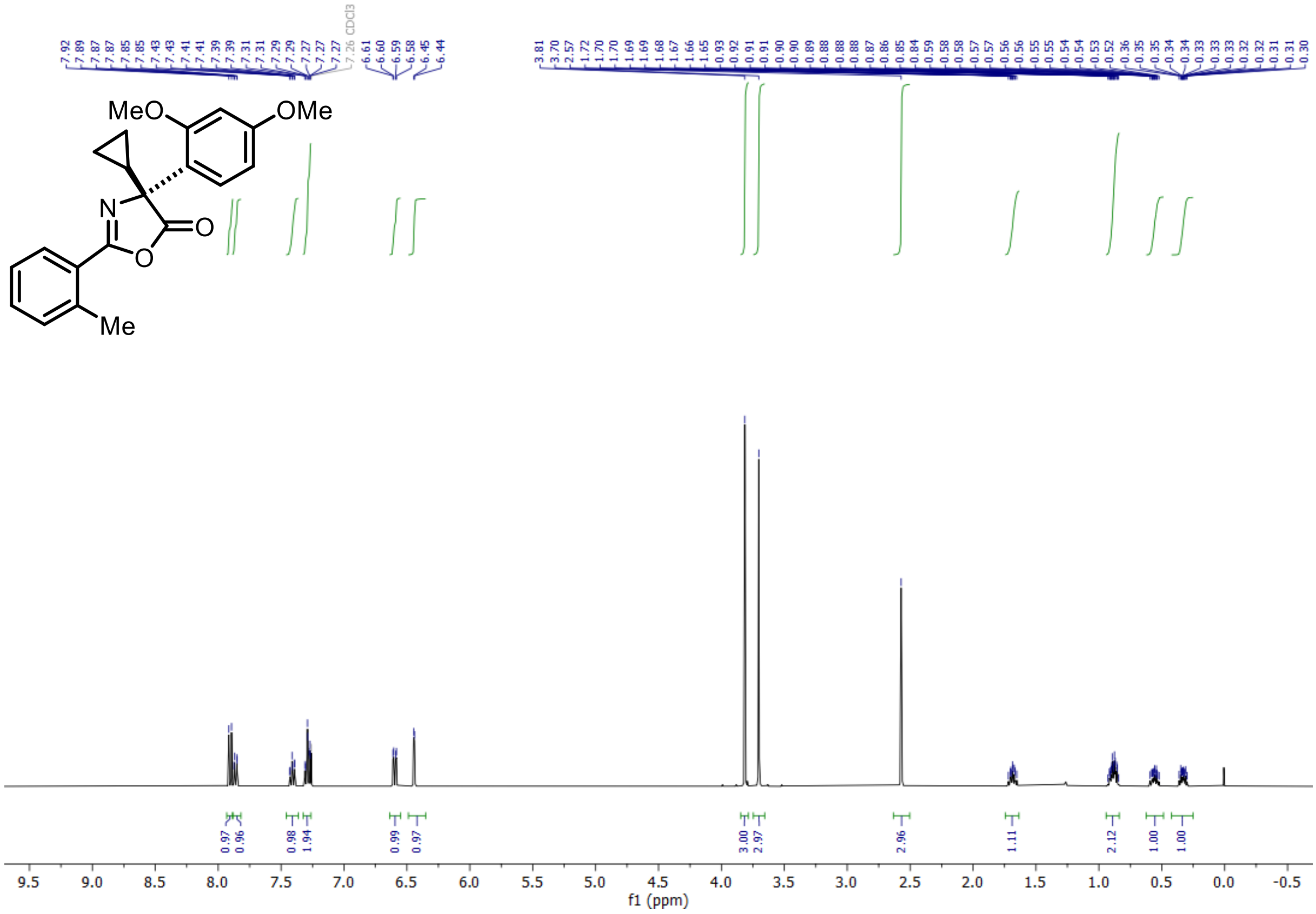

^{1}H NMR spectra of **4o**.

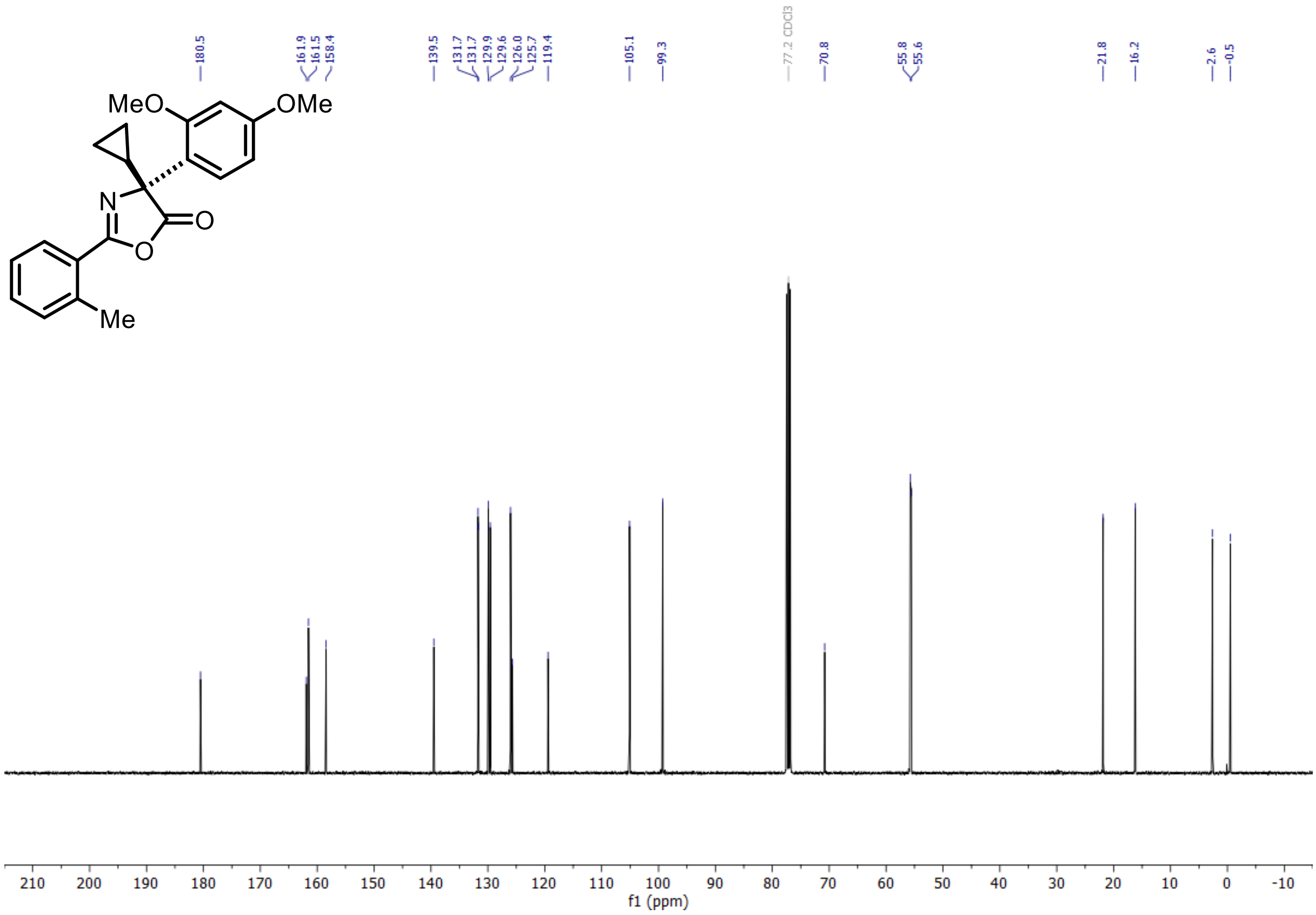

^{13}C NMR spectra of **4o**.

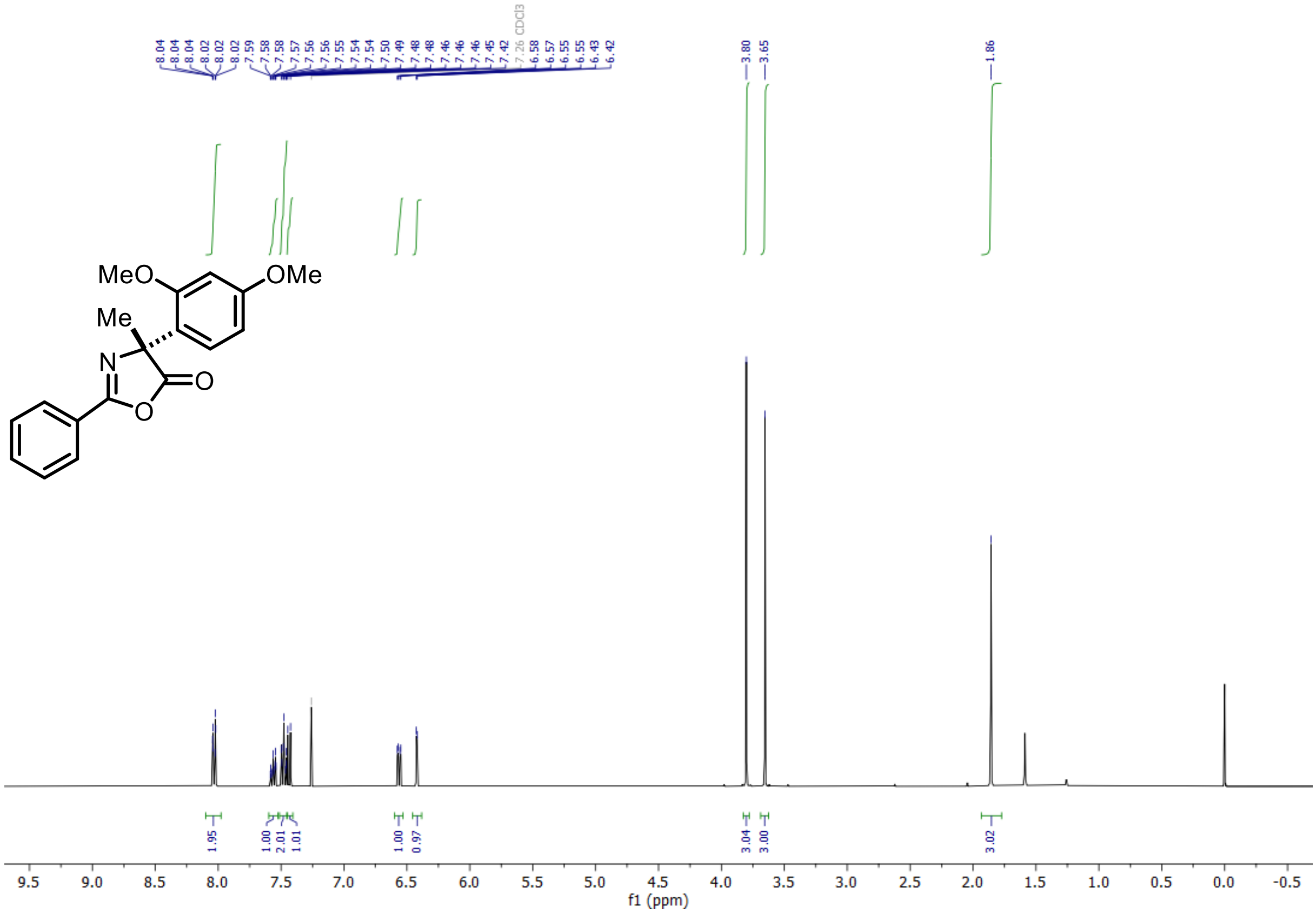

^{1}H NMR spectra of **4p**.

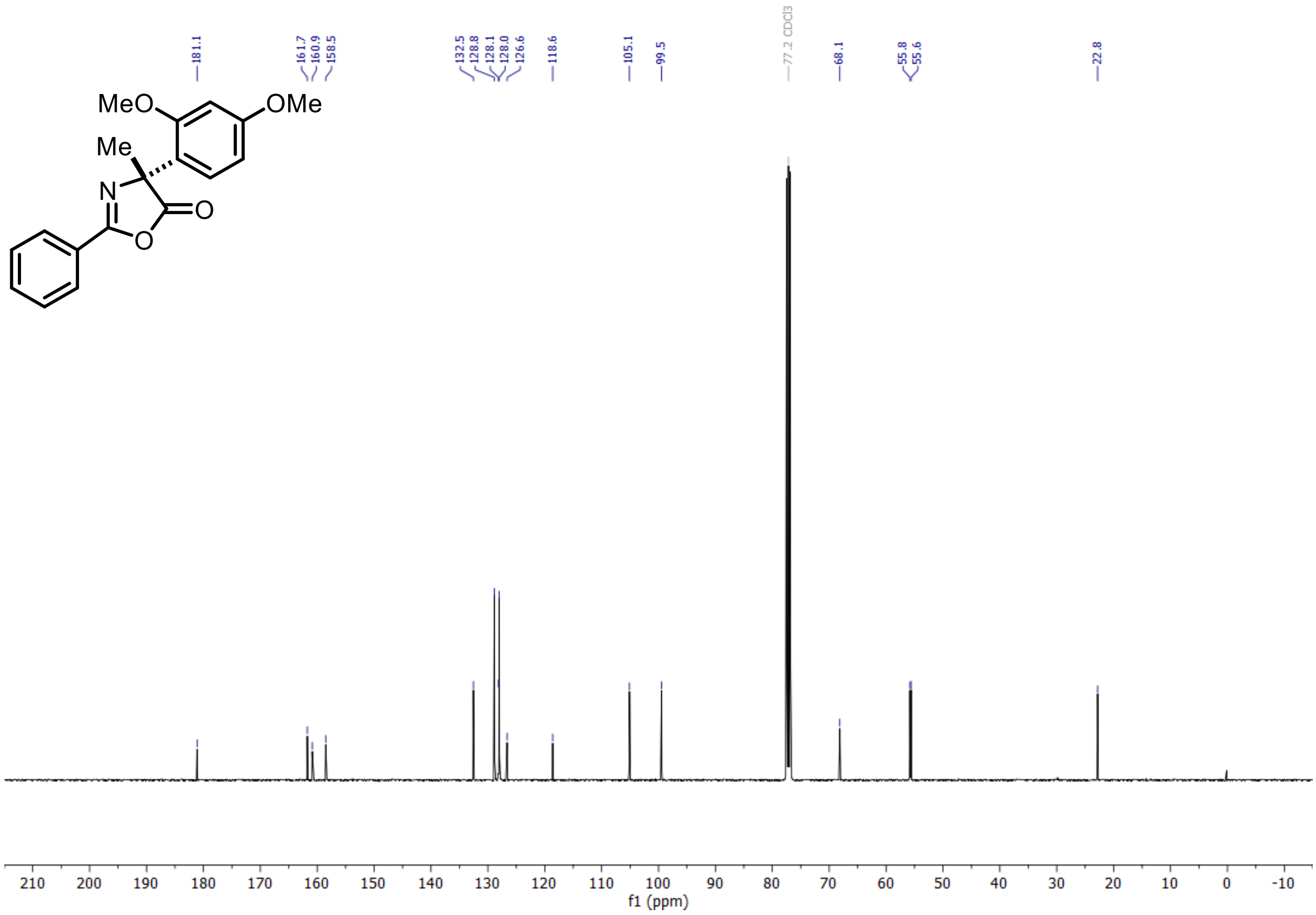

^{13}C NMR spectra of **4p**.

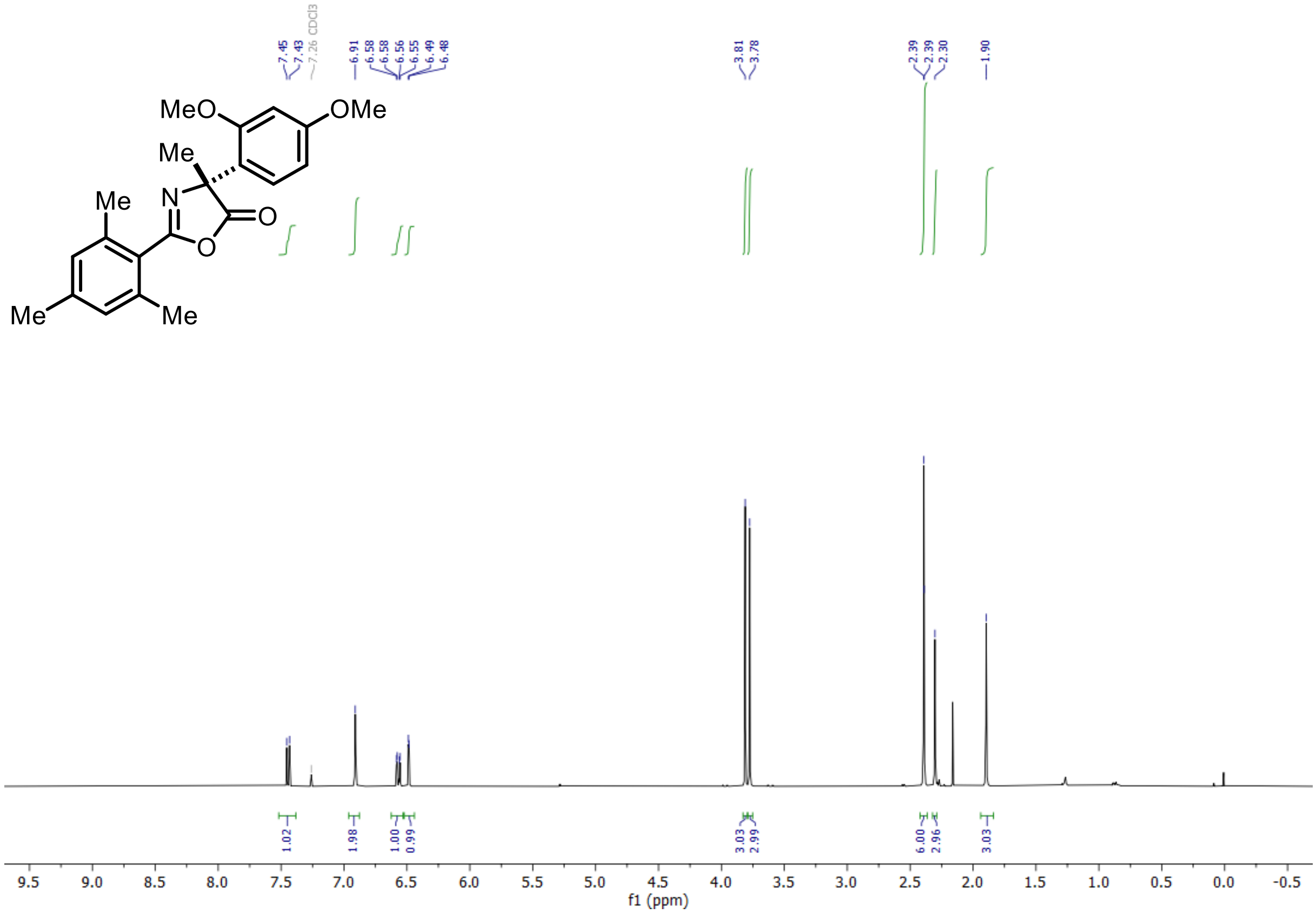

^{1}H NMR spectra of **4q**.

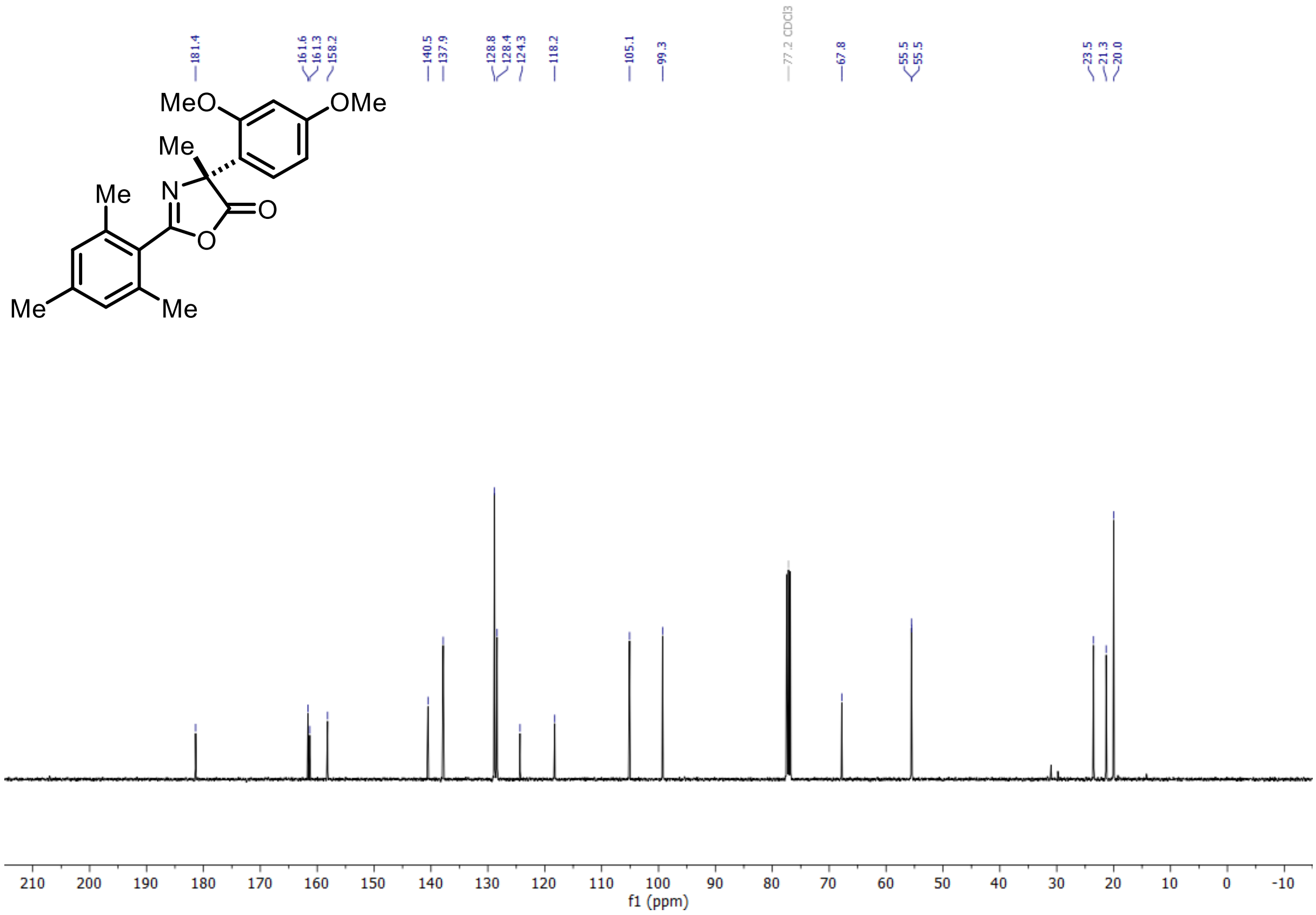

^{13}C NMR spectra of **4q**.

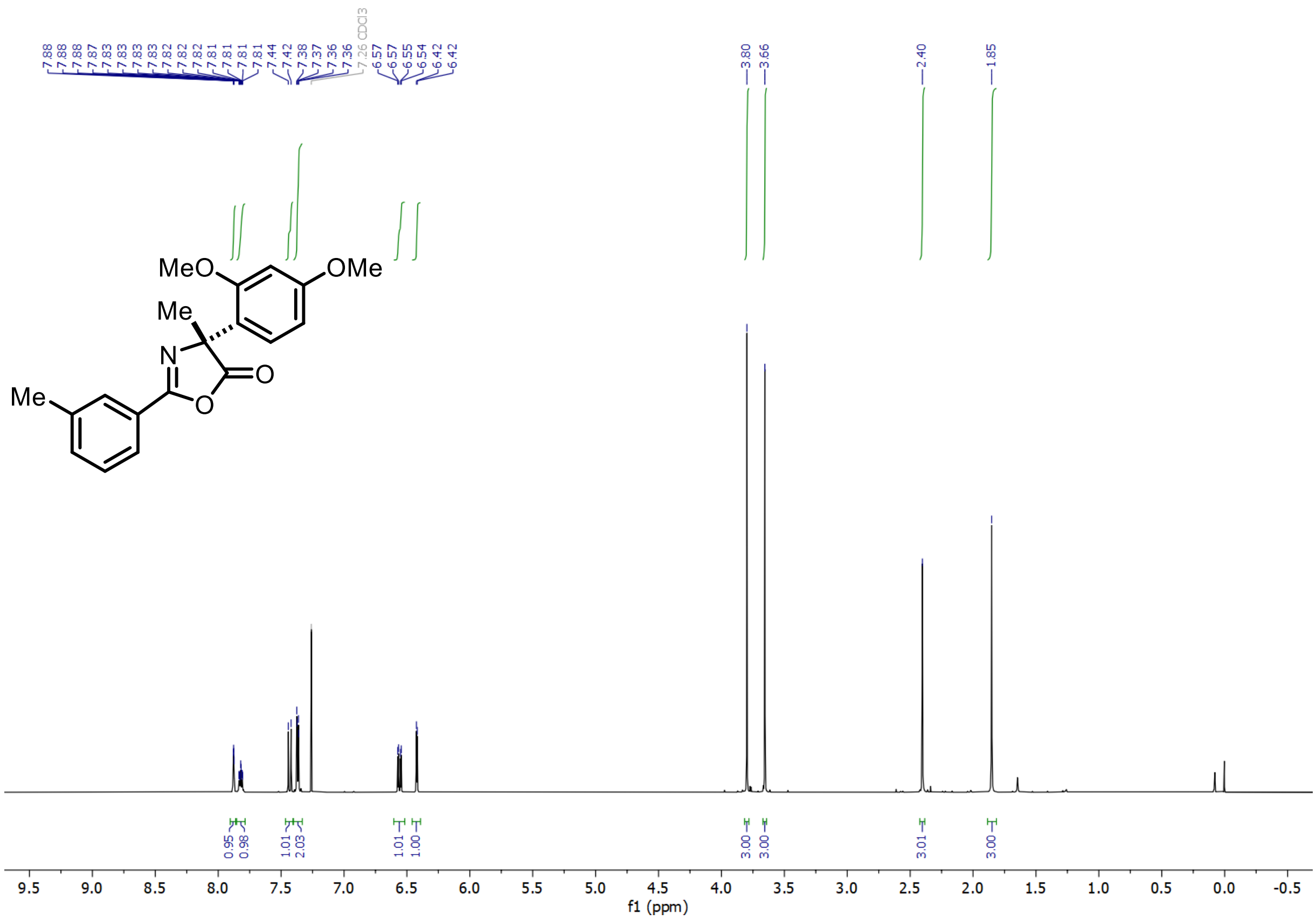

^{1}H NMR spectra of **4r**.

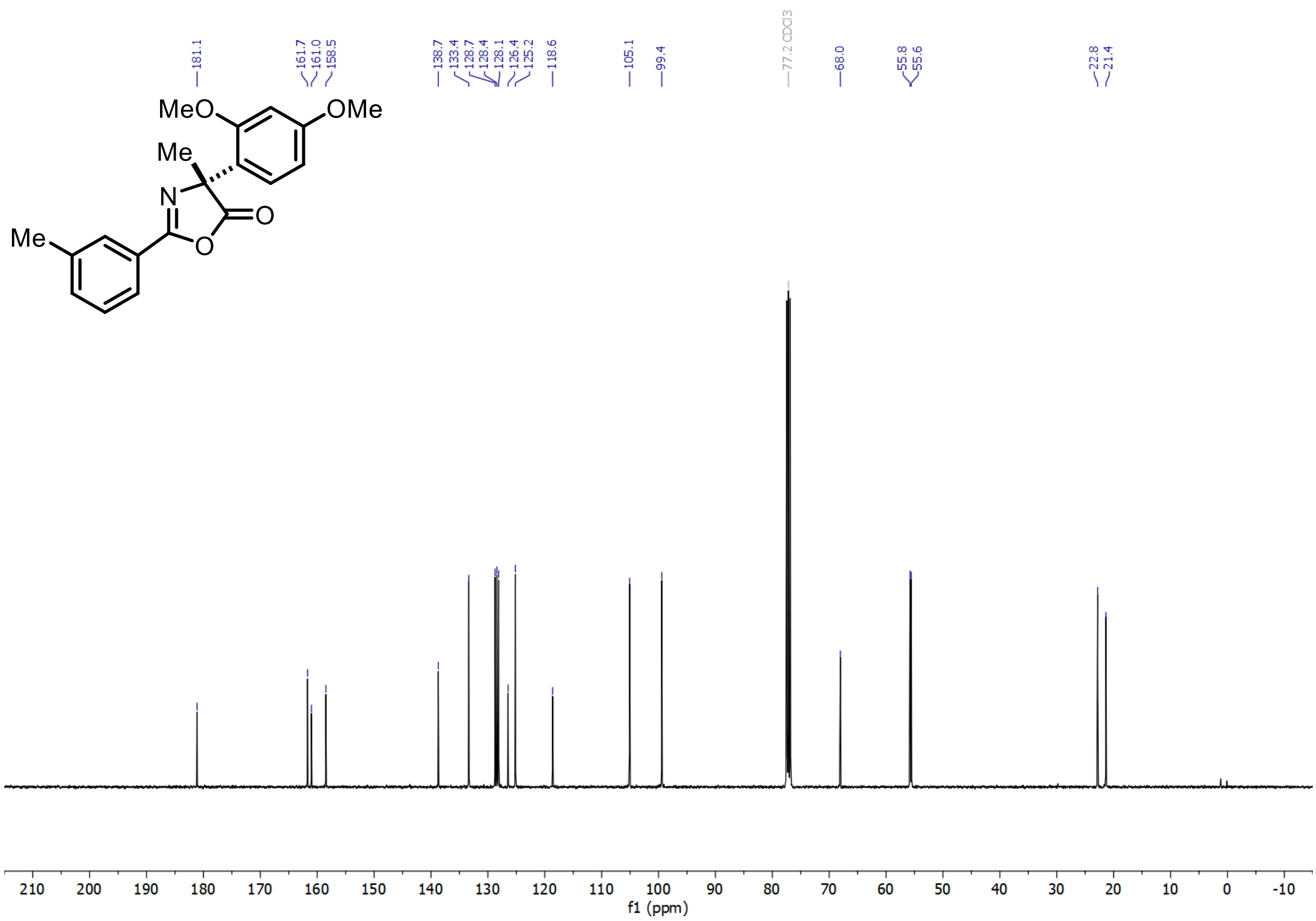

^{13}C NMR spectra of **4r**.

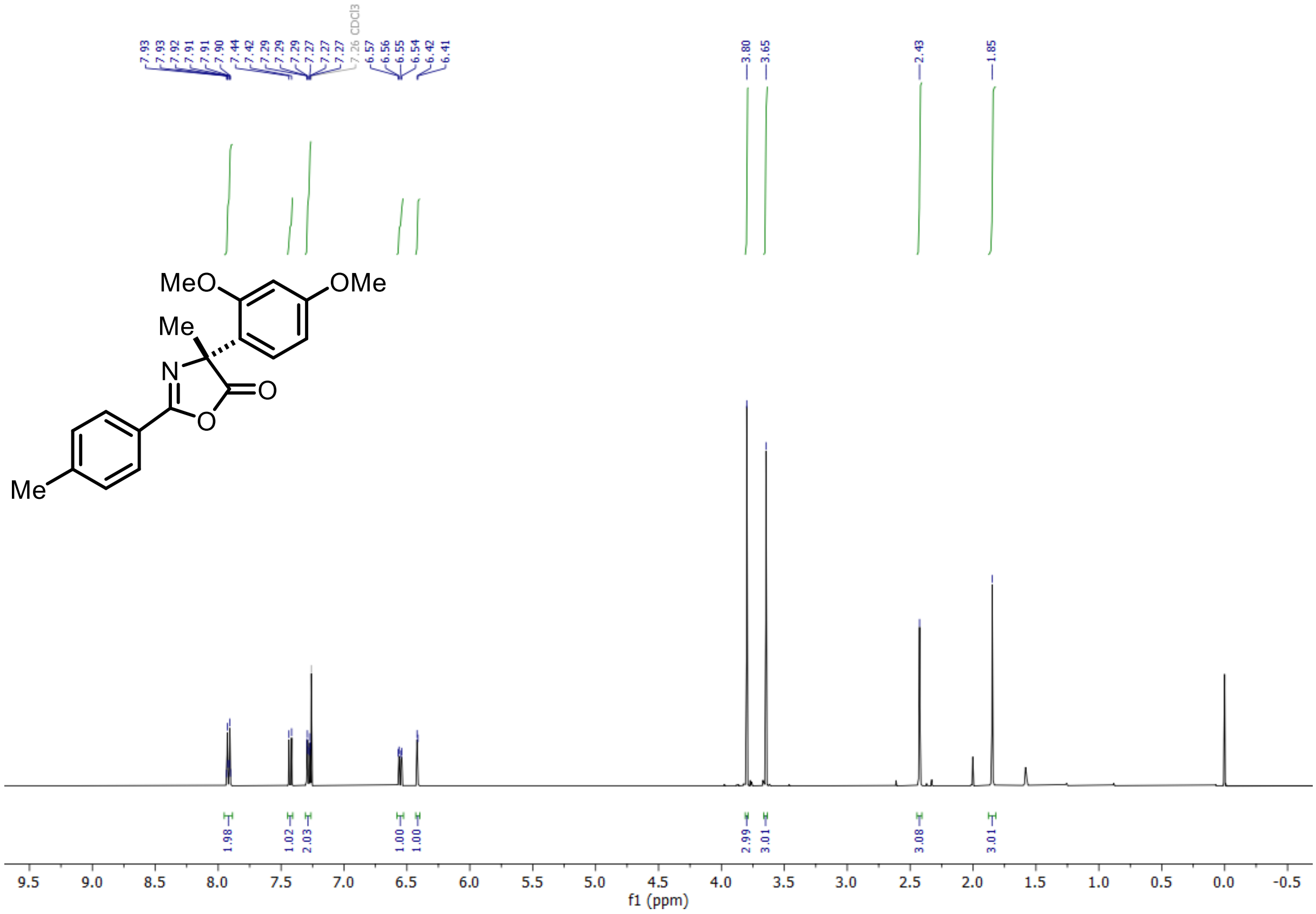

^{1}H NMR spectra of **4s**.

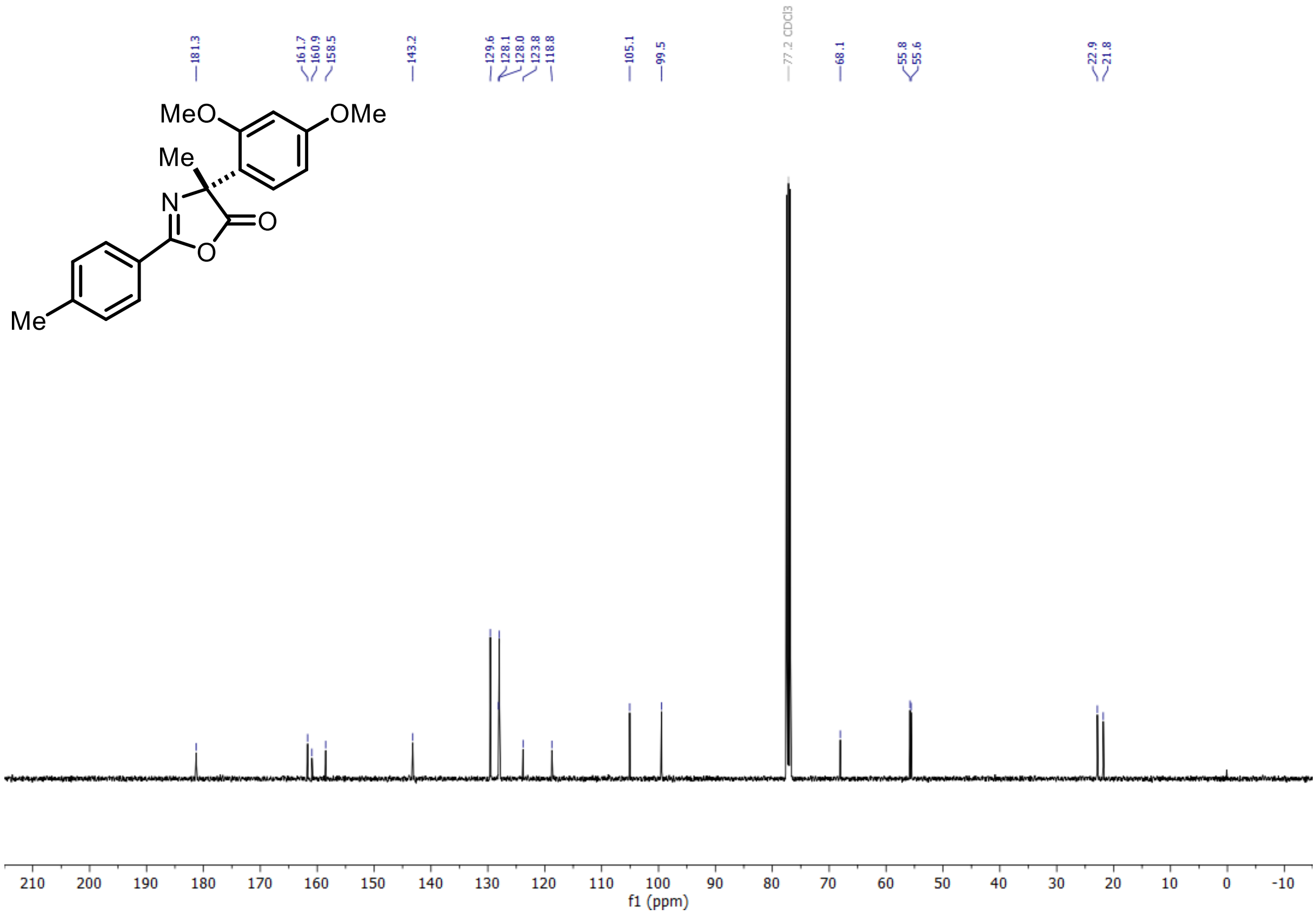

^{13}C NMR spectra of **4s**.

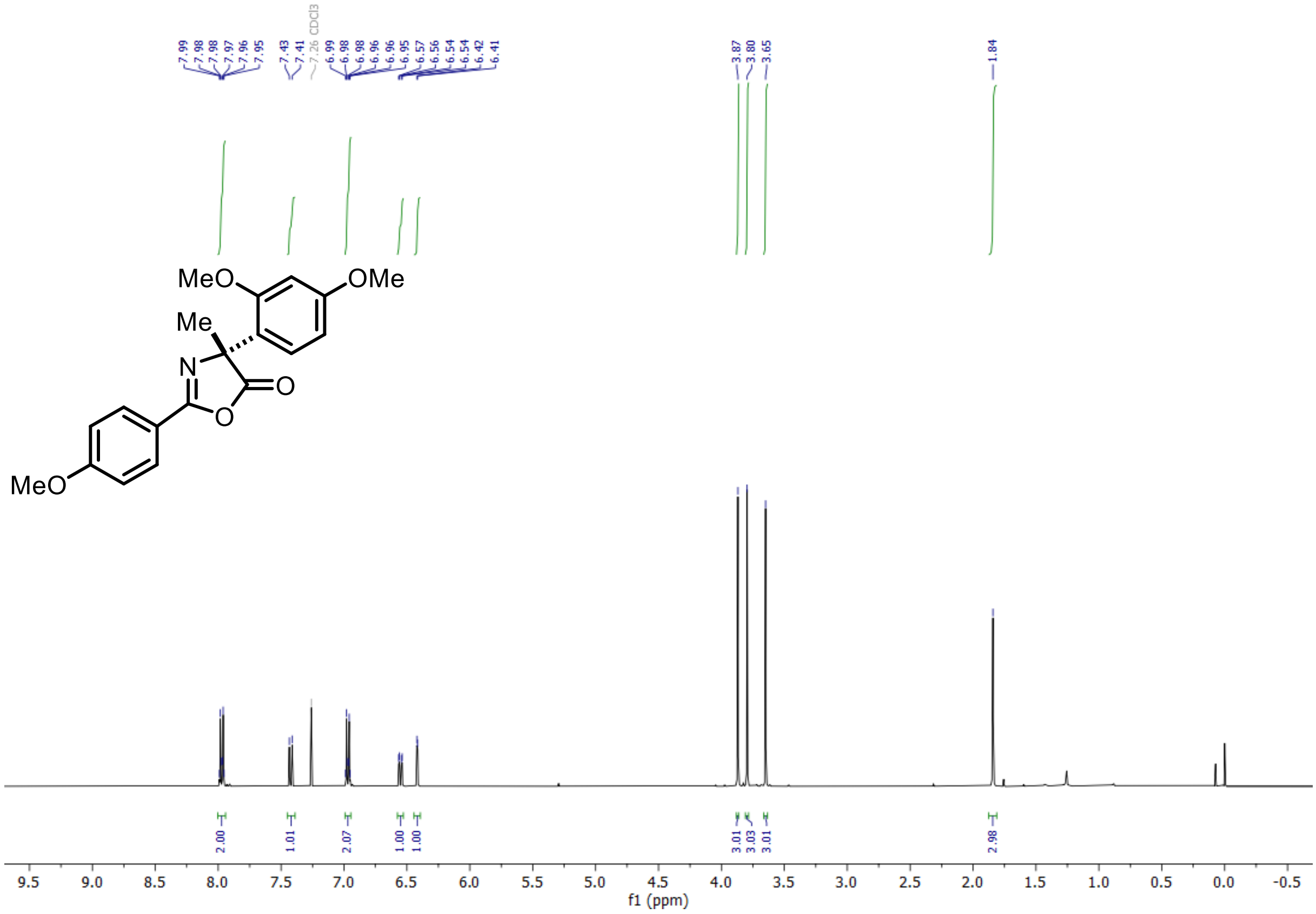

^{1}H NMR spectra of **4t**.

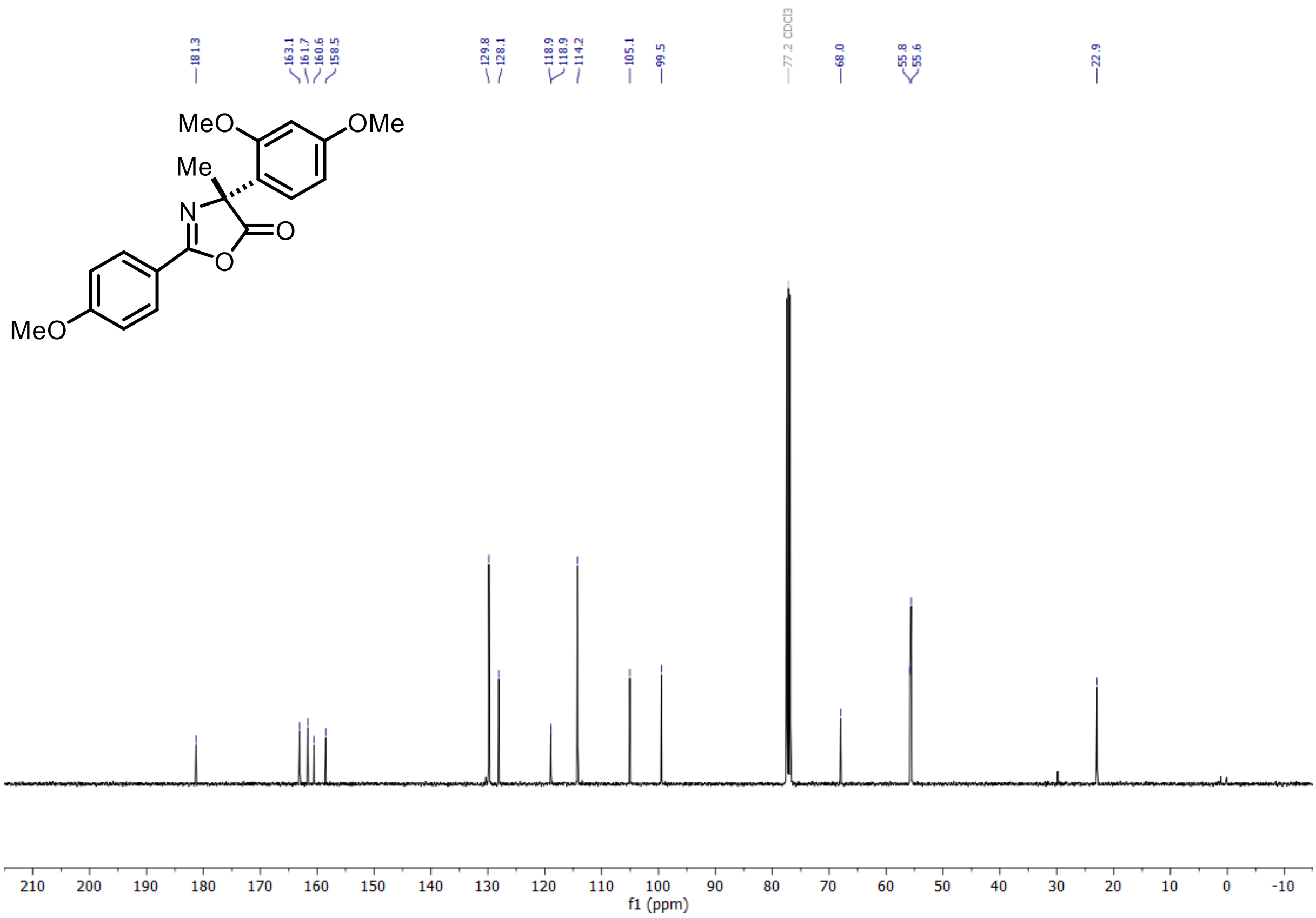

^{13}C NMR spectra of **4t**.

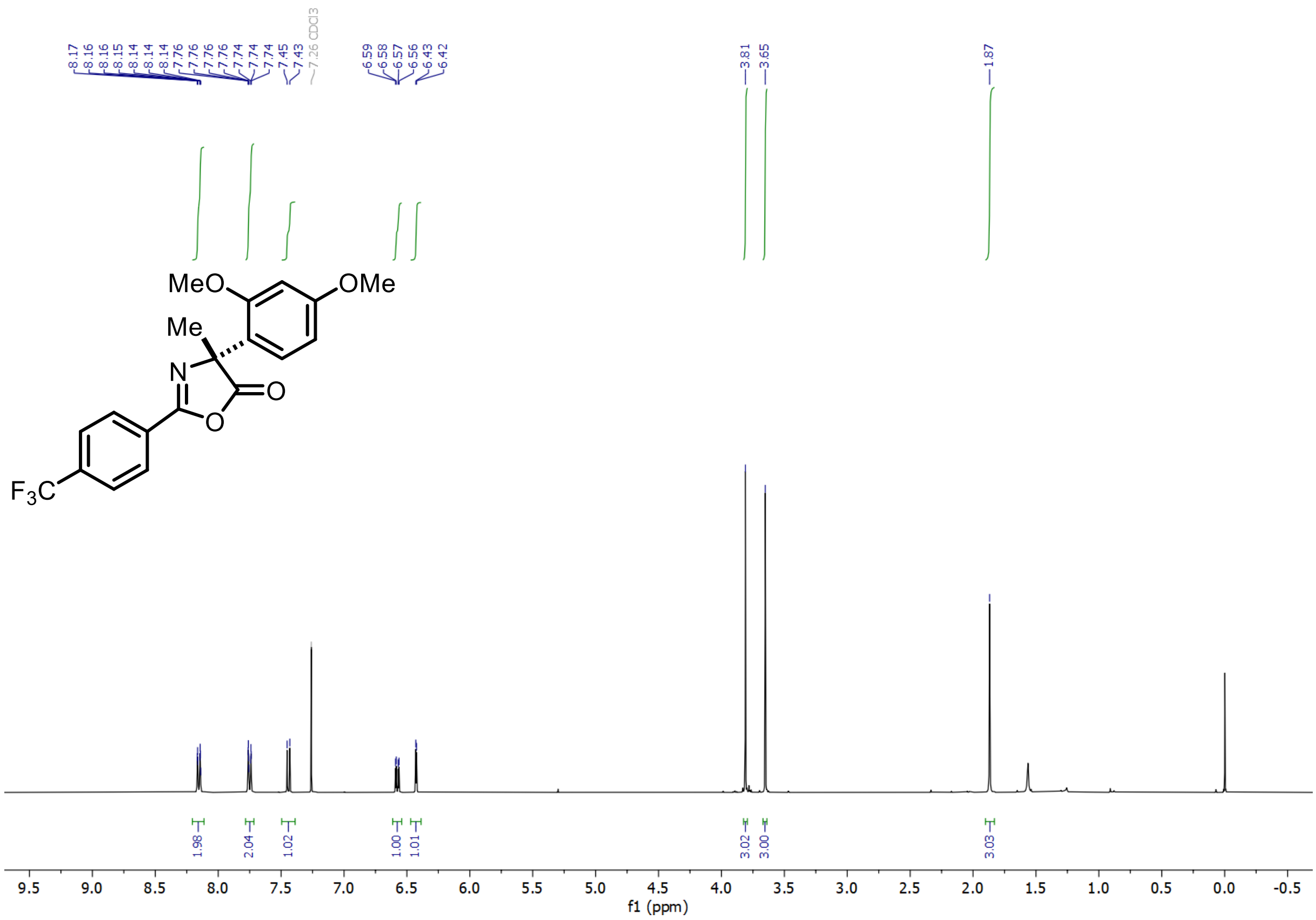

^{1}H NMR spectra of **4u**.

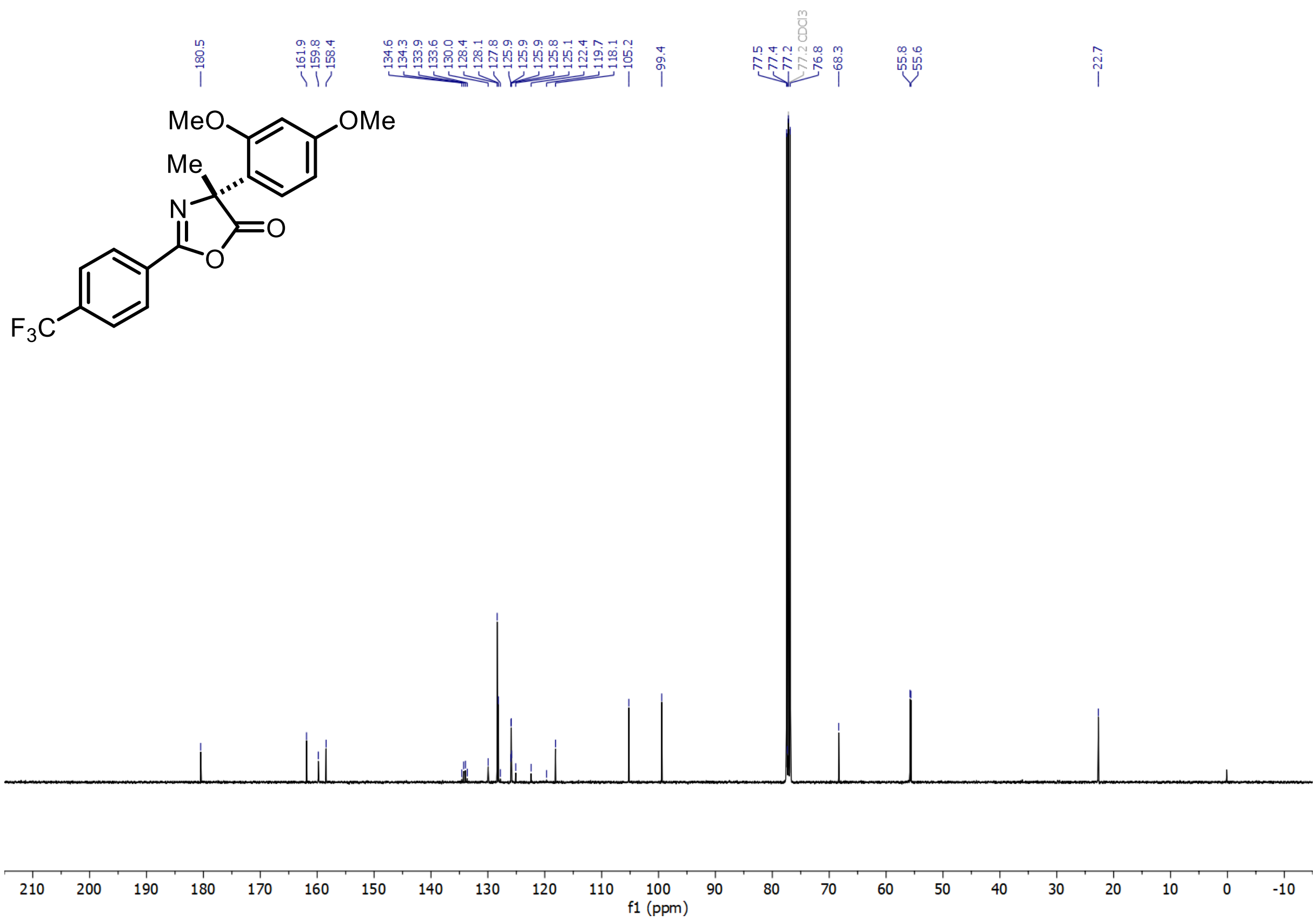

^{13}C NMR spectra of **4u**.

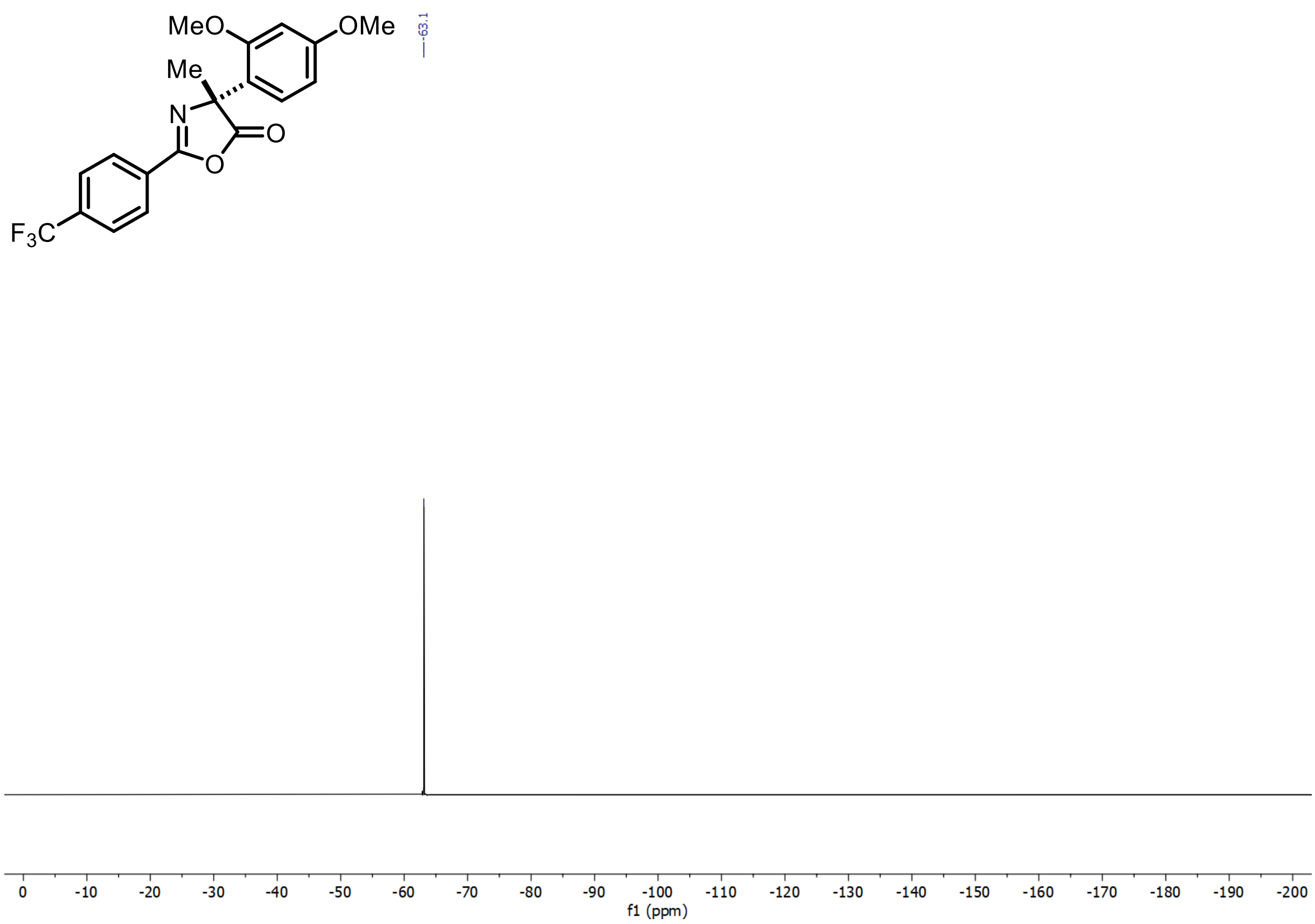

^{19}F NMR spectra of **4u**.

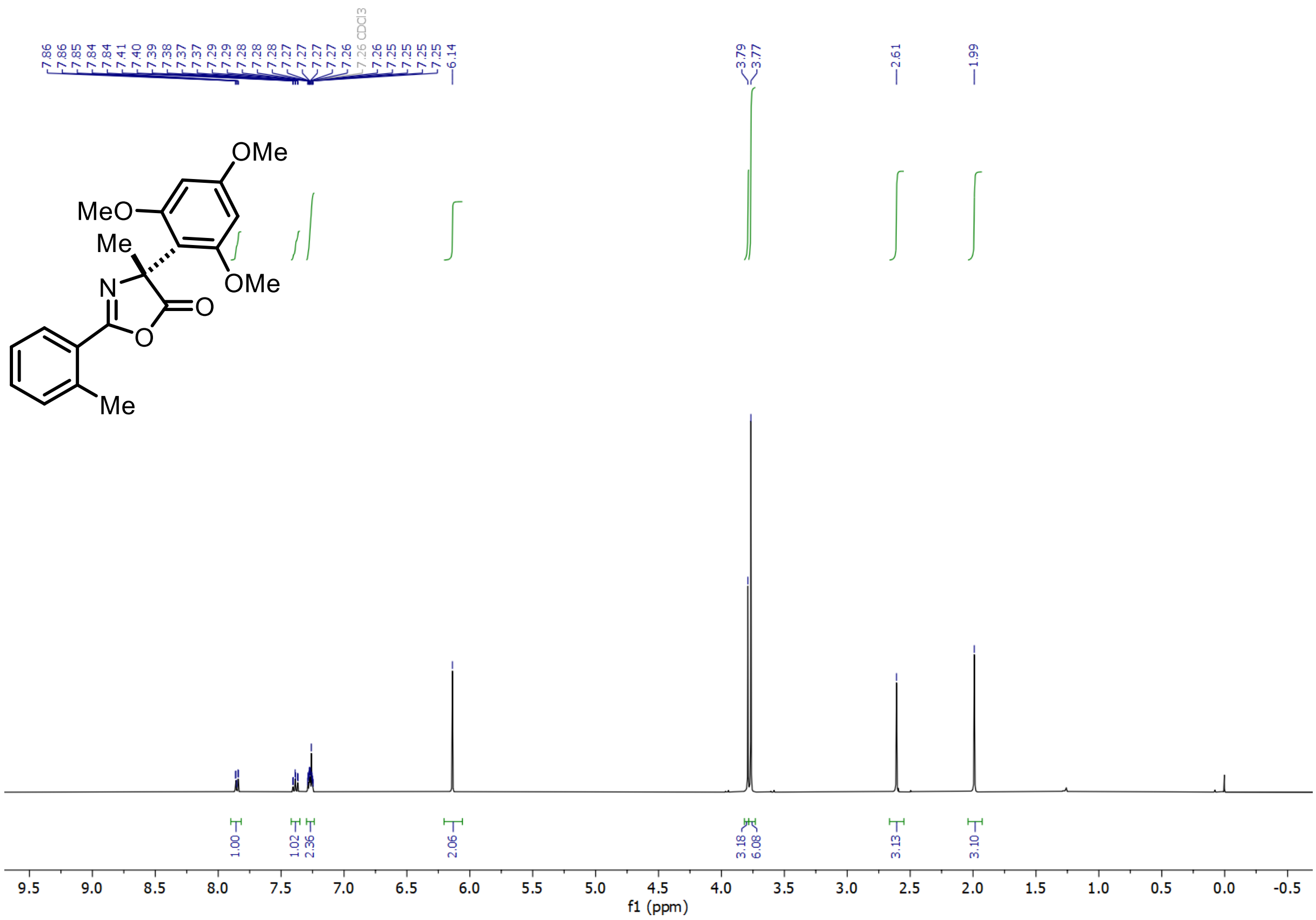

^{1}H NMR spectra of **4v**.

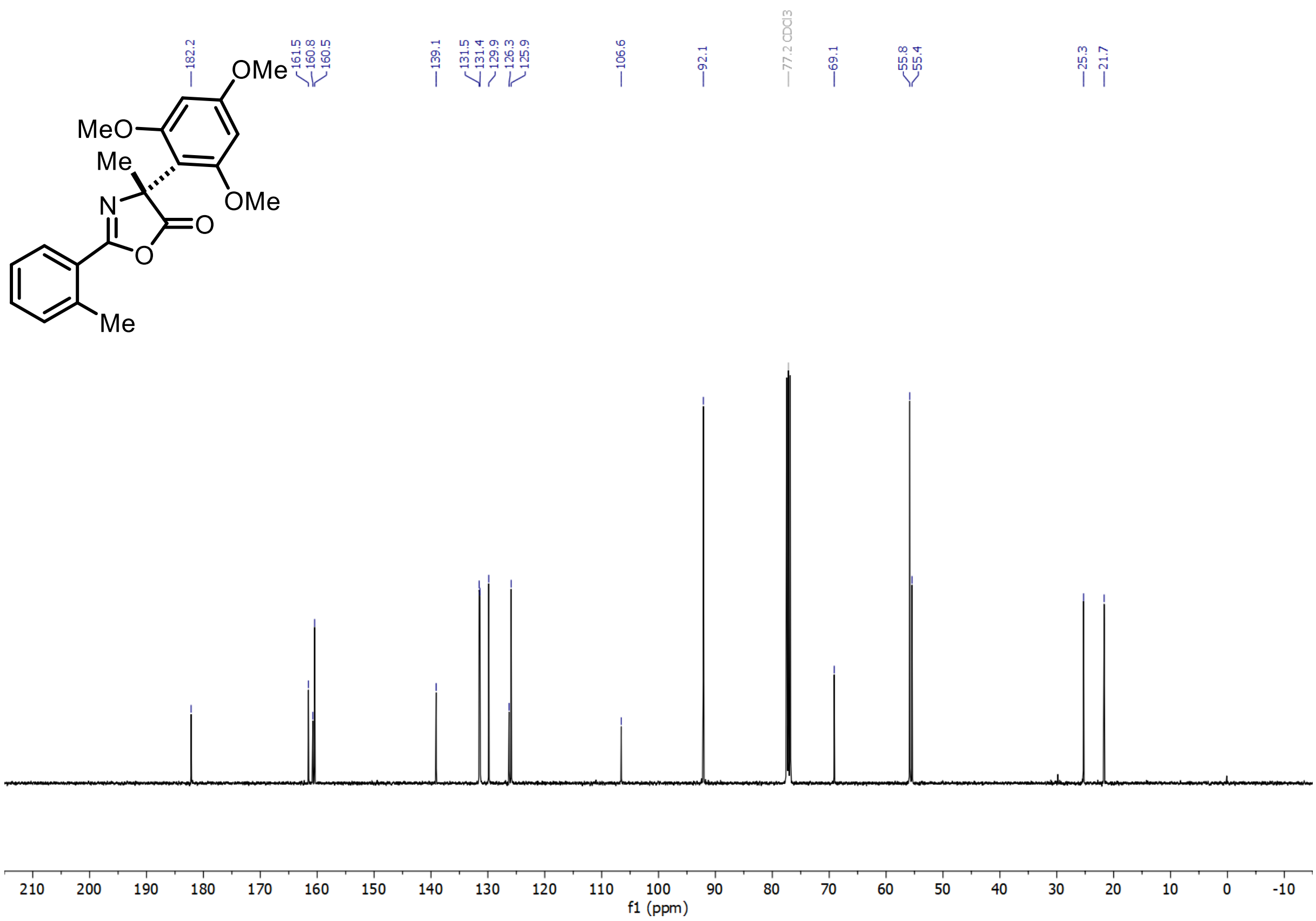

^{13}C NMR spectra of **4v**.

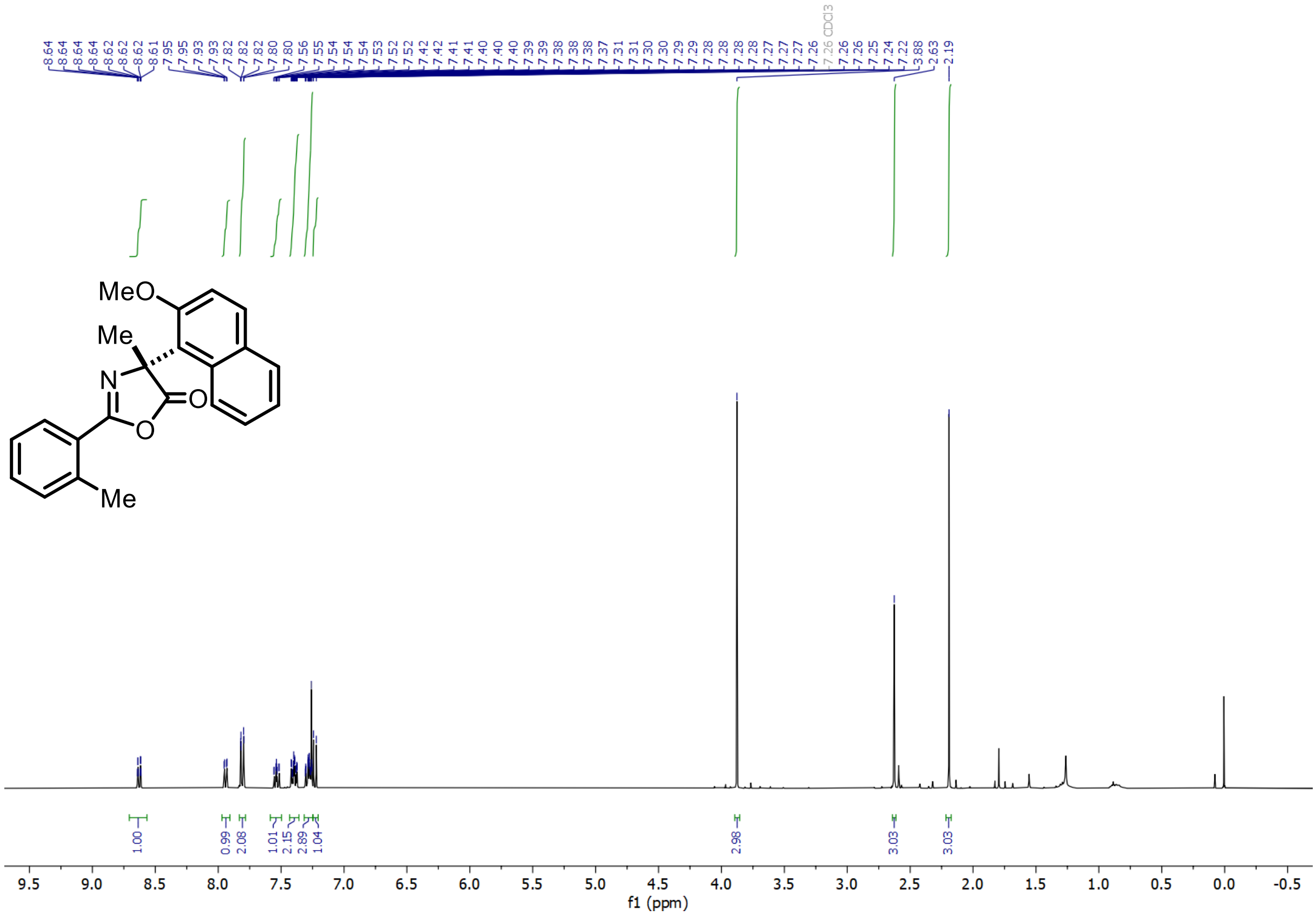

^{1}H NMR spectra of **4w**.

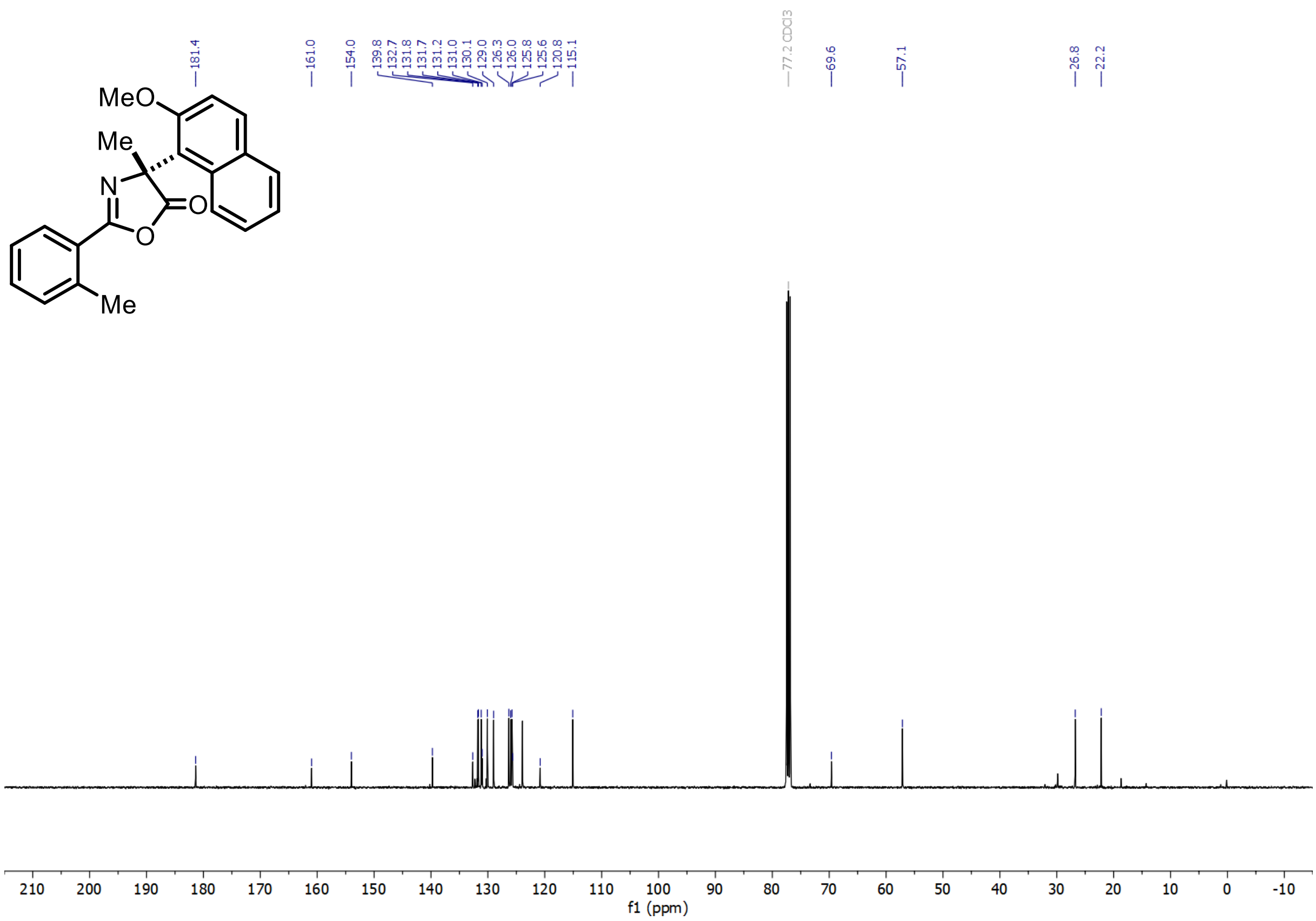

^{13}C NMR spectra of **4w**.

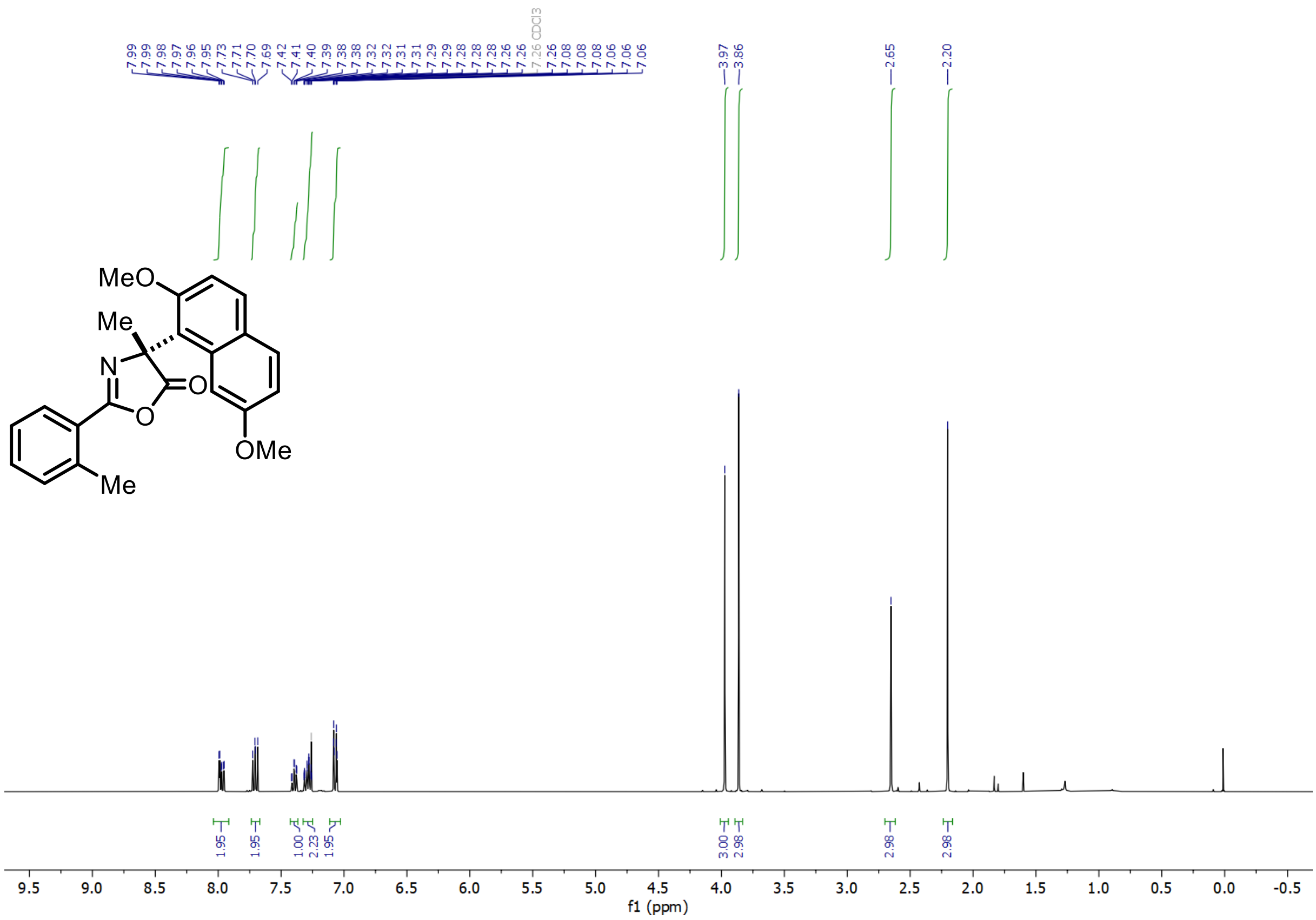

^{1}H NMR spectra of **4x**.

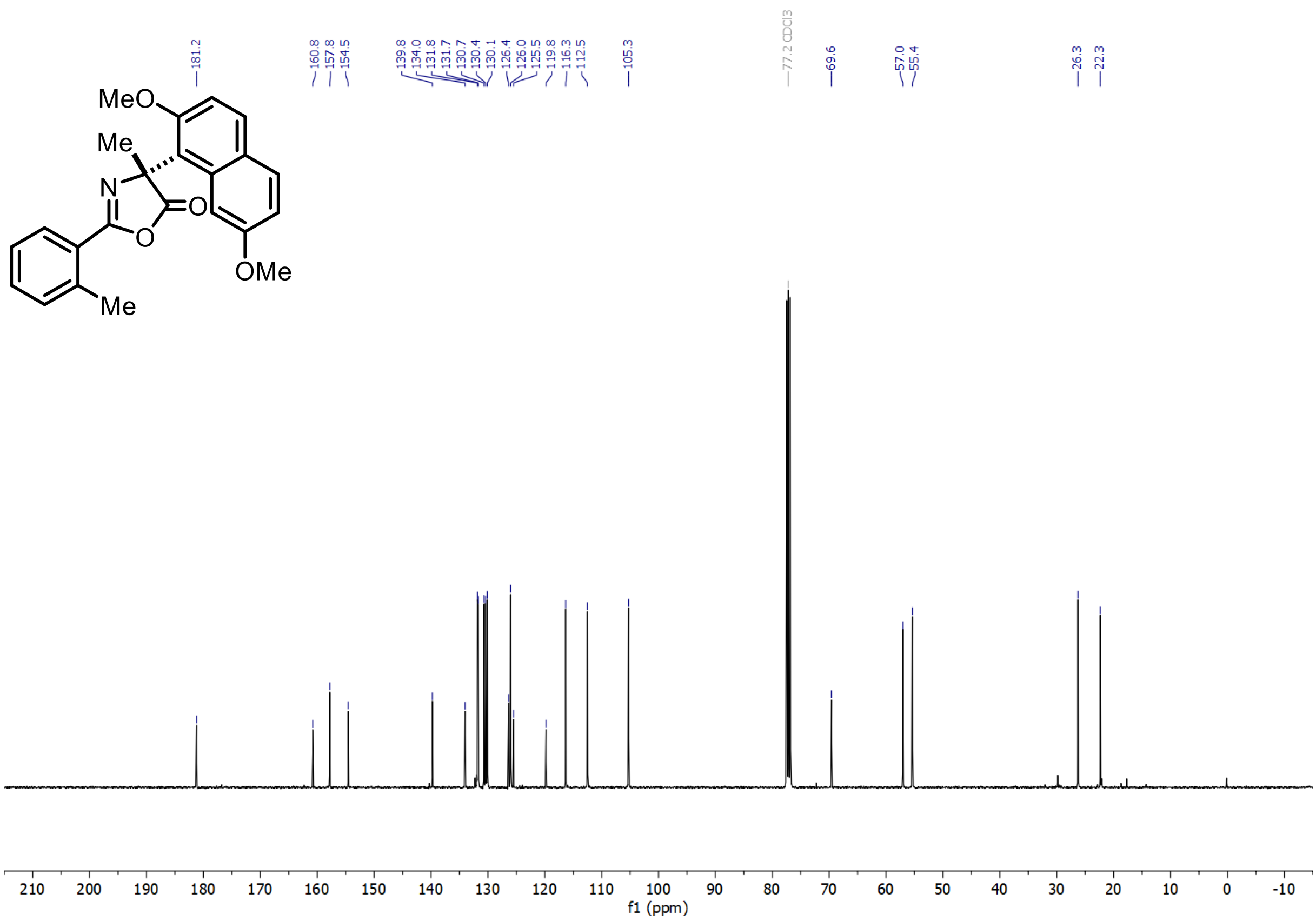

^{13}C NMR spectra of **4x**.

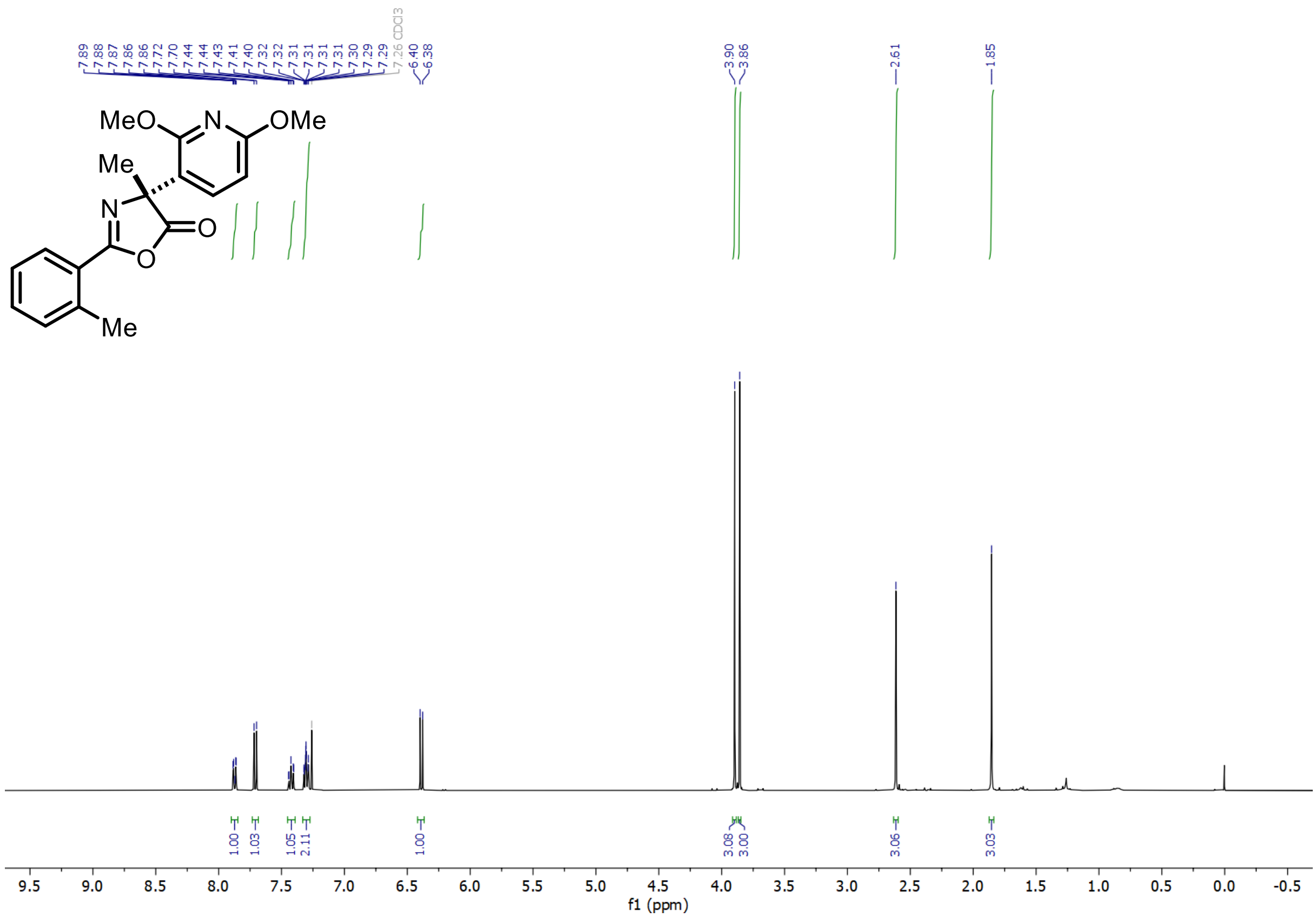

^{1}H NMR spectra of **4y**.

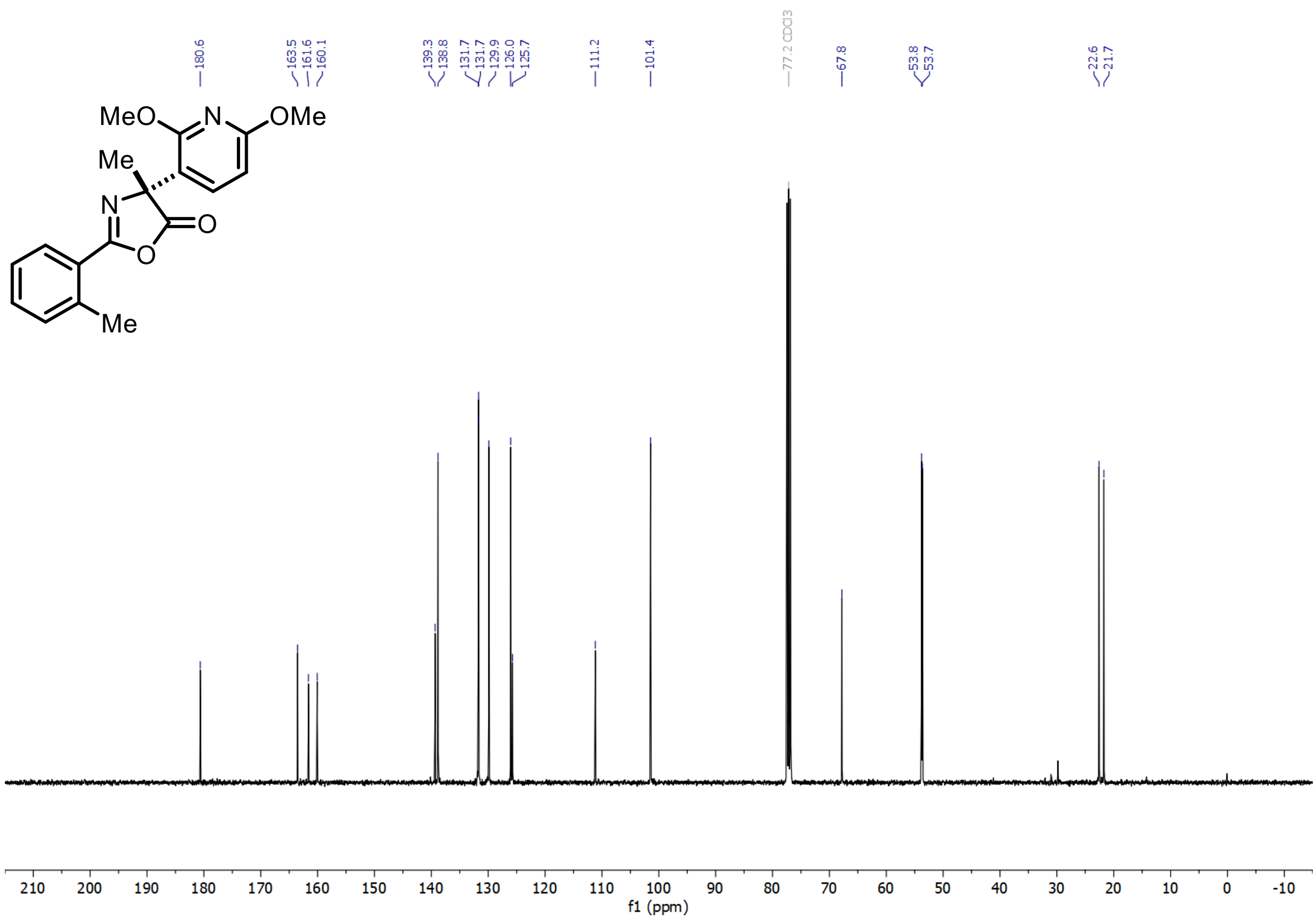

^{13}C NMR spectra of **4y**.

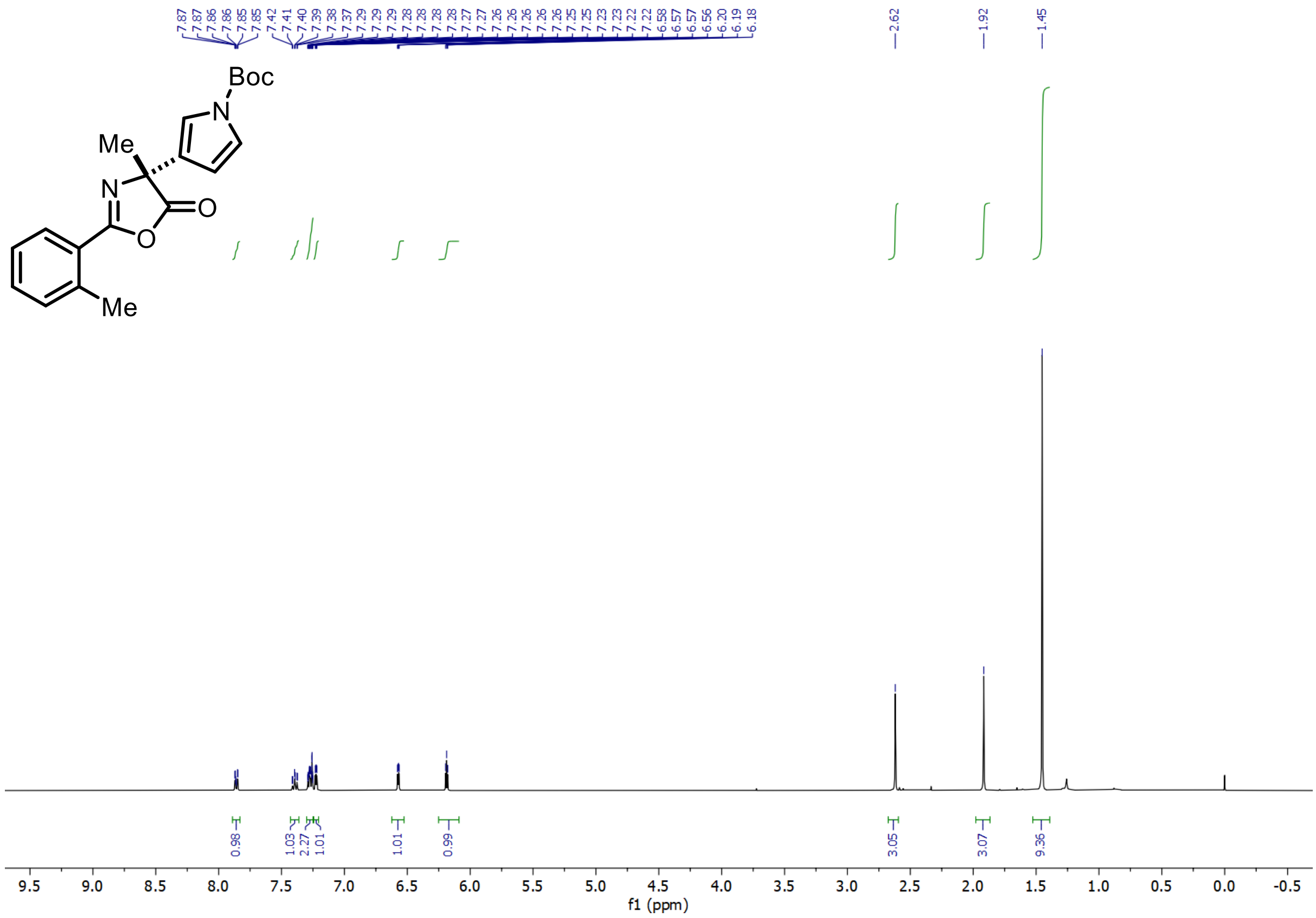

^{1}H NMR spectra of **4z**.

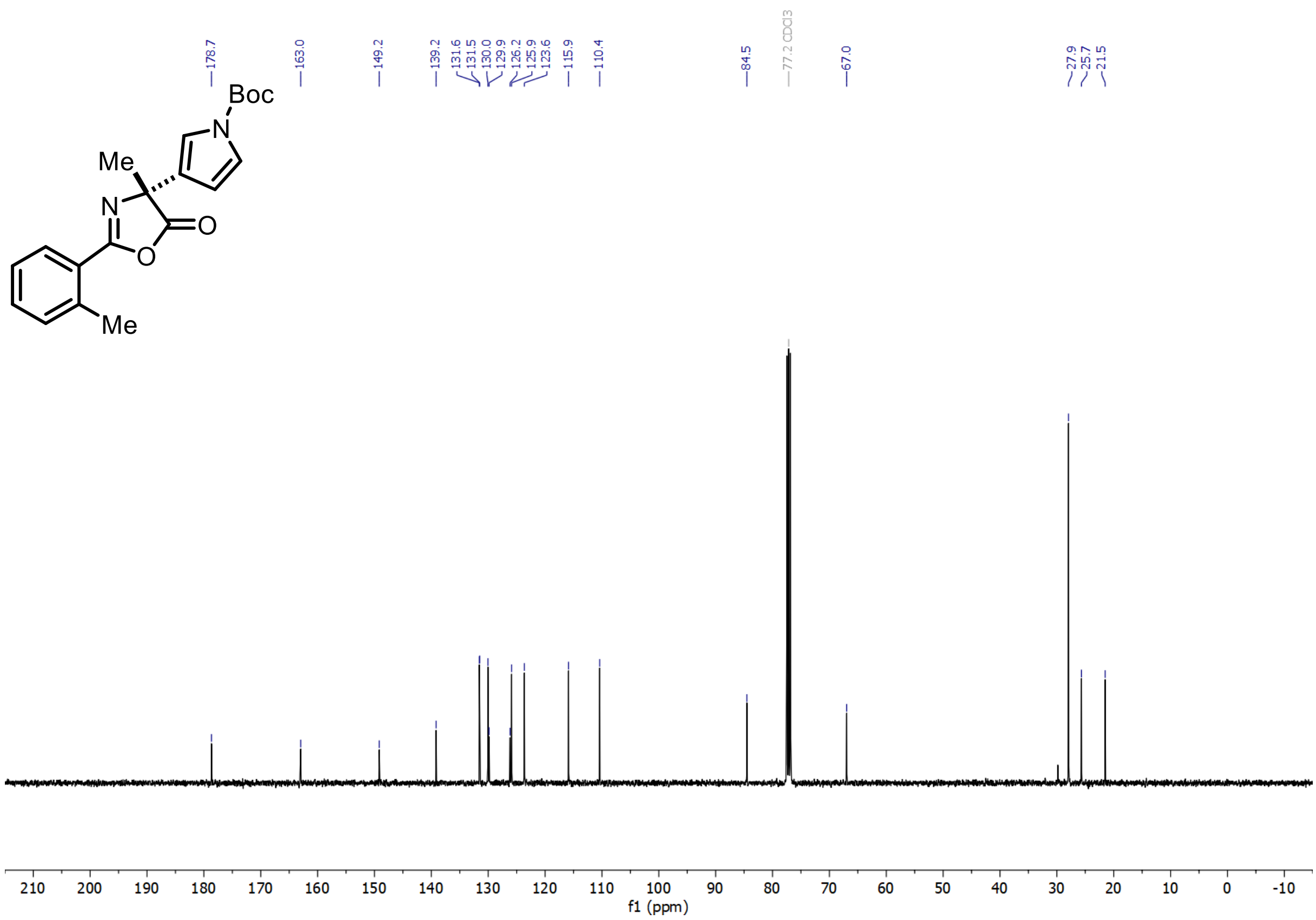

^{13}C NMR spectra of **4z**.

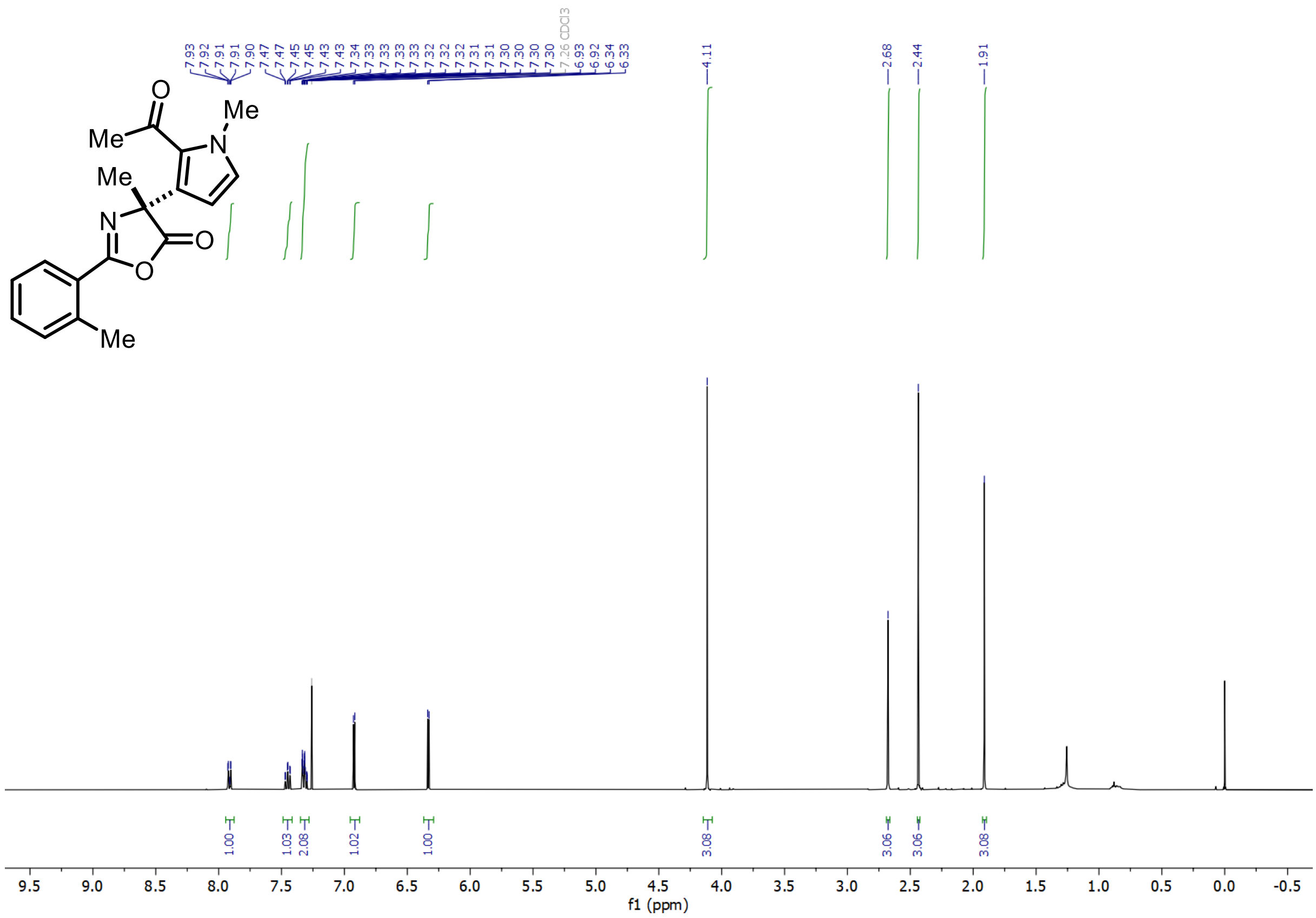

^{1}H NMR spectra of **4aaα**.

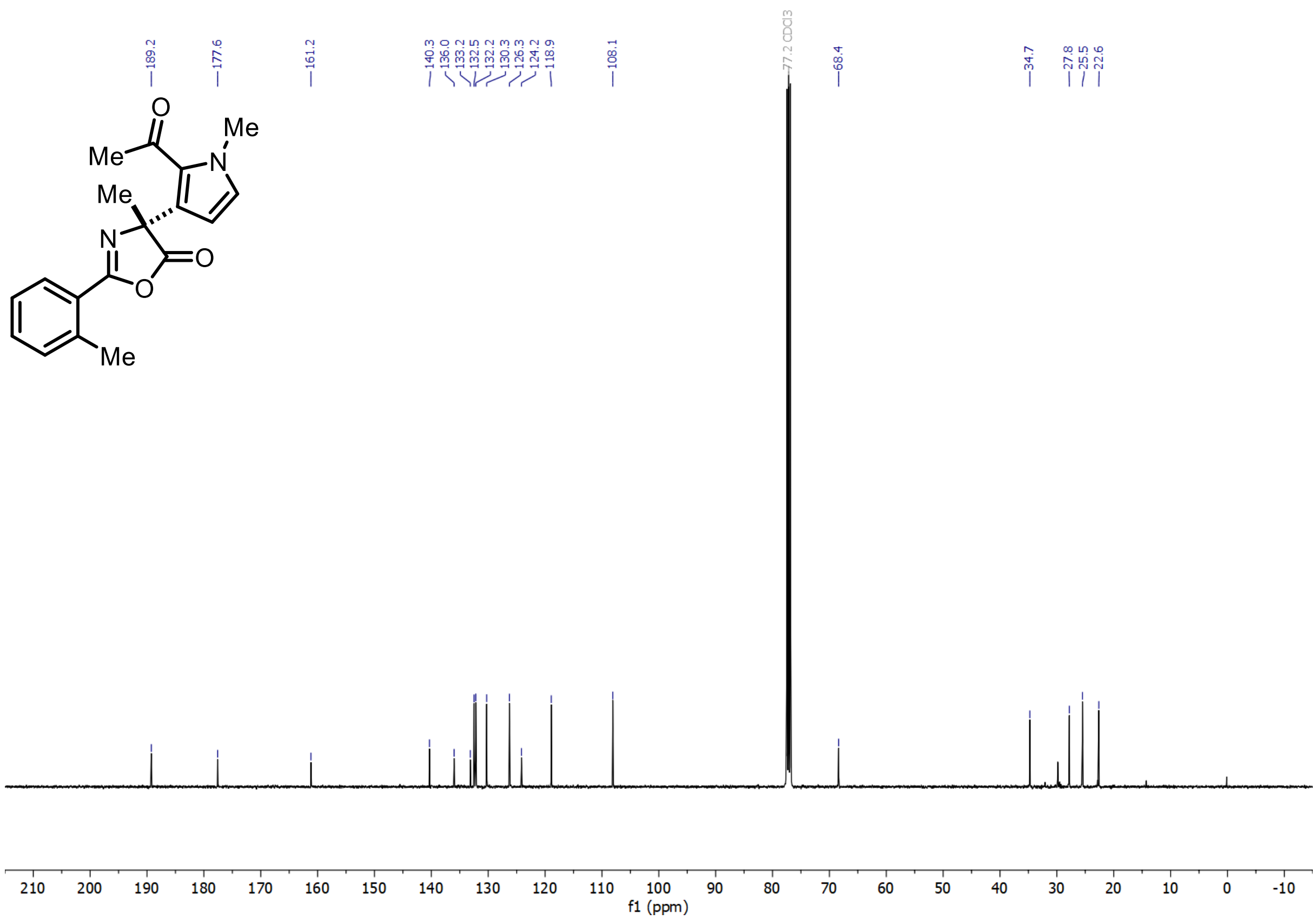

^{13}C NMR spectra of **4aaα**.

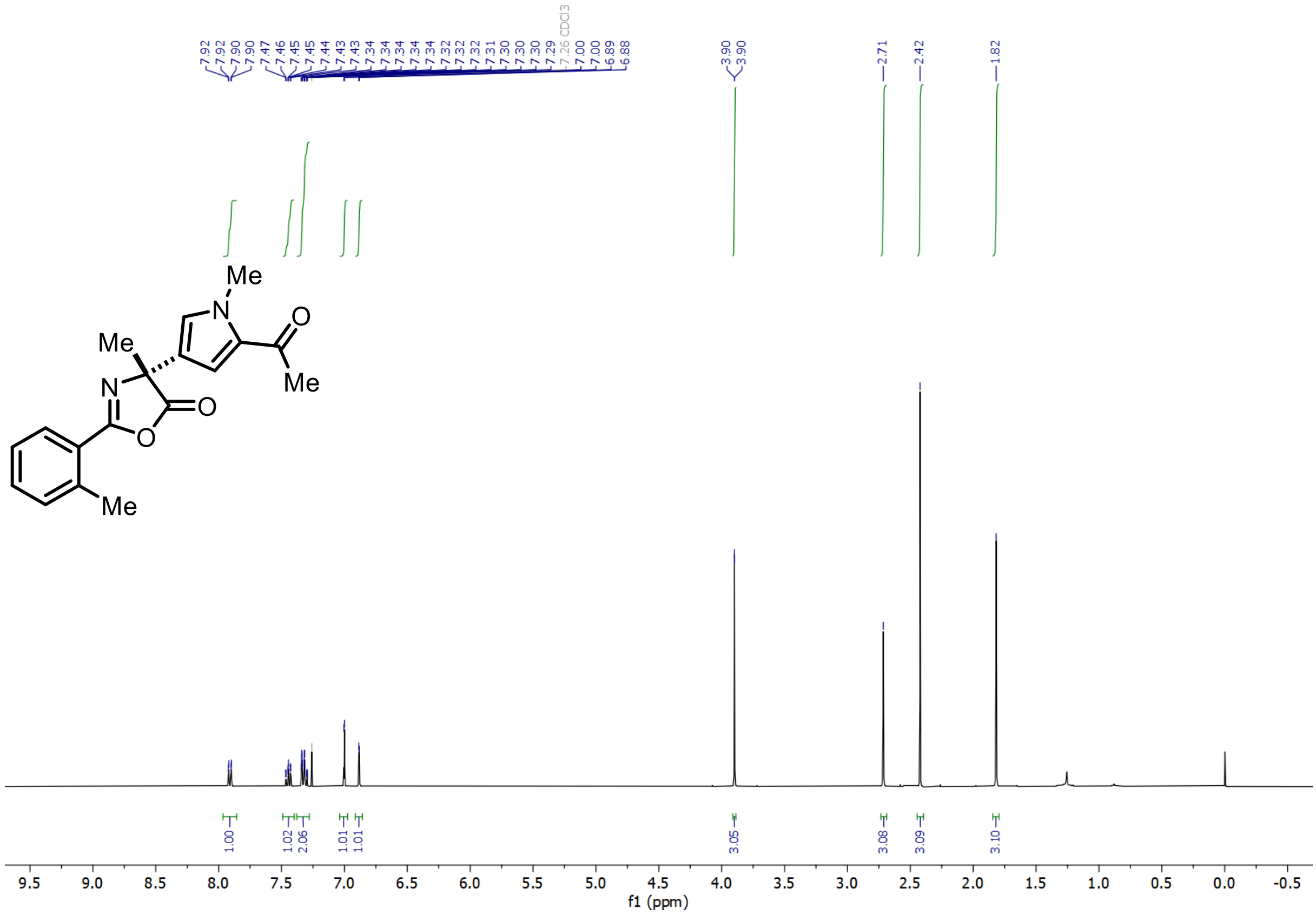

^{1}H NMR spectra of **4aaβ**.

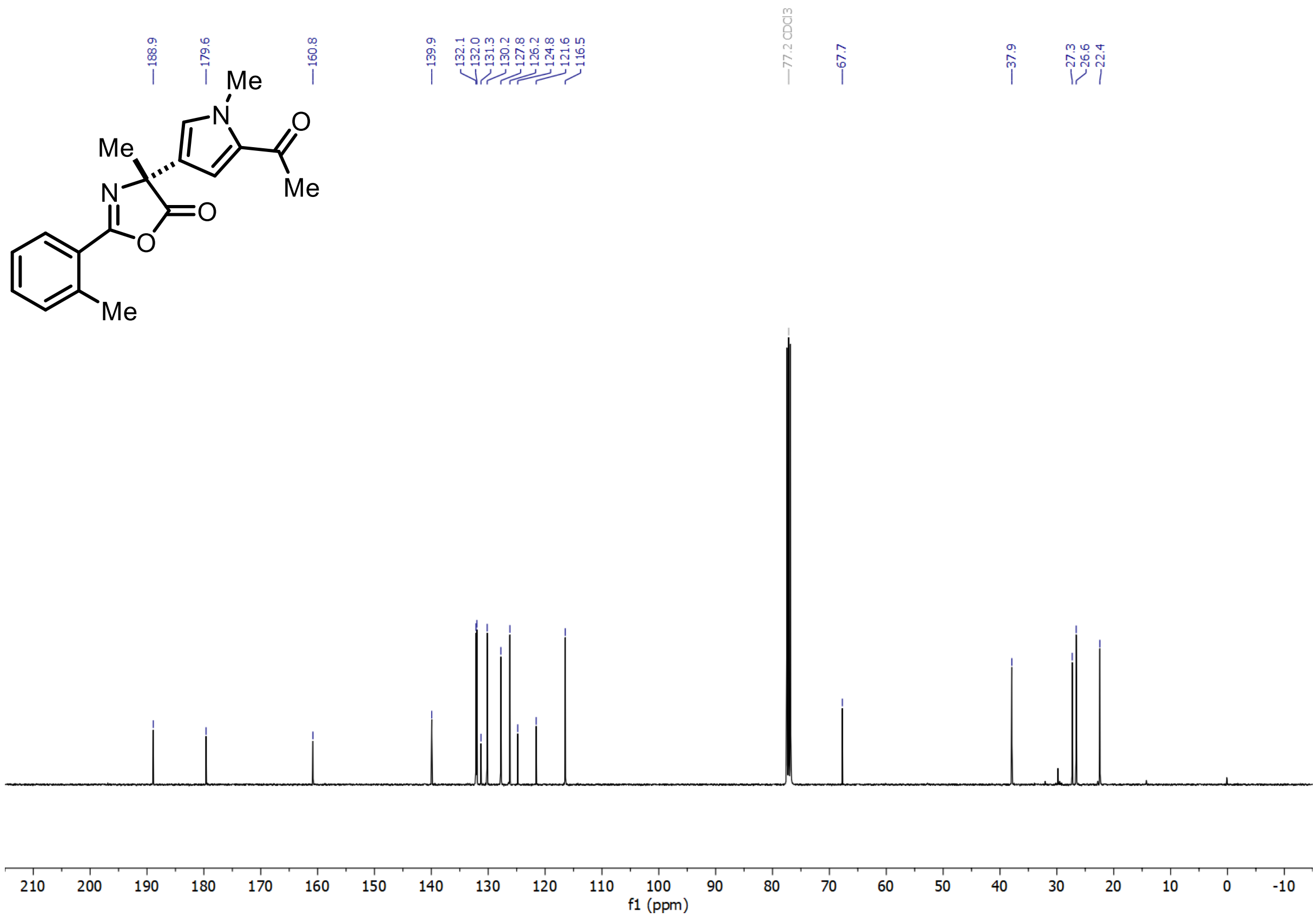

^{13}C NMR spectra of **4aaβ**.

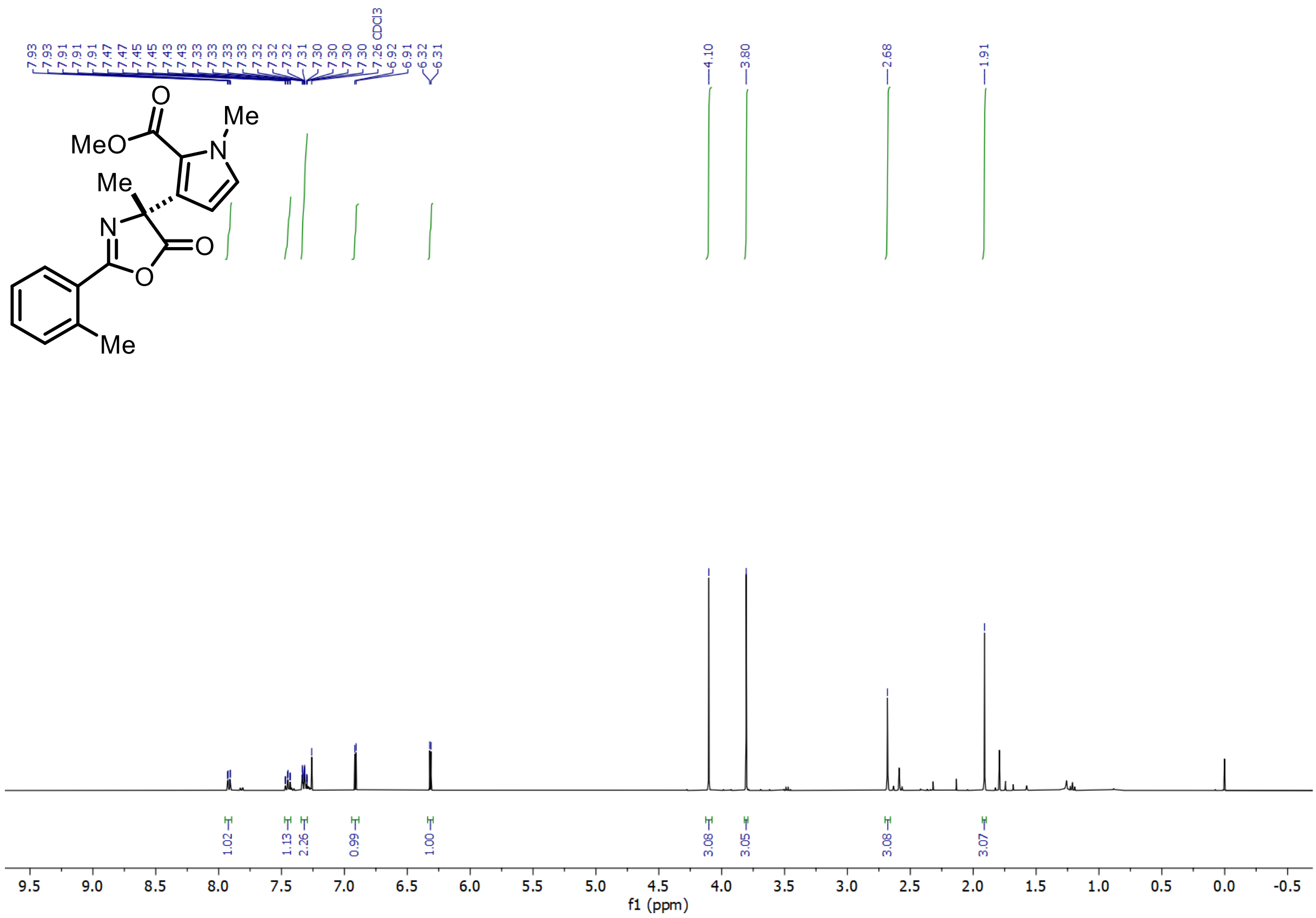

^{1}H NMR spectra of **4abα**.

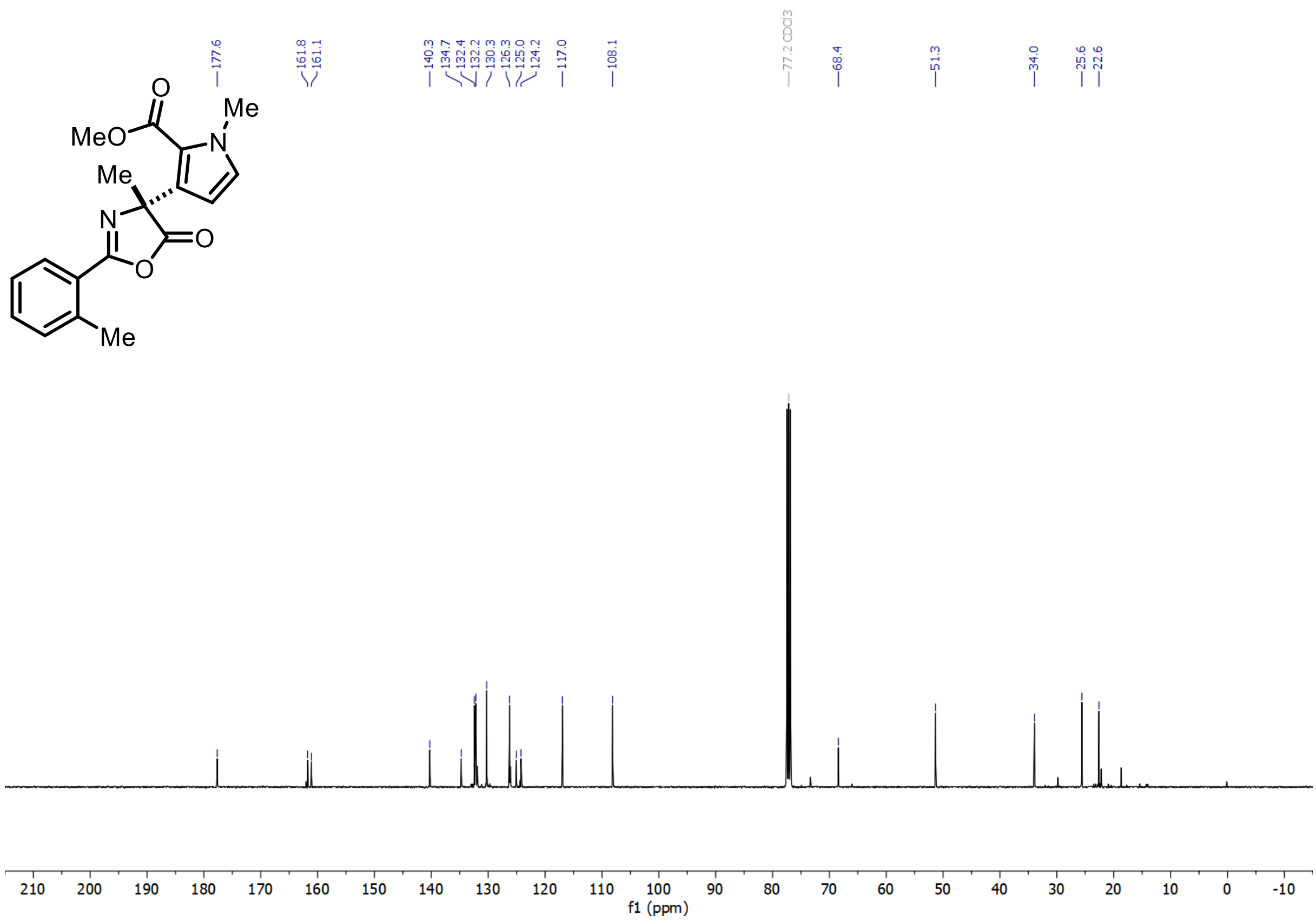

^{13}C NMR spectra of **4abα**.

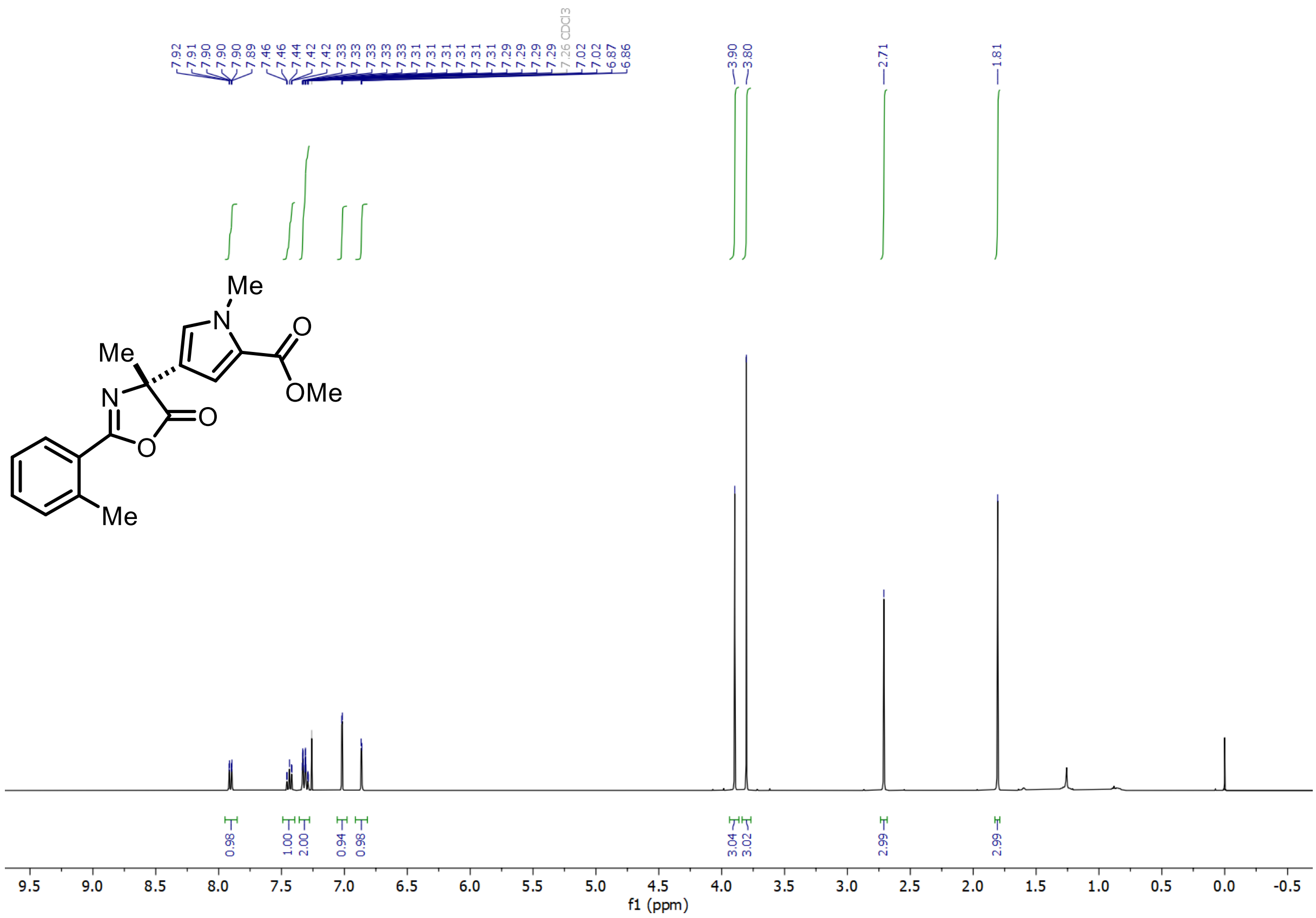

^{1}H NMR spectra of **4abβ**.

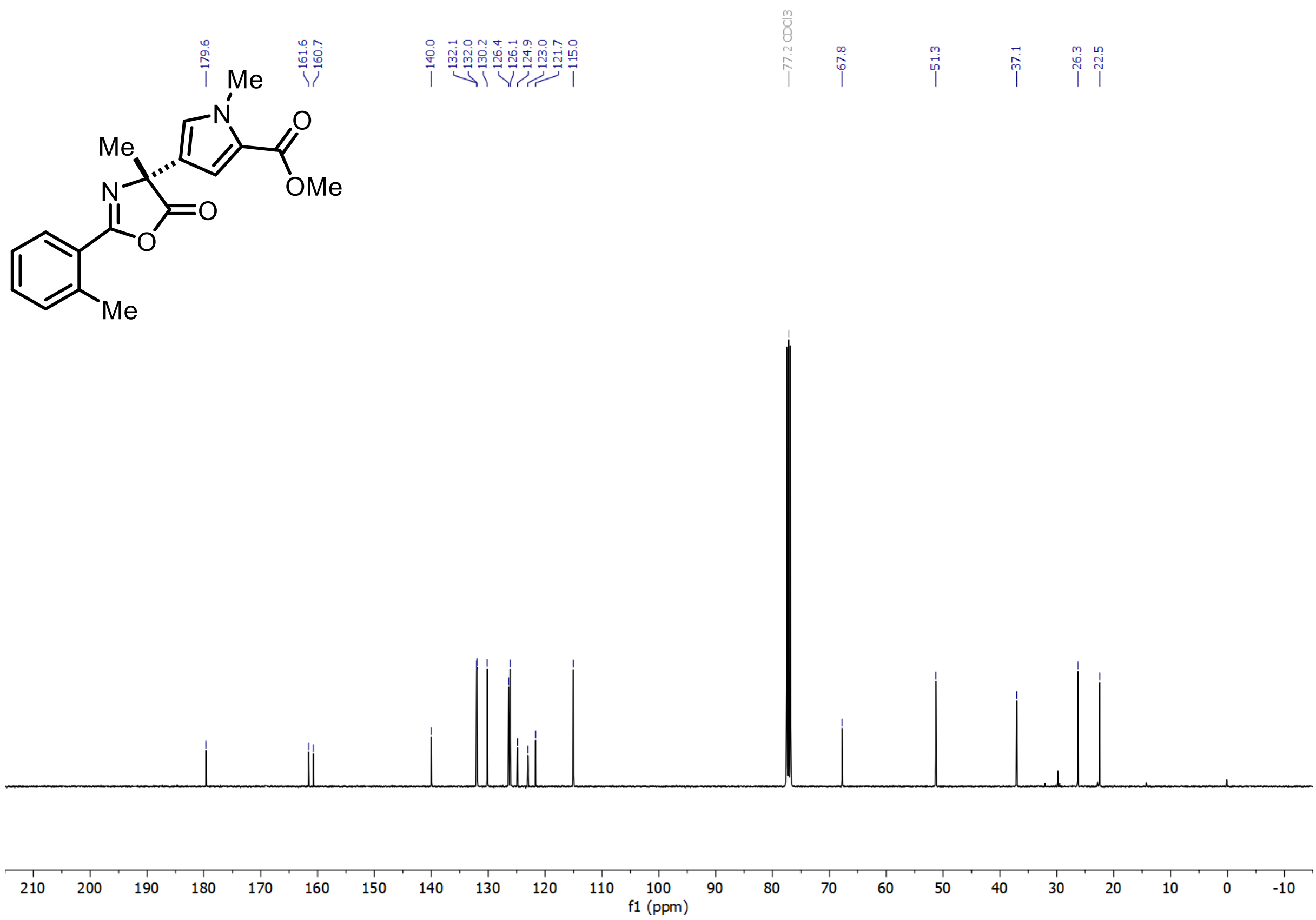

^{13}C NMR spectra of **4abβ**.

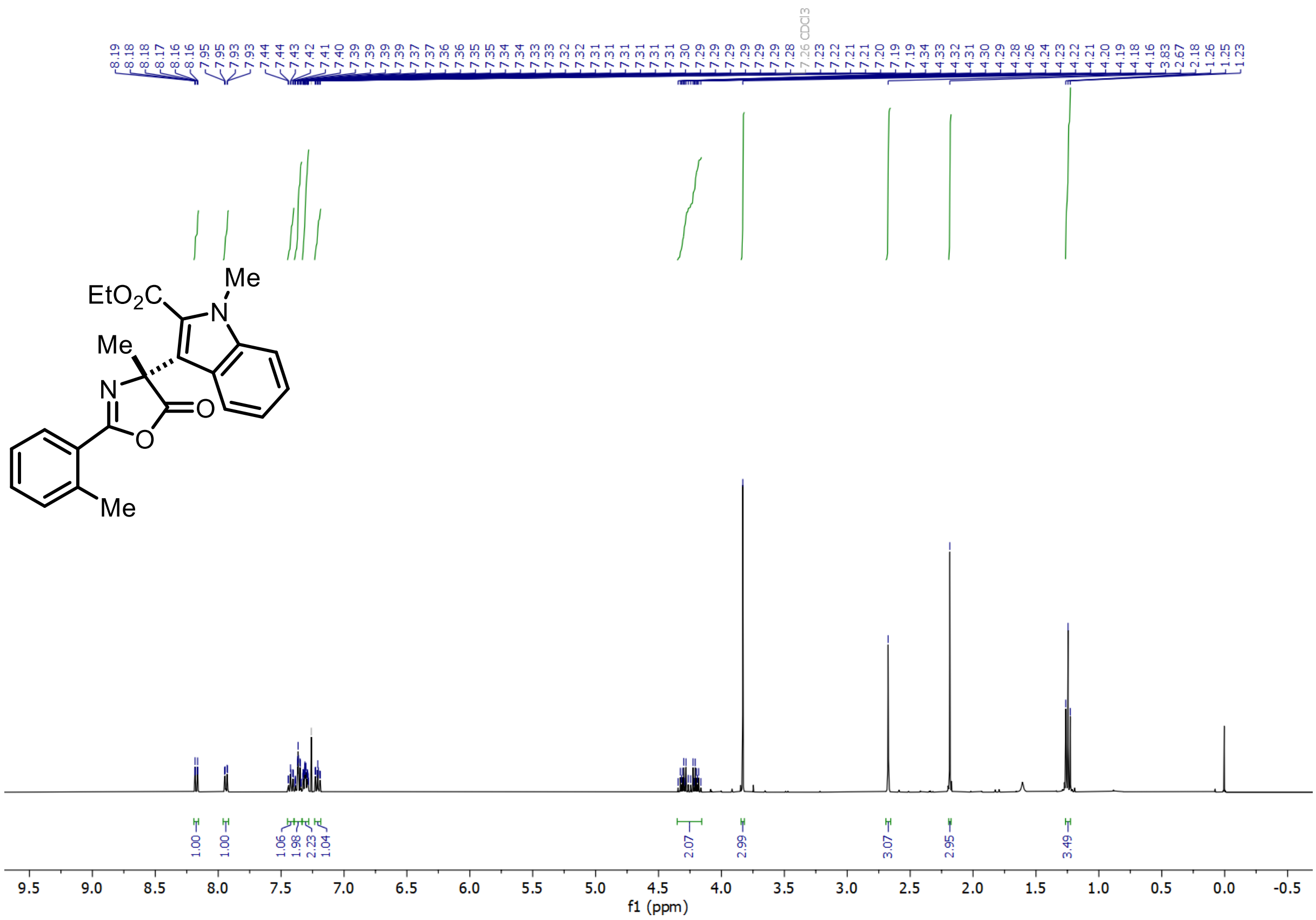

^{1}H NMR spectra of **4ac**.

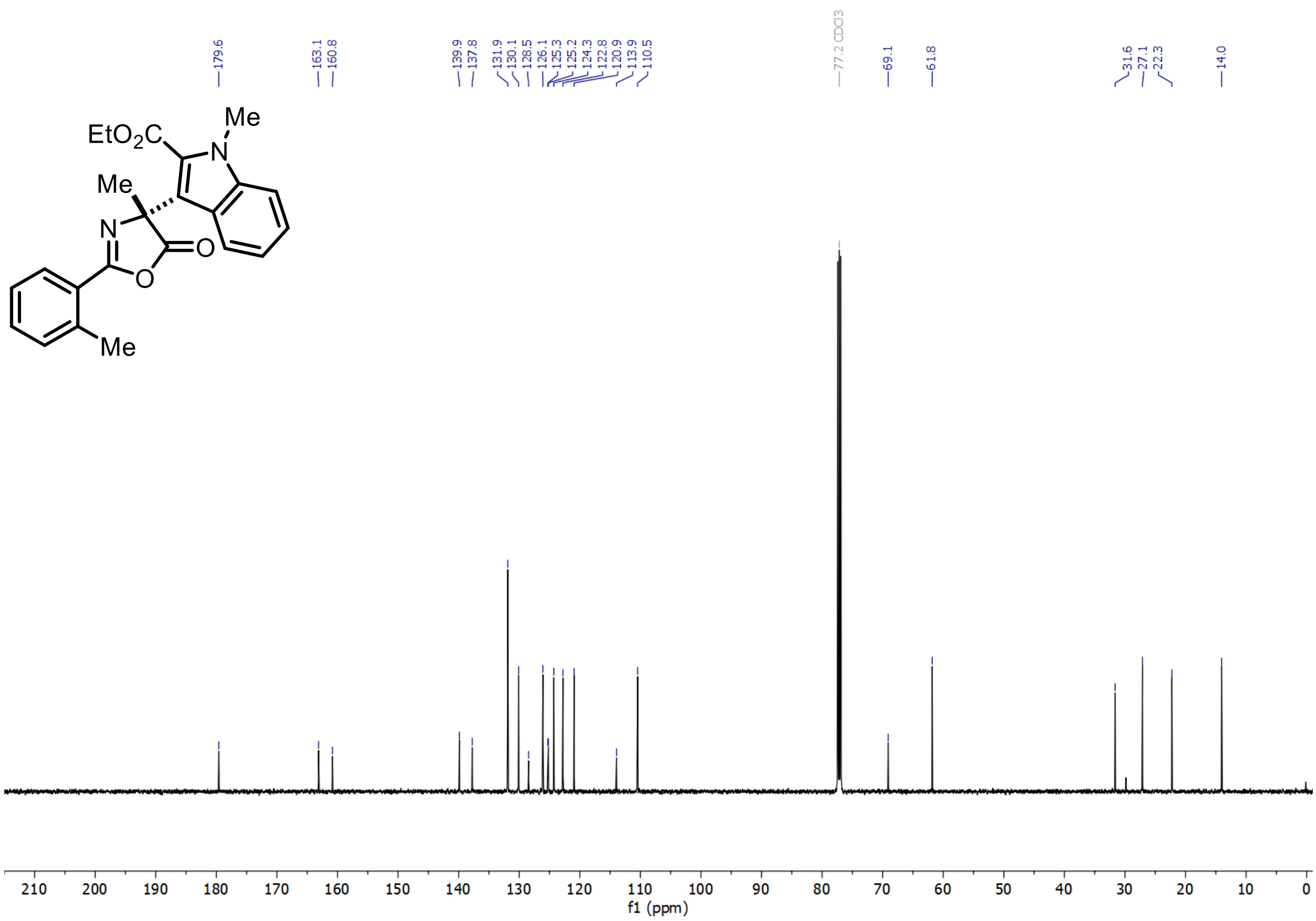

^{13}C NMR spectra of **4ac**.

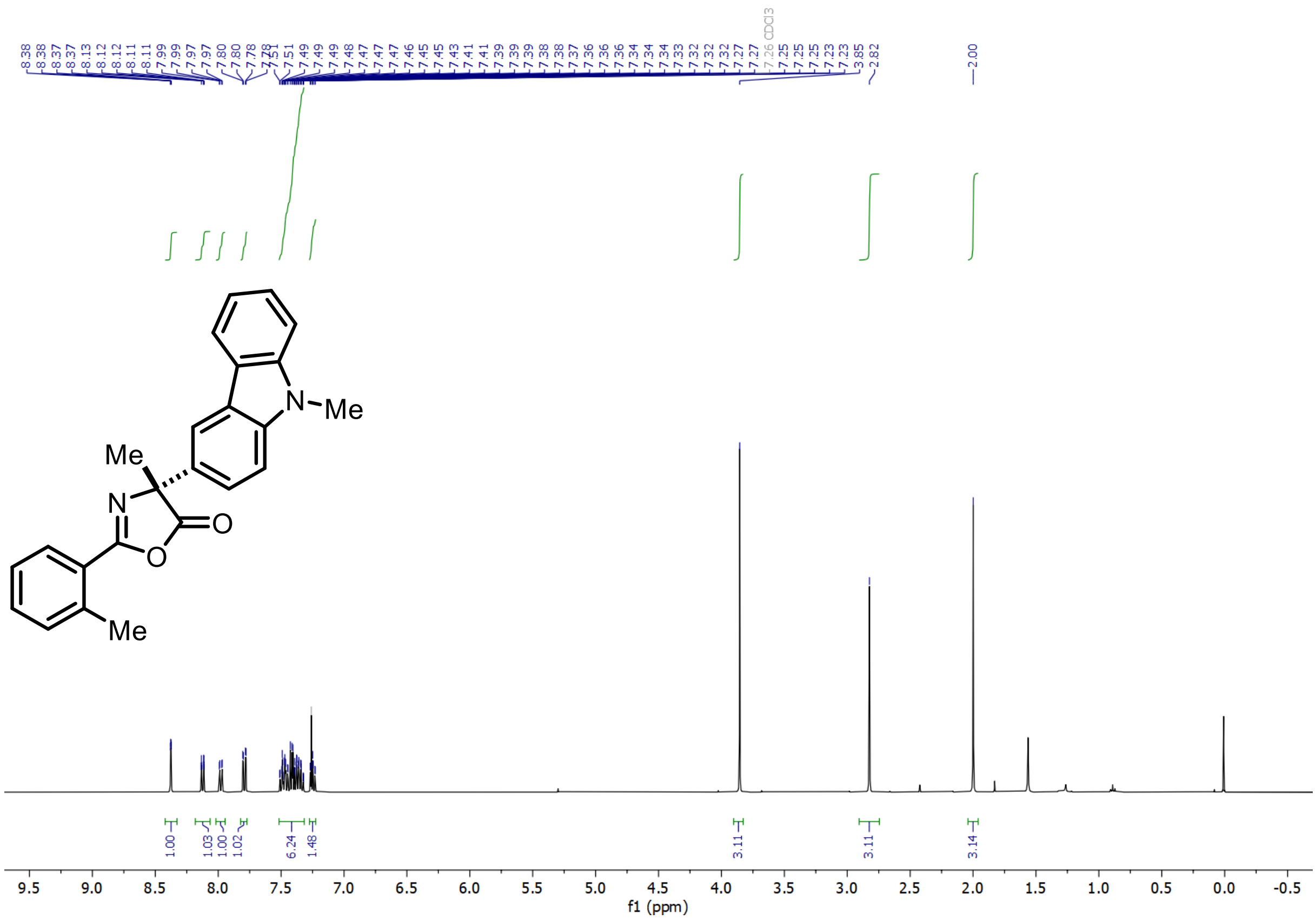

^{1}H NMR spectra of **4ad**.

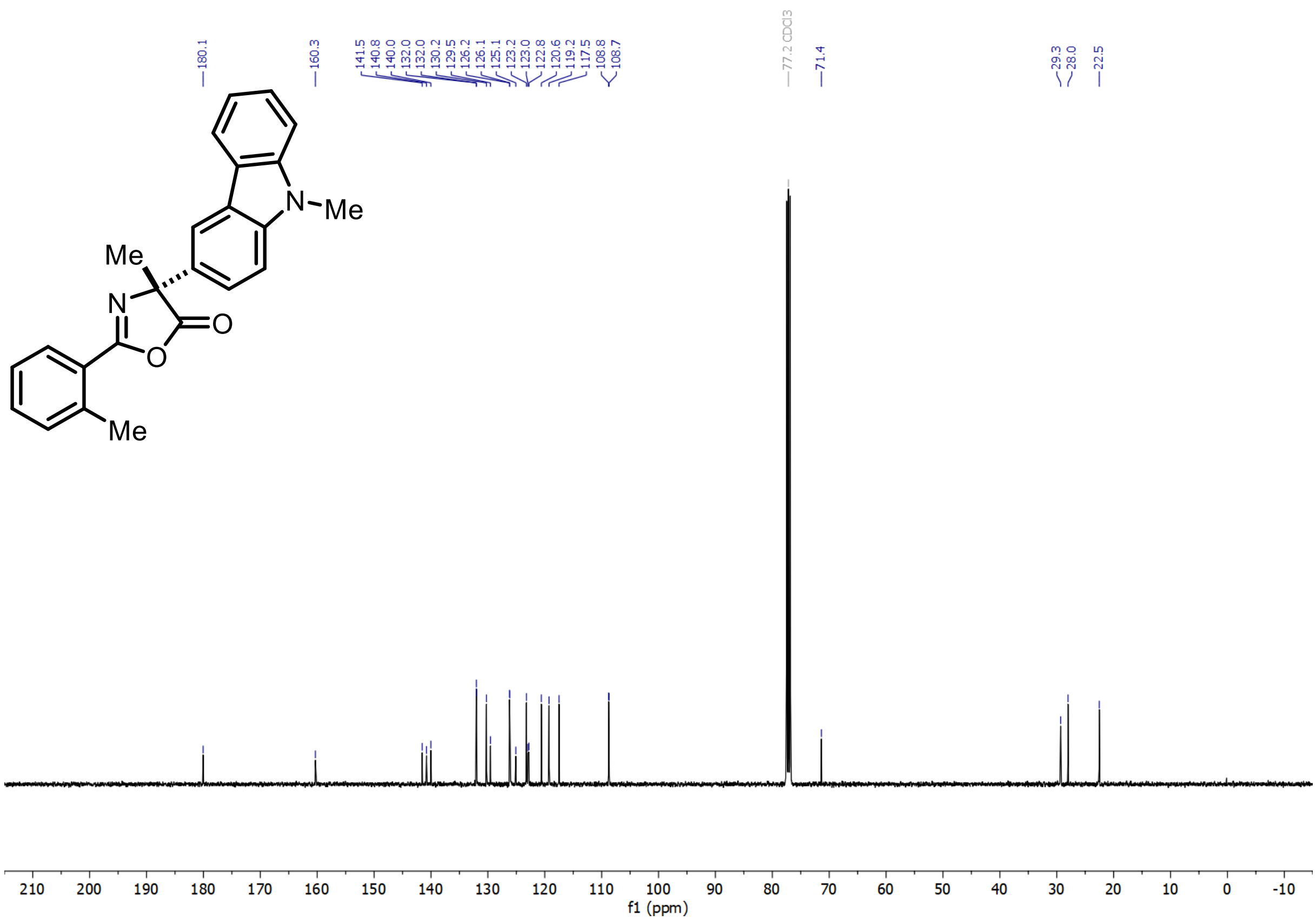

^{13}C NMR spectra of **4ad**.

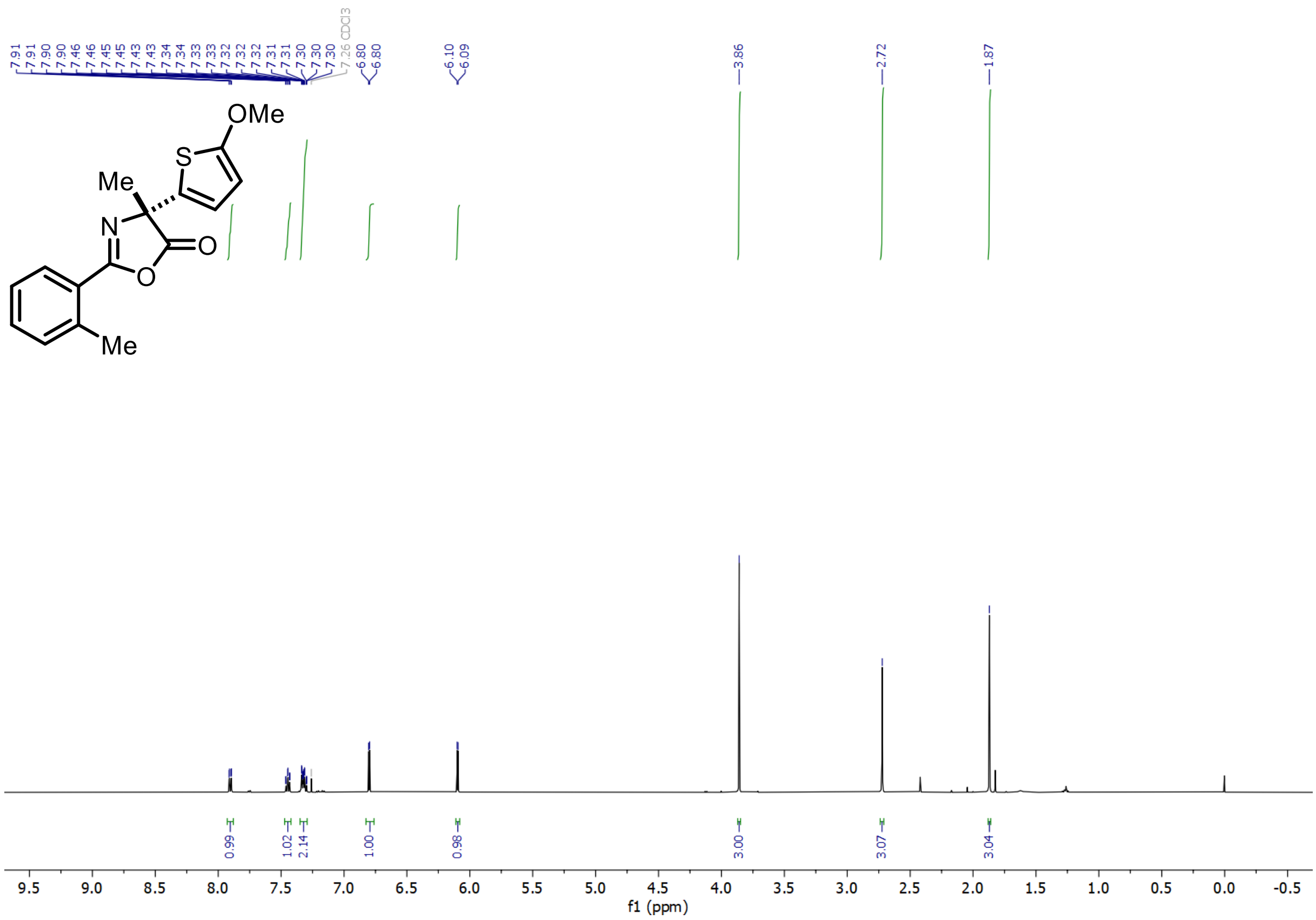

^{1}H NMR spectra of **4ae**.

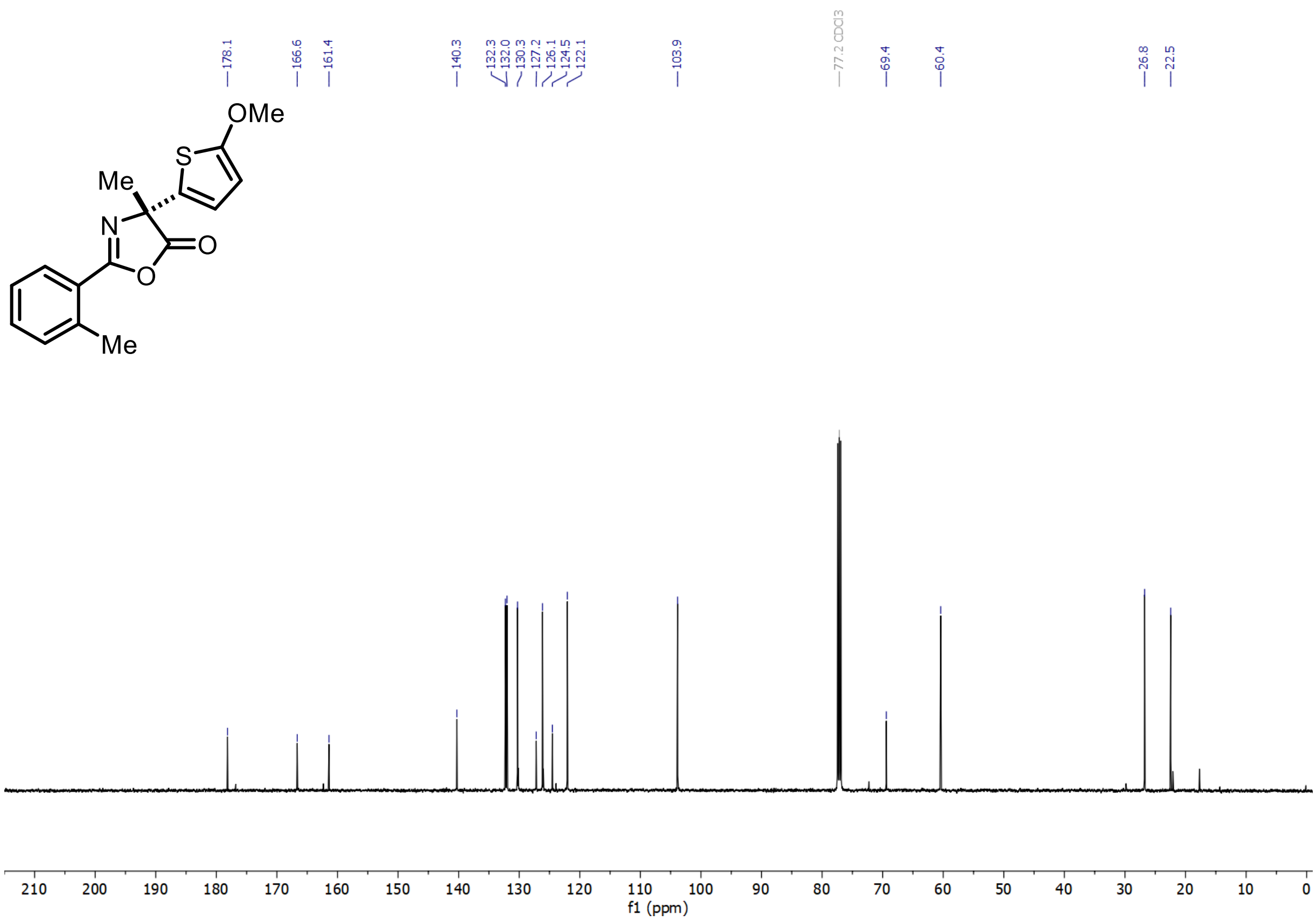

^{13}C NMR spectra of **4ae**.

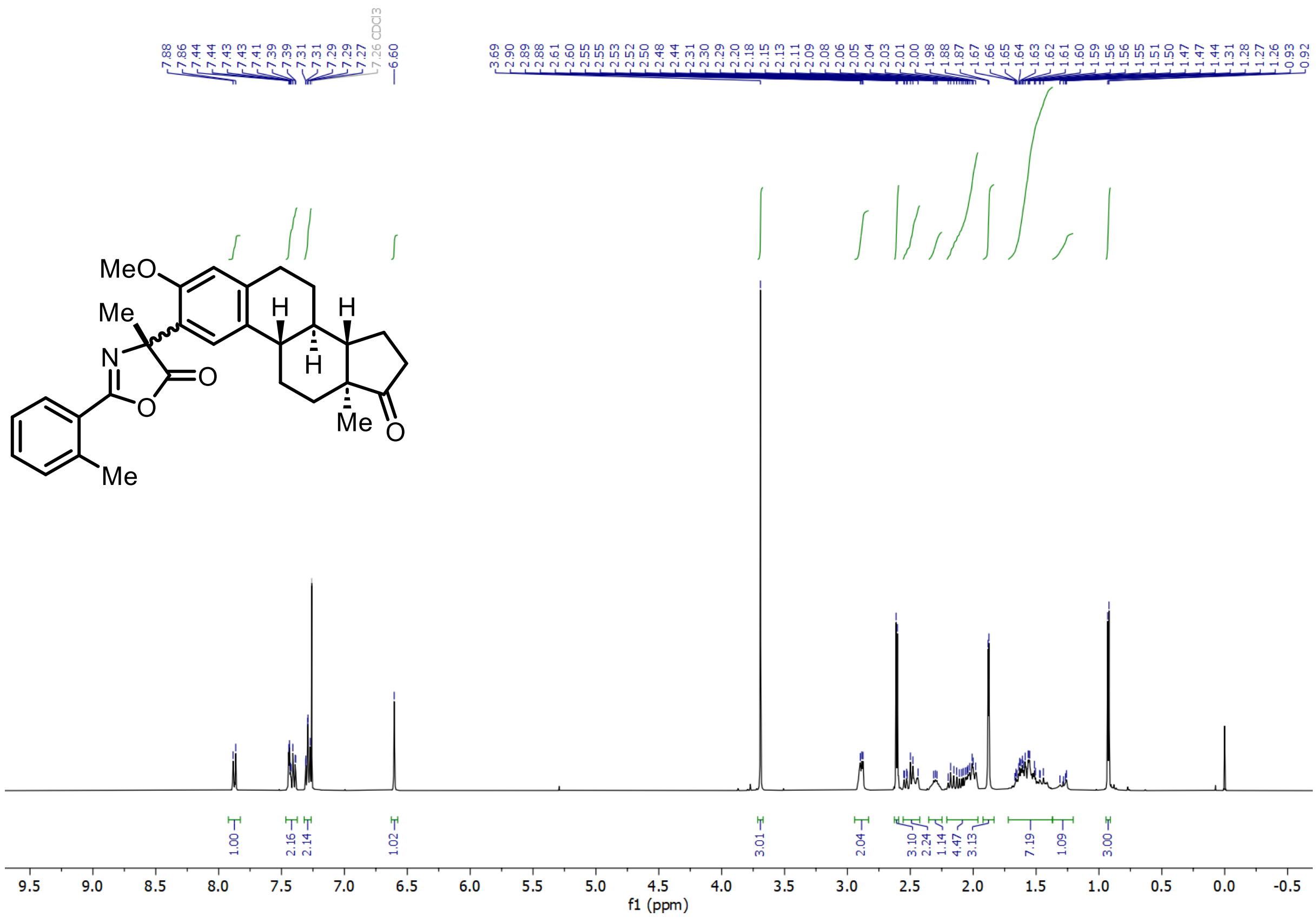

^{1}H NMR spectra of **4af**.

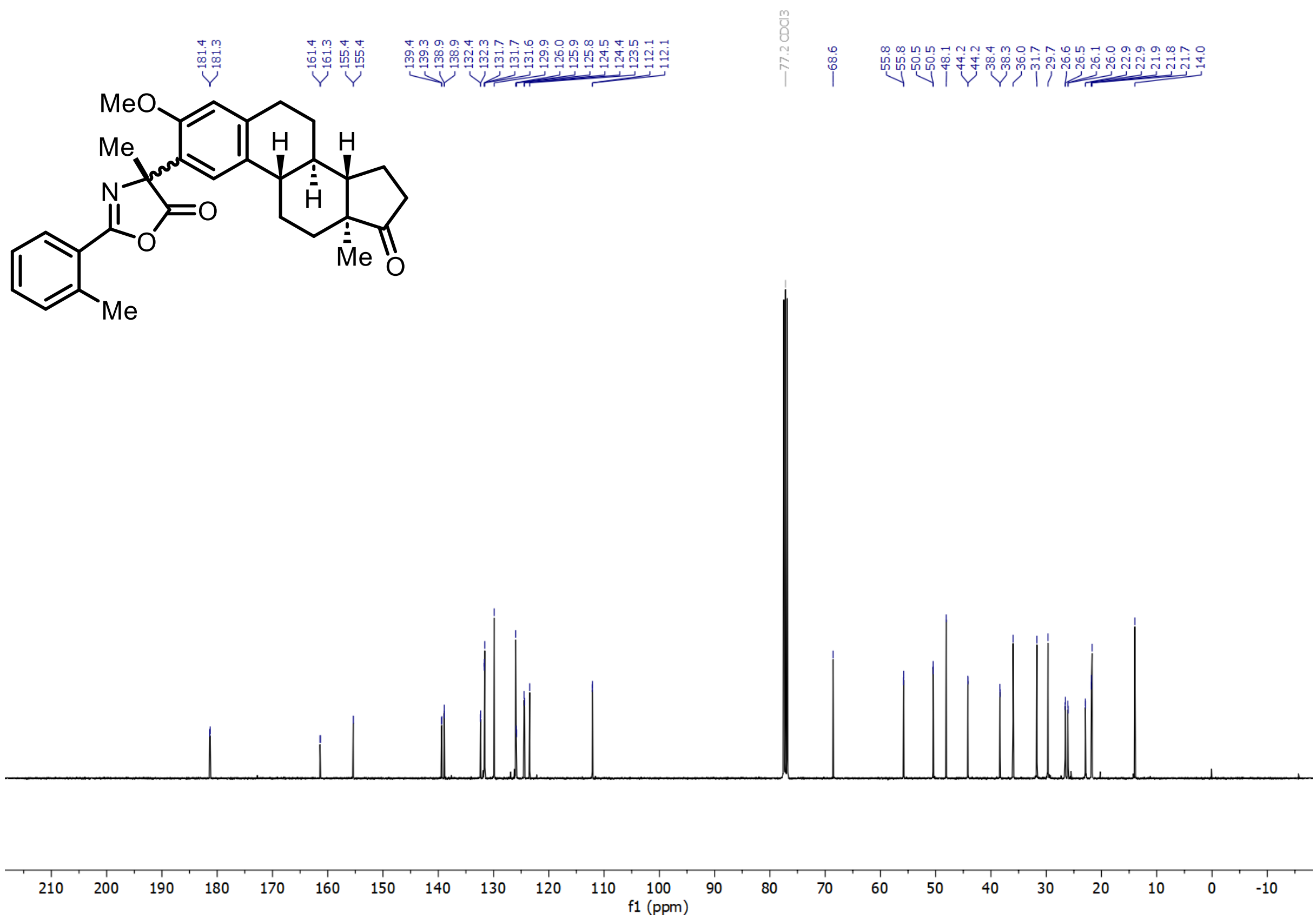

^{13}C NMR spectra of **4af**.

M.4. NMR spectra of products 10

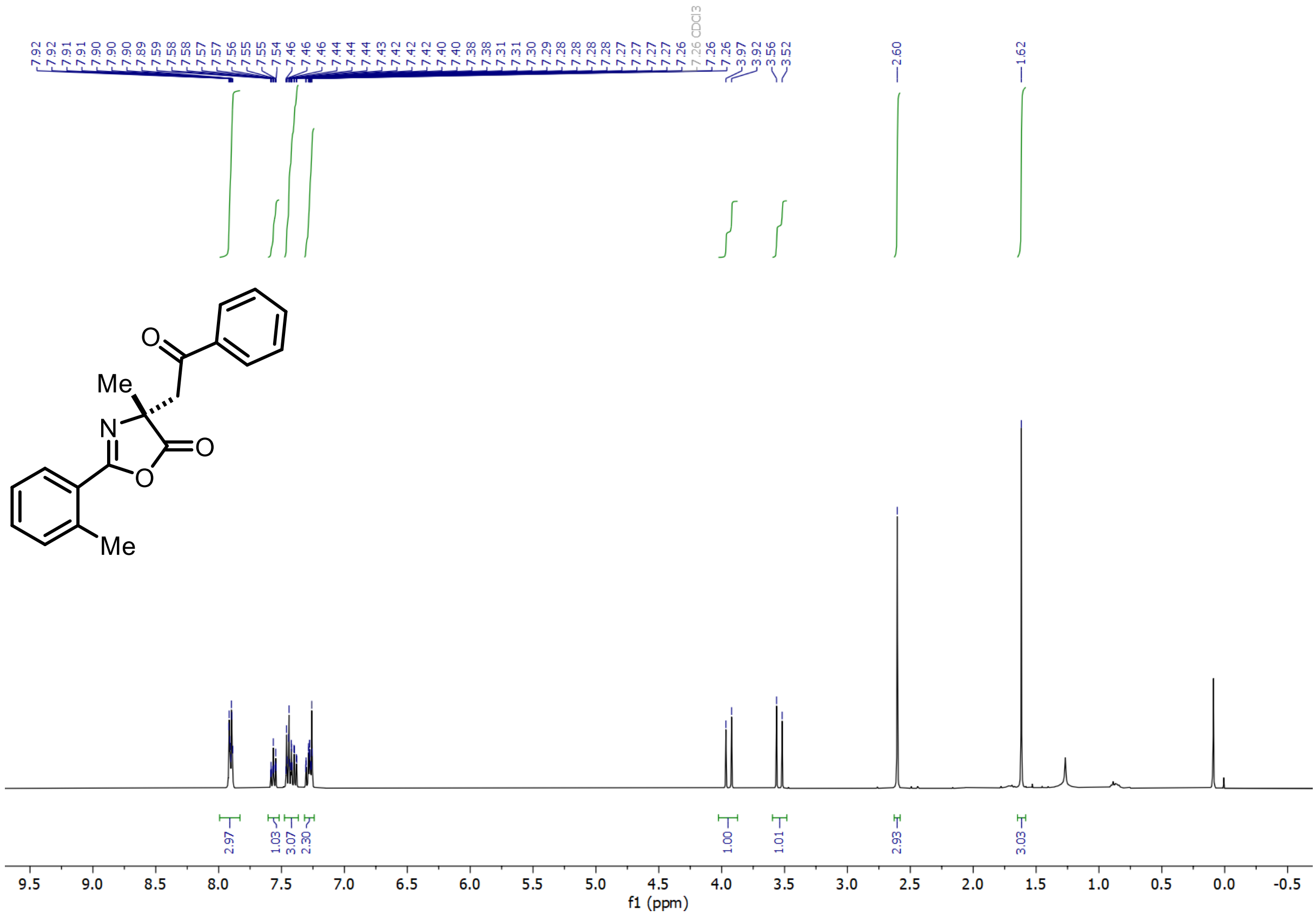

^{1}H NMR spectra of **10a**.

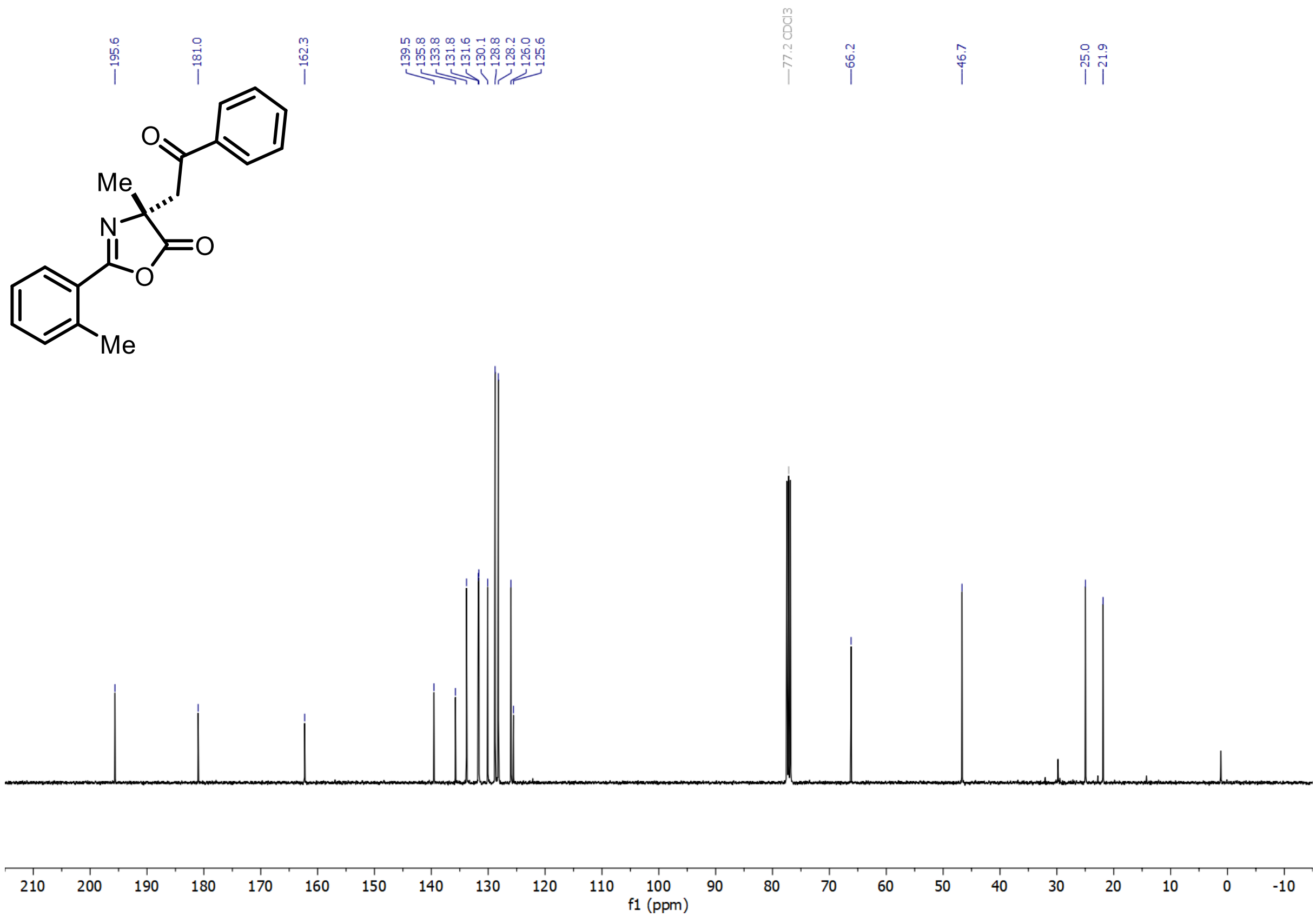

^{13}C NMR spectra of **10a**.

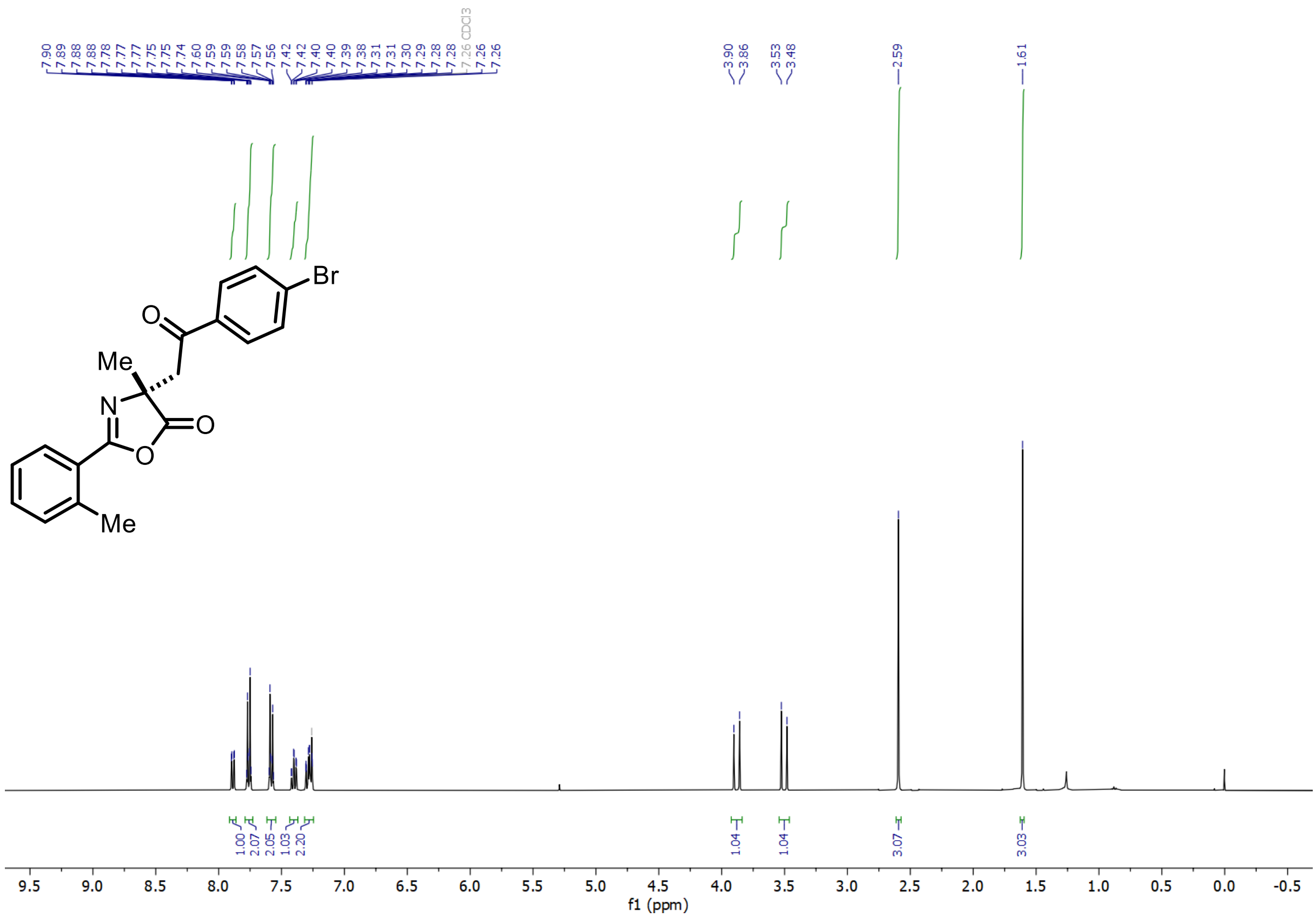

^{1}H NMR spectra of **10b**.

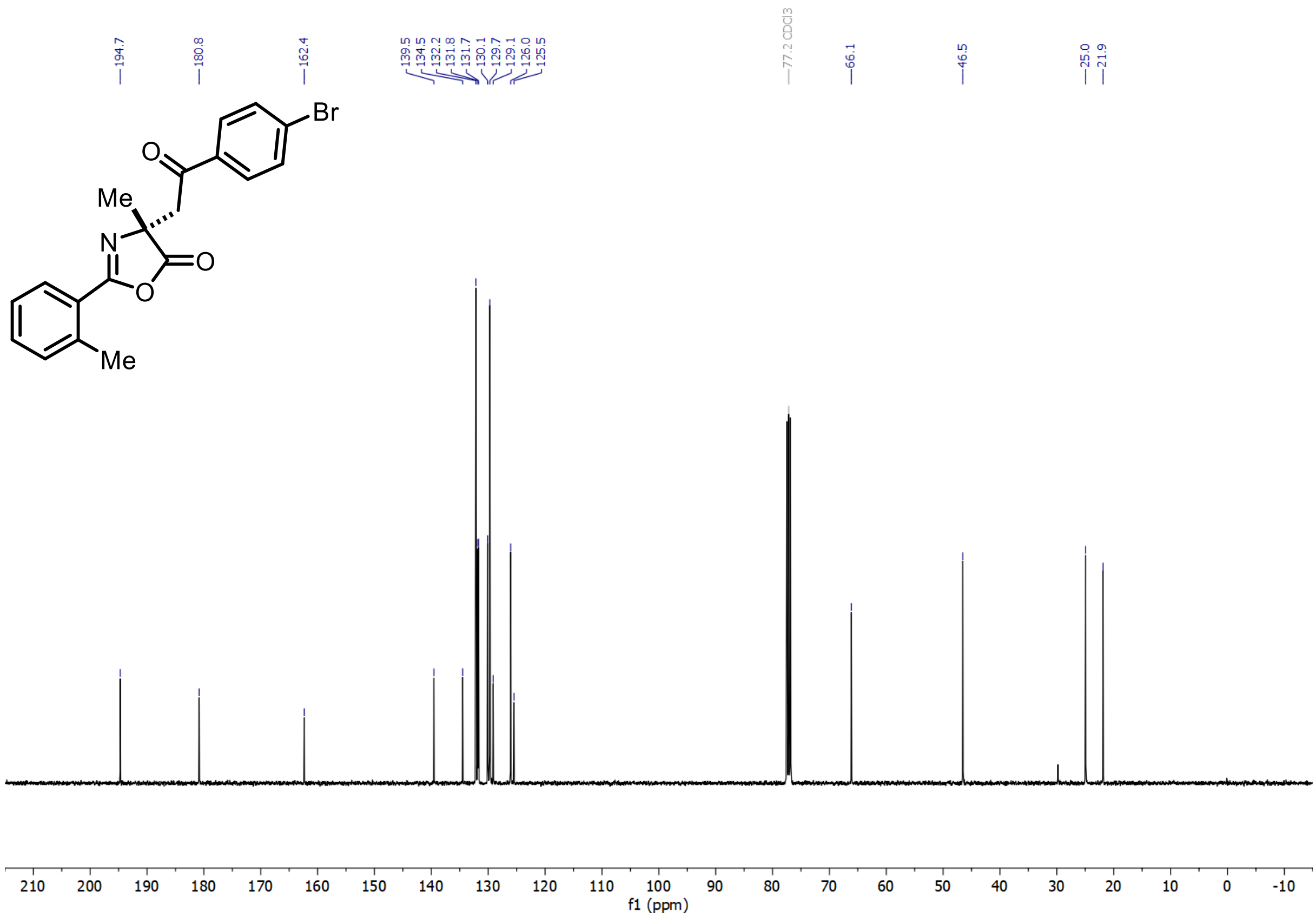

^{13}C NMR spectra of **10b**.

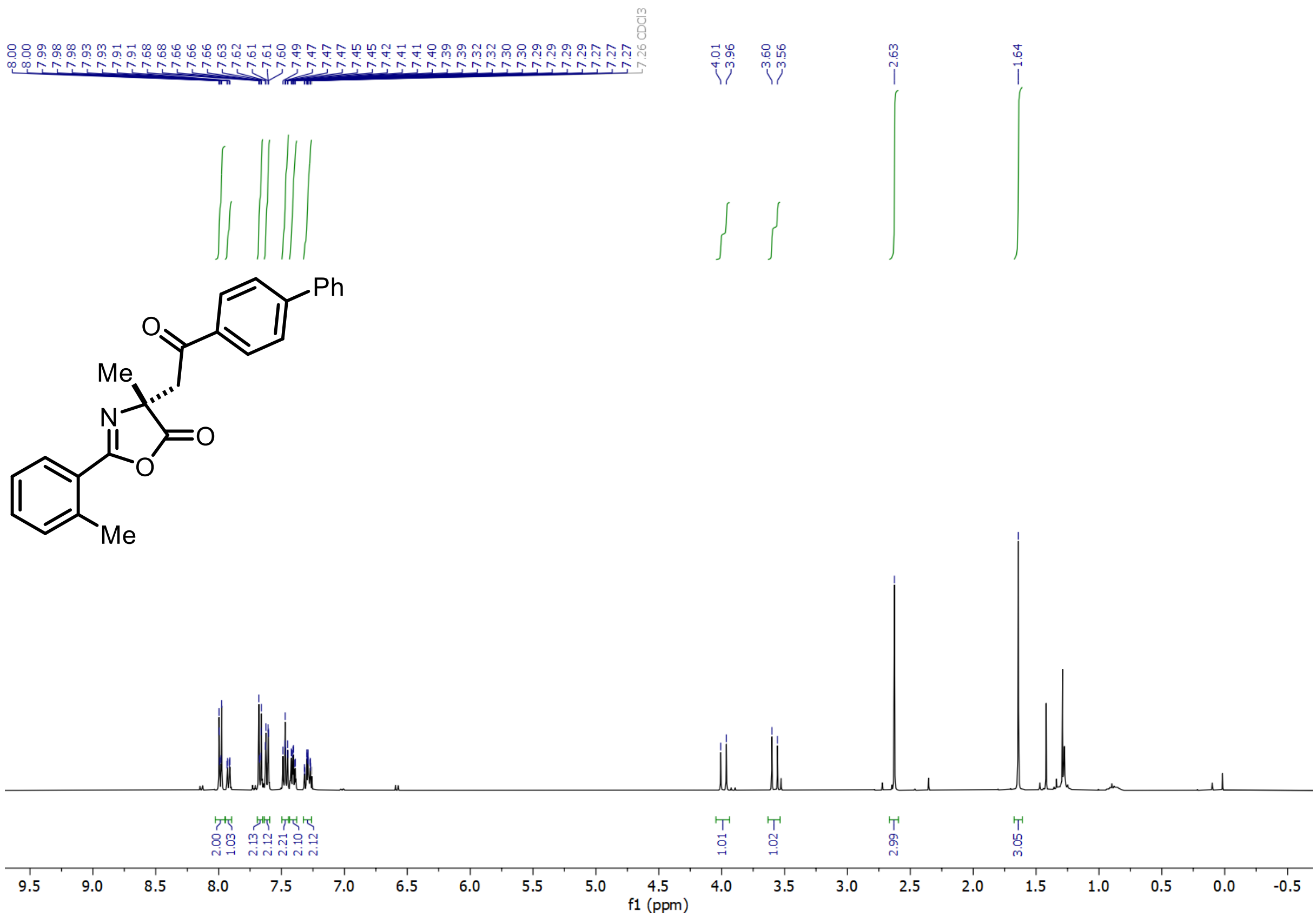

^{1}H NMR spectra of **10c**.

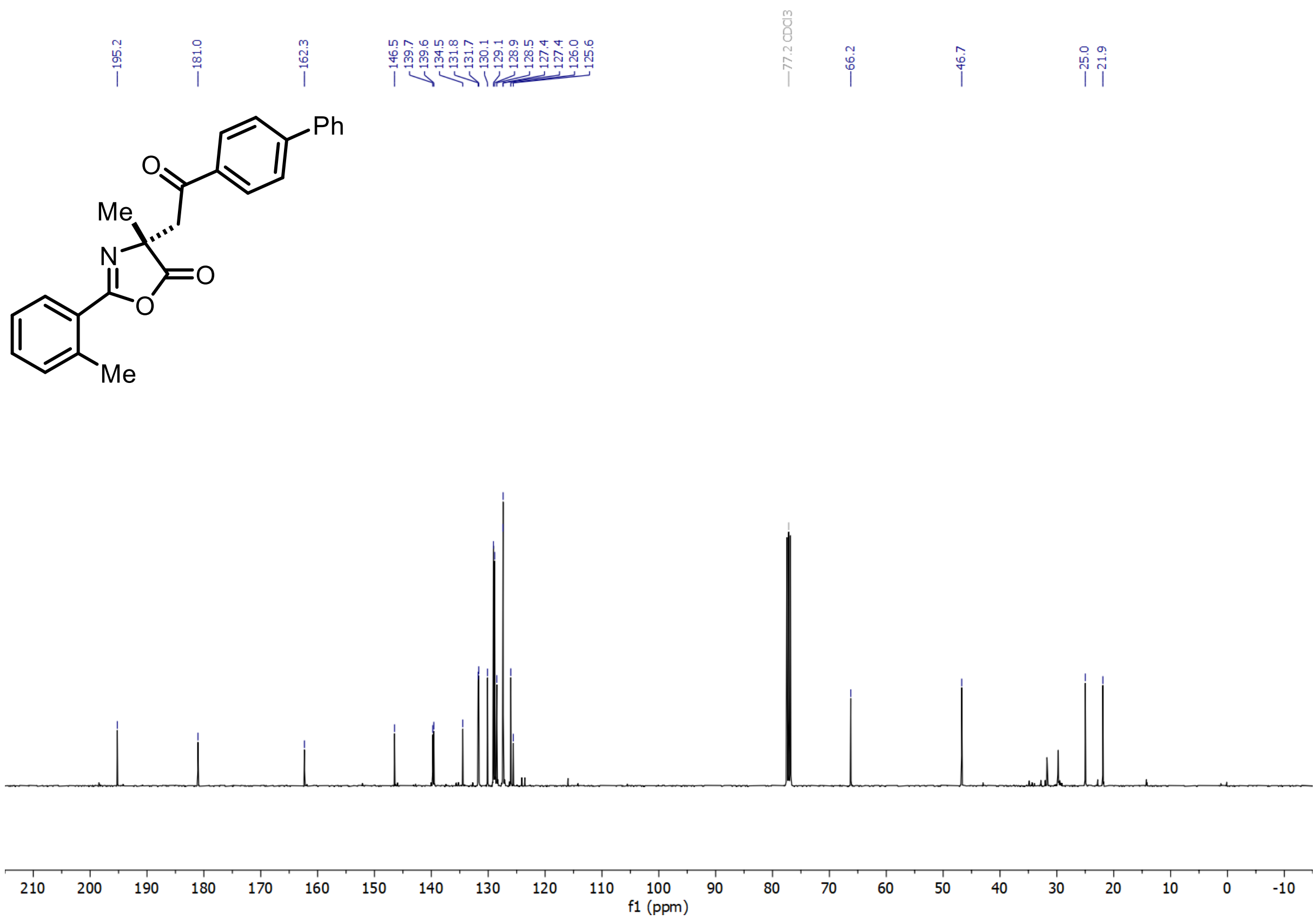

^{13}C NMR spectra of **10c**.

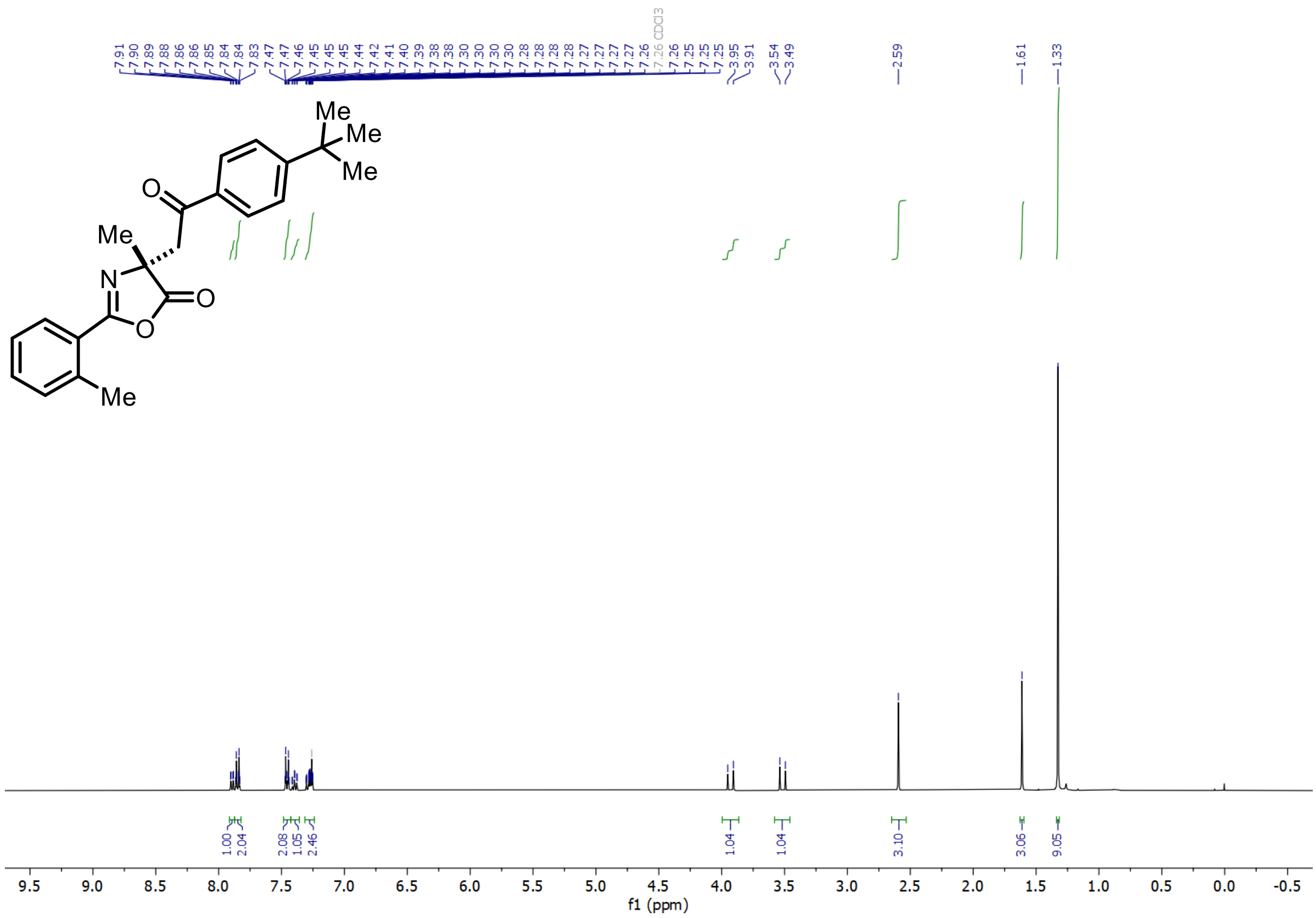

^{1}H NMR spectra of **10d**.

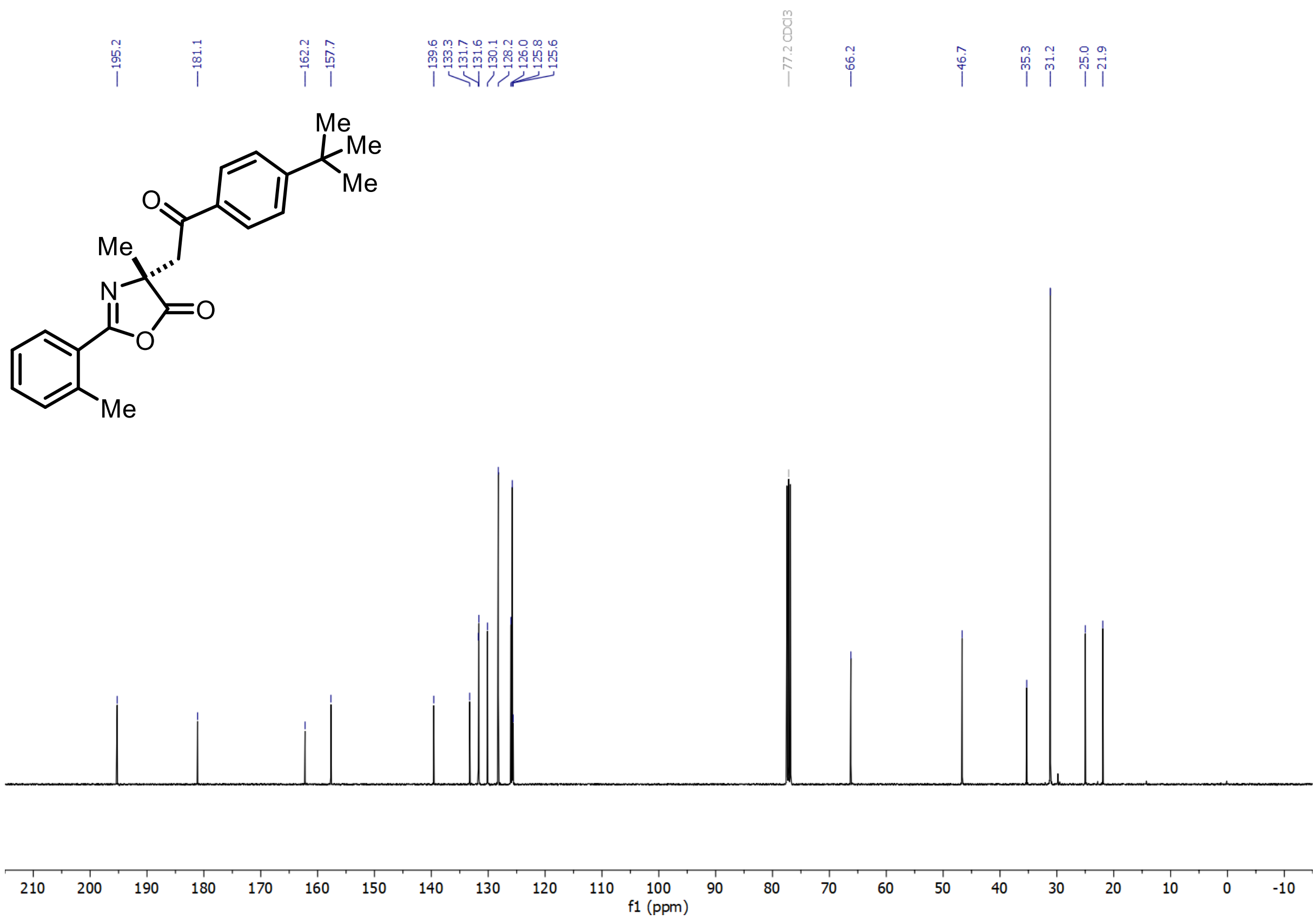

^{13}C NMR spectra of **10d**.

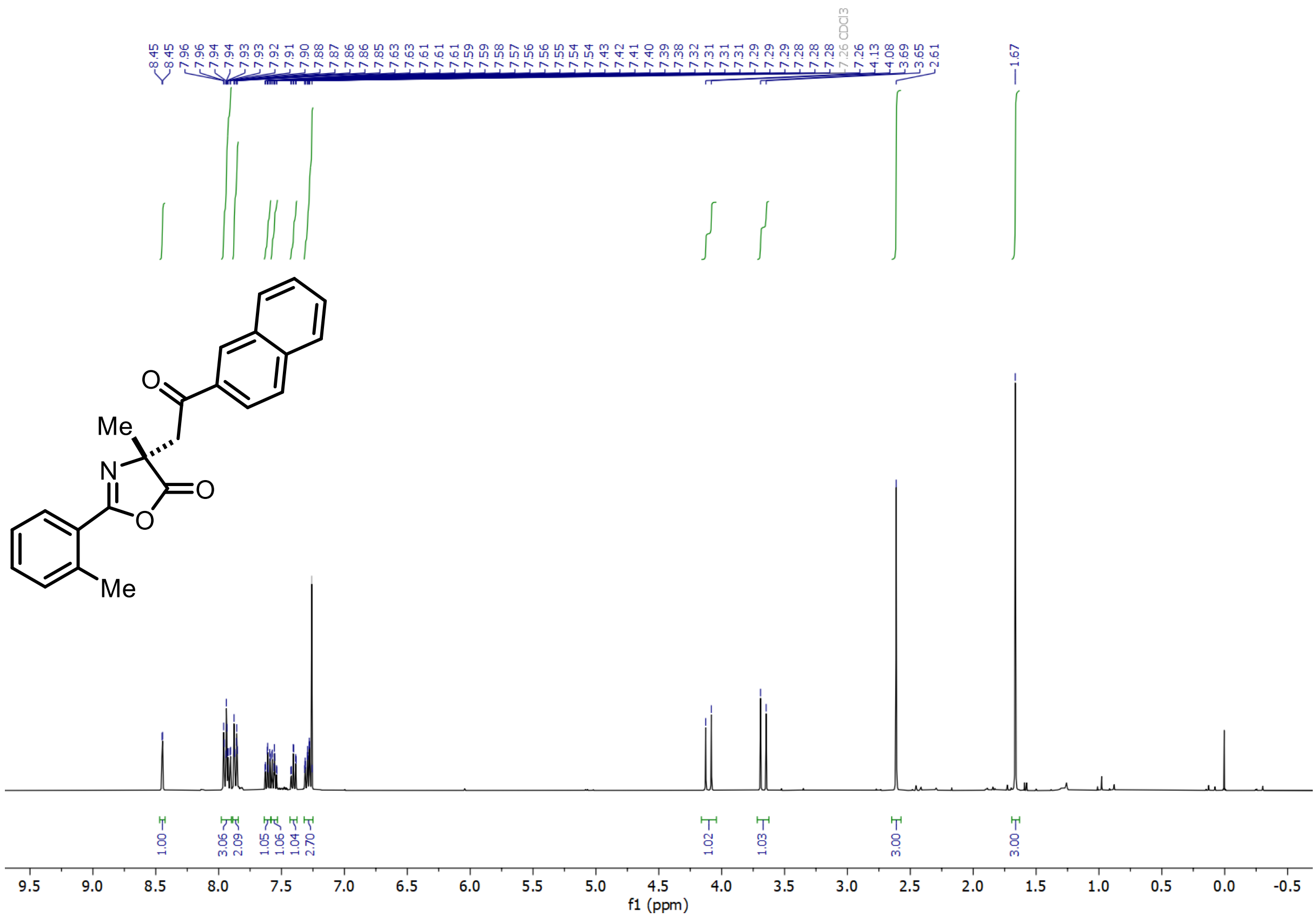

^{1}H NMR spectra of **10e**.

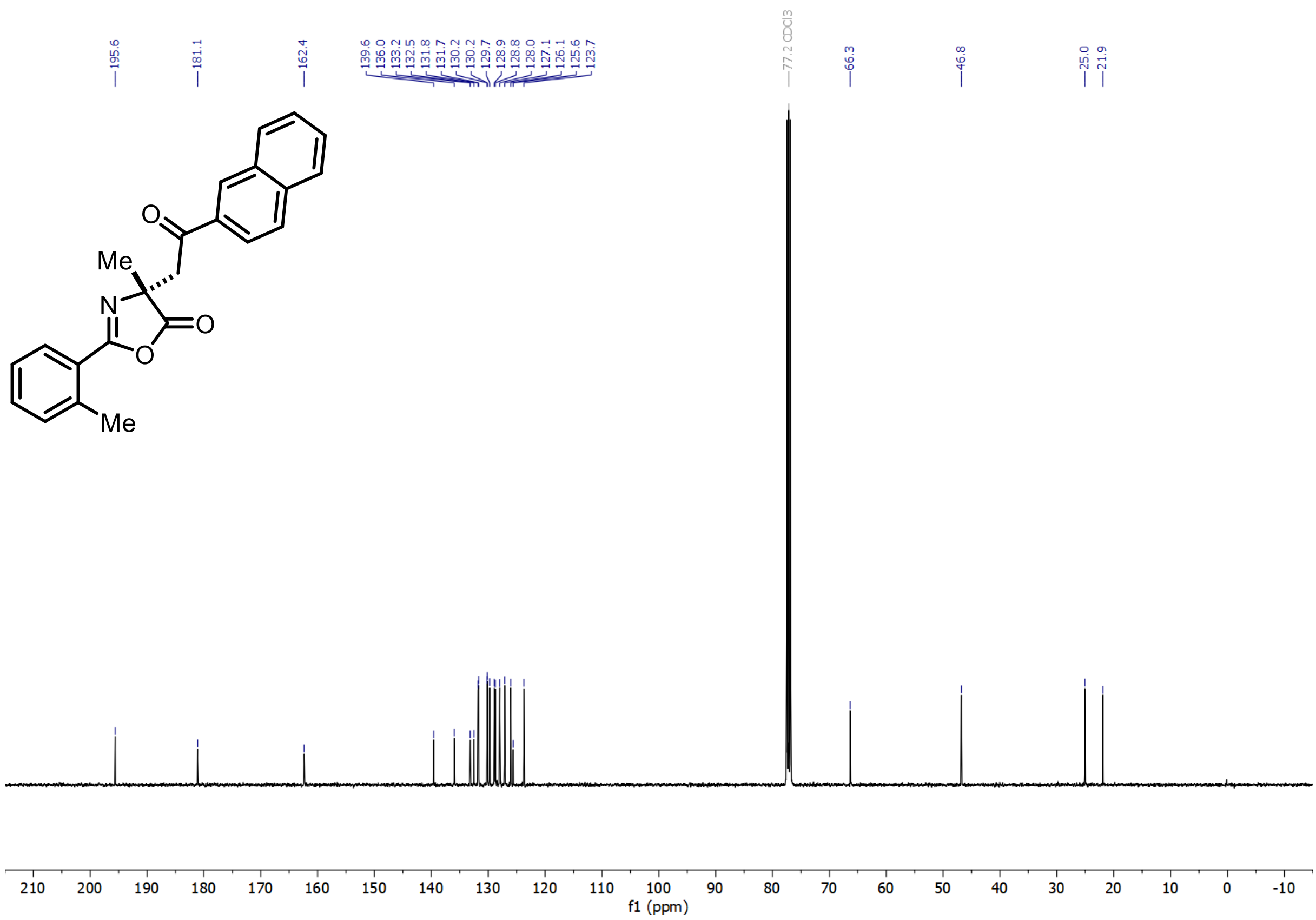

^{13}C NMR spectra of **10e**.

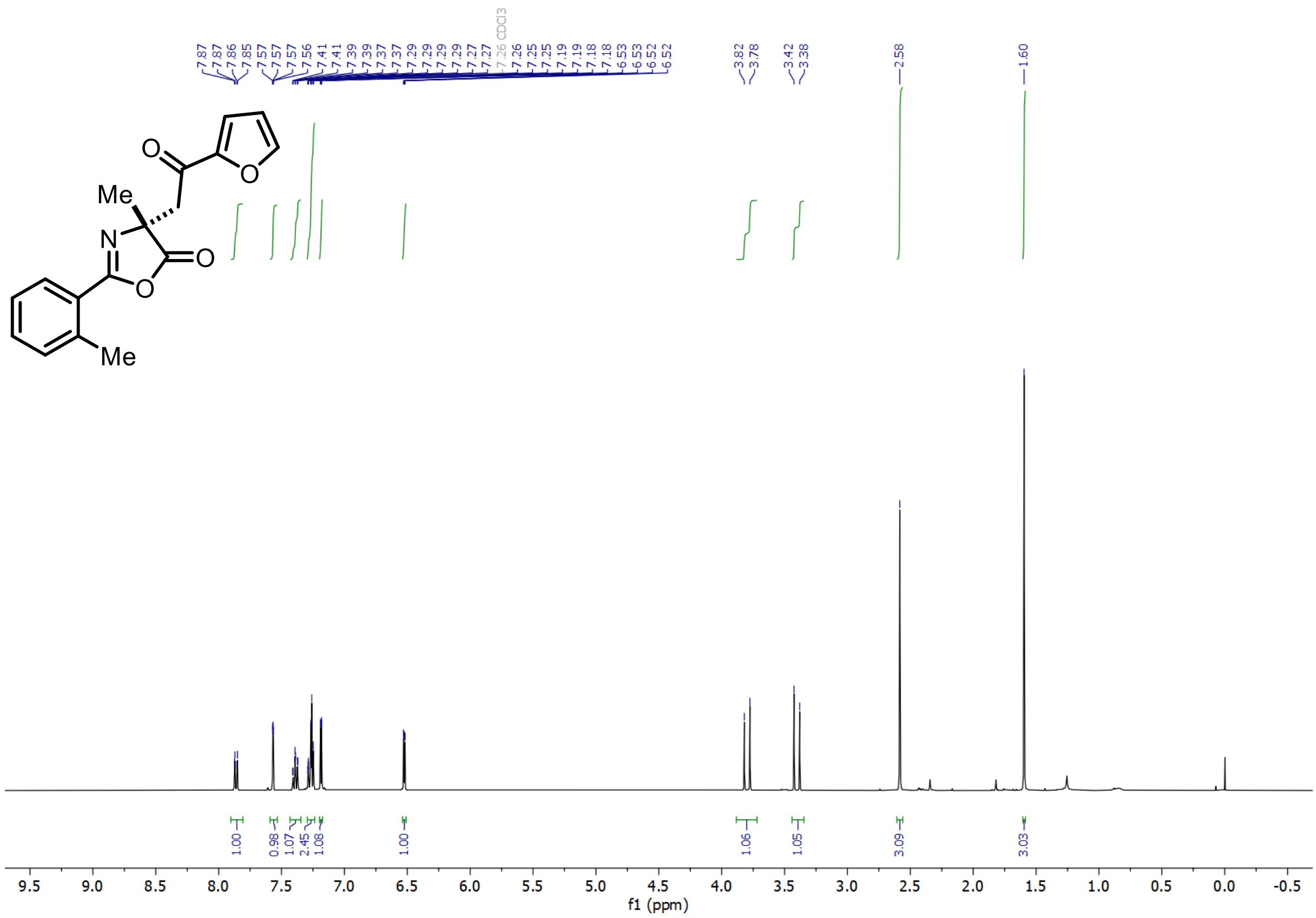

^{1}H NMR spectra of **10f**.

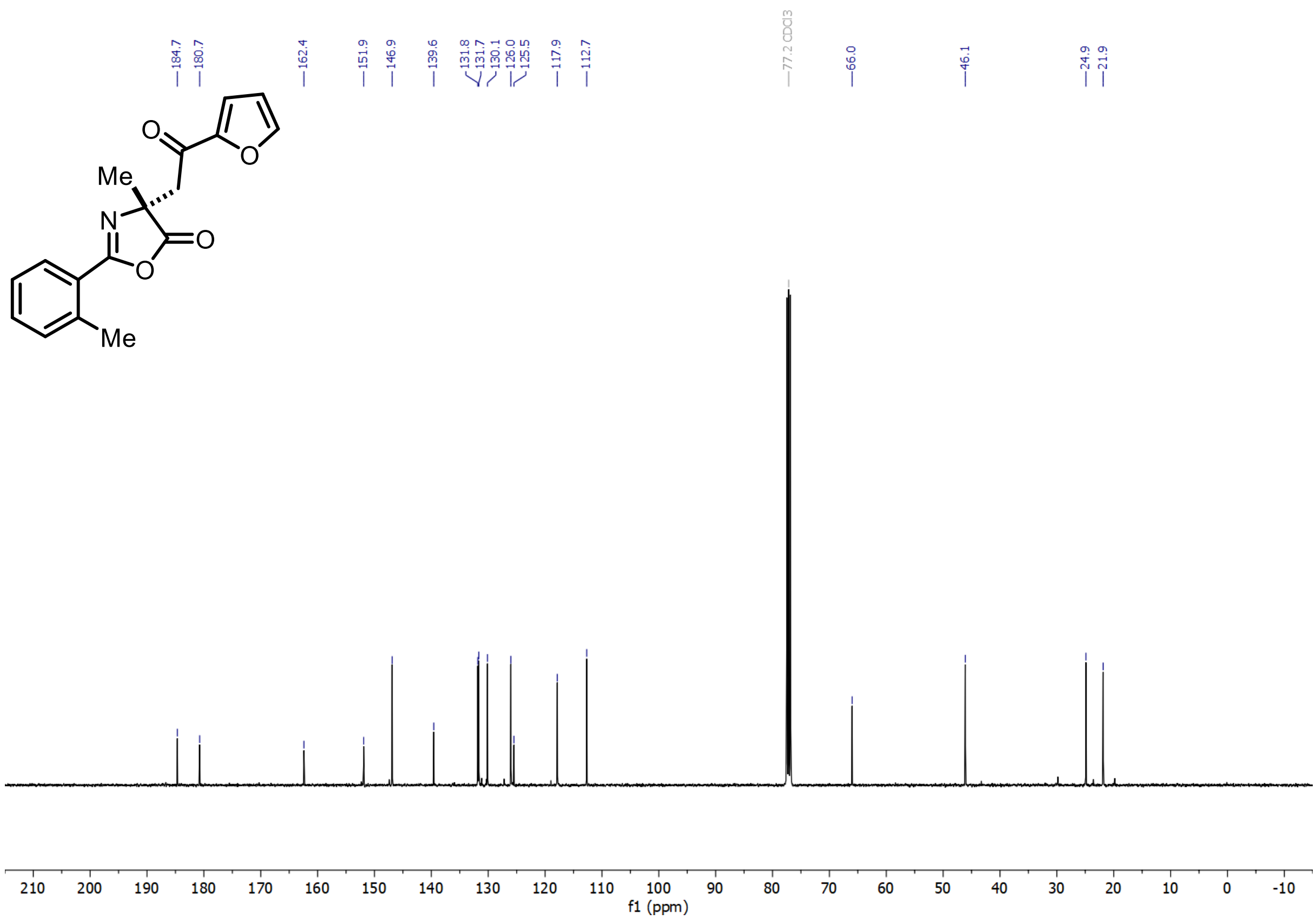

^{13}C NMR spectra of **10f**.

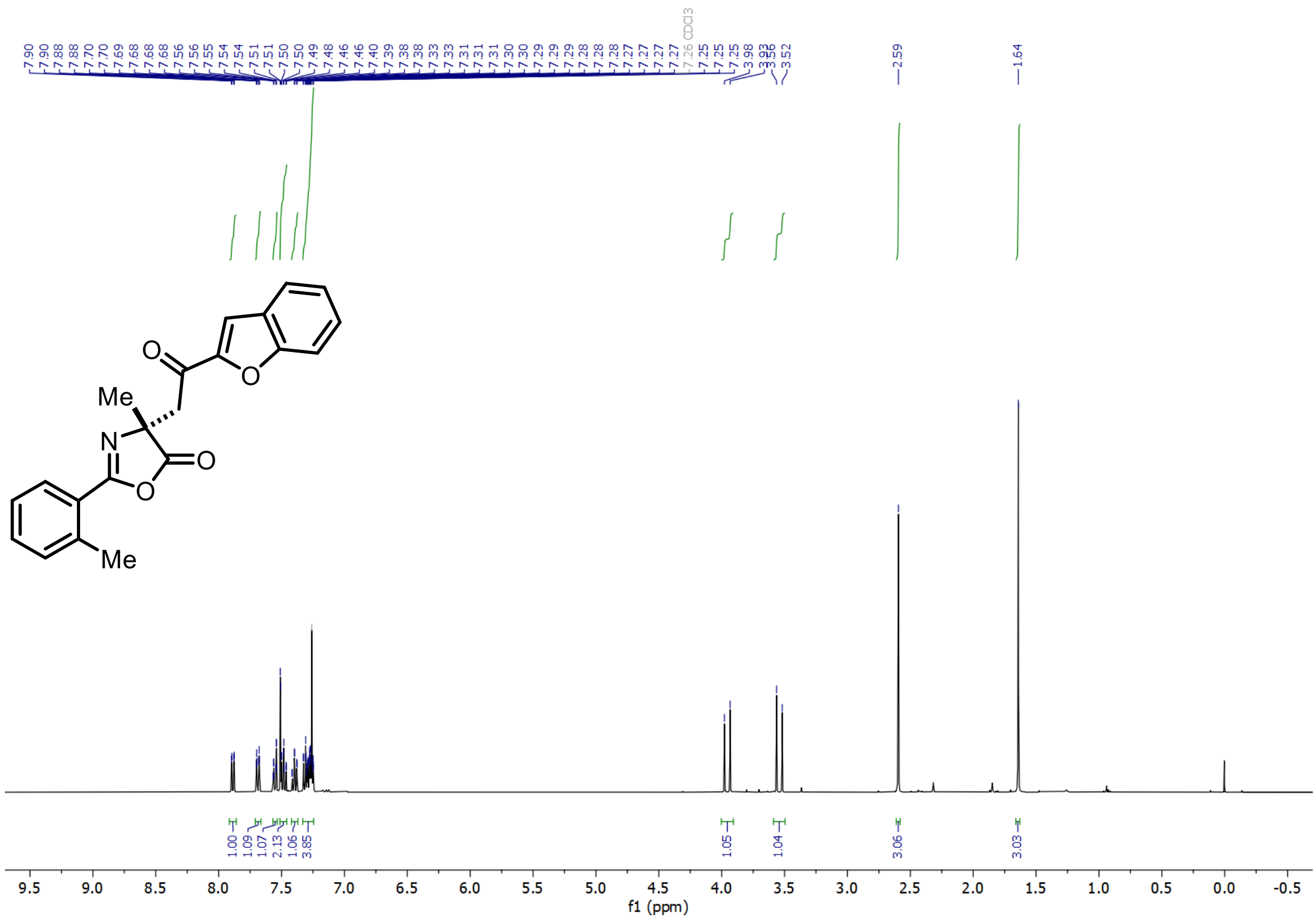

^{1}H NMR spectra of **10g**.

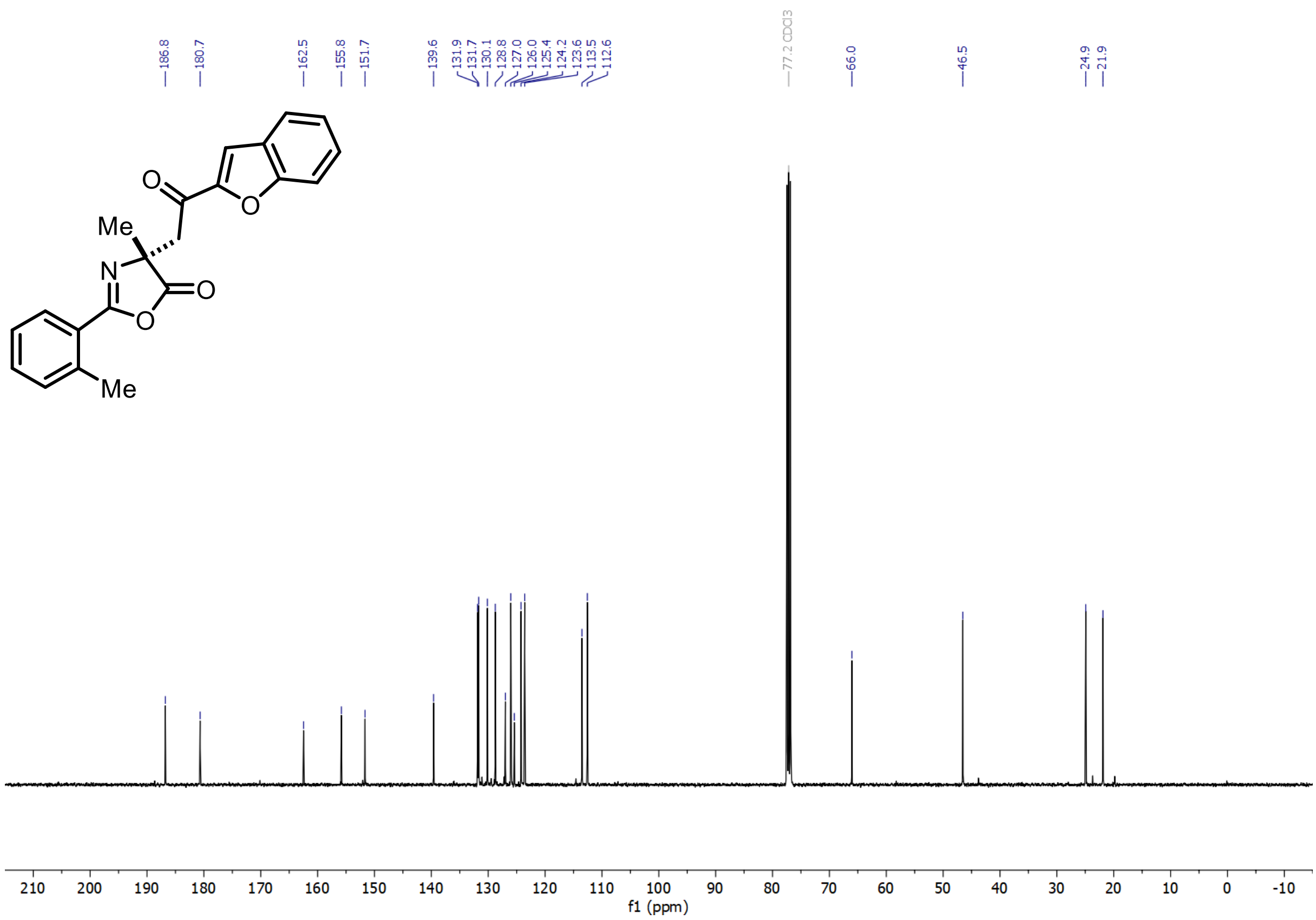

^{13}C NMR spectra of **10g.**

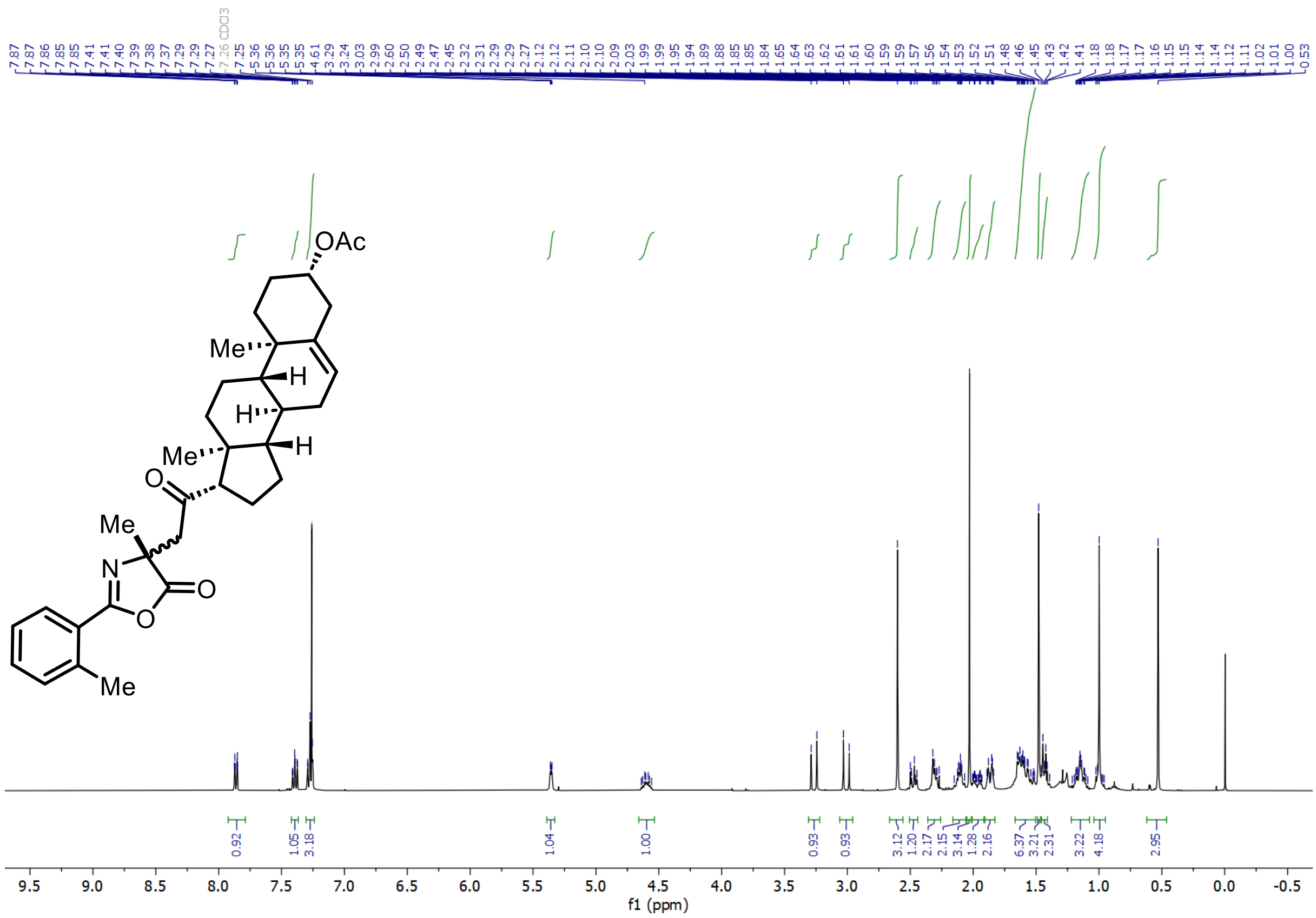

^{1}H NMR spectra of **10h*a***.

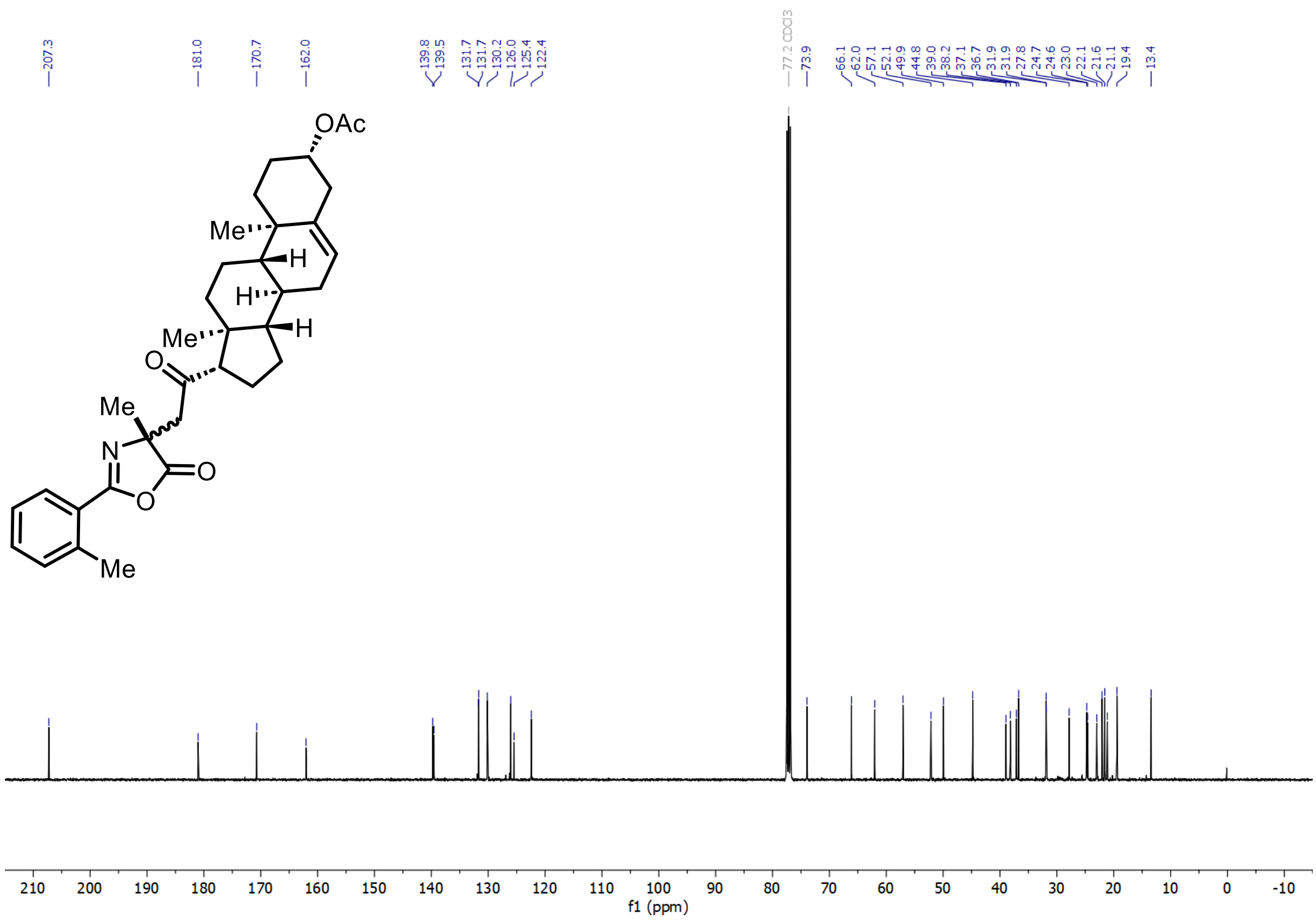

^{13}C NMR spectra of **10h*a*.**

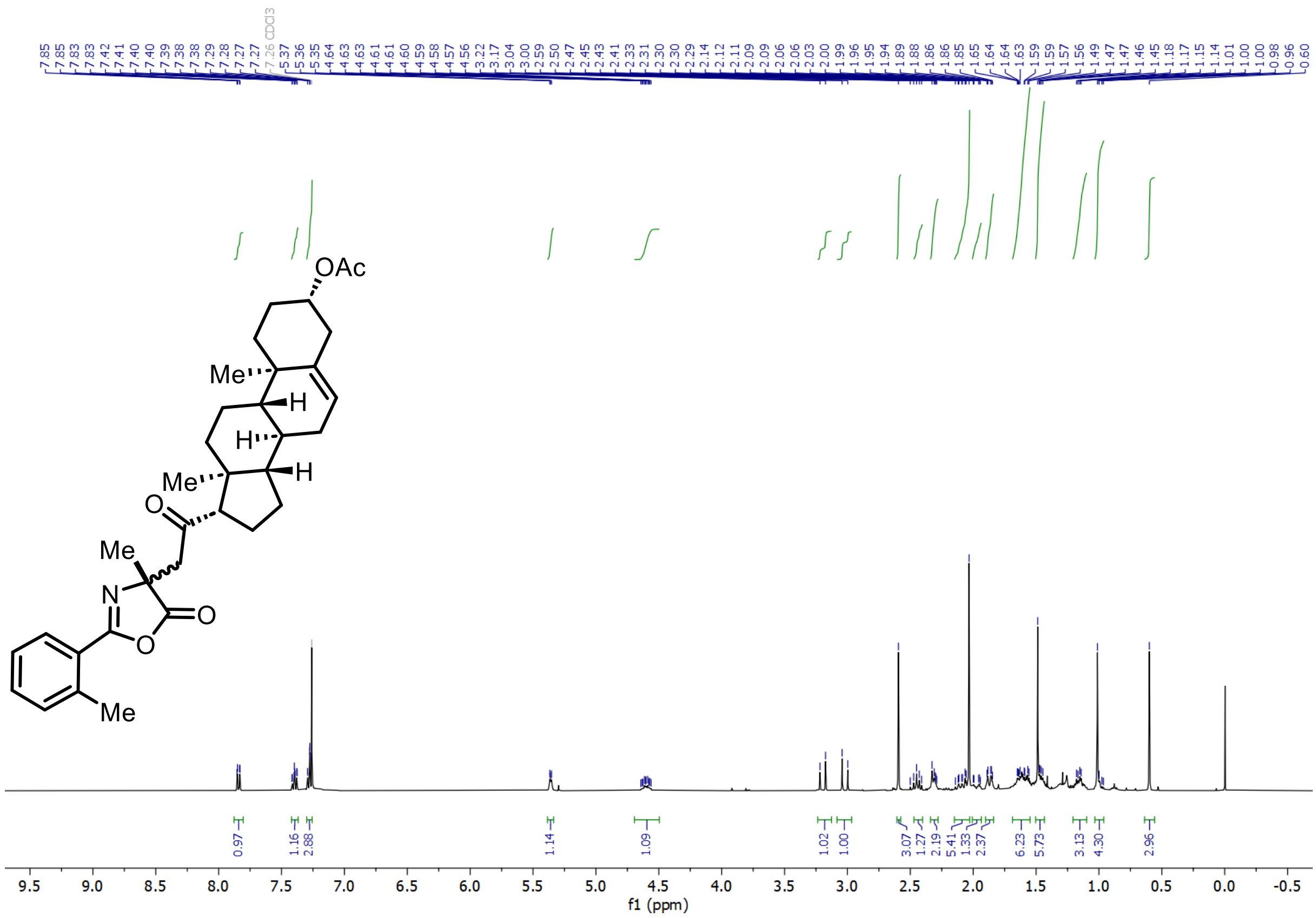

^{1}H NMR spectra of **10h*b***.

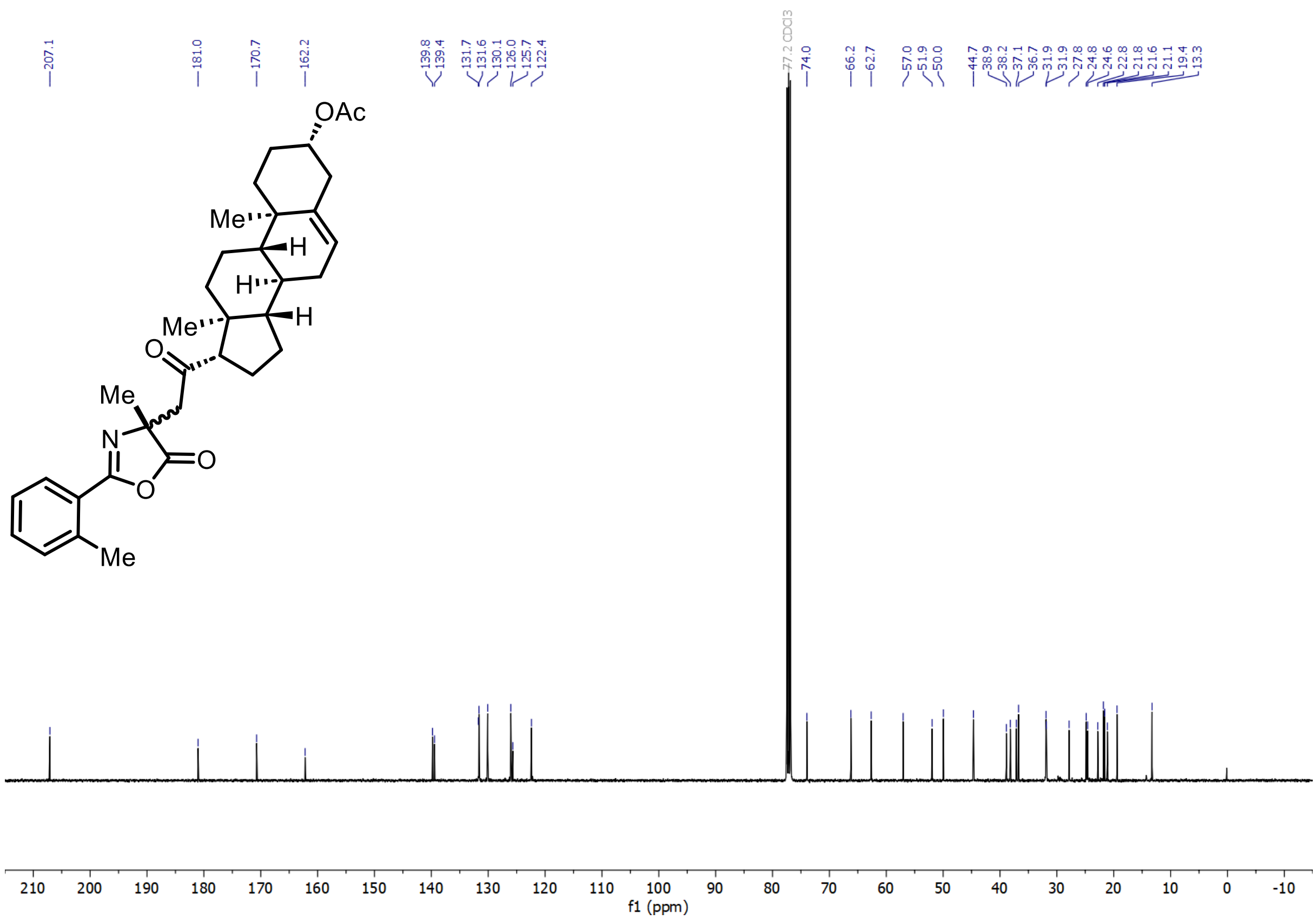

^{13}C NMR spectra of **10h*b*.**

M.5. NMR spectra of products 12 and 14

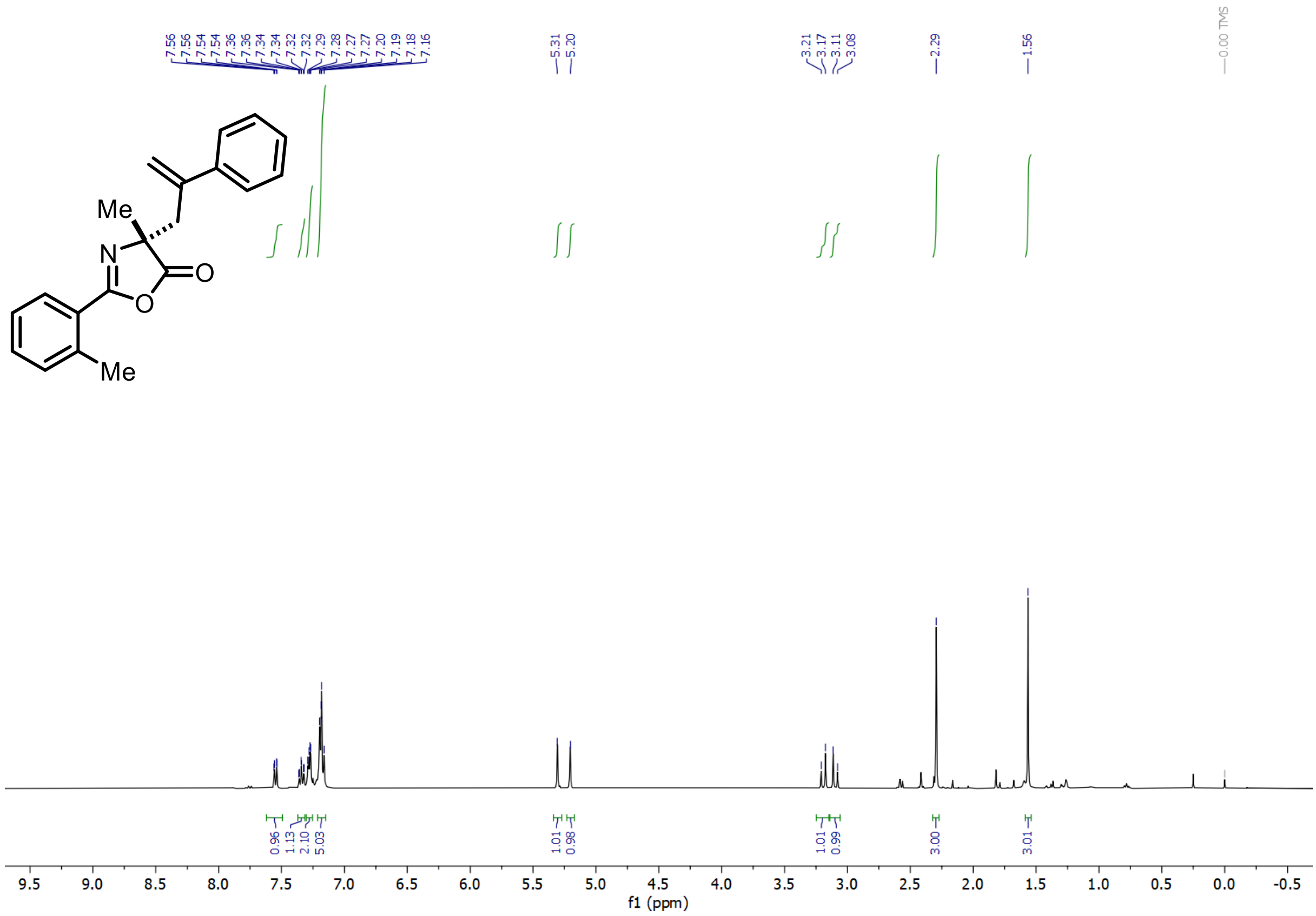

^{1}H NMR spectra of **12**.

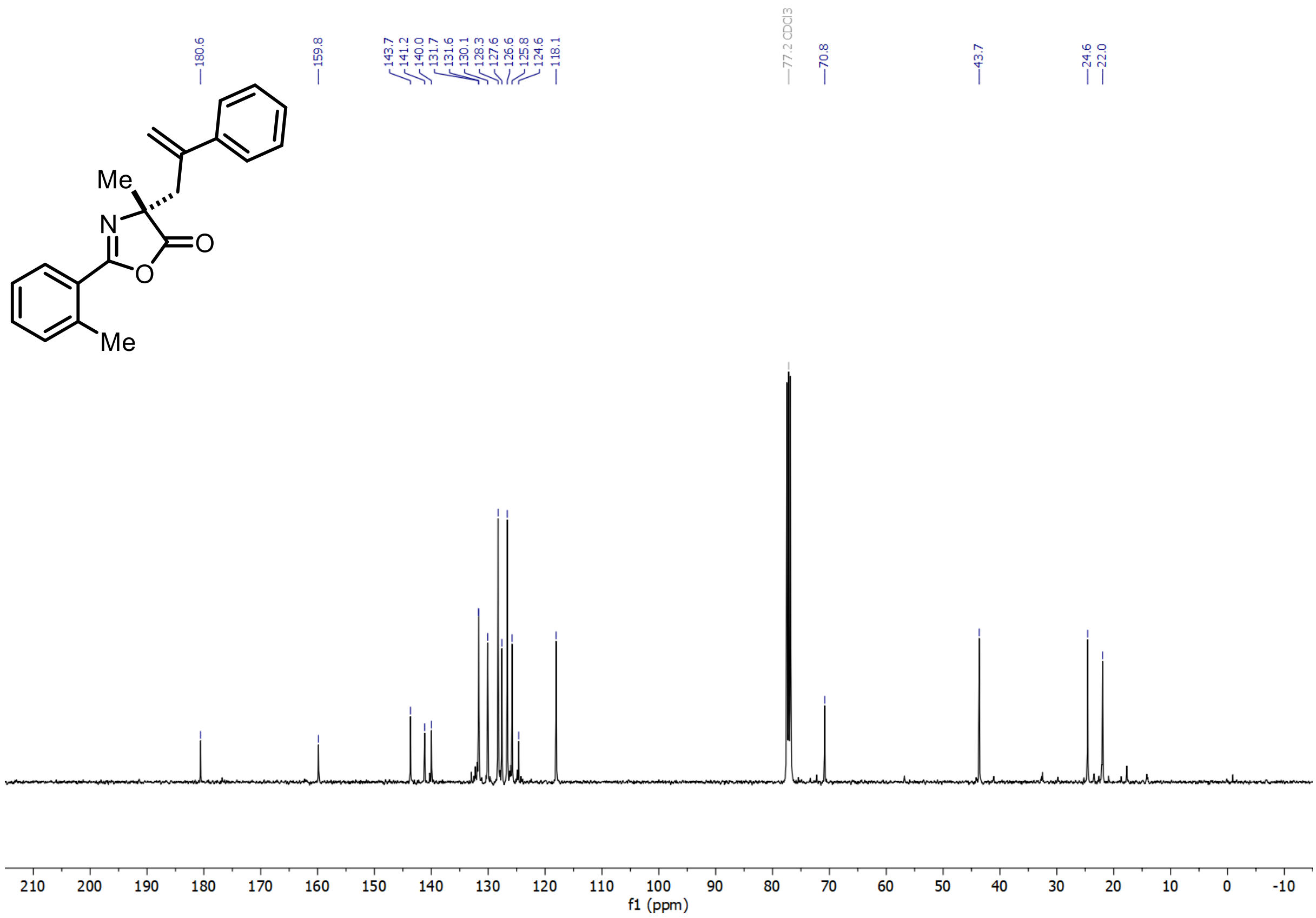

^{13}C NMR spectra of **12.**

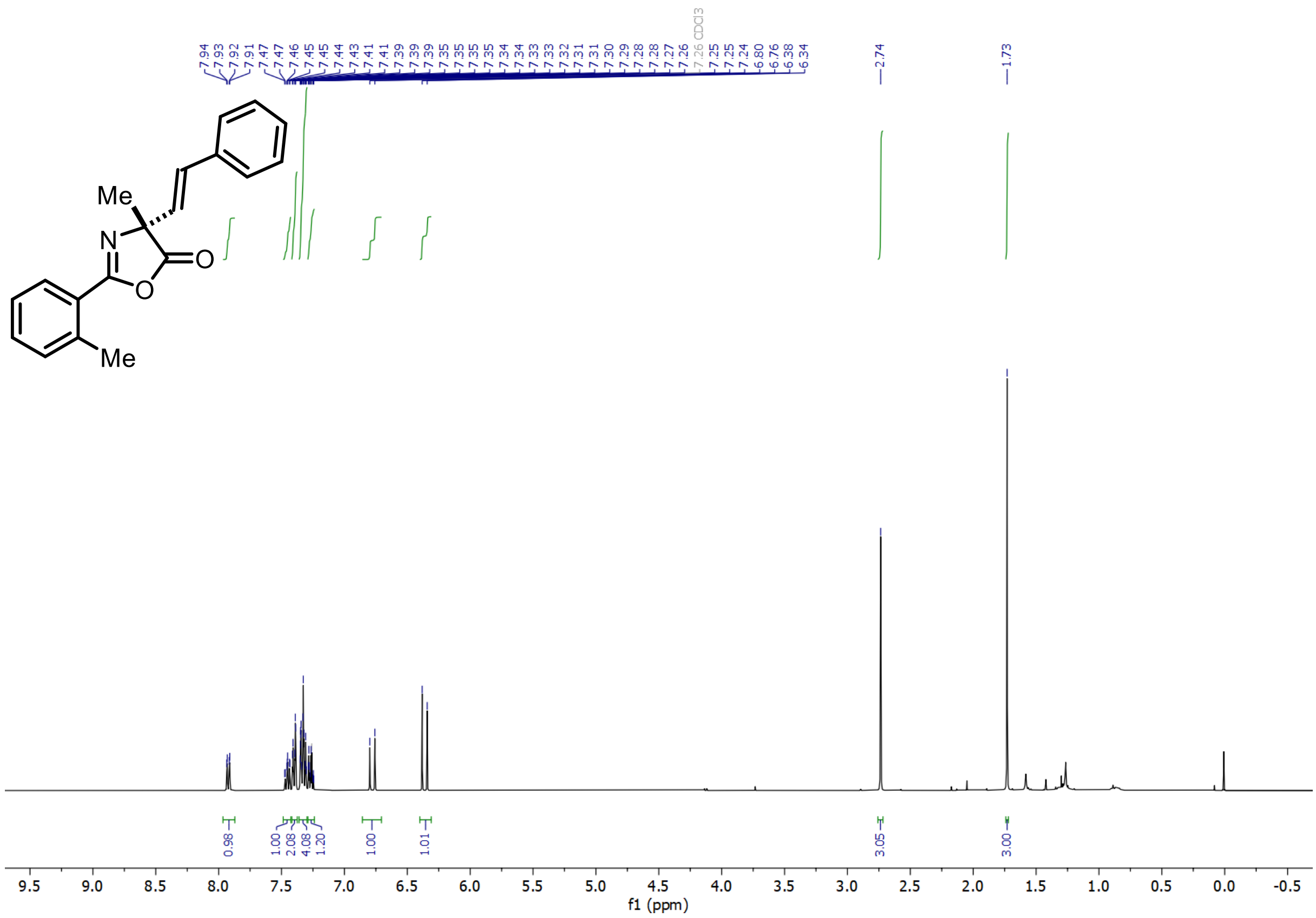

^{1}H NMR spectra of **14**.

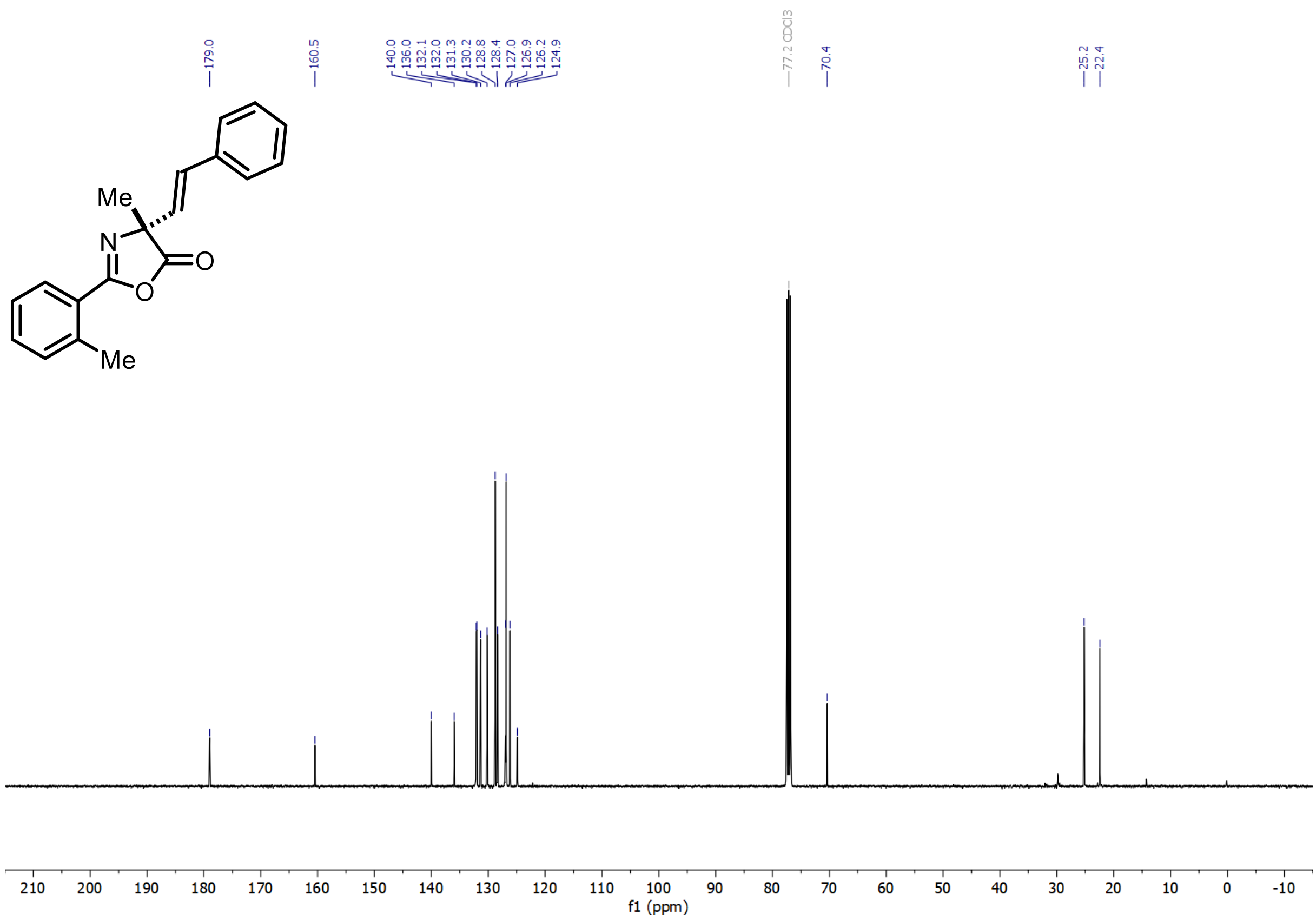

^{13}C NMR spectra of **14.**

M.6. NMR spectra of derivatisation products

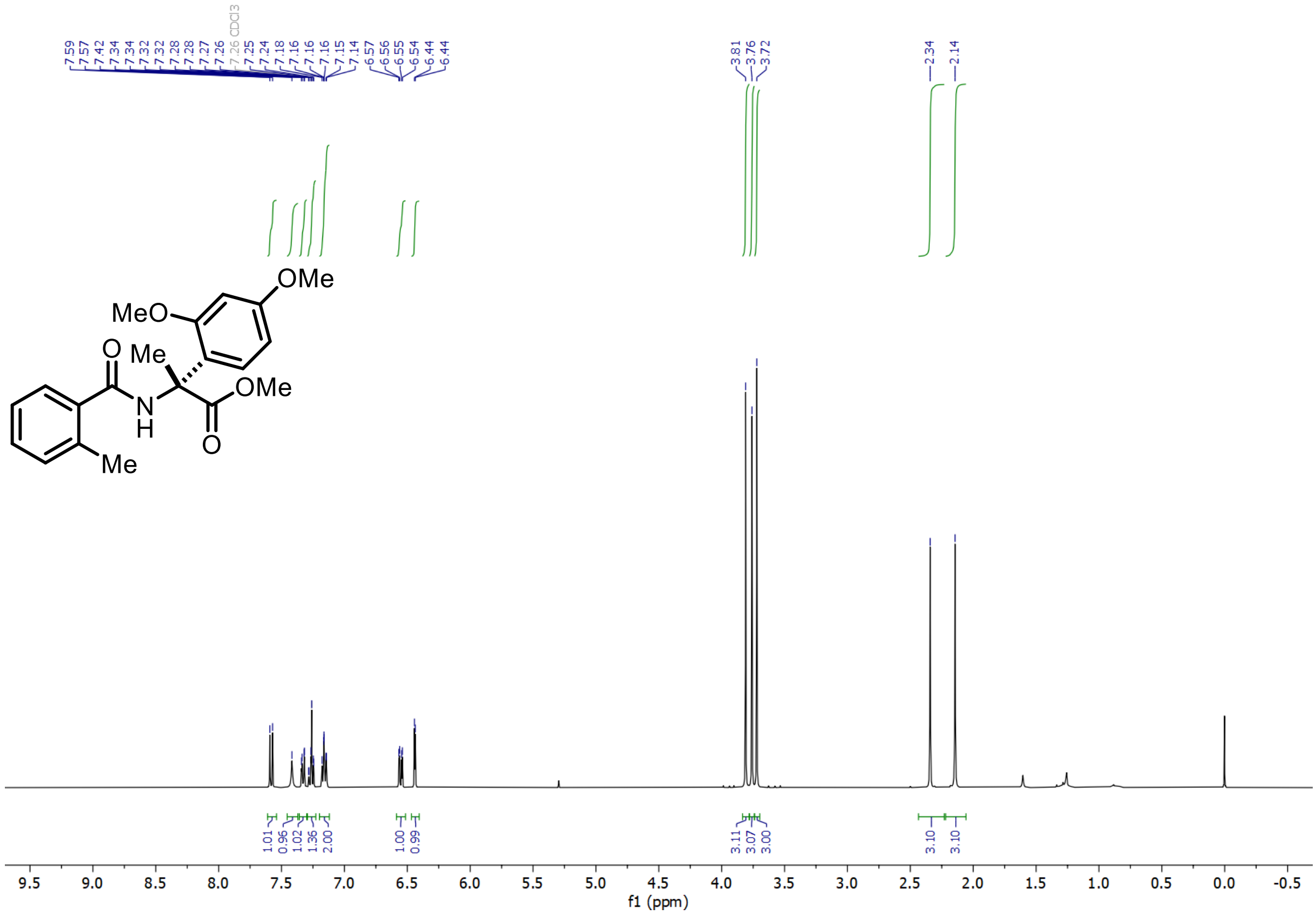

^{1}H NMR spectra of **15**.

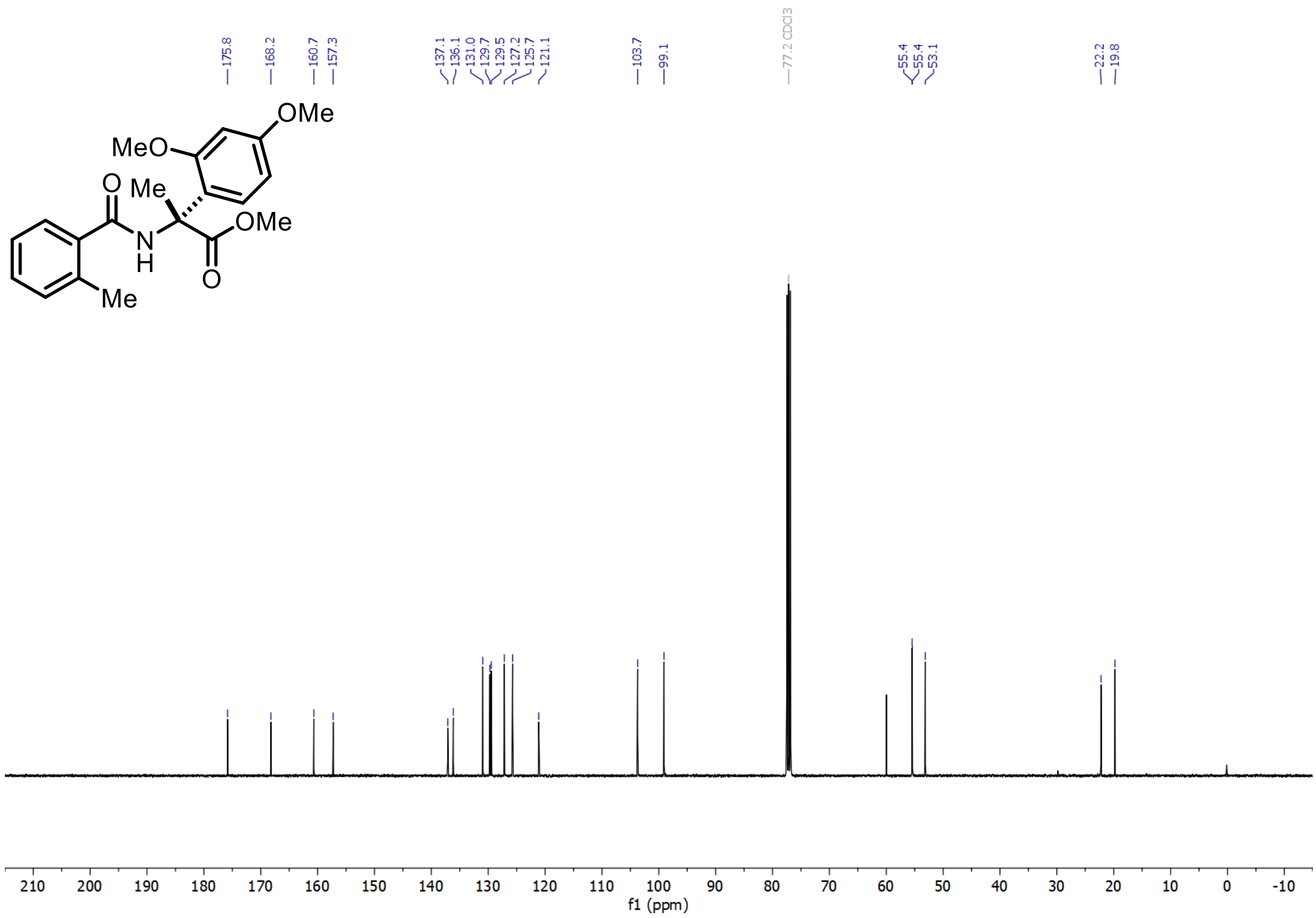

^{13}C NMR spectra of **15.**

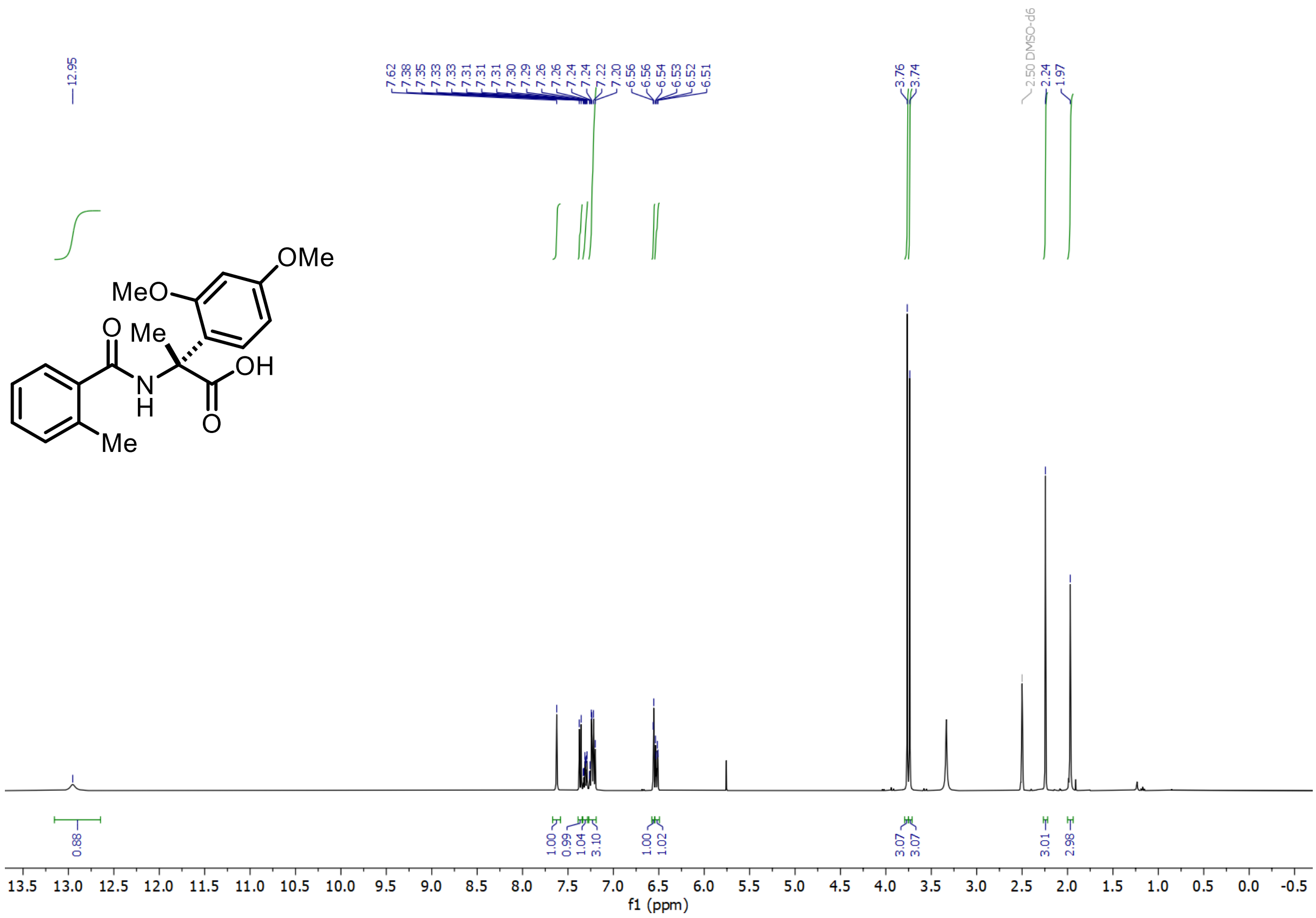

^{1}H NMR spectra of **16**.

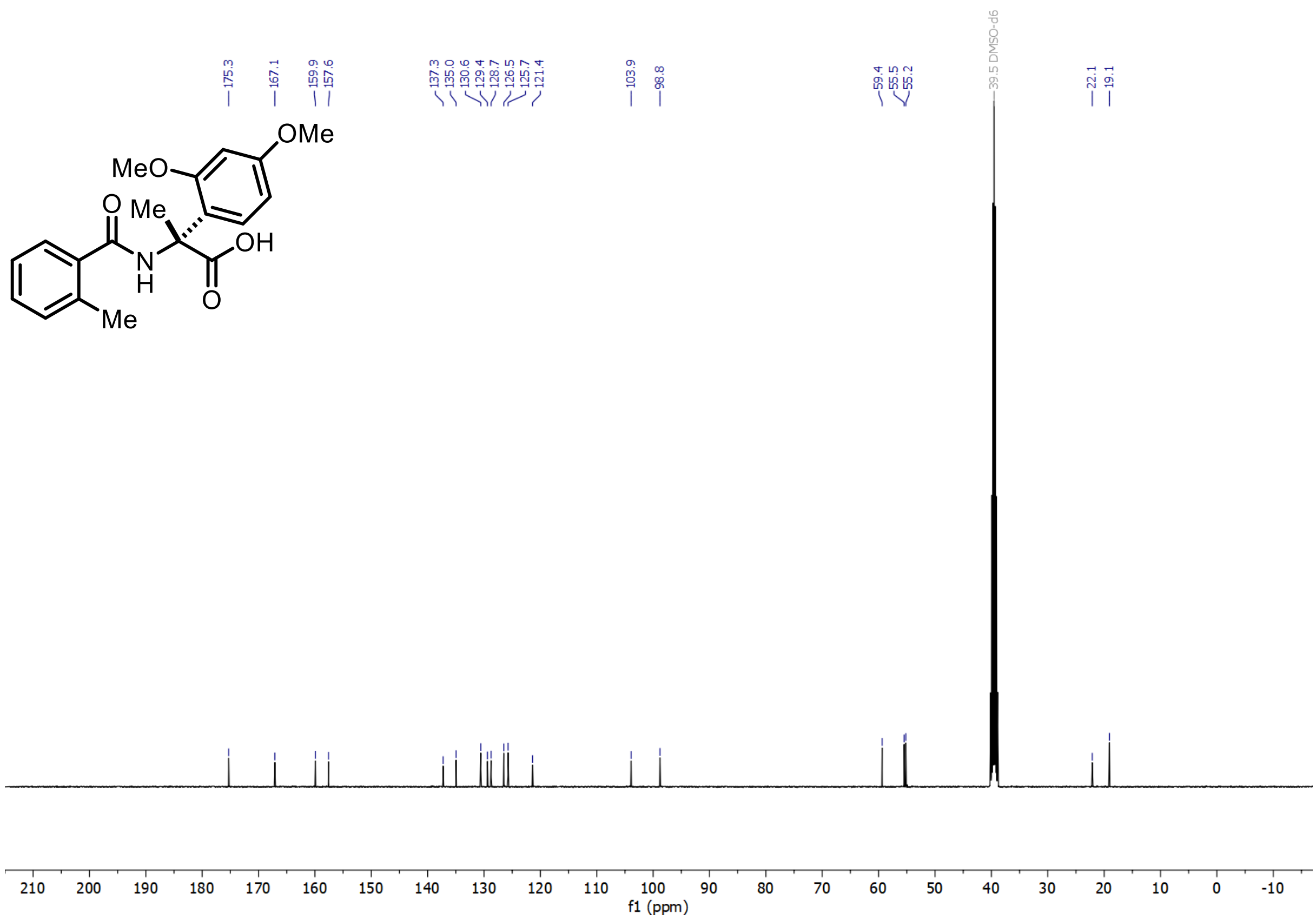

^{13}C NMR spectra of **16.**

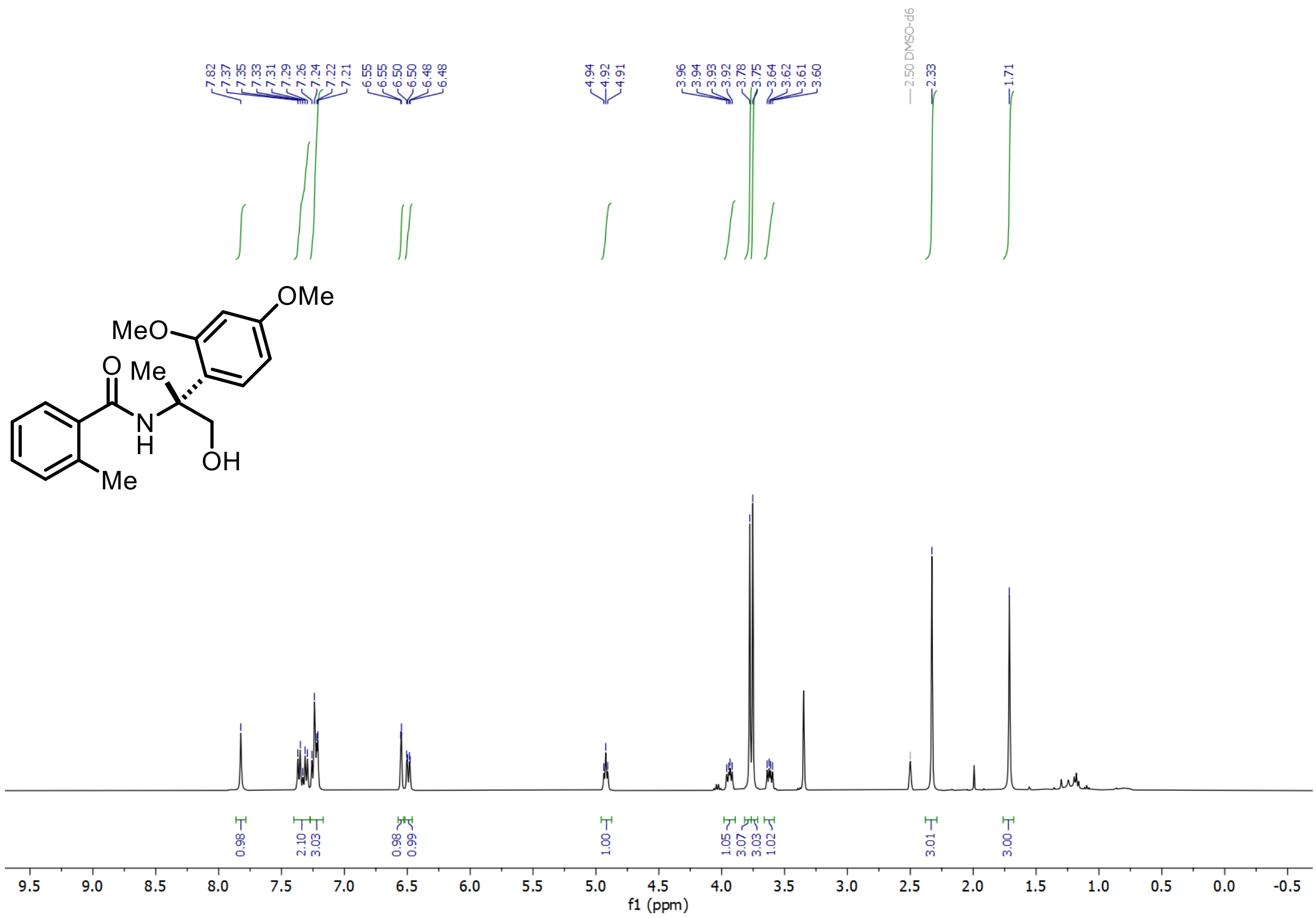

^{1}H NMR spectra of **17**.

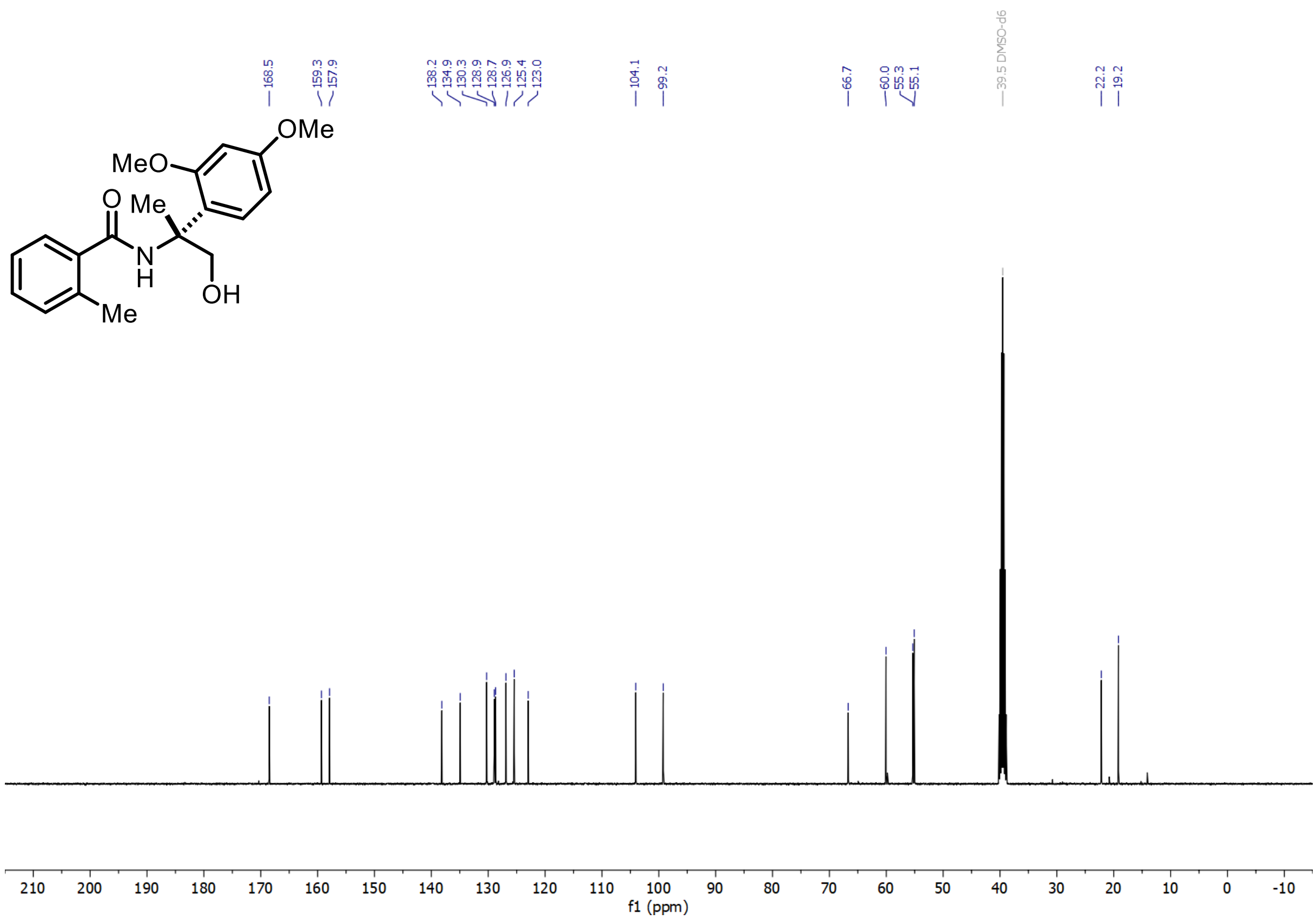

^{13}C NMR spectra of **17.**

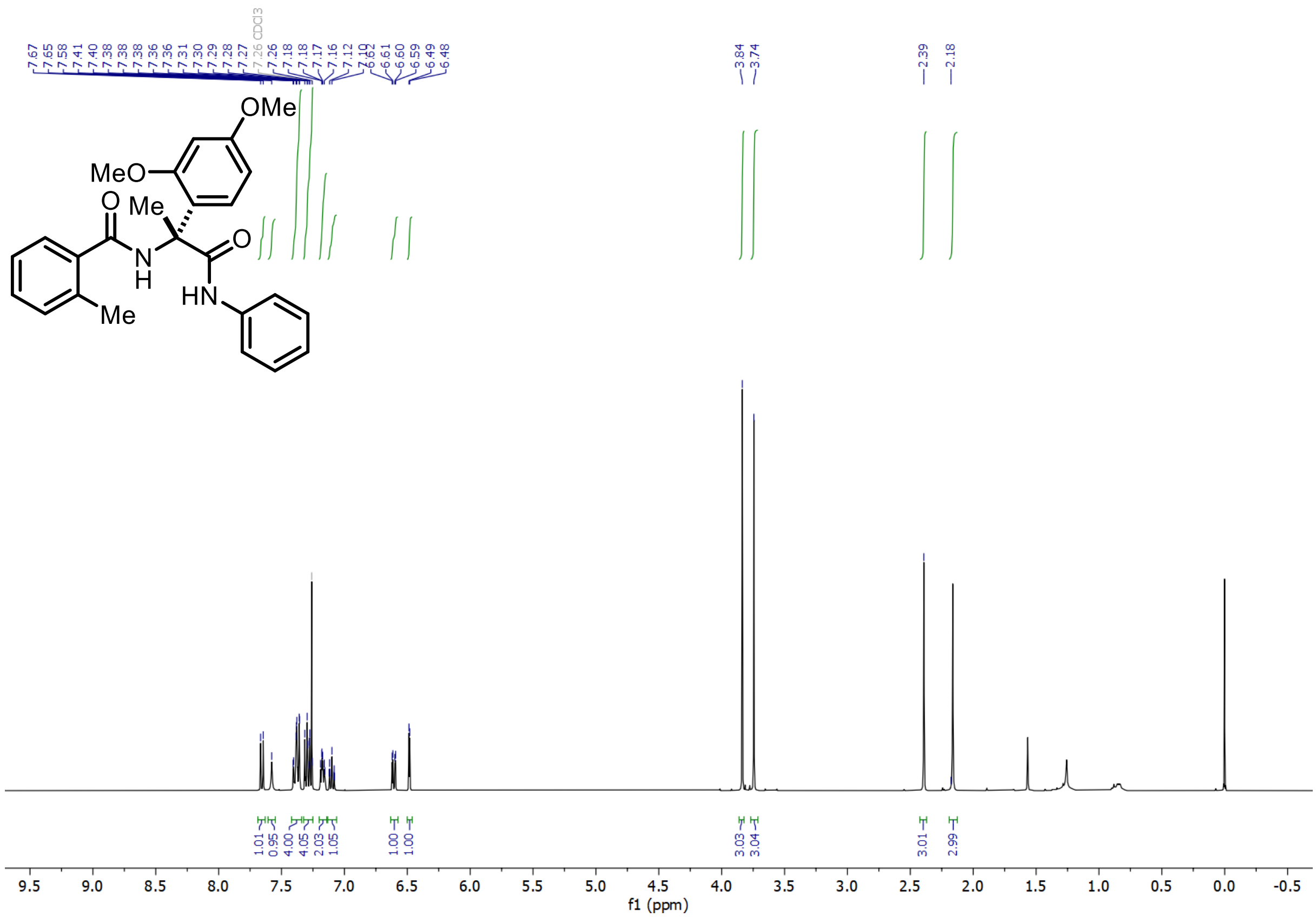

^{1}H NMR spectra of **18**.

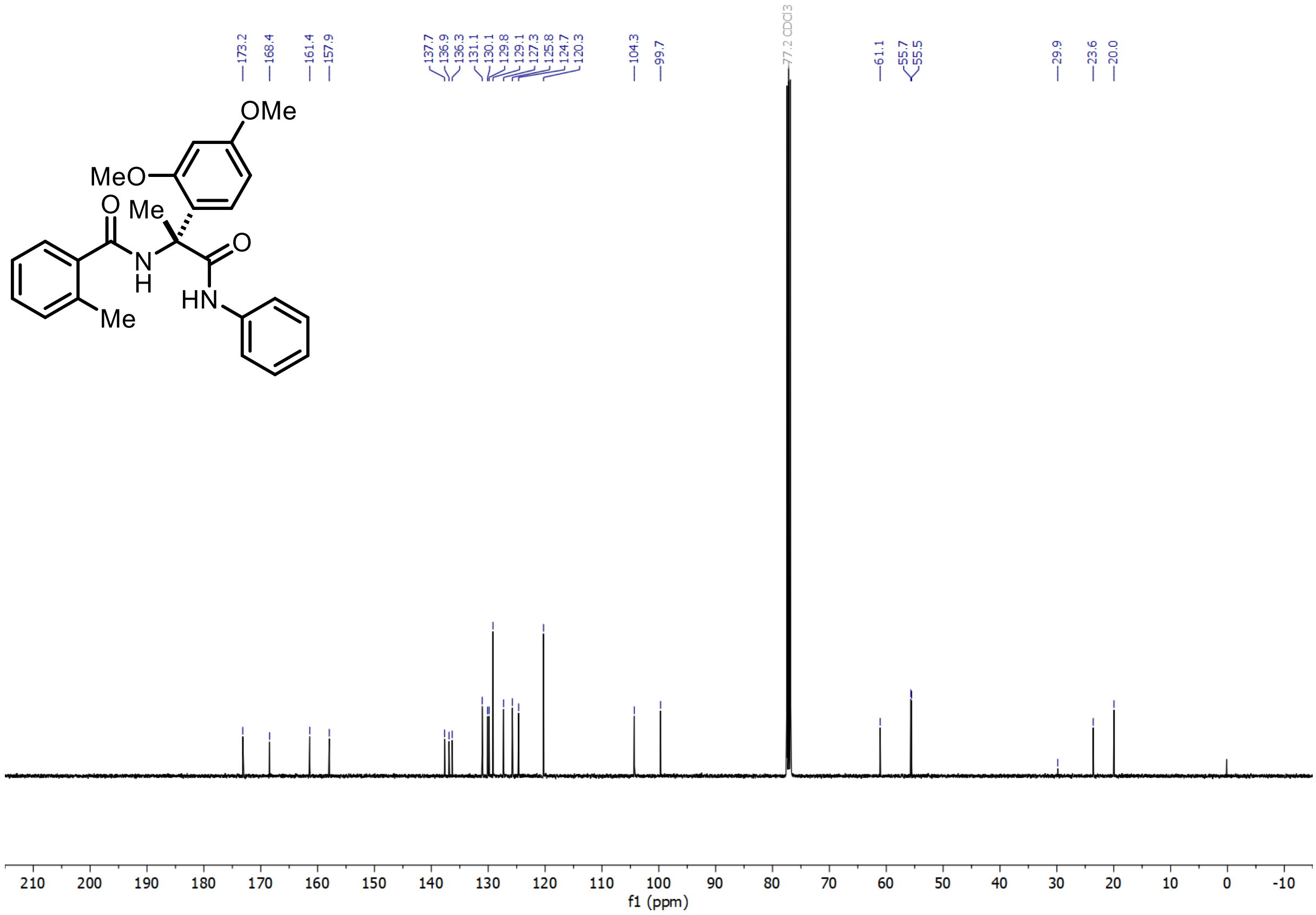

^{13}C NMR spectra of **18.**

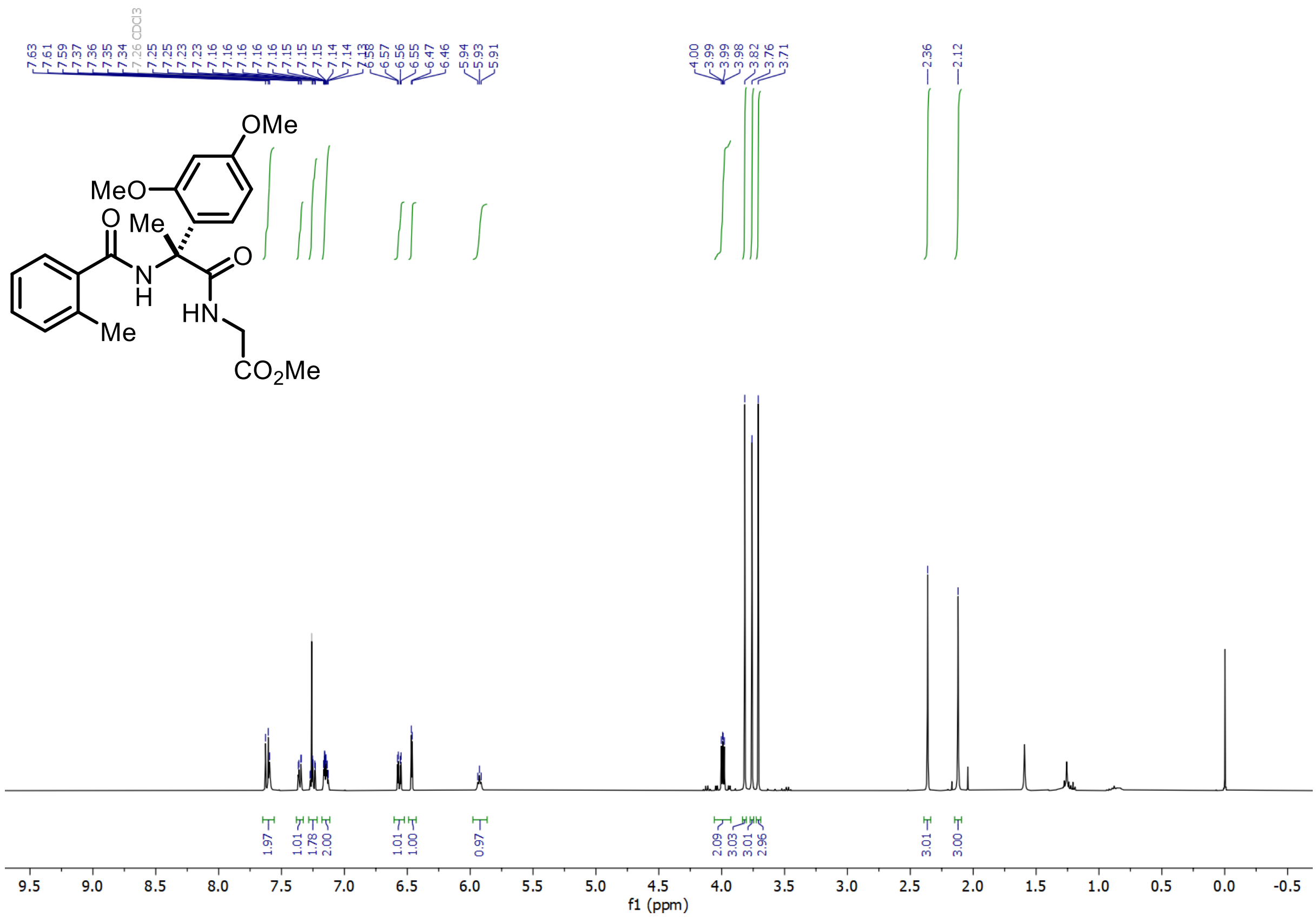

^{1}H NMR spectra of **19**.

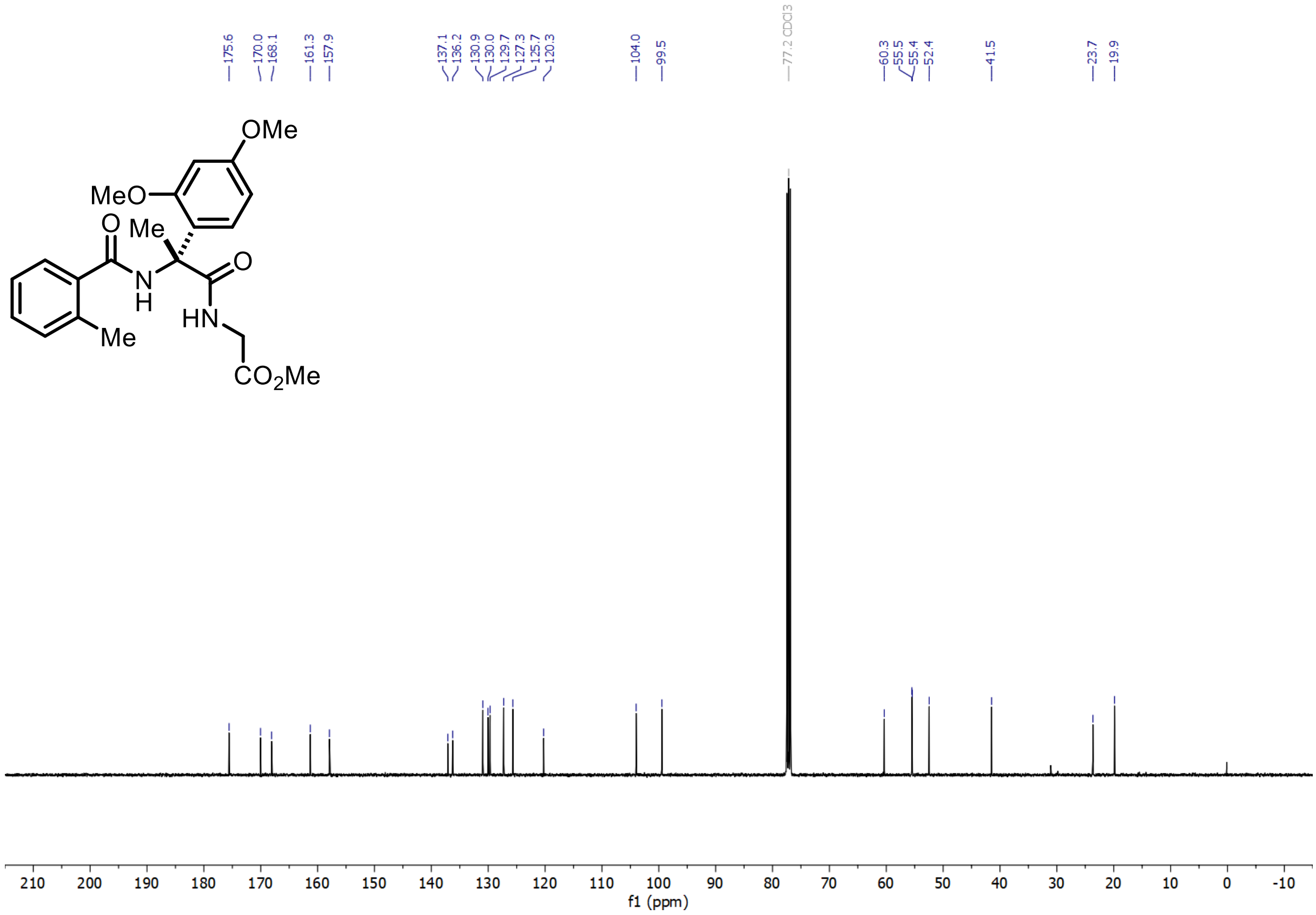

^{13}C NMR spectra of **19.**

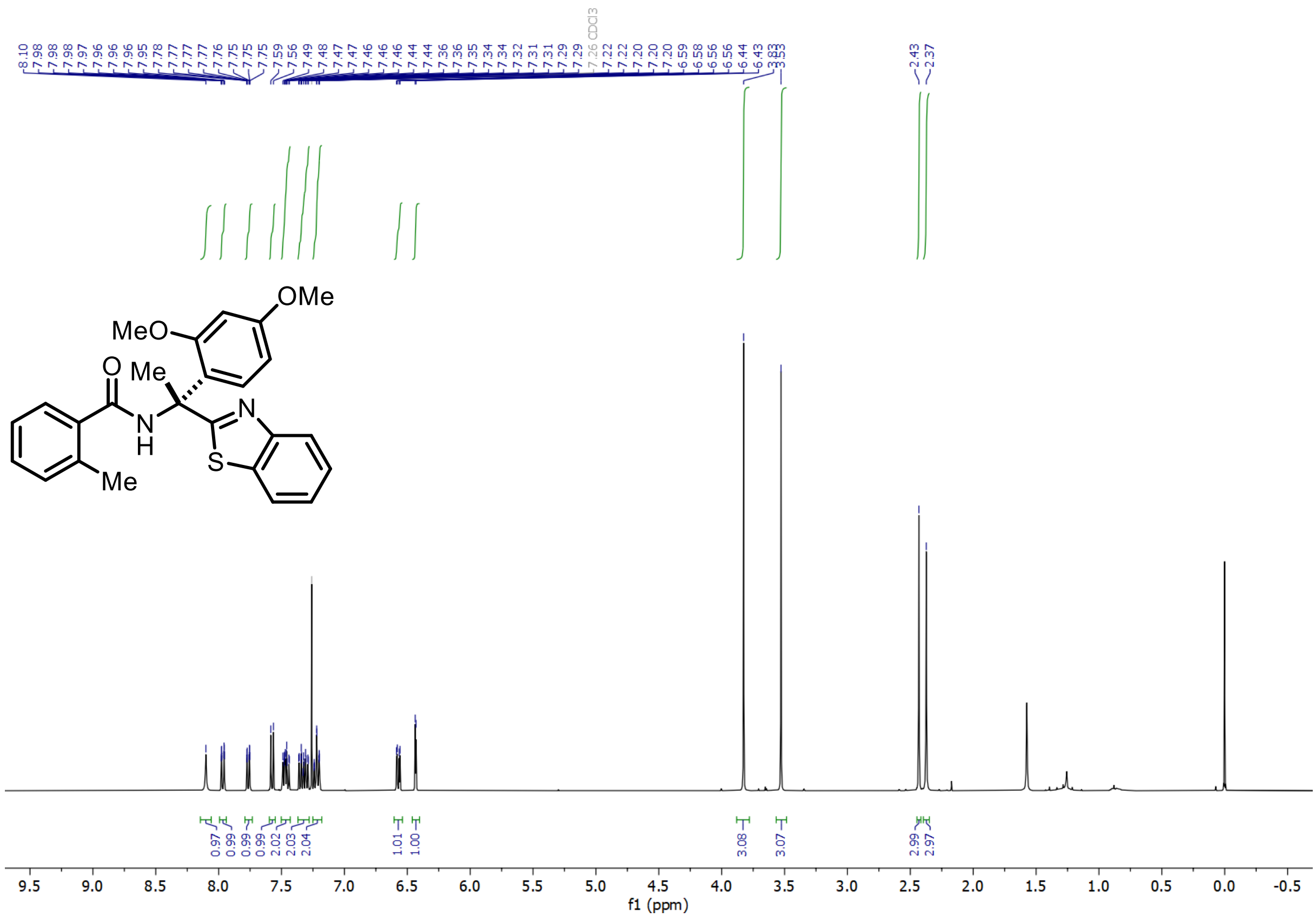

^{1}H NMR spectra of **20**.

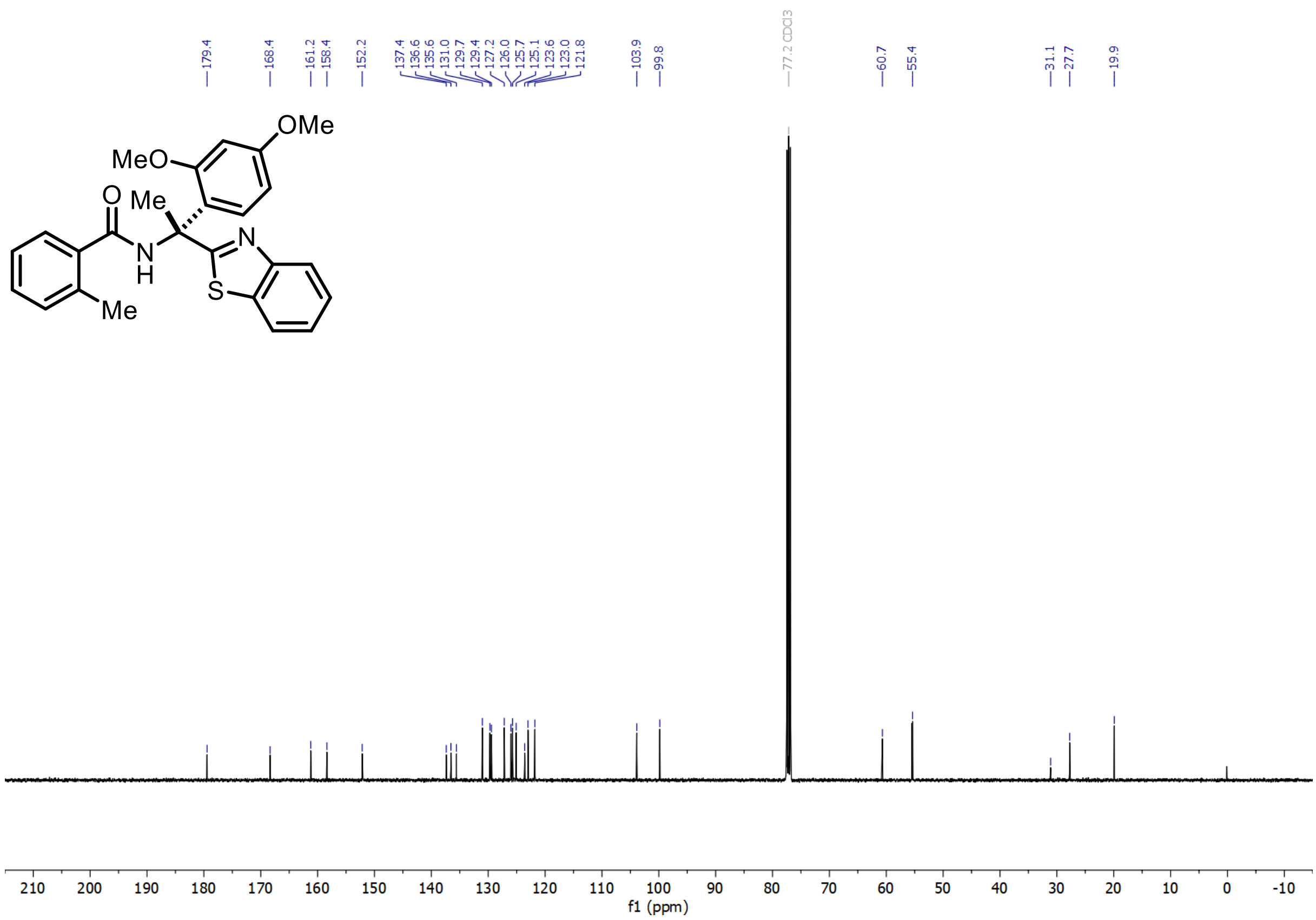

^{13}C NMR spectra of **20.**

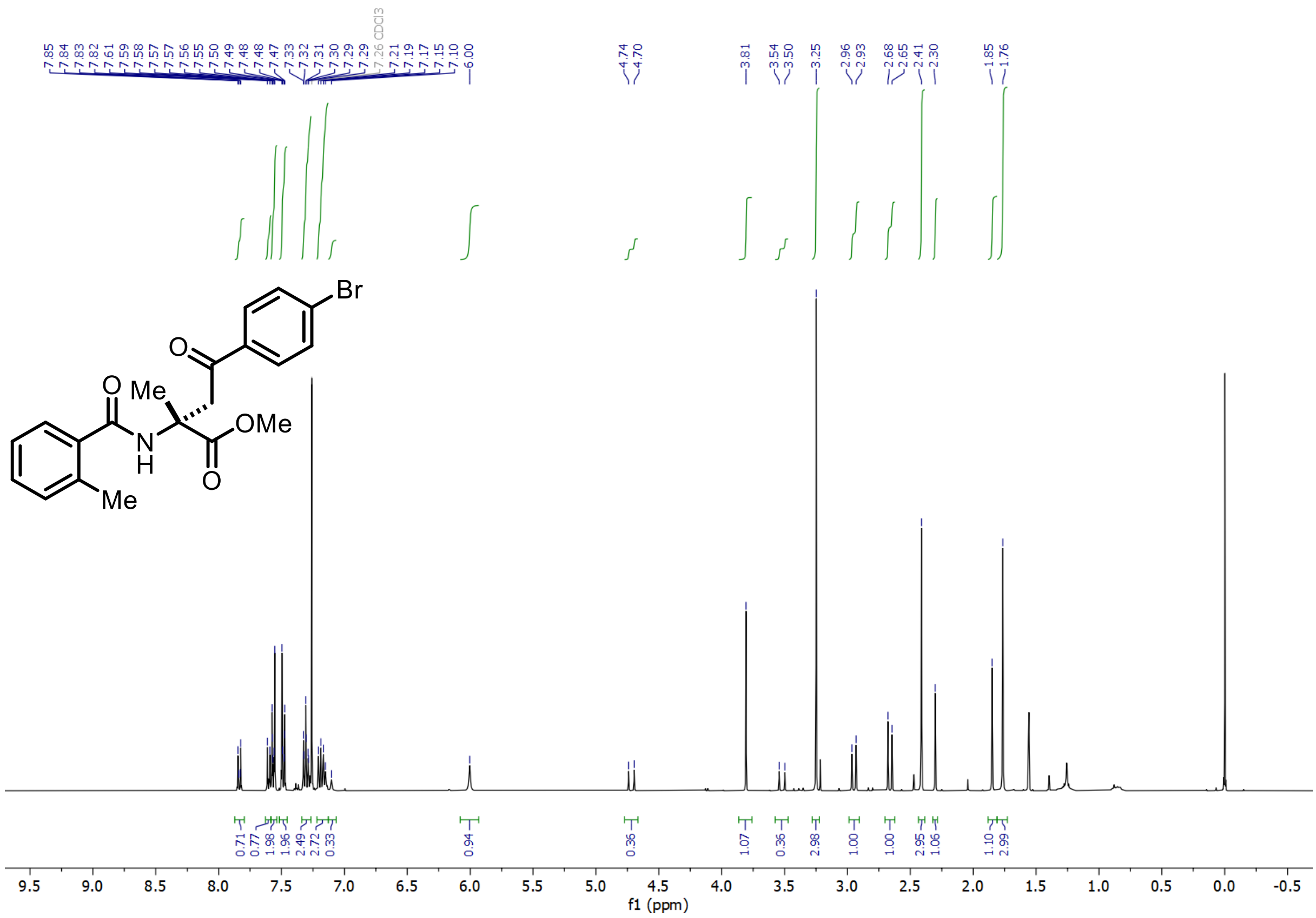

^{1}H NMR spectra of **21**.

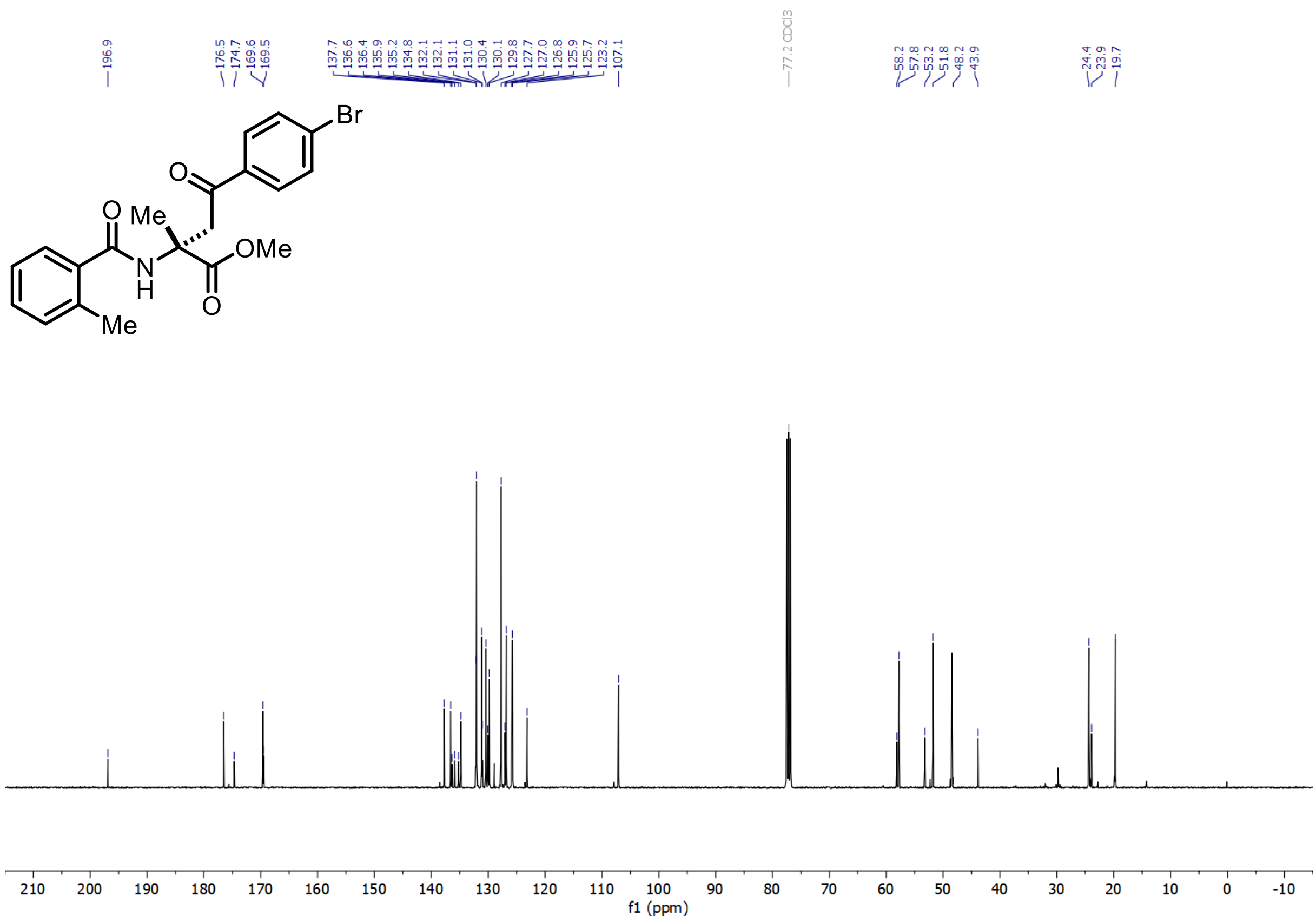

^{13}C NMR spectra of **21.**

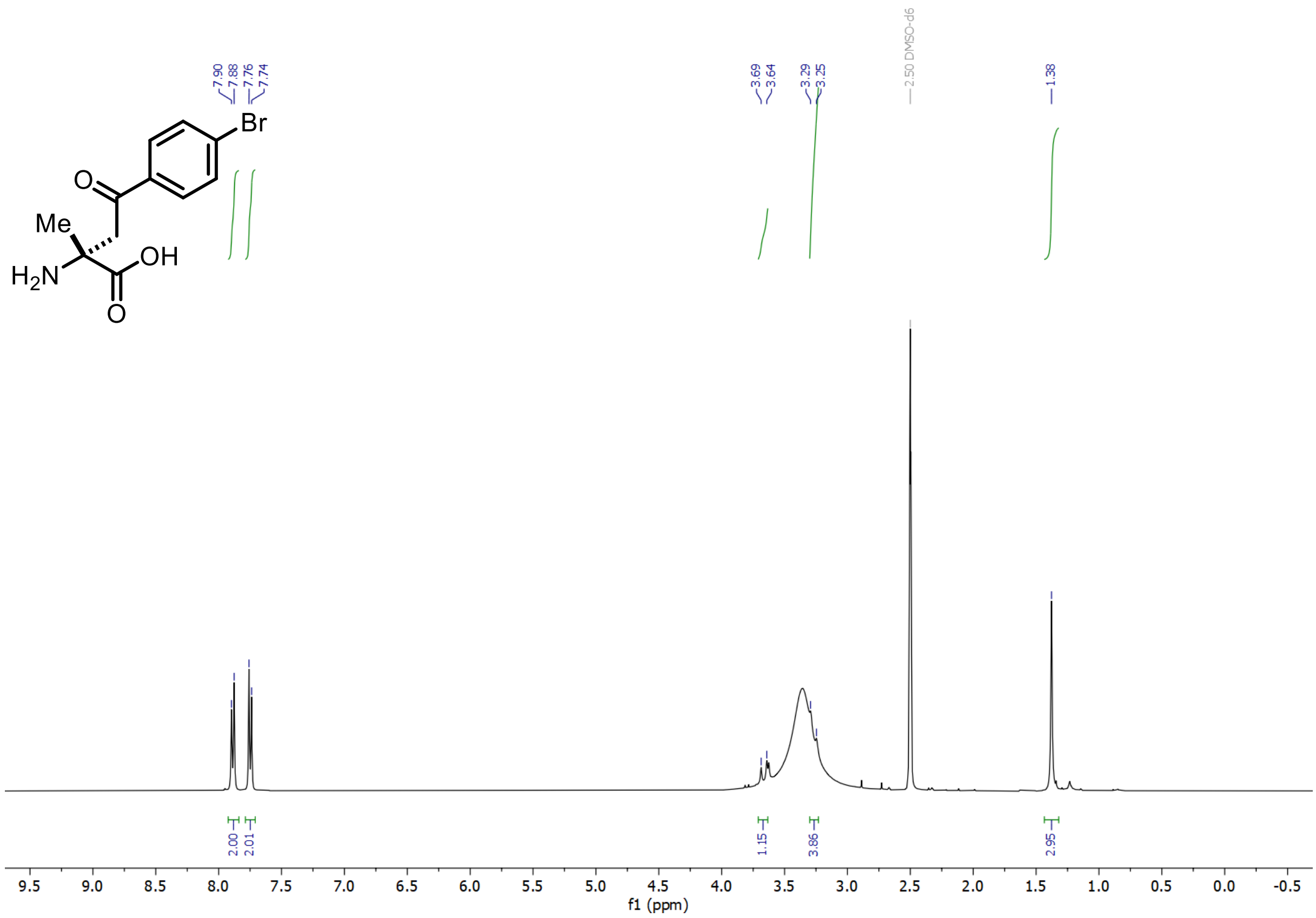

^{1}H NMR spectra of **22**.

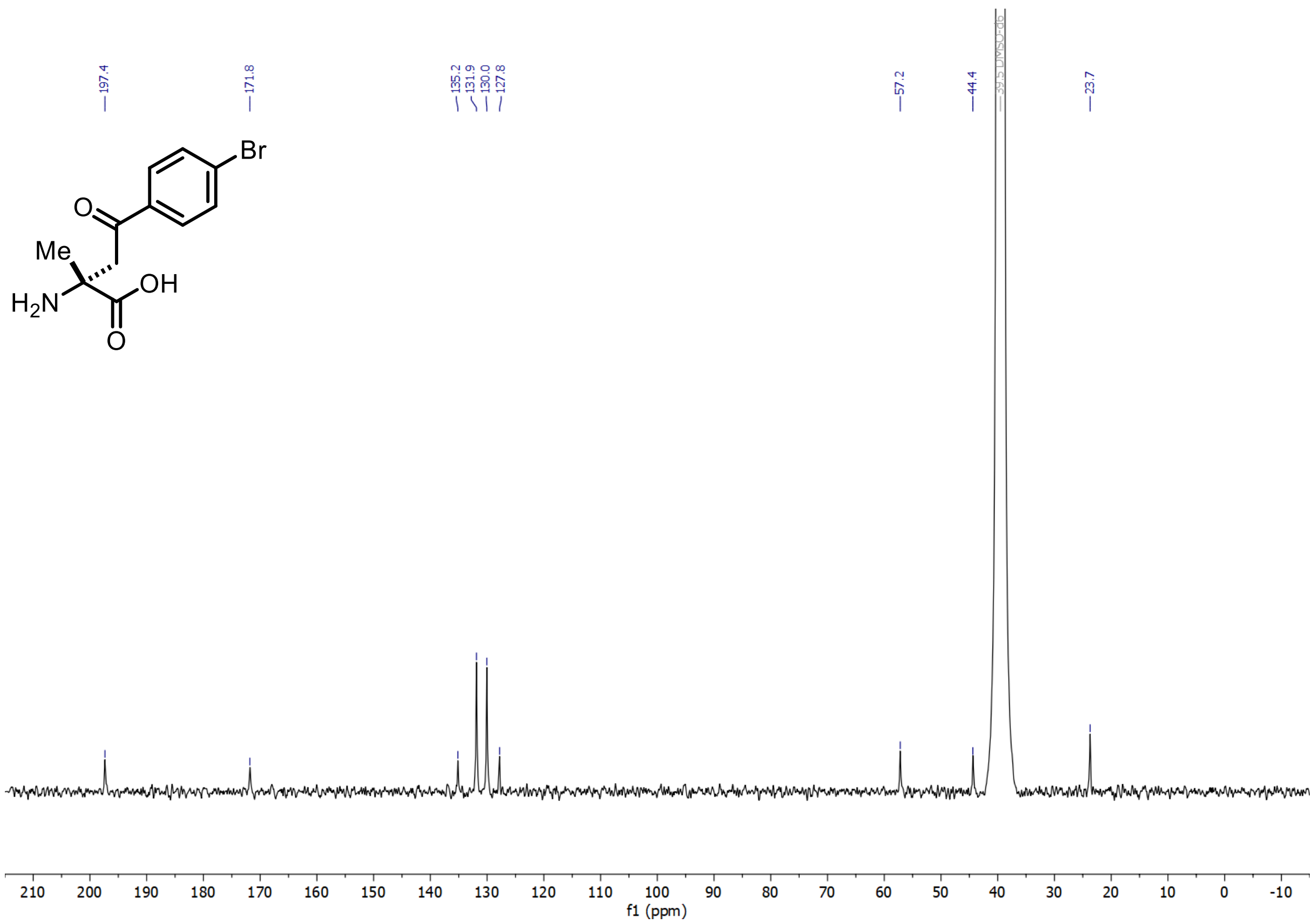

^{13}C NMR spectra of **22.**

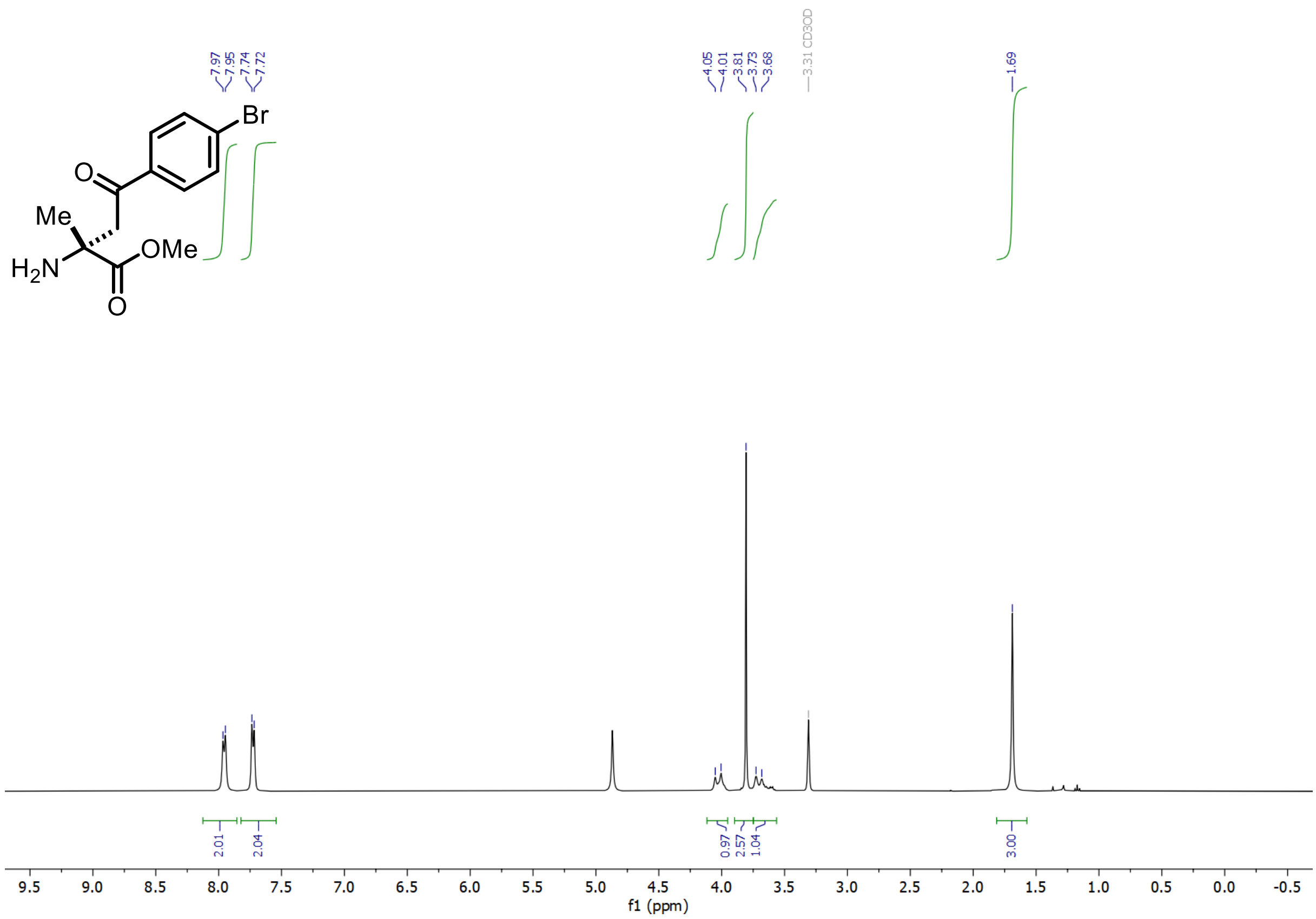

^{1}H NMR spectra of **23**.

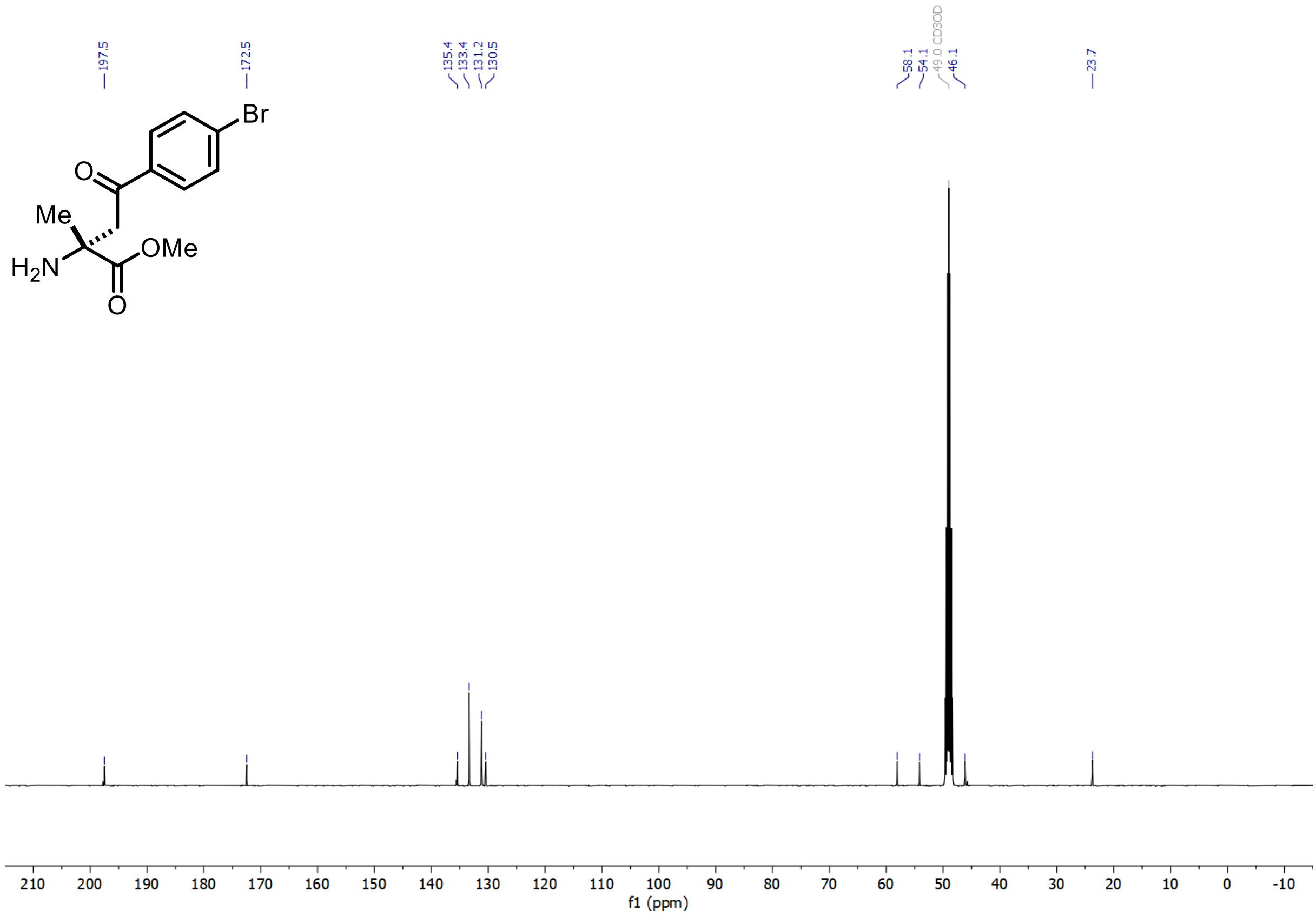

^{13}C NMR spectra of **23.**
